# Diversity of SCC*mec* Elements in *Staphylococcus aureus* as Observed in South-Eastern Germany

**DOI:** 10.1371/journal.pone.0162654

**Published:** 2016-09-20

**Authors:** Stefan Monecke, Lutz Jatzwauk, Elke Müller, Hedda Nitschke, Katharina Pfohl, Peter Slickers, Annett Reissig, Antje Ruppelt-Lorz, Ralf Ehricht

**Affiliations:** 1 Institute for Medical Microbiology and Hygiene (IMMH), Technische Universität Dresden, Dresden, Germany; 2 Department of Hospital Infection Control, Dresden University Hospital, Dresden, Germany; 3 Alere Technologies GmbH, Jena, Germany; 4 Department of Laboratory Medicine, Hospital Dresden Neustadt, Dresden, Germany; 5 InfectoGnostics Research Campus, Jena, Germany; Rockefeller University, UNITED STATES

## Abstract

SCC*mec* elements are very important mobile genetic elements in Staphylococci that carry beta-lactam resistance genes *mecA/mecC*, recombinase genes and a variety of accessory genes. Twelve main types and a couple of variants have yet been described. In addition, there are also other SCC elements harbouring other markers. In order to subtype strains of methicillin-resistant *S*. *aureus* (MRSA) based on variations within their SCC*mec* elements, 86 markers were selected from published SCC sequences for an assay based on multiplexed primer extension reactions followed by hybridisation to the specific probes. These included *mecA/mecC*, *fusC*, regulatory genes, recombinase genes, genes from ACME and heavy metal resistance loci as well as several genes of unknown function. Hybridisation patterns for published genome or SCC sequences were theoretically predicted. For validation of the microarray based assay and for stringent hybridisation protocol optimization, real hybridization experiments with fully sequenced reference strains were performed modifying protocols until yielded the results were in concordance to the theoretical predictions. Subsequently, 226 clinical isolates from two hospitals in the city of Dresden, Germany, were characterised in detail. Beside previously described types and subtypes, a wide variety of additional SCC types or subtypes and pseudoSCC elements were observed as well as numerous composite elements. Within the study collection, 61 different such elements have been identified. Since hybridisation cannot recognise the localisation of target genes, gene duplications or inversions, this is a rather conservative estimate. Interestingly, some widespread epidemic strains engulf distinct variants with different SCC*mec* subtypes. Notable examples are ST239-MRSA-III, CC5-, CC22-, CC30-, and CC45-MRSA-IV or CC398-MRSA-V. Conversely, identical SCC elements were observed in different strains with SCC*mec* IVa being spread among the highest number of Clonal Complexes. The proposed microarray can help to distinguish isolates that appear similar or identical by other typing methods and it can be used as high-throughput screening tool for the detection of putative new SCC types or variants that warrant further investigation and sequencing. The high degree of diversity of SCC elements even within so-called strains could be helpful for epidemiological typing. It also raises the question on scale and speed of the evolution of SCC elements.

## Introduction

Methicillin-resistant *Staphylococcus aureus* (MRSA) is one of the major pathogens in hospitals and the community. MRSA is not only resistant to methicillin (which serves as indicator for this phenotype) but against all beta-lactam antibiotics with the two recently developed compounds (ceftobipirole and ceftaroline) being notable exceptions. Resistance is caused by a modified penicillin binding protein, PBP2a, which is encoded by alleles [1] of the gene *mecA*. In 2011, a second gene, *mecC* has been discovered that also causes methicillin/beta-lactam resistance [2,3]. Both genes are situated on large, potentially mobile genetic elements, so-called SCC*mec* elements (staphylococcal cassette chromosome *mec*). These elements also harbour regulatory genes, recombinase genes and a variety of accessory genes. Twelve different types of SCC*mec* elements have so far been described ([4,5,6,7,8]; http://www.sccmec.org/Pages/SCC_TypesEN.html). Their nomenclature relies on the identity of the *mec* complex, i.e., the immediate surroundings of *mecA*, including its regulatory genes, and on the identity of the recombinase gene (*ccr*) complex [6]. Furthermore, there are the so-called J-regions (“joining” or “junkyard” region) that might include a variety of other genes, including additional resistance or virulence determinants. Due to variations within the J-regions some SCC*mec* types can further be differentiated into subtypes.

Truncated SCC*mec* elements lacking *ccr* recombinase genes are known as pseudo-SCC*mec* elements [7].

In addition to SCC*mec* elements, a variety of different SCC elements have been described and/or sequenced that might lack the *mecA/mecC* genes but that carry a fusidic acid resistance marker *fusC* [9], various heavy metal resistance genes or other genes such as the arginine catabolic mobile element (ACME) or a high-affinity ATP-driven potassium transport system catalysing the hydrolysis of ATP coupled with the exchange of hydrogen and potassium ions (*kdp* locus). Their presence suggests that SCC elements as a system facilitating horizontal gene transfer between staphylococci predates the emergence of SCC*mec* elements, and that *mecA/C* genes could be regarded as just one “payload” for SCC elements among others.

Since MRSA are associated with high morbidity and mortality, rapid molecular tests without culture would be useful for infection control and timely guidance of treatment. SCC elements and *mecA* can also be found in other, clinically less relevant staphylococci, so that a mere PCR for the detection of *mecA* from a patient sample is not sufficient to diagnose the presence of MRSA. Additional markers need to be detected to prove that *mecA* was present in *S*. *aureus* and thus to ensure discrimination of MRSA from possibly colonising methicillin-resistant “coagulase-negatives”. Integration sites of SCC*mec* elements can be targeted for that purpose designing molecular tests in which one primer detects the species-specific sequences within the core genome while the other one aims on a primer-binding site within the SCC*mec* element. However, it is necessary to ensure to identify and to cover all relevant alleles of a potential primer-binding site in order to avoid false negatives. The constant evolution of MRSA and the emergence and/or geographic spread of new strains that could displace and marginalise previously epidemic strains requires close monitoring of these trends and a constant adaption of molecular tests because otherwise a decreasing performance of said tests is to be expected.

Another reason for studying variability of SCC*mec* elements could be their use for high-resolution typing purposes. Many strains that share more or less the same core genome (and thus yield identical *spa* and MLST types) differ in SCC*mec* elements. Detecting more SCC*mec*-related markers could allow a higher degree of discrimination and might be helpful especially for subtyping abundant and widespread strains.

For these reasons, a DNA hybridisation array was designed that in addition to a previously characterised system, facilitates detection of a total of 83 SCC*mec*-related markers, which were previously shown to be situated in SCC*mec* elements. It was used for validation with reference strains of known genome sequences as well as for characterisation of a collection of clinical isolates collected within 15 years at two primary care hospitals in the city of Dresden in Germany.

## Material and Methods

### Strain collection

The study was performed at a tertiary care hospital in Dresden, Saxony, *i*.*e*., in South-Eastern Germany. The hospital has approximately 1,200 beds and treats 57,000 in-patients per year (https://www.uniklinikum-dresden.de/de/das-klinikum/jahresberichte/). Isolates were collected routinely from intensive care units, diabetological or surgical wards, suspected transmissions or because of symptoms suggesting PVL-associated disease [10]. Approximately 1,300 isolates collected between 2000 and 2015/2016 were thus characterised using the previously described [11,12] arrays allowing assignment to clonal complexes, epidemic strains and main SCC*mec* types (Table 1). Additional isolates were obtained from another, secondary care hospital in the same city (http://www.khdn.de/). Here, no systematic typing was performed. Isolates were collected because of conspicuous susceptibility tests, clinical conditions or travel history, and they were typed using the same methods [11,12].

**Table 1:** Clonal complexes, strains and SCC elements as identified by array hybridisation. Prevalence data (percentages and absolute numbers) refer to routine MRSA typing from the Dresden University Hospital, 2000—April 2016 (n = 1277).

CC	Strain	Prevalence data	SCC*mec* subtypes	Number of isolates subtyped
**CC1**	CC1-MSSA-SCC*fus*	*Sporadic*°	*fus*+*tir*+*ccrA/B-*1 (MSSA476)	1
	CC1-MRSA-IV	0.6% (n = 8)	SCC*mec* IVa (MW2)	5
	CC1-MRSA-V (PVL+)	*Sporadic*°°	SCC*mec* V (Bengal Bay)	1
	CC1-MRSA-V+*fus* (PVL+)	*Sporadic*°°	SCC*mec* V+*fus*+*tir*+*ccrA/B-*1*	1
**CC5**	ST228-MRSA-I, “South German EMRSA”	2.9% (n = 37)	SCC*mec* I (COL)	9
		2.0% (n = 26)	PseudoSCC*mec*, class B *mec* complex*	3
	CC5-MRSA-SCC [I+*fus*], “Geraldine Clone”	0.1% (n = 1)	SCC*mec* I+*fus+tirS* (Geraldine Clone)	1
	CC5-MRSA-II, “USA300Rhine-Hesse EMRSA“	20.2% (n = 258)	SCC*mec* II (JH1/JH9)	14
	CC5-MRSA-II+*ccrA/B-*4	0.3% (n = 4)	SCC*mec* II+*czrC*+*ccrA/B-*4*	2
			SCC*mec* II+*speG*+*czrC*+*ccrA/B-*4*	2
	CC5-MRSA-IV, “Paediatric clone”	0.5% (n = 6)	SCC*mec* IVa (MW2)	1
			SCC*mec* IVb/d/i (JCSC1978/6668/4469)	3
			SCC*mec* IVc (IS-105) [merA/B+]	2
	CC5-MRSA-IV (PVL+)	0.2% (n = 2)	SCC*mec* IVc (TCH60)	2
	CC5-MRSA-V	0.1% (n = 1)	SCC*mec* V*	1
	CC5-MRSA-[IV+*ccrA/B-*4]	0.1% (n = 1)	SCC*mec* IVc+*speG*+*ccrA/B-*4 (SA_ST125)	1
**CC6**	CC6-MRSA-IV	0.2% (n = 3)	SCC*mec* IVa (MW2)	3
**CC7**	CC7-MRSA-IV	0.2% (n = 3)	SCC*mec* IVa (MW2)	1
			SCC*mec* IVb/d/i (JCSC1978/6668/4469)	2
	CC7-MRSA-VT	*Sporadic*°°°	SCC*mec* VT (var.1)*	1
	CC7-MRSA-[VI+*fus*]	0.1% (n = 1)	SCC*mec* VI+*fus* (MRSA18)	1
**CC8**	CC8-MSSA with SCC elements	*Sporadic*°	*speG+czrC+ccrA/B*-4*	3
			ACME II*	3
			ACME II+*speG*+*czrC*+*ccrA/B-*4*	3
	ST247-MRSA-I, “North German EMRSA”	0.2% (n = 2)	SCC*mec* I (PSP1996)	1
	ST8-MRSA-IIA/B/D, “Irish AR13/14”	*Sporadic*°°°	SCC*mec* IIA/B/D without *ccrA/B-*4 (Irish AR13/14)	1
	CC8-MRSA-IV, “UK-EMRSA-14”	0.2% (n = 2)	SCC*mec* IVc (TCH60)	1
			SCC*mec* IVh/j (HO50960412/JCSC6670)	1
	CC8-MRSA-IV, “Lyon Clone”	0.4% (n = 5)	SCC*mec* IVc (TCH60)	1
			SCC*mec* IVc (IS-105)	1
	CC8-MRSA-IV, “USA500”	0.2% (n = 3)	SCC*mec* IVa*	1
			SCC*mec* IVb/d/i (Strain 21209)	2
	ST8-MRSA-[IV+ACME] (PVL+), “USA300”	0.5% (n = 6)	SCC*mec* IVa+ACME1+Cu (USA300-TCH1516)	3
	CC8-MRSA-IV (PVL+), ACME-negative	0.2% (n = 3)	SCC*mec* IVc+Cu/Hg (MRSA177)	3
			SCC*mec* IVa (MW2)	1
		*Sporadic*°°	PseudoSCC*mec*, class B *mec* complex+Cu/Hg*	1
	CC8/ST254-MRSA, “Hannover EMRSA”	0.2% (n = 3)	SCC*mec* IVa+ccrC*	2
		0.5% (n = 7)	PseudoSCC*mec*, class B *mec* complex+Hg*	3
	CC8-MRSA-VT	0.1% (n = 1)	SCC*mec* VT+*czrC* (SO385)	1
**CC22**	CC22-MSSA with SCC elements	*Sporadic*°	*speG+czrC+ccrA/B*-4*	6
			*arsB+ccrA/B-*4*	1
	CC22-MRSA-IV, “UK-15/Barnim EMRSA”	48.6% (n = 621)	SCC*mec* IVh/j (HO50960412/JCSC6670)	16
			SCC*mec* IVa (CMFT503)	1
			SCC*mec* IVc (TCH60)	1
			SCC*mec* IVc (IS-105)	1
	CC22-MRSA-IV (*tst1*+), “Gaza EMRSA”	0.2% (n = 2)	SCC*mec* IVa (CMFT503)	2
	CC22-MRSA-IV (PVL+)	0.4% (n = 5)	SCC*mec* IVh/j (HO50960412/JCSC6670)	3
			SCC*mec* IVc (IS-105)	1
			SCC*mec* IVa (MW2)	1
	CC22-MRSA-[IV+ACME]	0.1% (n = 1)	SCC*mec* IVh/j+ACME2 (M08-0126)	1
	CC22-MRSA with SCC*mec*IV/composite elements	0.3% (n = 4)	SCC*mec* IV+*speG*+*czrC*+*ccrA/B-*4 (var.1)*	2
			SCC*mec* IV+*speG*+*czrC*+*ccrA/B-*4 (var.2)*	1
			SCC*mec* IV+*speG*+Cu/*czrC*+*ccrA/B-*4*	1
	CC22-MRSA with SCC*mec*V/composite elements	*Sporadic*°°	SCC*mec* V+speG+czrC+ccrA/B-4*	1
	CC22-MRSA-[V+*fus*]	0.3% (n = 4)	SCC*mec* VT+*fus*+czrC*	3
**CC30**	ST36/39-MRSA-II, “UK-EMRSA-16”	0.1% (n = 1)	SCC*mec* II (N315/Mu50)	1
	CC30-MRSA-IV (PVL+),	0.2% (n = 3)	SCC*mec* IVa (MW2)	7
	“Southwest Pacific /WSPP”		SCC*mec* IVa (H131520133)	1
			SCC*mec* IVb/d/i+Cu*	1
	CC30-MRSA-VT	0.2% (n = 3)	SCC*mec* VT (var.2)*	1
**CC45**	CC45-MRSA-IV (*aphA3/sat*+), „Berlin EMRSA“	13.7% (n = 175)	SCC*mec* IVa (MW2)	12
	CC45-MRSA-IV (*aphA3/sat* -), „Berlin EMRSA“	1.3% (n = 17)	SCC*mec* IVa (MW2)	5
			SCC*mec* IVb/d/i (JCSC1978/6668/4469)	2
			SCC*mec* IVc (TCH60)	4
	CC45-MRSA-IV+ACME	0.1% (n = 1)	SCC*mec* IVa+ACME II*	1
	CC45-MRSA-V	*Sporadic*°°	SCC*mec* VT (PM1)	1
			SCC*mec* VT (var.3)*	1
	CC45-MRSA-V+ACME	*Sporadic*°°	SCC*mec* VT+ACME II*	1
**CC59**	CC59-MRSA-VT (PVL+), “Taiwan Clone”	0.2% (n = 2)	SCC*mec* VT (PM1)	3
**ST72**	ST72-MRSA-IV A	0.1% (n = 1)	SCC*mec* IV A (CN1)	1
**CC80**	atypical CC80-MSSA-SCC (ORF CM14, PVL+)	*Sporadic*°°°°	ACME III+*ccrA/B-*1 (Strain 21342)	1
	CC80-MRSA-IV (PVL+)	0.9% (n = 11)	SCC*mec* IVc (TCH60)	8
**CC88**	CC88-MRSA-IV	0.2% (n = 2)	SCC*mec* IVa (MW2)	1
			SCC*mec* IVa (CMFT503)	1
			SCC*mec* IVc (TCH60)	1
	CC88-MRSA-IV (PVL+)	0.3% (n = 4)	SCC*mec* IVa (MW2)	2
			SCC*mec* IVa (H131520133)	1
**ST93**	CC93-MRSA-IV (PVL+) “Queensland Clone”	Sporadic°°	SCC*mec* IVa (MW2)	2
**CC97**	CC97-MRSA-IV	0.2% (n = 2)	SCC*mec* IVa (MW2)	2
	CC97-MRSA-[V+*fus*]	*Sporadic*°°	SCC*mec* V+fus*	1
**CC130**	CC130-MRSA-XI	0.2% (n = 2)	SCC*mec* XI (LGA251/M10-61)	2
**CC152**	CC152-MRSA-V (PVL+)	0.1% (n = 1)	SCC*mec* VT (GR1)	1
**CC182**	CC182-MSSA-SCC	*Sporadic*°	*kdp*+*ccrA/B-*2*	1
**ST239**	ST239-MRSA-III	0.7% (n = 8)	SCC*mec* III+Cd/Hg+*ccrC* (SK1585)	3
			SCC*mec* III+Cd/Hg+*ccrC* (Bmb9393)	1
			SCC*mec* III+*ccrC*+Cd (CN79)	1
			SCC*mec* III+*ccrC*+Cd*	1
**CC398**	CC398-MRSA-IV	0.1% (n = 1)	SCC*mec* IVc (WW2703/97)	1
	CC398-MRSA-V, “Livestock-assoc. MRSA”	1.3% (n = 16)	SCC*mec* VT+*czrC* (SO385)	13
			SCC*mec* VT+*czrC* (as in SO385, but *ydhK*-negat.)*	1
		0.1% (n = 1)	SCC*mec* VT+Cu/*czrC*+*ccrA/B-*1*	1
		0.1% (n = 1)	PseudoSCC*mec*, class C *mec* complex+As/Cu*	1
**ST617**	ST617-MRSA-IV	*Sporadic*°°	SCC*mec* IVa (MW2)	1
**ST772**	ST772-MRSA-V, “Bengal Bay Clone”	0.4% (n = 5)	SCC*mec* V (Bengal Bay)	5

° Since routine typing of MSSA is not performed, no reliable data on the prevalence of this strain can be provided. However, based on data from other regional studies that included MSSA [32,33,34,35], it appears locally not to be common.

°° This strain was only identified in sporadic cases from Dresden Neustadt Hospital (where no systematic typing was performed). The absence from Dresden University Hospital indicates that it either generally very rare in Saxony, and/or that infections might be associated with travel and thus randomly detected.

°°° This strain was accidentally detected in one healthy carrier, not in a patient. Thus it is not included into the routine typing figures.

°°°° This strain was found once in an imported case tested for diagnostic purposes.

* Unknown variant, no matching sequence identified among published genome or SCC sequences. For details see Table 3.

A subset of 226 isolates from both sites was selected for SCC*mec* subtyping aiming on a high diversity of isolates (see S1 Table). For that purpose, epidemiologically linked or consecutive isolates from a single patient were excluded, while isolates from different years and different wards were prioritised as well as isolates that differed in carriage of additional resistance or toxin genes.

### Bacteriological procedures

MRSA isolates passed through standard clinical routine diagnostics. After primary culture and subculturing of single colonies, clumping factor was detected utilising the Pastorex StaphPlus kit (Bio-Rad Laboratories GmbH, Munich, Germany). Antibiotic susceptibility tests were performed by VITEK 1 or VITEK 2 systems (BioMerieux, Nuertingen, Germany). Methicillin resistance was confirmed by detection of PBP2a using the Innogenetics MRSA-screen agglutination assay (Innogenetics, Ghent, Belgium). Isolates were stored frozen using cryobank tubes (Microbank, Pro-Lab Diagnostics, Richmond Hill, Canada) at -80°C. Only one isolate per patient was considered.

### Linear DNA amplification, labelling and array procedures

An initial characterisation of the isolates was performed using StaphyType DNA microarrays (Alere Technologies GmbH, Jena, Germany). This array covers 333 different targets that correspond to approximately 170 distinct genes and their allelic variants. These genes include species and typing markers, toxin genes and resistance genes. Detailed descriptions have been published previously [11,12,13]. This array also covers several SCC-associated markers such as *mecA*, *mecC*, *fusC*, recombinase genes etc. that are listed in Table 2 as well as in S2 Table.

**Table 2:** SCC-associated markers used for this study, sorted alphabetically, references and estimated abundances in MRSA from Dresden (2000–2016; based on prevalence data from Table 1 and [10]).

Gene/Marker	Gene product/Function	Comments	Reference sequence	Reference for probes and primers	Estimated prevalence
***adhC***_**(FPR3757)**_	Alcohol dehydrogenase, zinc-containing	Part of ACME 1 and ACME 3 clusters, that occurs alone or in combination with SCC*mec* elements. Consensus marker. Allele from the “USA300”CA-MRSA strain (FPR3757, GenBank CP000255.1 and TCH1516, GenBank CP000730.1)	CP000255.1, 64125…65222	This paper	<0.5%
***arcA-*SCC**	Arginine deiminase	Part of ACME 1 and ACME 2 clusters, that occurs alone or in combination with SCC*mec* elements. Common in Coagulase-negatives, present in the “USA300”CA-MRSA strain (FPR3757, GenBank CP000255.1 and TCH1516, GenBank CP000730.1)	CP000255.1, 73113…74348	[11,12,13]	<0.5%
***arcB-*SCC**	Ornithine carbamoyltransferase	Part of ACME 1 and ACME 2 clusters, that occurs alone or in combination with SCC*mec* elements. Common in Coagulase-negatives, present in the “USA300”CA-MRSA strain (FPR3757, GenBank CP000255.1 and TCH1516, GenBank CP000730.1)	CP000255.1, 69839…70837	[11,12,13]	<0.5%
***arcC-*SCC**	Carbamate kinase	Part of ACME 1 and ACME 2 clusters, that occurs alone or in combination with SCC*mec* elements. Common in Coagulase-negatives, present in the “USA300”CA-MRSA strain (FPR3757, GenBank CP000255.1 and TCH1516, GenBank CP000730.1)	CP000255.1, 68890…69819	[11,12,13]	<0.5%
***arcD-*SCC**	Arginine/ornithine antiporter	Part of ACME 1 and ACME 2 clusters, that occurs alone or in combination with SCC*mec* elements. Common in Coagulase-negatives, present in the “USA300”CA-MRSA strain (FPR3757, GenBank CP000255.1 and TCH1516, GenBank CP000730.1)	CP000255.1, 71606…73027	[11,12,13]	<0.5%
***arsB-*SCC**	Arsenical pump membrane protein	Three different probes were designed for SCC-born allele(s) of that gene, as for instance in JCSC6943, JCSC6945, M10/0061	FR823292.1, 28161…29450; AB505628.1, 31886…33175; AB705453.1, 2380…3669	This paper	<0.5%
***arsC-*SCC**	Arsenate reductase	Probe was designed for SCC-born allele(s) of that gene, as for instance in JCSC6943, JCSC6945	AB505628.1, 31467…31868	This paper	<0.1%
**B2Y834**	Abortive phage resistance protein	Subtyping SCC*mec* IV, *i*.*e*., identification of SCC*mec* IV A, G, c and SCC*mec* MRSAZH47	AE015929.1, 50641…51441	This paper	3,5%
**B6VQU0**	Putative protein	Subtyping SCC*mec* IV, *i*.*e*., identification of SCC*mec* IVh/j	AB425824.1, 19950…20882	This paper	47%
***blaZ***_**(SCCmec XI)**_	Beta-lactamase	Solely present in SCC*mec* XI; this is a different *blaZ* allele than in ubiquitous staphylococcal plasmids	FR821779.1, 34736…35587	[11,12,13]	<0.5%
**C5QAP8**_**(SCCmec XI)**_	Putative protein	Identification of SCC*mec* XI	FR821779.1, 53134…53913	This paper	<0.5%
***cadD***_**(R35)**_	Cadmium transport protein D	Probe was designed for SCC-born allele(s) of that gene, as for instance in strain R35, GenBank L10909.1 or strain 85/2082, GenBank AB037671.1	L10909.1, 5577…6194	This paper	<1%
***cadX***_**(JCSC6943)**_	Putative regulator of cadmium efflux	Probe was designed for SCC-born allele(s) of that gene, as for instance in JCSC6943, GenBank AB505628.1	AB505628.1, 31106…31447	This paper	0%
***cap 1***	Locus encoding SCC associated capsule type 1	Individual probes for probes for *cap* H1, J1, K1	U10927.2	This paper	0%
***cas1***_**(M06-0171)**_	CRISPR-associated endonuclease 1	Present in M06/0171, GenBank HE980450.1	HE980450.1, 48518…49423	This paper	0%
***ccrA-1***	Cassette chromosome recombinase A, type 1	Cassette chromosome recombinase A allele found in SCC*mec* I, IX, X, SCC*fus* (as in MSSA476, GenBank BX571857.1) and in composite SCC*mec*/*fus* elements	CP000046.1, 47998…49347	[11,12,13]	3%
***ccrA-2***	Cassette chromosome recombinase A, type 2	Cassette chromosome recombinase A allele found in SCC*mec* II and IV elements	BA000033.2, 48017…49366	[11,12,13]	90%
***ccrA-3***	Cassette chromosome recombinase A, type 3	Cassette chromosome recombinase A allele found in SCC*mec* III elements	AB037671.1, 5430…6776	[11,12,13]	<1%
***ccrA-4***	Cassette chromosome recombinase A, type 4	Cassette chromosome recombinase A allele found in SCC*mec* VI and SCC*mec* VIII elements	AF411935.3, 7849…9210	[11,12,13]	<1%
***ccrAA***	“Cassette chromosome recombinase AA”	Gene for a hypothetical protein accompanying the *ccrC* gene in SCC*mec* V and SCC*mec* VT elements. Two separate probes were used that usually, but not always, yield identical results	AB121219.1, 14264…15907; AM292304.1, 5654…7273	[11,12,13]	3%
***ccrB-1***	Cassette chromosome recombinase B, type 1	Cassette chromosome recombinase B allele found in SCC*mec* I, IX, SCC*fus* (as in MSSA476, GenBank BX571857.1) and in composite SCC*mec*/*fus* elements	GU122149.1, 11999…13624	[11,12,13]	3%
***ccrB-2***	Cassette chromosome recombinase B, type 2	Cassette chromosome recombinase B allele found in SCC*mec* II and IV elements	BA000033.2, 46367…47995	[11,12,13]	90%
***ccrB-3***	Cassette chromosome recombinase B, type 3	Cassette chromosome recombinase B allele found in SCC*mec* III elements	AB037671.1, 6797…8425	[11,12,13]	<1%
***ccrB-4***	Cassette chromosome recombinase B, type 4	Cassette chromosome recombinase B allele found in SCC*mec* VI and SCC*mec* VIII elements	AE015929.1, 58592…60220	[11,12,13]	<1%
***ccrC***_**(85–2082)**_	Cassette chromosome recombinase C	Cassette chromosome recombinase C allele found in in SCC*mec* V, SCC*mec* VT and SCC*mec* VII elements	AB037671.1, 6797…8425	[11,12,13]	4%
***copA2-*SCC**	copper exporting ATPase	Probe was designed for SCC-born allele(s) of that gene, as for instance in FPR3757, GenBank CP000255.1 or JCSC6943, GenBank AB505628.1	CP000255.1, 86055…88118	This paper	<1%
***cstB-*SCC1 (Q2G1R6)**	CsoR-like sulfur transferase-regulated genes B/metallo-beta-lactamase superfamily protein. Pseudogene containing two stop codons	Subtyping SCC*mec* II (usually present, but absent in Irish SCC*mec* II variants A to E) and III (usually present, but absent in CMFT492 GenBank HF569112.1). Also present in SCC*mec* VIII and irregular elements such as *Staphylococcus fleurettii* GenBank AB546266	BA000017.4, 50957…52291	This paper	21%
***cstB*-SCC2 (Q2G1R6)**	CsoR-like sulfur transferase-regulated genes B/metallo-beta-lactamase superfamily protein.	Present in SCC*mec* IVa (truncated) and SCC*mec* X, variably present in SCC*mec* I (usually present, but absent from MR1 GenBank ACZQ, Geraldine Clone) and VT. The corresponding probe was used in this study to distinguish SCC*mec* IVa from other SCC*mec* IV subtypes.	CP000046.1, 53428…54756	This paper	25%
***czrC***	Cadmium and zinc resistance gene C, heavy metal translocating P-type ATPase	Frequently associated with SCC*mec* elements from livestock MRSA	AE015929.1, 64066…66000	This paper	2%
**D1GU38**	Putative protein	Subtyping SCC*mec* III, identification of SCC*mec* VT, SCC*mec* ZH47, SCC*mec* VII because of an association with (additional/second) *ccrC* copies	FN433596.1, 34888…35751	This paper	3%
**D1GU55**	Putative membrane protein	Subtyping SCC*mec* III, additional marker for SCC*mec* VII	FN433596.1, 52909…53208	This paper	<0.5%
**D3JD07**	Putative protein	Present in some composite SCC elements such as, *e*.*g*., M06/0171, GenBank HE980450.1 and 45394F, GenBank GU122149.1	GU122149.1, 8985…9797	This paper	0%
**Delta *mec*R1**	Truncated methicillin resistance operon repressor 1	Truncated *mec*R1 is present in SCC*mec* I, IV, V, VI, VII; complete absence of *mec*R1 from SCC*mec* V, IX, X	BA000033.2, 41708…42682	[11,12,13]	76%
**DUF1958**	Putative protein	Subtyping SCC*mec* VT. Present, *e*.*g*., in PM1, GenBank BAFA but absent, *e*.*g*., in Strain 3957, GenBank AOFU	GQ902038.2, 38193…38519	This paper	<0.5%
***fusC* (Q6GD50)**	SCC-associated fusidic acid resistance gene	Present alone in “SCC*fus*” elements or together with *mecA* in composite elements. Most common in CC1 and CC5 strains	BX571857.1, 52820…53458	[11,12,13]	<0.5%
***kdpA-*SCC**	Potassium-translocating ATPase A, chain 2	Present in SCC*mec* II (although absent from Irish SCC*mec* II variants A to E)	BA000018.3, 77116…78792	[11,12,13]	20%
***kdpB-*SCC**	Potassium-transporting ATPase B, chain 1	Present in SCC*mec* II (although absent from Irish SCC*mec* II variants A to E)	BA000018.3, 78811…80832	[11,12,13]	20%
***kdpC-*SCC**	Potassium-translocating ATPase C, chain 2	Present in SCC*mec* II (although absent from Irish SCC*mec* II variants A to E)	BA000017.4, 80809…81366	[11,12,13]	20%
***kdpD-*SCC**	Sensor kinase protein	Present in SCC*mec* II (although absent from Irish SCC*mec* II variants A to E)	BA000018.3, 74179…76899	[11,12,13]	20%
***kdpE-*SCC**	KDP operon transcriptional regulatory protein	Present in SCC*mec* II (although absent from Irish SCC*mec* II variants A to E)	BA000018.3, 73509…74204	[11,12,13]	20%
***mco-*SCC**	Multi copper oxidase	Probe was designed for SCC-born allele(s) of that gene, as for instance in JCSC6943, GenBank AB505628.1	AB505628.1, 35823…37256	This paper	<0.5%
***mecA***	Modified penicillin binding protein (PBP2a)	Modified penicillin binding protein (PBP2a) causing oxacillin/methicillin resistance and thus defining MRSA	BA000017.4, 44992…46998	[11,12,13]	>99.5%
***mecC***	Alternate gene encoding a modified penicillin binding protein	Present in, and characteristic for, SCC*mec* XI	FR821779.1, 35681…37678	[11,12,13]	<0.5%
***mecI***	Methicillin-resistance regulatory protein	Present in SCC*mec* II (although absent from Irish SCC*mec* II variants C and E), SCC*mec* III, SCC*mec* VIII	BA000017.4, 48855…49226	[11,12,13]	21%
***mec*R1**	Methicillin resistance operon repressor 1	Un-truncated sequence in SCC*mec* II, SCC*mec* III, SCC*mec* VIII	BA000017.4, 47098…48855	[11,12,13]	21%
***merA***	Mercury reductase	Part of a mercury resistance operon that is plasmid born, although the plasmid can be integrated into SCC*mec* elements (for instance, in strains 85/2082, GenBank AB037671.1 or TW20, GenBank FN433596.1)	AB037671.1, 38289…39932	[11,12,13]	<1%
***merB***	Alkylmercury lyase	Part of a mercury resistance operon that is plasmid born, although the plasmid can be integrated into SCC*mec* elements (for instance, in strain TW20, GenBank FN433596.1)	AB037671.1, 37557…38207	[11,12,13]	<1%
***mvaS-*SCC**	Truncated 3-hydroxy-3-methylglutaryl CoA synthase	Subtyping SCC*mec* I, II, IV, V	BA000033.2, 37179…37531	This paper	78%
***opp3B* (*opp3B***_**(C427)**_ and ***opp3B***_**(FPR3757)**_**)**	Oligopeptide permease, channel-forming protein	Part of ACME 1 and ACME 3 clusters that occurs alone or in combination with SCC*mec* elements. A consensus probe as well as specific probes for alleles known from coagulase-negatives and from the USA 300 CA-MRSA strain (FPR3757, GenBank CP000255.1 and TCH1516, GenBank CP000730.1) were used	ACSQ01000050.1, 4183…5139; CP000255.1, 81950…82906	This paper	<0.5%
***opp3C***_**(C427)**_	Oligopeptide permease, channel-forming protein	Part of ACME 1 and ACME 3 clusters that occurs alone or in combination with SCC*mec* elements. Allele from known coagulase-negatives	ACSQ01000050.1, 5139…5906	This paper	0%
***opp3C***_**(FPR3757)**_	Oligopeptide permease, channel-forming protein	Part of ACME 1 and ACME 3 clusters that occurs alone or in combination with SCC*mec* elements. Allele from the USA 300 CA-MRSA strain (FPR3757, GenBank CP000255.1 and TCH1516, GenBank CP000730.1)	CP000255.1, 82906…83673	This paper	<0.5%
***pls-*SCC**_**(COL)**_	Plasmin-sensitive surface protein, prevents bacterial adhesion in vitro, located in SCC, close to *mec* operon	Subtyping SCC*mec* I (Note: an additional, but unrecognised allele is also present in the irregular SCC element of WA MRSA-40: JQ746621.1)	CP000046.1, 57212…61858	This paper	5%
**PSM*-mec***	Phenol soluble modulin from SCC*mec*	Present in SCC*mec* II (although absent from Irish SCC*mec* II variants C and E), SCC*mec* III, SCC*mec* VIII	BA000017.4, 49311…49379	This paper	21%
**Q3YK51**	Putative protein	Subtyping SCC*mec* IV, *i*.*e*., identification of SCC*mec* IV g	DQ106887.1, 196…1944	This paper	0%
**Q4LAG7**	Putative protein located within SCC*mec* type V/SCC*fus* elements	Identification of SCC*mec* V/VT elements and of SCC*fus* elements	AM990992.1, 50512…50940 (V/VT); BX571857.1, 55452…55880 (fus)	This paper	3%
**Q8CU82**	Putative protein	Present in some SCC*mec*/*fus* composite elements such as, *e*.*g*., CMFT120, GenBank HF569094.1 and CMFT2, GenBank HF569101.1	AE015929.1, 32604…32786	This paper	<0.1%
**Q933A2**	Putative ADP-ribosyltransferase	Subtyping SCC*mec* III and SCC*mec* IX	FN433596.1, 101805…102377	This paper	<1%
**Q93IB7**	LytTR domain DNA-binding regulator	Subtyping SCC*mec* III (present in, *e*.*g*., TW20 GenBank FN433596.1, but absent in, *e*.*g*., Bmb9393 GenBank CP005288.1) and IV (usually absent, but present in, *e*.*g*., CMFT503 GenBank HF569113.1)	FN433596.1, 67873…68115	This paper	1%
**Q9S0M4**	Putative protein	Subtyping SCC*mec* I, SCC*mec*/ACME composites and SCC*mec* from WA40	JQ746621.1, 10406…11456	This paper	5%
***Q9XB68-dcs***	Located at the terminus of SCC*mec* directly next to *orfX*.	This locus comprises the downstream constant segment (*dcs*) that in turn comprises a copy of the SCC direct repeat DR_SCC (AGAAGCTTATCATAAGTAA)	*dcs*: CP000046.1, 34192…34371; Q9XB68: CP000046.1, 34372…35667	[11,12,13]	95%
**SCC terminus 01**	SCC integration site alternate to *dcs*		GU235983.1, 488…808	This paper	1%
**SCC terminus 02**	SCC integration site alternate to *dcs*		FN433596.1, 34140…34456	This paper	3%
**SCC terminus 03**	SCC integration site alternate to *dcs*		FR753166.1, 481…568	This paper	<0.5%
**SCC terminus 04**	SCC integration site alternate to *dcs*		ACSW01000146.1, 46259…46316	This paper	<0.5%
**SCC terminus 05**	SCC integration site alternate to *dcs*		AB425427.1, 606…1027	This paper	<0.5%
**SCC terminus 06**	SCC integration site alternate to *dcs*	Associated mainly to SCC*fus* (as in MSSA476, GenBank BX571857.1)	BX571857.1, 34169…34545	This paper	<0.1%
**SCC terminus 07**	SCC integration site alternate to *dcs*		GU122149.1, 119…222	This paper	<1%
**SCC terminus 09**	SCC integration site alternate to *dcs*		AB121219.1, 898…1198	This paper	0%
**SCC terminus 10**	SCC integration site alternate to *dcs*		AB505630.1, 581…881	This paper	<0.5%
**SCC terminus 11**	SCC integration site alternate to *dcs*		HF569096.1, 746…1034	This paper	<1%
**SCC terminus 12**	SCC integration site alternate to *dcs*		CP003808.1, 34287…34414	This paper	<0.1%
**SCC terminus 13**	SCC integration site alternate to *dcs*		ARXY01000001.1, 131181…131299	This paper	0%
**SCC terminus 14**	SCC integration site alternate to *dcs*		HF569093.1, 481…644	This paper	0%
***speG***_**(FPR3757)**_	Spermidine N-acetyltransferase	Usually associated with ACME or composite SCC*mec*/ACME elements	CP000255.1, 63100…63597	This paper	<1%
***tirS***	Staphylococcal TIR-protein binding protein	Subtyping SCC*fus* because it is frequently, but not always, accompanying *fusC*	BX571857.1, 50640…51482	This paper	<0.5%
***ugpQ***	Glycerophosphoryl diester phosphodiesterase	Accompanies *mecA* in all SCC*mec* sequences except SCC*mec* IV A from CN1 GenBank CP003979.1 and an irregular/composite element from *Staphylococcus epidermidis*, BCM-HMP0060 GenBank ACHE	BA000018.3, 43717…44460	[11,12,13]	>99.5%
***xylR/mecR2***	Methicillin resistance operon repressor 2, Homolog of xylose repressor	Located next to *mec* operon downstream of *mecI* (not present if *mecI* is truncated). Present in SCC*mec* II (although absent from Irish SCC*mec* II variants C and E), SCC*mec* III, SCC*mec* VIII	BA000018.3, 49738…50882	[11,12,13]	21%
***ydhK***_**(FPR3757)**_	Putative lipoprotein	Present in some composite elements comprising SCC*mec* and heavy metal resistance genes including the one in FPR3757, GenBank: CP000255.1	CP000255.1, 88136…88681	This paper	2%
***yeeA***	Putative DNA methyltransferase	Subtyping SCC*mec*IV/*fus* composite elements	HF569093.1, 2580…5294	This paper	0%

A further characterisation of SCC*mec* elements of selected isolates (see above) was performed using probes and primers for new targets also listed in Table 2. Criteria for the selection of these target genes are discussed below, in the Results section. Probes and primers for SCC-related targets are described in S2 Table.

The procedures for all array experiments were identical and they have been described previously [11,12]. *S*. *aureus* was cultured and cloned on Columbia blood agar plates, harvested and enzymatically lysed. DNA was purified using Qiagen spin columns. A linear amplification was performed using one specific primer per target. Biotin-16-dUTP was randomly incorporated into the amplicons during that step. After incubation with the array and after washing steps, hybridization to probes immobilised to the array was detected using streptavidin-horseradish-peroxidase that catalyses a local precipitation of a dye. Microarrays were then photographed and analysed using a designated reader and software (Iconoclust, Alere Technologies GmbH, Jena, Germany). This allowed establishing the presence or absence of certain genes or alleles as well as, by automated comparison of resulting patterns to a database, an assignment to clonal complexes, strains and SCC*mec* types.

### Virtual hybridisations

For comparison of real-life experiments with published genome sequences, a computer-based method for predicting DNA array hybridization patterns from full genome sequences was used. Predicted patterns were generated either from fully finished genomic sequences (all gaps closed) or from partially assembled sequences as typically obtained from next generation sequencing (NGS). A large number of partially assembled sequences of staphylococci is available in the WGS section of NCBI GenBank (http://www.ncbi.nlm.nih.gov/Traces/wgs/). The computational method identified the binding sites of the hybridisation probes in the genomic sequences. For simplicity, only the probe binding sites were determined, while the binding sites of the labelling primers were not considered. If more than one binding site was found, only the one with the highest number of matches between probe and target was taken into account. The number of mismatches between probe and target sequence was used to predict the strength of the normalized hybridization signal. Perfect matches (*i*.*e*., no mismatches) were set to the maximum signal, while four or more mismatches were set to no signal at all. One mismatch yielded a slightly attenuated maximum signal, two mismatches yielded half of the maximal signal, and three mismatches yielded a weak signal which is set slightly above the noise level. The computation method thus resulted in datasets comparable to those from real experiments. This approach was validated by comparing experimental to predicted data of fully sequenced, well known reference strains (such as MSSA476, GenBank BX571857; N315, GenBank BA000018; COL, GenBank CP000046; MRSA252, GenBank BX571856; or strains with well-characterised SCC*mec* elements [14]; see S1 Table). Several predicted hybridisation patterns matched experimental results of clinical isolates characterised herein (see Table 3 and S1 Table). However, some isolates also were observed that yielded patterns for which no matching sequence could be identified (see Table 3 and S1 Table).

**Table 3:** SCC elements as identified by array hybridisation in this study, reference sequences, their gene contents, distributions across clonal complexes and their estimated abundances in MRSA from Dresden (2000–2016; based on prevalence data from Table 1 and [10]).

SCC*mec* subtypes	GenBank accession and/or reference	*mec* complex	Other payload	Recombinase genes	SCC termini	Identified in CC	Estimated prevalence
SCC*mec* I (COL)	CP000046.1	*mecA*, *ugpQ*, Delta *mecR1*	*mvaS*, *cstB-*SCC2, Q9S0M4, *pls*SCC	*ccrA/B-1*	*dcs*	CC5	3%
SCC*mec* I (PSP1996)	ANHU	*mecA*, *ugpQ*, Delta *mecR1*	*cstB-*SCC2, Q9S0M4, *plsSCC*	*ccrA/B-1*	*dcs*	CC8	<0.5%
SCC*mec* I+*fus+tirS* (Geraldine Clone)	MRSA7 in [15]	*mecA*, *ugpQ*, Delta *mecR1*	*mvaS*, *fusC*, *tirS*	*ccrA/B-1*	*dcs*	CC5	<0.1%
SCC*mec* II (JH1/JH9)	CP000736,CP000703.1	*mecA*, *ugpQ*, *mecR*, *mecI*, *psmMEC*, *xylR*	*cstB-*SCC1, *kdp*	*ccrA/B-2*	*dcs*	CC5	20%
SCC*mec* II (N315/Mu50)	BA000018.3,BA000017.4	*mecA*, *ugpQ*, *mecR*, *mecI*, *psmMEC*, *xylR*	*mvaS*, *cstB-*SCC1, *kdp*	*ccrA/B-2*	*dcs*	CC30	<0.1%
SCC*mec* II+*czrC*+ccrA/B-4*	unknown	*mecA*, *ugpQ*, *mecR*, *mecI*, *psmMEC*, *xylR*	*cstB-*SCC1, *kdp*, *czrC*	*ccrA/B-2*, *ccrA/B-4*	*dcs*	CC5	<0.5%
SCC*mec* II+*speG*+*czrC*+ccrA/B-4*	unknown	*mecA*, *ugpQ*, *mecR*, *mecI*, *psmMEC*, *xylR*	*cstB-*SCC1, *kdp*, *speG*, *czrC*	*ccrA/B-2*, *ccrA/B-4*	*dcs*	CC5	<0.5%
SCC*mec* IIA/B/D without *ccrA/B-4* genes (Irish AR13/14)	AHVO	*mecA*, *ugpQ*, *mecR*, *mecI*, *psmMEC*, *xylR*	*cstB-*SCC1, *mvaS-*SCC, *speG*	*ccrA/B-2*	*dcs*	CC8	<0.1%
SCC*mec* III+*ccrC*+Cd (CN79)	ANCJ	*mecA*, *ugpQ*, *mecR*, *mecI*, *psmMEC*, *xylR*	*mvaS*, *cstB-*SCC1, D1GU38, Q933A2, D1GU55, *cadD*	*ccrA/B-3*, *ccrC*	*dcs*, SCCterm2	ST239	<0.1%
SCCmec III+ccrC+Cd*	unknown	*mecA*, *ugpQ*, *mecR*, *mecI*, *psmMEC*, *xylR*	*mvaS*, *cstB-*SCC1, D1GU38, Q933A2, D1GU55, *cadD*, Q4LAG7, Q9S0M4	*ccrA/B-3*, *ccrC*	*dcs*, SCCterm2, 5	ST239	<0.1%
SCC*mec* III+Cd/Hg+*ccrC* (Bmb9393)	CP005288.1	*mecA*, *ugpQ*, *mecR*, *mecI*, *psmMEC*, *xylR*	*mvaS*, *cstB-*SCC1, D1GU38, Q933A2, *merA/B*, *cadD*	*ccrA/B-3*, *ccrC*	SCCterm2	ST239	<0.1%
SCC*mec* III+Cd/Hg+*ccrC* (SK1585)	AYLT	*mecA*, *ugpQ*, *mecR*, *mecI*, *psmMEC*, *xylR*	*mvaS*, *cstB-*SCC1, Q93IB7, D1GU38, Q933A2, *merA/B*, *cadD*	*ccrA/B-3*, *ccrC*	SCCterm1, 2	ST239	<0.5%
SCC*mec* IVa (MW2)	BA000033.2	*mecA*, *ugpQ*, Delta *mecR1*	*mvaS*, *cstB-*SCC2	*ccrA/B-2*	*dcs*	CC1, CC5, CC6, CC7, CC8, CC22, CC30, CC45, CC88, ST93, CC97, ST617	16.5%
SCC*mec* IVa (H131520133)	BioSample SAMEA2385424	*mecA*, *ugpQ*, Delta *mecR1*	*cstB-*SCC2	*ccrA/B-2*	*dcs*	CC30	<0.5%
SCC*mec* IVa*	unknown	*mecA*, *ugpQ*, Delta *mecR1*	*mvaS*, *cstB-*SCC2	*ccrA/B-2*	SCCterm4	CC8	<0.1%
SCC*mec* IVa (CMFT503)	HF569113.1	*mecA*, *ugpQ*, Delta *mecR1*	*mvaS*, *cstB-*SCC2, Q93IB7	*ccrA/B-2*	SCCterm1	CC22, CC88	<1%
SCC*mec* IVa+ACME I+Cu (USA300)	CP000255.1CP000730.1	*mecA*, *ugpQ*, Delta *mecR1*	*mvaS*, *cstB-*SCC2, *ydhK*, ACME I°, *adhC*, *speG*, *copA2-*SCC	*ccrA/B-2*	*dcs*	CC8	<0.5%
SCC*mec* IVa+ACME II*	Unknown	*mecA*, *ugpQ*, Delta *mecR1*	*mvaS*, *cstB-*SCC2, ACME II°	*ccrA/B-2*	*dcs*, SCCterm3	CC45	<0.1%
SCC*mec* IVa+ccrC*	unknown	*mecA*, *ugpQ*, Delta *mecR1*	*mvaS*, *cstB-*SCC2, D1GU38	*ccrA/B-2*, *ccrC*	*dcs*, SCCterm1	CC8	<0.5%
SCC*mec* IVb/d/i (JCSC1978/6668/4469)	AB063173.1 (IVb), AB097677 .1(IV d), AB425823.1 (IV i)	*mecA*, *ugpQ*, Delta *mecR1*	*mvaS*	*ccrA/B-2*	*dcs*	CC5, CC7, CC45	<1%
SCC*mec* IVb/d/i (Strain 21209)	AGRP	*mecA*, *ugpQ*, Delta *mecR1*	-	*ccrA/B-2*	*dcs*	CC8	<0.5%
SCC*mec* IVb/d/i+Cu*	unknown	*mecA*, *ugpQ*, Delta *mecR1*	*mvaS*, *ydhK*, *copA2-*SCC	*ccrA/B-2*	*dcs*	CC30	<0.1%
SCC*mec* IVc (IS-105)	AHLR	*mecA*, *ugpQ*, Delta *mecR1*	B2Y834, (variably, *merA/B*)**	*ccrA/B-2*	*dcs*	CC5, CC8, CC22	<1%
SCC*mec* IVc (TCH60)	CP002110.1	*mecA*, *ugpQ*, Delta *mecR1*	*mvaS*, B2Y834	*ccrA/B-2*	*dcs*	CC5, CC8, CC22, CC45, CC80, CC88	2%
SCC*mec* IVc (WW2703/97)	ACSW	*mecA*, *ugpQ*, Delta *mecR1*	*mvaS*, B2Y834	*ccrA/B-2*	SCCterm4	CC398	<0.1%
SCC*mec* IVc+*speG*+*ccrA/B-4* (SA_ST125)	ASTH	*mecA*, *ugpQ*, Delta *mecR1*	B2Y834, *speG*	*ccrA/B-2*, *ccrA/B-4*	*dcs*, SCCterm7	CC5	<0.1%
SCC*mec* IVc+Cu/Hg (MRSA177)	AECP	*mecA*, *ugpQ*, Delta *mecR1*	*mvaS*, B2Y834, *ydhK*, *Q4LAG7*, *merA/B*, *copA2*-SCC, *mco*	*ccrA/B-2*	*dcs*	CC8	<0.5%
SCC*mec* IVh/j (HO50960412/JCSC6670)	HE681097.1 (IVh), AB425824.1 (IV j)	*mecA*, *ugpQ*, Delta *mecR1*	*mvaS*, B6VQU0	*ccrA/B-2*	*dcs*	CC8, CC22	47%
SCC*mec* IVh/j+ACME2 (M08-0126)	FR753166.1	*mecA*, *ugpQ*, Delta *mecR1*	*mvaS*, B6VQU0, Q9S0M4, ACME II°	*ccrA/B-2*	*dcs*, SCCterm3, 5	CC22	<0.1%
SCC*mec* IV+*speG*+Cu/*czrC*+ccrA/B-4*	unknown	*mecA*, *ugpQ*, Delta *mecR1*	*mvaS*, *cstB-*SCC2, *ydhK*, *speG*, *copA2-*SCC, *czrC*	*ccrA/B-2*, *ccrA/B-4*	*dcs*, SCCterm7	CC22	<0.1%
SCC*mec* IV+*speG*+*czrC*+*ccrA/B-4* (var.1)*	unknown	*mecA*, *ugpQ*, Delta *mecR1*	*mvaS*, *speG*, *czrC*	*ccrA/B-2*, *ccrA/B-4*	*dcs*, SCCterm7	CC22	<0.5%
SCC*mec* IV+*speG*+*czrC*+*ccrA/B-4* (var.2)*	unknown	*mecA*, *ugpQ*, Delta *mecR1*	*speG*, *czrC*	*ccrA/B-2*, *ccrA/B-4*	*dcs*, SCCterm7	CC22	<0.1%
SCC*mec* IV A (CN1)	CP003979.1	*mecA*, Delta *mecR1*	B2Y834	*ccrA/B-2*	*dcs*	ST72	<0.1%
SCC*mec* V (Bengal Bay)	HF569096.1,AZBT	*mecA*, *ugpQ*,	*mvaS*, Q4LAG7	*ccrAA*, *ccrC*	SCCterm11	ST772, CC1	<0.5%
SCC*mec* V*	unknown	*mecA*, *ugpQ*,	*mvaS*, Q4LAG7	*ccrAA*, *ccrC*	SCCterm10	CC5	<0.1%
SCC*mec* V+fus*	unknown	*mecA*, *ugpQ*	*mvaS*, Q4LAG7, *fusC*	*ccrAA*, *ccrC*	SCCterm3, 10	CC97	<0.1%
SCC*mec* V+*fus+tir*+*ccrA/B-*1*	unknown	*mecA*, *ugpQ*	*mvaS*, Q4LAG7 *fusC*, *tirS*	*ccrA/B-1*, *ccrAA*, *ccrC*	SCCterm3, 6, 11	CC1	<0.1%
SCCmec V+*speG*+ czrC+ccrA/B-4*	unknown	*mecA*, *ugpQ*	*mvaS*, Q4LAG7, Q8CU82, *speG*, *czrC*	*ccrC*, *ccrA/B-4*	SCCterm7	CC22	<0.1%
SCC*mec* VT (GR1)	AJLX	*mecA*, *ugpQ*	*mvaS*, D1GU38, Q4LAG7	*ccrAA*, *ccrC*	SCCterm2	CC152	<0.1%
SCC*mec* VT (PM1)	BAFA	*mecA*, *ugpQ*	*mvaS*, D1GU38, DUF1958, Q4LAG7	*ccrAA*, *ccrC*	SCCterm2	CC45, CC59	<0.5%
SCC*mec* VT (var.1)*	unknown	*mecA*, *ugpQ*	*mvaS*, D1GU38	*ccrC*	SCCterm2	CC7	<0.1%
SCC*mec* VT (var.2)*	unknown	*mecA*, *ugpQ*	D1GU38, DUF1958, Q4LAG7	*ccrAA*, *ccrC*	SCCterm2	CC30	<0.1%
SCC*mec* VT (var.3)*	unknown	*mecA*, *ugpQ*	*mvaS*, D1GU38, DUF1958, Q4LAG7, Q9S0M4	*ccrAA*, *ccrC*	SCCterm2, 5	CC45	<0.1%
SCC*mec* VT+ACME II*	unknown	*mecA*, *ugpQ*,	*mvaS*, D1GU38, Q4LAG7	*ccrAA*, *ccrC*	SCCterm2	CC45	<0.1%
SCC*mec* VT+Cu/*czrC*+*ccrA/B-*1*	unknown	*mecA*, *ugpQ*	*mvaS*, *cstB-*SCC2, D1GU38, B2Y834, *ydhK*, *Q4LAG7*, *copA2-*SCC, *czrC*	*ccrA/B-1*, *ccrAA*, *ccrC*	SCCterm2, 7	CC398	<0.1%
SCC*mec* VT+*czrC* (SO385)	AM990992.1	*mecA*, *ugpQ*	*mvaS*, *cstB-*SCC2, *ydhK*, D1GU38, Q4LAG7, *czrC*	*ccrAA*, *ccrC*	SCCterm2	CC8, CC398	1%
SCC*mec* VT+*czrC* (as in SO385, but *ydhK* negative)*	unknown	*mecA*, *ugpQ*	*mvaS*, *cstB-*SCC2, D1GU38, Q4LAG7, *czrC*	*ccrAA*, *ccrC*	SCCterm2	CC398	<0.5%
SCC*mec* VT+*fus*+czrC*	unknown	*mecA*, *ugpQ*	*mvaS*, *cstB-*SCC2, D1GU38, Q4LAG7, *fusC*, *czrC*	*ccrAA*, *ccrC*	SCCterm2, 12	CC22	<0.1%
SCC*mec* VI+*fus* (MRSA18)	MRSA18 in [15]	*mecA*, *ugpQ*, Delta *mecR1*	*mvaS*, Q4LAG7, *fusC*	*ccrB-4*	SCCterm7	CC7	<0.1%
SCC*mec* XI (LGA251/M10-61)	FR823292.1,FR821779.1	*mecC*	*blaZ-*SCC*mec XI*, *C5QAP8-M10-61*, *arsB-*SCC	*ccr*(*A/B*)°°	- °°°	CC130	<0.5%
PseudoSCC*mec*, class B *mec* complex+Hg*	unknown	*mecA*, *ugpQ*, Delta *mecR1*	*mvaS*, (*merA/B*)**	*-*	*dcs*	CC8	<1%
PseudoSCC*mec*, class B *mec* complex+Cu/Hg*	unknown	*mecA*, *ugpQ*, Delta *mecR1*	*mvaS*, *ydhK*, Q4LAG7, *merA/B*, *copA2-*SCC, *mco*	*-*	*dcs*	CC8	<0.1%
PseudoSCC*mec*, class B *mec* complex*	unknown	*mecA*, *ugpQ*, Delta *mecR1*	*mvaS*, *cstB-*SCC2, Q9S0M4, plsSCC	*-*	*dcs*	CC5	2%
PseudoSCC*mec*, class C *mec* complex+As/Cu*	unknown	*mecA*, *ugpQ*	*mvaS*, *ydhK*, *arsB-*SCC, *arsC*, *copA2-*SCC	*-*	SCCterm10	CC398	<0.1%
ACME II*	unknown	-	ACME II°, Q9S0M4	*-*	SCCterm3, 5	CC8	MSSA only
ACME II+*speG*+*czrC*+*ccrA/B-*4*	unknown	-	ACME II°, *speG*, *czrC*, Q9S0M4	*ccr*(A***)*/B-4*	SCCterm3, 5, 7	CC8	MSSA only
*fus+tir*+*ccrA/B-*1 (MSSA476)	BX571857.1	-	Q4LAG7, *fusC*, *tirS*	*ccrA/B-1*	SCCterm3, 6	CC1	MSSA only
*kdp*+*ccrA/B-*2*	unknown	-	*kdp*	*ccrA/B-2*	-	CC182	MSSA only
*arsB*+*ccrA/B-*4*	unknown	-	*mvaS*-SCC, B6VQU0, *arsB-*SCC	*ccrA/B-4*	*dcs*	CC22	MSSA only
ACME III+*ccrA*1 (Strain 21342)	AHKU	-	ACME III°	*ccrA/B-1*	SCCterm7	CC80	MSSA only
*speG*+*czrC*+ccrA/B-4*	unknown	-	*speG*, *czrC*,	*ccr*(A***)*/B-4*	SCCterm7	CC8, CC22	MSSA only

* Unknown variant, no matching sequence identified among published genome or SCC sequences.

** In absence of genome sequence data, it cannot be decided whether this operon was localised on SCCmec or on a plasmid.

*** Weak reactivity might indicate a divergent allele.

° ACME I = *arc* and *opp* genes, ACME II = *arc* genes only, ACME III = *opp* genes only.

°° Recombinase genes in SCC*mec* XI are described as *ccrA1/B3* (http://www.sccmec.org/Pages/SCC_TypesEN.html) but yielded weak signals with probes for *ccrB1/A3*

°°° There is one unique SCC terminal sequence associated to SCC*mec* XI but no primers and probes were designed for it.

## Results

### SCC*mec* typing markers

Probes including those that were used for clonal complex determination as well as detection of toxin genes and resistance genes have been previously discussed, and these probe and primer sequences were provided elsewhere [11,12].

SCC related markers together with definitions, short explanations and reference sequences are listed in Table 2. S2 Table contains, beyond this information, also the individual primers and probes utilised for this study. These markers have been selected from published genome sequences because of an unambiguous, strict linkage to SCC elements and their variable presence in those elements. Some have been annotated differently, introducing identical names or gene symbols to genes or features which are highly similar in sequence. For several genes, allelic variants were distinguished.

Table 2 also shows estimated prevalences for the individual markers. This is based on strain prevalences as shown in Table 1 and [10]. It should be noted that these are projections rather than actual figures because i) not all isolates recovered were fully characterised (especially, it was not possible to include each isolate of abundant strains such as CC22-MRSA-IV “Barnim EMRSA”), ii) no systematic testing was performed in one of the two hospitals (see above) and iii) there were clear changes to the population structure of MRSA over time [10], and this also affects marker prevalences.

Various resistance markers are typically located on transposons or insertion elements and are known to be occasionally associated with SCC*mec* elements (such as *aadD*, *erm*(A) or *tet* genes) were covered by the array. However, they were not used for SCC*mec* subtyping because DNA array hybridisation cannot provide information whether they are associated with SCC elements or carried on other mobile elements such as on plasmids or transposons.

The mercury resistance operon was used for subtyping some strains, but since it can be carried outside of SCC elements, these results should be regarded as preliminary. For other heavy metal resistances, probes were designed that distinguish alleles that are known to be associated with SCC from those that were described from other mobile elements; and only the former ones were analysed for this paper.

### SCC termini

SCC termini were investigated because of their relevance for the design of PCRs that span the integration sites of SCC elements into the *S*. *aureus* genome proving that a positive SCC*mec*/*mecA* amplification was attributable to the presence of MRSA in a sample rather than to *mecA*-positive staphylococci of other species. SCC elements insert into the chromosomes of staphylococcal species by site-specific recombination. Insertion is catalysed by the cassette chromosome recombinase which is encoded on the SCC element by genes *ccrA*, *ccrB* or *ccrC*. The recognition site is a stretch of 16 nucleotides located at the 3'-end of the coding sequences of *orfX* (a putative 23S rRNA methyltransferase). The sequence of the insertion site is doubled upon insertion giving rise to pairs of direct repeats. Composite SCC elements often have more than two direct repeats. In genomes carrying a SCC element, the terminal region of the SCC element located downstream of *orfX* has been called downstream constant segment (*dcs*). The name reflects that this region was found to be highly conserved in all the longer known sequences of SCC elements. Nowadays, a much larger and more diverse set of SCC sequences is available. The SCC terminal region is the intergenic region between *orfX* and the first codons annotated in the SCC element. In an analysis of complete SCC sequences from GenBank we have identified *dsc* but also 14 other distinct types of the intergenic region between *orfX* and the first codon annotated in the SCC element.

For *dcs* and another 13 terminal integration site sequences, primers and probes were developed and used to screen isolates. A 14th sequence was identified in published genome sequences of *mecC*/SCC*mec* XI-positive strains, but it was not screened for, being redundant to the other SCC*mec* XI-associated markers already covered by the array.

Ten terminal integration site sequences were indeed found among the isolates herein.

Multiple SCC termini, in 15 different combinations, were identified in strains and isolates that harbour composite or multiple SCC elements (see Table 3). This is in accordance to published genome sequences of strains harbouring composite or multiple SCC elements, where additional SCC termini can be found also in a distance from *orfX*. An example is GenBank FR753166.1, where SCC terminus 3 (positions 481 to 586), *dcs* (24011 to 24292) and SCC terminus 5 (13044 to 13465) are found.

The association of SCC termini with types and subtypes SCC elements is shown in Table 3.

Given the prevalences of strains as shown in Table 1, it can be estimated that *dcs* is present in about 95% of isolates from the study region. However, especially among the sporadic and/or travel-associated strains, other SCC termini were observed.

### Characterisation of SCC elements and subtypes

Sixty-one distinct SCC elements and subtypes were identified using the set of primers and probes described herein. An overview is given in Table 3, full data are provided in the S1 Table.

### Clonal complexes and strains that were found to harbour SCC elements

Full details on CC/strain assignments, SCC elements and subtypes are provided in Table 1.

#### CC1-MSSA

One CC1-MSSA isolate was identified that carried a SCC*fus* element apparently identical to the one in MSSA476, GenBank BX571857.1.

#### CC1-MRSA

PVL-negative CC1-MRSA-IV were rare and all of them harboured SCC*mec* IVa elements apparently identical to the one in the sequenced strain MW2 (BA000033.2). SCC*mec* IV/SCC*fus* composite elements as described elsewhere [15,16,17] were not identified. One PVL-positive CC1-MRSA was identified that carried a SCC*mec* V element as also observed in the Bengal Bay clone (ST772, see below) and another one harboured a SCC*mec* V+SCC *fus* composite element.

#### CC5-MRSA

As previously described, CC5/ST228-MRSA-I, the so-called “South German” epidemic MRSA (EMRSA) strain used to be common in Dresden around the year 2000, but it nearly disappeared since [10]. With regard to SCC*mec* elements, two variants were observed. One appeared identical to SCC*mec* I from the CC8 strain COL (GenBank CP000046.1) as well as to those of CC5-MRSA-I strains from Switzerland (GenBank HE579059.1 to HE579069.1). These genome sequences also suggest that the variable presence of the *mer* operon in CC5-MRSA-I was related to plasmid carriage rather than to variability of the SCC*mec* element. A second variant harboured a pseudoSCC*mec* element, lacking recombinase genes. Nearly all isolates harbouring this variant were cultured within one year suggesting an epidemiological linkage.

A single isolate of CC5-MRSA-I/*fus*, “Geraldine Clone” [18], was found in 2012 in a patient with a history of foreign travel. It carried a combined SCC*mec*I/SCC*fus* element that also included *tirS* matching the predicted pattern for the sequence of strain MRSA-7 [15].

CC5-MRSA-II is a common strain known as “New York/Japan clone” or, in Germany, as “Rhine-Hesse EMRSA”. Analysis of published sequences indicate that there are two different variants of SCC*mec* II that differ in presence (Mu50: BA000017.4, N315: BA000018.3) or absence (JH1: CP000736.1, JH9: CP000703.1) of *mvaS*. All tested isolates from Dresden lacked this gene. Recently (2014/2015), sporadic isolates were found that additionally carried *ccrA/B-4*, *czrC* and, variably, *speG*.

CC5-MRSA-IV, “Paediatric clone”, was only sporadically found. SCC*mec* IVa, IVb/d/i and IVc were identified among PVL-negative isolates of this “strain”. All PVL-positives yielded SCC*mec* IVc. One CC5-MRSA-[IV+*ccrA/B-4*] (as in a strain from Spain, SA_ST125, GenBank ASTH) was detected in a patient with travel history to the Canary Islands.

SCC*mec* V was only found once in a CC5 MRSA.

#### CC6-MRSA

Three isolates were found; and two of them originated from patients with Middle Eastern travel history. All harboured SCC*mec* IVa elements.

#### CC7-MRSA

Just five isolates were identified. Two carried SCC*mec* IVb/d/I; SCC*mec* IVa, VT and VI+*fus* elements were found once each.

#### CC8-MSSA

Three different strains of MSSA were identified that harboured SCC elements without *mecA/mecC*. They yielded signals for different combinations of ACME II, *speG*, *czrC* and *ccA/B-4* genes (Table 1).

#### CC8-MRSA

Only a single isolate of ST247-MRSA-I, “North German/Iberian EMRSA”, was found. Its hybridisation pattern, with regard to SCC genes, was in concordance to the predicted pattern for strain PSP1996 (GenBank ANHU) but differed from SCC*mec* elements of COL (GenBank CP000046.1) and the local CC5/ST228-MRSA-I strain in the absence of *mvaS*-SCC.

One isolate of ST8-MRSA-IIA/B/D, “Irish AR13/14” [19], was detected in a healthy carrier (a student in a microbiology course).

For CC8-MRSA-IV, several different strains of PVL-negative have been described previously that could be distinguished mainly based on enterotoxin gene carriage. All were only sporadically found. “UK-EMRSA-14”, without enterotoxin genes, was found twice, harbouring SCC*mec* IVc or h/j. The “Lyon Clone”, i.e., *sea*-positive CC8-MRSA-IV yielded SCC*mec* IVc. “USA500” (*seb*-positive CC8-MRSA-IV that occasionally also carry *sea*, *sek*, *seq*) were found to carry SCC*mec* IVa (from a patient with Ethiopian background) or an *mvaS*-negative variant of SCC*mec* IVb/d/i (from two cases with infections acquired in Mozambique and Zimbabwe).

USA300-like, PVL-positive CC8-MRSA could be assigned by SCC*mec* subtyping to four distinct strains out of which the most common one was ACME-positive. This strain harboured SCC*mec* IVa, an ACME I element and a copper resistance gene as present in genome sequences FPR3757, GenBank CP000255.1, and TCH1516 GenBank CP000730.1. A second strain harboured a SCC*mec* IVc element, two copper resistance genes (*copA2*-SCC, *mco-*SCC), and the mercury resistance operon but lacked ACME. It is likely to be identical to the SCC*mec* element from MRSA177, GenBank AECP. A third strain lacked *ccrA/B-*2 and B2Y834. A forth strain, identified once, harboured SCC*mec* IVa (as also present in the genome sequence of a USA300-like strain, IS-88, GenBank AHLO).

At least three distinct strains could be distinguished that were originally merged under the label “Hannover EMRSA”. Two of these strains were common in Dresden around the turn of the century [10]. One harboured a pseudoSCC*mec* element and the mercury resistance operon. The other one harboured a composite element including SCC*mec* IV, *ccrC* and D1GU38 (a marker that accompanies “additional” *ccrC*, see Table 1) as well as Q2G1R6 suggesting that this composite element derived from a SCC*mec* IVa t. A third variant of the “Hannover Epidemic Strain” was represented by “Hannover 100–93” from the Harmony Strain Collection and UK-EMRSA-10 (courtesy of G. Coombs). However, they harboured B6VQU0 indicating relationship to SCC*mec* IVh/j rather than to IVa.

Finally, a single CC8-MRSA-VT strain was found that carried *czrC*, thus resembling the SCC elements of CC398 livestock-associated MRSA.

#### CC22-MSSA

CC22-MSSA with two different SCC elements were identified. One element, harbouring *arsB* and *ccrA/B-4*, was identified just once. The other one comprised *speG*, *czrC* and *ccrA/B-4* and it was found in several recent (2014/2015) isolates some of which showed growth on MRSA selective media.

#### CC22-MRSA

The vast majority of CC22 were assigned to the CC22-MRSA-IV strain known as “UK-EMRSA-15” or locally also as “Barnim EMRSA” [20]. This strain appeared first in 2001 [10] become rapidly more common and accounted nearly 80% of all genotyped MRSA isolates in 2013. These isolates harboured SCC*mec* IVh/j elements.

Few CC22-MRSA-IV isolates were identified that yielded positive signals for *fnbB* (indicating that they could belong to different lineage within CC22 in which *fnbA* and *fnbB* are not fused, [21]). Some (4 out of 7) *fnbB*-positive isolates also carried SCC*mec* IVh/j elements, one had a SCC*mec* IVa element, and two were assigned to SCC*mec* IVc.

Another CC22-MRSA-IV that differs from UK-15/Barnim in being positive for *tst1* has been described from the Middle East and Mediterranean regions as “Gaza Strain” [22,23]. A single isolate was identified in 2015 from a patient with a Middle Eastern name, and it harboured SCC*mec* IVa.

PVL-positive CC22-MRSA-IV were rarely found. Older isolates (one each from the years 2000, 2001 and 2002) carried SCC*mec* IVh/j as observed for UK-15/Barnim. More recent isolates (2014–15) yielded SCC*mec* IVa or IVc.

A single CC22 isolate was found that carried a SCC*mec* IVh/j+ACME2 composite element. An apparently identical element has previously been described in a CC22 strain from Ireland [24].

A few isolates were also identified that harboured composite elements in various constellations (see Tables 1 and 3).

#### CC30-MRSA

CC30/ST36-MRSA-II, UK-EMRSA-16 was identified only once, in 2002 [10]. It carried a N315-like SCC*mec* II element.

PVL-positive CC30-MRSA-IV are known as “Southwest Pacific Clone”, “WSPP” (West Samoa Phage Pattern) clone or "USA1100”. Published genome sequences indicate the presence of different SCC*mec* subtypes in different isolates of that “strain” (SCC*mec* IVb/d/i in Strain WBG10049, GenBank ACSV; SCC*mec* IVc in Strain 122051, GenBank AHZJ and Strain TCH60, GenBank CP002110.1). The majority of the few Dresden isolates proved to carry SCC*mec* IVa elements with (7 out of 9 isolates) or without (1 out of 9) *mvaS*-SCC; a putative composite of SCC*mec* IVb/d/i+*copA*2-SCC was found once.

A CC30 strain with a SCC*mec* VT element was identified once in a child (with the mother carrying most likely the same strain).

#### CC45-MRSA

CC45-MRSA-IV (“Berlin EMRSA”) was frequently identified in the early 2000s. However, it nearly disappeared since and is only sporadically found usually in elderly patients. It was already noted that most, although not all, “Berlin EMRSA” isolates carried *apha3+sat* (kana-/neomycin and streptothricin resistance [10]). All *aphA3+sat*-positive isolates harboured SCC*mec* IVa. Among *aphA3+sat*-negatives, SCC*mec* IVa was found as well as SCC*mec* IVb/d/I and IVc.

Published genome sequences of CC45-MRSA-IV also show different SCC*mec* IV subtypes (IVa in CIG1524 GenBank AHVI; IVb/d/I in 300–169, GenBank JASL, and 301–188, GenBank JASK).

CC45-MRSA-V were rare, only two isolates (with SCC*mec* VT elements) were identified. CC45-MRSA-IV and -V with additional ACME I elements were found once each.

#### CC59-MRSA

Only three MRSA isolates were identified and assigned to CC59-MRSA-VT (PVL+), “Taiwan Clone”. Their SCC*mec* VT elements appeared to be identical to that of the prototypical strain PM1, GenBank BAFA.

#### ST72-MRSA

Although MLST indicates similarity/relationship to CC8, ST72 differs in several core genomic features such as the presence of the enterotoxin gene cluster [11]. A single isolate of that lineage was identified and it carried the same SCC*mec* IV A element as present in the genome sequence of the ST72 strain CN1, GenBank CP003979.1,. This element is rather unique due to the absence of *ugpQ*.

#### CC80-MSSA

One CC80-MSSA isolate was isolated from an abscess of an Eritrean refugee. It was PVL-positive, but contrarily to other CC80 it carried the enterotoxin homologue ORF CM14, and it also harboured an ACME III element.

#### CC80-MRSA

PVL-positive CC80-MRSA-IV were sporadically identified from 2004 on, and frequently cases were associated with travel to Mediterranean regions or the Middle East [25]. All tested isolates invariably carried TCH60-like SCC*mec* IVc elements.

#### CC88-MRSA

CC88-MRSA were only found very sporadically. PVL-negative CC88-MRSA-IV isolates harboured SCC*mec* IVa (although two different variants) or IVc elements. PVL-positive isolates also carried SCC*mec* IVa (as also found in HST-105, GenBank AZTH) but showed a variable presence of *mvaS-*SCC.

#### ST93-MRSA

Two PVL-positive isolates of ST93-MRSA-IV, “Queensland clone” [26,27], were found to carry SCC*mec* IVa elements.

#### CC97-MRSA

CC97-MRSA were found twice. Both isolates harboured MW2-like SCC*mec* IVa elements, as the CC97 genome sequence IS-55, GenBank AHLN does.

#### CC130-MRSA

CC130-MRSA-XI have been identified three times (2009, 2014, 2015). Two isolates were available for further characterisation, being positive for *mecC* and essentially identical to the strain M10-0061 from the strain’s original description [2].

#### CC152-MRSA

A single isolate of CC152-MRSA-V (PVL+) was found. It carried a SCC*mec* VT element that appeared identical to the one in GR1, GenBank AJLX.

#### CC182-MSSA

Some (but not all) CC182-MSSA carried a SCC element comprising the *kdp* locus and *ccrA/B-2* genes.

#### ST239-MRSA

ST239 is a chimeric lineage in which a CC30-like stretch of genomic DNA is inserted into a CC8 parent genome. There are several genome sequences available, and their analysis shows a remarkable variability of their SCC*mec* elements, especially with regard to the presence of heavy metal resistance operons and an additional recombinase gene, *ccrC*.

This strain was only sporadically found in Dresden, always related to importation. Isolates from an outbreak in 2001, with an index patient repatriated from Greece [10], showed a composite SCC*mec* III element including *ccrC* as well as cadmium and mercury resistance operons that was identical to the predicted pattern of strain SK1585 (GenBank AYLT). Another isolate was found in 2015 in a patient with history of Middle Eastern travel. It also harboured a slightly different composite SCC*mec* III element (see Table 3) that resembled Bmb9393, GenBank CP005288.1. One 2008 isolate from a patient with history of hospitalisation in Turkey [10] yielded a composite SCC*mec* III element including *ccrC* as well as the Cd resistance operon. It appeared to be identical to the SCC*mec* element in CN79, GenBank ANCJ, and 16K, GenBank BABZ. Another 2016 isolate, also from a patient with travel history, showed a similar element that matched no known sequence (see Table 3).

#### CC398-MRSA

“Livestock-associated” CC398-MRSA have sporadically been detected from 2005 on, with a slight increase in recent years. The majority of isolates carried the SCC*mec* VT/*czrC* composite element as the sequenced strain SO385. However, sporadic isolates with an *ydhK*-negative variant thereof, a SCC*mec* IVc, a composite SCC*mec* VT/heavy metal resistance element and a pseudoSCC*mec* element have also been observed (see Table 3).

#### ST617-MRSA

ST617 was described as a putative recombinant of CC8 and CC45 parents sporadically observed in Germany [28]. A single isolate was found to carry a SCC*mec* IVa element.

#### ST772-MRSA

ST772 is lineage that is by MLST related to CC1. As previously described [29], it differs in several core genomic features. One emerging community-associated MRSA strain, the “Bengal Bay Clone”, belongs to this lineage. Five isolates have been found; all carried a unique variant of SCC*mec* V that was also present in several published genome sequences of the “Bengal Bay Clone”.

## Discussion

Although the characterised strain collection that was rather small, confined to a sampling period of a few years and a restricted geographic area, a remarkable variety of SCC*mec* elements was observed. For all major SCC*mec* types, distinct subtypes could be identified, and some additional rare or even not yet sequenced SCC*mec* elements were observed. When expanding the panel of genes used for SCC*mec* typing/subtyping, a number of additional variants of SCC*mec* types or subtypes can be discerned. Several common strains showed a remarkable variability of SCC*mec* types or subtypes. These include ST239-MRSA-III, CC5-MRSA-IV, CC22-MRSA-IV or CC398-MRSA-V/VT.

Theoretically, there are two explanations for this observation.

A common and widespread strain might further evolve, after geographic dissemination, by acquiring additional markers of selective advantage (such as additional antibiotic resistance genes or heavy metal resistance genes), or by losing genes that do not actually confer an advantage (such as possibly additional recombinase genes from composite elements). This could be an explanation for the different variants of ST239-MRSA-III that can harbour a number of different SCC*mec* III/heavy metal resistance composite elements. Another example could be the observation of SCC*mec* II and IVa subtypes that just differ in the presence or absence of *mvaS*-SCC. The most parsimonious explanation would just be a random deletion of that gene from SCC*mec* elements in some specimens.

Another possibility is that a “strain” was in fact polyphyletic. This means that related parental MSSA from one lineage independently might have acquired different (although sometimes similar or related) subtypes of SCC*mec* elements. This could be the case for CC5-MRSA-IV, CC22-MRSA-IV and PVL-positive CC30-MRSA-IV. These observations also imply that the emergence of novel MRSA strains by acquisition of SCC*mec* elements might be rather common.

Another issue is strain definition and nomenclature. While naming strains is highly practical for routine use one should be aware that a nomenclature is an artificial convention and depends on resolution power of typing methods. Traditionally, a strain or clone has been defined as a “group of isolates that can be distinguished from other isolates of the same genus and species by phenotypic characteristics or genotypic characteristics or both” [30,31]. This might be feasible when using MLST/*spa* or PFGE typing but is not practical when applying microarrays or genome sequencing. This might appear to be a somewhat esoteric issue but it has practical consequences, especially when ruling out or confirming identity in outbreak investigations. For practical purposes, clinicians and infection control officers need clear breakpoints indicating how many “differences” (in terms of numbers of single nucleotide polymorphisms or of mobile genes being present or absent) safely rule out a possible transmission or how many might still be considered as consistent with “identity” and thus with a possible transmission.

Regarding SCC*mec* nomenclature, the main types easily can be categorised using the framework previously provided (http://www.sccmec.org/Pages/SCC_TypesEN.html). However, microarrays and genome sequencing reveal a high degree of variability on a “subtype” level as well as a rather common presence of irregular and/or composite elements. In order to avoid cumbersome, subjective and eventually ambiguous designations, sequencing and referencing on sequence data (*i*.*e*., accession numbers or strain designations unambiguously linked to accession numbers) appears to be inevitable.

Since SCC*mec* elements often contain repetitive and mobile sequences (such as *IS*431) they are especially prone to be fragmented and split across several contigs when performing NGS. As array hybridisation, NGS thus cannot always and instantly provide information on gene localisations, and it also cannot reliably recognise duplications or inversions. A set of probes and targets as used herein could not only be used *in vitro* for typing and for selecting isolates that warrant sequencing, but also for a computerised analysis of NGS sequences allowing a quick assignment to strains and variants. With increasing availability of NGS technology, rapid data analysis and data transfer to non-expert users will become a major challenge. The development of specific sets of markers that can be interrogated *in vitro* as well as in NGS datasets might help to solve that problem and might also help to find a practical solution to the problem of defining identity or non-identity as discussed above.

The proposed microarray can help to distinguish isolates that appear similar or identical by other typing methods and it can be used as high-throughput screening tool for the detection of novel SCC variants that warrant detailed investigation and sequence analysis. The high degree of heterogeneity of SCC elements even within so-called strains can be utilised for epidemiological typing.

## Supporting Information

S1 Table Hybridisation profiles for tested reference strains (highlighted in red) and clinical isolates as well as predicted hybridisation patterns for reference sequences (highlighted in blue). (PDF)

S2 Table Target genes, primers and probes. (PDF)

## References

[1] Monecke S, Muller E, Schwarz S, Hotzel H, Ehricht R (2012) Rapid microarray based identification of different *mecA* alleles in Staphylococci. Antimicrob Agents Chemother 56: 5547–5554. 10.1128/AAC.00574-12 22890767 PMC3486549

[2] Shore AC, Deasy EC, Slickers P, Brennan G, O'Connell B, Monecke S, et al (2011) Detection of staphylococcal cassette chromosome *mec* type XI carrying highly divergent *mecA*, *mecI*, *mecR1*, *blaZ*, and *ccr* genes in human clinical isolates of clonal complex 130 methicillin-resistant *Staphylococcus aureus*. Antimicrob Agents Chemother 55: 3765–3773. 10.1128/AAC.00187-11 21636525 PMC3147645

[3] Garcia-Alvarez L, Holden MT, Lindsay H, Webb CR, Brown DF, Curran MD, et al (2011) Meticillin-resistant *Staphylococcus aureus* with a novel *mecA* homologue in human and bovine populations in the UK and Denmark: a descriptive study. Lancet Infect Dis 11: 595–603. 10.1016/S1473-3099(11)70126-8 21641281 PMC3829197

[4] Ito T, Katayama Y, Asada K, Mori N, Tsutsumimoto K, Tiensasitorn C, et al (2001) Structural comparison of three types of staphylococcal cassette chromosome *mec* integrated in the chromosome in methicillin-resistant *Staphylococcus aureus*. Antimicrob Agents Chemother 45: 1323–1336. 11302791 10.1128/AAC.45.5.1323-1336.2001 PMC90469

[5] Ito T, Ma XX, Takeuchi F, Okuma K, Yuzawa H, Hiramatsu K. (2004) Novel type V staphylococcal cassette chromosome *mec* driven by a novel cassette chromosome recombinase, *ccrC*. Antimicrob Agents Chemother 48: 2637–2651. 15215121 10.1128/AAC.48.7.2637-2651.2004 PMC434217

[6] IWG-SCC (2009) Classification of staphylococcal cassette chromosome *mec* (SCC*mec*): guidelines for reporting novel SCC*mec* elements. Antimicrob Agents Chemother 53: 4961–4967. 10.1128/AAC.00579-09 19721075 PMC2786320

[7] Shore AC, Coleman DC (2013) Staphylococcal cassette chromosome *mec*: Recent advances and new insights. Int J Med Microbiol 303: 350–359. 10.1016/j.ijmm.2013.02.002 23499303

[8] Wu Z, Li F, Liu D, Xue H, Zhao X (2015) Novel Type XII Staphylococcal Cassette Chromosome *mec* Harboring a New Cassette Chromosome Recombinase, CcrC2. Antimicrobial Agents and Chemotherapy 59: 7597–7601. 10.1128/AAC.01692-15 26416872 PMC4649182

[9] Holden MTG, Feil EJ, Lindsay JA, Peacock SJ, Day NPJ, Enright MC, et al (2004) Complete genomes of two clinical *Staphylococcus aureus* strains: Evidence for the rapid evolution of virulence and drug resistance. PNAS 101: 9786–9791. 15213324 10.1073/pnas.0402521101 PMC470752

[10] Albrecht N, Jatzwauk L, Slickers P, Ehricht R, Monecke S (2011) Clonal replacement of epidemic methicillin-resistant *Staphylococcus aureus* strains in a German university hospital over a period of eleven years. PLoS ONE 6: e28189 10.1371/journal.pone.0028189 22140542 PMC3227659

[11] Monecke S, Coombs G, Shore AC, Coleman DC, Akpaka P, Borg M, et al (2011) A Field Guide to Pandemic, Epidemic and Sporadic Clones of Methicillin-Resistant *Staphylococcus aureus*. PLoS One 6: e17936 10.1371/journal.pone.0017936 21494333 PMC3071808

[12] Monecke S, Slickers P, Ehricht R (2008) Assignment of *Staphylococcus aureus* isolates to clonal complexes based on microarray analysis and pattern recognition. FEMS Immunol Med Microbiol 53: 237–251. 10.1111/j.1574-695X.2008.00426.x 18507678

[13] Monecke S, Gavier-Widen D, Mattsson R, Rangstrup-Christensen L, Lazaris A, Coleman DC et al (2013) Detection of *mecC-*positive *Staphylococcus aureus* (CC130-MRSA-XI) in diseased European hedgehogs (Erinaceus europaeus) in Sweden. PLoS ONE 8: e66166 10.1371/journal.pone.0066166 23776626 PMC3680430

[14] Kinnevey PM, Shore AC, Brennan GI, Sullivan DJ, Ehricht R, Monecke S, et al (2014) Extensive Genetic Diversity Identified among Sporadic Methicillin-Resistant *Staphylococcus aureus* Isolates Recovered in Irish Hospitals between 2000 and 2012. Antimicrobial Agents and Chemotherapy 58: 1907–1917. 10.1128/AAC.02653-13 24395241 PMC4023797

[15] Ellington MJ, Reuter S, Harris SR, Holden MT, Cartwright EJ, Greaves D, et al (2015) Emergent and evolving antimicrobial resistance cassettes in community-associated fusidic acid and meticillin-resistant *Staphylococcus aureus*. Int J Antimicrob Agents 45: 477–484. 10.1016/j.ijantimicag.2015.01.009 25769787 PMC4415905

[16] Williamson DA, Monecke S, Heffernan H, Ritchie SR, Roberts SA, Upton A, et al (2014) High Usage of Topical Fusidic Acid and Rapid Clonal Expansion of Fusidic Acid–Resistant *Staphylococcus aureus*: A Cautionary Tale. Clinical Infectious Diseases 59: 1451–1454. 10.1093/cid/ciu658 25139961

[17] Baines SL, Howden BP, Heffernan H, Stinear TP, Carter GP, Seemann T, et al (2016) Rapid emergence and evolution of *Staphylococcus aureus* clones harbouring fusC-containing Staphylococcal Cassette Chromosome elements. Antimicrobial Agents and Chemotherapy. 60: 2359–65 10.1128/AAC.03020-15 26856837 PMC4808225

[18] Dauwalder O, Lina G, Durand G, Bes M, Meugnier H, Jarlier V, et al (2008) Epidemiology of invasive methicillin-resistant *Staphylococcus aureus* clones collected in France in 2006 and 2007. J Clin Microbiol 46: 3454–3458. 10.1128/JCM.01050-08 18667599 PMC2566079

[19] Shore A, Rossney AS, Keane CT, Enright MC, Coleman DC (2005) Seven novel variants of the staphylococcal chromosomal cassette *mec* in methicillin-resistant *Staphylococcus aureus* isolates from Ireland. Antimicrob Agents Chemother 49: 2070–2083. 15855533 10.1128/AAC.49.5.2070-2083.2005 PMC1087651

[20] Witte W, Enright M, Schmitz FJ, Cuny C, Braulke C, Heuck D (2001) Characteristics of a new epidemic MRSA in Germany ancestral to United Kingdom EMRSA 15. Int J Med Microbiol 290: 677–682. 11310446 10.1016/S1438-4221(01)80006-0

[21] Holden MTG, Hsu L-Y, Kurt K, Weinert LA, Mather AE, Harris SR, et al (2013) A genomic portrait of the emergence, evolution, and global spread of a methicillin-resistant *Staphylococcus aureus* pandemic. Genome Research 23: 653–664. 10.1101/gr.147710.112 23299977 PMC3613582

[22] Aqel AA, Alzoubi HM, Vickers A, Pichon B, Kearns AM (2015) Molecular epidemiology of nasal isolates of methicillin-resistant *Staphylococcus aureus* from Jordan. Journal of Infection and Public Health 8: 90–97. 10.1016/j.jiph.2014.05.007 25002017

[23] Laham NA, Mediavilla JR, Chen L, Abdelateef N, Elamreen FA, Ginocchio CC, et al (2015) MRSA Clonal Complex 22 Strains Harboring Toxic Shock Syndrome Toxin (TSST-1) Are Endemic in the Primary Hospital in Gaza, Palestine. PLoS ONE 10: e0120008 10.1371/journal.pone.0120008 25781188 PMC4364023

[24] Shore AC, Rossney AS, Brennan OM, Kinnevey PM, Humphreys H, Sullivan DJ, et al (2011) Characterization of a novel arginine catabolic mobile element (ACME) and staphylococcal chromosomal cassette *mec* composite island with significant homology to *Staphylococcus epidermidis* ACME type II in methicillin-resistant *Staphylococcus aureus* genotype ST22-MRSA-IV. Antimicrob Agents Chemother 55: 1896–1905. 10.1128/AAC.01756-10 21343442 PMC3088263

[25] Monecke S, Slickers P, Hotzel H, Richter-Huhn G, Pohle M, Weber S, et al (2006) Microarray-based characterisation of a Panton-Valentine leukocidin-positive community-acquired strain of methicillin-resistant *Staphylococcus aureus*. Clin Microbiol Infect 12: 718–728. 16842566 10.1111/j.1469-0691.2006.01420.x

[26] Coombs GW, Goering RV, Chua KY, Monecke S, Howden BP, Stinear TP, et al (2012) The Molecular Epidemiology of the Highly Virulent ST93 Australian Community *Staphylococcus aureus* Strain. PLoS ONE 7: e43037 10.1371/journal.pone.0043037 22900085 PMC3416834

[27] Munckhof WJ, Schooneveldt J, Coombs GW, Hoare J, Nimmo GR (2003) Emergence of community-acquired methicillin-resistant *Staphylococcus aureus* (MRSA) infection in Queensland, Australia. Int J Infect Dis 7: 259–264. 14656416 10.1016/s1201-9712(03)90104-4

[28] Strommenger B, Braulke C, Heuck D, Schmidt C, Pasemann B, Nübel U, et al (2008) *spa* Typing of *Staphylococcus aureus* as a Frontline Tool in Epidemiological Typing. Journal of Clinical Microbiology 46: 574–581. 18032612 10.1128/JCM.01599-07 PMC2238071

[29] Monecke S, Baier V, Coombs GW, Slickers P, Ziegler A, Ehricht R. (2013) Genome sequencing and molecular characterisation of *Staphylococcus aureus* ST772-MRSA-V, “Bengal Bay Clone”.BMC Research Notes 6: 1–7. 24359724 10.1186/1756-0500-6-548 PMC3878137

[30] Tenover FC, Arbeit RD, Goering RV, Mickelsen PA, Murray BE, Persing DH, et al (1995) Interpreting chromosomal DNA restriction patterns produced by pulsed-field gel electrophoresis: criteria for bacterial strain typing. J Clin Microbiol 33: 2233–2239. 7494007 10.1128/jcm.33.9.2233-2239.1995 PMC228385

[31] Dijkshoorn L, Ursing BM, Ursing JB (2000) Strain, clone and species: comments on three basic concepts of bacteriology. J Med Microbiol 49: 397–401. 10798550 10.1099/0022-1317-49-5-397

[32] Luedicke C, Slickers P, Ehricht R, Monecke S (2010) Molecular fingerprinting of *Staphylococcus aureus* from bone and joint infections. Eur J Clin Microbiol Infect Dis 29: 457–463. 10.1007/s10096-010-0884-4 20186451

[33] Monecke S, Luedicke C, Slickers P, Ehricht R (2009) Molecular epidemiology of *Staphylococcus aureus* in asymptomatic carriers. Eur J Clin Microbiol Infect Dis 28: 1159–1165. 10.1007/s10096-009-0752-2 19434432

[34] Monecke S, Müller E, Buechler J, Rejman J, Stieber B, Akpaka PE, et al (2013) Rapid detection of Panton-Valentine leukocidin in *Staphylococcus aureus* cultures by use of a lateral flow assay based on monoclonal antibodies. J Clin Microbiol 51: 487–495. 10.1128/JCM.02285-12 23175260 PMC3553917

[35] Monecke S, Slickers P, Ellington M, Kearns A, Ehricht R (2007) High diversity of Panton-Valentine leucocidin-positive, methicillin-susceptible isolates of Staphylococcus aureus and implications for the evolution of community-associated MRSA. Clin Microbiol Infect 13: 1157–1164. 17949441 10.1111/j.1469-0691.2007.01833.x

