# Supplementary material for: Diversity of SCCmec Elements in Staphylococcus aureus as Observed in South-Eastern Germany
Source: PLoS One. 2016 Sep 20;11(9):e0162654. doi: 10.1371/journal.pone.0162654 (PMC5029946; doi:10.1371/journal.pone.0162654)
Supplement: S1 Table — (PDF) [file pone.0162654.s001.pdf]

Isolate ID	SCCmec type	Clonal complex	Strain	METHICILLIN RESISTANCE AND SCCmec TYPING													
				glycerophosphoryl diester phosphodi-esterase, associated with mecA	Modified penicillin binding protein (PBP2a)	truncated signal transducer protein MecR1	signal transducer protein MecR1	methicillin-resistance regulatory protein	phenol soluble modulin	CsoR-like sulfur transferase-regulated genes B/metallo-beta-lactamase superfamily protein (SCCmec II/III)	homolog of xylose repressor, associated with SCCmec-elements	alternate mec gene	betalactamase from SCCmec XI element	Plasmin-sensitive surface protein	Truncated 3-hydroxy-3-methyl-glutaryl CoA synthase (SCCmec I, Subtyping SCCmec II, IV, V)	CsoR-like sulfur transferase-regulated genes B/ metallo-b-lactamase superfamily protein (SCCmec I, IV a, V und X) (SCCmec I, IV	Putative protein (SCCmec I und IV/ACME, WA40)
				ugpQ	mecA	delta_mecR	mecR	mecI	PSMmec	cstB-SCC1 (ex Q2G1R6)	xylR/mecR2	mecC	blaZ (SCCmec XI)	plsSCC (COL)	mvaS-SCC	cstB-scc2, ex Q2G1R6	Q9S0M4
VIRTUAL HYBRIDISATION for COL, GenBank CP000046	SCCmec I (COL)	CC8	ST250-MRSA-I, Early/Ancestral MRSA	POS	POS	POS	NEG	NEG	NEG	NEG	NEG	NEG	NEG	POS	POS	POS	POS
Reference strain COL	SCCmec I (COL)	CC8	ST250-MRSA-I, Early/Ancestral MRSA	POS	POS	POS	NEG	NEG	NEG	NEG	NEG	NEG	NEG	POS	POS	POS	POS
UKD-0007063	SCCmec I (COL)	CC5	CC5-MRSA-I, South German EMRSA/Italian Clone	POS	POS	POS	AMB	NEG	NEG	NEG	AMB		NEG	POS	POS	POS	POS
UKD-0220759	SCCmec I (COL)	CC5	CC5-MRSA-I, South German EMRSA/Italian Clone	POS	POS	POS	NEG	NEG	NEG	NEG	NEG		NEG	POS	POS	POS	POS
UKD-0308986	SCCmec I (COL)	CC5	CC5-MRSA-I, South German EMRSA/Italian Clone	POS	POS	POS	NEG	NEG	NEG	NEG	AMB		NEG	POS	POS	POS	POS
UKD-0500009	SCCmec I (COL)	CC5	CC5-MRSA-I, South German EMRSA/Italian Clone	POS	POS	POS	NEG	NEG	NEG	NEG	NEG	NEG	NEG	POS	POS	POS	POS
UKD-0508483	SCCmec I (COL)	CC5	CC5-MRSA-I, South German EMRSA/Italian Clone	POS	POS	POS	NEG	NEG	NEG	NEG	NEG		NEG	POS	POS	POS	POS
UKD-0519001	SCCmec I (COL)	CC5	CC5-MRSA-I, South German EMRSA/Italian Clone	POS	POS	POS	NEG	NEG	NEG	NEG	NEG		NEG	POS	POS	POS	POS
UKD-0701876	SCCmec I (COL)	CC5	CC5-MRSA-I, South German EMRSA/Italian Clone	POS	POS	POS	NEG	NEG	NEG	NEG	NEG	NEG	NEG	POS	POS	POS	POS
UKD-0708865	SCCmec I (COL)	CC5	CC5-MRSA-I, South German EMRSA/Italian Clone	POS	POS	POS	NEG	NEG	NEG	NEG	NEG		NEG	POS	POS	POS	POS
UKD-1001782	SCCmec I (COL)	CC5	CC5-MRSA-I, South German EMRSA/Italian Clone	POS	POS	POS	NEG	NEG	NEG	NEG	NEG		NEG	POS	POS	POS	POS
Prediction for PSP1996, GenBank ANHU	SCCmec I (PSP1996)	CC8	ST247-MRSA-I, North German/Iberian EMRSA	POS	POS	POS	NEG	NEG	NEG	NEG	NEG	NEG	NEG	POS	NEG	POS	POS
UKD-0261727	SCCmec I (PSP1996)	CC8	ST247-MRSA-I, North German/Iberian EMRSA	POS	POS	POS	NEG	NEG	NEG	NEG	NEG		NEG	POS	NEG	POS	POS
VIRTUAL HYBRIDISATION for MRSA7 : VIRTUAL HYBRIL	SCCmec I+fus+tirS (Geraldine Clone)	CC5	ST5-MRSA-[I+fus], Geraldine Clone	POS	POS	POS	NEG	NEG	NEG	NEG	NEG	NEG	NEG	NEG	POS	NEG	NEG
UKD-1221930	SCCmec I+fus+tirS (Geraldine Clone)	CC5	ST5-MRSA-[I+fus], Geraldine Clone	POS	POS	POS	NEG	NEG	NEG	NEG	NEG		NEG	NEG	POS	NEG	NEG
Prediction for N315, GenBank BA000018.3	SCCmec II (N315)	CC5	ST5/ST225-MRSA-II, Rhine-Hesse EMRSA/New York-Japan Clone	POS	POS	POS	POS	POS	POS	POS	POS	NEG	NEG	NEG	POS	NEG	NEG
Reference strain N315	SCCmec II (N315)	CC5	ST5/ST225-MRSA-II, Rhine-Hesse EMRSA/New York-Japan Clone	POS	POS	POS	POS	POS	POS	POS	POS	NEG	POS	NEG	POS	NEG	NEG
Prediction for MRSA252, GenBank BX571856.1	SCCmec II (N315)	CC30	ST36/39-MRSA-II, UK-EMRSA-16	POS	POS	POS	POS	POS	POS	POS	POS	NEG	NEG	NEG	POS	NEG	NEG
Reference strain Sanger 252	SCCmec II (N315)	CC30	ST36/39-MRSA-II, UK-EMRSA-16	POS	POS	POS	POS	POS	POS	POS	POS		NEG	NEG	POS	NEG	NEG
UKD-0230792	SCCmec II (N315)	CC30	ST36/39-MRSA-II, UK-EMRSA-16	POS	POS	POS	POS	POS	POS	POS	POS		NEG	NEG	POS	NEG	NEG
Prediction for Mu50, GenBank BA000017.4	SCCmec II (N315)	CC5	ST5/ST225-MRSA-II, Rhine-Hesse EMRSA/New York-Japan Clone	POS	POS	POS	POS	POS	POS	POS	POS	NEG	NEG	NEG	POS	NEG	NEG
Reference strain MU50	SCCmec II (N315)	CC5	ST5/ST225-MRSA-II, Rhine-Hesse EMRSA/New York-Japan Clone	POS	POS	POS	POS	POS	POS	POS	POS	NEG	NEG	NEG	POS	NEG	NEG
Prediction for JH1, GenBank CP000736.1	SCCmec II (JH1/JH9)	CC5	ST5/ST225-MRSA-II, Rhine-Hesse EMRSA/New York-Japan Clone	POS	POS	POS	POS	POS	POS	POS	POS	NEG	NEG	NEG	NEG	NEG	NEG
Prediction for JH9, GenBank CP000703.1	SCCmec II (JH1/JH9)	CC5	ST5/ST225-MRSA-II, Rhine-Hesse EMRSA/New York-Japan Clone	POS	POS	POS	POS	POS	POS	POS	POS	NEG	NEG	NEG	NEG	NEG	NEG
Reference strain Dublin-DSH_AR07.4_0237	SCCmec II (JH1/JH9)		ST5/ST225-MRSA-II, Rhine-Hesse EMRSA/New York-Japan Clone	POS	POS	POS	POS	POS	POS	POS	POS	NEG	NEG	NEG	NEG	NEG	NEG
Reference strain NARSA_382 / USA100	SCCmec II (JH1/JH9)	CC5	ST5/ST225-MRSA-II, Rhine-Hesse EMRSA/New York-Japan Clone	POS	POS	POS	POS	POS	POS	POS	POS	neg	NEG	NEG	NEG	NEG	NEG
UKD-0046921	SCCmec II (JH1/JH9)	CC5	ST5/ST225-MRSA-II, Rhine-Hesse EMRSA/New York-Japan Clone	POS	POS	POS	POS	POS	POS	POS	POS		NEG	NEG	NEG	NEG	NEG
UKD-0333415	SCCmec II (JH1/JH9)	CC5	ST5/ST225-MRSA-II, Rhine-Hesse EMRSA/New York-Japan Clone	POS	POS	POS	POS	POS	POS	POS	POS		NEG	NEG	NEG	NEG	NEG
UKD-0341645	SCCmec II (JH1/JH9)	CC5	ST5/ST225-MRSA-II, Rhine-Hesse EMRSA/New York-Japan Clone	POS	POS	POS	POS	POS	POS	POS	POS		NEG	NEG	NEG	NEG	NEG
UKD-0504626	SCCmec II (JH1/JH9)	CC5	ST5/ST225-MRSA-II, Rhine-Hesse EMRSA/New York-Japan Clone	POS	POS	POS	POS	POS	POS	POS	POS		NEG	NEG	NEG	NEG	NEG
UKD-0507581	SCCmec II (JH1/JH9)	CC5	ST5/ST225-MRSA-II, Rhine-Hesse EMRSA/New York-Japan Clone	POS	POS	POS	POS	POS	POS	POS	POS		NEG	NEG	NEG	NEG	NEG
UKD-0507903	SCCmec II (JH1/JH9)	CC5	ST5/ST225-MRSA-II, Rhine-Hesse EMRSA/New York-Japan Clone	POS	POS	POS	POS	POS	POS	POS	POS		NEG	NEG	NEG	NEG	NEG
UKD-0700817	SCCmec II (JH1/JH9)	CC5	ST5/ST225-MRSA-II, Rhine-Hesse EMRSA/New York-Japan Clone	POS	POS	POS	POS	POS	POS	POS	POS		NEG	NEG	NEG	NEG	NEG
UKD-1014561	SCCmec II (JH1/JH9)	CC5	ST5/ST225-MRSA-II, Rhine-Hesse EMRSA/New York-Japan Clone	POS	POS	POS	POS	POS	POS	POS	POS		NEG	NEG	NEG	NEG	NEG
UKD-1037507	SCCmec II (JH1/JH9)	CC5	ST5/ST225-MRSA-II, Rhine-Hesse EMRSA/New York-Japan Clone	POS	POS	AMB	POS	POS	POS	POS	POS		NEG	NEG	NEG	NEG	NEG
UKD-1038321	SCCmec II (JH1/JH9)	CC5	ST5/ST225-MRSA-II, Rhine-Hesse EMRSA/New York-Japan Clone	POS	POS	POS	POS	POS	POS	POS	POS		NEG	NEG	NEG	NEG	NEG
UKD-1111608	SCCmec II (JH1/JH9)	CC5	ST5/ST225-MRSA-II, Rhine-Hesse EMRSA/New York-Japan Clone	POS	POS	POS	POS	POS	POS	POS	POS		NEG	NEG	NEG	NEG	NEG
UKD-12753135	SCCmec II (JH1/JH9)	CC5	ST5/ST225-MRSA-II, Rhine-Hesse EMRSA/New York-Japan Clone	POS	POS	POS	POS	POS	POS	POS	POS		NEG	NEG	NEG	NEG	NEG
UKD-1312816	SCCmec II (JH1/JH9)	CC5	ST5/ST225-MRSA-II, Rhine-Hesse EMRSA/New York-Japan Clone	POS	POS	POS	POS	POS	POS	POS	POS	NEG	NEG	NEG	NEG	NEG	NEG
UKD-1401623	SCCmec II (JH1/JH9)	CC5	ST5/ST225-MRSA-II, Rhine-Hesse EMRSA/New York-Japan Clone	POS	POS	POS	POS	POS	POS	POS	POS	NEG	NEG	NEG	NEG	NEG	NEG
UKD-15571943	SCCmec II+czrC+ccrA/B-4	CC5	CC5-MRSA-[II+ccrAB4]	POS	POS	POS	POS	POS	POS	POS	POS	NEG	NEG	NEG	NEG	NEG	NEG
UKD-15576888	SCCmec II+czrC+ccrA/B-4	CC5	CC5-MRSA-[II+ccrAB4]	POS	POS	POS	POS	POS	POS	POS	POS	NEG	NEG	NEG	NEG	NEG	NEG
UKD-14576461	SCCmec II+speG+czrC+ccrA/B-4	CC5	CC5-MRSA-[II+ccrAB4]	POS	POS	POS	POS	POS	POS	POS	POS	NEG	NEG	NEG	NEG	NEG	NEG
UKD-15581649	SCCmec II+speG+czrC+ccrA/B-4	CC5	CC5-MRSA-[II+ccrAB4]	POS	POS	POS	POS	POS	POS	POS	POS	NEG	NEG	NEG	NEG	NEG	NEG
Prediction for CIGC345D, GenBank AHVO	SCCmec II A/B/D	CC8	ST8-MRSA-IIA/B/D, Irish AR13/14	POS	POS	POS	POS	POS	POS	POS	POS	NEG	NEG	NEG	POS	NEG	NEG
UKD-12K00171	SCCmec II A/B/D	CC8	ST8-MRSA-IIA/B/D, Irish AR13/14	POS	POS	POS	POS	POS	POS	POS	POS	NEG	NEG	NEG	POS	NEG	NEG
Prediction for JKD6008, GenBank CP002120.1	SCC [mec III+Cd] (JKD6008)	CC8 (ST239)	ST239-MRSA-III+ccrC, Vienna/Hungarian/Brazilian Clone	POS	POS	POS	POS	POS	POS	POS	POS	NEG	NEG	NEG	POS	NEG	NEG
Prediction for JKD6009, GenBank ABSA	SCC [mec III+Cd] (JKD6008)	CC8 (ST239)	ST239-MRSA-III+ccrC, Vienna/Hungarian/Brazilian Clone	POS	POS	POS	POS	POS	POS	POS	POS	NEG	NEG	NEG	POS	NEG	NEG
Reference strain ATCC_33592	SCC [mec III+Cd] (ATCC33592)	CC8 (ST239)	ST239-MRSA-III+ccrC, Vienna/Hungarian/Brazilian Clone	POS	POS	POS	POS	POS	POS	POS	POS	neg	NEG	NEG	POS	NEG	NEG
Prediction for PPUKM-775-2009, GenBank AMRE	SCC [mec III+Cd+ccrC] (PPUKM-775-2009)	CC8 (ST239)	ST239-MRSA-[III+ccrC], Vienna/Hungarian/Brazilian Clone	POS	POS	POS	POS	POS	POS	POS	POS	NEG	NEG	NEG	NEG	NEG	NEG
Prediction for T0131, GenBank CP002643.1	SCC [mec III+Cd+ccrC] (T0131)	CC8 (ST239)	ST239-MRSA-[III+ccrC], Vienna/Hungarian/Brazilian Clone	POS	POS	POS	POS	POS	POS	POS	POS	NEG	NEG	NEG	POS	NEG	NEG
Prediction for XN108, GenBank CP007447.1	SCC [mec III+Cd+ccrC] (XN108)	CC8 (ST239)	ST239-MRSA-[III+ccrC], Vienna/Hungarian/Brazilian Clone	POS	POS	POS	POS	POS	POS	POS	POS	NEG	NEG	NEG	POS	NEG	NEG
Prediction for CN79, GenBank ANCJ	SCC [mec III+Cd+ccrC] (CN79)	CC8 (ST239)	ST239-MRSA-[III+ccrC], Vienna/Hungarian/Brazilian Clone	POS	POS	POS	POS	POS	POS	POS	POS	NEG	NEG	NEG	POS	NEG	NEG
Prediction for 16K, GenBank BABZ	SCC [mec III+Cd+ccrC] (CN79)	CC8 (ST239)	ST239-MRSA-[III+ccrC], Vienna/Hungarian/Brazilian Clone	POS	POS	POS	POS	POS	POS	POS	POS	NEG	NEG	NEG	POS	NEG	NEG
UKD-0835987	SCC [mec III+Cd+ccrC] (CN79)	CC8 (ST239)	ST239-MRSA-[III+ccrC], Vienna/Hungarian/Brazilian Clone	POS	POS	POS	POS	POS	POS	POS	POS		NEG	NEG	POS	NEG	NEG
UKD-16581769	SCCmec III+Cd+ccrC	CC8 (ST239)	ST239-MRSA-[III+ccrC], Vienna/Hungarian/Brazilian Clone	POS	POS	POS	POS	POS	POS	POS	POS	NEG	NEG	NEG	POS	NEG	POS
Prediction for Bmb9393, GenBank CP005288.1	SCCmec III+Cd/Hg+ccrC (Bmb9393)	CC8 (ST239)	ST239-MRSA-[III+SCCmer+ccrC], Vienna/Hungarian/Brazilian Clone	POS	POS	POS	POS	POS	POS	POS	POS	NEG	NEG	NEG	POS	NEG	NEG
UKD-15574263	SCCmec III+Cd/Hg+ccrC (Bmb9393)	CC8 (ST239)	ST239-MRSA-[III+SCCmer+ccrC], Vienna/Hungarian/Brazilian Clone	POS	POS	POS	POS	POS	POS	POS	POS	NEG	NEG	NEG	POS	NEG	NEG
Prediction for SK1585, GenBank AYLT	SCCmec III+ccrC+Cd/Hg (SK1585)	CC8 (ST239)	ST239-MRSA-[III+SCCmer+ccrC], Vienna/Hungarian/Brazilian Clone	POS	POS	POS	POS	POS	POS	POS	POS	NEG	NEG	NEG	POS	NEG	NEG
UKD-105861	SCCmec III+ccrC+Cd/Hg (SK1585)	CC8 (ST239)	ST239-MRSA-[III+SCCmer+ccrC], Vienna/Hungarian/Brazilian Clone	POS	POS	POS	POS	POS	POS	POS	POS		NEG	NEG	POS	NEG	NEG
UKD-134572	SCCmec III+ccrC+Cd/Hg (SK1585)	CC8 (ST239)	ST239-MRSA-[III+SCCmer+ccrC], Vienna/Hungarian/Brazilian Clone	POS	POS	POS	POS	POS	POS	POS	POS		NEG	NEG	POS	NEG	NEG
UKD-139143	SCCmec III+ccrC+Cd/Hg (SK1585)	CC8 (ST239)	ST239-MRSA-[III+SCCmer+ccrC], Vienna/Hungarian/Brazilian Clone	POS	POS	POS	POS	POS	POS	POS	POS		NEG	NEG	POS	NEG	NEG
Prediction for TW20, GenBank FN433596.1	SCC [mec III+Cd/Hg+ccrC] (TW20)	CC8 (ST239)	ST239-MRSA-[III+SCCmer+ccrC], Vienna/Hungarian/Brazilian Clone	POS	POS	POS	POS	POS	POS	POS	POS	NEG	NEG	NEG	POS	NEG	NEG
Prediction for ATCC BAA-39, GenBank AEEK	SCC [mec III+Cd/Hg+ccrC] (TW20)	CC8 (ST239)	ST239-MRSA-[III+SCCmer+ccrC], Vienna/Hungarian/Brazilian Clone	POS	POS	POS	POS	POS	POS	POS	POS	NEG	NEG	NEG	POS	NEG	NEG

Isolate ID	SCCmec type	Clonal complex	Strain	METHICILLIN RESISTANCE AND SCCmec TYPING													
				glycerophosphoryl diester phosphodi-esterase, associated with mecA	Modified penicillin binding protein (PBP2a)	truncated signal transducer protein MecR1	signal transducer protein MecR1	methicillin-resistance regulatory protein	phenol soluble modulin	CsoR-like sulfur transferase-regulated genes B/metallo-beta-lactamase superfamily protein (SCCmec II/III)	homolog of xylose repressor, associated with SCCmec-elements	alternate mec gene	betalactamase from SCCmec XI element	Plasmin-sensitive surface protein	Truncated 3-hydroxy-3-methyl-glutaryl CoA synthase (SCCmec I, Subtyping SCCmec II, IV, V)	CsoR-like sulfur transferase-regulated genes B/ metallo-b-lactamase superfamily protein (SCCmec I, IV a, V und X) (SCCmec I, IV	Putative protein (SCCmec I und IV/ACME, WA40)
				ugpQ	mecA	delta_mecR	mecR	mecI	PSMmec	cstB-SCC1 (ex Q2G1R6)	xylR/mecR2	mecC	blaZ (SCCmec XI)	plsSCC (COL)	mvaS-SCC	cstB-scc2, ex Q2G1R6	Q9S0M4
Prediction for MW2, GenBank BA000033.2	SCCmec IVa (MW2)	CC1	CC1-MRSA-IV (PVL+), USA400	POS	POS	POS	NEG	NEG	NEG	NEG	NEG	NEG	NEG	NEG	POS	POS	NEG
Reference strain MW2	SCCmec IVa (MW2)	CC1	CC1-MRSA-IV (PVL+), USA400	POS	POS	POS	NEG	NEG	NEG	NEG	NEG	NEG	NEG	NEG	POS	POS	NEG
Reference strain CA05 (JCSC1968)	SCCmec IVa (MW2)	CC45	CC45-MRSA-IV (PVL+)	POS	POS	POS	NEG	NEG	NEG	NEG	NEG	NEG	NEG	NEG	POS	POS	NEG
UKD-0127106	SCCmec IVa (MW2)	CC1	CC1-MRSA-IV, WA MRSA-1/57	POS	POS	POS	NEG	NEG	NEG	NEG	NEG		NEG	NEG	POS	POS	NEG
UKD-0701446	SCCmec IVa (MW2)	CC1	CC1-MRSA-IV, WA MRSA-1/57	POS	POS	POS	NEG	NEG	NEG	NEG	NEG		NEG	NEG	POS	POS	NEG
UKD-0947486	SCCmec IVa (MW2)	CC1	CC1-MRSA-IV, WA MRSA-1/57	POS	POS	POS	NEG	NEG	NEG	NEG	NEG		NEG	NEG	POS	POS	NEG
UKD-1031910	SCCmec IVa (MW2)	CC1	CC1-MRSA-IV, WA MRSA-1/57	POS	POS	POS	NEG	NEG	NEG	NEG	NEG		NEG	NEG	POS	POS	NEG
UKD-14571328	SCCmec IVa (MW2)	CC1	CC1-MRSA-IV, WA MRSA-1/57	POS	POS	POS	NEG	NEG	NEG	NEG	NEG	NEG	NEG	NEG	POS	POS	NEG
UKD-03003012	SCCmec IVa (MW2)	CC5	CC5-MRSA-IV, Paediatric clone	POS	POS	POS	NEG	NEG	NEG	NEG	NEG	NEG	NEG	NEG	POS	POS	NEG
UKD-15582582	SCCmec IVa (MW2)	CC6	CC6-MRSA-IV, WA MRSA-51	POS	POS	POS	NEG	NEG	NEG	NEG	NEG	NEG	NEG	NEG	POS	POS	NEG
UKD-16574230	SCCmec IVa (MW2)	CC6	CC6-MRSA-IV, WA MRSA-51	POS	POS	POS	NEG	NEG	NEG	NEG	NEG	NEG	NEG	NEG	POS	POS	NEG
UKD-16582560	SCCmec IVa (MW2)	CC6	CC6-MRSA-IV, WA MRSA-51	POS	POS	POS	NEG	NEG	NEG	NEG	NEG	NEG	NEG	NEG	POS	POS	NEG
UKD-0802855	SCCmec IVa (MW2)	CC7	CC7-MRSA-IV	POS	POS	POS	NEG	NEG	NEG	NEG	NEG		NEG	NEG	POS	POS	NEG
KHDN-10405258	SCCmec IVa (MW2)	CC8	CC8-MRSA-IV (PVL+), ACME-neg-/Spanish or Latin American USA300	POS	POS	POS	NEG	NEG	NEG	NEG	NEG	NEG	NEG	NEG	POS	POS	NEG
UKD-15573497	SCCmec IVa (MW2)	CC22	CC22-MRSA-IV (PVL+)	POS	POS	POS	NEG	NEG	NEG	NEG	NEG	NEG	NEG	NEG	POS	POS	NEG
UKD-0634729	SCCmec IVa (MW2)	CC30	CC30-MRSA-IV (PVL+), Southwest Pacific Clone	POS	POS	POS	NEG	NEG	NEG	NEG	NEG		NEG	NEG	POS	POS	NEG
UKD-1119771	SCCmec IVa (MW2)	CC30	CC30-MRSA-IV (PVL+), Southwest Pacific Clone	POS	POS	POS	NEG	NEG	NEG	NEG	NEG		NEG	NEG	POS	POS	NEG
KHDN-10411458	SCCmec IVa (MW2)	CC30	CC30-MRSA-IV (PVL+), Southwest Pacific Clone	POS	POS	POS	NEG	NEG	NEG	NEG	NEG	NEG	NEG	NEG	POS	POS	NEG
KHDN-398609	SCCmec IVa (MW2)	CC30	CC30-MRSA-IV (PVL+), Southwest Pacific Clone	POS	POS	POS	NEG	NEG	NEG	NEG	NEG		NEG	NEG	POS	POS	NEG
KHDN-01081998	SCCmec IVa (MW2)	CC30	CC30-MRSA-IV (PVL+), Southwest Pacific Clone	POS	POS	POS	NEG	NEG	NEG	NEG	NEG		NEG	NEG	POS	POS	NEG
KHDN-10399223	SCCmec IVa (MW2)	CC30	CC30-MRSA-IV (PVL+), Southwest Pacific Clone	POS	POS	POS	NEG	NEG	NEG	NEG	NEG		NEG	NEG	POS	POS	NEG
KHDN-W261158	SCCmec IVa (MW2)	CC30	CC30-MRSA-IV (PVL+), Southwest Pacific Clone	POS	POS	POS	NEG	NEG	NEG	NEG	NEG		NEG	NEG	POS	POS	NEG
UKD-0002046	SCCmec IVa (MW2)	CC45 [agr I]	CC45-MRSA-IV, Berlin EMRSA	POS	POS	POS	NEG	NEG	NEG	NEG	NEG		NEG	NEG	POS	POS	NEG
UKD-0029618	SCCmec IVa (MW2)	CC45 [agr I]	CC45-MRSA-IV, Berlin EMRSA	POS	POS	POS	NEG	NEG	NEG	NEG	NEG		NEG	NEG	POS	POS	NEG
UKD-0043085	SCCmec IVa (MW2)	CC45 [agr I]	CC45-MRSA-IV, Berlin EMRSA	POS	POS	POS	NEG	NEG	NEG	NEG	NEG		NEG	NEG	POS	POS	NEG
UKD-0047053	SCCmec IVa (MW2)	CC45 [agr I]	CC45-MRSA-IV, Berlin EMRSA	POS	POS	POS	NEG	NEG	NEG	NEG	NEG		NEG	NEG	POS	POS	NEG
UKD-0047429	SCCmec IVa (MW2)	CC45 [agr I]	CC45-MRSA-IV, Berlin EMRSA	POS	POS	POS	NEG	NEG	NEG	NEG	NEG		NEG	NEG	POS	POS	NEG
UKD-0112465	SCCmec IVa (MW2)	CC45 [agr I]	CC45-MRSA-IV, Berlin EMRSA	POS	POS	POS	NEG	NEG	NEG	NEG	NEG		NEG	NEG	POS	POS	NEG
UKD-0103483	SCCmec IVa (MW2)	CC45 [agr I]	CC45-MRSA-IV, Berlin EMRSA	POS	POS	POS	NEG	NEG	NEG	NEG	NEG		NEG	NEG	POS	POS	NEG
UKD-0305014	SCCmec IVa (MW2)	CC45 [agr I]	CC45-MRSA-IV, Berlin EMRSA	POS	POS	POS	NEG	NEG	NEG	NEG	NEG		NEG	NEG	POS	POS	NEG
UKD-0401119	SCCmec IVa (MW2)	CC45 [agr I]	CC45-MRSA-IV, Berlin EMRSA	POS	POS	POS	NEG	NEG	NEG	NEG	NEG		NEG	NEG	POS	POS	NEG
UKD-0537969	SCCmec IVa (MW2)	CC45 [agr I]	CC45-MRSA-IV, Berlin EMRSA	POS	POS	POS	NEG	NEG	NEG	NEG	NEG		NEG	NEG	POS	POS	NEG
UKD-0703373	SCCmec IVa (MW2)	CC45 [agr I]	CC45-MRSA-IV, Berlin EMRSA	POS	POS	POS	NEG	NEG	NEG	NEG	NEG		NEG	NEG	POS	POS	NEG
UKD-0813653	SCCmec IVa (MW2)	CC45 [agr I]	CC45-MRSA-IV, Berlin EMRSA	POS	POS	POS	NEG	NEG	NEG	NEG	NEG		NEG	NEG	POS	POS	NEG
UKD-0839175	SCCmec IVa (MW2)	CC45 [agr I]	CC45-MRSA-IV, Berlin EMRSA	POS	POS	POS	NEG	NEG	NEG	NEG	NEG	NEG	NEG	NEG	POS	POS	NEG
UKD-1108422	SCCmec IVa (MW2)	CC45 [agr I]	CC45-MRSA-IV, Berlin EMRSA	POS	POS	POS	NEG	NEG	NEG	NEG	NEG		NEG	NEG	POS	POS	NEG
UKD-1380645	SCCmec IVa (MW2)	CC45 [agr I]	CC45-MRSA-IV, Berlin EMRSA	POS	POS	POS	NEG	NEG	NEG	NEG	NEG	NEG	NEG	NEG	POS	POS	NEG
UKD-148016	SCCmec IVa (MW2)	CC45 [agr I]	CC45-MRSA-IV, Berlin EMRSA	POS	POS	POS	NEG	NEG	NEG	NEG	NEG	NEG	NEG	NEG	POS	POS	NEG
UKD-16571773	SCCmec IVa (MW2)	CC45 [agr I]	CC45-MRSA-IV, Berlin EMRSA	POS	POS	POS	NEG	NEG	NEG	NEG	NEG	NEG	NEG	NEG	POS	POS	NEG
KHDN-10407738	SCCmec IVa (MW2)	CC45 [ST617]	ST617-MRSA-IV	POS	POS	POS	NEG	NEG	NEG	NEG	NEG	NEG	NEG	NEG	POS	POS	NEG
UKD-0853670	SCCmec IVa (MW2)	CC88	CC88-MRSA-IV, WA MRSA-2	POS	POS	POS	NEG	NEG	NEG	NEG	NEG		NEG	NEG	POS	POS	NEG
UKD-1417352	SCCmec IVa (MW2)	CC88	CC88-MRSA-IV (PVL+)	POS	POS	POS	NEG	NEG	NEG	NEG	NEG	NEG	NEG	NEG	POS	POS	NEG
UKD-15571283	SCCmec IVa (MW2)	CC88	CC88-MRSA-IV (PVL+)	POS	POS	POS	AMB	NEG	NEG	NEG	NEG	NEG	NEG	NEG	POS	POS	NEG
KHDN-10406719	SCCmec IVa (MW2)	ST93	ST93-MRSA-IV (PVL+), Queensland Clone	POS	POS	POS	NEG	NEG	NEG	NEG	NEG	NEG	NEG	NEG	POS	POS	NEG
KHDN-0060294	SCCmec IVa (MW2)	ST93	ST93-MRSA-IV (PVL+), Queensland Clone	POS	POS	POS	NEG	NEG	NEG	NEG	NEG	NEG	NEG	NEG	POS	POS	NEG
UKD-0734190	SCCmec IVa (MW2)	CC97	CC97-MRSA-IV, WA MRSA-54/63	POS	POS	POS	NEG	NEG	NEG	NEG	NEG		NEG	NEG	POS	POS	NEG
UKD-13006983	SCCmec IVa (MW2)	CC97	CC97-MRSA-IV, WA MRSA-54/63	POS	POS	POS	NEG	NEG	NEG	NEG	NEG	NEG	NEG	NEG	POS	POS	NEG
Prediction for H131520133	SCCmec IVa (H131520133)	CC152	CC152-MRSA-IV (PVL+)	POS	POS	POS	NEG	NEG	NEG	NEG	NEG	NEG	NEG	NEG	NEG	POS	NEG
KHDN-325892	SCCmec IVa (H131520133)	CC30	CC30-MRSA-IV (PVL+), Southwest Pacific Clone	POS	POS	POS	NEG	NEG	NEG	NEG	NEG		NEG	NEG	NEG	POS	NEG
UKD-15580207	SCCmec IVa (H131520133)	CC88	CC88-MRSA-IV (PVL+)	POS	POS	POS	NEG	NEG	NEG	NEG	NEG	NEG	NEG	NEG	NEG	POS	NEG
UKD-0521159	SCCmec Iva	CC8	CC8-MRSA-IV, USA500	POS	POS	POS	NEG	NEG	NEG	NEG	NEG		NEG	NEG	POS	POS	NEG
Prediction for CMFT503 : HF569113.1	SCCmec IVa (CMFT503)			POS	POS	POS	NEG	NEG	NEG	NEG	NEG	NEG	NEG	NEG	POS	POS	NEG
Prediction for 21343, GenBank AHKV	SCCmec IVa (CMFT503)	CC88	CC88-MRSA-IV (etA+)	POS	POS	POS	NEG	NEG	NEG	NEG	NEG	NEG	NEG	NEG	POS	POS	NEG
UKD-1420740	SCCmec IVa (CMFT503)	CC22	CC22-MRSA-IV (fnbB+), UK-EMRSA-15/Barnim EMRSA-like MRSA	POS	POS	POS	NEG	NEG	NEG	NEG	NEG	NEG	NEG	NEG	POS	POS	NEG
UKD-15575124	SCCmec IVa (CMFT503)	CC22	CC22-MRSA-IV (tst1+)	POS	POS	POS	NEG	NEG	NEG	NEG	NEG	NEG	NEG	NEG	POS	POS	NEG
UKD-16584947	SCCmec IVa (CMFT503)	CC22	CC22-MRSA-IV (tst1+)	POS	POS	POS	NEG	NEG	NEG	NEG	NEG	NEG	NEG	NEG	POS	POS	NEG
UKD-16575077	SCCmec IVa (CMFT503)	CC88	CC88-MRSA-IV, WA MRSA-2	POS	POS	POS	NEG	NEG	NEG	NEG	NEG	NEG	NEG	NEG	POS	POS	NEG
Prediction for TCH1516, GenBank CP000730.1	SCCmec IVa+ACME1+Cu (USA300)	CC8	ST8-MRSA-[IV+ACME] (PVL+), USA300	POS	POS	POS	NEG	NEG	NEG	NEG	NEG	NEG	NEG	NEG	POS	POS	NEG
Reference strain USA300-TCH1516	SCCmec IVa+ACME1+Cu (USA300)	CC8	ST8-MRSA-[IV+ACME] (PVL+), USA300	POS	POS	POS	NEG	NEG	NEG	NEG	NEG		NEG	NEG	POS	POS	NEG
Prediction for FPR3757, GenBank CP000255.1	SCCmec IVa+ACME1+Cu (USA300)	CC8	ST8-MRSA-[IV+ACME] (PVL+), USA300	POS	POS	POS	NEG	NEG	NEG	NEG	NEG	NEG	NEG	NEG	POS	POS	NEG
UKD-0516386	SCCmec IVa+ACME1+Cu (USA300)	CC8	ST8-MRSA-[IV+ACME] (PVL+), USA300	POS	POS	POS	NEG	NEG	NEG	NEG	NEG		NEG	NEG	POS	POS	NEG
UKD-0815773	SCCmec IVa+ACME1+Cu (USA300)	CC8	ST8-MRSA-[IV+ACME] (PVL+), USA300	POS	POS	POS	NEG	NEG	NEG	NEG	NEG		NEG	NEG	POS	POS	NEG
UKD-1414654	SCCmec IVa+ACME1+Cu (USA300)	CC8	ST8-MRSA-[IV+ACME] (PVL+), USA300	POS	POS	POS	NEG	NEG	NEG	NEG	NEG	NEG	NEG	NEG	POS	POS	NEG
UKD-0408036	SCCmec IVa+ACME2	CC45 [agr I]	CC45-MRSA-[IV+ACME]	POS	POS	POS	NEG	NEG	NEG	NEG	NEG		NEG	NEG	POS	POS	NEG
UKD-0044035	SCCmec IVa+ccrC (Hannover)	CC8	ST254-MRSA-[IV+ccrC], UK-EMRSA-10/Hannover EMRSA	POS	POS	POS	NEG	NEG	NEG	NEG	NEG		NEG	NEG	POS	POS	NEG
UKD-0136287	SCCmec IVa+ccrC (Hannover)	CC8	ST254-MRSA-[IV+ccrC], UK-EMRSA-10/Hannover EMRSA	POS	POS	POS	NEG	NEG	NEG	NEG	NEG		NEG	NEG	POS	POS	NEG

Isolate ID	SCCmec type	Clonal complex	Strain	METHICILLIN RESISTANCE AND SCCmec TYPING													
				glycerophosphoryl diester phosphodi-esterase, associated with mecA	Modified penicillin binding protein (PBP2a)	truncated signal transducer protein MecR1	signal transducer protein MecR1	methicillin-resistance regulatory protein	phenol soluble modulin	CsoR-like sulfur transferase-regulated genes B/metallo-beta lactamase superfamily protein (SCCmec II/III)	homolog of xylose repressor, associated with SCCmec-elements	alternate mec gene	betalactamase from SCCmec XI element	Plasmin-sensitive surface protein	Truncated 3-hydroxy-3-methyl-glutaryl CoA synthase (SCCmec I, Subtyping SCCmec II, IV, V)	CsoR-like sulfur transferase-regulated genes B/ metallo-b-lactamase superfamily protein (SCCmec I, IV a, V und X) [illegible]	Putative protein (SCCmec I und IV/ACME, WA40)
				ugpQ	mecA	delta_mecR	mecR	mecI	PSMmec	cstB-SCC1 (ex Q2G1R6)	xylR/mecR2	mecC	blaZ (SCCmec XI)	plsSCC (COL)	mvaS-SCC	cstB-scc2, ex Q2G1R6	Q9S0M4
Prediction for JCSC1978, GenBank AB063173.1	*SCCmec IVb/d/i (JCSC1978/6668/4469)*			*POS*	*POS*	*POS*	*NEG*	*NEG*	*NEG*	*NEG*	*NEG*	*NEG*	*NEG*	*NEG*	*POS*	*NEG*	*NEG*
Reference strain JCSC1978 (8/6-3P)	SCCmec IVb/d/i (JCSC1978/6668/4469)	CC8	CC8-MRSA-IV	POS	POS	POS	NEG	NEG	NEG	NEG	NEG	neg	NEG	NEG	POS	NEG	NEG
Prediction for JCSC6668, GenBank AB425823.1	*SCCmec IVb/d/i (JCSC1978/6668/4469)*			*POS*	*POS*	*POS*	*NEG*	*NEG*	*NEG*	*NEG*	*NEG*	*NEG*	*NEG*	*NEG*	*POS*	*NEG*	*NEG*
Reference strain JCSC4469	SCCmec IVb/d/i (JCSC1978/6668/4469)	CC5	CC5-MRSA-IV, Paediatric clone	POS	POS	POS	NEG	NEG	NEG	NEG	NEG	neg	NEG	NEG	POS	NEG	NEG
UKD-1103429	SCCmec IVb/d/i (JCSC1978/6668/4469)	CC7	CC7-MRSA-IV	POS	POS	POS	NEG	NEG	NEG	NEG	NEG		NEG	NEG	POS	NEG	NEG
UKD-1306141	SCCmec IVb/d/i (JCSC1978/6668/4469)	CC7	CC7-MRSA-IV	POS	POS	POS	NEG	NEG	NEG	NEG	NEG		NEG	NEG	POS	NEG	NEG
UKD-0500089	SCCmec IVb/d/i (JCSC1978/6668/4469)	CC5	CC5-MRSA-IV (sed/j/r+), Paediatric clone	POS	POS	POS	NEG	NEG	NEG	NEG	NEG		NEG	NEG	POS	NEG	NEG
UKD-0501416	SCCmec IVb/d/i (JCSC1978/6668/4469)	CC5	CC5-MRSA-IV (sed/j/r+), Paediatric clone	POS	POS	POS	NEG	NEG	NEG	NEG	NEG		NEG	NEG	POS	NEG	NEG
UKD-0934574	SCCmec IVb/d/i (JCSC1978/6668/4469)	CC5	CC5-MRSA-IV (sed/j/r+), Paediatric clone	POS	POS	POS	NEG	NEG	NEG	NEG	NEG		NEG	NEG	POS	NEG	NEG
UKD-0746779	SCCmec IVb/d/i (JCSC1978/6668/4469)	CC45 [agr I]	CC45-MRSA-IV, Berlin EMRSA	POS	POS	POS	NEG	NEG	NEG	NEG	NEG		NEG	NEG	POS	NEG	NEG
UKD-0812518	SCCmec IVb/d/i (JCSC1978/6668/4469)	CC45 [agr I]	CC45-MRSA-IV, Berlin EMRSA	POS	POS	POS	NEG	NEG	NEG	NEG	NEG	NEG	NEG	NEG	POS	NEG	NEG
Prediction for 21209, GenBank AGRP	*SCCmec IVb/d/i (21209)*	*CC8*	*CC8-MRSA-IV, USA500*	*POS*	*POS*	*POS*	*NEG*	*NEG*	*NEG*	*NEG*	*NEG*	*NEG*	*NEG*	*NEG*	*NEG*	*NEG*	*NEG*
UKD-0233579	SCCmec IVb/d/i (21209)	CC8	CC8-MRSA-IV, USA500	POS	POS	POS	NEG	NEG	NEG	NEG	NEG		NEG	AMB	NEG	NEG	NEG
UKD-0504920	SCCmec IVb/d/i (21209)	CC8	CC8-MRSA-IV, USA500	POS	POS	POS	NEG	NEG	NEG	NEG	NEG		NEG	NEG	NEG	NEG	NEG
UKD-0725999	SCCmecIV b/d/i+Cu	CC30	CC30-MRSA-IV (PVL+), Southwest Pacific Clone	POS	POS	POS	NEG	NEG	NEG	NEG	NEG	NEG	NEG	NEG	POS	NEG	NEG
Prediction for IS-105, GenBank AHLR	*SCCmec IVc (IS-105)*	*CC22*	*ST22-MRSA-IV (PVL+)*	*POS*	*POS*	*POS*	*NEG*	*NEG*	*NEG*	*NEG*	*NEG*	*NEG*	*NEG*	*NEG*	*NEG*	*NEG*	*NEG*
UKD-0402421	SCCmec IVc (IS-105), merA/B+	CC5	CC5-MRSA-IV (sed/j/r+), Paediatric clone	POS	POS	POS	NEG	NEG	NEG	NEG	NEG	NEG	NEG	NEG	NEG	NEG	NEG
UKD-0731319	SCCmec IVc (IS-105), merA/B+	CC5	CC5-MRSA-IV (sed/j/r+), Paediatric clone	POS	POS	POS	NEG	NEG	NEG	NEG	NEG		NEG	NEG	NEG	NEG	NEG
UKD-0733137	SCCmec IVc (IS-105)	CC8	CC8-MRSA-IV (sea+), Lyon Clone/UK-EMRSA-2	POS	POS	POS	NEG	NEG	NEG	NEG	NEG	NEG	NEG	NEG	NEG	NEG	NEG
UKD-0305690	SCCmec IVc (IS-105)	CC22	CC22-MRSA-IV (fnbB+), UK-EMRSA-15/Barnim EMRSA-like MRSA	POS	POS	POS	NEG	NEG	NEG	NEG	NEG		NEG	NEG	NEG	NEG	NEG
UKD-1424706	SCCmec IVc (IS-105)	CC22	CC22-MRSA-IV (PVL+)	POS	POS	POS	NEG	NEG	NEG	NEG	NEG	NEG	NEG	NEG	NEG	NEG	NEG
Prediction for TCH60, GenBank CP002110.1	*SCCmec IVc (TCH60)*	*CC30*	*CC30-MRSA-IV (PVL+), Southwest Pacific Clone*	*POS*	*POS*	*POS*	*NEG*	*NEG*	*NEG*	*NEG*	*NEG*	*NEG*	*NEG*	*NEG*	*POS*	*NEG*	*NEG*
Reference strain JCSC4788	SCCmec IVc (TCH60)	CC8	CC8-MRSA-IV	POS	POS	POS	NEG	NEG	NEG	NEG	NEG	NEG	NEG	NEG	POS	NEG	NEG
UKD-1318318	SCCmec IVc (TCH60)	CC5	CC5-MRSA-IV (PVL+)	POS	POS	POS	NEG	NEG	NEG	NEG	NEG	NEG	NEG	NEG	POS	NEG	NEG
UKD-14580050	SCCmec IVc (TCH60)	CC5	CC5-MRSA-IV (PVL+)	POS	POS	POS	NEG	NEG	NEG	NEG	NEG	NEG	NEG	NEG	POS	NEG	NEG
UKD-08F01314	SCCmec IVc (TCH60)	CC8	CC8-MRSA-IV, UK-EMRSA-14/WA MRSA-5	POS	POS	POS	NEG	NEG	NEG	NEG	NEG		NEG	NEG	POS	NEG	NEG
KHDN-50361518	SCCmec IVc (TCH60)	CC8	CC8-MRSA-IV (sea+), Lyon Clone/UK-EMRSA-2	POS	POS	POS	NEG	NEG	NEG	NEG	NEG	NEG	NEG	NEG	POS	NEG	NEG
UKD-0307912	SCCmec IVc (TCH60)	CC22	CC22-MRSA-IV (fnbB+), UK-EMRSA-15/Barnim EMRSA-like MRSA	POS	POS	POS	AMB	NEG	NEG	NEG	AMB		NEG	NEG	POS	NEG	NEG
UKD-0220390	SCCmec IVc (TCH60)	CC45 [agr I]	CC45-MRSA-IV, Berlin EMRSA	POS	POS	POS	NEG	NEG	NEG	NEG	NEG		NEG	NEG	POS	NEG	NEG
UKD-0311106	SCCmec IVc (TCH60)	CC45 [agr I]	CC45-MRSA-IV, Berlin EMRSA	POS	POS	POS	NEG	NEG	NEG	NEG	NEG		NEG	NEG	POS	NEG	NEG
UKD-0719855	SCCmec IVc (TCH60)	CC45 [agr I]	CC45-MRSA-IV, Berlin EMRSA	POS	POS	POS	NEG	NEG	NEG	NEG	NEG		NEG	NEG	POS	NEG	NEG
UKD-0422079	SCCmec IVc (TCH60)	CC45 [agr I]	CC45-MRSA-IV, Berlin EMRSA	POS	POS	POS	NEG	NEG	NEG	NEG	NEG		NEG	NEG	POS	NEG	NEG
UKD-0409235	SCCmec IVc (TCH60)	CC80	CC80-MRSA-IV (PVL+)	POS	POS	POS	NEG	NEG	NEG	NEG	NEG		NEG	NEG	POS	NEG	NEG
UKD-0409807	SCCmec IVc (TCH60)	CC80	CC80-MRSA-IV (PVL+)	POS	POS	POS	NEG	NEG	NEG	NEG	NEG	NEG	NEG	NEG	POS	NEG	NEG
UKD-0416073	SCCmec IVc (TCH60)	CC80	CC80-MRSA-IV (PVL+)	POS	POS	POS	NEG	NEG	NEG	NEG	NEG		NEG	NEG	POS	NEG	NEG
UKD-0431728	SCCmec IVc (TCH60)	CC80	CC80-MRSA-IV (PVL+)	POS	POS	POS	NEG	NEG	NEG	NEG	NEG		NEG	NEG	POS	NEG	NEG
UKD-1001508	SCCmec IVc (TCH60)	CC80	CC80-MRSA-IV (PVL+)	POS	POS	POS	NEG	NEG	NEG	NEG	NEG		NEG	NEG	POS	NEG	NEG
UKD-1010412	SCCmec IVc (TCH60)	CC80	CC80-MRSA-IV (PVL+)	POS	POS	AMB	NEG	NEG	NEG	NEG	AMB		NEG	NEG	POS	NEG	NEG
UKD-12753005	SCCmec IVc (TCH60)	CC80	CC80-MRSA-IV (PVL+)	POS	POS	POS	NEG	NEG	NEG	NEG	NEG		NEG	NEG	POS	NEG	NEG
UKD-15574809	SCCmec IVc (TCH60)	CC80	CC80-MRSA-IV (PVL+)	POS	POS	POS	NEG	NEG	NEG	NEG	NEG	NEG	NEG	NEG	POS	NEG	NEG
KHDN-16120291	SCCmec IVc (TCH60)	CC88	CC88-MRSA-IV, WA MRSA-2	POS	POS	POS	NEG	NEG	NEG	NEG	NEG	NEG	NEG	NEG	POS	NEG	NEG
Prediction for WW2703/97, GenBank ACSW	*SCCmec IVc (WW2703/97)*	*CC30*	*CC30-MRSA-IV (PVL-/tst1-)*	*POS*	*POS*	*POS*	*NEG*	*NEG*	*NEG*	*NEG*	*NEG*	*NEG*	*NEG*	*NEG*	*POS*	*NEG*	*NEG*
UKD-14580088	SCCmec IVc (WW2703/97)	CC398	CC398-MRSA-IV	POS	POS	POS	NEG	NEG	NEG	NEG	NEG	NEG	NEG	NEG	POS	NEG	NEG
Prediction for SA_ST125_MupR, GenBank ASTH	*SCCmec IVc+speG+ccrAB4 (SA_ST125)*	*CC5*	*CC5-MRSA-[IV+ccrAB4]*	*POS*	*POS*	*POS*	*NEG*	*NEG*	*NEG*	*NEG*	*NEG*	*NEG*	*NEG*	*NEG*	*NEG*	*NEG*	*NEG*
UKD-0439909	SCCmec IVc+speG+ccrAB4 (SA_ST125)	CC5	CC5-MRSA-[IV+ccrAB4]	POS	POS	POS	NEG	NEG	NEG	NEG	NEG		NEG	NEG	NEG	NEG	NEG
Prediction for MRSA177, GenBank AECP	*SCCmec IVc+Cu/Hg (MRSA177)*			*POS*	*POS*	*POS*	*NEG*	*NEG*	*NEG*	*NEG*	*NEG*	*NEG*	*NEG*	*NEG*	*POS*	*NEG*	*NEG*
UKD-1279734	SCCmec IVc+Cu/Hg (MRSA177)	CC8	CC8-MRSA-IV (PVL+), ACME-neg-/Spanish or Latin American USA300	POS	POS	POS	NEG	NEG	NEG	NEG	NEG		NEG	NEG	POS	NEG	NEG
UKD-14580899	SCCmec IVc+Cu/Hg (MRSA177)	CC8	CC8-MRSA-IV (PVL+), ACME-neg-/Spanish or Latin American USA300	POS	POS	POS	NEG	NEG	NEG	NEG	NEG	NEG	NEG	NEG	POS	NEG	NEG
UKD-16002228	SCCmec IVc+Cu/Hg (MRSA177)	CC8	CC8-MRSA-IV (PVL+), ACME-neg-/Spanish or Latin American USA300	POS	POS	POS	NEG	NEG	NEG	NEG	NEG	NEG	NEG	NEG	POS	NEG	NEG
Prediction for HO 5096 0412, GenBank HE681097.1	*SCCmec IVh/j (HO50960412/JCSC6670)*	*CC22*	*CC22-MRSA-IV (fnbB-,sec/l-), UK-EMRSA-15/Barnim EMRSA*	*POS*	*POS*	*POS*	*NEG*	*NEG*	*NEG*	*NEG*	*NEG*	*NEG*	*NEG*	*NEG*	*POS*	*NEG*	*NEG*
Prediction for JCSC6670, GenBank AB425824.1	*SCCmec IVh/j (HO50960412/JCSC6670)*			*POS*	*POS*	*POS*	*NEG*	*NEG*	*NEG*	*NEG*	*NEG*	*NEG*	*NEG*	*NEG*	*POS*	*NEG*	*NEG*
Reference strain Dublin_DSH_E1749	SCCmec IVh/j (HO50960412/JCSC6670)		CC22-MRSA-IV (fnbB-,sec/l+), UK-EMRSA-15/Barnim EMRSA	POS	POS	POS	NEG	NEG	NEG	NEG	NEG	NEG	NEG	NEG	POS	NEG	NEG
UKD-14576248	SCCmec IVh/j (HO50960412/JCSC6670)	CC8	CC8-MRSA-IV, UK-EMRSA-14/WA MRSA-5	POS	POS	POS	NEG	NEG	NEG	NEG	NEG	NEG	NEG	NEG	POS	NEG	NEG
UKD-0120122	SCCmec IVh/j (HO50960412/JCSC6670)	CC22	CC22-MRSA-IV (fnbB-,sec/l+), UK-EMRSA-15/Barnim EMRSA	POS	POS	POS	NEG	NEG	NEG	NEG	NEG		NEG	NEG	POS	NEG	NEG
UKD-0205319	SCCmec IVh/j (HO50960412/JCSC6670)	CC22	CC22-MRSA-IV (fnbB-,sec/l-), UK-EMRSA-15/Barnim EMRSA	POS	POS	POS	NEG	NEG	NEG	NEG	NEG		NEG	NEG	POS	NEG	NEG
UKD-0221259	SCCmec IVh/j (HO50960412/JCSC6670)	CC22	CC22-MRSA-IV (fnbB-,sec/l+), UK-EMRSA-15/Barnim EMRSA	POS	POS	POS	NEG	NEG	NEG	NEG	NEG		NEG	NEG	POS	NEG	NEG
UKD-0418654	SCCmec IVh/j (HO50960412/JCSC6670)	CC22	CC22-MRSA-IV (fnbB-,sec/l-), UK-EMRSA-15/Barnim EMRSA	POS	POS	POS	NEG	NEG	NEG	NEG	NEG	NEG	NEG	NEG	POS	NEG	NEG
UKD-0429721	SCCmec IVh/j (HO50960412/JCSC6670)	CC22	CC22-MRSA-IV (fnbB-,sec/l-), UK-EMRSA-15/Barnim EMRSA	POS	POS	POS	NEG	NEG	NEG	NEG	NEG		NEG	NEG	POS	NEG	NEG
UKD-1116194	SCCmec IVh/j (HO50960412/JCSC6670)	CC22	CC22-MRSA-IV (fnbB-,sec/l+), UK-EMRSA-15/Barnim EMRSA	POS	POS	POS	NEG	NEG	NEG	NEG	NEG		NEG	NEG	POS	NEG	NEG
UKD-1122367	SCCmec IVh/j (HO50960412/JCSC6670)	CC22	CC22-MRSA-IV (fnbB-,sec/l-), UK-EMRSA-15/Barnim EMRSA	POS	POS	POS	NEG	NEG	NEG	NEG	NEG		NEG	NEG	POS	NEG	NEG
UKD-14012840	SCCmec IVh/j (HO50960412/JCSC6670)	CC22	CC22-MRSA-IV (fnbB-,sec/l+), UK-EMRSA-15/Barnim EMRSA	POS	POS	POS	NEG	NEG	NEG	NEG	NEG	NEG	NEG	NEG	POS	NEG	NEG
UKD-14579833	SCCmec IVh/j (HO50960412/JCSC6670)	CC22	CC22-MRSA-IV (fnbB-,sec/l-), UK-EMRSA-15/Barnim EMRSA	POS	POS	POS	NEG	NEG	NEG	NEG	NEG	NEG	NEG	NEG	POS	NEG	NEG
UKD-15579198	SCCmec IVh/j (HO50960412/JCSC6670)	CC22	CC22-MRSA-IV (fnbB-,sec/l+), UK-EMRSA-15/Barnim EMRSA	POS	POS	POS	NEG	NEG	NEG	NEG	NEG	NEG	NEG	NEG	POS	NEG	NEG
UKD-4052193	SCCmec IVh/j (HO50960412/JCSC6670)	CC22	CC22-MRSA-IV (fnbB-,sec/l+), UK-EMRSA-15/Barnim EMRSA	POS	POS	POS	NEG	NEG	NEG	NEG	NEG	NEG	NEG	NEG	POS	NEG	NEG
UKD-124968	SCCmec IVh/j (HO50960412/JCSC6670)	CC22	CC22-MRSA-IV (fnbB-,sec/l-), UK-EMRSA-15/Barnim EMRSA	POS	POS	POS	NEG	NEG	NEG	NEG	NEG	NEG	NEG	NEG	POS	NEG	NEG
UKD-14572546	SCCmec IVh/j (HO50960412/JCSC6670)	CC22	CC22-MRSA-IV (fnbB+), UK-EMRSA-15/Barnim EMRSA-like MRSA	POS	POS	POS	NEG	NEG	NEG	NEG	NEG	NEG	NEG	NEG	POS	NEG	NEG
UKD-14582287	SCCmec IVh/j (HO50960412/JCSC6670)	CC22	CC22-MRSA-IV (fnbB+), UK-EMRSA-15/Barnim EMRSA-like MRSA	POS	POS	POS	NEG	NEG	NEG	NEG	NEG	NEG	NEG	NEG	POS	NEG	NEG
UKD-14583376	SCCmec IVh/j (HO50960412/JCSC6670)	CC22	CC22-MRSA-IV (fnbB+), UK-EMRSA-15/Barnim EMRSA-like MRSA	POS	POS	POS	NEG	NEG	NEG	NEG	NEG	NEG	NEG	NEG	POS	NEG	NEG
UKD-14583496	SCCmec IVh/j (HO50960412/JCSC6670)	CC22	CC22-MRSA-IV (fnbB+), UK-EMRSA-15/Barnim EMRSA-like MRSA	POS	POS	POS	NEG	NEG	NEG	NEG	NEG	NEG	NEG	NEG	POS	NEG	NEG
UKD-0013324	SCCmec IVh/j (HO50960412/JCSC6670)	CC22	CC22-MRSA-IV (PVL+)	POS	POS	POS	NEG	NEG	NEG	NEG	NEG		NEG	NEG	POS	NEG	NEG
UKD-0125329	SCCmec IVh/j (HO50960412/JCSC6670)	CC22	CC22-MRSA-IV (PVL+)	POS	POS	POS	NEG	NEG	NEG	NEG	NEG		NEG	NEG	POS	NEG	NEG
UKD-0215498	SCCmec IVh/j (HO50960412/JCSC6670)	CC22	CC22-MRSA-IV (PVL+)	POS	POS	POS	NEG	NEG	NEG	NEG	NEG		NEG	NEG	POS	NEG	NEG
Prediction for M08-0126, GenBank FR753166.1	*SCCmec IVh/j+ACME2 (M08-0126)*	*CC22*	*CC22-MRSA-[IV+ACME], UK-EMRSA-15/Dublin variant*	*POS*	*POS*	*POS*	*NEG*	*NEG*	*NEG*	*NEG*	*NEG*	*NEG*	*NEG*	*NEG*	*POS*	*NEG*	*POS*
UKD-1379483I	SCCmec IVh/j+ACME2 (M08-0126)	CC22	CC22-MRSA-[IV+ACME], UK-EMRSA-15/Dublin variant	POS	POS	POS	NEG	NEG	NEG	NEG	NEG		NEG	NEG	POS	NEG	POS
UKD-5150204	SCCmec IV+speG+Cu/czrC+ccrAB4	CC22	CC22-MRSA-[IV+ccrAB4]	POS	POS	POS	NEG	NEG	NEG	NEG	NEG	NEG	NEG	NEG	POS	POS	NEG
UKD-15578423	SCCmec IV+speG+czrC+ccrAB4 var. 1	CC22	CC22-MRSA-[IV+ccrAB4]	POS	POS	POS	NEG	NEG	NEG	NEG	NEG	NEG	NEG	NEG	POS	NEG	NEG
UKD-16582870	SCCmec IV+speG+czrC+ccrAB4 var. 1	CC22	CC22-MRSA-[IV+ccrAB4]	POS	POS	POS	NEG	NEG	NEG	NEG	NEG	NEG	NEG	NEG	POS	NEG	NEG
UKD-15571940	SCCmec IV+speG+czrC+ccrAB4 var. 2	CC22	CC22-MRSA-[IV+ccrAB4]	POS	POS	POS	NEG	NEG	NEG	NEG	NEG	NEG	NEG	NEG	NEG	NEG	NEG

Isolate ID	SCCmec type	Clonal complex	Strain	METHICILLIN RESISTANCE AND SCCmec TYPING													
				glycerophosphoryl diester phosphodi-esterase, associated with mecA	Modified penicillin binding protein (PBP2a)	truncated signal transducer protein MecR1	signal transducer protein MecR1	methicillin-resistance regulatory protein	phenol soluble modulin	CsoR-like sulfur transferase-regulated genes B/metallo-beta-lactamase superfamily protein (SCCmec II/III)	homolog of xylose repressor, associated with SCCmec-elements	alternate mec gene	betalactamase from SCCmec XI element	Plasmin-sensitive surface protein	Truncated 3-hydroxy-3-methyl-glutaryl CoA synthase (SCCmec I, Subtyping SCCmec II, IV, V)	CsoR-like sulfur transferase-regulated genes B/ metallo-b-lactamase superfamily protein (SCCmec I, IV a, V und X) (SCCmec I, IV [illegible]	Putative protein (SCCmec I und IV/ACME, WA40)
				ugpQ	mecA	delta_mecR	mecR	mecI	PSMmec	cstB-SCC1 (ex Q2G1R6)	xylR/mecR2	mecC	blaZ (SCCmec XI)	plsSCC (COL)	mvaS-SCC	cstB-scc2, ex Q2G1R6	Q9S0M4
Prediction for CN1, GenBank CP003979.1	*SCCmec IVA (CN1)*	*CC8 (ST72)*	*ST72-MRSA-IVA*	NEG	POS	POS	NEG	NEG	NEG	NEG	NEG	NEG	NEG	NEG	NEG	NEG	NEG
UKD-0900871	SCCmec IVA (CN1)	CC8 (ST72)	ST72-MRSA-IVA	NEG	POS	POS	NEG	NEG	NEG	NEG	NEG		NEG	NEG	NEG	NEG	NEG
Prediction for CMFT1723, GenBank HF569096.1	*SCCmec V (Bengal Bay)*			POS	POS	NEG	NEG	NEG	NEG	NEG	NEG	NEG	NEG	NEG	POS	NEG	NEG
KHDN-10405134	SCCmec V (Bengal Bay)	CC1	CC1-MRSA-V (PVL+)	POS	POS	NEG	NEG	NEG	NEG	NEG	NEG	NEG	NEG	NEG	POS	NEG	NEG
UKD-0932405	SCCmec V (Bengal Bay)	CC1 (ST573/772)	ST772-MRSA-V (PVL+), Bengal Bay Clone	POS	POS	NEG	NEG	NEG	NEG	NEG	NEG		NEG	NEG	POS	NEG	NEG
UKD-1003510	SCCmec V (Bengal Bay)	CC1 (ST573/772)	ST772-MRSA-V (PVL+), Bengal Bay Clone	POS	POS	NEG	NEG	NEG	NEG	NEG	NEG		NEG	NEG	POS	NEG	NEG
UKD-1112420	SCCmec V (Bengal Bay)	CC1 (ST573/772)	ST772-MRSA-V (PVL+), Bengal Bay Clone	POS	POS	NEG	NEG	NEG	NEG	NEG	NEG		NEG	NEG	POS	NEG	NEG
UKD-1275832	SCCmec V (Bengal Bay)	CC1 (ST573/772)	ST772-MRSA-V (PVL+), Bengal Bay Clone	POS	POS	NEG	NEG	NEG	NEG	NEG	NEG		NEG	NEG	POS	NEG	NEG
UKD-13023811	SCCmec V (Bengal Bay)	CC1 (ST573/772)	ST772-MRSA-V (PVL+), Bengal Bay Clone	POS	POS	NEG	NEG	NEG	NEG	NEG	NEG		NEG	NEG	POS	NEG	NEG
UKD-14572163	SCCmec V	CC5	CC5-MRSA-V (sed/j/r+), WA MRSA-11/34/35/90/108	POS	POS	NEG	NEG	NEG	NEG	NEG	NEG	NEG	NEG	NEG	POS	NEG	NEG
KHDN-10421133	SCCmec V+fus	CC97	CC97-MRSA-[V+fus]	POS	POS	NEG	NEG	NEG	NEG	NEG	NEG	NEG	NEG	NEG	POS	NEG	NEG
KHDN-21343541	SCCmec V+fus+tir+ccrAB1	CC1	CC1-MRSA-[V+fus+ccrAB1] (PVL+)	POS	POS	NEG	NEG	NEG	NEG	NEG	NEG	NEG	NEG	NEG	POS	NEG	NEG
KHDN-10416893	SCCmec V+czrC+speG+ccrAB4	CC22	CC22-MRSA-[V+ccrAB4]	POS	POS	NEG	NEG	NEG	NEG	NEG	NEG	NEG	NEG	NEG	POS	NEG	NEG
Prediction for GR1, GenBank AJLX	*SCCmec VT (GR1)*	*CC361*	*CC361-MRSA-V*	POS	POS	NEG	NEG	NEG	NEG	NEG	NEG	NEG	NEG	NEG	POS	NEG	NEG
UKD-0422872	SCCmec VT (GR1)	CC152	CC152-MRSA-V (PVL+)	POS	POS	NEG	NEG	NEG	NEG	NEG	NEG		NEG	NEG	POS	NEG	NEG
Prediction for PM1, GenBank BAFA	*SCCmec VT (PM1)*	*CC59*	*CC59-MRSA-VT (PVL+), Taiwan Clone*	POS	POS	NEG	NEG	NEG	NEG	NEG	NEG	NEG	NEG	NEG	POS	NEG	NEG
KHDN-000871	SCCmec VT (PM1)	CC45 (agr I)	CC45-MRSA-V	POS	POS	NEG	NEG	NEG	NEG	NEG	NEG	NEG	NEG	NEG	POS	NEG	NEG
UKD-0725052	SCCmec VT (PM1)	CC59	CC59-MRSA-VT (PVL+), Taiwan Clone	POS	POS	NEG	NEG	NEG	NEG	NEG	NEG		NEG	NEG	POS	NEG	NEG
UKD-1312138	SCCmec VT (PM1)	CC59	CC59-MRSA-VT (PVL+), Taiwan Clone	POS	POS	NEG	NEG	NEG	NEG	NEG	NEG	NEG	NEG	NEG	POS	NEG	NEG
UKD-Z4728	SCCmec VT (PM1)	CC59	CC59-MRSA-VT (PVL+), Taiwan Clone	POS	POS	NEG	NEG	NEG	NEG	NEG	NEG	NEG	NEG	NEG	POS	NEG	NEG
UKD-K0199	SCCmec VT unknown var.1	CC7	CC7-MRSA-V, WA-MRSA-131	POS	POS	NEG	NEG	NEG	NEG	NEG	NEG	NEG	NEG	NEG	POS	NEG	NEG
UKD-15584619	SCCmec VT unknown var.2	CC30	CC30-MRSA-V/VT	POS	POS	NEG	NEG	NEG	NEG	NEG	NEG	NEG	NEG	NEG	NEG	NEG	NEG
UKD-0712568	SCCmec VT unknown var.3	CC45 (agr I)	CC45-MRSA-V	POS	POS	NEG	NEG	NEG	NEG	NEG	NEG	NEG	NEG	NEG	POS	NEG	POS
KHDN-10420296	SCCmec VT+ACME2	CC45 (agr I)	CC45-MRSA-[V+ACME], WA MRSA-106	POS	POS	NEG	NEG	NEG	NEG	NEG	NEG	NEG	NEG	NEG	POS	NEG	NEG
UKD-15577956	SCCmec VT+Cu/czrC+ccrA1	CC398	CC398-MRSA-[V+ccrA1]	POS	POS	NEG	NEG	NEG	NEG	NEG	NEG	NEG	NEG	NEG	POS	POS	NEG
Prediction for S0385, GenBank AM990992	*SCCmec VT+czrC (SO385)*	*CC398*	*CC398-MRSA-V/VT, Western European/Dutch Livestock-assoc. MRSA*	POS	POS	NEG	NEG	NEG	NEG	NEG	NEG	NEG	NEG	NEG	POS	POS	NEG
UKD-0628560	SCCmec VT+czrC (SO385)	CC8	CC8-MRSA-V	POS	POS	NEG	NEG	NEG	NEG	NEG	NEG		NEG	NEG	POS	POS	NEG
UKD-0510018	SCCmec VT+czrC (SO385)	CC398	CC398-MRSA-V/VT, Western European/Dutch Livestock-assoc. MRSA	POS	POS	NEG	NEG	NEG	NEG	NEG	NEG		NEG	NEG	POS	POS	NEG
UKD-0903558	SCCmec VT+czrC (SO385)	CC398	CC398-MRSA-V/VT, Western European/Dutch Livestock-assoc. MRSA	POS	POS	NEG	NEG	NEG	NEG	NEG	NEG		NEG	NEG	POS	POS	NEG
UKD-0929815	SCCmec VT+czrC (SO385)	CC398	CC398-MRSA-V/VT, Western European/Dutch Livestock-assoc. MRSA	POS	POS	NEG	NEG	NEG	NEG	NEG	NEG		NEG	NEG	POS	POS	NEG
UKD-0907762	SCCmec VT+czrC (SO385)	CC398	CC398-MRSA-V/VT, Western European/Dutch Livestock-assoc. MRSA	POS	POS	NEG	NEG	NEG	NEG	NEG	NEG		NEG	NEG	POS	POS	NEG
UKD-1025105	SCCmec VT+czrC (SO385)	CC398	CC398-MRSA-V/VT, Western European/Dutch Livestock-assoc. MRSA	POS	POS	NEG	NEG	NEG	NEG	NEG	NEG		NEG	NEG	POS	POS	NEG
UKD-11780898	SCCmec VT+czrC (SO385)	CC398	CC398-MRSA-V/VT, Western European/Dutch Livestock-assoc. MRSA	POS	POS	NEG	NEG	NEG	NEG	NEG	NEG		NEG	NEG	POS	POS	NEG
UKD-1377009	SCCmec VT+czrC (SO385)	CC398	CC398-MRSA-V/VT, Western European/Dutch Livestock-assoc. MRSA	POS	POS	NEG	NEG	NEG	NEG	NEG	NEG	NEG	NEG	NEG	POS	POS	NEG
UKD-143406	SCCmec VT+czrC (SO385)	CC398	CC398-MRSA-V/VT, Western European/Dutch Livestock-assoc. MRSA	POS	POS	NEG	NEG	NEG	NEG	NEG	NEG	NEG	NEG	NEG	POS	POS	NEG
UKD-1400363	SCCmec VT+czrC (SO385)	CC398	CC398-MRSA-V/VT, Western European/Dutch Livestock-assoc. MRSA	POS	POS	NEG	NEG	NEG	NEG	NEG	NEG	NEG	NEG	NEG	POS	POS	NEG
UKD-14571852	SCCmec VT+czrC (SO385)	CC398	CC398-MRSA-V/VT, Western European/Dutch Livestock-assoc. MRSA	POS	POS	NEG	NEG	NEG	NEG	NEG	NEG	NEG	NEG	NEG	POS	POS	NEG
UKD-14582574	SCCmec VT+czrC (SO385)	CC398	CC398-MRSA-V/VT, Western European/Dutch Livestock-assoc. MRSA	POS	POS	NEG	NEG	NEG	NEG	NEG	NEG	NEG	NEG	NEG	POS	POS	NEG
UKD-152108	SCCmec VT+czrC (SO385)	CC398	CC398-MRSA-V/VT, Western European/Dutch Livestock-assoc. MRSA	POS	POS	NEG	NEG	NEG	NEG	NEG	NEG	NEG	NEG	NEG	POS	POS	NEG
UKD-15573321	SCCmec VT+czrC (SO385)	CC398	CC398-MRSA-V/VT, Western European/Dutch Livestock-assoc. MRSA	POS	POS	NEG	NEG	NEG	NEG	NEG	NEG	NEG	NEG	NEG	POS	POS	NEG
UKD-15584034	SCCmec VT+czrC , ydhK-neg. variant	CC398	CC398-MRSA-V/VT, Western European/Dutch Livestock-assoc. MRSA	POS	POS	NEG	NEG	NEG	NEG	NEG	NEG	NEG	NEG	NEG	POS	AMB	NEG
UKD-0932581	SCCmecVT+fus+czrC	CC22	CC22-MRSA-[V+fus]	POS	POS	NEG	NEG	NEG	NEG	NEG	NEG	neg	NEG	NEG	POS	POS	NEG
UKD-1121994	SCCmecVT+fus+czrC	CC22	CC22-MRSA-[V+fus]	POS	POS	NEG	NEG	NEG	NEG	NEG	NEG	neg	NEG	NEG	POS	POS	NEG
UKD-1272252	SCCmecVT+fus+czrC	CC22	CC22-MRSA-[V+fus]	POS	POS	NEG	NEG	NEG	NEG	NEG	NEG	neg	NEG	NEG	POS	POS	NEG
Prediction for MRSA18	*SCCmec VI+fus (MRSA18)*			POS	POS	POS	NEG	NEG	NEG	NEG	NEG	NEG	NEG	NEG	POS	NEG	NEG
UKD-1103380	SCCmec VI+fus (MRSA18)	CC7	CC7-MRSA-[VI+fus]	POS	POS	POS	NEG	NEG	NEG	NEG	NEG	neg	NEG	NEG	POS	NEG	NEG
Prediction for M10/0061, GenBank FR823292.1	*SCCmec XI (LGA251/M10-61)*	*CC130*	*CC130-MRSA-XI*	NEG	NEG	NEG	NEG	NEG	NEG	NEG	NEG	POS	POS	NEG	NEG	NEG	NEG
**Reference strain Dublin-DSH_M10 0061**	**SCCmec XI (LGA251/M10-61)**	**CC130**	**CC130-MRSA-XI**	NEG	NEG	NEG	NEG	NEG	NEG	NEG	NEG	POS	POS	NEG	NEG	NEG	NEG
Prediction for LGA251, GenBank FR821779.1	*SCCmec XI (LGA251/M10-61)*	*ST425*	*ST425-MRSA-XI*	NEG	NEG	NEG	NEG	NEG	NEG	NEG	NEG	POS	POS	NEG	NEG	NEG	NEG
UKD-14015835	SCCmec XI (LGA251/M10-61)	CC130	CC130-MRSA-XI	NEG	NEG	NEG	NEG	NEG	NEG	NEG	NEG	POS	POS	NEG	NEG	NEG	NEG
UKD-16573252	SCCmec XI (LGA251/M10-61)	CC130	CC130-MRSA-XI	NEG	NEG	NEG	NEG	NEG	NEG	NEG	NEG	POS	POS	NEG	NEG	NEG	NEG
UKD-0217002	PseudoSCCmec, class B mec complex+Hg	CC8	ST254-MRSA-[PseudoSCCmec class B], Hannover EMRSA (subclone)	POS	POS	POS	NEG	NEG	NEG	NEG	NEG		NEG	NEG	POS	NEG	NEG
UKD-0008510	PseudoSCCmec, class B mec complex+Hg	CC8	ST254-MRSA-[PseudoSCCmec class B], Hannover EMRSA (subclone)	POS	POS	POS	NEG	NEG	NEG	NEG	NEG		NEG	NEG	POS	NEG	NEG
UKD-0110962	PseudoSCCmec, class B mec complex+Hg	CC8	ST254-MRSA-[PseudoSCCmec class B], Hannover EMRSA (subclone)	POS	POS	POS	NEG	NEG	NEG	NEG	NEG		NEG	NEG	POS	NEG	NEG
KHDN-10408593	PseudoSCCmec, class B mec complex+Cu/Hg	CC8	CC8-MRSA-IV (PVL+), ACME-negative/Spanish or Latin American variant of	POS	POS	POS	NEG	NEG	NEG	NEG	NEG	NEG	NEG	NEG	POS	NEG	NEG
UKD-0007201	PseudoSCCmec, class B mec complex	CC5	ST228-MRSA-[pseudoSCCmec class B], South German EMRSA (subclone)	POS	POS	POS	NEG	NEG	NEG	NEG	NEG		NEG	POS	POS	POS	POS
UKD-0033204	PseudoSCCmec, class B mec complex	CC5	ST228-MRSA-[pseudoSCCmec class B], South German EMRSA (subclone)	POS	POS	POS	NEG	NEG	NEG	NEG	NEG		NEG	POS	POS	POS	POS
UKD-0134329	PseudoSCCmec, class B mec complex	CC5	ST228-MRSA-[pseudoSCCmec class B], South German EMRSA (subclone)	POS	AMB	POS	NEG	NEG	NEG	NEG	NEG		NEG	AMB	POS	POS	POS
UKD-0806946	PseudoSCCmec class C+As/Cu	CC398	CC398-MRSA-PseudoSCCmec [class C]	POS	POS	NEG	NEG	NEG	NEG	NEG	NEG		NEG	NEG	POS	NEG	NEG
UKD-0500610	ACME2	CC8	CC8-MSSA-[ACME]	NEG	NEG	NEG	NEG	NEG	NEG	NEG	NEG		NEG	NEG	NEG	NEG	POS
UKD-0610438	ACME2	CC8	CC8-MSSA-[ACME]	NEG	NEG	NEG	NEG	NEG	NEG	NEG	NEG	NEG	NEG	NEG	NEG	NEG	POS
KHDN-10409344	ACME2	CC8	CC8-MSSA-[ACME]	NEG	NEG	NEG	NEG	NEG	NEG	NEG	NEG	NEG	NEG	NEG	NEG	NEG	POS
UKD-0322559	ACME2+speG+czrC+ccrAB4	CC8	CC8-MSSA-[ACME+ccrAB4]	NEG	NEG	NEG	NEG	NEG	NEG	NEG	NEG	NEG	NEG	NEG	NEG	NEG	POS
UKD-0501998	ACME2+speG+czrC+ccrAB4	CC8	CC8-MSSA-[ACME+ccrAB4]	NEG	NEG	NEG	NEG	NEG	NEG	NEG	NEG		NEG	NEG	NEG	NEG	POS
UKD-0505213	ACME2+speG+czrC+ccrAB4	CC8	CC8-MSSA-[ACME+ccrAB4]	NEG	NEG	NEG	NEG	NEG	NEG	NEG	NEG		NEG	NEG	NEG	NEG	NEG
Prediction for MSSA476, GenBank BX571857.1	*fus+tir+ccrAB1 (MSSA476)*	*CC1*	*CC1-MSSA-[fus+ccrAB1]*	NEG	NEG	NEG	NEG	NEG	NEG	NEG	NEG	NEG	NEG	NEG	NEG	NEG	NEG
**Reference strain Sanger 476**	**fus+tir+ccrAB1 (MSSA476)**	**CC1**	**CC1-MSSA-[fus+ccrAB1]**	NEG	NEG	NEG	NEG	NEG	NEG	NEG	NEG	NEG	NEG	NEG	NEG	NEG	NEG
UKD-14023914	fus+tir+ccrAB1 (MSSA476)	CC1	CC1-MSSA-[fus+ccrAB1]	NEG	NEG	NEG	NEG	NEG	NEG	NEG	NEG	NEG	NEG	NEG	NEG	NEG	NEG
UKD-K00034	kdp+ccrAB2	CC182	CC182-MSSA-[kdp+ccrAB2]	NEG	NEG	NEG	NEG	NEG	NEG	NEG	NEG	NEG	NEG	NEG	NEG	NEG	NEG
UKD-15580369	As+ccrAB4	CC22	CC22-MSSA-[ccrAB4]	NEG	NEG	NEG	NEG	NEG	NEG	NEG	NEG	neg	NEG	NEG	POS	NEG	NEG
UKD-15570100	ACME3+ccrAB1 (Strain 21342)	CC80	atypical CC80-MSSA-[ccrA1+ACME III] (ORF CM14+, PVL+)	NEG	NEG	NEG	NEG	NEG	NEG	NEG	NEG	NEG	NEG	NEG	NEG	NEG	NEG
UKD-1010678	speG+czrC+ccrAB4	CC8	CC8-MSSA-[ccrAB4]	NEG	NEG	NEG	NEG	NEG	NEG	NEG	NEG	NEG	NEG	NEG	NEG	NEG	NEG
UKD-1008243	speG+czrC+ccrAB4	CC8	CC8-MSSA-[ccrAB4]	NEG	NEG	NEG	NEG	NEG	NEG	NEG	NEG	NEG	NEG	NEG	NEG	NEG	NEG
UKD-1009430	speG+czrC+ccrAB4	CC8	CC8-MSSA-[ccrAB4]	NEG	NEG	NEG	NEG	NEG	NEG	NEG	NEG	NEG	NEG	NEG	NEG	NEG	NEG
UKD-12071828	speG+czrC+ccrAB4	CC22	CC22-MSSA-[ccrAB4]	NEG	NEG	NEG	NEG	NEG	NEG	NEG	NEG		NEG	NEG	NEG	NEG	NEG
UKD-14011883	speG+czrC+ccrAB4	CC22	CC22-MSSA-[ccrAB4]	NEG	NEG	NEG	NEG	NEG	NEG	NEG	NEG	NEG	NEG	NEG	NEG	NEG	NEG
UKD-15008293	speG+czrC+ccrAB4	CC22	CC22-MSSA-[ccrAB4]	NEG	NEG	NEG	NEG	NEG	NEG	NEG	NEG	NEG	NEG	NEG	NEG	NEG	NEG
UKD-1514877	speG+czrC+ccrAB4	CC22	CC22-MSSA-[ccrAB4]	NEG	NEG	NEG	NEG	NEG	NEG	NEG	NEG	NEG	NEG	NEG	NEG	NEG	NEG
UKD-15580971	speG+czrC+ccrAB4	CC22	CC22-MSSA-[ccrAB4]	NEG	NEG	NEG	NEG	NEG	NEG	NEG	NEG	NEG	NEG	NEG	NEG	NEG	NEG
UKD-15582646	speG+czrC+ccrAB4	CC22	CC22-MSSA-[ccrAB4]	NEG	NEG	NEG	NEG	NEG	NEG	NEG	NEG	NEG	NEG	NEG	NEG	NEG	NEG

Isolate ID	SCCmec type	Clonal complex	METHICILLIN RESISTANCE AND SCCmec TYPING																			
			Potassium-translocating ATPase operon					LytTR domain DNA-binding regulator Q93IB7 (subtyping SCCmec III and IV)	Putative protein D1GU38 (Subtyping SCCmec III, identi-fication of VT,ZH47,VII)	Putative protein D1GU38-TW20 (Subtyping SCCmec III, identi-fication of VII)	Putative ADP-ribosyltransferaseQ933A2	Putative protein D1GU55 (SCCmec III/VII)	Abortive phage resistance protein B2Y834 (SCCmecIV A,G,c and MRSAZH47)	Putative protein B6VQU0 (SCCmec IV h,j)	Q3YK51 (SCCmec IVg)	Putative lipoprotein	Putative protein (Subtyping SCCmec V)	Putative protein located within SCCmec type V/SCCfus elements			Putative protein (SCCmec XI)	Putative protein (composite SCCs)
			kdpA-SCC	kdpB-SCC	kdpC-SCC	kdpD-SCC	kdpE-SCC	Q93IB7	D1GU38	D1GU38-TW20	Q933A2 (SCCmec III, IX)	D1GU55	B2Y834	B6VQU0	Q3YK51	ydhK (FPR3757)	DUF1958	Q4LAG7 (SCC consensus)	Q4LAG7 (SCCmecV, SO385)	Q4LAG7 (SCCfus 45394F/MSSA476)	C5QAP8-M10-61	Q8CU82
VIRTUAL HYBRIDISATION for COL, GenBank CP000046	SCCmec I (COL)	CC8	NEG	NEG	NEG	NEG	NEG	NEG	NEG	NEG	NEG	NEG	NEG	NEG	NEG	NEG	NEG	NEG	NEG	NEG	NEG	NEG
Reference strain COL	SCCmec I (COL)	CC8	NEG	NEG	NEG	NEG	NEG	NEG	NEG	NEG	NEG	NEG	NEG	NEG	NEG	NEG	NEG	NEG	NEG	NEG	NEG	NEG
UKD-0007063	SCCmec I (COL)	CC5	NEG	NEG	NEG	NEG	NEG	NEG	NEG	NEG	NEG	NEG	NEG	NEG	NEG	NEG	NEG	NEG	NEG	NEG	NEG	NEG
UKD-0220759	SCCmec I (COL)	CC5	NEG	NEG	NEG	NEG	NEG	NEG	NEG	NEG	NEG	NEG	NEG	NEG	NEG	NEG	NEG	NEG	NEG	NEG	NEG	NEG
UKD-0308986	SCCmec I (COL)	CC5	NEG	NEG	NEG	NEG	NEG	NEG	NEG	NEG	NEG	NEG	NEG	NEG	NEG	NEG	NEG	NEG	NEG	NEG	NEG	NEG
UKD-0500009	SCCmec I (COL)	CC5	NEG	NEG	NEG	NEG	NEG	NEG	NEG	NEG	NEG	NEG	NEG	NEG	NEG	NEG	NEG	NEG	NEG	NEG	NEG	NEG
UKD-0508483	SCCmec I (COL)	CC5	NEG	NEG	NEG	NEG	NEG	NEG	NEG	NEG	NEG	NEG	NEG	NEG	NEG	NEG	NEG	NEG	NEG	NEG	NEG	NEG
UKD-0519001	SCCmec I (COL)	CC5	NEG	NEG	NEG	NEG	NEG	NEG	NEG	NEG	NEG	NEG	NEG	NEG	NEG	NEG	NEG	NEG	NEG	NEG	NEG	NEG
UKD-0701876	SCCmec I (COL)	CC5	NEG	NEG	NEG	NEG	NEG	NEG	NEG	NEG	NEG	NEG	NEG	NEG	NEG	NEG	NEG	NEG	NEG	NEG	NEG	NEG
UKD-0708865	SCCmec I (COL)	CC5	NEG	NEG	NEG	NEG	NEG	NEG	NEG	NEG	NEG	NEG	NEG	NEG	NEG	NEG	NEG	NEG	NEG	NEG	NEG	NEG
UKD-1001782	SCCmec I (COL)	CC5	NEG	NEG	NEG	NEG	NEG	NEG	NEG	NEG	NEG	NEG	NEG	NEG	NEG	NEG	NEG	NEG	NEG	NEG	NEG	NEG
Prediction for PSP1996, GenBank ANHU	SCCmec I (PSP1996)	CC8	NEG	NEG	NEG	NEG	NEG	NEG	NEG	NEG	NEG	NEG	NEG	NEG	NEG	NEG	NEG	NEG	NEG	NEG	NEG	NEG
UKD-0261727	SCCmec I (PSP1996)	CC8	NEG	NEG	NEG	NEG	NEG	NEG	NEG	NEG	NEG	NEG	NEG	NEG	NEG	NEG	NEG	NEG	NEG	NEG	NEG	NEG
VIRTUAL HYBRIDISATION for MRSA7 : VIRTUAL HYBRII	SCCmec I+fus+tirS (Geraldine Clone)	CC5	NEG	NEG	NEG	NEG	NEG	NEG	NEG	NEG	NEG	NEG	NEG	NEG	NEG	NEG	NEG	NEG	NEG	NEG	NEG	NEG
UKD-1221930	SCCmec I+fus+tirS (Geraldine Clone)	CC5	NEG	NEG	NEG	NEG	NEG	NEG	NEG	NEG	NEG	NEG	NEG	NEG	NEG	NEG	NEG	NEG	NEG	NEG	NEG	NEG
Prediction for N315, GenBank BA000018.3	SCCmec II (N315)	CC5	POS	POS	POS	POS	POS	NEG	NEG	NEG	NEG	NEG	NEG	NEG	NEG	NEG	NEG	NEG	NEG	NEG	NEG	NEG
Reference strain N315	SCCmec II (N315)	CC5	POS	POS	POS	POS	POS	NEG	NEG	NEG	NEG	NEG	NEG	NEG	NEG	NEG	NEG	NEG	NEG	NEG	NEG	NEG
Prediction for MRSA252, GenBank BX571856.1	SCCmec II (N315)	CC30	POS	POS	POS	POS	POS	NEG	NEG	NEG	NEG	NEG	NEG	NEG	NEG	NEG	NEG	NEG	NEG	NEG	NEG	NEG
Reference strain Sanger 252	SCCmec II (N315)	CC30	POS	POS	POS	POS	POS	NEG	NEG	NEG	NEG	NEG	NEG	NEG	NEG	NEG	NEG	NEG	NEG	NEG	NEG	NEG
UKD-0230792	SCCmec II (N315)	CC30	POS	POS	POS	POS	POS	NEG	NEG	NEG	NEG	NEG	NEG	NEG	NEG	NEG	NEG	NEG	NEG	NEG	NEG	NEG
Prediction for Mu50, GenBank BA000017.4	SCCmec II (N315)	CC5	POS	POS	POS	POS	POS	NEG	NEG	NEG	NEG	NEG	NEG	NEG	NEG	NEG	NEG	NEG	NEG	NEG	NEG	NEG
Reference strain MU50	SCCmec II (N315)	CC5	POS	POS	POS	POS	POS	NEG	NEG	NEG	NEG	NEG	NEG	NEG	NEG	NEG	NEG	NEG	NEG	NEG	NEG	NEG
Prediction for JH1, GenBank CP000736.1	SCCmec II (JH1/JH9)	CC5	POS	POS	POS	POS	POS	NEG	NEG	NEG	NEG	NEG	NEG	NEG	NEG	NEG	NEG	NEG	NEG	NEG	NEG	NEG
Prediction for JH9, GenBank CP000703.1	SCCmec II (JH1/JH9)	CC5	POS	POS	POS	POS	POS	NEG	NEG	NEG	NEG	NEG	NEG	NEG	NEG	NEG	NEG	NEG	NEG	NEG	NEG	NEG
Reference strain Dublin-DSH_AR07.4_0237	SCCmec II (JH1/JH9)		POS	POS	POS	POS	POS	NEG	NEG	NEG	NEG	NEG	NEG	NEG	NEG	NEG	NEG	NEG	NEG	NEG	NEG	NEG
Reference strain NARSA_382 / USA100	SCCmec II (JH1/JH9)	CC5	POS	POS	POS	POS	POS	NEG	NEG	NEG	NEG	NEG	NEG	NEG	NEG	NEG	NEG	NEG	NEG	NEG	NEG	NEG
UKD-0046921	SCCmec II (JH1/JH9)	CC5	POS	POS	POS	POS	POS	NEG	NEG	NEG	NEG	NEG	NEG	NEG	NEG	NEG	NEG	NEG	NEG	NEG	NEG	NEG
UKD-0333415	SCCmec II (JH1/JH9)	CC5	POS	POS	POS	POS	POS	NEG	NEG	NEG	NEG	NEG	NEG	NEG	NEG	NEG	NEG	NEG	NEG	NEG	NEG	NEG
UKD-0341645	SCCmec II (JH1/JH9)	CC5	POS	POS	POS	POS	POS	NEG	NEG	NEG	NEG	NEG	NEG	NEG	NEG	NEG	NEG	NEG	NEG	NEG	NEG	NEG
UKD-0504626	SCCmec II (JH1/JH9)	CC5	POS	POS	POS	POS	POS	NEG	NEG	NEG	NEG	NEG	NEG	NEG	NEG	NEG	NEG	NEG	NEG	NEG	NEG	NEG
UKD-0507581	SCCmec II (JH1/JH9)	CC5	POS	POS	POS	POS	POS	NEG	NEG	NEG	NEG	NEG	NEG	NEG	NEG	NEG	NEG	NEG	NEG	NEG	NEG	NEG
UKD-0507903	SCCmec II (JH1/JH9)	CC5	POS	POS	POS	POS	POS	NEG	NEG	NEG	NEG	NEG	NEG	NEG	NEG	NEG	NEG	NEG	NEG	NEG	NEG	NEG
UKD-0700817	SCCmec II (JH1/JH9)	CC5	POS	POS	POS	POS	AMB	NEG	NEG	NEG	NEG	NEG	NEG	NEG	NEG	NEG	NEG	NEG	NEG	NEG	NEG	NEG
UKD-1014561	SCCmec II (JH1/JH9)	CC5	POS	POS	POS	POS	POS	NEG	NEG	NEG	NEG	NEG	NEG	NEG	NEG	NEG	NEG	NEG	NEG	NEG	NEG	NEG
UKD-1037507	SCCmec II (JH1/JH9)	CC5	POS	POS	POS	POS	POS	NEG	NEG	NEG	NEG	NEG	NEG	NEG	NEG	NEG	NEG	NEG	NEG	NEG	NEG	NEG
UKD-1038321	SCCmec II (JH1/JH9)	CC5	POS	POS	POS	POS	POS	NEG	NEG	NEG	NEG	NEG	NEG	NEG	NEG	NEG	NEG	NEG	NEG	NEG	NEG	NEG
UKD-1111608	SCCmec II (JH1/JH9)	CC5	POS	POS	POS	POS	POS	NEG	NEG	NEG	NEG	NEG	NEG	NEG	NEG	NEG	NEG	NEG	NEG	NEG	NEG	NEG
UKD-12753135	SCCmec II (JH1/JH9)	CC5	POS	POS	POS	POS	POS	NEG	NEG	NEG	NEG	NEG	NEG	NEG	NEG	NEG	NEG	NEG	NEG	NEG	NEG	NEG
UKD-1312816	SCCmec II (JH1/JH9)	CC5	POS	POS	POS	POS	POS	NEG	NEG	NEG	NEG	NEG	NEG	NEG	NEG	NEG	NEG	NEG	NEG	NEG	NEG	NEG
UKD-1401623	SCCmec II (JH1/JH9)	CC5	POS	POS	POS	POS	POS	NEG	NEG	NEG	NEG	NEG	NEG	NEG	NEG	NEG	NEG	NEG	NEG	NEG	NEG	NEG
UKD-15571943	SCCmec II+czrC+ccrA/B-4	CC5	POS	POS	POS	POS	POS	NEG	NEG	NEG	NEG	NEG	NEG	NEG	NEG	NEG	NEG	NEG	NEG	NEG	NEG	NEG
UKD-15576888	SCCmec II+czrC+ccrA/B-4	CC5	POS	POS	POS	POS	POS	NEG	NEG	NEG	NEG	NEG	NEG	NEG	NEG	NEG	NEG	NEG	NEG	NEG	NEG	NEG
UKD-14576461	SCCmec II+speG+czrC+ccrA/B-4	CC5	POS	POS	POS	POS	POS	NEG	NEG	NEG	NEG	NEG	NEG	NEG	NEG	NEG	NEG	NEG	NEG	NEG	NEG	NEG
UKD-15581649	SCCmec II+speG+czrC+ccrA/B-4	CC5	POS	POS	POS	POS	POS	NEG	NEG	NEG	NEG	NEG	NEG	NEG	NEG	NEG	NEG	NEG	NEG	NEG	NEG	NEG
Prediction for CIGC345D, GenBank AHVO	SCCmec II A/B/D	CC8	NEG	NEG	NEG	NEG	NEG	NEG	NEG	NEG	NEG	NEG	NEG	NEG	NEG	NEG	NEG	NEG	NEG	NEG	NEG	NEG
UKD-12K00171	SCCmec II A/B/D	CC8	NEG	NEG	NEG	NEG	NEG	NEG	NEG	NEG	NEG	NEG	NEG	NEG	NEG	NEG	NEG	NEG	NEG	NEG	NEG	NEG
Prediction for JKD6008, GenBank CP002120.1	SCC [mec III+Cd] (JKD6008)	CC8 (ST239)	NEG	NEG	NEG	NEG	NEG	NEG	NEG	NEG	POS	NEG	NEG	NEG	NEG	NEG	NEG	NEG	NEG	NEG	NEG	NEG
Prediction for JKD6009, GenBank ABSA	SCC [mec III+Cd] (JKD6008)	CC8 (ST239)	NEG	NEG	NEG	NEG	NEG	NEG	NEG	NEG	POS	NEG	NEG	NEG	NEG	NEG	NEG	NEG	NEG	NEG	NEG	NEG
Reference strain ATCC_33592	SCC [mec III+Cd] (ATCC33592)	CC8 (ST239)	NEG	NEG	NEG	NEG	NEG	POS	NEG	NEG	POS	NEG	NEG	NEG	NEG	NEG	NEG	NEG	NEG	NEG	NEG	NEG
Prediction for PPUKM-775-2009, GenBank AMRE	SCC [mec III+Cd+ccrC] (PPUKM-775-2009)	CC8 (ST239)	NEG	NEG	NEG	NEG	NEG	NEG	POS	POS	POS	NEG	NEG	NEG	NEG	NEG	NEG	NEG	NEG	NEG	NEG	NEG
Prediction for T0131, GenBank CP002643.1	SCC [mec III+Cd+ccrC] (T0131)	CC8 (ST239)	NEG	NEG	NEG	NEG	NEG	NEG	POS	POS	POS	NEG	NEG	NEG	NEG	NEG	NEG	NEG	NEG	NEG	NEG	NEG
Prediction for XN108, GenBank CP007447.1	SCC [mec III+Cd+ccrC] (XN108)	CC8 (ST239)	NEG	NEG	NEG	NEG	NEG	NEG	POS	POS	POS	NEG	NEG	NEG	NEG	NEG	NEG	NEG	NEG	NEG	NEG	NEG
Prediction for CN79, GenBank ANCJ	SCC [mec III+Cd+ccrC] (CN79)	CC8 (ST239)	NEG	NEG	NEG	NEG	NEG	NEG	POS	POS	POS	POS	NEG	NEG	NEG	NEG	NEG	NEG	NEG	NEG	NEG	NEG
Prediction for 16K, GenBank BABZ	SCC [mec III+Cd+ccrC] (CN79)	CC8 (ST239)	NEG	NEG	NEG	NEG	NEG	NEG	POS	POS	POS	POS	NEG	NEG	NEG	NEG	NEG	NEG	NEG	NEG	NEG	NEG
UKD-0835987	SCC [mec III+Cd+ccrC] (CN79)	CC8 (ST239)	NEG	NEG	NEG	NEG	NEG	NEG	POS	POS	POS	POS	NEG	NEG	NEG	NEG	NEG	NEG	NEG	NEG	NEG	NEG
UKD-16581769	SCCmec III+Cd+ccrC	CC8 (ST239)	NEG	NEG	NEG	NEG	NEG	NEG	POS	POS	POS	POS	NEG	NEG	NEG	NEG	NEG	POS	POS	NEG	NEG	NEG
Prediction for Bmb9393, GenBank CP005288.1	SCCmec III+Cd/Hg+ccrC (Bmb9393)	CC8 (ST239)	NEG	NEG	NEG	NEG	NEG	NEG	POS	POS	POS	NEG	NEG	NEG	NEG	NEG	NEG	NEG	NEG	NEG	NEG	NEG
UKD-15574263	SCCmec III+Cd/Hg+ccrC (Bmb9393)	CC8 (ST239)	NEG	NEG	NEG	NEG	NEG	NEG	POS	POS	POS	NEG	NEG	NEG	NEG	NEG	NEG	NEG	NEG	NEG	NEG	NEG
Prediction for SK1585, GenBank AYLT	SCCmec III+ccrC+Cd/Hg (SK1585)	CC8 (ST239)	NEG	NEG	NEG	NEG	NEG	POS	POS	POS	POS	POS	NEG	NEG	NEG	NEG	NEG	NEG	NEG	NEG	NEG	NEG
UKD-105861	SCCmec III+ccrC+Cd/Hg (SK1585)	CC8 (ST239)	NEG	NEG	NEG	NEG	NEG	POS	POS	POS	POS	POS	NEG	NEG	NEG	NEG	NEG	NEG	NEG	NEG	NEG	NEG
UKD-134572	SCCmec III+ccrC+Cd/Hg (SK1585)	CC8 (ST239)	NEG	NEG	NEG	NEG	NEG	POS	POS	POS	POS	POS	NEG	NEG	NEG	NEG	NEG	NEG	NEG	NEG	NEG	NEG
UKD-139143	SCCmec III+ccrC+Cd/Hg (SK1585)	CC8 (ST239)	NEG	NEG	NEG	NEG	NEG	POS	POS	POS	POS	POS	NEG	NEG	NEG	NEG	NEG	NEG	NEG	NEG	NEG	NEG
Prediction for TW20, GenBank FN433596.1	SCC [mec III+Cd/Hg+ccrC] (TW20)	CC8 (ST239)	NEG	NEG	NEG	NEG	NEG	POS	POS	POS	POS	NEG	NEG	NEG	NEG	NEG	NEG	NEG	NEG	NEG	NEG	NEG
Prediction for ATCC BAA-39, GenBank AEEK	SCC [mec III+Cd/Hg+ccrC] (TW20)	CC8 (ST239)	NEG	NEG	NEG	NEG	NEG	POS	POS	POS	POS	NEG	NEG	NEG	NEG	NEG	NEG	NEG	NEG	NEG	NEG	NEG

Isolate ID	SCCmec type	Clonal complex	METHICILLIN RESISTANCE AND SCCmec TYPING																			
			Potassium-translocating ATPase operon					LytTR domain DNA-binding regulator Q93IB7 (subtyping SCCmec III and IV)	Putative protein D1GU38 (Subtyping SCCmec III, identi-fication of VT,ZH47,VII)	Putative protein D1GU38-TW20 (Subtyping SCCmec III, identi-fication of VII)	Putative ADP-ribosyltransferaseQ933A2	Putative protein D1GU55 (SCCmec III/VII)	Abortive phage resistance protein B2Y834 (SCCmecIV A,G,c and MRSAZH47)	Putative protein B6VQU0 (SCCmec IV h,j)	Q3YK51 (SCCmec IVg)	Putative lipoprotein	Putative protein (Subtyping SCCmec V)	Putative protein located within SCCmec type V/SCCfus elements			Putative protein (SCCmec XI)	Putative protein (composite SCCs)
			kdpA-SCC	kdpB-SCC	kdpC-SCC	kdpD-SCC	kdpE-SCC	Q93IB7	D1GU38	D1GU38-TW20	Q933A2 (SCCmec III, IX)	D1GU55	B2Y834	B6VQU0	Q3YK51	ydhK (FPR3757)	DUF1958	Q4LAG7 (SCC consensus)	Q4LAG7 (SCCmecV, SO385)	Q4LAG7 (SCCfus 45394F/MSSA476)	C5QAP8-M10-61	Q8CU82
Prediction for MW2, GenBank BA000033.2	*SCCmec IVa (MW2)*	*CC1*	*NEG*	*NEG*	*NEG*	*NEG*	*NEG*	*NEG*	*NEG*	*NEG*	*NEG*	*NEG*	*NEG*	*NEG*	*NEG*	*NEG*	*NEG*	*NEG*	*NEG*	*NEG*	*NEG*	*NEG*
Reference strain MW2	SCCmec IVa (MW2)	CC1	NEG	NEG	NEG	NEG	NEG	NEG	NEG	NEG	NEG	NEG	NEG	NEG	NEG	NEG	NEG	NEG	NEG	NEG	NEG	NEG
Reference strain CA05 (JCSC1968)	SCCmec IVa (MW2)	CC45	NEG	NEG	NEG	NEG	NEG	NEG	NEG	NEG	NEG	NEG	NEG	NEG	NEG	NEG	NEG	NEG	NEG	NEG	NEG	NEG
UKD-0127106	SCCmec IVa (MW2)	CC1	NEG	NEG	NEG	NEG	NEG	NEG	NEG	NEG	AMB	NEG	NEG	NEG	NEG	NEG	NEG	NEG	NEG	NEG	NEG	NEG
UKD-0701446	SCCmec IVa (MW2)	CC1	NEG	NEG	NEG	NEG	NEG	NEG	NEG	NEG	NEG	NEG	NEG	NEG	NEG	NEG	NEG	NEG	NEG	NEG	NEG	NEG
UKD-0947486	SCCmec IVa (MW2)	CC1	NEG	NEG	NEG	NEG	NEG	NEG	NEG	NEG	NEG	NEG	NEG	NEG	NEG	NEG	NEG	NEG	NEG	NEG	NEG	NEG
UKD-1031910	SCCmec IVa (MW2)	CC1	NEG	NEG	NEG	NEG	NEG	NEG	NEG	NEG	NEG	NEG	NEG	NEG	NEG	NEG	NEG	NEG	NEG	NEG	NEG	NEG
UKD-14571328	SCCmec IVa (MW2)	CC1	NEG	NEG	NEG	NEG	NEG	NEG	NEG	NEG	NEG	NEG	NEG	NEG	NEG	NEG	NEG	NEG	NEG	NEG	NEG	NEG
UKD-03003012	SCCmec IVa (MW2)	CC5	NEG	NEG	NEG	NEG	NEG	NEG	NEG	NEG	AMB	NEG	NEG	NEG	NEG	NEG	NEG	NEG	NEG	NEG	NEG	NEG
UKD-15582582	SCCmec IVa (MW2)	CC6	NEG	NEG	NEG	NEG	NEG	NEG	NEG	NEG	NEG	NEG	NEG	NEG	NEG	NEG	NEG	NEG	NEG	NEG	NEG	NEG
UKD-16574230	SCCmec IVa (MW2)	CC6	NEG	NEG	NEG	NEG	NEG	NEG	NEG	NEG	NEG	NEG	NEG	NEG	NEG	NEG	NEG	NEG	NEG	NEG	NEG	NEG
UKD-16582560	SCCmec IVa (MW2)	CC6	NEG	NEG	NEG	NEG	NEG	NEG	NEG	NEG	NEG	NEG	NEG	NEG	NEG	NEG	NEG	NEG	NEG	NEG	NEG	NEG
UKD-0802855	SCCmec IVa (MW2)	CC7	NEG	NEG	NEG	NEG	NEG	NEG	NEG	NEG	NEG	NEG	NEG	NEG	NEG	NEG	NEG	NEG	NEG	NEG	NEG	NEG
KHDN-10405258	SCCmec IVa (MW2)	CC8	NEG	NEG	NEG	NEG	NEG	NEG	NEG	NEG	NEG	NEG	NEG	NEG	NEG	NEG	NEG	NEG	NEG	NEG	NEG	NEG
UKD-15573497	SCCmec IVa (MW2)	CC22	NEG	NEG	NEG	NEG	NEG	NEG	NEG	NEG	NEG	NEG	NEG	NEG	NEG	NEG	NEG	NEG	NEG	NEG	NEG	NEG
UKD-0634729	SCCmec IVa (MW2)	CC30	NEG	NEG	NEG	NEG	NEG	NEG	NEG	NEG	NEG	NEG	NEG	NEG	NEG	NEG	NEG	NEG	NEG	NEG	NEG	NEG
UKD-1119771	SCCmec IVa (MW2)	CC30	NEG	NEG	NEG	NEG	NEG	NEG	NEG	NEG	NEG	NEG	NEG	NEG	NEG	NEG	NEG	NEG	NEG	NEG	NEG	NEG
KHDN-10411458	SCCmec IVa (MW2)	CC30	NEG	NEG	NEG	NEG	NEG	NEG	NEG	NEG	NEG	NEG	NEG	NEG	NEG	NEG	NEG	NEG	NEG	NEG	NEG	NEG
KHDN-398609	SCCmec IVa (MW2)	CC30	NEG	NEG	NEG	NEG	NEG	NEG	NEG	NEG	NEG	NEG	NEG	NEG	NEG	NEG	NEG	NEG	NEG	NEG	NEG	NEG
KHDN-01081998	SCCmec IVa (MW2)	CC30	NEG	NEG	NEG	NEG	NEG	NEG	NEG	NEG	NEG	NEG	NEG	NEG	NEG	NEG	NEG	NEG	NEG	NEG	NEG	NEG
KHDN-10399223	SCCmec IVa (MW2)	CC30	NEG	NEG	NEG	NEG	NEG	NEG	NEG	NEG	NEG	NEG	NEG	NEG	NEG	NEG	NEG	NEG	NEG	NEG	NEG	NEG
KHDN-W261158	SCCmec IVa (MW2)	CC30	NEG	NEG	NEG	NEG	NEG	NEG	NEG	NEG	NEG	NEG	NEG	NEG	NEG	NEG	NEG	NEG	NEG	NEG	NEG	NEG
UKD-0002046	SCCmec IVa (MW2)	CC45 [agr I]	NEG	NEG	NEG	NEG	NEG	NEG	NEG	NEG	NEG	NEG	NEG	NEG	NEG	NEG	NEG	NEG	NEG	NEG	NEG	NEG
UKD-0029618	SCCmec IVa (MW2)	CC45 [agr I]	NEG	NEG	NEG	NEG	NEG	NEG	NEG	NEG	NEG	NEG	NEG	NEG	NEG	NEG	NEG	NEG	NEG	NEG	NEG	NEG
UKD-0043085	SCCmec IVa (MW2)	CC45 [agr I]	NEG	NEG	NEG	NEG	NEG	NEG	NEG	NEG	NEG	NEG	NEG	NEG	NEG	NEG	NEG	NEG	NEG	NEG	NEG	NEG
UKD-0047053	SCCmec IVa (MW2)	CC45 [agr I]	NEG	NEG	NEG	NEG	NEG	NEG	NEG	NEG	NEG	NEG	NEG	NEG	NEG	NEG	NEG	NEG	NEG	NEG	NEG	NEG
UKD-0047429	SCCmec IVa (MW2)	CC45 [agr I]	NEG	NEG	NEG	NEG	NEG	NEG	NEG	NEG	NEG	NEG	NEG	NEG	NEG	NEG	NEG	NEG	NEG	NEG	NEG	NEG
UKD-0112465	SCCmec IVa (MW2)	CC45 [agr I]	NEG	NEG	NEG	NEG	NEG	NEG	NEG	NEG	NEG	NEG	NEG	NEG	NEG	NEG	NEG	NEG	NEG	NEG	NEG	NEG
UKD-0103483	SCCmec IVa (MW2)	CC45 [agr I]	NEG	NEG	NEG	NEG	NEG	NEG	NEG	NEG	NEG	NEG	NEG	NEG	NEG	NEG	NEG	NEG	NEG	NEG	NEG	NEG
UKD-0305014	SCCmec IVa (MW2)	CC45 [agr I]	NEG	NEG	NEG	NEG	NEG	NEG	NEG	NEG	NEG	NEG	NEG	NEG	NEG	NEG	NEG	NEG	NEG	NEG	NEG	NEG
UKD-0401119	SCCmec IVa (MW2)	CC45 [agr I]	NEG	NEG	NEG	NEG	NEG	NEG	NEG	NEG	NEG	NEG	NEG	NEG	NEG	NEG	NEG	NEG	NEG	NEG	NEG	NEG
UKD-0537969	SCCmec IVa (MW2)	CC45 [agr I]	NEG	NEG	NEG	NEG	NEG	NEG	NEG	NEG	NEG	NEG	NEG	NEG	NEG	NEG	NEG	NEG	NEG	NEG	NEG	NEG
UKD-0703373	SCCmec IVa (MW2)	CC45 [agr I]	NEG	NEG	NEG	NEG	NEG	NEG	NEG	NEG	NEG	NEG	NEG	NEG	NEG	NEG	NEG	NEG	NEG	NEG	NEG	NEG
UKD-0813653	SCCmec IVa (MW2)	CC45 [agr I]	NEG	NEG	NEG	NEG	NEG	NEG	NEG	NEG	NEG	NEG	NEG	NEG	NEG	NEG	NEG	NEG	NEG	NEG	NEG	NEG
UKD-0839175	SCCmec IVa (MW2)	CC45 [agr I]	NEG	NEG	NEG	NEG	NEG	NEG	NEG	NEG	NEG	NEG	NEG	NEG	NEG	NEG	NEG	NEG	NEG	NEG	NEG	NEG
UKD-1108422	SCCmec IVa (MW2)	CC45 [agr I]	NEG	NEG	NEG	NEG	NEG	NEG	NEG	NEG	NEG	NEG	NEG	NEG	NEG	NEG	NEG	NEG	NEG	NEG	NEG	NEG
UKD-1380645	SCCmec IVa (MW2)	CC45 [agr I]	NEG	NEG	NEG	NEG	NEG	NEG	NEG	NEG	NEG	NEG	NEG	NEG	NEG	NEG	NEG	NEG	NEG	NEG	NEG	NEG
UKD-148016	SCCmec IVa (MW2)	CC45 [agr I]	NEG	NEG	NEG	NEG	NEG	NEG	NEG	NEG	NEG	NEG	NEG	NEG	NEG	NEG	NEG	NEG	NEG	NEG	NEG	NEG
UKD-16571773	SCCmec IVa (MW2)	CC45 [agr I]	NEG	NEG	NEG	NEG	NEG	NEG	NEG	NEG	NEG	NEG	NEG	NEG	NEG	NEG	NEG	NEG	NEG	NEG	NEG	NEG
KHDN-10407738	SCCmec IVa (MW2)	CC45 [ST617]	NEG	NEG	NEG	NEG	NEG	NEG	NEG	NEG	NEG	NEG	NEG	NEG	NEG	NEG	NEG	NEG	NEG	NEG	NEG	NEG
UKD-0853670	SCCmec IVa (MW2)	CC88	NEG	NEG	NEG	NEG	NEG	NEG	NEG	NEG	NEG	NEG	NEG	NEG	NEG	NEG	NEG	NEG	NEG	NEG	NEG	NEG
UKD-1417352	SCCmec IVa (MW2)	CC88	NEG	NEG	NEG	NEG	NEG	NEG	NEG	NEG	NEG	NEG	NEG	NEG	NEG	NEG	NEG	NEG	NEG	NEG	NEG	NEG
UKD-15571283	SCCmec IVa (MW2)	CC88	NEG	NEG	NEG	NEG	NEG	NEG	NEG	NEG	NEG	NEG	NEG	NEG	NEG	NEG	NEG	NEG	NEG	NEG	NEG	NEG
KHDN-10406719	SCCmec IVa (MW2)	ST93	NEG	NEG	NEG	NEG	NEG	NEG	NEG	NEG	NEG	NEG	NEG	NEG	NEG	NEG	NEG	NEG	NEG	NEG	NEG	NEG
KHDN-0060294	SCCmec IVa (MW2)	ST93	NEG	NEG	NEG	NEG	NEG	NEG	NEG	NEG	NEG	NEG	NEG	NEG	NEG	NEG	NEG	NEG	NEG	NEG	NEG	NEG
UKD-0734190	SCCmec IVa (MW2)	CC97	NEG	NEG	NEG	NEG	NEG	NEG	NEG	NEG	NEG	NEG	NEG	NEG	NEG	NEG	NEG	NEG	NEG	NEG	NEG	NEG
UKD-13006983	SCCmec IVa (MW2)	CC97	NEG	NEG	NEG	NEG	NEG	NEG	NEG	NEG	NEG	NEG	NEG	NEG	NEG	NEG	NEG	NEG	NEG	NEG	NEG	NEG
Prediction for H131520133	*SCCmec IVa (H131520133)*	*CC152*	*NEG*	*NEG*	*NEG*	*NEG*	*NEG*	*NEG*	*NEG*	*NEG*	*NEG*	*NEG*	*NEG*	*NEG*	*NEG*	*NEG*	*NEG*	*NEG*	*NEG*	*NEG*	*NEG*	*NEG*
KHDN-325892	SCCmec IVa (H131520133)	CC30	NEG	NEG	NEG	NEG	NEG	NEG	NEG	NEG	NEG	NEG	NEG	NEG	NEG	NEG	NEG	NEG	NEG	NEG	NEG	NEG
UKD-15580207	SCCmec IVa (H131520133)	CC88	NEG	NEG	NEG	NEG	NEG	NEG	NEG	NEG	NEG	NEG	NEG	NEG	NEG	NEG	NEG	NEG	NEG	NEG	NEG	NEG
UKD-0521159	SCCmec Iva	CC8	NEG	NEG	NEG	NEG	NEG	NEG	NEG	NEG	NEG	NEG	NEG	NEG	NEG	NEG	NEG	NEG	NEG	NEG	NEG	NEG
Prediction for CMFT503 : HF569113.1	*SCCmec IVa (CMFT503)*		*NEG*	*NEG*	*NEG*	*NEG*	*NEG*	*POS*	*NEG*	*NEG*	*NEG*	*NEG*	*NEG*	*NEG*	*NEG*	*NEG*	*NEG*	*NEG*	*NEG*	*NEG*	*NEG*	*NEG*
Prediction for 21343, GenBank AHKV	*SCCmec IVa (CMFT503)*	*CC88*	*NEG*	*NEG*	*NEG*	*NEG*	*NEG*	*POS*	*NEG*	*NEG*	*NEG*	*NEG*	*NEG*	*NEG*	*NEG*	*NEG*	*NEG*	*NEG*	*NEG*	*NEG*	*NEG*	*NEG*
UKD-1420740	SCCmec IVa (CMFT503)	CC22	NEG	NEG	NEG	NEG	NEG	POS	NEG	NEG	NEG	NEG	NEG	NEG	NEG	NEG	NEG	NEG	NEG	NEG	NEG	NEG
UKD-15575124	SCCmec IVa (CMFT503)	CC22	NEG	NEG	NEG	NEG	NEG	POS	NEG	NEG	NEG	NEG	NEG	NEG	NEG	NEG	NEG	NEG	NEG	NEG	NEG	NEG
UKD-16584947	SCCmec IVa (CMFT503)	CC22	NEG	NEG	NEG	NEG	NEG	POS	NEG	NEG	NEG	NEG	NEG	NEG	NEG	NEG	NEG	NEG	NEG	NEG	NEG	NEG
UKD-16575077	SCCmec IVa (CMFT503)	CC88	NEG	NEG	NEG	NEG	NEG	POS	NEG	NEG	NEG	NEG	NEG	NEG	NEG	NEG	NEG	NEG	NEG	NEG	NEG	NEG
Prediction for TCH1516, GenBank CP000730.1	*SCCmec IVa+ACME1+Cu (USA300)*	*CC8*	*NEG*	*NEG*	*NEG*	*NEG*	*NEG*	*NEG*	*NEG*	*NEG*	*NEG*	*NEG*	*NEG*	*NEG*	*NEG*	*POS*	*NEG*	*NEG*	*NEG*	*NEG*	*NEG*	*NEG*
Reference strain USA300-TCH1516	SCCmec IVa+ACME1+Cu (USA300)	CC8	NEG	NEG	NEG	NEG	NEG	NEG	NEG	NEG	NEG	NEG	NEG	NEG	NEG	POS	NEG	NEG	NEG	NEG	NEG	NEG
Prediction for FPR3757, GenBank CP000255.1	*SCCmec IVa+ACME1+Cu (USA300)*	*CC8*	*NEG*	*NEG*	*NEG*	*NEG*	*NEG*	*NEG*	*NEG*	*NEG*	*NEG*	*NEG*	*NEG*	*NEG*	*NEG*	*POS*	*NEG*	*NEG*	*NEG*	*NEG*	*NEG*	*NEG*
UKD-0516386	SCCmec IVa+ACME1+Cu (USA300)	CC8	NEG	NEG	NEG	NEG	NEG	NEG	NEG	NEG	NEG	NEG	NEG	NEG	NEG	POS	NEG	NEG	NEG	NEG	NEG	NEG
UKD-0815773	SCCmec IVa+ACME1+Cu (USA300)	CC8	NEG	NEG	NEG	NEG	NEG	NEG	NEG	NEG	NEG	NEG	NEG	NEG	NEG	POS	NEG	NEG	NEG	NEG	NEG	NEG
UKD-1414654	SCCmec IVa+ACME1+Cu (USA300)	CC8	NEG	NEG	NEG	NEG	NEG	NEG	NEG	NEG	NEG	NEG	NEG	NEG	NEG	POS	NEG	NEG	NEG	NEG	NEG	NEG
UKD-0408036	SCCmec IVa+ACME2	CC45 [agr I]	NEG	NEG	NEG	NEG	NEG	NEG	NEG	NEG	NEG	NEG	NEG	NEG	NEG	NEG	NEG	NEG	NEG	NEG	NEG	NEG
UKD-0044035	SCCmec IVa+ccrC (Hannover)	CC8	NEG	NEG	NEG	NEG	NEG	NEG	POS	POS	NEG	NEG	NEG	NEG	NEG	NEG	NEG	NEG	NEG	NEG	NEG	NEG
UKD-0136287	SCCmec IVa+ccrC (Hannover)	CC8	NEG	NEG	NEG	NEG	NEG	NEG	POS	POS	NEG	NEG	NEG	NEG	NEG	NEG	NEG	NEG	NEG	NEG	NEG	NEG

Isolate ID	SCCmec type	Clonal complex	METHICILLIN RESISTANCE AND SCCmec TYPING																			
			Potassium-translocating ATPase operon					LytTR domain DNA-binding regulator Q93IB7 (subtyping SCCmec III and IV)	Putative protein D1GU38 (Subtyping SCCmec III, identi-fication of VT,ZH47,VII)	Putative protein D1GU38-TW20 (Subtyping SCCmec III, identi-fication of VII)	Putative ADP-ribosyltransferaseQ933A2	Putative protein D1GU55 (SCCmec III/VII)	Abortive phage resistance protein B2Y834 (SCCmecIV A,G,c and MRSAZH47)	Putative protein B6VQU0 (SCCmec IV h,j)	Q3YK51 (SCCmec IVg)	Putative lipoprotein	Putative protein (Subtyping SCCmec V)	Putative protein located within SCCmec type V/SCCfus elements			Putative protein (SCCmec XI)	Putative protein (composite SCCs)
			kdpA-SCC	kdpB-SCC	kdpC-SCC	kdpD-SCC	kdpE-SCC	Q93IB7	D1GU38	D1GU38-TW20	Q933A2 (SCCmec III, IX)	D1GU55	B2Y834	B6VQU0	Q3YK51	ydhK (FPR3757)	DUF1958	Q4LAG7 (SCC consensus)	Q4LAG7 (SCCmecV, SO385)	Q4LAG7 (SCCfus 45394F/MSSA476)	C5QAP8-M10-61	Q8CU82
Prediction for JCSC1978, GenBank AB063173.1	SCCmec IVb/d/i (JCSC1978/6668/4469)		NEG	NEG	NEG	NEG	NEG	NEG	NEG	NEG	NEG	NEG	NEG	NEG	NEG	NEG	NEG	NEG	NEG	NEG	NEG	NEG
Reference strain JCSC1978 (8/6-3P)	SCCmec IVb/d/i (JCSC1978/6668/4469)	CC8	NEG	NEG	NEG	NEG	NEG	NEG	NEG	NEG	NEG	NEG	NEG	NEG	NEG	NEG	NEG	NEG	NEG	NEG	NEG	NEG
Prediction for JCSC6668, GenBank AB425823.1	SCCmec IVb/d/i (JCSC1978/6668/4469)		NEG	NEG	NEG	NEG	NEG	NEG	NEG	NEG	NEG	NEG	NEG	NEG	NEG	NEG	NEG	NEG	NEG	NEG	NEG	NEG
Reference strain JCSC4469	SCCmec IVb/d/i (JCSC1978/6668/4469)	CC5	NEG	NEG	NEG	NEG	NEG	NEG	NEG	NEG	NEG	NEG	NEG	NEG	NEG	NEG	NEG	NEG	NEG	NEG	NEG	NEG
UKD-1103429	SCCmec IVb/d/i (JCSC1978/6668/4469)	CC7	NEG	NEG	NEG	NEG	NEG	NEG	NEG	NEG	NEG	NEG	NEG	NEG	NEG	NEG	NEG	NEG	NEG	NEG	NEG	NEG
UKD-1306141	SCCmec IVb/d/i (JCSC1978/6668/4469)	CC7	NEG	NEG	NEG	NEG	NEG	NEG	NEG	NEG	NEG	NEG	NEG	NEG	NEG	NEG	NEG	NEG	NEG	NEG	NEG	NEG
UKD-0500089	SCCmec IVb/d/i (JCSC1978/6668/4469)	CC5	NEG	NEG	NEG	NEG	NEG	NEG	NEG	NEG	NEG	NEG	NEG	NEG	NEG	NEG	NEG	NEG	NEG	NEG	NEG	NEG
UKD-0501416	SCCmec IVb/d/i (JCSC1978/6668/4469)	CC5	NEG	NEG	NEG	NEG	NEG	NEG	NEG	NEG	NEG	NEG	NEG	NEG	NEG	NEG	NEG	NEG	NEG	NEG	NEG	NEG
UKD-0934574	SCCmec IVb/d/i (JCSC1978/6668/4469)	CC5	NEG	NEG	NEG	NEG	NEG	NEG	NEG	NEG	NEG	NEG	NEG	NEG	NEG	NEG	NEG	NEG	NEG	NEG	NEG	NEG
UKD-0746779	SCCmec IVb/d/i (JCSC1978/6668/4469)	CC45 [agr I]	NEG	NEG	NEG	NEG	NEG	NEG	NEG	NEG	NEG	NEG	NEG	NEG	NEG	NEG	NEG	NEG	NEG	NEG	NEG	NEG
UKD-0812518	SCCmec IVb/d/i (JCSC1978/6668/4469)	CC45 [agr I]	NEG	NEG	NEG	NEG	NEG	NEG	NEG	NEG	NEG	NEG	NEG	NEG	NEG	NEG	NEG	NEG	NEG	NEG	NEG	NEG
Prediction for 21209, GenBank AGRP	SCCmec IVb/d/i (21209)	CC8	NEG	NEG	NEG	NEG	NEG	NEG	NEG	NEG	NEG	NEG	NEG	NEG	NEG	NEG	NEG	NEG	NEG	NEG	NEG	NEG
UKD-0233579	SCCmec IVb/d/i (21209)	CC8	NEG	NEG	NEG	NEG	NEG	NEG	NEG	NEG	AMB	NEG	NEG	NEG	NEG	NEG	NEG	NEG	NEG	NEG	NEG	NEG
UKD-0504920	SCCmec IVb/d/i (21209)	CC8	NEG	NEG	NEG	NEG	NEG	NEG	NEG	NEG	NEG	NEG	NEG	NEG	NEG	NEG	NEG	NEG	NEG	NEG	NEG	NEG
UKD-0725999	SCCmecIV b/d/i+Cu	CC30	NEG	NEG	NEG	NEG	NEG	NEG	NEG	NEG	NEG	NEG	NEG	NEG	NEG	POS	NEG	NEG	NEG	NEG	NEG	NEG
Prediction for IS-105, GenBank AHLR	SCCmec IVc (IS-105)	CC22	NEG	NEG	NEG	NEG	NEG	NEG	NEG	NEG	NEG	NEG	POS	NEG	NEG	NEG	NEG	NEG	NEG	NEG	NEG	NEG
UKD-0402421	SCCmec IVc (IS-105), merA/B+	CC5	NEG	NEG	NEG	NEG	NEG	NEG	NEG	NEG	NEG	NEG	POS	NEG	NEG	NEG	NEG	NEG	NEG	NEG	NEG	NEG
UKD-0731319	SCCmec IVc (IS-105), merA/B+	CC5	NEG	NEG	NEG	NEG	NEG	NEG	NEG	NEG	NEG	NEG	POS	NEG	NEG	NEG	NEG	NEG	NEG	NEG	NEG	NEG
UKD-0733137	SCCmec IVc (IS-105)	CC8	NEG	NEG	NEG	NEG	NEG	NEG	NEG	NEG	NEG	NEG	POS	NEG	NEG	NEG	NEG	NEG	NEG	NEG	NEG	NEG
UKD-0305690	SCCmec IVc (IS-105)	CC22	NEG	NEG	NEG	NEG	NEG	NEG	NEG	NEG	NEG	NEG	POS	NEG	NEG	NEG	NEG	NEG	NEG	NEG	NEG	NEG
UKD-1424706	SCCmec IVc (IS-105)	CC22	NEG	NEG	NEG	NEG	NEG	NEG	NEG	NEG	NEG	NEG	POS	NEG	NEG	NEG	NEG	NEG	NEG	NEG	NEG	NEG
Prediction for TCH60, GenBank CP002110.1	SCCmec IVc (TCH60)	CC30	NEG	NEG	NEG	NEG	NEG	NEG	NEG	NEG	NEG	NEG	POS	NEG	NEG	NEG	NEG	NEG	NEG	NEG	NEG	NEG
Reference strain JCSC4788	SCCmec IVc (TCH60)	CC8	NEG	NEG	NEG	NEG	NEG	NEG	NEG	NEG	NEG	NEG	POS	NEG	NEG	NEG	NEG	NEG	NEG	NEG	NEG	NEG
UKD-1318318	SCCmec IVc (TCH60)	CC5	NEG	NEG	NEG	NEG	NEG	NEG	NEG	NEG	NEG	NEG	POS	NEG	NEG	NEG	NEG	NEG	NEG	NEG	NEG	NEG
UKD-14580050	SCCmec IVc (TCH60)	CC5	NEG	NEG	NEG	NEG	NEG	NEG	NEG	NEG	NEG	NEG	POS	NEG	NEG	NEG	NEG	NEG	NEG	NEG	NEG	NEG
UKD-08F01314	SCCmec IVc (TCH60)	CC8	NEG	NEG	NEG	NEG	NEG	NEG	NEG	NEG	NEG	NEG	POS	NEG	NEG	NEG	NEG	NEG	NEG	NEG	NEG	NEG
KHDN-50361518	SCCmec IVc (TCH60)	CC8	NEG	NEG	NEG	NEG	NEG	NEG	NEG	NEG	NEG	NEG	POS	NEG	NEG	NEG	NEG	NEG	NEG	NEG	NEG	NEG
UKD-0307912	SCCmec IVc (TCH60)	CC22	NEG	NEG	NEG	NEG	NEG	NEG	NEG	NEG	NEG	NEG	POS	NEG	NEG	NEG	NEG	NEG	NEG	NEG	NEG	NEG
UKD-0220390	SCCmec IVc (TCH60)	CC45 [agr I]	NEG	NEG	NEG	NEG	NEG	NEG	NEG	NEG	NEG	NEG	POS	NEG	NEG	NEG	NEG	NEG	NEG	NEG	NEG	NEG
UKD-0311106	SCCmec IVc (TCH60)	CC45 [agr I]	NEG	NEG	NEG	NEG	NEG	NEG	NEG	NEG	NEG	NEG	POS	NEG	NEG	NEG	NEG	NEG	NEG	NEG	NEG	NEG
UKD-0719855	SCCmec IVc (TCH60)	CC45 [agr I]	NEG	NEG	NEG	NEG	NEG	NEG	NEG	NEG	NEG	NEG	POS	NEG	NEG	NEG	NEG	NEG	NEG	NEG	NEG	NEG
UKD-0422079	SCCmec IVc (TCH60)	CC45 [agr I]	NEG	NEG	NEG	NEG	NEG	NEG	NEG	NEG	NEG	NEG	POS	NEG	NEG	NEG	NEG	NEG	NEG	NEG	NEG	NEG
UKD-0409235	SCCmec IVc (TCH60)	CC80	NEG	NEG	NEG	NEG	NEG	NEG	NEG	NEG	NEG	NEG	POS	NEG	NEG	NEG	NEG	NEG	NEG	NEG	NEG	NEG
UKD-0409807	SCCmec IVc (TCH60)	CC80	NEG	NEG	NEG	NEG	NEG	NEG	NEG	NEG	NEG	NEG	POS	NEG	NEG	NEG	NEG	NEG	NEG	NEG	NEG	NEG
UKD-0416073	SCCmec IVc (TCH60)	CC80	NEG	NEG	NEG	NEG	NEG	NEG	NEG	NEG	NEG	NEG	POS	NEG	NEG	NEG	NEG	NEG	NEG	NEG	NEG	NEG
UKD-0431728	SCCmec IVc (TCH60)	CC80	NEG	NEG	NEG	NEG	NEG	NEG	NEG	NEG	AMB	NEG	POS	NEG	NEG	NEG	NEG	NEG	NEG	NEG	NEG	NEG
UKD-1001508	SCCmec IVc (TCH60)	CC80	NEG	NEG	NEG	NEG	NEG	NEG	NEG	NEG	NEG	NEG	POS	NEG	NEG	NEG	NEG	NEG	NEG	NEG	NEG	NEG
UKD-1010412	SCCmec IVc (TCH60)	CC80	NEG	NEG	NEG	AMB	NEG	NEG	NEG	NEG	NEG	NEG	POS	NEG	NEG	NEG	NEG	NEG	NEG	NEG	NEG	NEG
UKD-12753005	SCCmec IVc (TCH60)	CC80	NEG	NEG	NEG	NEG	NEG	NEG	NEG	NEG	NEG	NEG	POS	NEG	NEG	NEG	NEG	NEG	NEG	NEG	NEG	NEG
UKD-15574809	SCCmec IVc (TCH60)	CC80	NEG	NEG	NEG	NEG	NEG	NEG	NEG	NEG	NEG	NEG	POS	NEG	NEG	NEG	NEG	NEG	NEG	NEG	NEG	NEG
KHDN-16120291	SCCmec IVc (TCH60)	CC88	NEG	NEG	NEG	NEG	NEG	NEG	NEG	NEG	NEG	NEG	POS	NEG	NEG	NEG	NEG	NEG	NEG	NEG	NEG	NEG
Prediction for WW2703/97, GenBank ACSW	SCCmec IVc (WW2703/97)	CC30	NEG	NEG	NEG	NEG	NEG	NEG	NEG	NEG	NEG	NEG	POS	NEG	NEG	NEG	NEG	NEG	NEG	NEG	NEG	NEG
UKD-14580088	SCCmec IVc (WW2703/97)	CC398	NEG	NEG	NEG	NEG	NEG	NEG	NEG	NEG	NEG	NEG	POS	NEG	NEG	NEG	NEG	NEG	NEG	NEG	NEG	NEG
Prediction for SA_ST125_MupR, GenBank ASTH	SCCmec IVc+speG+ccrAB4 (SA_ST125)	CC5	NEG	NEG	NEG	NEG	NEG	NEG	NEG	NEG	NEG	NEG	POS	NEG	NEG	NEG	NEG	NEG	NEG	NEG	NEG	NEG
UKD-0439909	SCCmec IVc+speG+ccrAB4 (SA_ST125)	CC5	NEG	NEG	NEG	NEG	NEG	NEG	NEG	NEG	NEG	NEG	POS	NEG	NEG	NEG	NEG	NEG	NEG	NEG	NEG	NEG
Prediction for MRSA177, GenBank AECP	SCCmec IVc+Cu/Hg (MRSA177)		NEG	NEG	NEG	NEG	NEG	NEG	NEG	NEG	NEG	NEG	POS	NEG	NEG	POS	NEG	POS	POS	NEG	NEG	NEG
UKD-1279734	SCCmec IVc+Cu/Hg (MRSA177)	CC8	NEG	NEG	NEG	NEG	NEG	NEG	NEG	NEG	NEG	NEG	POS	NEG	NEG	POS	NEG	POS	POS	NEG	NEG	NEG
UKD-14580899	SCCmec IVc+Cu/Hg (MRSA177)	CC8	NEG	NEG	NEG	NEG	NEG	NEG	NEG	NEG	NEG	NEG	POS	NEG	NEG	POS	NEG	POS	POS	NEG	NEG	NEG
UKD-16002228	SCCmec IVc+Cu/Hg (MRSA177)	CC8	NEG	NEG	NEG	NEG	NEG	NEG	NEG	NEG	NEG	NEG	POS	NEG	NEG	POS	NEG	POS	POS	NEG	NEG	NEG
Prediction for HO 5096 0412, GenBank HE681097.1	SCCmec IVh/j (HO50960412/JCSC6670)	CC22	NEG	NEG	NEG	NEG	NEG	NEG	NEG	NEG	NEG	NEG	NEG	POS	NEG	NEG	NEG	NEG	NEG	NEG	NEG	NEG
Prediction for JCSC6670, GenBank AB425824.1	SCCmec IVh/j (HO50960412/JCSC6670)		NEG	NEG	NEG	NEG	NEG	NEG	NEG	NEG	NEG	NEG	NEG	POS	NEG	NEG	NEG	NEG	NEG	NEG	NEG	NEG
Reference strain Dublin_DSH_E1749	SCCmec IVh/j (HO50960412/JCSC6670)		NEG	NEG	NEG	NEG	NEG	NEG	NEG	NEG	NEG	NEG	NEG	POS	NEG	NEG	NEG	NEG	NEG	NEG	NEG	NEG
UKD-14576248	SCCmec IVh/j (HO50960412/JCSC6670)	CC8	NEG	NEG	NEG	NEG	NEG	NEG	NEG	NEG	NEG	NEG	NEG	POS	NEG	NEG	NEG	NEG	NEG	NEG	NEG	NEG
UKD-0120122	SCCmec IVh/j (HO50960412/JCSC6670)	CC22	NEG	NEG	NEG	NEG	NEG	NEG	NEG	NEG	NEG	NEG	NEG	POS	NEG	NEG	NEG	NEG	NEG	NEG	NEG	NEG
UKD-0205319	SCCmec IVh/j (HO50960412/JCSC6670)	CC22	NEG	NEG	NEG	NEG	NEG	NEG	NEG	NEG	NEG	NEG	NEG	POS	NEG	NEG	NEG	NEG	NEG	NEG	NEG	NEG
UKD-0221259	SCCmec IVh/j (HO50960412/JCSC6670)	CC22	NEG	NEG	NEG	NEG	NEG	NEG	NEG	NEG	NEG	NEG	NEG	POS	NEG	NEG	NEG	NEG	NEG	NEG	NEG	NEG
UKD-0418654	SCCmec IVh/j (HO50960412/JCSC6670)	CC22	NEG	NEG	NEG	NEG	NEG	NEG	NEG	NEG	NEG	NEG	NEG	POS	NEG	NEG	NEG	NEG	NEG	NEG	AMB	NEG
UKD-0429721	SCCmec IVh/j (HO50960412/JCSC6670)	CC22	NEG	NEG	NEG	NEG	NEG	NEG	NEG	NEG	NEG	NEG	NEG	POS	NEG	NEG	NEG	NEG	NEG	NEG	NEG	NEG
UKD-1116194	SCCmec IVh/j (HO50960412/JCSC6670)	CC22	NEG	NEG	NEG	NEG	NEG	NEG	NEG	NEG	NEG	NEG	NEG	POS	NEG	NEG	NEG	NEG	NEG	NEG	NEG	NEG
UKD-1122367	SCCmec IVh/j (HO50960412/JCSC6670)	CC22	NEG	NEG	NEG	NEG	NEG	NEG	NEG	NEG	NEG	NEG	NEG	POS	NEG	NEG	NEG	NEG	NEG	NEG	NEG	NEG
UKD-14012840	SCCmec IVh/j (HO50960412/JCSC6670)	CC22	NEG	NEG	NEG	NEG	NEG	NEG	NEG	NEG	NEG	NEG	NEG	POS	NEG	NEG	NEG	NEG	NEG	NEG	NEG	NEG
UKD-14579833	SCCmec IVh/j (HO50960412/JCSC6670)	CC22	NEG	NEG	NEG	NEG	NEG	NEG	NEG	NEG	NEG	NEG	NEG	POS	NEG	NEG	NEG	NEG	NEG	NEG	NEG	NEG
UKD-15579198	SCCmec IVh/j (HO50960412/JCSC6670)	CC22	NEG	NEG	NEG	NEG	NEG	NEG	NEG	NEG	NEG	NEG	NEG	POS	NEG	NEG	NEG	NEG	NEG	NEG	NEG	NEG
UKD-4052193	SCCmec IVh/j (HO50960412/JCSC6670)	CC22	NEG	NEG	NEG	NEG	NEG	NEG	NEG	NEG	NEG	NEG	NEG	POS	NEG	NEG	NEG	NEG	NEG	NEG	NEG	NEG
UKD-124968	SCCmec IVh/j (HO50960412/JCSC6670)	CC22	NEG	NEG	NEG	NEG	NEG	NEG	NEG	NEG	NEG	NEG	NEG	POS	NEG	NEG	NEG	NEG	NEG	NEG	NEG	NEG
UKD-14572546	SCCmec IVh/j (HO50960412/JCSC6670)	CC22	NEG	NEG	NEG	NEG	NEG	NEG	NEG	NEG	NEG	NEG	NEG	POS	NEG	NEG	NEG	NEG	NEG	NEG	NEG	NEG
UKD-14582287	SCCmec IVh/j (HO50960412/JCSC6670)	CC22	NEG	NEG	NEG	NEG	NEG	NEG	NEG	NEG	NEG	NEG	NEG	POS	NEG	NEG	NEG	NEG	NEG	NEG	NEG	NEG
UKD-14583376	SCCmec IVh/j (HO50960412/JCSC6670)	CC22	NEG	NEG	NEG	NEG	NEG	NEG	NEG	NEG	NEG	NEG	NEG	POS	NEG	NEG	NEG	NEG	NEG	NEG	NEG	NEG
UKD-14583496	SCCmec IVh/j (HO50960412/JCSC6670)	CC22	NEG	NEG	NEG	NEG	NEG	NEG	NEG	NEG	NEG	NEG	NEG	POS	NEG	NEG	NEG	NEG	NEG	NEG	NEG	NEG
UKD-0013324	SCCmec IVh/j (HO50960412/JCSC6670)	CC22	NEG	NEG	NEG	NEG	NEG	NEG	NEG	NEG	NEG	NEG	NEG	POS	NEG	NEG	NEG	NEG	NEG	NEG	NEG	NEG
UKD-0125329	SCCmec IVh/j (HO50960412/JCSC6670)	CC22	NEG	NEG	NEG	NEG	NEG	NEG	NEG	NEG	NEG	NEG	NEG	POS	NEG	NEG	NEG	NEG	NEG	NEG	NEG	NEG
UKD-0215498	SCCmec IVh/j (HO50960412/JCSC6670)	CC22	NEG	NEG	NEG	NEG	NEG	NEG	NEG	NEG	AMB	NEG	NEG	POS	NEG	NEG	NEG	NEG	NEG	NEG	NEG	NEG
Prediction for M08-0126, GenBank FR753166.1	SCCmec IVh/j+ACME2 (M08-0126)	CC22	NEG	NEG	NEG	NEG	NEG	NEG	NEG	NEG	NEG	NEG	NEG	POS	NEG	NEG	NEG	NEG	NEG	NEG	NEG	NEG
UKD-13794831	SCCmec IVh/j+ACME2 (M08-0126)	CC22	NEG	NEG	NEG	NEG	NEG	NEG	NEG	NEG	NEG	NEG	NEG	POS	NEG	NEG	NEG	NEG	NEG	NEG	NEG	NEG
UKD-5150204	SCCmec IV+speG+Cu/czrC+ccrAB4	CC22	NEG	NEG	NEG	NEG	NEG	NEG	NEG	NEG	NEG	NEG	NEG	NEG	NEG	POS	NEG	NEG	NEG	NEG	NEG	NEG
UKD-15578423	SCCmec IV+speG+czrC+ccrAB4 var. 1	CC22	NEG	NEG	NEG	NEG	NEG	NEG	NEG	NEG	NEG	NEG	NEG	NEG	NEG	NEG	NEG	NEG	NEG	NEG	NEG	NEG
UKD-16582870	SCCmec IV+speG+czrC+ccrAB4 var. 1	CC22	NEG	NEG	NEG	NEG	NEG	NEG	NEG	NEG	NEG	NEG	NEG	NEG	NEG	NEG	NEG	NEG	NEG	NEG	NEG	NEG
UKD-15571940	SCCmec IV+speG+czrC+ccrAB4 var. 2	CC22	NEG	NEG	NEG	NEG	NEG	NEG	NEG	NEG	NEG	NEG	NEG	NEG	NEG	NEG	NEG	NEG	NEG	NEG	NEG	NEG

Isolate ID	SCCmec type	Clonal complex	METHICILLIN RESISTANCE AND SCCmec TYPING																			
			Potassium-translocating ATPase operon					LytTR domain DNA-binding regulator Q93IB7 (subtyping SCCmec III and IV)	Putative protein D1GU38 (Subtyping SCCmec III, identi-fication of VT,ZH47,VII)	Putative protein D1GU38-TW20 (Subtyping SCCmec III, identi-fication of VII)	Putative ADP-ribosyltransfer aseQ933A2	Putative protein D1GU55 (SCCmec III/VII)	Abortive phage resistance protein B2Y834 (SCCmecIV A,G,c and MRSAZH47)	Putative protein B6VQU0 (SCCmec IV h,j)	Q3YK51 (SCCmec IVg)	Putative lipoprotein	Putative protein (Subtyping SCCmec V)	Putative protein located within SCCmec type V/SCCfus elements			Putative protein (SCCmec XI)	Putative protein (composite SCCs)
			kdpA-SCC	kdpB-SCC	kdpC-SCC	kdpD-SCC	kdpE-SCC	Q93IB7	D1GU38	D1GU38-TW20	Q933A2 (SCCmec III, IX)	D1GU55	B2Y834	B6VQU0	Q3YK51	ydhK (FPR3757)	DUF1958	Q4LAG7 (SCC consensus)	Q4LAG7 (SCCmecV, SO385)	Q4LAG7 (SCCfus 45394F/MSSA476)	C5QAP8-M10-61	Q8CU82
Prediction for CN1, GenBank CP003979.1	SCCmec IVA (CN1)	CC8 (ST72)	NEG	NEG	NEG	NEG	NEG	NEG	NEG	NEG	NEG	NEG	POS	NEG	NEG	NEG	NEG	NEG	NEG	NEG	NEG	NEG
UKD-0900871	SCCmec IVA (CN1)	CC8 (ST72)	NEG	NEG	NEG	NEG	NEG	NEG	NEG	NEG	NEG	NEG	POS	NEG	NEG	NEG	NEG	NEG	NEG	NEG	NEG	NEG
Prediction for CMFT1723, GenBank HF569096.1	SCCmec V (Bengal Bay)		NEG	NEG	NEG	NEG	NEG	NEG	NEG	NEG	NEG	NEG	NEG	NEG	NEG	NEG	NEG	POS	POS	NEG	NEG	NEG
KHDN-10405134	SCCmec V (Bengal Bay)	CC1	NEG	NEG	NEG	NEG	NEG	NEG	NEG	NEG	NEG	NEG	NEG	NEG	NEG	NEG	NEG	POS	POS	NEG	NEG	NEG
UKD-0932405	SCCmec V (Bengal Bay)	CC1 (ST573/772)	NEG	NEG	NEG	NEG	NEG	NEG	NEG	NEG	NEG	NEG	NEG	NEG	NEG	NEG	NEG	POS	POS	NEG	NEG	NEG
UKD-1003510	SCCmec V (Bengal Bay)	CC1 (ST573/772)	NEG	NEG	NEG	NEG	NEG	NEG	NEG	NEG	NEG	NEG	NEG	NEG	NEG	NEG	NEG	POS	POS	NEG	NEG	NEG
UKD-1112420	SCCmec V (Bengal Bay)	CC1 (ST573/772)	NEG	NEG	NEG	NEG	NEG	NEG	NEG	NEG	NEG	NEG	NEG	NEG	NEG	NEG	NEG	POS	POS	NEG	NEG	NEG
UKD-1275832	SCCmec V (Bengal Bay)	CC1 (ST573/772)	NEG	NEG	NEG	NEG	NEG	NEG	NEG	NEG	NEG	NEG	NEG	NEG	NEG	NEG	NEG	POS	POS	NEG	NEG	NEG
UKD-13023811	SCCmec V (Bengal Bay)	CC1 (ST573/772)	NEG	NEG	NEG	NEG	NEG	NEG	NEG	NEG	NEG	NEG	NEG	NEG	NEG	NEG	NEG	POS	POS	NEG	NEG	NEG
UKD-14572163	SCCmec V	CC5	NEG	NEG	NEG	NEG	NEG	NEG	NEG	NEG	NEG	NEG	NEG	NEG	NEG	NEG	NEG	POS	POS	NEG	NEG	NEG
KHDN-10421133	SCCmec V+fus	CC97	NEG	NEG	NEG	NEG	NEG	NEG	NEG	NEG	NEG	NEG	NEG	NEG	NEG	NEG	NEG	POS	POS	NEG	NEG	NEG
KHDN-21343541	SCCmec V+fus+tir+ccrAB1	CC1	NEG	NEG	NEG	NEG	NEG	NEG	NEG	NEG	NEG	NEG	NEG	NEG	NEG	NEG	NEG	POS	POS	POS	NEG	NEG
KHDN-10416893	SCCmec V+czrC+speG+ccrAB4	CC22	NEG	NEG	NEG	NEG	NEG	NEG	NEG	NEG	NEG	NEG	NEG	NEG	NEG	NEG	NEG	POS	POS	NEG	NEG	POS
Prediction for GR1, GenBank AJLX	SCCmec VT (GR1)	CC361	NEG	NEG	NEG	NEG	NEG	NEG	POS	NEG	NEG	NEG	NEG	NEG	NEG	NEG	NEG	POS	POS	NEG	NEG	NEG
UKD-0422872	SCCmec VT (GR1)	CC152	NEG	NEG	NEG	NEG	NEG	NEG	POS	NEG	NEG	NEG	NEG	NEG	NEG	NEG	NEG	POS	POS	NEG	NEG	NEG
Prediction for PM1, GenBank BAFA	SCCmec VT (PM1)	CC59	NEG	NEG	NEG	NEG	NEG	NEG	POS	NEG	NEG	NEG	NEG	NEG	NEG	NEG	POS	POS	POS	NEG	NEG	NEG
KHDN-000871	SCCmec VT (PM1)	CC45 (agr I)	NEG	NEG	NEG	NEG	NEG	NEG	POS	NEG	NEG	NEG	NEG	NEG	NEG	NEG	POS	POS	POS	NEG	NEG	NEG
UKD-0725052	SCCmec VT (PM1)	CC59	NEG	NEG	NEG	NEG	NEG	NEG	POS	NEG	NEG	NEG	NEG	NEG	NEG	NEG	POS	POS	POS	NEG	NEG	NEG
UKD-1312138	SCCmec VT (PM1)	CC59	NEG	NEG	NEG	NEG	NEG	NEG	POS	NEG	NEG	NEG	NEG	NEG	NEG	NEG	POS	POS	POS	NEG	NEG	NEG
UKD-Z4728	SCCmec VT (PM1)	CC59	NEG	NEG	NEG	NEG	NEG	NEG	POS	NEG	NEG	NEG	NEG	NEG	NEG	NEG	POS	POS	POS	NEG	NEG	NEG
UKD-K0199	SCCmec VT unknown var.1	CC7	NEG	NEG	NEG	NEG	NEG	NEG	POS	NEG	NEG	NEG	NEG	NEG	NEG	NEG	NEG	NEG	NEG	NEG	NEG	NEG
UKD-15584619	SCCmec VT unknown var.2	CC30	NEG	NEG	NEG	NEG	NEG	NEG	POS	POS	NEG	NEG	NEG	NEG	NEG	NEG	POS	POS	POS	NEG	NEG	NEG
UKD-0712568	SCCmec VT unknown var.3	CC45 (agr I)	NEG	NEG	NEG	NEG	NEG	NEG	POS	NEG	NEG	NEG	NEG	NEG	NEG	NEG	POS	POS	POS	NEG	NEG	NEG
KHDN-10420296	SCCmec VT+ACME2	CC45 (agr I)	NEG	NEG	NEG	NEG	NEG	NEG	POS	NEG	NEG	NEG	NEG	NEG	NEG	NEG	NEG	POS	POS	NEG	NEG	NEG
UKD-15577956	SCCmec VT+Cu/czrC+ccrA1	CC398	NEG	NEG	NEG	NEG	NEG	NEG	POS	NEG	NEG	NEG	POS	NEG	NEG	POS	NEG	POS	POS	NEG	NEG	NEG
Prediction for S0385, GenBank AM990992	SCCmec VT+czrC (SO385)	CC398	NEG	NEG	NEG	NEG	NEG	NEG	POS	NEG	NEG	NEG	NEG	NEG	NEG	POS	NEG	POS	POS	NEG	NEG	NEG
UKD-0628560	SCCmec VT+czrC (SO385)	CC8	NEG	NEG	NEG	NEG	NEG	NEG	POS	NEG	NEG	NEG	NEG	NEG	NEG	POS	NEG	POS	POS	NEG	NEG	NEG
UKD-0510018	SCCmec VT+czrC (SO385)	CC398	NEG	NEG	NEG	NEG	NEG	NEG	POS	NEG	NEG	NEG	NEG	NEG	NEG	POS	NEG	POS	POS	NEG	NEG	NEG
UKD-0903558	SCCmec VT+czrC (SO385)	CC398	NEG	NEG	NEG	NEG	NEG	NEG	POS	NEG	NEG	NEG	NEG	NEG	NEG	POS	NEG	POS	POS	NEG	NEG	NEG
UKD-0929815	SCCmec VT+czrC (SO385)	CC398	NEG	NEG	NEG	NEG	NEG	NEG	POS	NEG	NEG	NEG	NEG	NEG	NEG	POS	NEG	POS	POS	NEG	NEG	NEG
UKD-0907762	SCCmec VT+czrC (SO385)	CC398	NEG	NEG	NEG	NEG	NEG	NEG	POS	NEG	NEG	NEG	NEG	NEG	NEG	POS	NEG	POS	POS	NEG	NEG	NEG
UKD-1025105	SCCmec VT+czrC (SO385)	CC398	NEG	NEG	NEG	NEG	NEG	NEG	POS	NEG	NEG	NEG	NEG	NEG	NEG	POS	NEG	POS	POS	NEG	NEG	NEG
UKD-11780898	SCCmec VT+czrC (SO385)	CC398	NEG	NEG	NEG	NEG	NEG	NEG	POS	NEG	NEG	NEG	NEG	NEG	NEG	POS	NEG	POS	POS	NEG	NEG	NEG
UKD-1377009	SCCmec VT+czrC (SO385)	CC398	NEG	NEG	NEG	NEG	NEG	NEG	POS	NEG	NEG	NEG	NEG	NEG	NEG	POS	NEG	POS	POS	NEG	NEG	NEG
UKD-143406	SCCmec VT+czrC (SO385)	CC398	NEG	NEG	NEG	NEG	NEG	NEG	POS	NEG	NEG	NEG	NEG	NEG	NEG	POS	NEG	POS	POS	NEG	NEG	NEG
UKD-1400363	SCCmec VT+czrC (SO385)	CC398	NEG	NEG	NEG	NEG	NEG	NEG	POS	NEG	NEG	NEG	NEG	NEG	NEG	POS	NEG	POS	POS	NEG	NEG	NEG
UKD-14571852	SCCmec VT+czrC (SO385)	CC398	NEG	NEG	NEG	NEG	NEG	NEG	POS	NEG	NEG	NEG	NEG	NEG	NEG	POS	NEG	POS	POS	NEG	NEG	NEG
UKD-14582574	SCCmec VT+czrC (SO385)	CC398	NEG	NEG	NEG	NEG	NEG	NEG	POS	NEG	NEG	NEG	NEG	NEG	NEG	POS	NEG	POS	POS	NEG	NEG	NEG
UKD-152108	SCCmec VT+czrC (SO385)	CC398	NEG	NEG	NEG	NEG	NEG	NEG	POS	NEG	NEG	NEG	NEG	NEG	NEG	POS	NEG	POS	POS	NEG	NEG	NEG
UKD-15573321	SCCmec VT+czrC (SO385)	CC398	NEG	NEG	NEG	NEG	NEG	NEG	POS	NEG	NEG	NEG	NEG	NEG	NEG	POS	NEG	POS	POS	NEG	NEG	NEG
UKD-15584034	SCCmec VT+czrC , ydhK-neg. variant	CC398	NEG	NEG	NEG	NEG	NEG	NEG	POS	NEG	NEG	NEG	NEG	NEG	NEG	NEG	NEG	POS	POS	NEG	NEG	NEG
UKD-0932581	SCCmecVT+fus+czrC	CC22	NEG	NEG	NEG	NEG	NEG	NEG	POS	NEG	NEG	NEG	NEG	NEG	NEG	NEG	NEG	NEG	POS	NEG	NEG	NEG
UKD-1121994	SCCmecVT+fus+czrC	CC22	NEG	NEG	NEG	NEG	NEG	NEG	POS	NEG	NEG	NEG	NEG	NEG	NEG	POS	NEG	POS	POS	NEG	NEG	NEG
UKD-1272252	SCCmecVT+fus+czrC	CC22	NEG	NEG	NEG	NEG	NEG	NEG	POS	NEG	NEG	NEG	NEG	NEG	NEG	POS	NEG	POS	POS	NEG	NEG	NEG
Prediction for MRSA18	SCCmec VI+fus (MRSA18)		NEG	NEG	NEG	NEG	NEG	NEG	NEG	NEG	NEG	NEG	NEG	NEG	NEG	NEG	NEG	POS	NEG	POS	NEG	NEG
UKD-1103380	SCCmec VI+fus (MRSA18)	CC7	NEG	NEG	NEG	NEG	NEG	NEG	NEG	NEG	NEG	NEG	NEG	NEG	NEG	NEG	NEG	POS	NEG	POS	NEG	NEG
Prediction for M10/0061, GenBank FR823292.1	SCCmec XI (LGA251/M10-61)	CC130	NEG	NEG	NEG	NEG	NEG	NEG	NEG	NEG	NEG	NEG	NEG	NEG	NEG	NEG	NEG	NEG	NEG	NEG	POS	NEG
Reference strain Dublin-DSH_M10 0061	SCCmec XI (LGA251/M10-61)	CC130	NEG	NEG	NEG	NEG	NEG	NEG	NEG	NEG	NEG	NEG	NEG	NEG	NEG	NEG	NEG	NEG	NEG	NEG	POS	NEG
Prediction for LGA251, GenBank FR821779.1	SCCmec XI (LGA251/M10-61)	ST425	NEG	NEG	NEG	NEG	NEG	NEG	NEG	NEG	NEG	NEG	NEG	NEG	NEG	NEG	NEG	NEG	NEG	NEG	POS	NEG
UKD-14015835	SCCmec XI (LGA251/M10-61)	CC130	NEG	NEG	NEG	NEG	NEG	NEG	NEG	NEG	NEG	NEG	NEG	NEG	NEG	NEG	NEG	NEG	NEG	NEG	POS	NEG
UKD-16573252	SCCmec XI (LGA251/M10-61)	CC130	NEG	NEG	NEG	NEG	NEG	NEG	NEG	NEG	NEG	NEG	NEG	NEG	NEG	NEG	NEG	NEG	NEG	NEG	POS	NEG
UKD-0217002	PseudoSCCmec, class B mec complex+Hg	CC8	NEG	NEG	NEG	NEG	NEG	NEG	NEG	NEG	NEG	NEG	NEG	NEG	NEG	NEG	NEG	NEG	NEG	NEG	NEG	NEG
UKD-0008510	PseudoSCCmec, class B mec complex+Hg	CC8	NEG	NEG	NEG	NEG	NEG	NEG	NEG	NEG	NEG	NEG	NEG	NEG	NEG	NEG	NEG	NEG	NEG	NEG	NEG	NEG
UKD-0110962	PseudoSCCmec, class B mec complex+Hg	CC8	NEG	NEG	NEG	NEG	NEG	NEG	NEG	NEG	NEG	NEG	NEG	NEG	NEG	NEG	NEG	NEG	NEG	NEG	NEG	NEG
KHDN-10408593	PseudoSCCmec, class B mec complex+Cu/Hg	CC8	NEG	NEG	NEG	NEG	NEG	NEG	NEG	NEG	NEG	NEG	NEG	NEG	NEG	POS	NEG	NEG	NEG	NEG	NEG	NEG
UKD-0007201	PseudoSCCmec, class B mec complex	CC5	NEG	NEG	NEG	NEG	NEG	NEG	NEG	NEG	NEG	NEG	NEG	NEG	NEG	NEG	NEG	NEG	NEG	NEG	NEG	NEG
UKD-0033204	PseudoSCCmec, class B mec complex	CC5	NEG	NEG	NEG	NEG	NEG	NEG	NEG	NEG	NEG	NEG	NEG	NEG	NEG	NEG	NEG	NEG	NEG	NEG	NEG	NEG
UKD-0134329	PseudoSCCmec, class B mec complex	CC5	NEG	NEG	NEG	NEG	NEG	NEG	NEG	NEG	NEG	NEG	NEG	NEG	NEG	NEG	NEG	NEG	NEG	NEG	NEG	NEG
UKD-0806946	PseudoSCCmec class C+As/Cu	CC398	NEG	NEG	NEG	NEG	NEG	NEG	NEG	NEG	NEG	NEG	NEG	NEG	NEG	POS	NEG	NEG	NEG	NEG	NEG	NEG
UKD-0500610	ACME2	CC8	NEG	NEG	NEG	NEG	NEG	NEG	NEG	NEG	NEG	NEG	NEG	NEG	NEG	NEG	NEG	NEG	NEG	NEG	NEG	NEG
UKD-0610438	ACME2	CC8	NEG	NEG	NEG	NEG	NEG	NEG	NEG	NEG	NEG	NEG	NEG	NEG	NEG	NEG	NEG	NEG	NEG	NEG	NEG	NEG
KHDN-10409344	ACME2	CC8	NEG	NEG	NEG	NEG	NEG	NEG	NEG	NEG	NEG	NEG	NEG	NEG	NEG	NEG	NEG	NEG	NEG	NEG	NEG	NEG
UKD-0322559	ACME2+speG+czrC+ccrAB4	CC8	NEG	NEG	NEG	NEG	NEG	NEG	NEG	NEG	NEG	NEG	NEG	NEG	NEG	NEG	NEG	NEG	NEG	NEG	NEG	NEG
UKD-0501998	ACME2+speG+czrC+ccrAB4	CC8	NEG	NEG	NEG	NEG	NEG	NEG	NEG	NEG	NEG	NEG	NEG	NEG	NEG	NEG	NEG	NEG	NEG	NEG	NEG	NEG
UKD-0505213	ACME2+speG+czrC+ccrAB4	CC8	NEG	NEG	NEG	NEG	NEG	NEG	NEG	NEG	NEG	NEG	NEG	NEG	NEG	NEG	NEG	NEG	NEG	NEG	NEG	NEG
Prediction for MSSA476, GenBank BX571857.1	fus+tir+ccrAB1 (MSSA476)	CC1	NEG	NEG	NEG	NEG	NEG	NEG	NEG	NEG	NEG	NEG	NEG	NEG	NEG	NEG	NEG	POS	NEG	POS	NEG	NEG
Reference strain Sanger 476	fus+tir+ccrAB1 (MSSA476)	CC1	NEG	NEG	NEG	NEG	NEG	NEG	NEG	NEG	NEG	NEG	NEG	NEG	NEG	NEG	NEG	POS	NEG	POS	NEG	NEG
UKD-14023914	fus+tir+ccrAB1 (MSSA476)	CC1	NEG	NEG	NEG	NEG	NEG	NEG	NEG	NEG	NEG	NEG	NEG	NEG	NEG	NEG	NEG	POS	NEG	POS	NEG	NEG
UKD-K00034	kdp+ccrAB2	CC182	POS	POS	POS	POS	POS	NEG	NEG	NEG	NEG	NEG	NEG	NEG	NEG	NEG	NEG	NEG	NEG	NEG	NEG	NEG
UKD-15580369	As+ccrAB4	CC22	NEG	NEG	NEG	NEG	NEG	NEG	NEG	NEG	NEG	NEG	NEG	POS	NEG	NEG	NEG	NEG	NEG	NEG	NEG	NEG
UKD-15570100	ACME3+ccrAB1 (Strain 21342)	CC80	NEG	NEG	NEG	NEG	NEG	NEG	NEG	NEG	NEG	NEG	NEG	NEG	NEG	NEG	NEG	NEG	NEG	NEG	NEG	NEG
UKD-1010678	speG+czrC+ccrAB4	CC8	NEG	NEG	NEG	NEG	NEG	NEG	NEG	NEG	NEG	NEG	NEG	NEG	NEG	NEG	NEG	NEG	NEG	NEG	NEG	NEG
UKD-1008243	speG+czrC+ccrAB4	CC8	NEG	NEG	NEG	NEG	NEG	NEG	NEG	NEG	NEG	NEG	NEG	NEG	NEG	NEG	NEG	NEG	NEG	NEG	NEG	NEG
UKD-1009430	speG+czrC+ccrAB4	CC8	NEG	NEG	NEG	NEG	NEG	NEG	NEG	NEG	NEG	NEG	NEG	NEG	NEG	NEG	NEG	NEG	NEG	NEG	NEG	NEG
UKD-12071828	speG+czrC+ccrAB4	CC22	NEG	NEG	NEG	NEG	NEG	NEG	NEG	NEG	NEG	NEG	NEG	NEG	NEG	NEG	NEG	NEG	NEG	NEG	NEG	NEG
UKD-14011883	speG+czrC+ccrAB4	CC22	NEG	NEG	NEG	NEG	NEG	NEG	NEG	NEG	NEG	NEG	NEG	NEG	NEG	NEG	NEG	NEG	NEG	NEG	NEG	NEG
UKD-15008293	speG+czrC+ccrAB4	CC22	NEG	NEG	NEG	NEG	NEG	NEG	NEG	NEG	NEG	NEG	NEG	NEG	NEG	NEG	NEG	NEG	NEG	NEG	NEG	NEG
UKD-1514877	speG+czrC+ccrAB4	CC22	NEG	NEG	NEG	NEG	NEG	NEG	NEG	NEG	NEG	NEG	NEG	NEG	NEG	NEG	NEG	NEG	NEG	NEG	NEG	NEG
UKD-15580971	speG+czrC+ccrAB4	CC22	NEG	NEG	NEG	NEG	NEG	NEG	NEG	NEG	NEG	NEG	NEG	NEG	NEG	NEG	NEG	NEG	NEG	NEG	NEG	NEG
UKD-15582646	speG+czrC+ccrAB4	CC22	NEG	NEG	NEG	NEG	NEG	NEG	NEG	NEG	NEG	NEG	NEG	NEG	NEG	NEG	NEG	NEG	NEG	NEG	NEG	NEG

Isolate ID	SCCmec type	Clonal complex	METHICILLIN RESISTANCE AND SCCmec TYPING																					
			Putative protein (from CC779-MRSA with novel SCCmec+ SCCfus)	CRISPR-associated endonuclease 1	SCC fusidic acid resistance locus			ACME-associated genes				ACME-associated oligopeptide permease, channel-forming protein					Alcohol dehydro-genase	Spermidine N-acetyltrans-ferase	cassette chromosome recombinase genes					
			D3JD07	cas1v2-M060171	fusC (Q6GD50)	tirS	yeeA (SCCmecIV/ fus)	arcA-SCC	arcB-SCC	arcC-SCC	arcD-SCC	opp3B	opp3B-FPR3757	opp3B-C427	opp3C-FPR3757	opp3C-C427	adhC	speG	ccrA-1	ccrB-1	ccrA-2	ccrB-2	ccrA-3	ccrB-3
VIRTUAL HYBRIDISATION for COL, GenBank CP000046	SCCmec I (COL)	CC8	NEG	NEG	NEG	NEG	NEG	NEG	NEG	NEG	NEG	NEG	NEG	NEG	NEG	NEG	NEG	NEG	POS	POS	NEG	NEG	NEG	NEG
Reference strain COL	SCCmec I (COL)	CC8	NEG	NEG	NEG	NEG	NEG	NEG	NEG	NEG	NEG	NEG	NEG	NEG	NEG	NEG	NEG	NEG	POS	POS	NEG	NEG	NEG	NEG
UKD-0007063	SCCmec I (COL)	CC5	NEG	NEG	NEG	NEG	NEG	NEG	NEG	NEG	NEG	NEG	NEG	NEG	NEG	NEG	NEG	NEG	POS	POS	NEG	NEG	NEG	NEG
UKD-0220759	SCCmec I (COL)	CC5	NEG	NEG	NEG	NEG	NEG	NEG	NEG	NEG	NEG	NEG	NEG	NEG	NEG	NEG	NEG	NEG	POS	POS	NEG	NEG	NEG	NEG
UKD-0308986	SCCmec I (COL)	CC5	NEG	NEG	NEG	NEG	NEG	NEG	NEG	NEG	NEG	NEG	NEG	NEG	NEG	NEG	NEG	NEG	POS	POS	NEG	NEG	NEG	NEG
UKD-0500009	SCCmec I (COL)	CC5	NEG	NEG	NEG	NEG	NEG	NEG	NEG	NEG	NEG	NEG	NEG	NEG	NEG	NEG	NEG	NEG	POS	POS	NEG	NEG	NEG	NEG
UKD-0508483	SCCmec I (COL)	CC5	NEG	NEG	NEG	NEG	NEG	NEG	NEG	NEG	NEG	NEG	NEG	NEG	NEG	NEG	NEG	NEG	POS	POS	NEG	NEG	NEG	NEG
UKD-0519001	SCCmec I (COL)	CC5	NEG	NEG	NEG	NEG	NEG	NEG	NEG	NEG	NEG	NEG	NEG	NEG	NEG	NEG	NEG	NEG	POS	POS	NEG	NEG	NEG	NEG
UKD-0701876	SCCmec I (COL)	CC5	NEG	NEG	NEG	NEG	NEG	NEG	NEG	NEG	NEG	NEG	NEG	NEG	NEG	NEG	NEG	NEG	POS	POS	NEG	NEG	NEG	NEG
UKD-0708865	SCCmec I (COL)	CC5	NEG	NEG	NEG	NEG	NEG	NEG	NEG	NEG	NEG	NEG	NEG	NEG	NEG	NEG	NEG	NEG	POS	POS	NEG	NEG	NEG	NEG
UKD-1001782	SCCmec I (COL)	CC5	NEG	NEG	NEG	NEG	NEG	NEG	NEG	NEG	NEG	NEG	NEG	NEG	NEG	NEG	NEG	NEG	POS	POS	NEG	NEG	NEG	NEG
Prediction for PSP1996, GenBank ANHU	SCCmec I (PSP1996)	CC8	NEG	NEG	NEG	NEG	NEG	NEG	NEG	NEG	NEG	NEG	NEG	NEG	NEG	NEG	NEG	NEG	POS	POS	NEG	NEG	NEG	NEG
UKD-0261727	SCCmec I (PSP1996)	CC8	NEG	NEG	NEG	NEG	NEG	NEG	NEG	NEG	NEG	NEG	NEG	NEG	NEG	NEG	NEG	NEG	POS	POS	NEG	NEG	NEG	NEG
VIRTUAL HYBRIDISATION for MRSA7 : VIRTUAL HYBRII	SCCmec I+fus+tirS (Geraldine Clone)	CC5	NEG	NEG	POS	POS	NEG	NEG	NEG	NEG	NEG	NEG	NEG	NEG	NEG	NEG	NEG	NEG	POS	POS	NEG	NEG	NEG	NEG
UKD-1221930	SCCmec I+fus+tirS (Geraldine Clone)	CC5	NEG	NEG	POS	POS	NEG	NEG	NEG	NEG	NEG	NEG	NEG	NEG	NEG	NEG	NEG	NEG	POS	POS	NEG	NEG	NEG	NEG
Prediction for N315, GenBank BA000018.3	SCCmec II (N315)	CC5	NEG	NEG	NEG	NEG	NEG	NEG	NEG	NEG	NEG	NEG	NEG	NEG	NEG	NEG	NEG	NEG	NEG	NEG	POS	POS	NEG	NEG
Reference strain N315	SCCmec II (N315)	CC5	NEG	NEG	NEG	NEG	NEG	NEG	NEG	NEG	NEG	NEG	NEG	NEG	NEG	NEG	NEG	NEG	NEG	NEG	POS	POS	NEG	NEG
Prediction for MRSA252, GenBank BX571856.1	SCCmec II (N315)	CC30	NEG	NEG	NEG	NEG	NEG	NEG	NEG	NEG	NEG	NEG	NEG	NEG	NEG	NEG	NEG	NEG	NEG	NEG	POS	POS	NEG	NEG
Reference strain Sanger 252	SCCmec II (N315)	CC30	NEG	NEG	NEG	NEG	NEG	NEG	NEG	NEG	NEG	NEG	NEG	NEG	NEG	NEG	NEG	NEG	NEG	NEG	POS	POS	NEG	NEG
UKD-0230792	SCCmec II (N315)	CC30	NEG	NEG	NEG	NEG	NEG	NEG	NEG	NEG	NEG	NEG	NEG	NEG	NEG	NEG	NEG	NEG	NEG	NEG	POS	POS	NEG	NEG
Prediction for Mu50, GenBank BA000017.4	SCCmec II (N315)	CC5	NEG	NEG	NEG	NEG	NEG	NEG	NEG	NEG	NEG	NEG	NEG	NEG	NEG	NEG	NEG	NEG	NEG	NEG	POS	POS	NEG	NEG
Reference strain MU50	SCCmec II (N315)	CC5	NEG	NEG	NEG	NEG	NEG	NEG	NEG	NEG	NEG	NEG	NEG	NEG	NEG	NEG	NEG	NEG	NEG	NEG	POS	POS	NEG	NEG
Prediction for JH1, GenBank CP000736.1	SCCmec II (JH1/JH9)	CC5	NEG	NEG	NEG	NEG	NEG	NEG	NEG	NEG	NEG	NEG	NEG	NEG	NEG	NEG	NEG	NEG	NEG	NEG	POS	POS	NEG	NEG
Prediction for JH9, GenBank CP000703.1	SCCmec II (JH1/JH9)	CC5	NEG	NEG	NEG	NEG	NEG	NEG	NEG	NEG	NEG	NEG	NEG	NEG	NEG	NEG	NEG	NEG	NEG	NEG	POS	POS	NEG	NEG
Reference strain Dublin-DSH_AR07.4_0237	SCCmec II (JH1/JH9)		NEG	NEG	NEG	NEG	NEG	NEG	NEG	NEG	NEG	NEG	NEG	NEG	NEG	NEG	NEG	NEG	NEG	NEG	POS	POS	NEG	NEG
Reference strain NARSA_382 / USA100	SCCmec II (JH1/JH9)	CC5	NEG	NEG	NEG	NEG	NEG	NEG	NEG	NEG	NEG	NEG	NEG	NEG	NEG	NEG	NEG	NEG	NEG	NEG	POS	POS	NEG	NEG
UKD-0046921	SCCmec II (JH1/JH9)	CC5	NEG	NEG	NEG	NEG	NEG	NEG	NEG	NEG	NEG	NEG	NEG	NEG	NEG	NEG	NEG	NEG	NEG	NEG	POS	POS	NEG	NEG
UKD-0333415	SCCmec II (JH1/JH9)	CC5	NEG	NEG	NEG	NEG	NEG	NEG	NEG	NEG	NEG	NEG	NEG	NEG	NEG	NEG	NEG	NEG	NEG	NEG	POS	POS	NEG	NEG
UKD-0341645	SCCmec II (JH1/JH9)	CC5	NEG	NEG	NEG	NEG	NEG	NEG	NEG	NEG	NEG	NEG	NEG	NEG	NEG	NEG	NEG	NEG	NEG	NEG	POS	POS	NEG	NEG
UKD-0504626	SCCmec II (JH1/JH9)	CC5	NEG	NEG	NEG	NEG	NEG	NEG	NEG	NEG	NEG	NEG	NEG	NEG	NEG	NEG	NEG	NEG	NEG	NEG	POS	POS	NEG	NEG
UKD-0507581	SCCmec II (JH1/JH9)	CC5	NEG	NEG	NEG	NEG	NEG	NEG	NEG	NEG	NEG	NEG	NEG	NEG	NEG	NEG	NEG	NEG	NEG	NEG	POS	POS	NEG	NEG
UKD-0507903	SCCmec II (JH1/JH9)	CC5	NEG	NEG	NEG	NEG	NEG	NEG	NEG	NEG	NEG	NEG	NEG	NEG	NEG	NEG	NEG	NEG	NEG	NEG	POS	POS	NEG	NEG
UKD-0700817	SCCmec II (JH1/JH9)	CC5	NEG	NEG	NEG	NEG	NEG	NEG	NEG	NEG	NEG	NEG	NEG	NEG	NEG	NEG	NEG	NEG	NEG	NEG	POS	POS	NEG	NEG
UKD-1014561	SCCmec II (JH1/JH9)	CC5	NEG	NEG	NEG	NEG	NEG	NEG	NEG	NEG	NEG	NEG	NEG	NEG	NEG	NEG	NEG	NEG	NEG	NEG	POS	POS	NEG	NEG
UKD-1037507	SCCmec II (JH1/JH9)	CC5	NEG	NEG	NEG	NEG	NEG	NEG	NEG	NEG	NEG	NEG	NEG	NEG	NEG	NEG	NEG	NEG	NEG	NEG	POS	POS	NEG	NEG
UKD-1038321	SCCmec II (JH1/JH9)	CC5	NEG	NEG	NEG	NEG	NEG	NEG	NEG	NEG	NEG	NEG	NEG	NEG	NEG	NEG	NEG	NEG	NEG	NEG	POS	POS	NEG	NEG
UKD-1111608	SCCmec II (JH1/JH9)	CC5	NEG	NEG	NEG	NEG	NEG	NEG	NEG	NEG	NEG	NEG	NEG	NEG	NEG	NEG	NEG	NEG	NEG	NEG	POS	POS	NEG	NEG
UKD-12753135	SCCmec II (JH1/JH9)	CC5	NEG	NEG	NEG	NEG	NEG	NEG	NEG	NEG	NEG	NEG	NEG	NEG	NEG	NEG	NEG	NEG	NEG	NEG	POS	POS	NEG	NEG
UKD-1312816	SCCmec II (JH1/JH9)	CC5	NEG	NEG	NEG	NEG	NEG	NEG	NEG	NEG	NEG	NEG	NEG	NEG	NEG	NEG	NEG	NEG	NEG	NEG	POS	POS	NEG	NEG
UKD-1401623	SCCmec II (JH1/JH9)	CC5	NEG	NEG	NEG	NEG	NEG	NEG	NEG	NEG	NEG	NEG	NEG	NEG	NEG	NEG	NEG	NEG	NEG	NEG	POS	POS	NEG	NEG
UKD-15571943	SCCmec II+czrC+ccrA/B-4	CC5	NEG	NEG	NEG	NEG	NEG	NEG	NEG	NEG	NEG	NEG	NEG	NEG	NEG	NEG	NEG	NEG	NEG	NEG	POS	POS	NEG	NEG
UKD-15576888	SCCmec II+czrC+ccrA/B-4	CC5	NEG	NEG	NEG	NEG	NEG	NEG	NEG	NEG	NEG	NEG	NEG	NEG	NEG	NEG	NEG	NEG	NEG	NEG	POS	POS	NEG	NEG
UKD-14576461	SCCmec II+speG+czrC+ccrA/B-4	CC5	NEG	NEG	NEG	NEG	NEG	NEG	NEG	NEG	NEG	NEG	NEG	NEG	NEG	NEG	NEG	POS	NEG	NEG	POS	POS	NEG	NEG
UKD-15581649	SCCmec II+speG+czrC+ccrA/B-4	CC5	NEG	NEG	NEG	NEG	NEG	NEG	NEG	NEG	NEG	NEG	NEG	NEG	NEG	NEG	NEG	POS	NEG	NEG	POS	POS	NEG	NEG
Prediction for CIGC345D, GenBank AHVO	SCCmec II A/B/D	CC8	NEG	NEG	NEG	NEG	NEG	NEG	NEG	NEG	NEG	NEG	NEG	NEG	NEG	NEG	NEG	POS	NEG	NEG	POS	POS	NEG	NEG
UKD-12K00171	SCCmec II A/B/D	CC8	NEG	NEG	NEG	NEG	NEG	NEG	NEG	NEG	NEG	NEG	NEG	NEG	NEG	NEG	NEG	POS	NEG	NEG	POS	POS	NEG	NEG
Prediction for JKD6008, GenBank CP002120.1	SCC [mec III+Cd] (JKD6008)	CC8 (ST239)	NEG	NEG	NEG	NEG	NEG	NEG	NEG	NEG	NEG	NEG	NEG	NEG	NEG	NEG	NEG	NEG	NEG	NEG	NEG	NEG	POS	POS
Prediction for JKD6009, GenBank ABSA	SCC [mec III+Cd] (JKD6008)	CC8 (ST239)	NEG	NEG	NEG	NEG	NEG	NEG	NEG	NEG	NEG	NEG	NEG	NEG	NEG	NEG	NEG	NEG	NEG	NEG	NEG	NEG	POS	POS
Reference strain ATCC_33592	SCC [mec III+Cd] (ATCC33592)	CC8 (ST239)	NEG	NEG	NEG	NEG	NEG	NEG	NEG	neg	NEG	NEG	NEG	NEG	NEG	NEG	NEG	NEG	NEG	POS	NEG	NEG	POS	POS
Prediction for PPUKM-775-2009, GenBank AMRE	SCC [mec III+Cd+ccrC] (PPUKM-775-2009)	CC8 (ST239)	NEG	NEG	NEG	NEG	NEG	NEG	NEG	NEG	NEG	NEG	NEG	NEG	NEG	NEG	NEG	NEG	NEG	NEG	NEG	NEG	POS	POS
Prediction for T0131, GenBank CP002643.1	SCC [mec III+Cd+ccrC] (T0131)	CC8 (ST239)	NEG	NEG	NEG	NEG	NEG	NEG	NEG	NEG	NEG	NEG	NEG	NEG	NEG	NEG	NEG	NEG	NEG	NEG	NEG	NEG	POS	POS
Prediction for XN108, GenBank CP007447.1	SCC [mec III+Cd+ccrC] (XN108)	CC8 (ST239)	NEG	NEG	NEG	NEG	NEG	NEG	NEG	NEG	NEG	NEG	NEG	NEG	NEG	NEG	NEG	NEG	NEG	NEG	NEG	NEG	POS	POS
Prediction for CN79, GenBank ANCJ	SCC [mec III+Cd+ccrC] (CN79)	CC8 (ST239)	NEG	NEG	NEG	NEG	NEG	NEG	NEG	NEG	NEG	NEG	NEG	NEG	NEG	NEG	NEG	NEG	NEG	NEG	NEG	NEG	POS	POS
Prediction for 16K, GenBank BABZ	SCC [mec III+Cd+ccrC] (CN79)	CC8 (ST239)	NEG	NEG	NEG	NEG	NEG	NEG	NEG	NEG	NEG	NEG	NEG	NEG	NEG	NEG	NEG	NEG	NEG	NEG	NEG	NEG	POS	POS
UKD-0835987	SCC [mec III+Cd+ccrC] (CN79)	CC8 (ST239)	NEG	NEG	NEG	NEG	NEG	NEG	NEG	NEG	NEG	NEG	NEG	NEG	NEG	NEG	NEG	NEG	NEG	POS	NEG	NEG	POS	POS
UKD-16581769	SCCmec III+Cd+ccrC	CC8 (ST239)	NEG	NEG	NEG	NEG	NEG	NEG	NEG	NEG	NEG	NEG	NEG	NEG	NEG	NEG	NEG	NEG	NEG	NEG	NEG	NEG	POS	POS
Prediction for Bmb9393, GenBank CP005288.1	SCCmec III+Cd/Hg+ccrC (Bmb9393)	CC8 (ST239)	NEG	NEG	NEG	NEG	NEG	NEG	NEG	NEG	NEG	NEG	NEG	NEG	NEG	NEG	NEG	NEG	NEG	NEG	NEG	NEG	POS	POS
UKD-15574263	SCCmec III+Cd/Hg+ccrC (Bmb9393)	CC8 (ST239)	NEG	NEG	NEG	NEG	NEG	NEG	NEG	NEG	NEG	NEG	NEG	NEG	NEG	NEG	NEG	NEG	NEG	POS	NEG	NEG	POS	POS
Prediction for SK1585, GenBank AYLT	SCCmec III+ccrC+Cd/Hg (SK1585)	CC8 (ST239)	NEG	NEG	NEG	NEG	NEG	NEG	NEG	NEG	NEG	NEG	NEG	NEG	NEG	NEG	NEG	NEG	NEG	NEG	NEG	NEG	POS	POS
UKD-105861	SCCmec III+ccrC+Cd/Hg (SK1585)	CC8 (ST239)	NEG	NEG	NEG	NEG	NEG	NEG	NEG	NEG	NEG	NEG	NEG	NEG	NEG	NEG	NEG	NEG	NEG	AMB	NEG	NEG	POS	POS
UKD-134572	SCCmec III+ccrC+Cd/Hg (SK1585)	CC8 (ST239)	NEG	NEG	NEG	NEG	NEG	NEG	NEG	neg	NEG	NEG	NEG	NEG	NEG	NEG	NEG	NEG	NEG	NEG	NEG	NEG	POS	POS
UKD-139143	SCCmec III+ccrC+Cd/Hg (SK1585)	CC8 (ST239)	NEG	NEG	NEG	NEG	NEG	NEG	NEG	NEG	NEG	NEG	NEG	NEG	NEG	NEG	NEG	NEG	NEG	NEG	NEG	NEG	POS	POS
Prediction for TW20, GenBank FN433596.1	SCC [mec III+Cd/Hg+ccrC] (TW20)	CC8 (ST239)	NEG	NEG	NEG	NEG	NEG	NEG	NEG	NEG	NEG	NEG	NEG	NEG	NEG	NEG	NEG	NEG	NEG	NEG	NEG	NEG	POS	POS
Prediction for ATCC BAA-39, GenBank AEEK	SCC [mec III+Cd/Hg+ccrC] (TW20)	CC8 (ST239)	NEG	NEG	NEG	NEG	NEG	NEG	NEG	NEG	NEG	NEG	NEG	NEG	NEG	NEG	NEG	NEG	NEG	NEG	NEG	NEG	POS	POS

Isolate ID	SCCmec type	Clonal complex	METHICILLIN RESISTANCE AND SCCmec TYPING																					
			Putative protein (from CC779-MRSA with novel SCCmec+ SCCfus)	CRISPR-associated endonuclease 1	SCC fusidic acid resistance locus			ACME-associated genes				ACME-associated oligopeptide permease, channel-forming protein					Alcohol dehydro-genase	Spermidine N-acetyltrans-ferase	cassette chromosome recombinase genes					
			D3JD07	cas1v2-M060171	fusC (Q6GD50)	tirS	yeeA (SCCmecIV/ fus)	arcA-SCC	arcB-SCC	arcC-SCC	arcD-SCC	opp3B	opp3B-FPR3757	opp3B-C427	opp3C-FPR3757	opp3C-C427	adhC	speG	ccrA-1	ccrB-1	ccrA-2	ccrB-2	ccrA-3	ccrB-3
Prediction for MW2, GenBank BA000033.2	SCCmec IVa (MW2)	CC1	NEG	NEG	NEG	NEG	NEG	NEG	NEG	NEG	NEG	NEG	NEG	NEG	NEG	NEG	NEG	NEG	NEG	NEG	POS	POS	NEG	NEG
Reference strain MW2	SCCmec IVa (MW2)	CC1	NEG	NEG	NEG	NEG	NEG	NEG	NEG	NEG	NEG	NEG	NEG	NEG	NEG	NEG	NEG	NEG	NEG	NEG	POS	POS	NEG	NEG
Reference strain CA05 (JCSC1968)	SCCmec IVa (MW2)	CC45	NEG	NEG	NEG	NEG	NEG	NEG	NEG	NEG	NEG	NEG	NEG	NEG	NEG	NEG	NEG	NEG	NEG	NEG	POS	POS	NEG	NEG
UKD-0127106	SCCmec IVa (MW2)	CC1	NEG	NEG	NEG	NEG	NEG	NEG	NEG	NEG	NEG	NEG	NEG	NEG	NEG	NEG	NEG	NEG	NEG	NEG	POS	POS	NEG	NEG
UKD-0701446	SCCmec IVa (MW2)	CC1	NEG	NEG	NEG	NEG	NEG	NEG	NEG	NEG	NEG	NEG	NEG	NEG	NEG	NEG	NEG	NEG	NEG	NEG	POS	POS	NEG	NEG
UKD-0947486	SCCmec IVa (MW2)	CC1	NEG	NEG	NEG	NEG	NEG	NEG	NEG	NEG	NEG	NEG	NEG	NEG	NEG	NEG	NEG	NEG	NEG	NEG	POS	POS	NEG	NEG
UKD-1031910	SCCmec IVa (MW2)	CC1	NEG	NEG	NEG	NEG	NEG	NEG	NEG	NEG	NEG	NEG	NEG	NEG	NEG	NEG	NEG	NEG	NEG	NEG	POS	POS	NEG	NEG
UKD-14571328	SCCmec IVa (MW2)	CC1	NEG	NEG	NEG	NEG	NEG	NEG	NEG	NEG	NEG	NEG	NEG	NEG	NEG	NEG	NEG	NEG	NEG	NEG	POS	POS	NEG	NEG
UKD-03003012	SCCmec IVa (MW2)	CC5	NEG	NEG	NEG	NEG	NEG	NEG	NEG	NEG	NEG	NEG	NEG	NEG	NEG	NEG	NEG	NEG	NEG	NEG	POS	POS	NEG	NEG
UKD-15582582	SCCmec IVa (MW2)	CC6	NEG	NEG	NEG	NEG	NEG	NEG	NEG	NEG	NEG	NEG	NEG	NEG	NEG	NEG	NEG	NEG	NEG	NEG	POS	POS	NEG	NEG
UKD-16574230	SCCmec IVa (MW2)	CC6	NEG	NEG	NEG	NEG	NEG	NEG	NEG	NEG	NEG	NEG	NEG	NEG	NEG	NEG	NEG	NEG	NEG	NEG	POS	POS	NEG	NEG
UKD-16582560	SCCmec IVa (MW2)	CC6	NEG	NEG	NEG	NEG	NEG	NEG	NEG	NEG	NEG	NEG	NEG	NEG	NEG	NEG	NEG	NEG	NEG	NEG	POS	POS	NEG	NEG
UKD-0802855	SCCmec IVa (MW2)	CC7	NEG	NEG	NEG	NEG	NEG	NEG	NEG	NEG	NEG	NEG	NEG	NEG	NEG	NEG	NEG	NEG	NEG	NEG	POS	POS	NEG	NEG
KHDN-10405258	SCCmec IVa (MW2)	CC8	NEG	NEG	NEG	NEG	NEG	NEG	NEG	NEG	NEG	NEG	NEG	NEG	NEG	NEG	NEG	NEG	NEG	NEG	POS	POS	NEG	NEG
UKD-15573497	SCCmec IVa (MW2)	CC22	NEG	NEG	NEG	NEG	NEG	NEG	NEG	NEG	NEG	NEG	NEG	NEG	NEG	NEG	NEG	NEG	NEG	NEG	POS	POS	NEG	NEG
UKD-0634729	SCCmec IVa (MW2)	CC30	NEG	NEG	NEG	NEG	NEG	NEG	NEG	NEG	NEG	NEG	NEG	NEG	NEG	NEG	NEG	NEG	NEG	NEG	POS	POS	NEG	AMB
UKD-1119771	SCCmec IVa (MW2)	CC30	NEG	NEG	NEG	NEG	NEG	NEG	NEG	NEG	NEG	NEG	NEG	NEG	NEG	NEG	NEG	NEG	NEG	NEG	POS	POS	NEG	NEG
KHDN-10411458	SCCmec IVa (MW2)	CC30	NEG	NEG	NEG	NEG	NEG	NEG	NEG	NEG	NEG	NEG	NEG	NEG	NEG	NEG	NEG	NEG	NEG	NEG	POS	POS	NEG	NEG
KHDN-398609	SCCmec IVa (MW2)	CC30	NEG	NEG	NEG	NEG	NEG	NEG	NEG	NEG	NEG	NEG	NEG	NEG	NEG	NEG	NEG	NEG	NEG	NEG	POS	POS	NEG	NEG
KHDN-01081998	SCCmec IVa (MW2)	CC30	NEG	NEG	NEG	NEG	NEG	NEG	NEG	NEG	NEG	NEG	NEG	NEG	NEG	NEG	NEG	NEG	NEG	NEG	POS	POS	NEG	NEG
KHDN-10399223	SCCmec IVa (MW2)	CC30	NEG	NEG	NEG	NEG	NEG	NEG	NEG	NEG	NEG	NEG	NEG	NEG	NEG	NEG	NEG	NEG	NEG	NEG	POS	POS	NEG	NEG
KHDN-W261158	SCCmec IVa (MW2)	CC30	NEG	AMB	NEG	NEG	NEG	NEG	NEG	NEG	NEG	NEG	NEG	NEG	NEG	NEG	NEG	NEG	NEG	NEG	POS	POS	NEG	NEG
UKD-0002046	SCCmec IVa (MW2)	CC45 [agr I]	NEG	NEG	NEG	NEG	NEG	NEG	NEG	NEG	NEG	NEG	NEG	NEG	NEG	NEG	NEG	NEG	NEG	NEG	POS	POS	NEG	NEG
UKD-0029618	SCCmec IVa (MW2)	CC45 [agr I]	NEG	NEG	NEG	NEG	NEG	NEG	NEG	NEG	NEG	NEG	NEG	NEG	NEG	NEG	NEG	NEG	NEG	NEG	POS	POS	NEG	NEG
UKD-0043085	SCCmec IVa (MW2)	CC45 [agr I]	NEG	NEG	NEG	NEG	NEG	NEG	NEG	NEG	NEG	NEG	NEG	NEG	NEG	NEG	NEG	NEG	NEG	NEG	POS	POS	NEG	NEG
UKD-0047053	SCCmec IVa (MW2)	CC45 [agr I]	NEG	NEG	NEG	NEG	NEG	NEG	NEG	NEG	NEG	NEG	NEG	NEG	NEG	NEG	NEG	NEG	NEG	NEG	POS	POS	NEG	NEG
UKD-0047429	SCCmec IVa (MW2)	CC45 [agr I]	NEG	NEG	NEG	NEG	NEG	NEG	NEG	NEG	NEG	NEG	NEG	NEG	NEG	NEG	NEG	NEG	NEG	NEG	POS	POS	NEG	NEG
UKD-0112465	SCCmec IVa (MW2)	CC45 [agr I]	NEG	NEG	NEG	NEG	NEG	NEG	NEG	NEG	NEG	NEG	NEG	NEG	NEG	NEG	NEG	NEG	NEG	NEG	POS	POS	NEG	NEG
UKD-0103483	SCCmec IVa (MW2)	CC45 [agr I]	NEG	NEG	NEG	NEG	NEG	NEG	NEG	NEG	NEG	NEG	NEG	NEG	NEG	NEG	NEG	NEG	NEG	NEG	POS	POS	NEG	NEG
UKD-0305014	SCCmec IVa (MW2)	CC45 [agr I]	NEG	NEG	NEG	NEG	NEG	NEG	NEG	NEG	NEG	NEG	NEG	NEG	NEG	NEG	NEG	NEG	NEG	NEG	POS	POS	NEG	NEG
UKD-0401119	SCCmec IVa (MW2)	CC45 [agr I]	NEG	NEG	NEG	NEG	NEG	NEG	NEG	NEG	NEG	NEG	NEG	NEG	NEG	NEG	NEG	NEG	NEG	NEG	POS	POS	NEG	NEG
UKD-0537969	SCCmec IVa (MW2)	CC45 [agr I]	NEG	NEG	NEG	NEG	NEG	NEG	NEG	NEG	NEG	NEG	NEG	NEG	NEG	NEG	NEG	NEG	NEG	NEG	POS	POS	NEG	NEG
UKD-0703373	SCCmec IVa (MW2)	CC45 [agr I]	NEG	NEG	NEG	NEG	NEG	NEG	NEG	NEG	NEG	NEG	NEG	NEG	NEG	NEG	NEG	NEG	NEG	NEG	POS	POS	NEG	NEG
UKD-0813653	SCCmec IVa (MW2)	CC45 [agr I]	NEG	NEG	NEG	NEG	NEG	NEG	NEG	NEG	NEG	NEG	NEG	NEG	NEG	NEG	NEG	NEG	NEG	NEG	POS	POS	NEG	NEG
UKD-0839175	SCCmec IVa (MW2)	CC45 [agr I]	NEG	NEG	NEG	NEG	NEG	NEG	NEG	NEG	NEG	NEG	NEG	NEG	NEG	NEG	NEG	NEG	NEG	NEG	POS	POS	NEG	NEG
UKD-1108422	SCCmec IVa (MW2)	CC45 [agr I]	NEG	NEG	NEG	NEG	NEG	NEG	NEG	NEG	NEG	NEG	NEG	NEG	NEG	NEG	NEG	NEG	NEG	NEG	POS	POS	NEG	NEG
UKD-1380645	SCCmec IVa (MW2)	CC45 [agr I]	NEG	NEG	NEG	NEG	NEG	NEG	NEG	NEG	NEG	NEG	NEG	NEG	NEG	NEG	NEG	NEG	NEG	NEG	POS	POS	NEG	NEG
UKD-148016	SCCmec IVa (MW2)	CC45 [agr I]	NEG	NEG	NEG	NEG	NEG	NEG	NEG	NEG	NEG	NEG	NEG	NEG	NEG	NEG	NEG	NEG	NEG	NEG	POS	POS	NEG	NEG
UKD-16571773	SCCmec IVa (MW2)	CC45 [agr I]	NEG	NEG	NEG	NEG	NEG	NEG	NEG	NEG	NEG	NEG	NEG	NEG	NEG	NEG	NEG	NEG	NEG	NEG	POS	POS	NEG	NEG
KHDN-10407738	SCCmec IVa (MW2)	CC45 [ST617]	NEG	NEG	NEG	NEG	NEG	NEG	NEG	NEG	NEG	NEG	NEG	NEG	NEG	NEG	NEG	NEG	NEG	NEG	POS	POS	NEG	NEG
UKD-0853670	SCCmec IVa (MW2)	CC88	NEG	NEG	NEG	NEG	NEG	NEG	NEG	NEG	NEG	NEG	NEG	NEG	NEG	NEG	NEG	NEG	NEG	NEG	POS	POS	NEG	NEG
UKD-1417352	SCCmec IVa (MW2)	CC88	NEG	NEG	NEG	NEG	NEG	NEG	NEG	NEG	NEG	NEG	NEG	NEG	NEG	NEG	NEG	NEG	NEG	NEG	POS	POS	NEG	NEG
UKD-15571283	SCCmec IVa (MW2)	CC88	NEG	NEG	NEG	NEG	NEG	NEG	NEG	NEG	NEG	NEG	NEG	NEG	NEG	NEG	NEG	NEG	NEG	NEG	POS	POS	NEG	NEG
KHDN-10406719	SCCmec IVa (MW2)	ST93	NEG	NEG	NEG	NEG	NEG	NEG	NEG	NEG	NEG	NEG	NEG	NEG	NEG	NEG	NEG	NEG	NEG	NEG	POS	POS	NEG	NEG
KHDN-0060294	SCCmec IVa (MW2)	ST93	NEG	NEG	NEG	NEG	NEG	NEG	NEG	NEG	NEG	NEG	NEG	NEG	NEG	NEG	NEG	NEG	NEG	NEG	POS	POS	NEG	NEG
UKD-0734190	SCCmec IVa (MW2)	CC97	NEG	NEG	NEG	NEG	NEG	NEG	NEG	NEG	NEG	NEG	NEG	NEG	NEG	NEG	NEG	NEG	NEG	NEG	POS	POS	NEG	NEG
UKD-13006983	SCCmec IVa (MW2)	CC97	NEG	NEG	NEG	NEG	NEG	NEG	NEG	NEG	NEG	NEG	NEG	NEG	NEG	NEG	NEG	NEG	NEG	NEG	POS	POS	NEG	NEG
Prediction for H131520133	SCCmec IVa (H131520133)	CC152	NEG	NEG	NEG	NEG	NEG	NEG	NEG	NEG	NEG	NEG	NEG	NEG	NEG	NEG	NEG	NEG	NEG	NEG	POS	POS	NEG	NEG
KHDN-325892	SCCmec IVa (H131520133)	CC30	NEG	NEG	NEG	NEG	NEG	NEG	NEG	NEG	NEG	NEG	NEG	NEG	NEG	NEG	NEG	NEG	NEG	NEG	POS	POS	NEG	NEG
UKD-15580207	SCCmec IVa (H131520133)	CC88	NEG	NEG	NEG	NEG	NEG	NEG	NEG	NEG	NEG	NEG	NEG	NEG	NEG	NEG	NEG	NEG	NEG	NEG	POS	POS	NEG	NEG
UKD-0521159	SCCmec Iva	CC8	NEG	NEG	NEG	NEG	NEG	NEG	NEG	NEG	NEG	NEG	NEG	NEG	NEG	NEG	NEG	NEG	NEG	NEG	POS	POS	NEG	NEG
Prediction for CMFT503 : HF569113.1	SCCmec IVa (CMFT503)		NEG	NEG	NEG	NEG	NEG	NEG	NEG	NEG	NEG	NEG	NEG	NEG	NEG	NEG	NEG	NEG	NEG	NEG	POS	POS	NEG	NEG
Prediction for 21343, GenBank AHKV	SCCmec IVa (CMFT503)	CC88	NEG	NEG	NEG	NEG	NEG	NEG	NEG	NEG	NEG	NEG	NEG	NEG	NEG	NEG	NEG	NEG	NEG	NEG	POS	POS	NEG	NEG
UKD-1420740	SCCmec IVa (CMFT503)	CC22	NEG	NEG	NEG	NEG	NEG	NEG	NEG	NEG	NEG	NEG	NEG	NEG	NEG	NEG	NEG	NEG	NEG	NEG	POS	POS	NEG	NEG
UKD-15575124	SCCmec IVa (CMFT503)	CC22	NEG	NEG	NEG	NEG	NEG	NEG	NEG	NEG	NEG	NEG	NEG	NEG	NEG	NEG	NEG	NEG	NEG	NEG	POS	POS	NEG	NEG
UKD-16584947	SCCmec IVa (CMFT503)	CC22	NEG	NEG	NEG	NEG	NEG	NEG	NEG	NEG	NEG	NEG	NEG	NEG	NEG	NEG	NEG	NEG	NEG	NEG	POS	POS	NEG	NEG
UKD-16575077	SCCmec IVa (CMFT503)	CC88	NEG	NEG	NEG	NEG	NEG	NEG	NEG	NEG	NEG	NEG	NEG	NEG	NEG	NEG	NEG	NEG	NEG	NEG	POS	POS	NEG	NEG
Prediction for TCH1516, GenBank CP000730.1	SCCmec IVa+ACME1+Cu (USA300)	CC8	NEG	NEG	NEG	NEG	NEG	POS	POS	POS	POS	POS	POS	AMB	POS	NEG	POS	POS	NEG	NEG	POS	POS	NEG	NEG
Reference strain USA300-TCH1516	SCCmec IVa+ACME1+Cu (USA300)	CC8	NEG	NEG	NEG	NEG	NEG	POS	POS	POS	POS	POS	POS	AMB	POS	NEG	POS	POS	NEG	NEG	POS	POS	NEG	NEG
Prediction for FPR3757, GenBank CP000255.1	SCCmec IVa+ACME1+Cu (USA300)	CC8	NEG	NEG	NEG	NEG	NEG	POS	POS	POS	POS	POS	POS	AMB	POS	NEG	POS	POS	NEG	NEG	POS	POS	NEG	NEG
UKD-0516386	SCCmec IVa+ACME1+Cu (USA300)	CC8	NEG	NEG	NEG	NEG	NEG	POS	POS	POS	POS	POS	POS	AMB	POS	NEG	POS	POS	NEG	NEG	POS	POS	NEG	NEG
UKD-0815773	SCCmec IVa+ACME1+Cu (USA300)	CC8	NEG	NEG	NEG	NEG	NEG	POS	POS	POS	POS	POS	POS	AMB	POS	NEG	POS	POS	NEG	NEG	POS	POS	NEG	POS
UKD-1414654	SCCmec IVa+ACME1+Cu (USA300)	CC8	NEG	NEG	NEG	NEG	NEG	POS	POS	POS	POS	POS	POS	AMB	POS	NEG	POS	POS	NEG	NEG	POS	POS	NEG	NEG
UKD-0408036	SCCmec IVa+ACME2	CC45 [agr I]	NEG	NEG	NEG	NEG	NEG	POS	POS	POS	POS	NEG	NEG	NEG	NEG	NEG	NEG	NEG	NEG	NEG	POS	POS	NEG	NEG
UKD-0044035	SCCmec IVa+ccrC (Hannover)	CC8	NEG	NEG	NEG	NEG	NEG	NEG	NEG	NEG	NEG	NEG	NEG	NEG	NEG	NEG	NEG	NEG	NEG	NEG	POS	POS	NEG	NEG
UKD-0136287	SCCmec IVa+ccrC (Hannover)	CC8	NEG	NEG	NEG	NEG	NEG	NEG	NEG	NEG	NEG	NEG	NEG	NEG	NEG	NEG	NEG	NEG	NEG	NEG	POS	POS	NEG	NEG

Isolate ID	SCCmec type	Clonal complex	METHICILLIN RESISTANCE AND SCCmec TYPING																					
			Putative protein (from CC779-MRSA with novel SCCmec+ SCCfus)	CRISPR-associated endonuclease 1	SCC fusidic acid resistance locus			ACME-associated genes				ACME-associated oligopeptide permease, channel-forming protein					Alcohol dehydro-genase	Spermidine N-acetyltrans-ferase	cassette chromosome recombinase genes					
			D3JD07	cas1v2-M060171	fusC (Q6GD50)	tirS	yeeA (SCCmecIV/ fus)	arcA-SCC	arcB-SCC	arcC-SCC	arcD-SCC	opp3B	opp3B-FPR3757	opp3B-C427	opp3C-FPR3757	opp3C-C427	adhC	speG	ccrA-1	ccrB-1	ccrA-2	ccrB-2	ccrA-3	ccrB-3
Prediction for JCSC1978, GenBank AB063173.1	SCCmec IVb/d/i (JCSC1978/6668/4469)		NEG	NEG	NEG	NEG	NEG	NEG	NEG	NEG	NEG	NEG	NEG	NEG	NEG	NEG	NEG	NEG	NEG	NEG	POS	POS	NEG	NEG
Reference strain JCSC1978 (8/6-3P)	SCCmec IVb/d/i (JCSC1978/6668/4469)	CC8	NEG	NEG	NEG	NEG	NEG	NEG	NEG	NEG	NEG	NEG	NEG	NEG	NEG	NEG	NEG	NEG	NEG	NEG	POS	POS	NEG	NEG
Prediction for JCSC6668, GenBank AB425823.1	SCCmec IVb/d/i (JCSC1978/6668/4469)		NEG	NEG	NEG	NEG	NEG	NEG	NEG	NEG	NEG	NEG	NEG	NEG	NEG	NEG	NEG	NEG	NEG	NEG	POS	POS	NEG	NEG
Reference strain JCSC4469	SCCmec IVb/d/i (JCSC1978/6668/4469)	CC5	NEG	NEG	NEG	NEG	NEG	NEG	NEG	NEG	NEG	NEG	NEG	NEG	NEG	NEG	NEG	NEG	NEG	NEG	POS	POS	NEG	NEG
UKD-1103429	SCCmec IVb/d/i (JCSC1978/6668/4469)	CC7	NEG	NEG	NEG	NEG	NEG	NEG	NEG	NEG	NEG	NEG	NEG	NEG	NEG	NEG	NEG	NEG	NEG	NEG	POS	POS	NEG	NEG
UKD-1306141	SCCmec IVb/d/i (JCSC1978/6668/4469)	CC7	NEG	NEG	NEG	NEG	NEG	NEG	NEG	NEG	NEG	NEG	NEG	NEG	NEG	NEG	NEG	NEG	NEG	NEG	POS	POS	NEG	NEG
UKD-0500089	SCCmec IVb/d/i (JCSC1978/6668/4469)	CC5	NEG	NEG	NEG	NEG	NEG	NEG	NEG	NEG	NEG	NEG	NEG	NEG	NEG	NEG	NEG	NEG	NEG	NEG	POS	POS	NEG	NEG
UKD-0501416	SCCmec IVb/d/i (JCSC1978/6668/4469)	CC5	NEG	NEG	NEG	NEG	NEG	NEG	NEG	NEG	NEG	NEG	NEG	NEG	NEG	NEG	NEG	NEG	NEG	NEG	POS	POS	NEG	NEG
UKD-0934574	SCCmec IVb/d/i (JCSC1978/6668/4469)	CC5	NEG	NEG	NEG	NEG	NEG	NEG	NEG	NEG	NEG	NEG	NEG	NEG	NEG	NEG	NEG	NEG	NEG	NEG	POS	POS	NEG	NEG
UKD-0746779	SCCmec IVb/d/i (JCSC1978/6668/4469)	CC45 [agr I]	NEG	NEG	NEG	NEG	NEG	NEG	NEG	NEG	NEG	NEG	NEG	NEG	NEG	NEG	NEG	NEG	NEG	NEG	POS	POS	NEG	AMB
UKD-0812518	SCCmec IVb/d/i (JCSC1978/6668/4469)	CC45 [agr I]	NEG	NEG	NEG	NEG	NEG	NEG	NEG	NEG	NEG	NEG	NEG	NEG	NEG	NEG	NEG	NEG	NEG	NEG	POS	POS	NEG	NEG
Prediction for 21209, GenBank AGRP	SCCmec IVb/d/i (21209)	CC8	NEG	NEG	NEG	NEG	NEG	NEG	NEG	NEG	NEG	NEG	NEG	NEG	NEG	NEG	NEG	NEG	NEG	NEG	POS	POS	NEG	NEG
UKD-0233579	SCCmec IVb/d/i (21209)	CC8	NEG	NEG	NEG	NEG	NEG	NEG	AMB	NEG	NEG	NEG	NEG	NEG	NEG	NEG	NEG	NEG	NEG	NEG	POS	POS	NEG	NEG
UKD-0504920	SCCmec IVb/d/i (21209)	CC8	NEG	NEG	NEG	NEG	NEG	NEG	NEG	NEG	NEG	NEG	NEG	NEG	NEG	NEG	NEG	NEG	NEG	NEG	POS	POS	NEG	NEG
UKD-0725999	SCCmecIV b/d/i+Cu	CC30	NEG	NEG	NEG	NEG	NEG	NEG	NEG	NEG	NEG	NEG	NEG	NEG	NEG	NEG	NEG	NEG	NEG	NEG	NEG	POS	NEG	NEG
Prediction for IS-105, GenBank AHLR	SCCmec IVc (IS-105)	CC22	NEG	NEG	NEG	NEG	NEG	NEG	NEG	NEG	NEG	NEG	NEG	NEG	NEG	NEG	NEG	NEG	NEG	NEG	POS	POS	NEG	NEG
UKD-0402421	SCCmec IVc (IS-105), merA/B+	CC5	NEG	NEG	NEG	NEG	NEG	NEG	NEG	NEG	NEG	NEG	NEG	NEG	NEG	NEG	NEG	NEG	NEG	NEG	POS	POS	NEG	NEG
UKD-0731319	SCCmec IVc (IS-105), merA/B+	CC5	NEG	NEG	NEG	NEG	NEG	NEG	NEG	NEG	NEG	NEG	NEG	NEG	NEG	NEG	NEG	NEG	NEG	NEG	POS	POS	NEG	NEG
UKD-0733137	SCCmec IVc (IS-105)	CC8	NEG	NEG	NEG	NEG	NEG	NEG	NEG	NEG	NEG	NEG	NEG	NEG	NEG	NEG	NEG	NEG	NEG	NEG	POS	POS	NEG	NEG
UKD-0305690	SCCmec IVc (IS-105)	CC22	NEG	NEG	NEG	NEG	NEG	NEG	NEG	NEG	NEG	NEG	NEG	NEG	NEG	NEG	NEG	NEG	NEG	NEG	POS	POS	NEG	NEG
UKD-1424706	SCCmec IVc (IS-105)	CC22	NEG	NEG	NEG	NEG	NEG	NEG	NEG	NEG	NEG	NEG	NEG	NEG	NEG	NEG	NEG	NEG	NEG	NEG	POS	POS	NEG	NEG
Prediction for TCH60, GenBank CP002110.1	SCCmec IVc (TCH60)	CC30	NEG	NEG	NEG	NEG	NEG	NEG	NEG	NEG	NEG	NEG	NEG	NEG	NEG	NEG	NEG	NEG	NEG	NEG	POS	POS	NEG	NEG
Reference strain JCSC4788	SCCmec IVc (TCH60)	CC8	NEG	NEG	NEG	NEG	NEG	NEG	NEG	NEG	NEG	NEG	NEG	NEG	NEG	NEG	NEG	NEG	NEG	NEG	POS	POS	NEG	NEG
UKD-1318318	SCCmec IVc (TCH60)	CC5	NEG	NEG	NEG	NEG	NEG	NEG	NEG	NEG	NEG	NEG	NEG	NEG	NEG	NEG	NEG	NEG	NEG	NEG	POS	POS	NEG	NEG
UKD-14580050	SCCmec IVc (TCH60)	CC5	NEG	NEG	NEG	NEG	NEG	NEG	NEG	NEG	NEG	NEG	NEG	NEG	NEG	NEG	NEG	NEG	NEG	NEG	POS	POS	NEG	NEG
UKD-08F01314	SCCmec IVc (TCH60)	CC8	NEG	NEG	NEG	NEG	NEG	NEG	NEG	NEG	NEG	NEG	NEG	NEG	NEG	NEG	NEG	NEG	NEG	NEG	POS	POS	NEG	NEG
KHDN-50361518	SCCmec IVc (TCH60)	CC8	NEG	NEG	NEG	NEG	NEG	NEG	NEG	NEG	NEG	NEG	NEG	NEG	NEG	NEG	NEG	NEG	NEG	NEG	POS	POS	NEG	NEG
UKD-0307912	SCCmec IVc (TCH60)	CC22	NEG	NEG	NEG	NEG	NEG	NEG	NEG	NEG	NEG	NEG	NEG	NEG	NEG	NEG	NEG	NEG	NEG	AMB	POS	POS	NEG	NEG
UKD-0220390	SCCmec IVc (TCH60)	CC45 [agr I]	NEG	NEG	NEG	NEG	NEG	NEG	NEG	NEG	NEG	NEG	NEG	NEG	NEG	NEG	NEG	NEG	NEG	NEG	POS	POS	NEG	NEG
UKD-0311106	SCCmec IVc (TCH60)	CC45 [agr I]	NEG	NEG	NEG	NEG	NEG	NEG	NEG	NEG	NEG	NEG	NEG	NEG	NEG	NEG	NEG	NEG	NEG	NEG	POS	POS	NEG	NEG
UKD-0719855	SCCmec IVc (TCH60)	CC45 [agr I]	NEG	NEG	NEG	NEG	NEG	NEG	NEG	NEG	NEG	NEG	NEG	NEG	NEG	NEG	NEG	NEG	NEG	NEG	POS	POS	NEG	NEG
UKD-0422079	SCCmec IVc (TCH60)	CC45 [agr I]	NEG	NEG	NEG	NEG	NEG	NEG	NEG	NEG	NEG	NEG	NEG	NEG	NEG	NEG	NEG	NEG	NEG	NEG	POS	POS	NEG	NEG
UKD-0409235	SCCmec IVc (TCH60)	CC80	NEG	NEG	NEG	NEG	NEG	NEG	NEG	NEG	NEG	NEG	NEG	NEG	NEG	NEG	NEG	NEG	NEG	NEG	POS	POS	NEG	NEG
UKD-0409807	SCCmec IVc (TCH60)	CC80	NEG	NEG	NEG	NEG	NEG	NEG	NEG	NEG	NEG	NEG	NEG	NEG	NEG	NEG	NEG	NEG	NEG	NEG	POS	POS	NEG	NEG
UKD-0416073	SCCmec IVc (TCH60)	CC80	NEG	NEG	NEG	NEG	NEG	NEG	NEG	neg	NEG	NEG	NEG	NEG	NEG	NEG	NEG	NEG	NEG	NEG	POS	POS	NEG	NEG
UKD-0431728	SCCmec IVc (TCH60)	CC80	NEG	NEG	NEG	NEG	NEG	NEG	NEG	NEG	NEG	NEG	NEG	NEG	NEG	NEG	NEG	NEG	NEG	NEG	POS	POS	NEG	NEG
UKD-1001508	SCCmec IVc (TCH60)	CC80	NEG	NEG	NEG	NEG	NEG	NEG	NEG	NEG	NEG	NEG	NEG	NEG	NEG	NEG	NEG	NEG	NEG	NEG	POS	POS	NEG	NEG
UKD-1010412	SCCmec IVc (TCH60)	CC80	NEG	NEG	NEG	NEG	NEG	NEG	NEG	NEG	AMB	NEG	NEG	NEG	NEG	NEG	NEG	NEG	NEG	NEG	POS	POS	NEG	NEG
UKD-12753005	SCCmec IVc (TCH60)	CC80	NEG	NEG	NEG	NEG	NEG	NEG	NEG	NEG	NEG	NEG	NEG	NEG	NEG	NEG	NEG	NEG	NEG	NEG	POS	POS	NEG	NEG
UKD-15574809	SCCmec IVc (TCH60)	CC80	NEG	NEG	NEG	NEG	NEG	NEG	NEG	NEG	NEG	NEG	NEG	NEG	NEG	NEG	NEG	NEG	NEG	NEG	POS	POS	NEG	NEG
KHDN-16120291	SCCmec IVc (TCH60)	CC88	NEG	NEG	NEG	NEG	NEG	NEG	NEG	NEG	NEG	NEG	NEG	NEG	NEG	NEG	NEG	NEG	NEG	NEG	POS	POS	NEG	NEG
Prediction for WW2703/97, GenBank ACSW	SCCmec IVc (WW2703/97)	CC30	NEG	NEG	NEG	NEG	NEG	NEG	NEG	NEG	NEG	NEG	NEG	NEG	NEG	NEG	NEG	NEG	NEG	NEG	POS	POS	NEG	NEG
UKD-14580088	SCCmec IVc (WW2703/97)	CC398	NEG	NEG	NEG	NEG	NEG	NEG	NEG	NEG	NEG	NEG	NEG	NEG	NEG	NEG	NEG	NEG	NEG	NEG	POS	POS	NEG	NEG
Prediction for SA_ST125_MupR, GenBank ASTH	SCCmec IVc+speG+ccrAB4 (SA_ST125)	CC5	NEG	NEG	NEG	NEG	NEG	NEG	NEG	NEG	NEG	NEG	NEG	NEG	NEG	NEG	NEG	POS	NEG	NEG	POS	POS	NEG	NEG
UKD-0439909	SCCmec IVc+speG+ccrAB4 (SA_ST125)	CC5	NEG	NEG	NEG	NEG	NEG	NEG	NEG	NEG	NEG	NEG	NEG	NEG	NEG	NEG	NEG	POS	NEG	NEG	POS	POS	NEG	neg
Prediction for MRSA177, GenBank AECP	SCCmec IVc+Cu/Hg (MRSA177)		NEG	NEG	NEG	NEG	NEG	NEG	NEG	NEG	NEG	NEG	NEG	NEG	NEG	NEG	NEG	NEG	NEG	NEG	POS	POS	NEG	NEG
UKD-1279734	SCCmec IVc+Cu/Hg (MRSA177)	CC8	NEG	NEG	NEG	NEG	NEG	NEG	NEG	NEG	NEG	NEG	NEG	NEG	NEG	NEG	NEG	NEG	NEG	NEG	POS	POS	NEG	NEG
UKD-14580899	SCCmec IVc+Cu/Hg (MRSA177)	CC8	NEG	NEG	NEG	NEG	NEG	NEG	NEG	NEG	NEG	NEG	NEG	NEG	NEG	NEG	NEG	NEG	NEG	NEG	POS	POS	NEG	NEG
UKD-16002228	SCCmec IVc+Cu/Hg (MRSA177)	CC8	NEG	NEG	NEG	NEG	NEG	NEG	NEG	NEG	NEG	NEG	NEG	NEG	NEG	NEG	NEG	NEG	NEG	NEG	POS	POS	NEG	NEG
Prediction for HO 5096 0412, GenBank HE681097.1	SCCmec IVh/j (HO50960412/JCSC6670)	CC22	NEG	NEG	NEG	NEG	NEG	NEG	NEG	NEG	NEG	NEG	NEG	NEG	NEG	NEG	NEG	NEG	NEG	NEG	POS	POS	NEG	NEG
Prediction for JCSC6670, GenBank AB425824.1	SCCmec IVh/j (HO50960412/JCSC6670)		NEG	NEG	NEG	NEG	NEG	NEG	NEG	NEG	NEG	NEG	NEG	NEG	NEG	NEG	NEG	NEG	NEG	NEG	POS	POS	NEG	NEG
Reference strain Dublin_DSH_E1749	SCCmec IVh/j (HO50960412/JCSC6670)		NEG	NEG	NEG	NEG	NEG	NEG	NEG	NEG	NEG	NEG	NEG	NEG	NEG	NEG	NEG	NEG	NEG	NEG	POS	POS	NEG	NEG
UKD-14576248	SCCmec IVh/j (HO50960412/JCSC6670)	CC8	NEG	NEG	NEG	NEG	NEG	NEG	NEG	NEG	NEG	NEG	NEG	NEG	NEG	NEG	NEG	NEG	NEG	NEG	POS	POS	NEG	NEG
UKD-0120122	SCCmec IVh/j (HO50960412/JCSC6670)	CC22	NEG	NEG	NEG	NEG	NEG	NEG	NEG	NEG	NEG	NEG	NEG	NEG	NEG	NEG	NEG	NEG	NEG	NEG	POS	POS	NEG	NEG
UKD-0205319	SCCmec IVh/j (HO50960412/JCSC6670)	CC22	NEG	NEG	NEG	NEG	NEG	NEG	NEG	NEG	NEG	NEG	NEG	NEG	NEG	NEG	NEG	NEG	NEG	NEG	POS	POS	NEG	NEG
UKD-0221259	SCCmec IVh/j (HO50960412/JCSC6670)	CC22	NEG	NEG	NEG	NEG	NEG	NEG	NEG	NEG	NEG	NEG	NEG	NEG	NEG	NEG	NEG	NEG	NEG	NEG	POS	POS	NEG	NEG
UKD-0418654	SCCmec IVh/j (HO50960412/JCSC6670)	CC22	NEG	NEG	NEG	NEG	NEG	NEG	NEG	NEG	NEG	NEG	NEG	NEG	NEG	NEG	NEG	NEG	NEG	NEG	POS	POS	NEG	NEG
UKD-0429721	SCCmec IVh/j (HO50960412/JCSC6670)	CC22	NEG	NEG	NEG	NEG	NEG	NEG	NEG	NEG	NEG	NEG	NEG	NEG	NEG	NEG	NEG	NEG	NEG	NEG	POS	POS	NEG	NEG
UKD-1116194	SCCmec IVh/j (HO50960412/JCSC6670)	CC22	NEG	NEG	NEG	NEG	NEG	NEG	NEG	NEG	NEG	NEG	NEG	NEG	NEG	NEG	NEG	NEG	NEG	NEG	POS	POS	NEG	NEG
UKD-1122367	SCCmec IVh/j (HO50960412/JCSC6670)	CC22	NEG	NEG	NEG	NEG	NEG	NEG	NEG	NEG	NEG	NEG	NEG	NEG	NEG	NEG	NEG	NEG	NEG	NEG	POS	POS	NEG	NEG
UKD-14012840	SCCmec IVh/j (HO50960412/JCSC6670)	CC22	NEG	NEG	NEG	NEG	NEG	NEG	NEG	NEG	NEG	NEG	NEG	NEG	NEG	NEG	NEG	NEG	NEG	NEG	POS	POS	NEG	NEG
UKD-14579833	SCCmec IVh/j (HO50960412/JCSC6670)	CC22	NEG	NEG	NEG	NEG	NEG	NEG	NEG	NEG	NEG	NEG	NEG	NEG	NEG	NEG	NEG	NEG	NEG	NEG	POS	POS	NEG	NEG
UKD-15579198	SCCmec IVh/j (HO50960412/JCSC6670)	CC22	NEG	NEG	NEG	NEG	NEG	NEG	NEG	NEG	NEG	NEG	NEG	NEG	NEG	NEG	NEG	NEG	NEG	NEG	POS	POS	NEG	NEG
UKD-4052193	SCCmec IVh/j (HO50960412/JCSC6670)	CC22	NEG	NEG	NEG	NEG	NEG	NEG	NEG	NEG	NEG	NEG	NEG	NEG	NEG	NEG	NEG	NEG	NEG	NEG	POS	POS	NEG	NEG
UKD-124968	SCCmec IVh/j (HO50960412/JCSC6670)	CC22	NEG	NEG	NEG	NEG	NEG	NEG	NEG	NEG	NEG	NEG	NEG	NEG	NEG	NEG	NEG	NEG	NEG	NEG	POS	POS	NEG	NEG
UKD-14572546	SCCmec IVh/j (HO50960412/JCSC6670)	CC22	NEG	NEG	NEG	NEG	NEG	NEG	NEG	NEG	NEG	NEG	NEG	NEG	NEG	NEG	NEG	NEG	NEG	NEG	POS	POS	NEG	NEG
UKD-14582287	SCCmec IVh/j (HO50960412/JCSC6670)	CC22	NEG	NEG	NEG	NEG	NEG	NEG	NEG	NEG	NEG	NEG	NEG	NEG	NEG	NEG	NEG	NEG	NEG	NEG	POS	POS	AMB	NEG
UKD-14583376	SCCmec IVh/j (HO50960412/JCSC6670)	CC22	NEG	NEG	NEG	NEG	NEG	NEG	NEG	NEG	NEG	NEG	NEG	NEG	NEG	NEG	NEG	NEG	NEG	NEG	POS	POS	AMB	NEG
UKD-14583496	SCCmec IVh/j (HO50960412/JCSC6670)	CC22	NEG	NEG	NEG	NEG	NEG	NEG	NEG	NEG	NEG	NEG	NEG	NEG	NEG	NEG	NEG	NEG	NEG	NEG	POS	POS	POS	NEG
UKD-0013324	SCCmec IVh/j (HO50960412/JCSC6670)	CC22	NEG	NEG	NEG	NEG	NEG	NEG	NEG	NEG	NEG	NEG	NEG	NEG	NEG	NEG	NEG	NEG	NEG	NEG	POS	POS	NEG	NEG
UKD-0125329	SCCmec IVh/j (HO50960412/JCSC6670)	CC22	NEG	NEG	NEG	NEG	NEG	NEG	NEG	NEG	NEG	NEG	NEG	NEG	NEG	NEG	NEG	NEG	NEG	NEG	POS	POS	NEG	NEG
UKD-0215498	SCCmec IVh/j (HO50960412/JCSC6670)	CC22	NEG	NEG	NEG	NEG	NEG	NEG	NEG	NEG	NEG	NEG	NEG	NEG	NEG	NEG	NEG	NEG	NEG	NEG	POS	POS	NEG	NEG
Prediction for M08-0126, GenBank FR753166.1	SCCmec IVh/j+ACME2 (M08-0126)	CC22	NEG	NEG	NEG	NEG	NEG	POS	POS	POS	POS	NEG	NEG	NEG	NEG	NEG	NEG	NEG	NEG	NEG	POS	POS	NEG	NEG
UKD-1379483I	SCCmec IVh/j+ACME2 (M08-0126)	CC22	NEG	NEG	NEG	NEG	NEG	POS	POS	POS	POS	NEG	NEG	NEG	NEG	NEG	NEG	NEG	NEG	NEG	POS	POS	NEG	NEG
UKD-5150204	SCCmec IV+speG+Cu/czrC+ccrAB4	CC22	NEG	NEG	NEG	NEG	NEG	NEG	NEG	NEG	NEG	NEG	NEG	NEG	NEG	NEG	NEG	POS	NEG	NEG	POS	POS	NEG	NEG
UKD-15578423	SCCmec IV+speG+czrC+ccrAB4 var. 1	CC22	NEG	NEG	NEG	NEG	NEG	NEG	NEG	NEG	NEG	NEG	NEG	NEG	NEG	NEG	NEG	POS	NEG	NEG	POS	POS	NEG	NEG
UKD-16582870	SCCmec IV+speG+czrC+ccrAB4 var. 1	CC22	NEG	NEG	NEG	NEG	NEG	NEG	NEG	NEG	NEG	NEG	NEG	NEG	NEG	NEG	NEG	POS	NEG	NEG	POS	POS	NEG	NEG
UKD-15571940	SCCmec IV+speG+czrC+ccrAB4 var. 2	CC22	NEG	NEG	NEG	NEG	NEG	NEG	NEG	NEG	NEG	NEG	NEG	NEG	NEG	NEG	NEG	POS	NEG	NEG	POS	POS	NEG	NEG

Isolate ID	SCCmec type	Clonal complex	METHICILLIN RESISTANCE AND SCCmec TYPING																					
			Putative protein (from CC779-MRSA with novel SCCmec+ SCCfus)	CRISPR-associated endonuclease 1	SCC fusidic acid resistance locus			ACME-associated genes				ACME-associated oligopeptide permease, channel-forming protein					Alcohol dehydro-genase	Spermidine N-acetyltrans-ferase	cassette chromosome recombinase genes					
			D3JD07	cas1v2-M060171	fusC (Q6GD50)	tirS	yeeA (SCCmecIV/ fus)	arcA-SCC	arcB-SCC	arcC-SCC	arcD-SCC	opp3B	opp3B-FPR3757	opp3B-C427	opp3C-FPR3757	opp3C-C427	adhC	speG	ccrA-1	ccrB-1	ccrA-2	ccrB-2	ccrA-3	ccrB-3
Prediction for CN1, GenBank CP003979.1	SCCmec IVA (CN1)	CC8 (ST72)	NEG	NEG	NEG	NEG	NEG	NEG	NEG	NEG	NEG	NEG	NEG	NEG	NEG	NEG	NEG	NEG	NEG	NEG	POS	POS	NEG	NEG
UKD-0900871	SCCmec IVA (CN1)	CC8 (ST72)	NEG	NEG	NEG	NEG	NEG	NEG	NEG	NEG	NEG	NEG	NEG	NEG	NEG	NEG	NEG	NEG	NEG	NEG	POS	POS	NEG	NEG
Prediction for CMFT1723, GenBank HF569096.1	SCCmec V (Bengal Bay)		NEG	NEG	NEG	NEG	NEG	NEG	NEG	NEG	NEG	NEG	NEG	NEG	NEG	NEG	NEG	NEG	NEG	NEG	NEG	NEG	NEG	NEG
KHDN-10405134	SCCmec V (Bengal Bay)	CC1	NEG	NEG	NEG	NEG	NEG	NEG	NEG	NEG	NEG	NEG	NEG	NEG	NEG	NEG	NEG	NEG	NEG	NEG	NEG	NEG	NEG	NEG
UKD-0932405	SCCmec V (Bengal Bay)	CC1 (ST573/772)	NEG	NEG	NEG	NEG	NEG	NEG	NEG	NEG	NEG	NEG	NEG	NEG	NEG	NEG	NEG	NEG	NEG	NEG	NEG	NEG	NEG	NEG
UKD-1003510	SCCmec V (Bengal Bay)	CC1 (ST573/772)	NEG	NEG	NEG	NEG	NEG	NEG	NEG	NEG	NEG	NEG	NEG	NEG	NEG	NEG	NEG	NEG	NEG	NEG	NEG	NEG	NEG	NEG
UKD-1112420	SCCmec V (Bengal Bay)	CC1 (ST573/772)	NEG	NEG	NEG	NEG	NEG	NEG	NEG	NEG	NEG	NEG	NEG	NEG	NEG	NEG	NEG	NEG	NEG	NEG	NEG	NEG	NEG	NEG
UKD-1275832	SCCmec V (Bengal Bay)	CC1 (ST573/772)	NEG	NEG	NEG	NEG	NEG	NEG	NEG	NEG	NEG	NEG	NEG	NEG	NEG	NEG	NEG	NEG	NEG	NEG	NEG	NEG	NEG	NEG
UKD-13023811	SCCmec V (Bengal Bay)	CC1 (ST573/772)	NEG	NEG	NEG	NEG	NEG	NEG	NEG	NEG	NEG	NEG	NEG	NEG	NEG	NEG	NEG	NEG	NEG	NEG	NEG	NEG	NEG	NEG
UKD-14572163	SCCmec V	CC5	NEG	NEG	NEG	NEG	NEG	NEG	NEG	NEG	NEG	NEG	NEG	NEG	NEG	NEG	NEG	NEG	NEG	NEG	NEG	NEG	NEG	NEG
KHDN-10421133	SCCmec V+fus	CC97	NEG	NEG	POS	NEG	NEG	NEG	NEG	NEG	NEG	NEG	NEG	NEG	NEG	NEG	NEG	NEG	NEG	NEG	NEG	NEG	NEG	NEG
KHDN-21343541	SCCmec V+fus+tir+ccrAB1	CC1	NEG	NEG	POS	POS	NEG	NEG	NEG	NEG	NEG	NEG	NEG	NEG	NEG	NEG	NEG	NEG	POS	POS	NEG	NEG	NEG	AMB
KHDN-10416893	SCCmec V+czrC+speG+ccrAB4	CC22	NEG	NEG	NEG	NEG	NEG	NEG	NEG	NEG	NEG	NEG	NEG	NEG	NEG	NEG	NEG	POS	NEG	NEG	NEG	NEG	NEG	NEG
Prediction for GR1, GenBank AJLX	SCCmec VT (GR1)	CC361	NEG	NEG	NEG	NEG	NEG	NEG	NEG	NEG	NEG	NEG	NEG	NEG	NEG	NEG	NEG	NEG	NEG	NEG	NEG	NEG	NEG	NEG
UKD-0422872	SCCmec VT (GR1)	CC152	NEG	NEG	NEG	NEG	NEG	NEG	NEG	NEG	NEG	NEG	NEG	NEG	NEG	NEG	NEG	NEG	NEG	NEG	NEG	NEG	NEG	NEG
Prediction for PM1, GenBank BAFA	SCCmec VT (PM1)	CC59	NEG	NEG	NEG	NEG	NEG	NEG	NEG	NEG	NEG	NEG	NEG	NEG	NEG	NEG	NEG	NEG	NEG	NEG	NEG	NEG	NEG	NEG
KHDN-000871	SCCmec VT (PM1)	CC45 (agr I)	NEG	NEG	NEG	NEG	NEG	NEG	NEG	NEG	NEG	NEG	NEG	NEG	NEG	NEG	NEG	NEG	NEG	NEG	NEG	NEG	NEG	NEG
UKD-0725052	SCCmec VT (PM1)	CC59	NEG	NEG	NEG	NEG	NEG	NEG	NEG	NEG	NEG	NEG	NEG	NEG	NEG	NEG	NEG	NEG	NEG	NEG	NEG	NEG	NEG	NEG
UKD-1312138	SCCmec VT (PM1)	CC59	NEG	NEG	NEG	NEG	NEG	NEG	NEG	NEG	NEG	NEG	NEG	NEG	NEG	NEG	NEG	NEG	NEG	NEG	NEG	NEG	NEG	NEG
UKD-Z4728	SCCmec VT (PM1)	CC59	NEG	NEG	NEG	NEG	NEG	NEG	NEG	NEG	NEG	NEG	NEG	NEG	NEG	NEG	NEG	NEG	NEG	NEG	NEG	NEG	NEG	NEG
UKD-K0199	SCCmec VT unknown var.1	CC7	NEG	NEG	NEG	NEG	NEG	NEG	NEG	NEG	NEG	NEG	NEG	NEG	NEG	NEG	NEG	NEG	NEG	NEG	NEG	NEG	NEG	NEG
UKD-15584619	SCCmec VT unknown var.2	CC30	NEG	NEG	NEG	NEG	NEG	NEG	NEG	NEG	NEG	NEG	NEG	NEG	NEG	NEG	NEG	NEG	NEG	NEG	NEG	NEG	NEG	NEG
UKD-0712568	SCCmec VT unknown var.3	CC45 (agr I)	NEG	NEG	NEG	NEG	NEG	NEG	NEG	NEG	NEG	NEG	NEG	NEG	NEG	NEG	NEG	NEG	NEG	NEG	NEG	NEG	NEG	NEG
KHDN-10420296	SCCmec VT+ACME2	CC45 (agr I)	NEG	NEG	NEG	NEG	NEG	POS	POS	POS	POS	NEG	NEG	NEG	NEG	NEG	NEG	NEG	NEG	NEG	NEG	NEG	NEG	NEG
UKD-15577956	SCCmec VT+Cu/czrC+ccrA1	CC398	NEG	NEG	NEG	NEG	NEG	NEG	NEG	NEG	NEG	NEG	NEG	NEG	NEG	NEG	NEG	NEG	POS	POS	NEG	NEG	NEG	NEG
Prediction for S0385, GenBank AM990992	SCCmec VT+czrC (SO385)	CC398	NEG	NEG	NEG	NEG	NEG	NEG	NEG	NEG	NEG	NEG	NEG	NEG	NEG	NEG	NEG	NEG	NEG	NEG	NEG	NEG	NEG	NEG
UKD-0628560	SCCmec VT+czrC (SO385)	CC8	NEG	NEG	NEG	NEG	NEG	NEG	NEG	NEG	NEG	NEG	NEG	NEG	NEG	NEG	NEG	NEG	NEG	NEG	NEG	NEG	NEG	NEG
UKD-0510018	SCCmec VT+czrC (SO385)	CC398	NEG	NEG	NEG	NEG	NEG	NEG	NEG	NEG	NEG	NEG	NEG	NEG	NEG	NEG	NEG	NEG	NEG	NEG	NEG	NEG	NEG	NEG
UKD-0903558	SCCmec VT+czrC (SO385)	CC398	NEG	NEG	NEG	NEG	NEG	NEG	NEG	NEG	NEG	NEG	NEG	NEG	NEG	NEG	NEG	NEG	NEG	NEG	NEG	NEG	NEG	NEG
UKD-0929815	SCCmec VT+czrC (SO385)	CC398	NEG	NEG	NEG	NEG	NEG	NEG	NEG	NEG	NEG	NEG	NEG	NEG	NEG	NEG	NEG	NEG	NEG	NEG	NEG	NEG	NEG	NEG
UKD-0907762	SCCmec VT+czrC (SO385)	CC398	NEG	NEG	NEG	NEG	NEG	NEG	NEG	NEG	NEG	NEG	NEG	NEG	NEG	NEG	NEG	NEG	NEG	NEG	NEG	NEG	NEG	NEG
UKD-1025105	SCCmec VT+czrC (SO385)	CC398	NEG	NEG	NEG	NEG	NEG	NEG	NEG	NEG	NEG	NEG	NEG	NEG	NEG	NEG	NEG	NEG	NEG	NEG	NEG	NEG	NEG	NEG
UKD-11780898	SCCmec VT+czrC (SO385)	CC398	NEG	NEG	NEG	NEG	NEG	NEG	NEG	NEG	NEG	NEG	NEG	NEG	NEG	NEG	NEG	NEG	NEG	NEG	NEG	NEG	NEG	NEG
UKD-1377009	SCCmec VT+czrC (SO385)	CC398	NEG	NEG	NEG	NEG	NEG	NEG	NEG	NEG	NEG	NEG	NEG	NEG	NEG	NEG	NEG	NEG	NEG	NEG	NEG	NEG	NEG	NEG
UKD-143406	SCCmec VT+czrC (SO385)	CC398	NEG	NEG	NEG	NEG	NEG	NEG	NEG	NEG	NEG	NEG	NEG	NEG	NEG	NEG	NEG	NEG	NEG	NEG	NEG	NEG	NEG	NEG
UKD-1400363	SCCmec VT+czrC (SO385)	CC398	NEG	NEG	NEG	NEG	NEG	NEG	NEG	NEG	NEG	NEG	NEG	NEG	NEG	NEG	NEG	NEG	NEG	NEG	NEG	NEG	NEG	NEG
UKD-14571852	SCCmec VT+czrC (SO385)	CC398	NEG	NEG	NEG	NEG	NEG	NEG	NEG	NEG	NEG	NEG	NEG	NEG	NEG	NEG	NEG	NEG	NEG	NEG	NEG	NEG	NEG	NEG
UKD-14582574	SCCmec VT+czrC (SO385)	CC398	NEG	NEG	NEG	NEG	NEG	NEG	NEG	NEG	NEG	NEG	NEG	NEG	NEG	NEG	NEG	NEG	NEG	NEG	NEG	NEG	NEG	NEG
UKD-152108	SCCmec VT+czrC (SO385)	CC398	NEG	NEG	NEG	NEG	NEG	NEG	NEG	NEG	NEG	NEG	NEG	NEG	NEG	NEG	NEG	NEG	NEG	NEG	NEG	NEG	NEG	NEG
UKD-15573321	SCCmec VT+czrC (SO385)	CC398	NEG	NEG	NEG	NEG	NEG	NEG	NEG	NEG	NEG	NEG	NEG	NEG	NEG	NEG	NEG	NEG	NEG	NEG	NEG	NEG	NEG	NEG
UKD-15584034	SCCmec VT+czrC , ydhK-neg. variant	CC398	NEG	NEG	NEG	NEG	NEG	NEG	NEG	NEG	NEG	NEG	NEG	NEG	NEG	NEG	NEG	NEG	NEG	NEG	NEG	NEG	NEG	NEG
UKD-0932581	SCCmecVT+fus+czrC	CC22	NEG	NEG	POS	NEG	NEG	NEG	NEG	NEG	NEG	NEG	NEG	NEG	NEG	NEG	NEG	NEG	NEG	NEG	NEG	NEG	NEG	NEG
UKD-1121994	SCCmecVT+fus+czrC	CC22	NEG	NEG	POS	NEG	NEG	NEG	NEG	NEG	NEG	NEG	NEG	NEG	NEG	NEG	NEG	NEG	NEG	NEG	NEG	NEG	NEG	NEG
UKD-1272252	SCCmecVT+fus+czrC	CC22	NEG	NEG	POS	NEG	NEG	NEG	NEG	NEG	NEG	NEG	NEG	NEG	NEG	NEG	NEG	NEG	NEG	NEG	NEG	NEG	NEG	NEG
Prediction for MRSA18	SCCmec VI+fus (MRSA18)		NEG	NEG	POS	NEG	NEG	NEG	NEG	NEG	NEG	NEG	NEG	NEG	NEG	NEG	NEG	NEG	NEG	NEG	NEG	NEG	NEG	NEG
UKD-1103380	SCCmec VI+fus (MRSA18)	CC7	NEG	NEG	POS	NEG	NEG	NEG	NEG	NEG	NEG	NEG	NEG	NEG	NEG	NEG	NEG	NEG	NEG	NEG	NEG	NEG	NEG	NEG
Prediction for M10/0061, GenBank FR823292.1	SCCmec XI (LGA251/M10-61)	CC130	NEG	NEG	NEG	NEG	NEG	NEG	NEG	NEG	NEG	NEG	NEG	NEG	NEG	NEG	NEG	NEG	NEG	AMB	NEG	NEG	NEG	NEG
Reference strain Dublin-DSH_M10 0061	SCCmec XI (LGA251/M10-61)	CC130	NEG	NEG	NEG	NEG	NEG	NEG	NEG	NEG	NEG	NEG	NEG	NEG	NEG	NEG	NEG	NEG	NEG	AMB	NEG	NEG	NEG	AMB
Prediction for LGA251, GenBank FR821779.1	SCCmec XI (LGA251/M10-61)	ST425	NEG	NEG	NEG	NEG	NEG	NEG	NEG	NEG	NEG	NEG	NEG	NEG	NEG	NEG	NEG	NEG	NEG	AMB	NEG	NEG	NEG	NEG
UKD-14015835	SCCmec XI (LGA251/M10-61)	CC130	NEG	NEG	NEG	NEG	NEG	NEG	NEG	NEG	NEG	NEG	NEG	NEG	NEG	NEG	NEG	NEG	NEG	NEG	NEG	NEG	NEG	NEG
UKD-16573252	SCCmec XI (LGA251/M10-61)	CC130	NEG	NEG	NEG	NEG	NEG	NEG	NEG	NEG	NEG	NEG	NEG	NEG	NEG	NEG	NEG	NEG	NEG	AMB	NEG	NEG	AMB	NEG
UKD-0217002	PseudoSCCmec, class B mec complex+Hg	CC8	NEG	NEG	NEG	NEG	NEG	NEG	NEG	NEG	NEG	NEG	NEG	NEG	NEG	NEG	NEG	NEG	NEG	NEG	NEG	NEG	NEG	NEG
UKD-0008510	PseudoSCCmec, class B mec complex+Hg	CC8	NEG	NEG	NEG	NEG	NEG	NEG	NEG	NEG	NEG	NEG	NEG	NEG	NEG	NEG	NEG	NEG	NEG	NEG	NEG	NEG	NEG	NEG
UKD-0110962	PseudoSCCmec, class B mec complex+Hg	CC8	NEG	NEG	NEG	NEG	NEG	NEG	NEG	NEG	NEG	NEG	NEG	NEG	NEG	NEG	NEG	NEG	NEG	NEG	NEG	NEG	NEG	AMB
KHDN-10408593	PseudoSCCmec, class B mec complex+Cu/Hg	CC8	NEG	NEG	NEG	NEG	NEG	NEG	NEG	NEG	NEG	NEG	NEG	NEG	NEG	NEG	NEG	NEG	NEG	NEG	NEG	NEG	NEG	NEG
UKD-0007201	PseudoSCCmec, class B mec complex	CC5	NEG	NEG	NEG	NEG	NEG	NEG	NEG	NEG	NEG	NEG	NEG	NEG	NEG	NEG	NEG	NEG	NEG	NEG	NEG	NEG	NEG	NEG
UKD-0033204	PseudoSCCmec, class B mec complex	CC5	NEG	NEG	NEG	NEG	NEG	NEG	NEG	NEG	NEG	NEG	NEG	NEG	NEG	NEG	NEG	NEG	NEG	NEG	NEG	NEG	NEG	NEG
UKD-0134329	PseudoSCCmec, class B mec complex	CC5	NEG	NEG	NEG	NEG	NEG	NEG	NEG	NEG	NEG	NEG	NEG	NEG	NEG	NEG	NEG	NEG	NEG	NEG	NEG	NEG	NEG	NEG
UKD-0806946	PseudoSCCmec class C+As/Cu	CC398	NEG	NEG	NEG	NEG	NEG	NEG	NEG	NEG	NEG	NEG	NEG	NEG	NEG	NEG	NEG	NEG	NEG	NEG	NEG	NEG	NEG	NEG
UKD-0500610	ACME2	CC8	NEG	NEG	NEG	NEG	NEG	POS	POS	POS	POS	NEG	NEG	NEG	NEG	NEG	NEG	NEG	NEG	NEG	NEG	NEG	NEG	AMB
UKD-0610438	ACME2	CC8	NEG	NEG	NEG	NEG	NEG	POS	POS	neg	POS	NEG	NEG	NEG	NEG	NEG	NEG	NEG	NEG	NEG	NEG	NEG	NEG	NEG
KHDN-10409344	ACME2	CC8	NEG	NEG	NEG	NEG	NEG	POS	POS	POS	POS	NEG	NEG	NEG	NEG	NEG	NEG	NEG	NEG	NEG	NEG	NEG	NEG	NEG
UKD-0322559	ACME2+speG+czrC+ccrAB4	CC8	NEG	NEG	NEG	NEG	NEG	POS	POS	POS	POS	NEG	NEG	NEG	NEG	NEG	NEG	POS	NEG	NEG	NEG	NEG	NEG	NEG
UKD-0501998	ACME2+speG+czrC+ccrAB4	CC8	NEG	NEG	NEG	NEG	NEG	POS	POS	POS	POS	NEG	NEG	NEG	NEG	NEG	NEG	POS	NEG	NEG	NEG	NEG	NEG	NEG
UKD-0505213	ACME2+speG+czrC+ccrAB4	CC8	NEG	NEG	NEG	NEG	NEG	POS	POS	POS	POS	NEG	NEG	NEG	NEG	NEG	NEG	POS	NEG	NEG	NEG	NEG	NEG	AMB
Prediction for MSSA476, GenBank BX571857.1	fus+tir+ccrAB1 (MSSA476)	CC1	NEG	NEG	POS	POS	NEG	NEG	NEG	NEG	NEG	NEG	NEG	NEG	NEG	NEG	NEG	NEG	POS	POS	NEG	NEG	NEG	AMB
Reference strain Sanger 476	fus+tir+ccrAB1 (MSSA476)	CC1	NEG	NEG	POS	POS	NEG	NEG	NEG	NEG	NEG	NEG	NEG	NEG	NEG	NEG	NEG	NEG	POS	POS	NEG	NEG	NEG	NEG
UKD-14023914	fus+tir+ccrAB1 (MSSA476)	CC1	NEG	NEG	POS	POS	NEG	NEG	NEG	NEG	NEG	NEG	NEG	NEG	NEG	NEG	NEG	NEG	POS	POS	NEG	NEG	NEG	NEG
UKD-K00034	kdp+ccrAB2	CC182	NEG	NEG	NEG	NEG	NEG	NEG	NEG	NEG	NEG	NEG	NEG	NEG	NEG	NEG	NEG	NEG	NEG	NEG	POS	POS	NEG	NEG
UKD-15580369	As+ccrAB4	CC22	NEG	NEG	NEG	NEG	NEG	NEG	NEG	NEG	NEG	NEG	NEG	NEG	NEG	NEG	NEG	NEG	NEG	NEG	NEG	NEG	NEG	NEG
UKD-15570100	ACME3+ccrAB1 (Strain 21342)	CC80	NEG	NEG	NEG	NEG	NEG	NEG	NEG	NEG	NEG	POS	AMB	POS	NEG	POS	NEG	NEG	POS	POS	NEG	NEG	NEG	NEG
UKD-1010678	speG+czrC+ccrAB4	CC8	NEG	NEG	NEG	NEG	NEG	NEG	NEG	NEG	NEG	NEG	NEG	NEG	NEG	NEG	NEG	POS	NEG	NEG	NEG	NEG	NEG	NEG
UKD-1008243	speG+czrC+ccrAB4	CC8	NEG	NEG	NEG	NEG	NEG	NEG	NEG	NEG	NEG	NEG	NEG	NEG	NEG	NEG	NEG	POS	NEG	NEG	NEG	NEG	NEG	NEG
UKD-1009430	speG+czrC+ccrAB4	CC8	NEG	NEG	NEG	NEG	NEG	NEG	NEG	NEG	NEG	NEG	NEG	NEG	NEG	NEG	NEG	POS	NEG	NEG	NEG	NEG	NEG	NEG
UKD-12071828	speG+czrC+ccrAB4	CC22	NEG	NEG	NEG	NEG	NEG	NEG	NEG	NEG	NEG	NEG	NEG	NEG	NEG	NEG	NEG	POS	NEG	NEG	NEG	NEG	NEG	NEG
UKD-14011883	speG+czrC+ccrAB4	CC22	NEG	NEG	NEG	NEG	NEG	NEG	NEG	NEG	NEG	NEG	NEG	NEG	NEG	NEG	NEG	POS	NEG	NEG	NEG	NEG	NEG	NEG
UKD-15008293	speG+czrC+ccrAB4	CC22	NEG	NEG	NEG	NEG	NEG	NEG	NEG	NEG	NEG	NEG	NEG	NEG	NEG	NEG	NEG	POS	NEG	NEG	NEG	NEG	NEG	NEG
UKD-1514877	speG+czrC+ccrAB4	CC22	NEG	NEG	NEG	NEG	NEG	NEG	NEG	NEG	NEG	NEG	NEG	NEG	NEG	NEG	NEG	POS	NEG	NEG	NEG	NEG	NEG	NEG
UKD-15580971	speG+czrC+ccrAB4	CC22	NEG	NEG	NEG	NEG	NEG	NEG	NEG	NEG	NEG	NEG	NEG	NEG	NEG	NEG	NEG	POS	NEG	NEG	NEG	NEG	NEG	NEG
UKD-15582646	speG+czrC+ccrAB4	CC22	NEG	NEG	NEG	NEG	NEG	NEG	NEG	NEG	NEG	NEG	NEG	NEG	NEG	NEG	NEG	POS	NEG	NEG	NEG	NEG	NEG	NEG

Isolate ID	SCCmec type	Clonal complex	METHICILLIN RESISTANCE AND SCCmec TYPING																		
			cassette chromosome recombinase genes					hypothetical protein from SCCmec elements	SCCmec Termini												
			ccrAA (MRSAZH47)_probe 1	ccrAA (MRSAZH47)_probe 2	ccrC (85-2082)	ccrA-4	ccrB-4	Q9XB68-dcs	SCCmec Terminus 1	SCCmec Terminus 2	SCCmec Terminus 3	SCCmec Terminus 4	SCCmec Terminus 5	SCCmec Terminus 6	SCCmec Terminus 7	SCCmec Terminus 9	SCCmec Terminus 10	SCCmec Terminus 11	SCCmec Terminus 12	SCCmec Terminus 13	SCCmec Terminus 14
VIRTUAL HYBRIDISATION for COL, GenBank CP000046	SCCmec I (COL)	CC8	NEG	NEG	NEG	NEG	NEG	POS	NEG	NEG	NEG	NEG	NEG	NEG	NEG	NEG	NEG	NEG	NEG	NEG	NEG
Reference strain COL	SCCmec I (COL)	CC8	NEG	NEG	NEG	NEG	NEG	POS	NEG	NEG	NEG	NEG	NEG	NEG	NEG	NEG	NEG	NEG	NEG	NEG	NEG
UKD-0007063	SCCmec I (COL)	CC5	NEG	NEG	NEG	NEG	NEG	POS	NEG	NEG	NEG	NEG	NEG	NEG	NEG	NEG	NEG	NEG	NEG	NEG	NEG
UKD-0220759	SCCmec I (COL)	CC5	NEG	NEG	NEG	NEG	NEG	AMB	NEG	NEG	NEG	NEG	NEG	NEG	NEG	NEG	NEG	NEG	NEG	NEG	NEG
UKD-0308986	SCCmec I (COL)	CC5	NEG	NEG	NEG	NEG	NEG	POS	NEG	NEG	NEG	NEG	NEG	NEG	NEG	NEG	NEG	NEG	NEG	NEG	NEG
UKD-0500009	SCCmec I (COL)	CC5	NEG	NEG	NEG	NEG	NEG	POS	NEG	NEG	NEG	NEG	NEG	NEG	NEG	NEG	NEG	NEG	NEG	NEG	NEG
UKD-0508483	SCCmec I (COL)	CC5	NEG	NEG	NEG	NEG	NEG	POS	NEG	NEG	NEG	NEG	NEG	NEG	NEG	NEG	NEG	NEG	NEG	NEG	NEG
UKD-0519001	SCCmec I (COL)	CC5	NEG	NEG	NEG	NEG	NEG	POS	NEG	NEG	NEG	NEG	NEG	NEG	NEG	NEG	NEG	NEG	NEG	NEG	NEG
UKD-0701876	SCCmec I (COL)	CC5	NEG	NEG	NEG	NEG	NEG	POS	NEG	NEG	NEG	NEG	NEG	NEG	NEG	NEG	NEG	NEG	NEG	NEG	NEG
UKD-0708865	SCCmec I (COL)	CC5	NEG	NEG	NEG	NEG	NEG	POS	NEG	NEG	NEG	NEG	NEG	NEG	NEG	NEG	NEG	NEG	NEG	NEG	NEG
UKD-1001782	SCCmec I (COL)	CC5	NEG	NEG	NEG	NEG	NEG	POS	NEG	NEG	NEG	NEG	NEG	NEG	NEG	NEG	NEG	NEG	NEG	NEG	NEG
Prediction for PSP1996, GenBank ANHU	SCCmec I (PSP1996)	CC8	NEG	NEG	NEG	NEG	NEG	POS	NEG	NEG	NEG	NEG	NEG	NEG	NEG	NEG	NEG	NEG	NEG	NEG	NEG
UKD-0261727	SCCmec I (PSP1996)	CC8	NEG	NEG	NEG	NEG	NEG	POS	NEG	NEG	NEG	NEG	NEG	NEG	NEG	NEG	NEG	NEG	NEG	NEG	NEG
VIRTUAL HYBRIDISATION for MRSA7 : VIRTUAL HYBRIL	SCCmec I+fus+tirS (Geraldine Clone)	CC5	NEG	NEG	NEG	NEG	NEG	POS	NEG	NEG	NEG	NEG	NEG	NEG	NEG	NEG	NEG	NEG	NEG	NEG	NEG
UKD-1221930	SCCmec I+fus+tirS (Geraldine Clone)	CC5	NEG	NEG	NEG	NEG	NEG	POS	NEG	NEG	NEG	NEG	NEG	NEG	NEG	NEG	NEG	NEG	NEG	NEG	NEG
Prediction for N315, GenBank BA000018.3	SCCmec II (N315)	CC5	NEG	NEG	NEG	NEG	NEG	POS	AMB	NEG	NEG	NEG	NEG	NEG	NEG	NEG	NEG	NEG	NEG	NEG	NEG
Reference strain N315	SCCmec II (N315)	CC5	NEG	NEG	NEG	NEG	NEG	POS	NEG	NEG	NEG	NEG	NEG	NEG	NEG	NEG	NEG	NEG	NEG	NEG	NEG
Prediction for MRSA252, GenBank BX571856.1	SCCmec II (N315)	CC30	NEG	NEG	NEG	NEG	NEG	POS	AMB	NEG	NEG	NEG	NEG	NEG	NEG	NEG	NEG	NEG	NEG	NEG	NEG
Reference strain Sanger 252	SCCmec II (N315)	CC30	NEG	NEG	NEG	NEG	NEG	POS	NEG	NEG	NEG	NEG	NEG	NEG	NEG	NEG	NEG	NEG	NEG	NEG	NEG
UKD-0230792	SCCmec II (N315)	CC30	NEG	NEG	NEG	NEG	NEG	POS	NEG	NEG	NEG	NEG	NEG	NEG	NEG	NEG	NEG	NEG	NEG	NEG	NEG
Prediction for Mu50, GenBank BA000017.4	SCCmec II (N315)	CC5	NEG	NEG	NEG	NEG	NEG	POS	AMB	NEG	NEG	NEG	NEG	NEG	NEG	NEG	NEG	NEG	NEG	NEG	NEG
Reference strain MU50	SCCmec II (N315)	CC5	NEG	NEG	NEG	NEG	NEG	POS	NEG	NEG	NEG	NEG	NEG	NEG	NEG	NEG	NEG	NEG	NEG	NEG	NEG
Prediction for JH1, GenBank CP000736.1	SCCmec II (JH1/JH9)	CC5	NEG	NEG	NEG	NEG	NEG	POS	AMB	NEG	NEG	NEG	NEG	NEG	NEG	NEG	NEG	NEG	NEG	NEG	NEG
Prediction for JH9, GenBank CP000703.1	SCCmec II (JH1/JH9)	CC5	NEG	NEG	NEG	NEG	NEG	POS	AMB	NEG	NEG	NEG	NEG	NEG	NEG	NEG	NEG	NEG	NEG	NEG	NEG
Reference strain Dublin-DSH_AR07.4_0237	SCCmec II (JH1/JH9)		NEG	NEG	NEG	NEG	NEG	POS	NEG	NEG	NEG	NEG	NEG	NEG	NEG	NEG	NEG	NEG	NEG	NEG	NEG
Reference strain NARSA_382 / USA100	SCCmec II (JH1/JH9)	CC5	NEG	NEG	NEG	NEG	NEG	POS	NEG	NEG	NEG	NEG	NEG	NEG	NEG	NEG	NEG	NEG	NEG	NEG	NEG
UKD-0046921	SCCmec II (JH1/JH9)	CC5	NEG	NEG	NEG	NEG	NEG	POS	NEG	NEG	NEG	NEG	NEG	NEG	NEG	NEG	NEG	NEG	NEG	NEG	NEG
UKD-0333415	SCCmec II (JH1/JH9)	CC5	NEG	NEG	NEG	NEG	NEG	POS	NEG	NEG	NEG	NEG	NEG	NEG	NEG	NEG	NEG	NEG	NEG	NEG	NEG
UKD-0341645	SCCmec II (JH1/JH9)	CC5	NEG	NEG	NEG	NEG	NEG	POS	NEG	NEG	NEG	NEG	NEG	NEG	NEG	NEG	NEG	NEG	NEG	NEG	NEG
UKD-0504626	SCCmec II (JH1/JH9)	CC5	NEG	NEG	NEG	NEG	NEG	AMB	NEG	NEG	NEG	NEG	NEG	NEG	NEG	NEG	NEG	NEG	NEG	NEG	NEG
UKD-0507581	SCCmec II (JH1/JH9)	CC5	NEG	NEG	NEG	NEG	NEG	POS	NEG	NEG	NEG	NEG	NEG	NEG	NEG	NEG	NEG	NEG	NEG	NEG	NEG
UKD-0507903	SCCmec II (JH1/JH9)	CC5	NEG	NEG	NEG	NEG	NEG	POS	NEG	NEG	NEG	NEG	NEG	NEG	NEG	NEG	NEG	NEG	NEG	NEG	NEG
UKD-0700817	SCCmec II (JH1/JH9)	CC5	NEG	NEG	NEG	NEG	NEG	AMB	NEG	NEG	NEG	NEG	NEG	NEG	NEG	NEG	NEG	NEG	NEG	NEG	NEG
UKD-1014561	SCCmec II (JH1/JH9)	CC5	NEG	NEG	NEG	NEG	NEG	POS	NEG	NEG	NEG	NEG	NEG	NEG	NEG	NEG	NEG	NEG	NEG	NEG	NEG
UKD-1037507	SCCmec II (JH1/JH9)	CC5	NEG	NEG	NEG	NEG	NEG	POS	NEG	NEG	NEG	NEG	NEG	NEG	AMB	NEG	NEG	NEG	NEG	NEG	NEG
UKD-1038321	SCCmec II (JH1/JH9)	CC5	NEG	NEG	NEG	NEG	NEG	POS	NEG	NEG	NEG	NEG	NEG	NEG	AMB	NEG	NEG	NEG	NEG	NEG	NEG
UKD-1111608	SCCmec II (JH1/JH9)	CC5	NEG	NEG	NEG	NEG	NEG	POS	NEG	NEG	NEG	NEG	NEG	NEG	NEG	NEG	NEG	NEG	NEG	NEG	NEG
UKD-12753135	SCCmec II (JH1/JH9)	CC5	NEG	NEG	NEG	NEG	NEG	POS	NEG	NEG	NEG	NEG	NEG	NEG	AMB	NEG	NEG	NEG	NEG	NEG	NEG
UKD-1312816	SCCmec II (JH1/JH9)	CC5	NEG	NEG	NEG	NEG	NEG	POS	NEG	NEG	NEG	NEG	NEG	NEG	NEG	NEG	NEG	NEG	NEG	NEG	NEG
UKD-1401623	SCCmec II (JH1/JH9)	CC5	NEG	NEG	NEG	NEG	NEG	POS	NEG	NEG	NEG	NEG	NEG	NEG	POS	NEG	NEG	NEG	NEG	NEG	NEG
UKD-15571943	SCCmec II+czrC+ccrA/B-4	CC5	NEG	NEG	NEG	POS	POS	POS	NEG	NEG	NEG	NEG	NEG	NEG	NEG	NEG	NEG	NEG	NEG	NEG	NEG
UKD-15576888	SCCmec II+czrC+ccrA/B-4	CC5	NEG	NEG	NEG	POS	POS	POS	NEG	NEG	NEG	NEG	NEG	NEG	NEG	NEG	NEG	NEG	NEG	NEG	NEG
UKD-14576461	SCCmec II+speG+czrC+ccrA/B-4	CC5	NEG	NEG	NEG	POS	POS	POS	NEG	NEG	NEG	NEG	NEG	NEG	POS	NEG	NEG	NEG	NEG	NEG	NEG
UKD-15581649	SCCmec II+speG+czrC+ccrA/B-4	CC5	NEG	NEG	NEG	POS	POS	POS	NEG	NEG	NEG	NEG	NEG	NEG	POS	NEG	NEG	NEG	NEG	NEG	NEG
Prediction for CIGC345D, GenBank AHVO	SCCmec II A/B/D	CC8	NEG	NEG	NEG	NEG	NEG	POS	NEG	NEG	NEG	NEG	NEG	NEG	NEG	NEG	NEG	NEG	NEG	NEG	NEG
UKD-12K00171	SCCmec II A/B/D	CC8	NEG	NEG	NEG	NEG	NEG	POS	NEG	NEG	NEG	NEG	NEG	NEG	NEG	NEG	NEG	NEG	NEG	NEG	NEG
Prediction for JKD6008, GenBank CP002120.1	SCC [mec III+Cd] (JKD6008)	CC8 (ST239)	NEG	NEG	NEG	NEG	NEG	POS	NEG	NEG	NEG	NEG	NEG	NEG	NEG	NEG	NEG	NEG	NEG	NEG	NEG
Prediction for JKD6009, GenBank ABSA	SCC [mec III+Cd] (JKD6008)	CC8 (ST239)	NEG	NEG	NEG	NEG	NEG	POS	NEG	NEG	NEG	NEG	NEG	NEG	NEG	NEG	NEG	NEG	NEG	NEG	NEG
Reference strain ATCC_33592	SCC [mec III+Cd] (ATCC33592)	CC8 (ST239)	NEG	NEG	NEG	NEG	NEG	NEG	POS	NEG	NEG	NEG	NEG	NEG	NEG	NEG	NEG	NEG	NEG	NEG	NEG
Prediction for PPUKM-775-2009, GenBank AMRE	SCC [mec III+Cd+ccrC] (PPUKM-775-2009)	CC8 (ST239)	NEG	NEG	POS	NEG	NEG	NEG	NEG	POS	NEG	NEG	NEG	NEG	NEG	NEG	NEG	NEG	NEG	NEG	NEG
Prediction for T0131, GenBank CP002643.1	SCC [mec III+Cd+ccrC] (T0131)	CC8 (ST239)	NEG	NEG	POS	NEG	NEG	POS	NEG	POS	NEG	NEG	NEG	NEG	NEG	NEG	NEG	NEG	NEG	NEG	NEG
Prediction for XN108, GenBank CP007447.1	SCC [mec III+Cd+ccrC] (XN108)	CC8 (ST239)	NEG	NEG	POS	NEG	NEG	NEG	NEG	POS	NEG	NEG	NEG	NEG	NEG	NEG	NEG	NEG	NEG	NEG	NEG
Prediction for CN79, GenBank ANCJ	SCC [mec III+Cd+ccrC] (CN79)	CC8 (ST239)	NEG	NEG	POS	NEG	NEG	POS	NEG	POS	NEG	NEG	NEG	NEG	NEG	NEG	NEG	NEG	NEG	NEG	NEG
Prediction for 16K, GenBank BABZ	SCC [mec III+Cd+ccrC] (CN79)	CC8 (ST239)	NEG	NEG	POS	NEG	NEG	POS	NEG	POS	NEG	NEG	NEG	NEG	NEG	NEG	NEG	NEG	NEG	NEG	NEG
UKD-0835987	SCC [mec III+Cd+ccrC] (CN79)	CC8 (ST239)	NEG	NEG	POS	NEG	NEG	POS	NEG	POS	NEG	NEG	NEG	NEG	NEG	NEG	NEG	NEG	NEG	NEG	NEG
UKD-16581769	SCCmec III+Cd+ccrC	CC8 (ST239)	NEG	POS	POS	NEG	NEG	POS	NEG	POS	NEG	NEG	POS	NEG	NEG	NEG	NEG	NEG	NEG	NEG	NEG
Prediction for Bmb9393, GenBank CP005288.1	SCCmec III+Cd/Hg+ccrC (Bmb9393)	CC8 (ST239)	NEG	NEG	POS	NEG	NEG	NEG	NEG	POS	NEG	NEG	NEG	NEG	NEG	NEG	NEG	NEG	NEG	NEG	NEG
UKD-15574263	SCCmec III+Cd/Hg+ccrC (Bmb9393)	CC8 (ST239)	NEG	NEG	POS	NEG	NEG	NEG	NEG	POS	NEG	NEG	NEG	NEG	NEG	NEG	NEG	NEG	NEG	NEG	NEG
Prediction for SK1585, GenBank AYLT	SCCmec III+ccrC+Cd/Hg (SK1585)	CC8 (ST239)	NEG	NEG	POS	NEG	NEG	NEG	POS	POS	NEG	NEG	NEG	NEG	NEG	NEG	NEG	NEG	NEG	NEG	NEG
UKD-105861	SCCmec III+ccrC+Cd/Hg (SK1585)	CC8 (ST239)	NEG	NEG	POS	NEG	NEG	NEG	POS	POS	NEG	NEG	NEG	NEG	AMB	NEG	NEG	NEG	NEG	NEG	NEG
UKD-134572	SCCmec III+ccrC+Cd/Hg (SK1585)	CC8 (ST239)	NEG	NEG	POS	NEG	NEG	NEG	AMB	POS	NEG	NEG	NEG	NEG	NEG	NEG	NEG	NEG	NEG	NEG	NEG
UKD-139143	SCCmec III+ccrC+Cd/Hg (SK1585)	CC8 (ST239)	NEG	NEG	POS	NEG	NEG	NEG	POS	POS	NEG	NEG	NEG	NEG	NEG	NEG	NEG	NEG	NEG	NEG	NEG
Prediction for TW20, GenBank FN433596.1	SCC [mec III+Cd/Hg+ccrC] (TW20)	CC8 (ST239)	NEG	NEG	POS	NEG	NEG	NEG	POS	POS	NEG	NEG	NEG	NEG	NEG	NEG	NEG	NEG	NEG	NEG	NEG
Prediction for ATCC BAA-39, GenBank AEEK	SCC [mec III+Cd/Hg+ccrC] (TW20)	CC8 (ST239)	NEG	NEG	POS	NEG	NEG	NEG	POS	POS	NEG	NEG	NEG	NEG	NEG	NEG	NEG	NEG	NEG	NEG	NEG

Isolate ID	SCCmec type	Clonal complex	METHICILLIN RESISTANCE AND SCCmec TYPING																		
			cassette chromosome recombinase genes					hypothetical protein from SCCmec elements	SCCmec Termini												
			ccrAA (MRSAZH47)_probe 1	ccrAA (MRSAZH47)_probe 2	ccrC (85-2082)	ccrA-4	ccrB-4	Q9XB68-dcs	SCCmec Terminus 1	SCCmec Terminus 2	SCCmec Terminus 3	SCCmec Terminus 4	SCCmec Terminus 5	SCCmec Terminus 6	SCCmec Terminus 7	SCCmec Terminus 9	SCCmec Terminus 10	SCCmec Terminus 11	SCCmec Terminus 12	SCCmec Terminus 13	SCCmec Terminus 14
Prediction for MW2, GenBank BA000033.2	*SCCmec IVa (MW2)*	*CC1*	*NEG*	*NEG*	*NEG*	*NEG*	*NEG*	*POS*	*NEG*	*NEG*	*NEG*	*NEG*	*NEG*	*NEG*	*NEG*	*NEG*	*NEG*	*NEG*	*NEG*	*NEG*	*NEG*
**Reference strain MW2**	**SCCmec IVa (MW2)**	**CC1**	**NEG**	**NEG**	**NEG**	**NEG**	**NEG**	**POS**	**NEG**	**NEG**	**NEG**	**NEG**	**NEG**	**NEG**	**NEG**	**NEG**	**NEG**	**NEG**	**NEG**	**NEG**	**NEG**
**Reference strain CA05 (JCSC1968)**	**SCCmec IVa (MW2)**	**CC45**	**NEG**	**NEG**	**NEG**	**NEG**	**NEG**	**POS**	**NEG**	**NEG**	**NEG**	**NEG**	**NEG**	**NEG**	**NEG**	**NEG**	**NEG**	**NEG**	**NEG**	**NEG**	**NEG**
**UKD-0127106**	SCCmec IVa (MW2)	CC1	NEG	NEG	NEG	NEG	NEG	AMB	NEG	NEG	NEG	NEG	NEG	NEG	AMB	NEG	NEG	NEG	NEG	NEG	NEG
**UKD-0701446**	SCCmec IVa (MW2)	CC1	NEG	NEG	NEG	NEG	NEG	AMB	NEG	NEG	NEG	NEG	NEG	NEG	NEG	NEG	NEG	NEG	NEG	NEG	NEG
**UKD-0947486**	SCCmec IVa (MW2)	CC1	NEG	NEG	NEG	NEG	NEG	NEG	NEG	NEG	NEG	NEG	NEG	NEG	NEG	NEG	NEG	NEG	NEG	NEG	NEG
**UKD-1031910**	SCCmec IVa (MW2)	CC1	NEG	NEG	NEG	NEG	NEG	NEG	NEG	NEG	NEG	NEG	NEG	NEG	NEG	NEG	NEG	NEG	NEG	NEG	NEG
**UKD-14571328**	SCCmec IVa (MW2)	CC1	NEG	NEG	NEG	NEG	NEG	NEG	NEG	NEG	NEG	NEG	NEG	NEG	NEG	NEG	NEG	NEG	NEG	NEG	NEG
**UKD-03003012**	SCCmec IVa (MW2)	CC5	NEG	NEG	NEG	NEG	NEG	POS	NEG	NEG	NEG	NEG	NEG	NEG	NEG	NEG	NEG	NEG	NEG	NEG	NEG
**UKD-15582582**	SCCmec IVa (MW2)	CC6	NEG	NEG	NEG	NEG	NEG	POS	NEG	NEG	NEG	NEG	NEG	NEG	NEG	NEG	NEG	NEG	NEG	NEG	NEG
**UKD-16574230**	SCCmec IVa (MW2)	CC6	NEG	NEG	NEG	NEG	NEG	POS	NEG	NEG	NEG	NEG	NEG	NEG	NEG	NEG	NEG	NEG	NEG	NEG	NEG
**UKD-16582560**	SCCmec IVa (MW2)	CC6	NEG	NEG	NEG	NEG	NEG	POS	NEG	NEG	NEG	NEG	NEG	NEG	NEG	NEG	NEG	NEG	NEG	NEG	NEG
**UKD-0802855**	SCCmec IVa (MW2)	CC7	NEG	NEG	NEG	NEG	NEG	POS	NEG	NEG	NEG	NEG	NEG	NEG	NEG	NEG	NEG	NEG	NEG	NEG	NEG
**KHDN-10405258**	SCCmec IVa (MW2)	CC8	NEG	NEG	NEG	NEG	NEG	POS	NEG	NEG	NEG	NEG	NEG	NEG	NEG	NEG	NEG	NEG	NEG	NEG	NEG
**UKD-15573497**	SCCmec IVa (MW2)	CC22	NEG	NEG	NEG	NEG	NEG	POS	NEG	NEG	NEG	NEG	NEG	NEG	NEG	NEG	NEG	NEG	NEG	NEG	NEG
**UKD-0634729**	SCCmec IVa (MW2)	CC30	NEG	NEG	NEG	NEG	NEG	NEG	NEG	NEG	NEG	NEG	NEG	NEG	NEG	NEG	NEG	NEG	NEG	NEG	NEG
**UKD-1119771**	SCCmec IVa (MW2)	CC30	NEG	NEG	NEG	NEG	NEG	POS	NEG	NEG	NEG	NEG	NEG	NEG	NEG	NEG	NEG	NEG	NEG	NEG	NEG
**KHDN-10411458**	SCCmec IVa (MW2)	CC30	NEG	NEG	NEG	NEG	NEG	POS	neg	NEG	NEG	NEG	NEG	NEG	NEG	NEG	NEG	NEG	neg	NEG	NEG
**KHDN-398609**	SCCmec IVa (MW2)	CC30	NEG	NEG	NEG	NEG	NEG	POS	NEG	NEG	NEG	NEG	NEG	NEG	NEG	NEG	NEG	NEG	NEG	NEG	NEG
**KHDN-01081998**	SCCmec IVa (MW2)	CC30	NEG	NEG	NEG	NEG	NEG	POS	NEG	NEG	NEG	NEG	NEG	NEG	NEG	NEG	NEG	NEG	NEG	NEG	NEG
**KHDN-10399223**	SCCmec IVa (MW2)	CC30	NEG	NEG	NEG	NEG	NEG	POS	NEG	NEG	NEG	NEG	NEG	NEG	NEG	NEG	NEG	NEG	NEG	NEG	NEG
**KHDN-W261158**	SCCmec IVa (MW2)	CC30	NEG	NEG	NEG	NEG	NEG	POS	neg	NEG	NEG	NEG	NEG	NEG	NEG	NEG	NEG	NEG	neg	NEG	NEG
**UKD-0002046**	SCCmec IVa (MW2)	CC45 [agr I]	NEG	NEG	NEG	NEG	NEG	POS	NEG	NEG	NEG	NEG	NEG	NEG	NEG	NEG	NEG	NEG	NEG	NEG	NEG
**UKD-0029618**	SCCmec IVa (MW2)	CC45 [agr I]	NEG	NEG	NEG	NEG	NEG	POS	NEG	NEG	NEG	NEG	NEG	NEG	NEG	NEG	NEG	NEG	NEG	NEG	NEG
**UKD-0043085**	SCCmec IVa (MW2)	CC45 [agr I]	NEG	NEG	NEG	NEG	NEG	POS	NEG	NEG	NEG	NEG	NEG	NEG	NEG	NEG	NEG	NEG	NEG	NEG	NEG
**UKD-0047053**	SCCmec IVa (MW2)	CC45 [agr I]	NEG	NEG	NEG	NEG	NEG	AMB	NEG	NEG	NEG	NEG	NEG	NEG	NEG	NEG	NEG	NEG	NEG	NEG	NEG
**UKD-0047429**	SCCmec IVa (MW2)	CC45 [agr I]	NEG	NEG	NEG	NEG	NEG	POS	NEG	NEG	NEG	NEG	NEG	NEG	NEG	NEG	NEG	NEG	NEG	NEG	NEG
**UKD-0112465**	SCCmec IVa (MW2)	CC45 [agr I]	NEG	NEG	NEG	NEG	NEG	POS	NEG	NEG	NEG	NEG	NEG	NEG	NEG	NEG	NEG	NEG	NEG	NEG	NEG
**UKD-0103483**	SCCmec IVa (MW2)	CC45 [agr I]	NEG	NEG	NEG	NEG	NEG	POS	NEG	NEG	NEG	NEG	NEG	NEG	NEG	NEG	NEG	NEG	NEG	NEG	NEG
**UKD-0305014**	SCCmec IVa (MW2)	CC45 [agr I]	NEG	NEG	NEG	NEG	NEG	POS	NEG	NEG	NEG	NEG	NEG	NEG	NEG	NEG	NEG	NEG	NEG	NEG	NEG
**UKD-0401119**	SCCmec IVa (MW2)	CC45 [agr I]	NEG	NEG	NEG	NEG	NEG	POS	NEG	NEG	NEG	NEG	NEG	NEG	NEG	NEG	NEG	NEG	NEG	NEG	NEG
**UKD-0537969**	SCCmec IVa (MW2)	CC45 [agr I]	NEG	NEG	NEG	NEG	NEG	POS	NEG	NEG	NEG	NEG	NEG	NEG	NEG	NEG	NEG	NEG	NEG	NEG	NEG
**UKD-0703373**	SCCmec IVa (MW2)	CC45 [agr I]	NEG	NEG	NEG	NEG	NEG	AMB	NEG	NEG	NEG	NEG	NEG	NEG	NEG	NEG	NEG	NEG	NEG	NEG	NEG
**UKD-0813653**	SCCmec IVa (MW2)	CC45 [agr I]	NEG	NEG	NEG	NEG	NEG	POS	NEG	NEG	NEG	NEG	NEG	NEG	NEG	NEG	NEG	NEG	NEG	NEG	NEG
**UKD-0839175**	SCCmec IVa (MW2)	CC45 [agr I]	NEG	NEG	NEG	NEG	NEG	POS	NEG	NEG	NEG	NEG	NEG	NEG	NEG	NEG	NEG	NEG	NEG	NEG	NEG
**UKD-1108422**	SCCmec IVa (MW2)	CC45 [agr I]	NEG	NEG	NEG	NEG	NEG	POS	NEG	NEG	NEG	NEG	NEG	NEG	NEG	NEG	NEG	NEG	NEG	NEG	NEG
**UKD-1380645**	SCCmec IVa (MW2)	CC45 [agr I]	NEG	NEG	NEG	NEG	NEG	POS	NEG	NEG	NEG	NEG	NEG	NEG	NEG	NEG	NEG	NEG	NEG	NEG	NEG
**UKD-148016**	SCCmec IVa (MW2)	CC45 [agr I]	NEG	NEG	NEG	NEG	NEG	POS	NEG	NEG	NEG	NEG	NEG	NEG	NEG	NEG	NEG	NEG	NEG	NEG	NEG
**UKD-16571773**	SCCmec IVa (MW2)	CC45 [agr I]	NEG	NEG	NEG	NEG	NEG	POS	NEG	NEG	NEG	NEG	NEG	NEG	AMB	NEG	NEG	NEG	NEG	NEG	NEG
**KHDN-10407738**	SCCmec IVa (MW2)	CC45 [ST617]	NEG	NEG	NEG	NEG	NEG	POS	NEG	NEG	NEG	NEG	POS	NEG	NEG	NEG	NEG	NEG	NEG	NEG	NEG
**UKD-0853670**	SCCmec IVa (MW2)	CC88	NEG	NEG	NEG	NEG	NEG	AMB	NEG	NEG	NEG	NEG	NEG	NEG	NEG	NEG	NEG	NEG	NEG	NEG	NEG
**UKD-1417352**	SCCmec IVa (MW2)	CC88	NEG	NEG	NEG	NEG	NEG	POS	NEG	NEG	NEG	NEG	NEG	NEG	NEG	NEG	NEG	NEG	NEG	NEG	NEG
**UKD-15571283**	SCCmec IVa (MW2)	CC88	NEG	NEG	NEG	NEG	NEG	POS	NEG	NEG	NEG	NEG	NEG	NEG	NEG	NEG	NEG	NEG	NEG	NEG	NEG
**KHDN-10406719**	SCCmec IVa (MW2)	ST93	NEG	NEG	NEG	NEG	NEG	POS	neg	NEG	NEG	NEG	NEG	NEG	NEG	NEG	NEG	NEG	neg	NEG	NEG
**KHDN-0060294**	SCCmec IVa (MW2)	ST93	NEG	NEG	NEG	NEG	NEG	POS	neg	NEG	NEG	NEG	NEG	NEG	NEG	NEG	NEG	NEG	neg	NEG	NEG
**UKD-0734190**	SCCmec IVa (MW2)	CC97	NEG	NEG	NEG	NEG	NEG	AMB	NEG	NEG	NEG	NEG	NEG	NEG	NEG	NEG	NEG	NEG	NEG	NEG	NEG
**UKD-13006983**	SCCmec IVa (MW2)	CC97	NEG	NEG	NEG	NEG	NEG	POS	NEG	NEG	NEG	NEG	NEG	NEG	NEG	NEG	NEG	NEG	NEG	NEG	NEG
Prediction for H131520133	*SCCmec IVa (H131520133)*	*CC152*	*NEG*	*NEG*	*NEG*	*NEG*	*NEG*	*POS*	*NEG*	*NEG*	*NEG*	*NEG*	*NEG*	*NEG*	*NEG*	*NEG*	*NEG*	*NEG*	*NEG*	*NEG*	*NEG*
**KHDN-325892**	SCCmec IVa (H131520133)	CC30	NEG	NEG	NEG	NEG	NEG	POS	NEG	NEG	NEG	NEG	NEG	NEG	NEG	NEG	NEG	NEG	NEG	NEG	NEG
**UKD-15580207**	SCCmec IVa (H131520133)	CC88	NEG	NEG	NEG	NEG	NEG	POS	NEG	NEG	NEG	NEG	NEG	NEG	NEG	NEG	NEG	NEG	NEG	NEG	NEG
**UKD-0521159**	SCCmec Iva	CC8	NEG	NEG	NEG	NEG	NEG	NEG	NEG	NEG	NEG	POS	NEG	NEG	NEG	NEG	NEG	NEG	NEG	NEG	NEG
Prediction for CMFT503 : HF569113.1	*SCCmec IVa (CMFT503)*		*NEG*	*NEG*	*NEG*	*NEG*	*NEG*	*NEG*	*POS*	*NEG*	*NEG*	*NEG*	*NEG*	*NEG*	*NEG*	*NEG*	*NEG*	*NEG*	*NEG*	*NEG*	*NEG*
Prediction for 21343, GenBank AHKV	*SCCmec IVa (CMFT503)*	*CC88*	*NEG*	*NEG*	*NEG*	*NEG*	*NEG*	*NEG*	*POS*	*NEG*	*NEG*	*NEG*	*NEG*	*NEG*	*NEG*	*NEG*	*NEG*	*NEG*	*NEG*	*NEG*	*NEG*
**UKD-1420740**	SCCmec IVa (CMFT503)	CC22	NEG	NEG	NEG	NEG	NEG	NEG	POS	NEG	NEG	NEG	NEG	NEG	NEG	NEG	NEG	NEG	NEG	NEG	NEG
**UKD-15575124**	SCCmec IVa (CMFT503)	CC22	NEG	NEG	NEG	NEG	NEG	NEG	POS	NEG	NEG	NEG	NEG	NEG	NEG	NEG	NEG	NEG	NEG	NEG	NEG
**UKD-16584947**	SCCmec IVa (CMFT503)	CC22	NEG	NEG	NEG	NEG	NEG	NEG	POS	NEG	NEG	NEG	NEG	NEG	NEG	NEG	NEG	NEG	NEG	NEG	NEG
**UKD-16575077**	SCCmec IVa (CMFT503)	CC88	NEG	NEG	NEG	NEG	NEG	NEG	POS	NEG	NEG	NEG	NEG	NEG	NEG	NEG	NEG	NEG	NEG	NEG	NEG
Prediction for TCH1516, GenBank CP000730.1	*SCCmec IVa+ACME1+Cu (USA300)*	*CC8*	*NEG*	*NEG*	*NEG*	*NEG*	*NEG*	*POS*	*NEG*	*NEG*	*NEG*	*NEG*	*NEG*	*NEG*	*NEG*	*NEG*	*NEG*	*NEG*	*NEG*	*NEG*	*NEG*
**Reference strain USA300-TCH1516**	SCCmec IVa+ACME1+Cu (USA300)	CC8	NEG	NEG	NEG	NEG	AMB	POS	NEG	NEG	NEG	NEG	NEG	NEG	NEG	NEG	NEG	NEG	NEG	NEG	NEG
Prediction for FPR3757, GenBank CP000255.1	*SCCmec IVa+ACME1+Cu (USA300)*	*CC8*	*NEG*	*NEG*	*NEG*	*NEG*	*NEG*	*POS*	*NEG*	*NEG*	*NEG*	*NEG*	*NEG*	*NEG*	*NEG*	*NEG*	*NEG*	*NEG*	*NEG*	*NEG*	*NEG*
**UKD-0516386**	SCCmec IVa+ACME1+Cu (USA300)	CC8	NEG	NEG	NEG	NEG	NEG	AMB	NEG	NEG	NEG	NEG	NEG	NEG	NEG	NEG	NEG	NEG	NEG	NEG	NEG
**UKD-0815773**	SCCmec IVa+ACME1+Cu (USA300)	CC8	NEG	NEG	NEG	NEG	NEG	POS	NEG	NEG	NEG	NEG	NEG	NEG	NEG	NEG	NEG	NEG	NEG	NEG	NEG
**UKD-1414654**	SCCmec IVa+ACME1+Cu (USA300)	CC8	NEG	NEG	NEG	NEG	NEG	POS	NEG	NEG	NEG	NEG	NEG	NEG	NEG	NEG	NEG	NEG	NEG	NEG	NEG
**UKD-0408036**	SCCmec IVa+ACME2	CC45 [agr I]	NEG	NEG	NEG	NEG	NEG	POS	NEG	NEG	POS	NEG	NEG	NEG	NEG	NEG	NEG	NEG	NEG	NEG	NEG
**UKD-0044035**	SCCmec IVa+ccrC (Hannover)	CC8	NEG	NEG	POS	NEG	NEG	POS	NEG	POS	NEG	NEG	NEG	NEG	NEG	NEG	NEG	NEG	NEG	NEG	NEG
**UKD-0136287**	SCCmec IVa+ccrC (Hannover)	CC8	NEG	NEG	POS	NEG	NEG	POS	NEG	POS	NEG	NEG	NEG	NEG	NEG	NEG	NEG	NEG	NEG	NEG	NEG

Isolate ID	SCCmec type	Clonal complex	METHICILLIN RESISTANCE AND SCCmec TYPING																		
			cassette chromosome recombinase genes					hypothetical protein from SCCmec elements	SCCmec Termini												
			ccrAA (MRSAZH47)_probe 1	ccrAA (MRSAZH47)_probe 2	ccrC (85-2082)	ccrA-4	ccrB-4	Q9XB68-dcs	SCCmec Terminus 1	SCCmec Terminus 2	SCCmec Terminus 3	SCCmec Terminus 4	SCCmec Terminus 5	SCCmec Terminus 6	SCCmec Terminus 7	SCCmec Terminus 9	SCCmec Terminus 10	SCCmec Terminus 11	SCCmec Terminus 12	SCCmec Terminus 13	SCCmec Terminus 14
Prediction for JCSC1978, GenBank AB063173.1	*SCCmec IVb/d/i (JCSC1978/6668/4469)*		NEG	NEG	NEG	NEG	NEG	POS	NEG	NEG	NEG	NEG	NEG	NEG	NEG	NEG	NEG	NEG	NEG	NEG	NEG
**Reference strain JCSC1978 (8/6-3P)**	**SCCmec IVb/d/i (JCSC1978/6668/4469)**	**CC8**	NEG	NEG	NEG	NEG	NEG	POS	NEG	NEG	NEG	NEG	NEG	NEG	NEG	NEG	NEG	NEG	NEG	NEG	NEG
Prediction for JCSC6668, GenBank AB425823.1	*SCCmec IVb/d/i (JCSC1978/6668/4469)*		NEG	NEG	NEG	NEG	NEG	POS	NEG	NEG	NEG	NEG	NEG	NEG	NEG	NEG	NEG	NEG	NEG	NEG	NEG
**Reference strain JCSC4469**	**SCCmec IVb/d/i (JCSC1978/6668/4469)**	**CC5**	NEG	NEG	NEG	NEG	NEG	POS	NEG	NEG	NEG	NEG	NEG	NEG	NEG	NEG	NEG	NEG	NEG	NEG	NEG
UKD-1103429	SCCmec IVb/d/i (JCSC1978/6668/4469)	CC7	NEG	NEG	NEG	NEG	NEG	POS	NEG	NEG	NEG	NEG	NEG	NEG	NEG	NEG	NEG	NEG	NEG	NEG	NEG
UKD-1306141	SCCmec IVb/d/i (JCSC1978/6668/4469)	CC7	NEG	NEG	NEG	NEG	NEG	POS	NEG	NEG	NEG	NEG	NEG	NEG	NEG	NEG	NEG	NEG	NEG	NEG	NEG
UKD-0500089	SCCmec IVb/d/i (JCSC1978/6668/4469)	CC5	NEG	NEG	NEG	NEG	NEG	POS	NEG	NEG	NEG	NEG	NEG	NEG	NEG	NEG	NEG	NEG	NEG	NEG	NEG
UKD-0501416	SCCmec IVb/d/i (JCSC1978/6668/4469)	CC5	NEG	NEG	NEG	NEG	NEG	AMB	NEG	NEG	NEG	NEG	NEG	NEG	NEG	NEG	NEG	NEG	NEG	NEG	NEG
UKD-0934574	SCCmec IVb/d/i (JCSC1978/6668/4469)	CC5	NEG	NEG	NEG	NEG	NEG	POS	NEG	NEG	NEG	NEG	NEG	NEG	NEG	NEG	NEG	NEG	NEG	NEG	NEG
UKD-0746779	SCCmec IVb/d/i (JCSC1978/6668/4469)	CC45 [agr I]	NEG	NEG	NEG	NEG	NEG	POS	NEG	NEG	NEG	NEG	NEG	NEG	NEG	NEG	NEG	NEG	NEG	NEG	NEG
UKD-0812518	SCCmec IVb/d/i (JCSC1978/6668/4469)	CC45 [agr I]	NEG	NEG	NEG	NEG	NEG	POS	NEG	NEG	NEG	NEG	NEG	NEG	NEG	NEG	NEG	NEG	NEG	NEG	NEG
Prediction for 21209, GenBank AGRP	*SCCmec IVb/d/i (21209)*	*CC8*	NEG	NEG	NEG	NEG	NEG	POS	NEG	NEG	NEG	NEG	NEG	NEG	NEG	NEG	NEG	NEG	NEG	NEG	NEG
UKD-0233579	SCCmec IVb/d/i (21209)	CC8	NEG	NEG	NEG	NEG	NEG	POS	NEG	NEG	NEG	NEG	NEG	NEG	NEG	NEG	NEG	NEG	NEG	NEG	NEG
UKD-0504920	SCCmec IVb/d/i (21209)	CC8	NEG	NEG	NEG	NEG	NEG	AMB	NEG	NEG	NEG	NEG	NEG	NEG	NEG	NEG	NEG	NEG	NEG	NEG	NEG
UKD-0725999	SCCmecIV b/d/i+Cu	CC30	NEG	NEG	NEG	NEG	NEG	POS	NEG	NEG	NEG	NEG	NEG	NEG	NEG	NEG	NEG	NEG	NEG	NEG	NEG
Prediction for IS-105, GenBank AHLR	*SCCmec IVc (IS-105)*	*CC22*	NEG	NEG	NEG	NEG	NEG	POS	NEG	NEG	NEG	NEG	NEG	NEG	NEG	NEG	NEG	NEG	NEG	NEG	NEG
UKD-0402421	SCCmec IVc (IS-105), merA/B+	CC5	NEG	NEG	NEG	NEG	NEG	POS	NEG	NEG	NEG	NEG	NEG	NEG	NEG	NEG	NEG	POS	NEG	NEG	NEG
UKD-0731319	SCCmec IVc (IS-105), merA/B+	CC5	NEG	NEG	NEG	NEG	NEG	POS	NEG	NEG	NEG	NEG	NEG	NEG	NEG	NEG	NEG	POS	NEG	NEG	NEG
UKD-0733137	SCCmec IVc (IS-105)	CC8	NEG	NEG	NEG	NEG	NEG	POS	NEG	NEG	NEG	NEG	NEG	NEG	NEG	NEG	NEG	NEG	NEG	NEG	NEG
UKD-0305690	SCCmec IVc (IS-105)	CC22	NEG	NEG	NEG	NEG	NEG	POS	NEG	NEG	NEG	NEG	NEG	NEG	NEG	NEG	NEG	NEG	NEG	NEG	NEG
UKD-1424706	SCCmec IVc (IS-105)	CC22	NEG	NEG	NEG	NEG	NEG	POS	NEG	NEG	NEG	NEG	NEG	NEG	NEG	NEG	NEG	NEG	NEG	NEG	NEG
Prediction for TCH60, GenBank CP002110.1	*SCCmec IVc (TCH60)*	*CC30*	NEG	NEG	NEG	NEG	NEG	POS	NEG	NEG	NEG	NEG	NEG	NEG	NEG	NEG	NEG	NEG	NEG	NEG	NEG
**Reference strain JCSC4788**	**SCCmec IVc (TCH60)**	**CC8**	NEG	NEG	NEG	NEG	NEG	POS	NEG	NEG	NEG	NEG	NEG	NEG	NEG	NEG	NEG	NEG	NEG	NEG	NEG
UKD-1318318	SCCmec IVc (TCH60)	CC5	NEG	NEG	NEG	NEG	NEG	POS	NEG	NEG	NEG	NEG	NEG	NEG	NEG	NEG	NEG	NEG	NEG	NEG	NEG
UKD-14580050	SCCmec IVc (TCH60)	CC5	NEG	NEG	NEG	NEG	NEG	POS	NEG	NEG	NEG	NEG	NEG	NEG	NEG	NEG	NEG	NEG	NEG	NEG	NEG
UKD-08F01314	SCCmec IVc (TCH60)	CC8	NEG	NEG	NEG	NEG	NEG	AMB	NEG	NEG	NEG	NEG	NEG	NEG	NEG	NEG	NEG	NEG	NEG	NEG	NEG
KHDN-50361518	SCCmec IVc (TCH60)	CC8	NEG	NEG	NEG	NEG	NEG	POS	neg	NEG	NEG	NEG	NEG	NEG	NEG	NEG	NEG	NEG	neg	NEG	NEG
UKD-0307912	SCCmec IVc (TCH60)	CC22	NEG	NEG	NEG	NEG	NEG	POS	NEG	NEG	NEG	NEG	NEG	NEG	NEG	NEG	NEG	NEG	NEG	NEG	NEG
UKD-0220390	SCCmec IVc (TCH60)	CC45 [agr I]	NEG	NEG	NEG	NEG	NEG	POS	NEG	NEG	NEG	NEG	NEG	NEG	AMB	NEG	NEG	NEG	NEG	NEG	NEG
UKD-0311106	SCCmec IVc (TCH60)	CC45 [agr I]	NEG	NEG	NEG	NEG	NEG	POS	NEG	NEG	NEG	NEG	NEG	NEG	NEG	NEG	NEG	POS	NEG	NEG	NEG
UKD-0719855	SCCmec IVc (TCH60)	CC45 [agr I]	NEG	NEG	NEG	NEG	NEG	POS	NEG	NEG	NEG	NEG	NEG	NEG	NEG	NEG	NEG	NEG	NEG	NEG	NEG
UKD-0422079	SCCmec IVc (TCH60)	CC45 [agr I]	NEG	NEG	NEG	NEG	NEG	POS	NEG	NEG	NEG	NEG	NEG	NEG	NEG	NEG	NEG	NEG	NEG	NEG	NEG
UKD-0409235	SCCmec IVc (TCH60)	CC80	NEG	NEG	NEG	NEG	NEG	POS	NEG	NEG	NEG	NEG	NEG	NEG	NEG	NEG	NEG	NEG	NEG	NEG	NEG
UKD-0409807	SCCmec IVc (TCH60)	CC80	NEG	NEG	NEG	NEG	NEG	POS	NEG	NEG	NEG	NEG	NEG	NEG	AMB	NEG	NEG	NEG	NEG	NEG	NEG
UKD-0416073	SCCmec IVc (TCH60)	CC80	NEG	NEG	NEG	NEG	NEG	POS	NEG	NEG	NEG	NEG	NEG	NEG	NEG	NEG	NEG	NEG	NEG	NEG	NEG
UKD-0431728	SCCmec IVc (TCH60)	CC80	NEG	NEG	NEG	NEG	NEG	POS	NEG	NEG	NEG	NEG	NEG	NEG	AMB	NEG	NEG	NEG	NEG	NEG	NEG
UKD-1001508	SCCmec IVc (TCH60)	CC80	NEG	NEG	NEG	NEG	NEG	POS	NEG	NEG	NEG	NEG	NEG	NEG	AMB	NEG	NEG	NEG	NEG	NEG	NEG
UKD-1010412	SCCmec IVc (TCH60)	CC80	NEG	NEG	NEG	NEG	NEG	POS	NEG	NEG	NEG	NEG	NEG	NEG	NEG	NEG	NEG	NEG	NEG	NEG	NEG
UKD-12753005	SCCmec IVc (TCH60)	CC80	NEG	NEG	NEG	NEG	NEG	POS	NEG	NEG	NEG	NEG	NEG	NEG	NEG	NEG	NEG	NEG	NEG	NEG	NEG
UKD-15574809	SCCmec IVc (TCH60)	CC80	NEG	NEG	NEG	NEG	NEG	POS	NEG	NEG	NEG	NEG	NEG	NEG	NEG	NEG	NEG	NEG	NEG	NEG	NEG
KHDN-16120291	SCCmec IVc (TCH60)	CC88	NEG	NEG	NEG	NEG	NEG	POS	NEG	NEG	NEG	NEG	NEG	NEG	NEG	NEG	NEG	NEG	NEG	NEG	NEG
Prediction for WW2703/97, GenBank ACSW	*SCCmec IVc (WW2703/97)*	*CC30*	NEG	NEG	NEG	NEG	NEG	NEG	NEG	NEG	NEG	POS	NEG	NEG	NEG	NEG	NEG	NEG	NEG	NEG	NEG
UKD-14580088	SCCmec IVc (WW2703/97)	CC398	NEG	NEG	NEG	NEG	NEG	NEG	NEG	NEG	NEG	POS	NEG	NEG	NEG	NEG	NEG	NEG	NEG	NEG	NEG
Prediction for SA_ST125_MupR, GenBank ASTH	*SCCmec IVc+speG+ccrAB4 (SA_ST125)*	*CC5*	NEG	NEG	NEG	POS	POS	POS	NEG	NEG	NEG	NEG	NEG	NEG	AMB	NEG	NEG	NEG	NEG	NEG	NEG
UKD-0439909	SCCmec IVc+speG+ccrAB4 (SA_ST125)	CC5	NEG	NEG	NEG	AMB	POS	POS	NEG	NEG	NEG	NEG	NEG	NEG	POS	NEG	NEG	NEG	NEG	NEG	NEG
Prediction for MRSA177, GenBank AECP	*SCCmec IVc+Cu/Hg (MRSA177)*		NEG	NEG	NEG	NEG	NEG	POS	NEG	NEG	NEG	NEG	NEG	NEG	NEG	NEG	NEG	NEG	NEG	NEG	NEG
UKD-1279734	SCCmec IVc+Cu/Hg (MRSA177)	CC8	NEG	NEG	NEG	NEG	NEG	POS	NEG	NEG	NEG	NEG	NEG	NEG	NEG	NEG	NEG	NEG	NEG	NEG	NEG
UKD-14580899	SCCmec IVc+Cu/Hg (MRSA177)	CC8	NEG	NEG	NEG	NEG	NEG	POS	NEG	NEG	NEG	NEG	NEG	NEG	NEG	NEG	NEG	NEG	NEG	NEG	NEG
UKD-16002228	SCCmec IVc+Cu/Hg (MRSA177)	CC8	NEG	NEG	NEG	NEG	NEG	POS	NEG	NEG	NEG	NEG	NEG	NEG	AMB	NEG	NEG	NEG	NEG	NEG	NEG
Prediction for HO 5096 0412, GenBank HE681097.1	*SCCmec IVh/j (HO50960412/JCSC6670)*	*CC22*	NEG	NEG	NEG	NEG	NEG	POS	NEG	NEG	NEG	NEG	NEG	NEG	NEG	NEG	NEG	NEG	NEG	NEG	NEG
Prediction for JCSC6670, GenBank AB425824.1	*SCCmec IVh/j (HO50960412/JCSC6670)*		NEG	NEG	NEG	NEG	NEG	POS	NEG	NEG	NEG	NEG	NEG	NEG	NEG	NEG	NEG	NEG	NEG	NEG	NEG
**Reference strain Dublin_DSH_E1749**	**SCCmec IVh/j (HO50960412/JCSC6670)**		NEG	NEG	NEG	NEG	NEG	POS	NEG	NEG	NEG	NEG	NEG	NEG	NEG	NEG	NEG	NEG	NEG	NEG	NEG
UKD-14576248	SCCmec IVh/j (HO50960412/JCSC6670)	CC8	NEG	NEG	NEG	NEG	NEG	POS	NEG	NEG	NEG	NEG	NEG	NEG	NEG	NEG	NEG	NEG	NEG	NEG	NEG
UKD-0120122	SCCmec IVh/j (HO50960412/JCSC6670)	CC22	NEG	NEG	NEG	NEG	NEG	POS	NEG	NEG	NEG	NEG	NEG	NEG	NEG	NEG	NEG	NEG	NEG	NEG	NEG
UKD-0205319	SCCmec IVh/j (HO50960412/JCSC6670)	CC22	NEG	NEG	NEG	NEG	NEG	POS	NEG	NEG	NEG	NEG	NEG	NEG	NEG	NEG	NEG	NEG	NEG	NEG	NEG
UKD-0221259	SCCmec IVh/j (HO50960412/JCSC6670)	CC22	NEG	NEG	NEG	NEG	NEG	AMB	NEG	NEG	NEG	NEG	NEG	NEG	NEG	NEG	NEG	NEG	NEG	NEG	NEG
UKD-0418654	SCCmec IVh/j (HO50960412/JCSC6670)	CC22	NEG	NEG	NEG	NEG	NEG	POS	NEG	NEG	NEG	NEG	NEG	NEG	NEG	NEG	NEG	NEG	NEG	NEG	NEG
UKD-0429721	SCCmec IVh/j (HO50960412/JCSC6670)	CC22	NEG	NEG	NEG	NEG	NEG	POS	NEG	NEG	NEG	NEG	NEG	NEG	NEG	NEG	NEG	NEG	NEG	NEG	NEG
UKD-1116194	SCCmec IVh/j (HO50960412/JCSC6670)	CC22	NEG	NEG	NEG	NEG	NEG	POS	NEG	NEG	NEG	NEG	NEG	NEG	NEG	NEG	NEG	NEG	NEG	NEG	NEG
UKD-1122367	SCCmec IVh/j (HO50960412/JCSC6670)	CC22	NEG	NEG	NEG	NEG	NEG	POS	NEG	NEG	NEG	NEG	NEG	NEG	NEG	NEG	NEG	NEG	NEG	NEG	NEG
UKD-14012840	SCCmec IVh/j (HO50960412/JCSC6670)	CC22	NEG	NEG	NEG	NEG	NEG	POS	NEG	NEG	NEG	NEG	NEG	NEG	NEG	NEG	NEG	NEG	NEG	NEG	NEG
UKD-14579833	SCCmec IVh/j (HO50960412/JCSC6670)	CC22	NEG	NEG	NEG	NEG	NEG	POS	NEG	NEG	NEG	NEG	NEG	NEG	NEG	NEG	NEG	NEG	NEG	NEG	NEG
UKD-15579198	SCCmec IVh/j (HO50960412/JCSC6670)	CC22	NEG	NEG	NEG	NEG	NEG	POS	NEG	NEG	NEG	NEG	NEG	NEG	NEG	NEG	NEG	NEG	NEG	NEG	NEG
UKD-4052193	SCCmec IVh/j (HO50960412/JCSC6670)	CC22	NEG	NEG	NEG	NEG	NEG	POS	NEG	NEG	NEG	NEG	NEG	NEG	NEG	NEG	NEG	NEG	NEG	NEG	NEG
UKD-124968	SCCmec IVh/j (HO50960412/JCSC6670)	CC22	NEG	NEG	NEG	NEG	NEG	POS	NEG	NEG	NEG	NEG	NEG	NEG	NEG	NEG	NEG	NEG	NEG	NEG	NEG
UKD-14572546	SCCmec IVh/j (HO50960412/JCSC6670)	CC22	NEG	NEG	NEG	NEG	NEG	POS	NEG	NEG	NEG	NEG	NEG	NEG	NEG	NEG	NEG	NEG	NEG	NEG	NEG
UKD-14582287	SCCmec IVh/j (HO50960412/JCSC6670)	CC22	NEG	NEG	NEG	NEG	NEG	POS	NEG	NEG	NEG	NEG	NEG	NEG	NEG	NEG	NEG	NEG	NEG	NEG	NEG
UKD-14583376	SCCmec IVh/j (HO50960412/JCSC6670)	CC22	NEG	NEG	NEG	NEG	NEG	POS	NEG	NEG	NEG	NEG	NEG	NEG	NEG	NEG	NEG	NEG	NEG	NEG	NEG
UKD-14583496	SCCmec IVh/j (HO50960412/JCSC6670)	CC22	NEG	NEG	NEG	NEG	NEG	POS	NEG	NEG	NEG	NEG	NEG	NEG	NEG	NEG	NEG	NEG	NEG	NEG	NEG
UKD-0013324	SCCmec IVh/j (HO50960412/JCSC6670)	CC22	NEG	NEG	NEG	NEG	NEG	AMB	NEG	NEG	NEG	NEG	NEG	NEG	AMB	NEG	NEG	NEG	NEG	NEG	NEG
UKD-0125329	SCCmec IVh/j (HO50960412/JCSC6670)	CC22	NEG	NEG	NEG	NEG	NEG	POS	NEG	NEG	NEG	NEG	NEG	NEG	AMB	NEG	NEG	NEG	NEG	NEG	NEG
UKD-0215498	SCCmec IVh/j (HO50960412/JCSC6670)	CC22	NEG	NEG	NEG	NEG	NEG	AMB	NEG	NEG	NEG	NEG	NEG	NEG	AMB	NEG	NEG	NEG	NEG	NEG	NEG
Prediction for M08-0126, GenBank FR753166.1	*SCCmec IVh/j+ACME2 (M08-0126)*	*CC22*	NEG	NEG	NEG	NEG	NEG	POS	NEG	NEG	POS	NEG	POS	NEG	NEG	NEG	NEG	NEG	NEG	NEG	NEG
UKD-1379483I	SCCmec IVh/j+ACME2 (M08-0126)	CC22	NEG	NEG	NEG	NEG	NEG	POS	NEG	NEG	AMB	NEG	POS	NEG	NEG	NEG	NEG	NEG	NEG	NEG	NEG
UKD-5150204	SCCmec IV+speG+Cu/czrC+ccrAB4	CC22	NEG	NEG	NEG	POS	POS	POS	NEG	NEG	NEG	NEG	NEG	NEG	AMB	NEG	NEG	NEG	NEG	NEG	NEG
UKD-15578423	SCCmec IV+speG+czrC+ccrAB4 var. 1	CC22	NEG	NEG	NEG	POS	POS	POS	NEG	NEG	NEG	NEG	NEG	NEG	POS	NEG	NEG	NEG	NEG	NEG	NEG
UKD-16582870	SCCmec IV+speG+czrC+ccrAB4 var. 1	CC22	NEG	NEG	NEG	AMB	POS	POS	NEG	NEG	NEG	NEG	NEG	NEG	POS	NEG	NEG	NEG	NEG	NEG	NEG
UKD-15571940	SCCmec IV+speG+czrC+ccrAB4 var. 2	CC22	NEG	NEG	NEG	POS	POS	AMB	NEG	NEG	NEG	NEG	NEG	NEG	POS	NEG	NEG	NEG	NEG	NEG	NEG

Isolate ID	SCCmec type	Clonal complex	METHICILLIN RESISTANCE AND SCCmec TYPING																		
			cassette chromosome recombinase genes					hypothetical protein from SCCmec elements	SCCmec Termini												
			ccrAA (MRSAZH47)_probe 1	ccrAA (MRSAZH47)_probe 2	ccrC (85-2082)	ccrA-4	ccrB-4	Q9XB68-dcs	SCCmec Terminus 1	SCCmec Terminus 2	SCCmec Terminus 3	SCCmec Terminus 4	SCCmec Terminus 5	SCCmec Terminus 6	SCCmec Terminus 7	SCCmec Terminus 9	SCCmec Terminus 10	SCCmec Terminus 11	SCCmec Terminus 12	SCCmec Terminus 13	SCCmec Terminus 14
Prediction for CN1, GenBank CP003979.1	*SCCmec IVA (CN1)*	*CC8 (ST72)*	NEG	NEG	NEG	NEG	NEG	POS	NEG	NEG	NEG	NEG	NEG	NEG	NEG	NEG	NEG	NEG	NEG	NEG	NEG
UKD-0900871	SCCmec IVA (CN1)	CC8 (ST72)	NEG	NEG	NEG	NEG	NEG	POS	NEG	NEG	NEG	NEG	NEG	NEG	NEG	NEG	NEG	NEG	NEG	NEG	NEG
Prediction for CMFT1723, GenBank HF569096.1	*SCCmec V (Bengal Bay)*		POS	POS	POS	NEG	NEG	NEG	NEG	NEG	NEG	NEG	NEG	NEG	NEG	NEG	NEG	POS	NEG	NEG	NEG
KHDN-10405134	SCCmec V (Bengal Bay)	CC1	POS	POS	POS	NEG	NEG	NEG	NEG	NEG	NEG	NEG	NEG	NEG	NEG	NEG	NEG	POS	NEG	NEG	NEG
UKD-0932405	SCCmec V (Bengal Bay)	CC1 (ST573/772)	POS	POS	POS	NEG	NEG	NEG	NEG	NEG	NEG	NEG	NEG	NEG	NEG	NEG	NEG	POS	NEG	NEG	NEG
UKD-1003510	SCCmec V (Bengal Bay)	CC1 (ST573/772)	POS	POS	POS	NEG	NEG	NEG	NEG	NEG	NEG	NEG	NEG	NEG	NEG	NEG	NEG	POS	NEG	NEG	NEG
UKD-1112420	SCCmec V (Bengal Bay)	CC1 (ST573/772)	POS	POS	POS	NEG	NEG	NEG	NEG	NEG	NEG	NEG	NEG	NEG	NEG	NEG	NEG	POS	NEG	NEG	NEG
UKD-1275832	SCCmec V (Bengal Bay)	CC1 (ST573/772)	POS	POS	POS	NEG	NEG	NEG	NEG	NEG	NEG	NEG	NEG	NEG	NEG	NEG	NEG	POS	NEG	NEG	NEG
UKD-13023811	SCCmec V (Bengal Bay)	CC1 (ST573/772)	POS	POS	POS	NEG	NEG	NEG	NEG	NEG	NEG	NEG	NEG	NEG	NEG	NEG	NEG	POS	NEG	NEG	NEG
UKD-14572163	SCCmec V	CC5	POS	POS	POS	NEG	NEG	NEG	NEG	NEG	NEG	NEG	NEG	NEG	NEG	NEG	POS	NEG	NEG	NEG	NEG
KHDN-10421133	SCCmec V+fus	CC97	NEG	POS	POS	NEG	NEG	NEG	NEG	NEG	POS	NEG	NEG	NEG	AMB	NEG	POS	NEG	NEG	NEG	NEG
KHDN-21343541	SCCmec V+fus+tir+ccrAB1	CC1	POS	POS	POS	NEG	NEG	NEG	neg	NEG	POS	NEG	NEG	POS	NEG	NEG	NEG	POS	neg	NEG	NEG
KHDN-10416893	SCCmec V+czrC+speG+ccrAB4	CC22	NEG	NEG	POS	POS	POS	NEG	NEG	NEG	NEG	NEG	NEG	NEG	POS	NEG	NEG	NEG	NEG	NEG	NEG
Prediction for GR1, GenBank AJLX	*SCCmec VT (GR1)*	*CC361*	POS	POS	POS	NEG	NEG	NEG	NEG	POS	NEG	NEG	NEG	NEG	NEG	NEG	NEG	NEG	NEG	NEG	NEG
UKD-0422872	SCCmec VT (GR1)	CC152	POS	POS	POS	NEG	NEG	NEG	NEG	POS	NEG	NEG	NEG	NEG	NEG	NEG	NEG	NEG	NEG	NEG	NEG
Prediction for PM1, GenBank BAFA	*SCCmec VT (PM1)*	*CC59*	POS	POS	POS	NEG	NEG	NEG	NEG	POS	NEG	NEG	NEG	NEG	NEG	NEG	NEG	NEG	NEG	NEG	NEG
KHDN-000871	SCCmec VT (PM1)	CC45 (agr I)	POS	POS	POS	NEG	NEG	NEG	NEG	POS	NEG	NEG	NEG	NEG	NEG	NEG	NEG	NEG	NEG	NEG	NEG
UKD-0725052	SCCmec VT (PM1)	CC59	POS	POS	POS	NEG	NEG	NEG	NEG	POS	NEG	NEG	NEG	NEG	NEG	NEG	NEG	NEG	NEG	NEG	NEG
UKD-1312138	SCCmec VT (PM1)	CC59	POS	POS	POS	NEG	NEG	NEG	NEG	POS	NEG	NEG	NEG	NEG	NEG	NEG	NEG	NEG	NEG	NEG	NEG
UKD-Z4728	SCCmec VT (PM1)	CC59	NEG	POS	POS	NEG	NEG	NEG	NEG	POS	NEG	NEG	NEG	NEG	NEG	NEG	NEG	NEG	NEG	NEG	NEG
UKD-K0199	SCCmec VT unknown var.1	CC7	NEG	NEG	POS	NEG	NEG	NEG	NEG	POS	NEG	NEG	NEG	NEG	NEG	NEG	NEG	NEG	NEG	NEG	NEG
UKD-15584619	SCCmec VT unknown var.2	CC30	NEG	POS	POS	NEG	NEG	NEG	NEG	POS	NEG	NEG	NEG	NEG	NEG	NEG	NEG	NEG	NEG	NEG	NEG
UKD-0712568	SCCmec VT unknown var.3	CC45 (agr I)	POS	POS	POS	NEG	NEG	NEG	NEG	POS	NEG	NEG	POS	NEG	NEG	NEG	NEG	NEG	NEG	NEG	NEG
KHDN-10420296	SCCmec VT+ACME2	CC45 (agr I)	NEG	POS	POS	NEG	NEG	NEG	NEG	POS	NEG	NEG	NEG	NEG	NEG	NEG	NEG	NEG	NEG	NEG	NEG
UKD-15577956	SCCmec VT+Cu/czrC+ccrA1	CC398	POS	POS	POS	NEG	NEG	NEG	NEG	POS	NEG	NEG	NEG	NEG	POS	NEG	NEG	NEG	NEG	NEG	NEG
Prediction for S0385, GenBank AM990992	*SCCmec VT+czrC (SO385)*	*CC398*	POS	POS	POS	NEG	NEG	NEG	NEG	POS	NEG	NEG	NEG	NEG	NEG	NEG	NEG	NEG	NEG	NEG	NEG
UKD-0628560	SCCmec VT+czrC (SO385)	CC8	POS	POS	POS	NEG	NEG	NEG	NEG	POS	NEG	NEG	NEG	NEG	NEG	NEG	NEG	NEG	NEG	NEG	NEG
UKD-0510018	SCCmec VT+czrC (SO385)	CC398	POS	POS	POS	NEG	NEG	NEG	NEG	POS	NEG	NEG	NEG	NEG	NEG	NEG	NEG	NEG	NEG	NEG	NEG
UKD-0903558	SCCmec VT+czrC (SO385)	CC398	POS	POS	POS	NEG	NEG	NEG	NEG	POS	NEG	NEG	NEG	NEG	NEG	NEG	NEG	NEG	NEG	NEG	NEG
UKD-0929815	SCCmec VT+czrC (SO385)	CC398	POS	POS	POS	NEG	NEG	NEG	NEG	POS	NEG	NEG	NEG	NEG	NEG	NEG	NEG	NEG	NEG	NEG	NEG
UKD-0907762	SCCmec VT+czrC (SO385)	CC398	POS	POS	POS	NEG	NEG	NEG	NEG	POS	NEG	NEG	NEG	NEG	NEG	NEG	NEG	NEG	NEG	NEG	NEG
UKD-1025105	SCCmec VT+czrC (SO385)	CC398	POS	POS	POS	NEG	NEG	NEG	NEG	POS	NEG	NEG	NEG	NEG	NEG	NEG	NEG	NEG	NEG	NEG	NEG
UKD-11780898	SCCmec VT+czrC (SO385)	CC398	POS	POS	POS	NEG	NEG	NEG	NEG	POS	NEG	NEG	NEG	NEG	NEG	NEG	NEG	NEG	NEG	NEG	NEG
UKD-1377009	SCCmec VT+czrC (SO385)	CC398	POS	POS	POS	NEG	NEG	NEG	NEG	POS	NEG	NEG	NEG	NEG	NEG	NEG	NEG	NEG	NEG	NEG	NEG
UKD-143406	SCCmec VT+czrC (SO385)	CC398	POS	POS	POS	NEG	NEG	NEG	NEG	POS	NEG	NEG	NEG	NEG	NEG	NEG	NEG	NEG	NEG	NEG	NEG
UKD-1400363	SCCmec VT+czrC (SO385)	CC398	POS	POS	POS	NEG	NEG	NEG	NEG	POS	NEG	NEG	NEG	NEG	NEG	NEG	NEG	NEG	NEG	NEG	NEG
UKD-14571852	SCCmec VT+czrC (SO385)	CC398	POS	POS	POS	NEG	NEG	NEG	NEG	POS	NEG	NEG	NEG	NEG	NEG	NEG	NEG	NEG	NEG	NEG	NEG
UKD-14582574	SCCmec VT+czrC (SO385)	CC398	POS	POS	POS	NEG	NEG	NEG	NEG	POS	NEG	NEG	NEG	NEG	NEG	NEG	NEG	NEG	NEG	NEG	NEG
UKD-152108	SCCmec VT+czrC (SO385)	CC398	POS	POS	POS	NEG	NEG	NEG	NEG	POS	NEG	NEG	NEG	NEG	NEG	NEG	NEG	NEG	NEG	NEG	NEG
UKD-15573321	SCCmec VT+czrC (SO385)	CC398	POS	POS	POS	NEG	NEG	NEG	NEG	POS	NEG	NEG	NEG	NEG	NEG	NEG	NEG	NEG	NEG	NEG	NEG
UKD-15584034	SCCmec VT+czrC , ydhK-neg. variant	CC398	POS	POS	POS	NEG	NEG	NEG	NEG	POS	NEG	NEG	NEG	NEG	NEG	NEG	NEG	NEG	NEG	NEG	NEG
UKD-0932581	SCCmecVT+fus+czrC	CC22	POS	POS	POS	NEG	NEG	NEG	NEG	POS	NEG	NEG	NEG	NEG	NEG	NEG	NEG	NEG	POS	NEG	NEG
UKD-1121994	SCCmecVT+fus+czrC	CC22	POS	POS	POS	NEG	NEG	NEG	NEG	POS	NEG	NEG	NEG	NEG	NEG	NEG	NEG	NEG	POS	NEG	NEG
UKD-1272252	SCCmecVT+fus+czrC	CC22	POS	POS	POS	NEG	NEG	NEG	NEG	POS	NEG	NEG	NEG	NEG	NEG	NEG	NEG	NEG	POS	NEG	NEG
Prediction for MRSA18	*SCCmec VI+fus (MRSA18)*		NEG	NEG	NEG	NEG	POS	NEG	NEG	NEG	NEG	NEG	NEG	NEG	POS	NEG	NEG	NEG	NEG	NEG	NEG
UKD-1103380	SCCmec VI+fus (MRSA18)	CC7	NEG	NEG	NEG	NEG	POS	NEG	NEG	NEG	NEG	NEG	NEG	NEG	POS	NEG	NEG	NEG	NEG	NEG	NEG
Prediction for M10/0061, GenBank FR823292.1	*SCCmec XI (LGA251/M10-61)*	*CC130*	NEG	NEG	NEG	NEG	NEG	NEG	NEG	NEG	NEG	NEG	NEG	NEG	NEG	NEG	NEG	NEG	NEG	NEG	NEG
Reference strain Dublin-DSH_M10 0061	SCCmec XI (LGA251/M10-61)	CC130	NEG	NEG	NEG	NEG	NEG	NEG	NEG	NEG	NEG	NEG	NEG	NEG	NEG	NEG	NEG	NEG	NEG	NEG	NEG
Prediction for LGA251, GenBank FR821779.1	*SCCmec XI (LGA251/M10-61)*	*ST425*	NEG	NEG	NEG	NEG	NEG	NEG	NEG	NEG	NEG	NEG	NEG	NEG	NEG	NEG	NEG	NEG	NEG	NEG	NEG
UKD-14015835	SCCmec XI (LGA251/M10-61)	CC130	NEG	NEG	NEG	NEG	NEG	NEG	NEG	NEG	NEG	NEG	NEG	NEG	NEG	NEG	NEG	NEG	NEG	NEG	NEG
UKD-16573252	SCCmec XI (LGA251/M10-61)	CC130	NEG	NEG	NEG	NEG	NEG	NEG	NEG	NEG	NEG	NEG	NEG	NEG	NEG	NEG	NEG	NEG	NEG	NEG	NEG
UKD-0217002	PseudoSCCmec, class B mec complex+Hg	CC8	NEG	NEG	NEG	NEG	NEG	POS	NEG	NEG	NEG	NEG	NEG	NEG	NEG	NEG	NEG	NEG	NEG	NEG	NEG
UKD-0008510	PseudoSCCmec, class B mec complex+Hg	CC8	NEG	NEG	NEG	NEG	NEG	POS	NEG	NEG	NEG	NEG	NEG	NEG	NEG	NEG	NEG	NEG	NEG	NEG	NEG
UKD-0110962	PseudoSCCmec, class B mec complex+Hg	CC8	NEG	NEG	NEG	NEG	NEG	POS	NEG	NEG	NEG	NEG	NEG	NEG	NEG	NEG	NEG	NEG	NEG	NEG	NEG
KHDN-10408593	PseudoSCCmec, class B mec complex+Cu/Hg	CC8	NEG	NEG	NEG	NEG	NEG	POS	neg	NEG	NEG	NEG	NEG	NEG	NEG	NEG	NEG	NEG	neg	NEG	NEG
UKD-0007201	PseudoSCCmec, class B mec complex	CC5	NEG	NEG	NEG	NEG	NEG	POS	NEG	NEG	NEG	NEG	NEG	NEG	NEG	NEG	NEG	NEG	NEG	NEG	NEG
UKD-0033204	PseudoSCCmec, class B mec complex	CC5	NEG	NEG	NEG	NEG	NEG	POS	NEG	NEG	NEG	NEG	NEG	NEG	NEG	NEG	NEG	NEG	NEG	NEG	NEG
UKD-0134329	PseudoSCCmec, class B mec complex	CC5	NEG	NEG	NEG	NEG	NEG	POS	NEG	NEG	NEG	NEG	NEG	NEG	NEG	NEG	NEG	NEG	NEG	NEG	NEG
UKD-0806946	PseudoSCCmec class C+As/Cu	CC398	NEG	NEG	NEG	NEG	NEG	NEG	NEG	NEG	NEG	NEG	NEG	NEG	NEG	NEG	POS	NEG	NEG	NEG	NEG
UKD-0500610	ACME2	CC8	NEG	NEG	NEG	NEG	NEG	NEG	NEG	NEG	POS	NEG	POS	NEG	NEG	NEG	NEG	NEG	NEG	NEG	NEG
UKD-0610438	ACME2	CC8	NEG	NEG	NEG	NEG	NEG	NEG	NEG	NEG	AMB	NEG	POS	NEG	NEG	NEG	NEG	NEG	NEG	NEG	NEG
KHDN-10409344	ACME2	CC8	NEG	NEG	NEG	NEG	NEG	NEG	NEG	NEG	POS	NEG	POS	NEG	NEG	NEG	NEG	NEG	NEG	NEG	NEG
UKD-0322559	ACME2+speG+czrC+ccrAB4	CC8	NEG	NEG	NEG	AMB	POS	NEG	NEG	NEG	POS	NEG	POS	NEG	POS	NEG	NEG	NEG	NEG	NEG	NEG
UKD-0501998	ACME2+speG+czrC+ccrAB4	CC8	NEG	NEG	NEG	NEG	POS	NEG	NEG	NEG	AMB	NEG	POS	NEG	AMB	NEG	NEG	NEG	NEG	NEG	NEG
UKD-0505213	ACME2+speG+czrC+ccrAB4	CC8	NEG	NEG	NEG	POS	POS	NEG	NEG	NEG	POS	NEG	NEG	NEG	POS	NEG	AMB	NEG	NEG	NEG	NEG
Prediction for MSSA476, GenBank BX571857.1	*fus+tir+ccrAB1 (MSSA476)*	*CC1*	NEG	NEG	NEG	NEG	NEG	NEG	NEG	NEG	POS	NEG	NEG	POS	NEG	NEG	NEG	NEG	NEG	NEG	NEG
Reference strain Sanger 476	fus+tir+ccrAB1 (MSSA476)	CC1	NEG	NEG	NEG	NEG	NEG	NEG	NEG	NEG	AMB	NEG	NEG	POS	NEG	NEG	NEG	NEG	NEG	NEG	NEG
UKD-14023914	fus+tir+ccrAB1 (MSSA476)	CC1	NEG	NEG	NEG	NEG	NEG	NEG	NEG	NEG	POS	NEG	NEG	POS	NEG	NEG	NEG	NEG	NEG	NEG	NEG
UKD-K00034	kdp+ccrAB2	CC182	NEG	NEG	NEG	NEG	NEG	NEG	NEG	NEG	NEG	NEG	NEG	NEG	NEG	NEG	NEG	NEG	NEG	NEG	NEG
UKD-15580369	As+ccrAB4	CC22	NEG	NEG	NEG	POS	POS	AMB	NEG	NEG	NEG	NEG	NEG	NEG	NEG	NEG	NEG	NEG	NEG	NEG	NEG
UKD-15570100	ACME3+ccrAB1 (Strain 21342)	CC80	NEG	NEG	NEG	NEG	NEG	NEG	NEG	NEG	NEG	NEG	NEG	NEG	POS	NEG	NEG	NEG	NEG	NEG	NEG
UKD-1010678	speG+czrC+ccrAB4	CC8	NEG	NEG	NEG	NEG	POS	NEG	NEG	NEG	NEG	NEG	NEG	NEG	POS	NEG	NEG	NEG	NEG	NEG	NEG
UKD-1008243	speG+czrC+ccrAB4	CC8	NEG	NEG	NEG	POS	POS	NEG	NEG	NEG	NEG	NEG	NEG	NEG	POS	NEG	NEG	NEG	NEG	NEG	NEG
UKD-1009430	speG+czrC+ccrAB4	CC8	NEG	NEG	NEG	NEG	POS	NEG	NEG	NEG	NEG	NEG	NEG	NEG	POS	NEG	NEG	NEG	NEG	NEG	NEG
UKD-12071828	speG+czrC+ccrAB4	CC22	NEG	NEG	NEG	POS	POS	NEG	NEG	NEG	NEG	NEG	NEG	NEG	POS	NEG	NEG	NEG	NEG	NEG	NEG
UKD-14011883	speG+czrC+ccrAB4	CC22	NEG	NEG	NEG	POS	POS	NEG	NEG	NEG	NEG	NEG	NEG	NEG	POS	NEG	NEG	NEG	NEG	NEG	NEG
UKD-15008293	speG+czrC+ccrAB4	CC22	NEG	NEG	NEG	POS	POS	NEG	NEG	NEG	NEG	NEG	NEG	NEG	POS	NEG	NEG	NEG	NEG	NEG	NEG
UKD-1514877	speG+czrC+ccrAB4	CC22	NEG	NEG	NEG	POS	POS	NEG	NEG	NEG	NEG	NEG	NEG	NEG	POS	NEG	NEG	NEG	NEG	NEG	NEG
UKD-15580971	speG+czrC+ccrAB4	CC22	NEG	NEG	NEG	POS	POS	NEG	NEG	NEG	NEG	NEG	NEG	NEG	POS	NEG	NEG	NEG	NEG	NEG	NEG
UKD-15582646	speG+czrC+ccrAB4	CC22	NEG	NEG	NEG	POS	POS	NEG	NEG	NEG	NEG	NEG	NEG	NEG	POS	NEG	NEG	NEG	NEG	NEG	NEG

Isolate ID	SCCmec type	Clonal complex	HEAVY METAL RESISTANCES (SCC-associated)										
			mercury resistance operon		copper exporting ATPase	Multi copper oxidase	Arsenical pump membrane protein arsB (SCC)			Carbamate kinase	cadmium and zinc resistance gene C (formerly known as copA)	Cadmium transport protein D	Putative regulator of cadmium efflux
			merA	merB	copA2_SCC	mco-SCC	hp1478_arsB	hp1536_arsB	hp1526_arsB	arsC (SCC or plasmidic)	czrC	cadD (R35)	cadX (JCSC6943)
VIRTUAL HYBRIDISATION for COL, GenBank CP000046	SCCmec I (COL)	CC8	NEG	NEG	NEG	NEG	NEG	NEG	NEG	NEG	NEG	NEG	NEG
Reference strain COL	SCCmec I (COL)	CC8	NEG	NEG	NEG	NEG	NEG	NEG	NEG	NEG	NEG	NEG	NEG
UKD-0007063	SCCmec I (COL)	CC5	POS	POS	NEG	NEG	NEG	NEG	NEG	NEG	NEG	NEG	NEG
UKD-0220759	SCCmec I (COL)	CC5	POS	POS	NEG	NEG	NEG	NEG	NEG	NEG	NEG	NEG	NEG
UKD-0308986	SCCmec I (COL)	CC5	NEG	NEG	NEG	NEG	NEG	NEG	NEG	NEG	NEG	NEG	NEG
UKD-0500009	SCCmec I (COL)	CC5	POS	POS	NEG	NEG	NEG	NEG	NEG	NEG	NEG	NEG	NEG
UKD-0508483	SCCmec I (COL)	CC5	POS	POS	NEG	NEG	NEG	NEG	NEG	NEG	NEG	NEG	NEG
UKD-0519001	SCCmec I (COL)	CC5	NEG	NEG	NEG	NEG	NEG	NEG	NEG	NEG	NEG	NEG	NEG
UKD-0701876	SCCmec I (COL)	CC5	POS	POS	NEG	NEG	NEG	NEG	NEG	NEG	NEG	NEG	NEG
UKD-0708865	SCCmec I (COL)	CC5	NEG	NEG	NEG	NEG	NEG	NEG	NEG	NEG	NEG	NEG	NEG
UKD-1001782	SCCmec I (COL)	CC5	NEG	NEG	NEG	NEG	NEG	NEG	NEG	NEG	NEG	NEG	NEG
Prediction for PSP1996, GenBank ANHU	SCCmec I (PSP1996)	CC8	POS	POS	NEG	NEG	NEG	NEG	NEG	NEG	NEG	NEG	NEG
UKD-0261727	SCCmec I (PSP1996)	CC8	NEG	NEG	NEG	NEG	NEG	NEG	NEG	NEG	NEG	NEG	NEG
VIRTUAL HYBRIDISATION for MRSA7 : VIRTUAL HYBRIL	SCCmec I+fus+tirS (Geraldine Clone)	CC5	NEG	NEG	NEG	NEG	NEG	NEG	NEG	NEG	NEG	NEG	NEG
UKD-1221930	SCCmec I+fus+tirS (Geraldine Clone)	CC5	NEG	NEG	NEG	NEG	NEG	NEG	NEG	NEG	NEG	NEG	NEG
Prediction for N315, GenBank BA000018.3	SCCmec II (N315)	CC5	NEG	NEG	NEG	NEG	NEG	NEG	NEG	NEG	NEG	NEG	NEG
Reference strain N315	SCCmec II (N315)	CC5	NEG	NEG	NEG	NEG	NEG	NEG	NEG	NEG	NEG	NEG	NEG
Prediction for MRSA252, GenBank BX571856.1	SCCmec II (N315)	CC30	NEG	NEG	NEG	NEG	NEG	NEG	NEG	NEG	NEG	NEG	NEG
Reference strain Sanger 252	SCCmec II (N315)	CC30	NEG	NEG	NEG	NEG	NEG	NEG	NEG	NEG	NEG	NEG	NEG
UKD-0230792	SCCmec II (N315)	CC30	NEG	NEG	NEG	NEG	NEG	NEG	NEG	NEG	NEG	NEG	NEG
Prediction for Mu50, GenBank BA000017.4	SCCmec II (N315)	CC5	NEG	NEG	NEG	NEG	NEG	NEG	NEG	NEG	NEG	NEG	NEG
Reference strain MU50	SCCmec II (N315)	CC5	NEG	NEG	NEG	NEG	NEG	NEG	NEG	NEG	NEG	NEG	NEG
Prediction for JH1, GenBank CP000736.1	SCCmec II (JH1/JH9)	CC5	NEG	NEG	NEG	NEG	NEG	NEG	NEG	NEG	NEG	NEG	NEG
Prediction for JH9, GenBank CP000703.1	SCCmec II (JH1/JH9)	CC5	NEG	NEG	NEG	NEG	NEG	NEG	NEG	NEG	NEG	NEG	NEG
Reference strain Dublin-DSH_AR07.4_0237	SCCmec II (JH1/JH9)		NEG	NEG	NEG	NEG	NEG	NEG	NEG	NEG	NEG	NEG	NEG
Reference strain NARSA_382 / USA100	SCCmec II (JH1/JH9)	CC5	NEG	NEG	NEG	NEG	NEG	NEG	NEG	NEG	NEG	NEG	NEG
UKD-0046921	SCCmec II (JH1/JH9)	CC5	NEG	NEG	NEG	NEG	NEG	NEG	NEG	NEG	NEG	NEG	NEG
UKD-0333415	SCCmec II (JH1/JH9)	CC5	NEG	NEG	NEG	NEG	NEG	NEG	NEG	NEG	NEG	NEG	NEG
UKD-0341645	SCCmec II (JH1/JH9)	CC5	NEG	NEG	NEG	NEG	NEG	NEG	NEG	NEG	NEG	NEG	NEG
UKD-0504626	SCCmec II (JH1/JH9)	CC5	NEG	NEG	NEG	NEG	NEG	NEG	NEG	NEG	NEG	NEG	NEG
UKD-0507581	SCCmec II (JH1/JH9)	CC5	NEG	NEG	NEG	NEG	NEG	NEG	NEG	NEG	NEG	NEG	NEG
UKD-0507903	SCCmec II (JH1/JH9)	CC5	NEG	NEG	NEG	NEG	NEG	NEG	NEG	NEG	NEG	NEG	NEG
UKD-0700817	SCCmec II (JH1/JH9)	CC5	NEG	NEG	NEG	NEG	NEG	NEG	NEG	NEG	NEG	NEG	NEG
UKD-1014561	SCCmec II (JH1/JH9)	CC5	NEG	NEG	NEG	NEG	NEG	NEG	NEG	NEG	NEG	NEG	NEG
UKD-1037507	SCCmec II (JH1/JH9)	CC5	NEG	NEG	NEG	NEG	NEG	NEG	NEG	NEG	NEG	NEG	NEG
UKD-1038321	SCCmec II (JH1/JH9)	CC5	NEG	NEG	NEG	NEG	NEG	NEG	NEG	NEG	NEG	NEG	NEG
UKD-1111608	SCCmec II (JH1/JH9)	CC5	NEG	NEG	NEG	NEG	NEG	NEG	NEG	NEG	NEG	NEG	NEG
UKD-12753135	SCCmec II (JH1/JH9)	CC5	NEG	NEG	NEG	NEG	NEG	NEG	NEG	NEG	NEG	NEG	NEG
UKD-1312816	SCCmec II (JH1/JH9)	CC5	NEG	NEG	NEG	NEG	NEG	NEG	NEG	NEG	NEG	NEG	neg
UKD-1401623	SCCmec II (JH1/JH9)	CC5	NEG	NEG	NEG	NEG	NEG	NEG	NEG	NEG	NEG	NEG	NEG
UKD-15571943	SCCmec II+czrC+ccrA/B-4	CC5	NEG	NEG	NEG	NEG	AMB	NEG	NEG	NEG	POS	NEG	NEG
UKD-15576888	SCCmec II+czrC+ccrA/B-4	CC5	NEG	NEG	NEG	NEG	NEG	NEG	NEG	NEG	POS	NEG	NEG
UKD-14576461	SCCmec II+speG+czrC+ccrA/B-4	CC5	NEG	NEG	NEG	NEG	NEG	NEG	NEG	NEG	POS	NEG	NEG
UKD-15581649	SCCmec II+speG+czrC+ccrA/B-4	CC5	NEG	NEG	NEG	NEG	NEG	NEG	NEG	NEG	POS	NEG	NEG
Prediction for CIGC345D, GenBank AHVO	SCCmec II A/B/D	CC8	NEG	NEG	NEG	NEG	NEG	NEG	NEG	NEG	NEG	NEG	NEG
UKD-12K00171	SCCmec II A/B/D	CC8	NEG	NEG	NEG	NEG	NEG	NEG	NEG	NEG	NEG	NEG	NEG
Prediction for JKD6008, GenBank CP002120.1	SCC [mec III+Cd] (JKD6008)	CC8 (ST239)	NEG	NEG	NEG	NEG	NEG	NEG	NEG	NEG	NEG	POS	NEG
Prediction for JKD6009, GenBank ABSA	SCC [mec III+Cd] (JKD6008)	CC8 (ST239)	NEG	NEG	NEG	NEG	NEG	NEG	NEG	NEG	NEG	POS	NEG
Reference strain ATCC_33592	SCC [mec III+Cd] (ATCC33592)	CC8 (ST239)	NEG	NEG	NEG	NEG	NEG	NEG	NEG	NEG	NEG	POS	NEG
Prediction for PPUKM-775-2009, GenBank AMRE	SCC [mec III+Cd+ccrC] (PPUKM-775-2009)	CC8 (ST239)	NEG	NEG	NEG	NEG	NEG	NEG	NEG	NEG	NEG	POS	NEG
Prediction for T0131, GenBank CP002643.1	SCC [mec III+Cd+ccrC] (T0131)	CC8 (ST239)	NEG	NEG	NEG	NEG	NEG	NEG	NEG	NEG	NEG	POS	NEG
Prediction for XN108, GenBank CP007447.1	SCC [mec III+Cd+ccrC] (XN108)	CC8 (ST239)	NEG	NEG	NEG	NEG	NEG	NEG	NEG	NEG	NEG	POS	NEG
Prediction for CN79, GenBank ANCJ	SCC [mec III+Cd+ccrC] (CN79)	CC8 (ST239)	NEG	NEG	NEG	NEG	NEG	NEG	NEG	NEG	NEG	POS	NEG
Prediction for 16K, GenBank BABZ	SCC [mec III+Cd+ccrC] (CN79)	CC8 (ST239)	NEG	NEG	NEG	NEG	NEG	NEG	NEG	NEG	NEG	POS	NEG
UKD-0835987	SCC [mec III+Cd+ccrC] (CN79)	CC8 (ST239)	NEG	NEG	NEG	NEG	NEG	NEG	NEG	NEG	NEG	POS	NEG
UKD-16581769	SCCmec III+Cd+ccrC	CC8 (ST239)	NEG	NEG	NEG	NEG	NEG	NEG	NEG	NEG	NEG	POS	NEG
Prediction for Bmb9393, GenBank CP005288.1	SCCmec III+Cd/Hg+ccrC (Bmb9393)	CC8 (ST239)	POS	POS	NEG	NEG	NEG	NEG	NEG	NEG	NEG	POS	NEG
UKD-15574263	SCCmec III+Cd/Hg+ccrC (Bmb9393)	CC8 (ST239)	POS	POS	NEG	NEG	NEG	NEG	NEG	NEG	NEG	POS	NEG
Prediction for SK1585, GenBank AYLT	SCCmec III+ccrC+Cd/Hg (SK1585)	CC8 (ST239)	POS	POS	NEG	NEG	NEG	NEG	NEG	NEG	NEG	POS	NEG
UKD-105861	SCCmec III+ccrC+Cd/Hg (SK1585)	CC8 (ST239)	POS	POS	NEG	NEG	NEG	NEG	NEG	NEG	NEG	POS	NEG
UKD-134572	SCCmec III+ccrC+Cd/Hg (SK1585)	CC8 (ST239)	POS	POS	NEG	NEG	NEG	NEG	NEG	NEG	NEG	POS	NEG
UKD-139143	SCCmec III+ccrC+Cd/Hg (SK1585)	CC8 (ST239)	POS	POS	NEG	NEG	NEG	NEG	NEG	NEG	NEG	POS	NEG
Prediction for TW20, GenBank FN433596.1	SCC [mec III+Cd/Hg+ccrC] (TW20)	CC8 (ST239)	POS	POS	NEG	NEG	NEG	NEG	NEG	NEG	NEG	POS	NEG
Prediction for ATCC BAA-39, GenBank AEEK	SCC [mec III+Cd/Hg+ccrC] (TW20)	CC8 (ST239)	POS	POS	NEG	NEG	NEG	NEG	NEG	NEG	NEG	POS	NEG

Isolate ID	SCCmec type	Clonal complex	HEAVY METAL RESISTANCES (SCC-associated)										
			mercury resistance operon		copper exporting ATPase	Multi copper oxidase	Arsenical pump membrane protein arsB (SCC)			Carbamate kinase	cadmium and zinc resistance gene C (formerly known as copA)	Cadmium transport protein D	Putative regulator of cadmium efflux
			merA	merB	copA2_SCC	mco-SCC	hp1478_ arsB	hp1536_ arsB	hp1526_ arsB	arsC (SCC or plasmidic)	czrC	cadD (R35)	cadX (JCSC6943)
Prediction for MW2, GenBank BA000033.2	SCCmec IVa (MW2)	CC1	NEG	NEG	NEG	NEG	NEG	NEG	NEG	NEG	NEG	NEG	NEG
Reference strain MW2	SCCmec IVa (MW2)	CC1	NEG	NEG	NEG	NEG	NEG	NEG	NEG	NEG	NEG	NEG	NEG
Reference strain CA05 (JCSC1968)	SCCmec IVa (MW2)	CC45	NEG	NEG	NEG	NEG	NEG	NEG	NEG	NEG	NEG	NEG	NEG
UKD-0127106	SCCmec IVa (MW2)	CC1	NEG	NEG	NEG	NEG	NEG	NEG	NEG	NEG	NEG	NEG	NEG
UKD-0701446	SCCmec IVa (MW2)	CC1	NEG	NEG	NEG	NEG	NEG	NEG	NEG	NEG	NEG	NEG	NEG
UKD-0947486	SCCmec IVa (MW2)	CC1	NEG	NEG	NEG	NEG	NEG	NEG	NEG	NEG	NEG	NEG	NEG
UKD-1031910	SCCmec IVa (MW2)	CC1	NEG	NEG	NEG	NEG	NEG	NEG	NEG	NEG	NEG	NEG	NEG
UKD-14571328	SCCmec IVa (MW2)	CC1	NEG	NEG	NEG	NEG	NEG	NEG	NEG	NEG	NEG	NEG	NEG
UKD-03003012	SCCmec IVa (MW2)	CC5	NEG	NEG	NEG	NEG	NEG	NEG	NEG	NEG	NEG	NEG	NEG
UKD-15582582	SCCmec IVa (MW2)	CC6	NEG	NEG	NEG	NEG	NEG	NEG	NEG	NEG	NEG	NEG	NEG
UKD-16574230	SCCmec IVa (MW2)	CC6	NEG	NEG	NEG	NEG	NEG	NEG	NEG	NEG	NEG	NEG	NEG
UKD-16582560	SCCmec IVa (MW2)	CC6	NEG	NEG	NEG	NEG	NEG	NEG	NEG	NEG	NEG	NEG	NEG
UKD-0802855	SCCmec IVa (MW2)	CC7	NEG	NEG	NEG	NEG	NEG	NEG	NEG	NEG	NEG	NEG	NEG
KHDN-10405258	SCCmec IVa (MW2)	CC8	NEG	NEG	NEG	NEG	NEG	NEG	NEG	NEG	NEG	NEG	NEG
UKD-15573497	SCCmec IVa (MW2)	CC22	NEG	NEG	NEG	NEG	NEG	NEG	NEG	NEG	NEG	NEG	NEG
UKD-0634729	SCCmec IVa (MW2)	CC30	NEG	NEG	NEG	NEG	NEG	NEG	NEG	NEG	NEG	NEG	NEG
UKD-1119771	SCCmec IVa (MW2)	CC30	NEG	NEG	NEG	NEG	NEG	NEG	NEG	NEG	NEG	NEG	NEG
KHDN-10411458	SCCmec IVa (MW2)	CC30	NEG	NEG	NEG	NEG	NEG	NEG	NEG	NEG	NEG	NEG	neg
KHDN-398609	SCCmec IVa (MW2)	CC30	NEG	NEG	NEG	NEG	NEG	NEG	NEG	NEG	NEG	NEG	NEG
KHDN-01081998	SCCmec IVa (MW2)	CC30	NEG	NEG	NEG	NEG	NEG	NEG	NEG	NEG	NEG	NEG	NEG
KHDN-10399223	SCCmec IVa (MW2)	CC30	NEG	NEG	NEG	NEG	NEG	NEG	NEG	NEG	NEG	NEG	NEG
KHDN-W261158	SCCmec IVa (MW2)	CC30	NEG	NEG	NEG	NEG	NEG	NEG	NEG	NEG	NEG	NEG	neg
UKD-0002046	SCCmec IVa (MW2)	CC45 [agr I]	NEG	NEG	NEG	NEG	NEG	NEG	NEG	NEG	NEG	NEG	NEG
UKD-0029618	SCCmec IVa (MW2)	CC45 [agr I]	NEG	NEG	NEG	NEG	NEG	NEG	NEG	NEG	NEG	NEG	NEG
UKD-0043085	SCCmec IVa (MW2)	CC45 [agr I]	NEG	NEG	NEG	NEG	NEG	NEG	NEG	NEG	NEG	NEG	NEG
UKD-0047053	SCCmec IVa (MW2)	CC45 [agr I]	NEG	NEG	NEG	NEG	NEG	NEG	NEG	NEG	NEG	NEG	NEG
UKD-0047429	SCCmec IVa (MW2)	CC45 [agr I]	NEG	NEG	NEG	NEG	NEG	NEG	NEG	NEG	NEG	NEG	NEG
UKD-0112465	SCCmec IVa (MW2)	CC45 [agr I]	NEG	NEG	NEG	NEG	NEG	NEG	NEG	NEG	NEG	NEG	NEG
UKD-0103483	SCCmec IVa (MW2)	CC45 [agr I]	NEG	NEG	NEG	NEG	NEG	NEG	NEG	NEG	NEG	NEG	NEG
UKD-0305014	SCCmec IVa (MW2)	CC45 [agr I]	NEG	NEG	NEG	NEG	NEG	NEG	NEG	NEG	NEG	NEG	NEG
UKD-0401119	SCCmec IVa (MW2)	CC45 [agr I]	NEG	NEG	NEG	NEG	NEG	NEG	NEG	NEG	NEG	NEG	NEG
UKD-0537969	SCCmec IVa (MW2)	CC45 [agr I]	NEG	NEG	NEG	NEG	NEG	NEG	NEG	NEG	NEG	NEG	NEG
UKD-0703373	SCCmec IVa (MW2)	CC45 [agr I]	NEG	NEG	NEG	NEG	NEG	NEG	NEG	NEG	NEG	NEG	NEG
UKD-0813653	SCCmec IVa (MW2)	CC45 [agr I]	NEG	NEG	NEG	NEG	NEG	NEG	NEG	NEG	NEG	NEG	NEG
UKD-0839175	SCCmec IVa (MW2)	CC45 [agr I]	NEG	NEG	NEG	NEG	NEG	NEG	NEG	NEG	NEG	NEG	NEG
UKD-1108422	SCCmec IVa (MW2)	CC45 [agr I]	NEG	NEG	NEG	NEG	NEG	NEG	NEG	NEG	NEG	NEG	NEG
UKD-1380645	SCCmec IVa (MW2)	CC45 [agr I]	NEG	NEG	NEG	NEG	NEG	NEG	NEG	NEG	NEG	NEG	NEG
UKD-148016	SCCmec IVa (MW2)	CC45 [agr I]	NEG	NEG	NEG	NEG	NEG	NEG	NEG	NEG	NEG	NEG	NEG
UKD-16571773	SCCmec IVa (MW2)	CC45 [agr I]	NEG	NEG	NEG	NEG	NEG	NEG	NEG	NEG	NEG	NEG	NEG
KHDN-10407738	SCCmec IVa (MW2)	CC45 [ST617]	NEG	NEG	NEG	NEG	NEG	NEG	NEG	NEG	NEG	NEG	NEG
UKD-0853670	SCCmec IVa (MW2)	CC88	NEG	NEG	NEG	NEG	NEG	NEG	NEG	NEG	NEG	NEG	NEG
UKD-1417352	SCCmec IVa (MW2)	CC88	NEG	NEG	NEG	NEG	NEG	NEG	NEG	NEG	NEG	NEG	NEG
UKD-15571283	SCCmec IVa (MW2)	CC88	NEG	NEG	NEG	NEG	NEG	NEG	NEG	NEG	NEG	NEG	NEG
KHDN-10406719	SCCmec IVa (MW2)	ST93	NEG	NEG	NEG	NEG	NEG	NEG	NEG	NEG	NEG	NEG	neg
KHDN-0060294	SCCmec IVa (MW2)	ST93	NEG	NEG	NEG	NEG	NEG	NEG	NEG	NEG	NEG	NEG	neg
UKD-0734190	SCCmec IVa (MW2)	CC97	NEG	NEG	NEG	NEG	NEG	NEG	NEG	NEG	NEG	NEG	NEG
UKD-13006983	SCCmec IVa (MW2)	CC97	NEG	NEG	NEG	NEG	NEG	NEG	NEG	NEG	NEG	NEG	NEG
Prediction for H131520133	SCCmec IVa (H131520133)	CC152	NEG	NEG	NEG	NEG	NEG	NEG	NEG	NEG	NEG	NEG	NEG
KHDN-325892	SCCmec IVa (H131520133)	CC30	NEG	NEG	NEG	NEG	NEG	NEG	NEG	NEG	NEG	NEG	NEG
UKD-15580207	SCCmec IVa (H131520133)	CC88	NEG	NEG	NEG	NEG	NEG	NEG	NEG	NEG	NEG	NEG	NEG
UKD-0521159	SCCmec Iva	CC8	NEG	NEG	NEG	NEG	NEG	NEG	NEG	NEG	NEG	NEG	neg
Prediction for CMFT503 : HF569113.1	SCCmec IVa (CMFT503)		NEG	NEG	NEG	NEG	NEG	NEG	NEG	NEG	NEG	NEG	NEG
Prediction for 21343, GenBank AHKV	SCCmec IVa (CMFT503)	CC88	NEG	NEG	NEG	NEG	NEG	NEG	NEG	NEG	NEG	NEG	NEG
UKD-1420740	SCCmec IVa (CMFT503)	CC22	NEG	NEG	NEG	NEG	NEG	NEG	NEG	NEG	NEG	NEG	NEG
UKD-15575124	SCCmec IVa (CMFT503)	CC22	NEG	NEG	NEG	NEG	NEG	NEG	NEG	NEG	NEG	NEG	NEG
UKD-16584947	SCCmec IVa (CMFT503)	CC22	NEG	NEG	NEG	NEG	NEG	NEG	NEG	NEG	NEG	NEG	NEG
UKD-16575077	SCCmec IVa (CMFT503)	CC88	NEG	NEG	NEG	NEG	NEG	NEG	NEG	NEG	NEG	NEG	NEG
Prediction for TCH1516, GenBank CP000730.1	SCCmec IVa+ACME1+Cu (USA300)	CC8	NEG	NEG	POS	NEG	NEG	NEG	NEG	NEG	NEG	NEG	NEG
Reference strain USA300-TCH1516	SCCmec IVa+ACME1+Cu (USA300)	CC8	NEG	NEG	POS	NEG	NEG	NEG	NEG	NEG	NEG	NEG	NEG
Prediction for FPR3757, GenBank CP000255.1	SCCmec IVa+ACME1+Cu (USA300)	CC8	NEG	NEG	POS	NEG	NEG	NEG	NEG	NEG	NEG	NEG	NEG
UKD-0516386	SCCmec IVa+ACME1+Cu (USA300)	CC8	NEG	NEG	POS	NEG	NEG	NEG	NEG	NEG	NEG	NEG	neg
UKD-0815773	SCCmec IVa+ACME1+Cu (USA300)	CC8	NEG	NEG	POS	NEG	NEG	NEG	NEG	NEG	NEG	NEG	neg
UKD-1414654	SCCmec IVa+ACME1+Cu (USA300)	CC8	NEG	NEG	POS	NEG	NEG	NEG	NEG	NEG	NEG	NEG	NEG
UKD-0408036	SCCmec IVa+ACME2	CC45 [agr I]	NEG	NEG	NEG	NEG	NEG	NEG	NEG	NEG	NEG	NEG	NEG
UKD-0044035	SCCmec IVa+ccrC (Hannover)	CC8	NEG	NEG	NEG	NEG	NEG	NEG	NEG	NEG	NEG	NEG	NEG
UKD-0136287	SCCmec IVa+ccrC (Hannover)	CC8	NEG	NEG	NEG	NEG	NEG	NEG	NEG	NEG	NEG	NEG	NEG

Isolate ID	SCCmec type	Clonal complex	HEAVY METAL RESISTANCES (SCC-associated)										
			mercury resistance operon		copper exporting ATPase	Multi copper oxidase	Arsenical pump membrane protein arsB (SCC)			Carbamate kinase	cadmium and zinc resistance gene C (formerly known as copA)	Cadmium transport protein D	Putative regulator of cadmium efflux
			merA	merB	copA2_SCC	mco-SCC	hp1478_ arsB	hp1536_ arsB	hp1526_ arsB	arsC (SCC or plasmidic)	czrC	cadD (R35)	cadX (JCSC6943)
Prediction for JCSC1978, GenBank AB063173.1	SCCmec IVb/d/i (JCSC1978/6668/4469)		NEG	NEG	NEG	NEG	NEG	NEG	NEG	NEG	NEG	NEG	NEG
Reference strain JCSC1978 (8/6-3P)	SCCmec IVb/d/i (JCSC1978/6668/4469)	CC8	NEG	NEG	NEG	NEG	NEG	NEG	NEG	NEG	NEG	NEG	neg
Prediction for JCSC6668, GenBank AB425823.1	SCCmec IVb/d/i (JCSC1978/6668/4469)		NEG	NEG	NEG	NEG	NEG	NEG	NEG	NEG	NEG	NEG	NEG
Reference strain JCSC4469	SCCmec IVb/d/i (JCSC1978/6668/4469)	CC5	NEG	NEG	NEG	NEG	NEG	NEG	NEG	NEG	NEG	NEG	NEG
UKD-1103429	SCCmec IVb/d/i (JCSC1978/6668/4469)	CC7	NEG	NEG	NEG	NEG	NEG	NEG	NEG	NEG	NEG	NEG	NEG
UKD-1306141	SCCmec IVb/d/i (JCSC1978/6668/4469)	CC7	NEG	NEG	NEG	NEG	NEG	NEG	NEG	NEG	NEG	NEG	NEG
UKD-0500089	SCCmec IVb/d/i (JCSC1978/6668/4469)	CC5	NEG	NEG	NEG	NEG	NEG	NEG	NEG	NEG	NEG	NEG	NEG
UKD-0501416	SCCmec IVb/d/i (JCSC1978/6668/4469)	CC5	NEG	NEG	NEG	NEG	NEG	NEG	NEG	NEG	NEG	NEG	NEG
UKD-0934574	SCCmec IVb/d/i (JCSC1978/6668/4469)	CC5	NEG	NEG	NEG	NEG	NEG	NEG	NEG	NEG	NEG	NEG	NEG
UKD-0746779	SCCmec IVb/d/i (JCSC1978/6668/4469)	CC45 [agr I]	NEG	NEG	NEG	NEG	NEG	NEG	NEG	NEG	NEG	NEG	NEG
UKD-0812518	SCCmec IVb/d/i (JCSC1978/6668/4469)	CC45 [agr I]	NEG	NEG	NEG	NEG	NEG	NEG	NEG	NEG	NEG	NEG	NEG
Prediction for 21209, GenBank AGRP	SCCmec IVb/d/i (21209)	CC8	NEG	NEG	NEG	NEG	NEG	NEG	NEG	NEG	NEG	NEG	NEG
UKD-0233579	SCCmec IVb/d/i (21209)	CC8	NEG	NEG	NEG	NEG	NEG	NEG	NEG	NEG	NEG	NEG	NEG
UKD-0504920	SCCmec IVb/d/i (21209)	CC8	NEG	NEG	NEG	NEG	NEG	NEG	NEG	NEG	NEG	NEG	neg
UKD-0725999	SCCmecIV b/d/i+Cu	CC30	NEG	NEG	POS	NEG	NEG	NEG	NEG	NEG	NEG	NEG	NEG
Prediction for IS-105, GenBank AHLR	SCCmec IVc (IS-105)	CC22	NEG	NEG	NEG	NEG	NEG	NEG	NEG	NEG	NEG	NEG	NEG
UKD-0402421	SCCmec IVc (IS-105), merA/B+	CC5	POS	POS	NEG	NEG	NEG	NEG	NEG	NEG	NEG	NEG	NEG
UKD-0731319	SCCmec IVc (IS-105), merA/B+	CC5	POS	POS	NEG	NEG	NEG	NEG	NEG	NEG	NEG	NEG	NEG
UKD-0733137	SCCmec IVc (IS-105)	CC8	NEG	NEG	NEG	NEG	NEG	NEG	NEG	NEG	NEG	NEG	NEG
UKD-0305690	SCCmec IVc (IS-105)	CC22	NEG	NEG	NEG	NEG	NEG	NEG	NEG	NEG	NEG	NEG	NEG
UKD-1424706	SCCmec IVc (IS-105)	CC22	NEG	NEG	NEG	NEG	NEG	NEG	NEG	NEG	NEG	NEG	NEG
Prediction for TCH60, GenBank CP002110.1	SCCmec IVc (TCH60)	CC30	NEG	NEG	NEG	NEG	NEG	NEG	NEG	NEG	NEG	NEG	NEG
Reference strain JCSC4788	SCCmec IVc (TCH60)	CC8	NEG	NEG	NEG	NEG	NEG	NEG	NEG	NEG	NEG	NEG	NEG
UKD-1318318	SCCmec IVc (TCH60)	CC5	NEG	NEG	NEG	NEG	NEG	NEG	NEG	NEG	NEG	NEG	NEG
UKD-14580050	SCCmec IVc (TCH60)	CC5	NEG	NEG	NEG	NEG	NEG	NEG	NEG	NEG	NEG	NEG	NEG
UKD-08F01314	SCCmec IVc (TCH60)	CC8	NEG	NEG	NEG	NEG	NEG	NEG	NEG	NEG	NEG	NEG	NEG
KHDN-50361518	SCCmec IVc (TCH60)	CC8	NEG	NEG	NEG	NEG	NEG	NEG	NEG	NEG	NEG	NEG	neg
UKD-0307912	SCCmec IVc (TCH60)	CC22	NEG	NEG	NEG	NEG	NEG	NEG	NEG	NEG	NEG	NEG	NEG
UKD-0220390	SCCmec IVc (TCH60)	CC45 [agr I]	NEG	NEG	NEG	NEG	NEG	NEG	NEG	NEG	NEG	NEG	NEG
UKD-0311106	SCCmec IVc (TCH60)	CC45 [agr I]	NEG	NEG	NEG	NEG	NEG	NEG	NEG	NEG	NEG	NEG	NEG
UKD-0719855	SCCmec IVc (TCH60)	CC45 [agr I]	NEG	NEG	NEG	NEG	NEG	NEG	NEG	NEG	NEG	NEG	NEG
UKD-0422079	SCCmec IVc (TCH60)	CC45 [agr I]	NEG	NEG	NEG	NEG	NEG	NEG	NEG	NEG	NEG	NEG	NEG
UKD-0409235	SCCmec IVc (TCH60)	CC80	NEG	NEG	NEG	NEG	NEG	NEG	NEG	NEG	NEG	NEG	NEG
UKD-0409807	SCCmec IVc (TCH60)	CC80	NEG	NEG	NEG	NEG	NEG	NEG	NEG	NEG	NEG	NEG	NEG
UKD-0416073	SCCmec IVc (TCH60)	CC80	NEG	NEG	NEG	NEG	NEG	NEG	NEG	NEG	NEG	NEG	NEG
UKD-0431728	SCCmec IVc (TCH60)	CC80	NEG	NEG	NEG	NEG	NEG	NEG	NEG	NEG	NEG	NEG	NEG
UKD-1001508	SCCmec IVc (TCH60)	CC80	NEG	NEG	NEG	NEG	NEG	NEG	NEG	NEG	NEG	NEG	NEG
UKD-1010412	SCCmec IVc (TCH60)	CC80	NEG	NEG	NEG	NEG	NEG	NEG	NEG	NEG	NEG	NEG	neg
UKD-12753005	SCCmec IVc (TCH60)	CC80	NEG	NEG	NEG	NEG	NEG	NEG	NEG	NEG	NEG	NEG	NEG
UKD-15574809	SCCmec IVc (TCH60)	CC80	NEG	NEG	NEG	NEG	NEG	NEG	NEG	NEG	NEG	NEG	NEG
KHDN-16120291	SCCmec IVc (TCH60)	CC88	NEG	NEG	NEG	NEG	NEG	NEG	NEG	NEG	NEG	NEG	NEG
Prediction for WW2703/97, GenBank ACSW	SCCmec IVc (WW2703/97)	CC30	NEG	NEG	NEG	NEG	NEG	NEG	NEG	NEG	NEG	NEG	NEG
UKD-14580088	SCCmec IVc (WW2703/97)	CC398	NEG	NEG	NEG	NEG	NEG	NEG	NEG	NEG	NEG	NEG	NEG
Prediction for SA_ST125_MupR, GenBank ASTH	SCCmec IVc+speG+ccrAB4 (SA_ST125)	CC5	NEG	NEG	NEG	NEG	NEG	NEG	NEG	NEG	NEG	NEG	NEG
UKD-0439909	SCCmec IVc+speG+ccrAB4 (SA_ST125)	CC5	NEG	NEG	NEG	NEG	NEG	NEG	NEG	NEG	NEG	NEG	NEG
Prediction for MRSA177, GenBank AECP	SCCmec IVc+Cu/Hg (MRSA177)		POS	POS	POS	POS	NEG	NEG	NEG	NEG	NEG	NEG	NEG
UKD-1279734	SCCmec IVc+Cu/Hg (MRSA177)	CC8	POS	POS	POS	POS	NEG	NEG	NEG	NEG	NEG	NEG	NEG
UKD-14580899	SCCmec IVc+Cu/Hg (MRSA177)	CC8	POS	POS	POS	POS	NEG	NEG	NEG	NEG	NEG	NEG	NEG
UKD-16002228	SCCmec IVc+Cu/Hg (MRSA177)	CC8	POS	POS	POS	POS	NEG	NEG	NEG	NEG	NEG	NEG	NEG
Prediction for HO 5096 0412, GenBank HE681097.1	SCCmec IVh/j (HO50960412/JCSC6670)	CC22	NEG	NEG	NEG	NEG	NEG	NEG	NEG	NEG	NEG	NEG	NEG
Prediction for JCSC6670, GenBank AB425824.1	SCCmec IVh/j (HO50960412/JCSC6670)		NEG	NEG	NEG	NEG	NEG	NEG	NEG	NEG	NEG	NEG	NEG
Reference strain Dublin_DSH_E1749	SCCmec IVh/j (HO50960412/JCSC6670)		NEG	NEG	NEG	NEG	NEG	NEG	NEG	NEG	NEG	NEG	NEG
UKD-14576248	SCCmec IVh/j (HO50960412/JCSC6670)	CC8	NEG	NEG	NEG	NEG	NEG	NEG	NEG	NEG	NEG	NEG	NEG
UKD-0120122	SCCmec IVh/j (HO50960412/JCSC6670)	CC22	NEG	NEG	NEG	NEG	NEG	NEG	NEG	NEG	NEG	NEG	NEG
UKD-0205319	SCCmec IVh/j (HO50960412/JCSC6670)	CC22	NEG	NEG	NEG	NEG	NEG	NEG	NEG	NEG	NEG	NEG	NEG
UKD-0221259	SCCmec IVh/j (HO50960412/JCSC6670)	CC22	NEG	NEG	NEG	NEG	NEG	NEG	NEG	NEG	NEG	NEG	NEG
UKD-0418654	SCCmec IVh/j (HO50960412/JCSC6670)	CC22	NEG	NEG	NEG	NEG	NEG	AMB	NEG	NEG	NEG	NEG	NEG
UKD-0429721	SCCmec IVh/j (HO50960412/JCSC6670)	CC22	NEG	NEG	NEG	NEG	NEG	NEG	AMB	NEG	NEG	NEG	NEG
UKD-1116194	SCCmec IVh/j (HO50960412/JCSC6670)	CC22	NEG	NEG	NEG	NEG	NEG	NEG	NEG	NEG	NEG	NEG	NEG
UKD-1122367	SCCmec IVh/j (HO50960412/JCSC6670)	CC22	NEG	NEG	NEG	NEG	NEG	NEG	NEG	NEG	NEG	NEG	NEG
UKD-14012840	SCCmec IVh/j (HO50960412/JCSC6670)	CC22	NEG	NEG	NEG	NEG	NEG	NEG	NEG	NEG	NEG	NEG	NEG
UKD-14579833	SCCmec IVh/j (HO50960412/JCSC6670)	CC22	NEG	NEG	NEG	NEG	NEG	NEG	NEG	NEG	NEG	NEG	NEG
UKD-15579198	SCCmec IVh/j (HO50960412/JCSC6670)	CC22	NEG	NEG	NEG	NEG	NEG	NEG	NEG	NEG	NEG	NEG	NEG
UKD-4052193	SCCmec IVh/j (HO50960412/JCSC6670)	CC22	NEG	NEG	NEG	NEG	NEG	NEG	NEG	NEG	NEG	NEG	NEG
UKD-124968	SCCmec IVh/j (HO50960412/JCSC6670)	CC22	NEG	NEG	NEG	NEG	NEG	NEG	NEG	NEG	NEG	NEG	NEG
UKD-14572546	SCCmec IVh/j (HO50960412/JCSC6670)	CC22	NEG	NEG	NEG	NEG	NEG	NEG	NEG	NEG	NEG	NEG	NEG
UKD-14582287	SCCmec IVh/j (HO50960412/JCSC6670)	CC22	NEG	NEG	NEG	NEG	NEG	NEG	NEG	NEG	NEG	NEG	NEG
UKD-14583376	SCCmec IVh/j (HO50960412/JCSC6670)	CC22	NEG	NEG	NEG	NEG	NEG	NEG	NEG	NEG	NEG	NEG	NEG
UKD-14583496	SCCmec IVh/j (HO50960412/JCSC6670)	CC22	NEG	NEG	NEG	NEG	NEG	NEG	NEG	NEG	NEG	NEG	NEG
UKD-0013324	SCCmec IVh/j (HO50960412/JCSC6670)	CC22	NEG	NEG	NEG	NEG	NEG	NEG	NEG	NEG	NEG	NEG	NEG
UKD-0125329	SCCmec IVh/j (HO50960412/JCSC6670)	CC22	NEG	NEG	NEG	NEG	NEG	NEG	NEG	NEG	NEG	NEG	NEG
UKD-0215498	SCCmec IVh/j (HO50960412/JCSC6670)	CC22	NEG	NEG	NEG	NEG	NEG	NEG	NEG	NEG	NEG	NEG	NEG
Prediction for M08-0126, GenBank FR753166.1	SCCmec IVh/j+ACME2 (M08-0126)	CC22	NEG	NEG	NEG	NEG	NEG	NEG	NEG	NEG	NEG	NEG	NEG
UKD-13794831	SCCmec IVh/j+ACME2 (M08-0126)	CC22	NEG	NEG	NEG	NEG	NEG	NEG	NEG	NEG	NEG	NEG	neg
UKD-5150204	SCCmec IV+speG+Cu/czrC+ccrAB4	CC22	NEG	NEG	POS	NEG	NEG	NEG	NEG	NEG	POS	NEG	neg
UKD-15578423	SCCmec IV+speG+czrC+ccrAB4 var. 1	CC22	NEG	NEG	NEG	NEG	NEG	NEG	NEG	NEG	POS	NEG	NEG
UKD-16582870	SCCmec IV+speG+czrC+ccrAB4 var. 1	CC22	NEG	NEG	NEG	NEG	NEG	NEG	NEG	NEG	POS	NEG	NEG
UKD-15571940	SCCmec IV+speG+czrC+ccrAB4 var. 2	CC22	NEG	NEG	NEG	NEG	NEG	NEG	NEG	NEG	POS	NEG	NEG

Isolate ID	SCCmec type	Clonal complex	HEAVY METAL RESISTANCES (SCC-associated)										
			mercury resistance operon		copper exporting ATPase	Multi copper oxidase	Arsenical pump membrane protein arsB (SCC)			Carbamate kinase	cadmium and zinc resistance gene C (formerly known as copA)	Cadmium transport protein D	Putative regulator of cadmium efflux
			merA	merB	copA2_SCC	mco-SCC	hp1478_ arsB	hp1536_ arsB	hp1526_ arsB	arsC (SCC or plasmidic)	czrC	cadD (R35)	cadX (JCSC6943)
Prediction for CN1, GenBank CP003979.1	SCCmec IVA (CN1)	CC8 (ST72)	NEG	NEG	NEG	NEG	NEG	NEG	NEG	NEG	NEG	NEG	NEG
UKD-0900871	SCCmec IVA (CN1)	CC8 (ST72)	NEG	NEG	NEG	NEG	NEG	NEG	NEG	NEG	NEG	NEG	NEG
Prediction for CMFT1723, GenBank HF569096.1	SCCmec V (Bengal Bay)		NEG	NEG	NEG	NEG	NEG	NEG	NEG	NEG	NEG	NEG	NEG
KHDN-10405134	SCCmec V (Bengal Bay)	CC1	NEG	NEG	NEG	NEG	NEG	NEG	NEG	NEG	NEG	NEG	NEG
UKD-0932405	SCCmec V (Bengal Bay)	CC1 (ST573/772)	NEG	NEG	NEG	NEG	NEG	NEG	NEG	NEG	NEG	NEG	NEG
UKD-1003510	SCCmec V (Bengal Bay)	CC1 (ST573/772)	NEG	NEG	NEG	NEG	NEG	NEG	NEG	NEG	NEG	NEG	NEG
UKD-1112420	SCCmec V (Bengal Bay)	CC1 (ST573/772)	NEG	NEG	NEG	NEG	NEG	NEG	NEG	NEG	NEG	NEG	NEG
UKD-1275832	SCCmec V (Bengal Bay)	CC1 (ST573/772)	NEG	NEG	NEG	NEG	NEG	NEG	NEG	NEG	NEG	NEG	neg
UKD-13023811	SCCmec V (Bengal Bay)	CC1 (ST573/772)	NEG	NEG	NEG	NEG	NEG	NEG	NEG	NEG	NEG	NEG	NEG
UKD-14572163	SCCmec V	CC5	NEG	NEG	NEG	NEG	NEG	NEG	NEG	NEG	NEG	NEG	NEG
KHDN-10421133	SCCmec V+fus	CC97	NEG	NEG	NEG	NEG	NEG	NEG	NEG	NEG	NEG	NEG	NEG
KHDN-21343541	SCCmec V+fus+tir+ccrAB1	CC1	NEG	NEG	NEG	NEG	NEG	NEG	NEG	NEG	NEG	NEG	neg
KHDN-10416893	SCCmec V+czrC+speG+ccrAB4	CC22	NEG	NEG	NEG	NEG	NEG	NEG	NEG	NEG	POS	NEG	NEG
Prediction for GR1, GenBank AJLX	SCCmec VT (GR1)	CC361	NEG	NEG	NEG	NEG	NEG	NEG	NEG	NEG	NEG	NEG	NEG
UKD-0422872	SCCmec VT (GR1)	CC152	NEG	NEG	NEG	NEG	NEG	NEG	NEG	NEG	NEG	NEG	NEG
Prediction for PM1, GenBank BAFA	SCCmec VT (PM1)	CC59	NEG	NEG	NEG	NEG	NEG	NEG	NEG	NEG	NEG	NEG	NEG
KHDN-000871	SCCmec VT (PM1)	CC45 (agr I)	NEG	NEG	NEG	NEG	NEG	NEG	NEG	NEG	NEG	NEG	NEG
UKD-0725052	SCCmec VT (PM1)	CC59	NEG	NEG	NEG	NEG	NEG	NEG	NEG	NEG	NEG	NEG	neg
UKD-1312138	SCCmec VT (PM1)	CC59	NEG	NEG	NEG	NEG	NEG	NEG	NEG	NEG	NEG	NEG	NEG
UKD-Z4728	SCCmec VT (PM1)	CC59	NEG	NEG	NEG	NEG	NEG	NEG	NEG	NEG	NEG	NEG	NEG
UKD-K0199	SCCmec VT unknown var.1	CC7	NEG	NEG	NEG	NEG	NEG	NEG	NEG	NEG	NEG	NEG	NEG
UKD-15584619	SCCmec VT unknown var.2	CC30	NEG	NEG	NEG	NEG	NEG	NEG	NEG	NEG	NEG	NEG	NEG
UKD-0712568	SCCmec VT unknown var.3	CC45 (agr I)	NEG	NEG	NEG	NEG	NEG	NEG	NEG	NEG	NEG	NEG	NEG
KHDN-10420296	SCCmec VT+ACME2	CC45 (agr I)	NEG	NEG	NEG	NEG	NEG	NEG	NEG	NEG	NEG	NEG	NEG
UKD-15577956	SCCmec VT+Cu/czrC+ccrA1	CC398	NEG	NEG	POS	NEG	NEG	NEG	NEG	NEG	POS	NEG	NEG
Prediction for S0385, GenBank AM990992	SCCmec VT+czrC (SO385)	CC398	NEG	NEG	NEG	NEG	NEG	NEG	NEG	NEG	POS	NEG	NEG
UKD-0628560	SCCmec VT+czrC (SO385)	CC8	NEG	NEG	NEG	NEG	NEG	NEG	NEG	NEG	POS	NEG	NEG
UKD-0510018	SCCmec VT+czrC (SO385)	CC398	NEG	NEG	NEG	NEG	NEG	NEG	NEG	NEG	POS	NEG	NEG
UKD-0903558	SCCmec VT+czrC (SO385)	CC398	NEG	NEG	NEG	NEG	NEG	NEG	NEG	NEG	POS	NEG	NEG
UKD-0929815	SCCmec VT+czrC (SO385)	CC398	NEG	NEG	NEG	NEG	NEG	NEG	NEG	NEG	POS	NEG	NEG
UKD-0907762	SCCmec VT+czrC (SO385)	CC398	NEG	NEG	NEG	NEG	NEG	NEG	NEG	NEG	POS	NEG	NEG
UKD-1025105	SCCmec VT+czrC (SO385)	CC398	NEG	NEG	NEG	NEG	NEG	NEG	NEG	NEG	POS	NEG	NEG
UKD-11780898	SCCmec VT+czrC (SO385)	CC398	NEG	NEG	NEG	NEG	NEG	NEG	NEG	NEG	POS	NEG	NEG
UKD-1377009	SCCmec VT+czrC (SO385)	CC398	NEG	NEG	NEG	NEG	NEG	NEG	NEG	NEG	POS	NEG	NEG
UKD-143406	SCCmec VT+czrC (SO385)	CC398	NEG	NEG	NEG	NEG	NEG	NEG	NEG	NEG	POS	NEG	NEG
UKD-1400363	SCCmec VT+czrC (SO385)	CC398	NEG	NEG	NEG	NEG	NEG	NEG	NEG	NEG	POS	NEG	NEG
UKD-14571852	SCCmec VT+czrC (SO385)	CC398	NEG	NEG	NEG	NEG	NEG	NEG	NEG	NEG	POS	NEG	NEG
UKD-14582574	SCCmec VT+czrC (SO385)	CC398	NEG	NEG	NEG	NEG	NEG	NEG	NEG	NEG	POS	NEG	NEG
UKD-152108	SCCmec VT+czrC (SO385)	CC398	NEG	NEG	NEG	NEG	NEG	NEG	NEG	NEG	POS	NEG	NEG
UKD-15573321	SCCmec VT+czrC (SO385)	CC398	NEG	NEG	NEG	NEG	NEG	NEG	NEG	NEG	POS	NEG	NEG
UKD-15584034	SCCmec VT+czrC , ydhK-neg. variant	CC398	NEG	NEG	NEG	NEG	NEG	NEG	NEG	NEG	POS	NEG	NEG
UKD-0932581	SCCmecVT+fus+czrC	CC22	NEG	NEG	NEG	NEG	NEG	NEG	NEG	NEG	POS	NEG	NEG
UKD-1121994	SCCmecVT+fus+czrC	CC22	NEG	NEG	NEG	NEG	NEG	NEG	NEG	NEG	POS	NEG	NEG
UKD-1272252	SCCmecVT+fus+czrC	CC22	NEG	NEG	NEG	NEG	NEG	NEG	NEG	NEG	POS	NEG	neg
Prediction for MRSA18	SCCmec VI+fus (MRSA18)		NEG	NEG	NEG	NEG	NEG	NEG	NEG	NEG	NEG	NEG	NEG
UKD-1103380	SCCmec VI+fus (MRSA18)	CC7	NEG	NEG	NEG	NEG	NEG	NEG	NEG	NEG	NEG	NEG	NEG
Prediction for M10/0061, GenBank FR823292.1	SCCmec XI (LGA251/M10-61)	CC130	NEG	NEG	NEG	AMB	NEG	NEG	POS	NEG	NEG	NEG	NEG
Reference strain Dublin-DSH_M10 0061	SCCmec XI (LGA251/M10-61)	CC130	NEG	NEG	NEG	AMB	NEG	NEG	POS	NEG	NEG	NEG	NEG
Prediction for LGA251, GenBank FR821779.1	SCCmec XI (LGA251/M10-61)	ST425	NEG	NEG	NEG	AMB	NEG	NEG	POS	NEG	NEG	NEG	NEG
UKD-14015835	SCCmec XI (LGA251/M10-61)	CC130	NEG	NEG	NEG	AMB	NEG	NEG	POS	NEG	NEG	NEG	NEG
UKD-16573252	SCCmec XI (LGA251/M10-61)	CC130	NEG	NEG	NEG	AMB	NEG	NEG	POS	NEG	NEG	NEG	NEG
UKD-0217002	PseudoSCCmec, class B mec complex+Hg	CC8	POS	POS	NEG	NEG	NEG	NEG	NEG	NEG	NEG	NEG	NEG
UKD-0008510	PseudoSCCmec, class B mec complex+Hg	CC8	POS	POS	NEG	NEG	NEG	NEG	NEG	NEG	NEG	NEG	NEG
UKD-0110962	PseudoSCCmec, class B mec complex+Hg	CC8	POS	POS	NEG	NEG	NEG	NEG	NEG	NEG	NEG	NEG	NEG
KHDN-10408593	PseudoSCCmec, class B mec complex+Cu/Hg	CC8	POS	POS	POS	POS	NEG	NEG	NEG	NEG	NEG	NEG	neg
UKD-0007201	PseudoSCCmec, class B mec complex	CC5	POS	POS	NEG	NEG	NEG	NEG	NEG	NEG	NEG	NEG	NEG
UKD-0033204	PseudoSCCmec, class B mec complex	CC5	POS	POS	NEG	NEG	NEG	NEG	NEG	NEG	NEG	NEG	NEG
UKD-0134329	PseudoSCCmec, class B mec complex	CC5	POS	POS	NEG	NEG	NEG	NEG	NEG	NEG	NEG	NEG	NEG
UKD-0806946	PseudoSCCmec class C+As/Cu	CC398	NEG	NEG	POS	NEG	POS	NEG	NEG	POS	NEG	NEG	NEG
UKD-0500610	ACME2	CC8	NEG	NEG	NEG	NEG	NEG	NEG	NEG	NEG	NEG	NEG	NEG
UKD-0610438	ACME2	CC8	NEG	NEG	NEG	NEG	NEG	NEG	NEG	NEG	NEG	NEG	NEG
KHDN-10409344	ACME2	CC8	NEG	NEG	NEG	NEG	NEG	NEG	NEG	NEG	NEG	NEG	NEG
UKD-0322559	ACME2+speG+czrC+ccrAB4	CC8	NEG	NEG	NEG	NEG	NEG	NEG	NEG	NEG	POS	NEG	NEG
UKD-0501998	ACME2+speG+czrC+ccrAB4	CC8	NEG	NEG	NEG	NEG	NEG	NEG	NEG	NEG	POS	NEG	NEG
UKD-0505213	ACME2+speG+czrC+ccrAB4	CC8	NEG	NEG	NEG	NEG	NEG	NEG	NEG	NEG	POS	NEG	NEG
Prediction for MSSA476, GenBank BX571857.1	fus+tir+ccrAB1 (MSSA476)	CC1	NEG	NEG	NEG	NEG	NEG	NEG	NEG	NEG	NEG	NEG	NEG
Reference strain Sanger 476	fus+tir+ccrAB1 (MSSA476)	CC1	NEG	NEG	NEG	NEG	NEG	NEG	NEG	NEG	NEG	NEG	NEG
UKD-14023914	fus+tir+ccrAB1 (MSSA476)	CC1	NEG	NEG	NEG	NEG	NEG	NEG	NEG	NEG	NEG	NEG	NEG
UKD-K00034	kdp+ccrAB2	CC182	NEG	NEG	NEG	NEG	NEG	NEG	NEG	NEG	NEG	NEG	NEG
UKD-15580369	As+ccrAB4	CC22	NEG	NEG	NEG	NEG	NEG	NEG	POS	NEG	NEG	NEG	NEG
UKD-15570100	ACME3+ccrAB1 (Strain 21342)	CC80	NEG	NEG	NEG	NEG	NEG	NEG	NEG	NEG	NEG	NEG	NEG
UKD-1010678	speG+czrC+ccrAB4	CC8	NEG	NEG	NEG	NEG	NEG	NEG	NEG	NEG	POS	NEG	NEG
UKD-1008243	speG+czrC+ccrAB4	CC8	NEG	NEG	NEG	NEG	NEG	NEG	NEG	NEG	POS	NEG	NEG
UKD-1009430	speG+czrC+ccrAB4	CC8	NEG	NEG	NEG	NEG	NEG	NEG	NEG	NEG	POS	NEG	NEG
UKD-12071828	speG+czrC+ccrAB4	CC22	NEG	NEG	NEG	NEG	NEG	NEG	NEG	NEG	POS	NEG	NEG
UKD-14011883	speG+czrC+ccrAB4	CC22	NEG	NEG	NEG	NEG	NEG	NEG	NEG	NEG	POS	NEG	NEG
UKD-15008293	speG+czrC+ccrAB4	CC22	NEG	NEG	NEG	NEG	NEG	NEG	NEG	NEG	POS	NEG	NEG
UKD-1514877	speG+czrC+ccrAB4	CC22	NEG	NEG	NEG	NEG	NEG	NEG	NEG	NEG	POS	NEG	NEG
UKD-15580971	speG+czrC+ccrAB4	CC22	NEG	NEG	NEG	NEG	NEG	NEG	NEG	NEG	POS	NEG	NEG
UKD-15582646	speG+czrC+ccrAB4	CC22	NEG	NEG	NEG	NEG	NEG	NEG	NEG	NEG	POS	NEG	NEG

Isolate ID	SCCmec type	Clonal complex	RESISTANCE : PENICILLINASE			RESISTANCE : MLS-ANTIBIOTICS												RESISTANCE : AMINOGLYSOSIDES			RESISTANCE : MISCELLANEOUS GENES										
			beta-lactamase	beta lactamase repressor (inhibitor)	beta-lactamase regulatory protein	erythromycin/clindamycin resistance			Linco-samid-Nucleo-tidyltrans-ferase	energy-dependent efflux of erythro-mycin	macrolide efflux protein A	probable lysylphos-phatidyl-glycerol synthetase	virginia-mycin A acetyltransf erase	acetyl-transferase inactivating streptogra min A	ATP binding protein, streptogramin-A-resistance		virginia-mycin B hydrolase	bifunctional enzyme Aac/Aph, gentamicin resistance	amino-glycoside adenyl-transferase, tobramycin resistance	3'5'-aminog-lycoside phospho-transferase, neo-/kanamycin resistance	strepto-thricine-acetyl-transferase	dihydro-folate reductase type 1	fusidic acid resistence	mupirocin resistence protein	tetracycline-resistance		chloramphenicol acetyltransferase				
			blaZ	blaI	blaR	ermA	ermB	ermC	linA/ InuA	msrA	mefA	mph(C)	vat(A)	vat(B)	vga(A)	vga(A) (BM 3327)	vgb	aacA-aphD	aadD	aphA3	sat	dfrA	far1	mupA	tetK	tetM	cat	cat (pC221)	cat (pc223)	cat (pMC524)	cat (pSBK203R)
VIRTUAL HYBRIDISATION for COL, GenBank CP000046	SCCmec I (COL)	CC8	NEG	NEG	NEG	NEG	NEG	NEG	NEG	NEG	NEG	NEG	NEG	NEG	NEG	NEG	NEG	NEG	NEG	NEG	NEG	NEG	NEG	NEG	POS	NEG	NEG	NEG	NEG	NEG	NEG
Reference strain COL	SCCmec I (COL)	CC8	NEG	NEG	NEG	NEG		NEG	NEG	NEG	NEG	NEG	NEG	NEG	NEG	NEG	NEG	NEG	NEG	NEG	NEG	NEG	NEG	NEG	POS	NEG	NEG	NEG	NEG	NEG	NEG
UKD-0007063	SCCmec I (COL)	CC5	POS	POS	POS	POS	NEG	NEG	NEG	NEG	NEG	NEG	NEG	NEG	AMB	NEG	NEG	POS	NEG	POS	POS	NEG	NEG	NEG	NEG	NEG	NEG	NEG	NEG	NEG	NEG
UKD-0220759	SCCmec I (COL)	CC5	POS	POS	POS	POS	NEG	NEG	NEG	NEG	NEG	NEG	NEG	NEG	NEG	NEG	NEG	POS	NEG	POS	POS	NEG	NEG	NEG	NEG	NEG	NEG	NEG	NEG	NEG	NEG
UKD-0308986	SCCmec I (COL)	CC5	NEG	NEG	NEG	POS	NEG	NEG	NEG	NEG	NEG	NEG	NEG	NEG	NEG	NEG	NEG	POS	NEG	POS	POS	NEG	NEG	NEG	NEG	NEG	NEG	NEG	NEG	NEG	NEG
UKD-0500009	SCCmec I (COL)	CC5	POS	POS	POS	POS	NEG	NEG	NEG	NEG	NEG	NEG	NEG	NEG	NEG	NEG	NEG	POS	NEG	POS	POS	NEG	NEG	NEG	NEG	NEG	NEG	NEG	NEG	NEG	NEG
UKD-0508483	SCCmec I (COL)	CC5	POS	POS	POS	POS		NEG	NEG	NEG	NEG	NEG	NEG	NEG	NEG	NEG	NEG	POS	NEG	POS	POS	NEG	NEG	NEG	NEG	NEG	NEG	NEG	NEG	NEG	NEG
UKD-0519001	SCCmec I (COL)	CC5	POS	POS	POS	POS		NEG	NEG	NEG	NEG	NEG	NEG	NEG	NEG	NEG	NEG	POS	NEG	POS	POS	NEG	NEG	NEG	NEG	NEG	NEG	NEG	NEG	NEG	NEG
UKD-0701876	SCCmec I (COL)	CC5	POS	POS	POS	POS	NEG	NEG	NEG	NEG	NEG	NEG	NEG	NEG	NEG	NEG	NEG	POS	NEG	POS	POS	NEG	NEG	NEG	NEG	NEG	NEG	NEG	NEG	NEG	NEG
UKD-0708865	SCCmec I (COL)	CC5	POS	AMB	POS	POS		NEG	NEG	NEG		NEG	NEG	NEG	NEG	NEG	NEG	POS	NEG	POS	POS	NEG	NEG	NEG	NEG	NEG	NEG	NEG	NEG	NEG	NEG
UKD-1001782	SCCmec I (COL)	CC5	POS	POS	POS	NEG	NEG	NEG	NEG	NEG	NEG	NEG	NEG	NEG	NEG	NEG	NEG	POS	NEG	POS	POS	NEG	NEG	NEG	NEG	NEG	NEG	NEG	NEG	NEG	NEG
Prediction for PSP1996, GenBank ANHU	SCCmec I (PSP1996)	CC8	POS	POS	POS	POS	NEG	NEG	NEG	NEG	NEG	NEG	NEG	NEG	NEG	NEG	NEG	POS	POS	NEG	NEG	NEG	NEG	NEG	NEG	POS	NEG	NEG	NEG	NEG	NEG
UKD-0261727	SCCmec I (PSP1996)	CC8	NEG	NEG	NEG	POS	NEG	NEG	NEG	NEG	NEG	NEG	NEG	NEG	NEG	NEG	NEG	POS	POS	NEG	NEG	NEG	NEG	NEG	NEG	POS	NEG	NEG	NEG	NEG	NEG
VIRTUAL HYBRIDISATION for MRSA7 : VIRTUAL HYBRII	SCCmec I+fus+tirS (Geraldine Clone)	CC5	POS	POS	POS	NEG	NEG	NEG	NEG	NEG	NEG	NEG	NEG	NEG	NEG	NEG	NEG	NEG	NEG	NEG	NEG	NEG	NEG	NEG	NEG	NEG	NEG	NEG	NEG	NEG	NEG
UKD-1221930	SCCmec I+fus+tirS (Geraldine Clone)	CC5	POS	POS	POS	NEG	NEG	POS	NEG	NEG	NEG	NEG	NEG	NEG	NEG	NEG	NEG	NEG	POS	NEG	AMB	NEG	NEG	NEG	NEG	NEG	NEG	NEG	NEG	NEG	NEG
Prediction for N315, GenBank BA000018.3	SCCmec II (N315)	CC5	POS	POS	POS	POS	NEG	NEG	NEG	NEG	NEG	NEG	NEG	NEG	NEG	NEG	NEG	NEG	POS	NEG	NEG	NEG	NEG	NEG	NEG	NEG	NEG	NEG	NEG	NEG	NEG
Reference strain N315	SCCmec II (N315)	CC5	POS	POS	POS	POS	NEG	NEG	NEG	NEG	NEG	NEG	NEG	NEG	NEG	NEG	NEG	NEG	POS	NEG	NEG	NEG	NEG	NEG	NEG	NEG	NEG	NEG	NEG	NEG	NEG
Prediction for MRSA252, GenBank BX571856.1	SCCmec II (N315)	CC30	POS	POS	POS	POS	NEG	NEG	NEG	NEG	NEG	NEG	NEG	NEG	NEG	NEG	NEG	NEG	POS	NEG	NEG	NEG	NEG	NEG	NEG	NEG	NEG	NEG	NEG	NEG	NEG
Reference strain Sanger 252	SCCmec II (N315)	CC30	POS	POS	POS	POS		NEG	NEG	NEG	NEG	NEG	NEG	NEG	NEG	NEG	NEG	NEG	POS	NEG	NEG	NEG	NEG	NEG	NEG	NEG	NEG	NEG	NEG	NEG	NEG
UKD-0230792	SCCmec II (N315)	CC30	POS	POS	POS	POS	NEG	NEG	NEG	NEG	NEG	NEG	NEG	NEG	NEG	NEG	NEG	POS	POS	NEG	NEG	POS	NEG	NEG	NEG	NEG	NEG	NEG	NEG	NEG	NEG
Prediction for Mu50, GenBank BA000017.4	SCCmec II (N315)	CC5	NEG	NEG	NEG	POS	NEG	NEG	NEG	NEG	NEG	NEG	NEG	NEG	NEG	NEG	NEG	POS	POS	NEG	NEG	NEG	NEG	NEG	NEG	POS	NEG	NEG	NEG	NEG	NEG
Reference strain MU50	SCCmec II (N315)	CC5	NEG	NEG	NEG	POS	NEG	NEG	NEG	NEG	NEG	NEG	NEG	NEG	NEG	NEG	NEG	POS	POS	NEG	NEG	NEG	NEG	NEG	NEG	POS	NEG	NEG	NEG	NEG	NEG
Prediction for JH1, GenBank CP000736.1	SCCmec II (JH1/JH9)	CC5	POS	POS	POS	POS	NEG	NEG	NEG	NEG	NEG	NEG	NEG	NEG	NEG	NEG	NEG	POS	NEG	NEG	NEG	NEG	NEG	NEG	NEG	NEG	NEG	NEG	NEG	NEG	NEG
Prediction for JH9, GenBank CP000703.1	SCCmec II (JH1/JH9)	CC5	POS	POS	POS	POS	NEG	NEG	NEG	NEG	NEG	NEG	NEG	NEG	NEG	NEG	NEG	POS	NEG	NEG	NEG	NEG	NEG	NEG	NEG	NEG	NEG	NEG	NEG	NEG	NEG
Reference strain Dublin-DSH_AR07.4_0237	SCCmec II (JH1/JH9)		POS	POS	POS	POS	NEG	NEG	NEG	NEG	NEG	NEG	NEG	NEG	NEG	NEG	NEG	NEG	POS	NEG	NEG	NEG	NEG	NEG	NEG	NEG	NEG	NEG	NEG	NEG	NEG
Reference strain NARSA_382 / USA100	SCCmec II (JH1/JH9)	CC5	POS	POS	POS	POS	NEG	NEG	NEG	NEG	NEG	NEG	NEG	NEG	NEG	NEG	NEG	NEG	POS	NEG	NEG	NEG	NEG	NEG	NEG	NEG	NEG	NEG	NEG	NEG	NEG
UKD-0046921	SCCmec II (JH1/JH9)	CC5	POS	POS	POS	POS	NEG	NEG	NEG	NEG	NEG	NEG	NEG	NEG	NEG	NEG	NEG	NEG	POS	NEG	NEG	NEG	NEG	NEG	NEG	NEG	NEG	NEG	NEG	NEG	NEG
UKD-0333415	SCCmec II (JH1/JH9)	CC5	NEG	NEG	NEG	POS	NEG	NEG	NEG	NEG	NEG	NEG	NEG	NEG	NEG	NEG	NEG	NEG	POS	NEG	NEG	NEG	NEG	NEG	NEG	NEG	NEG	NEG	NEG	NEG	NEG
UKD-0341645	SCCmec II (JH1/JH9)	CC5	POS	POS	POS	POS	NEG	NEG	NEG	NEG	NEG	NEG	NEG	NEG	NEG	NEG	NEG	NEG	NEG	NEG	NEG	NEG	NEG	NEG	NEG	NEG	NEG	NEG	NEG	NEG	NEG
UKD-0504626	SCCmec II (JH1/JH9)	CC5	POS	POS	POS	POS		NEG	NEG	NEG	NEG	NEG	NEG	NEG	NEG	NEG	NEG	NEG	POS	NEG	NEG	NEG	NEG	NEG	NEG	NEG	NEG	NEG	NEG	NEG	NEG
UKD-0507581	SCCmec II (JH1/JH9)	CC5	POS	POS	POS	POS		NEG	NEG	NEG	NEG	NEG	NEG	NEG	NEG	NEG	NEG	NEG	POS	NEG	NEG	NEG	NEG	NEG	NEG	NEG	NEG	NEG	NEG	NEG	NEG
UKD-0507903	SCCmec II (JH1/JH9)	CC5	NEG	NEG	NEG	POS		NEG	NEG	NEG	NEG	NEG	NEG	NEG	NEG	NEG	NEG	NEG	POS	NEG	NEG	NEG	NEG	NEG	NEG	NEG	NEG	NEG	NEG	NEG	NEG
UKD-0700817	SCCmec II (JH1/JH9)	CC5	NEG	NEG	NEG	POS		NEG	NEG	NEG	NEG	NEG	NEG	NEG	NEG	NEG	NEG	NEG	NEG	NEG	NEG	NEG	NEG	NEG	NEG	NEG	NEG	NEG	NEG	NEG	NEG
UKD-1014561	SCCmec II (JH1/JH9)	CC5	NEG	NEG	NEG	POS	NEG	NEG	NEG	NEG	NEG	NEG	NEG	NEG	NEG	NEG	NEG	NEG	POS	NEG	NEG	NEG	NEG	NEG	NEG	NEG	NEG	NEG	NEG	NEG	NEG
UKD-1037507	SCCmec II (JH1/JH9)	CC5	POS	POS	POS	POS	NEG	NEG	NEG	NEG	NEG	NEG	NEG	NEG	NEG	NEG	NEG	NEG	NEG	NEG	NEG	NEG	NEG	NEG	NEG	NEG	NEG	NEG	NEG	NEG	NEG
UKD-1038321	SCCmec II (JH1/JH9)	CC5	POS	POS	POS	POS	NEG	NEG	NEG	NEG	NEG	NEG	NEG	NEG	NEG	NEG	NEG	NEG	NEG	NEG	NEG	NEG	NEG	NEG	NEG	NEG	NEG	NEG	NEG	NEG	NEG
UKD-1111608	SCCmec II (JH1/JH9)	CC5	POS	POS	POS	POS	NEG	NEG	NEG	NEG	NEG	NEG	NEG	NEG	NEG	NEG	NEG	NEG	POS	NEG	NEG	NEG	NEG	NEG	NEG	NEG	NEG	NEG	NEG	NEG	NEG
UKD-12753135	SCCmec II (JH1/JH9)	CC5	NEG	NEG	NEG	POS	NEG	NEG	NEG	NEG	NEG	NEG	NEG	NEG	NEG	NEG	NEG	NEG	NEG	NEG	NEG	NEG	NEG	NEG	NEG	NEG	NEG	NEG	NEG	NEG	NEG
UKD-1312816	SCCmec II (JH1/JH9)	CC5	POS	POS	POS	POS	NEG	NEG	NEG	NEG	NEG	NEG	NEG	NEG	NEG	NEG	NEG	NEG	POS	NEG	NEG	NEG	NEG	NEG	NEG	NEG	NEG	NEG	NEG	NEG	NEG
UKD-1401623	SCCmec II (JH1/JH9)	CC5	POS	POS	POS	POS	NEG	NEG	NEG	NEG	NEG	NEG	NEG	NEG	AMB	NEG	NEG	NEG	NEG	NEG	NEG	NEG	NEG	NEG	NEG	NEG	NEG	NEG	NEG	NEG	NEG
UKD-15571943	SCCmec II+czrC+ccrA/B-4	CC5	NEG	NEG	NEG	POS	NEG	NEG	NEG	NEG	NEG	NEG	NEG	NEG	POS	NEG	NEG	NEG	POS	NEG	NEG	NEG	NEG	NEG	NEG	NEG	NEG	NEG	NEG	NEG	NEG
UKD-15576888	SCCmec II+czrC+ccrA/B-4	CC5	NEG	NEG	NEG	POS	NEG	NEG	NEG	NEG	NEG	NEG	NEG	NEG	AMB	NEG	NEG	NEG	POS	NEG	NEG	NEG	NEG	NEG	NEG	NEG	NEG	NEG	NEG	NEG	NEG
UKD-14576461	SCCmec II+speG+czrC+ccrA/B-4	CC5	POS	POS	POS	POS	NEG	NEG	NEG	NEG	NEG	NEG	NEG	NEG	NEG	NEG	NEG	NEG	POS	NEG	NEG	NEG	NEG	NEG	NEG	NEG	NEG	NEG	NEG	NEG	NEG
UKD-15581649	SCCmec II+speG+czrC+ccrA/B-4	CC5	POS	POS	POS	POS	NEG	NEG	NEG	NEG	NEG	NEG	NEG	NEG	NEG	NEG	NEG	NEG	POS	NEG	NEG	NEG	NEG	NEG	NEG	NEG	NEG	NEG	NEG	NEG	NEG
Prediction for CIGC345D, GenBank AHVO	SCCmec II A/B/D	CC8	POS	POS	POS	POS	NEG	NEG	NEG	NEG	NEG	NEG	NEG	NEG	NEG	NEG	NEG	NEG	NEG	NEG	NEG	NEG	NEG	NEG	NEG	NEG	NEG	NEG	NEG	NEG	NEG
UKD-12K00171	SCCmec II A/B/D	CC8	POS	POS	POS	POS	NEG	NEG	NEG	NEG	NEG	NEG	NEG	NEG	NEG	NEG	NEG	NEG	NEG	NEG	NEG	NEG	NEG	NEG	NEG	NEG	NEG	NEG	NEG	NEG	NEG
Prediction for JKD6008, GenBank CP002120.1	SCC [mec III+Cd] (JKD6008)	CC8 (ST239)	POS	POS	POS	POS	NEG	NEG	NEG	NEG	NEG	NEG	NEG	NEG	NEG	NEG	NEG	POS	POS	NEG	NEG	POS	NEG	NEG	NEG	POS	NEG	NEG	NEG	NEG	NEG
Prediction for JKD6009, GenBank ABSA	SCC [mec III+Cd] (JKD6008)	CC8 (ST239)	POS	POS	POS	POS	NEG	NEG	NEG	NEG	NEG	NEG	NEG	NEG	NEG	NEG	NEG	POS	POS	NEG	NEG	POS	NEG	NEG	NEG	POS	NEG	NEG	NEG	NEG	NEG
Reference strain ATCC_33592	SCC [mec III+Cd] (ATCC33592)	CC8 (ST239)	POS	POS	POS	POS	NEG	POS	NEG	NEG	NEG	NEG	NEG	NEG	NEG	NEG	NEG	POS	POS	POS	POS	NEG	NEG	NEG	POS	POS	POS	NEG	NEG	POS	NEG
Prediction for PPUKM-775-2009, GenBank AMRE	SCC [mec III+Cd+ccrC] (PPUKM-775-2009)	CC8 (ST239)	POS	POS	POS	POS	NEG	NEG	NEG	NEG	NEG	NEG	NEG	NEG	NEG	NEG	NEG	POS	NEG	POS	POS	NEG	NEG	NEG	NEG	POS	NEG	NEG	NEG	NEG	NEG
Prediction for T0131, GenBank CP002643.1	SCC [mec III+Cd+ccrC] (T0131)	CC8 (ST239)	POS	POS	POS	POS	NEG	NEG	NEG	NEG	NEG	NEG	NEG	NEG	NEG	NEG	NEG	NEG	NEG	NEG	NEG	NEG	NEG	NEG	NEG	POS	NEG	NEG	NEG	NEG	NEG
Prediction for XN108, GenBank CP007447.1	SCC [mec III+Cd+ccrC] (XN108)	CC8 (ST239)	POS	POS	POS	POS	NEG	NEG	NEG	NEG	NEG	NEG	NEG	NEG	NEG	NEG	NEG	POS	NEG	POS	POS	NEG	NEG	NEG	NEG	POS	NEG	NEG	NEG	NEG	NEG
Prediction for CN79, GenBank ANCJ	SCC [mec III+Cd+ccrC] (CN79)	CC8 (ST239)	POS	POS	POS	NEG	NEG	NEG	NEG	NEG	NEG	NEG	NEG	NEG	NEG	NEG	NEG	NEG	NEG	NEG	NEG	NEG	NEG	NEG	NEG	POS	NEG	NEG	NEG	NEG	NEG
Prediction for 16K, GenBank BABZ	SCC [mec III+Cd+ccrC] (CN79)	CC8 (ST239)	POS	POS	POS	POS	NEG	NEG	NEG	NEG	NEG	NEG	NEG	NEG	NEG	NEG	NEG	POS	NEG	NEG	NEG	NEG	NEG	NEG	NEG	POS	POS	NEG	NEG	POS	NEG
UKD-0835987	SCC [mec III+Cd+ccrC] (CN79)	CC8 (ST239)	POS	POS	POS	POS	NEG	NEG	NEG	NEG	NEG	NEG	NEG	NEG	NEG	NEG	NEG	POS	NEG	NEG	NEG	NEG	NEG	NEG	NEG	POS	NEG	NEG	NEG	NEG	NEG
UKD-16581769	SCCmec III+Cd+ccrC	CC8 (ST239)	POS	POS	POS	POS	NEG	NEG	NEG	NEG	NEG	NEG	NEG	NEG	NEG	NEG	NEG	POS	NEG	NEG	NEG	NEG	NEG	NEG	NEG	POS	NEG	NEG	NEG	NEG	NEG
Prediction for Bmb9393, GenBank CP005288.1	SCCmec III+Cd/Hg+ccrC (Bmb9393)	CC8 (ST239)	POS	POS	POS	POS	NEG	NEG	NEG	NEG	NEG	NEG	NEG	NEG	NEG	NEG	NEG	POS	NEG	POS	POS	NEG	NEG	NEG	NEG	POS	POS	NEG	NEG	POS	NEG
UKD-15574263	SCCmec III+Cd/Hg+ccrC (Bmb9393)	CC8 (ST239)	POS	POS	POS	POS	NEG	NEG	NEG	NEG	NEG	NEG	NEG	NEG	NEG	NEG	NEG	POS	NEG	POS	POS	NEG	NEG	NEG	NEG	POS	NEG	NEG	NEG	NEG	NEG
Prediction for SK1585, GenBank AYLT	SCCmec III+ccrC+Cd/Hg (SK1585)	CC8 (ST239)	POS	POS	POS	POS	NEG	NEG	NEG	NEG	NEG	NEG	NEG	NEG	NEG	NEG	NEG	NEG	NEG	POS	POS	NEG	NEG	NEG	POS	POS	NEG	NEG	NEG	NEG	NEG
UKD-105861	SCCmec III+ccrC+Cd/Hg (SK1585)	CC8 (ST239)	POS	POS	POS	NEG	NEG	POS	NEG	NEG	NEG	NEG	NEG	NEG	NEG	NEG	NEG	AMB	NEG	POS	POS	NEG	NEG	NEG	NEG	POS	NEG	NEG	NEG	NEG	NEG
UKD-134572	SCCmec III+ccrC+Cd/Hg (SK1585)	CC8 (ST239)	POS	POS	POS	NEG		POS	NEG	NEG	NEG	NEG	NEG	NEG	NEG	NEG	NEG	POS	NEG	POS	POS	NEG	NEG	NEG	NEG	POS	NEG	NEG	NEG	NEG	NEG
UKD-139143	SCCmec III+ccrC+Cd/Hg (SK1585)	CC8 (ST239)	POS	POS	POS	NEG	NEG	POS	NEG	NEG	NEG	NEG	NEG	NEG	NEG	NEG	NEG	NEG	NEG	POS	POS	NEG	NEG	NEG	NEG	POS	NEG	NEG	NEG	NEG	NEG
Prediction for TW20, GenBank FN433596.1	SCC [mec III+Cd/Hg+ccrC] (TW20)	CC8 (ST239)	POS	POS	POS	POS	NEG	NEG	NEG	NEG	NEG	NEG	NEG	NEG	NEG	NEG	NEG	POS	NEG	POS	POS	NEG	NEG	NEG	POS	POS	NEG	NEG	NEG	NEG	NEG
Prediction for ATCC BAA-39, GenBank AEEK	SCC [mec III+Cd/Hg+ccrC] (TW20)	CC8 (ST239)	POS	POS	POS	POS	NEG	NEG	NEG	NEG	NEG	NEG	NEG	NEG	NEG	NEG	NEG	POS	NEG	NEG	NEG	NEG	NEG	NEG	POS	POS	NEG	NEG	NEG	NEG	NEG

Isolate ID	SCCmec type	Clonal complex	RESISTANCE : PENICILLINASE			RESISTANCE : MLS-ANTIBIOTICS														RESISTANCE : AMINOGLYCOSIDES			RESISTANCE : MISCELLANEOUS GENES										
			beta-lactamase	beta lactamase repressor (inhibitor)	beta-lactamase regulatory protein	erythromycin/clindamycin resistance			Linco-samid-Nucleo-tidyltrans-ferase	energy-dependent efflux of erythro-mycin	macrolide efflux protein A	probable lysylphos-phatidyl-glycerol synthetase	virginia-mycin A acetyltransf erase	acetyl-transferase inactivating streptogra min A	ATP binding protein, streptogramin-A-resistance		virginia-mycin B hydrolase	bifunctional enzyme Aac/Aph, gentamicin resistance	amino-glycoside adenyl-transferase, tobramycin resistance	3'5'-aminog-lycoside phospho-transferase, neo-/ kanamycin resistance	strepto-thricine-acetyl-transferase	dihydro-folate reductase type 1	fusidic acid resistence	mupirocin resistence protein	tetracycline-resistance		chloramphenicol acetyltransferase				
			blaZ	blaI	blaR	ermA	ermB	ermC	linA/ InuA	msrA	mefA	mph(C)	vat(A)	vat(B)	vga(A)	vga(A) (BM 3327)	vgb	aacA-aphD	aadD	aphA3	sat	dfrA	far1	mupA	tetK	tetM	cat	cat (pC221)	cat (pc223)	cat (pMC524)	cat (pSBK203R)
Prediction for MW2, GenBank BA000033.2	*SCCmec IVa (MW2)*	*CC1*	*POS*	*POS*	*POS*	*NEG*	*NEG*	*NEG*	*NEG*	*NEG*	*NEG*	*NEG*	*NEG*	*NEG*	*NEG*	*NEG*	*NEG*	*NEG*	*NEG*	*NEG*	*NEG*	*NEG*	*NEG*	*NEG*	*NEG*	*NEG*	*NEG*	*NEG*	*NEG*	*NEG*	*NEG*
Reference strain MW2	SCCmec IVa (MW2)	CC1	POS	POS	POS	NEG	NEG	NEG	NEG	NEG	NEG	NEG	NEG	NEG	NEG	NEG	NEG	NEG	NEG	NEG	NEG	NEG	NEG	NEG	NEG	NEG	NEG	NEG	NEG	NEG	NEG
Reference strain CA05 (JCSC1968)	SCCmec IVa (MW2)	CC45	POS	POS	POS	NEG	NEG	NEG	NEG	NEG	NEG	NEG	NEG	NEG	NEG	NEG	NEG	NEG	NEG	NEG	NEG	NEG	NEG	NEG	NEG	NEG	NEG	NEG	NEG	NEG	NEG
UKD-0127106	SCCmec IVa (MW2)	CC1	POS	POS	POS	NEG		NEG	NEG	NEG	NEG	NEG	NEG	NEG	NEG	NEG	NEG	NEG	NEG	NEG	NEG	NEG	NEG	NEG	NEG	NEG	NEG	NEG	NEG	NEG	NEG
UKD-0701446	SCCmec IVa (MW2)	CC1	POS	POS	POS	NEG	NEG	NEG	NEG	NEG	NEG	NEG	NEG	NEG	NEG	NEG	NEG	NEG	NEG	NEG	NEG	NEG	NEG	NEG	NEG	NEG	NEG	NEG	NEG	NEG	NEG
UKD-0947486	SCCmec IVa (MW2)	CC1	POS	POS	POS	AMB	NEG	POS	NEG	NEG	NEG	NEG	NEG	NEG	NEG	NEG	NEG	NEG	NEG	POS	POS	POS	NEG	NEG	POS	NEG	NEG	NEG	NEG	NEG	NEG
UKD-1031910	SCCmec IVa (MW2)	CC1	POS	POS	POS	NEG	NEG	POS	NEG	NEG	NEG	NEG	NEG	NEG	NEG	NEG	NEG	NEG	NEG	POS	POS	NEG	NEG	NEG	POS	NEG	NEG	NEG	NEG	NEG	NEG
UKD-14571328	SCCmec IVa (MW2)	CC1	POS	POS	POS	NEG	NEG	POS	NEG	NEG	NEG	NEG	NEG	NEG	NEG	NEG	NEG	POS	NEG	POS	POS	POS	NEG	POS	POS	NEG	NEG	NEG	NEG	NEG	NEG
UKD-03003012	SCCmec IVa (MW2)	CC5	POS	POS	POS	NEG		NEG	NEG	NEG	NEG	NEG	NEG	NEG	AMB	NEG	NEG	NEG	NEG	NEG	NEG	NEG	NEG	NEG	NEG	NEG	NEG	NEG	NEG	NEG	NEG
UKD-15582582	SCCmec IVa (MW2)	CC6	POS	POS	POS	NEG	NEG	POS	NEG	NEG	NEG	NEG	NEG	NEG	AMB	NEG	NEG	NEG	NEG	NEG	NEG	NEG	NEG	NEG	NEG	NEG	NEG	NEG	NEG	NEG	NEG
UKD-16574230	SCCmec IVa (MW2)	CC6	POS	POS	POS	NEG	NEG	NEG	NEG	NEG	NEG	NEG	NEG	NEG	NEG	NEG	NEG	NEG	NEG	NEG	NEG	NEG	NEG	NEG	NEG	NEG	NEG	NEG	NEG	NEG	NEG
UKD-16582560	SCCmec IVa (MW2)	CC6	POS	POS	POS	NEG	NEG	POS	NEG	NEG	NEG	NEG	NEG	NEG	NEG	NEG	NEG	NEG	NEG	NEG	NEG	NEG	NEG	NEG	NEG	NEG	NEG	NEG	NEG	NEG	NEG
UKD-0802855	SCCmec IVa (MW2)	CC7	POS	POS	POS	NEG	NEG	NEG	NEG	NEG	NEG	NEG	NEG	NEG	NEG	NEG	NEG	NEG	NEG	NEG	NEG	NEG	NEG	NEG	NEG	NEG	NEG	NEG	NEG	NEG	NEG
KHDN-10405258	SCCmec IVa (MW2)	CC8	POS	POS	POS	NEG	NEG	NEG	NEG	POS	NEG	POS	NEG	NEG	NEG	NEG	NEG	NEG	NEG	POS	POS	NEG	NEG	NEG	NEG	NEG	NEG	NEG	NEG	NEG	NEG
UKD-15573497	SCCmec IVa (MW2)	CC22	POS	POS	POS	NEG	NEG	NEG	NEG	NEG	NEG	NEG	NEG	NEG	POS	NEG	NEG	POS	NEG	NEG	NEG	POS	NEG	NEG	NEG	NEG	NEG	NEG	NEG	NEG	NEG
UKD-0634729	SCCmec IVa (MW2)	CC30	POS	POS	POS	NEG		NEG	NEG	NEG	NEG	NEG	NEG	NEG	NEG	NEG	NEG	NEG	NEG	NEG	NEG	NEG	NEG	NEG	NEG	NEG	NEG	NEG	NEG	NEG	NEG
UKD-1119771	SCCmec IVa (MW2)	CC30	POS	POS	POS	NEG	NEG	NEG	NEG	NEG	NEG	NEG	NEG	NEG	AMB	NEG	NEG	NEG	NEG	NEG	NEG	NEG	NEG	NEG	NEG	NEG	NEG	NEG	NEG	NEG	NEG
KHDN-10411458	SCCmec IVa (MW2)	CC30	POS	POS	POS	NEG	NEG	NEG	NEG	NEG	NEG	NEG	NEG	NEG	NEG	NEG	NEG	NEG	NEG	NEG	NEG	NEG	NEG	NEG	NEG	NEG	NEG	NEG	NEG	NEG	NEG
KHDN-398609	SCCmec IVa (MW2)	CC30	POS	POS	POS	NEG	NEG	NEG	NEG	NEG	NEG	NEG	NEG	NEG	NEG	NEG	NEG	NEG	NEG	NEG	NEG	NEG	NEG	NEG	NEG	NEG	NEG	NEG	NEG	NEG	NEG
KHDN-01081998	SCCmec IVa (MW2)	CC30	POS	POS	POS	NEG	NEG	NEG	NEG	NEG	NEG	NEG	NEG	NEG	NEG	NEG	NEG	NEG	NEG	NEG	NEG	NEG	NEG	NEG	NEG	NEG	NEG	NEG	NEG	NEG	NEG
KHDN-10399223	SCCmec IVa (MW2)	CC30	POS	POS	POS	NEG	NEG	NEG	NEG	NEG	NEG	NEG	NEG	NEG	NEG	NEG	NEG	NEG	NEG	NEG	NEG	NEG	NEG	NEG	NEG	NEG	NEG	NEG	NEG	NEG	NEG
KHDN-W261158	SCCmec IVa (MW2)	CC30	POS	POS	POS	NEG	NEG	NEG	NEG	NEG	NEG	NEG	NEG	NEG	NEG	NEG	NEG	NEG	NEG	NEG	NEG	NEG	NEG	NEG	NEG	NEG	NEG	NEG	NEG	NEG	NEG
UKD-0002046	SCCmec IVa (MW2)	CC45 [agr I]	POS	POS	POS	NEG	NEG	NEG	NEG	NEG	NEG	NEG	NEG	NEG	NEG	NEG	NEG	NEG	NEG	POS	POS	NEG	NEG	NEG	NEG	NEG	NEG	NEG	NEG	NEG	NEG
UKD-0029618	SCCmec IVa (MW2)	CC45 [agr I]	POS	POS	POS	NEG	NEG	NEG	NEG	NEG	NEG	NEG	NEG	NEG	NEG	NEG	NEG	NEG	NEG	POS	POS	NEG	NEG	NEG	NEG	NEG	NEG	NEG	NEG	NEG	NEG
UKD-0043085	SCCmec IVa (MW2)	CC45 [agr I]	POS	POS	POS	NEG	NEG	POS	NEG	NEG	NEG	NEG	NEG	NEG	NEG	NEG	NEG	NEG	NEG	POS	POS	NEG	NEG	NEG	NEG	NEG	NEG	NEG	NEG	NEG	NEG
UKD-0047053	SCCmec IVa (MW2)	CC45 [agr I]	POS	POS	POS	NEG		NEG	NEG	NEG	NEG	NEG	NEG	NEG	NEG	NEG	NEG	NEG	NEG	POS	POS	NEG	NEG	NEG	NEG	NEG	NEG	NEG	NEG	NEG	NEG
UKD-0047429	SCCmec IVa (MW2)	CC45 [agr I]	POS	POS	POS	NEG	NEG	POS	NEG	NEG	NEG	NEG	NEG	NEG	NEG	NEG	NEG	POS	NEG	POS	POS	POS	NEG	NEG	NEG	NEG	NEG	NEG	NEG	NEG	NEG
UKD-0112465	SCCmec IVa (MW2)	CC45 [agr I]	POS	POS	POS	NEG	NEG	NEG	NEG	NEG	NEG	NEG	NEG	NEG	NEG	NEG	NEG	NEG	NEG	POS	POS	NEG	NEG	NEG	NEG	NEG	NEG	NEG	NEG	NEG	NEG
UKD-0103483	SCCmec IVa (MW2)	CC45 [agr I]	POS	POS	POS	NEG	NEG	POS	NEG	NEG	NEG	NEG	NEG	NEG	NEG	NEG	NEG	NEG	NEG	POS	POS	NEG	NEG	NEG	NEG	NEG	NEG	NEG	NEG	NEG	NEG
UKD-0305014	SCCmec IVa (MW2)	CC45 [agr I]	POS	POS	POS	NEG	NEG	NEG	NEG	NEG	NEG	NEG	NEG	NEG	NEG	NEG	NEG	NEG	NEG	POS	POS	NEG	NEG	NEG	NEG	NEG	NEG	NEG	NEG	NEG	NEG
UKD-0401119	SCCmec IVa (MW2)	CC45 [agr I]	POS	POS	POS	NEG	NEG	NEG	NEG	NEG	NEG	NEG	NEG	NEG	NEG	NEG	NEG	NEG	NEG	NEG	NEG	NEG	NEG	NEG	NEG	NEG	NEG	NEG	NEG	NEG	NEG
UKD-0537969	SCCmec IVa (MW2)	CC45 [agr I]	POS	POS	POS	NEG		NEG	NEG	NEG	NEG	NEG	NEG	NEG	NEG	NEG	NEG	NEG	NEG	POS	POS	NEG	NEG	NEG	NEG	NEG	NEG	NEG	NEG	NEG	NEG
UKD-0703373	SCCmec IVa (MW2)	CC45 [agr I]	POS	AMB	POS	NEG		NEG	NEG	NEG	NEG	NEG	NEG	NEG	NEG	NEG	NEG	NEG	NEG	NEG	NEG	NEG	NEG	NEG	NEG	NEG	NEG	NEG	NEG	NEG	NEG
UKD-0813653	SCCmec IVa (MW2)	CC45 [agr I]	POS	POS	POS	NEG	NEG	NEG	NEG	NEG	NEG	NEG	NEG	NEG	NEG	NEG	NEG	NEG	NEG	POS	POS	NEG	NEG	NEG	NEG	NEG	NEG	NEG	NEG	NEG	NEG
UKD-0839175	SCCmec IVa (MW2)	CC45 [agr I]	POS	POS	POS	NEG	AMB	POS	NEG	NEG	NEG	NEG	NEG	NEG	AMB	NEG	NEG	NEG	NEG	POS	POS	NEG	NEG	NEG	NEG	NEG	NEG	NEG	NEG	NEG	NEG
UKD-1108422	SCCmec IVa (MW2)	CC45 [agr I]	NEG	NEG	NEG	NEG	NEG	NEG	NEG	NEG	NEG	NEG	NEG	NEG	NEG	NEG	NEG	NEG	NEG	POS	POS	NEG	NEG	NEG	NEG	NEG	NEG	NEG	NEG	NEG	NEG
UKD-1380645	SCCmec IVa (MW2)	CC45 [agr I]	POS	POS	POS	NEG	NEG	POS	NEG	NEG	NEG	NEG	NEG	NEG	NEG	NEG	NEG	NEG	NEG	NEG	NEG	NEG	NEG	NEG	NEG	NEG	POS	NEG	NEG	NEG	POS
UKD-148016	SCCmec IVa (MW2)	CC45 [agr I]	POS	POS	POS	NEG	NEG	NEG	NEG	NEG	NEG	NEG	NEG	NEG	NEG	NEG	NEG	NEG	NEG	NEG	NEG	NEG	NEG	NEG	NEG	NEG	POS	NEG	NEG	NEG	POS
UKD-16571773	SCCmec IVa (MW2)	CC45 [agr I]	POS	POS	POS	NEG	NEG	POS	NEG	NEG	NEG	NEG	NEG	NEG	POS	NEG	NEG	NEG	NEG	NEG	NEG	NEG	NEG	NEG	NEG	NEG	NEG	NEG	NEG	NEG	NEG
KHDN-10407738	SCCmec IVa (MW2)	CC45 [ST617]	POS	POS	POS	NEG	NEG	NEG	NEG	NEG	NEG	NEG	NEG	NEG	NEG	NEG	NEG	POS	NEG	NEG	NEG	NEG	NEG	NEG	NEG	POS	NEG	NEG	NEG	NEG	NEG
UKD-0853670	SCCmec IVa (MW2)	CC88	POS	POS	POS	POS	NEG	NEG	NEG	NEG	NEG	NEG	NEG	NEG	NEG	NEG	NEG	NEG	NEG	NEG	NEG	NEG	NEG	NEG	NEG	NEG	NEG	NEG	NEG	NEG	NEG
UKD-1417352	SCCmec IVa (MW2)	CC88	POS	POS	POS	NEG	NEG	POS	NEG	NEG	NEG	NEG	NEG	NEG	NEG	NEG	NEG	NEG	NEG	NEG	NEG	NEG	NEG	NEG	NEG	NEG	NEG	NEG	NEG	NEG	NEG
UKD-15571283	SCCmec IVa (MW2)	CC88	POS	POS	POS	NEG	NEG	NEG	NEG	NEG	NEG	NEG	NEG	NEG	POS	NEG	NEG	NEG	NEG	NEG	NEG	NEG	NEG	NEG	POS	NEG	NEG	NEG	NEG	NEG	NEG
KHDN-10406719	SCCmec IVa (MW2)	ST93	POS	POS	POS	NEG	NEG	NEG	NEG	NEG	NEG	NEG	NEG	NEG	NEG	NEG	NEG	NEG	NEG	NEG	NEG	NEG	NEG	NEG	NEG	NEG	NEG	NEG	NEG	NEG	NEG
KHDN-0060294	SCCmec IVa (MW2)	ST93	POS	POS	POS	NEG	NEG	NEG	NEG	NEG	NEG	NEG	NEG	NEG	NEG	NEG	NEG	NEG	NEG	NEG	NEG	NEG	NEG	NEG	NEG	NEG	NEG	NEG	NEG	NEG	NEG
UKD-0734190	SCCmec IVa (MW2)	CC97	POS	POS	POS	NEG	NEG	NEG	NEG	NEG	NEG	NEG	NEG	NEG	NEG	NEG	NEG	NEG	POS	NEG	NEG	NEG	NEG	NEG	POS	NEG	NEG	NEG	NEG	NEG	NEG
UKD-13006983	SCCmec IVa (MW2)	CC97	POS	POS	POS	NEG	NEG	NEG	NEG	NEG	NEG	NEG	NEG	NEG	POS	NEG	NEG	NEG	NEG	NEG	NEG	NEG	NEG	NEG	NEG	NEG	NEG	NEG	NEG	NEG	NEG
Prediction for H131520133	*SCCmec IVa (H131520133)*	*CC152*	*POS*	*POS*	*POS*	*NEG*	*NEG*	*NEG*	*NEG*	*NEG*	*NEG*	*NEG*	*NEG*	*NEG*	*NEG*	*NEG*	*NEG*	*NEG*	*NEG*	*NEG*	*NEG*	*NEG*	*NEG*	*NEG*	*POS*	*NEG*	*POS*	*POS*	*NEG*	*NEG*	*NEG*
KHDN-325892	SCCmec IVa (H131520133)	CC30	POS	POS	POS	NEG	NEG	NEG	NEG	NEG	NEG	NEG	NEG	NEG	NEG	NEG	NEG	NEG	NEG	NEG	NEG	NEG	NEG	NEG	NEG	NEG	NEG	NEG	NEG	NEG	NEG
UKD-15580207	SCCmec IVa (H131520133)	CC88	POS	POS	POS	NEG	NEG	NEG	NEG	NEG	NEG	NEG	NEG	NEG	POS	NEG	NEG	NEG	NEG	NEG	NEG	NEG	NEG	NEG	NEG	NEG	NEG	NEG	NEG	NEG	NEG
UKD-0521159	SCCmec Iva	CC8	POS	POS	POS	NEG		POS	NEG	NEG	NEG	NEG	NEG	NEG	NEG	NEG	NEG	NEG	NEG	NEG	NEG	NEG	NEG	NEG	NEG	NEG	NEG	NEG	NEG	NEG	NEG
Prediction for CMFT503 : HF569113.1	*SCCmec IVa (CMFT503)*																														
Prediction for 21343, GenBank AHKV	*SCCmec IVa (CMFT503)*	*CC88*	*POS*	*POS*	*POS*	*NEG*	*NEG*	*POS*	*NEG*	*NEG*	*NEG*	*NEG*	*NEG*	*NEG*	*NEG*	*NEG*	*NEG*	*NEG*	*NEG*	*NEG*	*NEG*	*POS*	*NEG*	*NEG*	*NEG*	*NEG*	*NEG*	*NEG*	*NEG*	*NEG*	*NEG*
UKD-1420740	SCCmec IVa (CMFT503)	CC22	POS	POS	POS	NEG	NEG	NEG	NEG	NEG	NEG	NEG	NEG	NEG	POS	NEG	NEG	NEG	NEG	NEG	NEG	POS	NEG	NEG	NEG	NEG	NEG	NEG	NEG	NEG	NEG
UKD-15575124	SCCmec IVa (CMFT503)	CC22	POS	POS	POS	NEG	NEG	NEG	NEG	NEG	NEG	NEG	NEG	NEG	NEG	NEG	NEG	NEG	NEG	NEG	NEG	POS	NEG	NEG	NEG	NEG	NEG	NEG	NEG	NEG	NEG
UKD-16584947	SCCmec IVa (CMFT503)	CC22	POS	POS	POS	NEG	NEG	NEG	NEG	NEG	NEG	NEG	NEG	NEG	NEG	NEG	NEG	NEG	NEG	NEG	NEG	POS	NEG	NEG	NEG	NEG	NEG	NEG	NEG	NEG	NEG
UKD-16575077	SCCmec IVa (CMFT503)	CC88	POS	POS	POS	NEG	NEG	NEG	NEG	NEG	NEG	NEG	NEG	NEG	NEG	NEG	NEG	NEG	NEG	NEG	NEG	POS	NEG	NEG	POS	NEG	NEG	NEG	NEG	NEG	NEG
Prediction for TCH1516, GenBank CP000730.1	*SCCmec IVa+ACME1+Cu (USA300)*	*CC8*	*POS*	*POS*	*POS*	*NEG*	*NEG*	*NEG*	*NEG*	*POS*	*NEG*	*POS*	*NEG*	*NEG*	*NEG*	*NEG*	*NEG*	*NEG*	*NEG*	*POS*	*POS*	*NEG*	*NEG*	*NEG*	*NEG*	*NEG*	*NEG*	*NEG*	*NEG*	*NEG*	*NEG*
Reference strain USA300-TCH1516	SCCmec IVa+ACME1+Cu (USA300)	CC8	POS	POS	POS	NEG	NEG	NEG	NEG	POS	NEG	POS	NEG	NEG	NEG	NEG	NEG	NEG	NEG	POS	POS	NEG	NEG	NEG	NEG	NEG	NEG	NEG	NEG	NEG	NEG
Prediction for FPR3757, GenBank CP000255.1	*SCCmec IVa+ACME1+Cu (USA300)*	*CC8*	*NEG*	*NEG*	*NEG*	*NEG*	*NEG*	*POS*	*NEG*	*NEG*	*NEG*	*NEG*	*NEG*	*NEG*	*NEG*	*NEG*	*NEG*	*NEG*	*NEG*	*NEG*	*NEG*	*NEG*	*NEG*	*POS*	*POS*	*NEG*	*NEG*	*NEG*	*NEG*	*NEG*	*NEG*
UKD-0516386	SCCmec IVa+ACME1+Cu (USA300)	CC8	POS	POS	POS	NEG		NEG	NEG	POS	NEG	POS	NEG	NEG	NEG	NEG	NEG	NEG	NEG	POS	POS	NEG	NEG	NEG	NEG	NEG	NEG	NEG	NEG	NEG	NEG
UKD-0815773	SCCmec IVa+ACME1+Cu (USA300)	CC8	POS	POS	POS	NEG	NEG	NEG	NEG	NEG	NEG	NEG	NEG	NEG	NEG	NEG	NEG	NEG	NEG	NEG	NEG	NEG	NEG	NEG	NEG	NEG	NEG	NEG	NEG	NEG	NEG
UKD-1414654	SCCmec IVa+ACME1+Cu (USA300)	CC8	POS	POS	POS	NEG	NEG	NEG	NEG	POS	NEG	POS	NEG	NEG	NEG	NEG	NEG	NEG	NEG	POS	POS	NEG	NEG	NEG	NEG	NEG	NEG	NEG	NEG	NEG	NEG
UKD-0408036	SCCmec IVa+ACME2	CC45 [agr I]	POS	POS	POS	NEG	NEG	NEG	NEG	NEG	NEG	NEG	NEG	NEG	AMB	NEG	NEG	NEG	NEG	POS	POS	NEG	NEG	NEG	NEG	NEG	NEG	NEG	NEG	NEG	NEG
UKD-0044035	SCCmec IVa+ccrC (Hannover)	CC8	POS	POS	POS	POS		NEG	NEG	NEG	NEG	NEG	NEG	NEG	NEG	NEG	NEG	POS	NEG	POS	POS	NEG	NEG	NEG	NEG	POS	NEG	NEG	NEG	NEG	NEG
UKD-0136287	SCCmec IVa+ccrC (Hannover)	CC8	POS	POS	POS	POS	NEG	NEG	NEG	NEG	NEG	NEG	NEG	NEG	NEG	NEG	NEG	POS	NEG	POS	POS	NEG	NEG	NEG	NEG	POS	NEG	NEG	NEG	NEG	NEG

Isolate ID	SCCmec type	Clonal complex	RESISTANCE : PENICILLINASE			RESISTANCE : MLS-ANTIBIOTICS												RESISTANCE : AMINOGLYSOSIDES			RESISTANCE : MISCELLANEOUS GENES										
			beta-lactamase	beta lactamase repressor (inhibitor)	beta-lactamase regulatory protein	erythromycin/clindamycin resistance			Linco-samid-Nucleo-tidyltrans-ferase	energy-dependent efflux of erythro-mycin	macrolide efflux protein A	probable lysylphos-phatidyl-glycerol synthetase	virginia-mycin A acetyltransf erase	acetyl-transferase inactivating streptogra min A	ATP binding protein, streptogramin-A-resistance		virginia-mycin B hydrolase	bifunctional enzyme Aac/Aph, gentamicin resistance	amino-glycoside adenyl-transferase, tobramycin resistance	3'5'-aminog-lycoside phospho-transferase, neo-/kanamycin resistance	strepto-thricine-acetyl-transferase	dihydro-folate reductase type 1	fusidic acid resistence	mupirocin resistence protein	tetracycline-resistance		chloramphenicol acetyltransferase				
			blaZ	blaI	blaR	ermA	ermB	ermC	linA/ lnuA	msrA	mefA	mph(C)	vat(A)	vat(B)	vga(A)	vga(A) (BM 3327)	vgb	aacA-aphD	aadD	aphA3	sat	dfrA	far1	mupA	tetK	tetM	cat	cat (pC221)	cat (pc223)	cat (pMC524)	cat (pSBK203R)
Prediction for JCSC1978, GenBank AB063173.1	SCCmec IVb/d/i (JCSC1978/6668/4469)																														
Reference strain JCSC1978 (8/6-3P)	SCCmec IVb/d/i (JCSC1978/6668/4469)	CC8	POS	POS	POS	NEG	NEG	NEG	NEG	NEG	NEG	NEG	NEG	NEG	NEG	NEG	NEG	NEG	NEG	NEG	NEG	NEG	NEG	NEG	NEG	NEG	NEG	NEG	NEG	NEG	NEG
Prediction for JCSC6668, GenBank AB425823.1	SCCmec IVb/d/i (JCSC1978/6668/4469)																														
Reference strain JCSC4469	SCCmec IVb/d/i (JCSC1978/6668/4469)	CC5	POS	POS	POS	POS	NEG	NEG	NEG	NEG	NEG	NEG	NEG	NEG	NEG	NEG	NEG	POS	NEG	NEG	NEG	NEG	NEG	NEG	NEG	NEG	NEG	NEG	NEG	NEG	NEG
UKD-1103429	SCCmec IVb/d/i (JCSC1978/6668/4469)	CC7	POS	POS	POS	NEG	NEG	NEG	NEG	NEG	NEG	NEG	NEG	NEG	NEG	NEG	NEG	NEG	NEG	NEG	NEG	NEG	NEG	NEG	NEG	NEG	NEG	NEG	NEG	NEG	NEG
UKD-1306141	SCCmec IVb/d/i (JCSC1978/6668/4469)	CC7	POS	POS	POS	NEG	NEG	NEG	NEG	NEG	NEG	NEG	NEG	NEG	NEG	NEG	NEG	NEG	NEG	NEG	NEG	NEG	NEG	NEG	NEG	NEG	NEG	NEG	NEG	NEG	NEG
UKD-0500089	SCCmec IVb/d/i (JCSC1978/6668/4469)	CC5	POS	POS	POS	NEG		POS	NEG	NEG	NEG	NEG	NEG	NEG	NEG	NEG	NEG	NEG	POS	NEG	NEG	NEG	NEG	NEG	NEG	NEG	NEG	NEG	NEG	NEG	NEG
UKD-0501416	SCCmec IVb/d/i (JCSC1978/6668/4469)	CC5	POS	POS	POS	NEG	NEG	AMB	NEG	NEG	NEG	NEG	NEG	NEG	NEG	NEG	NEG	NEG	NEG	NEG	NEG	NEG	NEG	NEG	NEG	NEG	NEG	NEG	NEG	NEG	NEG
UKD-0934574	SCCmec IVb/d/i (JCSC1978/6668/4469)	CC5	POS	POS	POS	POS	NEG	NEG	NEG	NEG	NEG	NEG	NEG	NEG	NEG	NEG	NEG	NEG	NEG	NEG	NEG	NEG	NEG	NEG	NEG	NEG	NEG	NEG	NEG	NEG	NEG
UKD-0746779	SCCmec IVb/d/i (JCSC1978/6668/4469)	CC45 [agr I]	POS	POS	POS	POS		NEG	NEG	NEG	NEG	NEG	NEG	NEG	NEG	NEG	NEG	NEG	NEG	NEG	NEG	NEG	NEG	NEG	NEG	NEG	NEG	NEG	NEG	NEG	NEG
UKD-0812518	SCCmec IVb/d/i (JCSC1978/6668/4469)	CC45 [agr I]	POS	POS	POS	POS	NEG	NEG	NEG	NEG	NEG	NEG	NEG	NEG	NEG	NEG	NEG	NEG	NEG	NEG	NEG	NEG	NEG	NEG	NEG	NEG	NEG	NEG	NEG	NEG	NEG
Prediction for 21209, GenBank AGRP	SCCmec IVb/d/i (21209)	CC8	POS	POS	POS	NEG	NEG	NEG	NEG	NEG	NEG	NEG	NEG	NEG	NEG	NEG	NEG	POS	POS	NEG	NEG	POS	NEG	NEG	NEG	POS	NEG	NEG	NEG	NEG	NEG
UKD-0233579	SCCmec IVb/d/i (21209)	CC8	POS	POS	POS	NEG		POS	NEG	NEG	NEG	NEG	NEG	NEG	NEG	NEG	NEG	POS	NEG	NEG	NEG	POS	NEG	NEG	NEG	POS	NEG	NEG	NEG	NEG	NEG
UKD-0504920	SCCmec IVb/d/i (21209)	CC8	POS	POS	POS	NEG		POS	NEG	NEG	NEG	NEG	NEG	NEG	NEG	NEG	NEG	POS	NEG	NEG	NEG	NEG	NEG	NEG	NEG	POS	NEG	NEG	NEG	NEG	NEG
UKD-0725999	SCCmecIV b/d/i+Cu	CC30	POS	POS	POS	NEG	NEG	NEG	NEG	NEG	NEG	NEG	NEG	NEG	NEG	NEG	NEG	POS	NEG	NEG	NEG	NEG	NEG	NEG	POS	NEG	NEG	NEG	NEG	NEG	NEG
Prediction for IS-105, GenBank AHLR	SCCmec IVc (IS-105)	CC22	POS	POS	AMB	NEG	NEG	POS	NEG	NEG	NEG	NEG	NEG	NEG	NEG	NEG	NEG	POS	POS	NEG	NEG	POS	NEG	NEG	NEG	NEG	NEG	NEG	NEG	NEG	NEG
UKD-0402421	SCCmec IVc (IS-105), merA/B+	CC5	POS	POS	POS	NEG	NEG	NEG	NEG	NEG	NEG	NEG	NEG	NEG	NEG	NEG	NEG	NEG	POS	NEG	NEG	NEG	NEG	NEG	AMB	NEG	NEG	NEG	NEG	NEG	NEG
UKD-0731319	SCCmec IVc (IS-105), merA/B+	CC5	POS	POS	POS	NEG	NEG	NEG	NEG	NEG	NEG	NEG	NEG	NEG	NEG	NEG	NEG	NEG	POS	NEG	NEG	NEG	NEG	NEG	POS	NEG	NEG	NEG	NEG	NEG	NEG
UKD-0733137	SCCmec IVc (IS-105)	CC8	POS	POS	POS	NEG	NEG	NEG	NEG	NEG	NEG	NEG	NEG	NEG	NEG	NEG	NEG	NEG	POS	NEG	NEG	NEG	POS	NEG	POS	NEG	NEG	NEG	NEG	NEG	NEG
UKD-0305690	SCCmec IVc (IS-105)	CC22	POS	POS	POS	NEG	NEG	NEG	NEG	NEG	NEG	NEG	NEG	NEG	NEG	NEG	NEG	NEG	POS	NEG	NEG	NEG	NEG	NEG	NEG	NEG	NEG	NEG	NEG	NEG	NEG
UKD-1424706	SCCmec IVc (IS-105)	CC22	POS	POS	POS	NEG	NEG	POS	NEG	NEG	NEG	NEG	NEG	NEG	NEG	NEG	NEG	POS	POS	NEG	NEG	POS	NEG	NEG	NEG	NEG	NEG	NEG	NEG	NEG	NEG
Prediction for TCH60, GenBank CP002110.1	SCCmec IVc (TCH60)	CC30	POS	POS	POS	NEG	NEG	NEG	NEG	NEG	NEG	NEG	NEG	NEG	NEG	NEG	NEG	NEG	NEG	NEG	NEG	NEG	NEG	NEG	NEG	NEG	NEG	NEG	NEG	NEG	NEG
Reference strain JCSC4788	SCCmec IVc (TCH60)	CC8	NEG	NEG	NEG	NEG	NEG	NEG	NEG	NEG	NEG	NEG	NEG	NEG	NEG	NEG	NEG	POS	NEG	NEG	NEG	NEG	NEG	NEG	NEG	NEG	NEG	NEG	NEG	NEG	NEG
UKD-1318318	SCCmec IVc (TCH60)	CC5	POS	POS	POS	NEG	NEG	POS	NEG	NEG	NEG	NEG	NEG	NEG	NEG	NEG	NEG	NEG	NEG	NEG	NEG	NEG	NEG	NEG	NEG	NEG	NEG	NEG	NEG	NEG	NEG
UKD-14580050	SCCmec IVc (TCH60)	CC5	POS	POS	POS	NEG	NEG	POS	NEG	NEG	NEG	NEG	NEG	NEG	NEG	NEG	NEG	NEG	NEG	NEG	NEG	NEG	NEG	NEG	NEG	NEG	NEG	NEG	NEG	NEG	NEG
UKD-08F01314	SCCmec IVc (TCH60)	CC8	POS	POS	POS	NEG	NEG	NEG	NEG	NEG	NEG	NEG	NEG	NEG	NEG	NEG	NEG	POS	NEG	NEG	NEG	NEG	NEG	NEG	NEG	NEG	POS	NEG	NEG	POS	NEG
KHDN-50361518	SCCmec IVc (TCH60)	CC8	NEG	NEG	NEG	NEG	NEG	POS	NEG	NEG	NEG	NEG	NEG	NEG	NEG	NEG	NEG	NEG	NEG	NEG	NEG	NEG	NEG	NEG	NEG	NEG	POS	NEG	NEG	POS	NEG
UKD-0307912	SCCmec IVc (TCH60)	CC22	POS	POS	POS	NEG		POS	NEG	NEG	NEG	NEG	NEG	NEG	NEG	NEG	NEG	NEG	NEG	NEG	NEG	NEG	NEG	NEG	NEG	NEG	NEG	NEG	NEG	NEG	NEG
UKD-0220390	SCCmec IVc (TCH60)	CC45 [agr I]	POS	POS	POS	NEG	NEG	NEG	NEG	NEG	NEG	NEG	NEG	NEG	NEG	NEG	NEG	NEG	NEG	NEG	NEG	NEG	NEG	NEG	AMB	NEG	NEG	NEG	NEG	NEG	NEG
UKD-0311106	SCCmec IVc (TCH60)	CC45 [agr I]	POS	POS	POS	NEG	NEG	NEG	NEG	NEG	NEG	NEG	NEG	NEG	NEG	NEG	NEG	NEG	NEG	NEG	NEG	NEG	NEG	NEG	NEG	NEG	NEG	NEG	NEG	NEG	NEG
UKD-0719855	SCCmec IVc (TCH60)	CC45 [agr I]	POS	POS	POS	NEG		NEG	NEG	NEG	NEG	NEG	NEG	NEG	NEG	NEG	NEG	NEG	NEG	NEG	NEG	NEG	NEG	NEG	NEG	NEG	NEG	NEG	NEG	NEG	NEG
UKD-0422079	SCCmec IVc (TCH60)	CC45 [agr I]	POS	POS	POS	NEG	NEG	POS	NEG	NEG	NEG	NEG	NEG	NEG	NEG	NEG	NEG	NEG	NEG	NEG	NEG	NEG	NEG	NEG	NEG	NEG	NEG	NEG	NEG	NEG	NEG
UKD-0409235	SCCmec IVc (TCH60)	CC80	NEG	NEG	NEG	NEG	NEG	NEG	NEG	NEG	NEG	NEG	NEG	NEG	NEG	NEG	NEG	NEG	NEG	POS	POS	NEG	NEG	NEG	NEG	NEG	NEG	NEG	NEG	NEG	NEG
UKD-0409807	SCCmec IVc (TCH60)	CC80	POS	POS	POS	NEG	NEG	NEG	NEG	NEG	NEG	NEG	NEG	NEG	AMB	NEG	NEG	NEG	NEG	POS	POS	NEG	POS	NEG	POS	NEG	NEG	NEG	NEG	NEG	NEG
UKD-0416073	SCCmec IVc (TCH60)	CC80	POS	POS	POS	NEG	NEG	NEG	NEG	NEG	NEG	NEG	NEG	NEG	NEG	NEG	NEG	NEG	NEG	POS	POS	NEG	POS	NEG	POS	NEG	NEG	NEG	NEG	NEG	NEG
UKD-0431728	SCCmec IVc (TCH60)	CC80	POS	POS	POS	NEG	NEG	NEG	NEG	NEG	NEG	NEG	NEG	NEG	NEG	NEG	NEG	NEG	NEG	POS	POS	NEG	POS	NEG	NEG	NEG	NEG	NEG	NEG	NEG	NEG
UKD-1001508	SCCmec IVc (TCH60)	CC80	POS	POS	POS	NEG	NEG	NEG	NEG	NEG	NEG	NEG	NEG	NEG	NEG	NEG	NEG	NEG	NEG	POS	POS	NEG	POS	NEG	POS	NEG	NEG	NEG	NEG	NEG	NEG
UKD-1010412	SCCmec IVc (TCH60)	CC80	NEG	NEG	NEG	NEG	NEG	NEG	NEG	NEG	NEG	NEG	NEG	NEG	NEG	NEG	NEG	NEG	NEG	POS	POS	NEG	NEG	NEG	NEG	NEG	NEG	NEG	NEG	NEG	NEG
UKD-12753005	SCCmec IVc (TCH60)	CC80	POS	POS	POS	NEG	NEG	NEG	NEG	NEG	NEG	NEG	NEG	NEG	NEG	NEG	NEG	NEG	NEG	POS	POS	NEG	POS	NEG	POS	NEG	NEG	NEG	NEG	NEG	NEG
UKD-15574809	SCCmec IVc (TCH60)	CC80	POS	POS	POS	NEG	NEG	POS	NEG	NEG	NEG	NEG	NEG	NEG	NEG	NEG	NEG	NEG	NEG	POS	POS	NEG	POS	NEG	POS	NEG	NEG	NEG	NEG	NEG	NEG
KHDN-16120291	SCCmec IVc (TCH60)	CC88	POS	POS	POS	NEG	NEG	NEG	NEG	NEG	NEG	NEG	NEG	NEG	NEG	NEG	NEG	NEG	NEG	NEG	NEG	NEG	NEG	NEG	NEG	NEG	NEG	NEG	NEG	NEG	NEG
Prediction for WW2703/97, GenBank ACSW	SCCmec IVc (WW2703/97)	CC30	POS	POS	POS	NEG	NEG	NEG	NEG	NEG	NEG	NEG	NEG	NEG	NEG	NEG	NEG	NEG	NEG	NEG	NEG	NEG	NEG	NEG	NEG	NEG	NEG	NEG	NEG	NEG	NEG
UKD-14580088	SCCmec IVc (WW2703/97)	CC398	POS	POS	POS	POS	NEG	NEG	NEG	NEG	NEG	NEG	NEG	NEG	NEG	NEG	NEG	NEG	NEG	NEG	NEG	POS	NEG	NEG	NEG	POS	NEG	NEG	NEG	NEG	NEG
Prediction for SA_ST125_MupR, GenBank ASTH	SCCmec IVc+speG+ccrAB4 (SA_ST125)	CC5	POS	POS	POS	NEG	NEG	NEG	NEG	POS	NEG	POS	NEG	NEG	NEG	NEG	NEG	POS	POS	POS	POS	NEG	NEG	POS	NEG	NEG	NEG	NEG	NEG	NEG	NEG
UKD-0439909	SCCmec IVc+speG+ccrAB4 (SA_ST125)	CC5	POS	POS	POS	NEG	NEG	POS	NEG	POS	NEG	POS	NEG	NEG	NEG	NEG	NEG	NEG	POS	POS	POS	NEG	NEG	NEG	NEG	NEG	NEG	NEG	NEG	NEG	NEG
Prediction for MRSA177, GenBank AECP	SCCmec IVc+Cu/Hg (MRSA177)		POS	POS	POS	NEG	NEG	NEG	NEG	NEG	NEG	NEG	NEG	NEG	NEG	NEG	NEG	NEG	NEG	NEG	NEG	NEG	NEG	NEG	POS	NEG	NEG	NEG	NEG	NEG	NEG
UKD-1279734	SCCmec IVc+Cu/Hg (MRSA177)	CC8	POS	POS	POS	NEG	NEG	NEG	NEG	NEG	NEG	NEG	NEG	NEG	NEG	NEG	NEG	NEG	NEG	NEG	NEG	NEG	NEG	NEG	POS	NEG	NEG	NEG	NEG	NEG	NEG
UKD-14580899	SCCmec IVc+Cu/Hg (MRSA177)	CC8	POS	POS	POS	NEG	NEG	NEG	NEG	NEG	NEG	NEG	NEG	NEG	NEG	NEG	NEG	NEG	NEG	NEG	NEG	NEG	NEG	NEG	NEG	NEG	NEG	NEG	NEG	NEG	NEG
UKD-16002228	SCCmec IVc+Cu/Hg (MRSA177)	CC8	POS	POS	POS	NEG	NEG	NEG	NEG	NEG	NEG	NEG	NEG	NEG	AMB	NEG	NEG	NEG	NEG	NEG	NEG	NEG	NEG	NEG	NEG	NEG	NEG	NEG	NEG	NEG	NEG
Prediction for HO 5096 0412, GenBank HE681097.1	SCCmec IVh/j (HO50960412/JCSC6670)	CC22	POS	POS	POS	NEG	NEG	NEG	NEG	NEG	NEG	NEG	NEG	NEG	NEG	NEG	NEG	NEG	NEG	NEG	NEG	NEG	NEG	NEG	NEG	NEG	NEG	NEG	NEG	NEG	NEG
Prediction for JCSC6670, GenBank AB425824.1	SCCmec IVh/j (HO50960412/JCSC6670)																														
Reference strain Dublin_DSH_E1749	SCCmec IVh/j (HO50960412/JCSC6670)		POS	POS	POS	NEG	NEG	POS	NEG	NEG	NEG	NEG	NEG	NEG	NEG	NEG	NEG	NEG	NEG	NEG	NEG	NEG	NEG	NEG	NEG	NEG	NEG	NEG	NEG	NEG	NEG
UKD-14576248	SCCmec IVh/j (HO50960412/JCSC6670)	CC8	NEG	NEG	NEG	NEG	NEG	NEG	NEG	NEG	NEG	NEG	NEG	NEG	NEG	NEG	NEG	NEG	NEG	NEG	NEG	NEG	NEG	NEG	NEG	NEG	NEG	NEG	NEG	NEG	NEG
UKD-0120122	SCCmec IVh/j (HO50960412/JCSC6670)	CC22	POS	POS	POS	NEG	NEG	NEG	NEG	NEG	NEG	NEG	NEG	NEG	NEG	NEG	NEG	NEG	NEG	NEG	NEG	NEG	NEG	NEG	NEG	NEG	NEG	NEG	NEG	NEG	NEG
UKD-0205319	SCCmec IVh/j (HO50960412/JCSC6670)	CC22	POS	POS	POS	NEG	NEG	POS	NEG	NEG	NEG	NEG	NEG	NEG	NEG	NEG	NEG	NEG	NEG	NEG	NEG	NEG	NEG	NEG	NEG	NEG	NEG	NEG	NEG	NEG	NEG
UKD-0221259	SCCmec IVh/j (HO50960412/JCSC6670)	CC22	POS	POS	POS	NEG	NEG	POS	NEG	NEG	NEG	NEG	NEG	NEG	NEG	NEG	NEG	NEG	NEG	NEG	NEG	NEG	NEG	NEG	NEG	NEG	NEG	NEG	NEG	NEG	NEG
UKD-0418654	SCCmec IVh/j (HO50960412/JCSC6670)	CC22	POS	POS	POS	NEG	NEG	NEG	NEG	NEG	NEG	NEG	NEG	NEG	NEG	NEG	NEG	NEG	NEG	NEG	NEG	NEG	NEG	NEG	NEG	NEG	NEG	NEG	NEG	NEG	NEG
UKD-0429721	SCCmec IVh/j (HO50960412/JCSC6670)	CC22	POS	POS	NEG	NEG	NEG	POS	NEG	NEG	NEG	NEG	NEG	NEG	NEG	NEG	NEG	NEG	NEG	NEG	NEG	NEG	NEG	NEG	NEG	NEG	NEG	NEG	NEG	NEG	NEG
UKD-1116194	SCCmec IVh/j (HO50960412/JCSC6670)	CC22	POS	POS	POS	NEG	NEG	POS	NEG	NEG	NEG	NEG	NEG	NEG	NEG	NEG	NEG	NEG	NEG	NEG	AMB	NEG	NEG	NEG	NEG	NEG	NEG	NEG	NEG	NEG	NEG
UKD-1122367	SCCmec IVh/j (HO50960412/JCSC6670)	CC22	POS	POS	POS	NEG	NEG	NEG	NEG	NEG	NEG	NEG	NEG	NEG	AMB	NEG	NEG	NEG	NEG	NEG	NEG	NEG	NEG	NEG	NEG	NEG	NEG	NEG	NEG	NEG	NEG
UKD-14012840	SCCmec IVh/j (HO50960412/JCSC6670)	CC22	POS	POS	POS	NEG	NEG	POS	NEG	NEG	NEG	NEG	NEG	NEG	NEG	NEG	NEG	NEG	NEG	NEG	NEG	NEG	NEG	NEG	NEG	NEG	NEG	NEG	NEG	NEG	NEG
UKD-14579833	SCCmec IVh/j (HO50960412/JCSC6670)	CC22	POS	POS	POS	NEG	NEG	POS	NEG	NEG	NEG	NEG	NEG	NEG	NEG	NEG	NEG	NEG	NEG	NEG	NEG	NEG	NEG	NEG	NEG	NEG	NEG	NEG	NEG	NEG	NEG
UKD-15579198	SCCmec IVh/j (HO50960412/JCSC6670)	CC22	POS	POS	POS	NEG	NEG	NEG	NEG	NEG	NEG	NEG	NEG	NEG	AMB	NEG	NEG	NEG	NEG	NEG	NEG	NEG	NEG	NEG	NEG	NEG	NEG	NEG	NEG	NEG	NEG
UKD-4052193	SCCmec IVh/j (HO50960412/JCSC6670)	CC22	POS	POS	POS	NEG	NEG	POS	NEG	NEG	NEG	NEG	NEG	NEG	AMB	NEG	NEG	NEG	NEG	NEG	NEG	NEG	NEG	NEG	NEG	NEG	NEG	NEG	NEG	NEG	NEG
UKD-124968	SCCmec IVh/j (HO50960412/JCSC6670)	CC22	POS	POS	POS	NEG	NEG	POS	NEG	NEG	NEG	NEG	NEG	NEG	NEG	NEG	NEG	POS	NEG	NEG	NEG	NEG	NEG	POS	NEG	NEG	NEG	NEG	NEG	NEG	NEG
UKD-14572546	SCCmec IVh/j (HO50960412/JCSC6670)	CC22	POS	POS	POS	NEG	AMB	POS	NEG	NEG	NEG	NEG	NEG	NEG	NEG	NEG	NEG	NEG	NEG	NEG	NEG	NEG	NEG	NEG	NEG	NEG	NEG	NEG	NEG	NEG	NEG
UKD-14582287	SCCmec IVh/j (HO50960412/JCSC6670)	CC22	POS	POS	POS	NEG	NEG	NEG	NEG	NEG	NEG	NEG	NEG	NEG	POS	NEG	NEG	NEG	NEG	NEG	NEG	AMB	NEG	NEG	NEG	NEG	NEG	NEG	NEG	NEG	NEG
UKD-14583376	SCCmec IVh/j (HO50960412/JCSC6670)	CC22	POS	POS	POS	NEG	NEG	NEG	NEG	NEG	NEG	NEG	NEG	NEG	POS	NEG	NEG	NEG	NEG	NEG	NEG	AMB	NEG	NEG	NEG	NEG	NEG	NEG	NEG	NEG	NEG
UKD-14583496	SCCmec IVh/j (HO50960412/JCSC6670)	CC22	POS	POS	POS	NEG	AMB	POS	NEG	NEG	NEG	NEG	NEG	NEG	POS	NEG	NEG	NEG	NEG	NEG	NEG	AMB	NEG	NEG	NEG	NEG	NEG	NEG	NEG	NEG	NEG
UKD-0013324	SCCmec IVh/j (HO50960412/JCSC6670)	CC22	POS	POS	AMB	NEG	NEG	NEG	NEG	NEG	NEG	NEG	NEG	NEG	NEG	NEG	NEG	NEG	NEG	NEG	NEG	NEG	NEG	NEG	NEG	NEG	NEG	NEG	NEG	NEG	NEG
UKD-0125329	SCCmec IVh/j (HO50960412/JCSC6670)	CC22	POS	POS	AMB	NEG	NEG	NEG	NEG	NEG	NEG	NEG	NEG	NEG	NEG	NEG	NEG	NEG	NEG	NEG	NEG	NEG	NEG	NEG	NEG	NEG	NEG	NEG	NEG	NEG	NEG
UKD-0215498	SCCmec IVh/j (HO50960412/JCSC6670)	CC22	POS	POS	POS	NEG	NEG	NEG	NEG	NEG	NEG	NEG	NEG	NEG	NEG	NEG	NEG	NEG	NEG	NEG	NEG	NEG	NEG	NEG	NEG	NEG	NEG	NEG	NEG	NEG	NEG
Prediction for M08-0126, GenBank FR753166.1	SCCmec IVh/j+ACME2 (M08-0126)	CC22																													
UKD-1379483I	SCCmec IVh/j+ACME2 (M08-0126)	CC22	POS	POS	POS	NEG	NEG	POS	NEG	NEG	NEG	NEG	NEG	NEG	AMB	NEG	NEG	NEG	NEG	NEG	AMB	NEG	NEG	NEG	NEG	NEG	NEG	NEG	NEG	NEG	NEG
UKD-5150204	SCCmec IV+speG+Cu/czrC+ccrAB4	CC22	POS	POS	POS	NEG	NEG	NEG	NEG	POS	NEG	POS	NEG	NEG	NEG	NEG	NEG	NEG	POS	NEG	NEG	NEG	NEG	NEG	NEG	NEG	NEG	NEG	NEG	NEG	NEG
UKD-15578423	SCCmec IV+speG+czrC+ccrAB4 var. 1	CC22	POS	POS	POS	NEG	NEG	NEG	NEG	NEG	NEG	NEG	NEG	NEG	AMB	NEG	NEG	NEG	POS	NEG	NEG	NEG	NEG	NEG	NEG	NEG	NEG	NEG	NEG	NEG	NEG
UKD-16582870	SCCmec IV+speG+czrC+ccrAB4 var. 1	CC22	POS	POS	POS	NEG	NEG	NEG	NEG	NEG	NEG	NEG	NEG	NEG	NEG	NEG	NEG	NEG	POS	NEG	NEG	NEG	NEG	NEG	NEG	NEG	NEG	NEG	NEG	NEG	NEG
UKD-15571940	SCCmec IV+speG+czrC+ccrAB4 var. 2	CC22	POS	POS	POS	NEG	NEG	NEG	NEG	NEG	NEG	NEG	NEG	NEG	AMB	NEG	NEG	NEG	NEG	NEG	NEG	NEG	NEG	NEG	NEG	NEG	NEG	NEG	NEG	NEG	NEG

Isolate ID	SCCmec type	Clonal complex	RESISTANCE : PENICILLINASE			RESISTANCE : MLS-ANTIBIOTICS												RESISTANCE : AMINOGLYSOSIDES			RESISTANCE : MISCELLANEOUS GENES										
			beta-lactamase	beta lactamase repressor (inhibitor)	beta-lactamase regulatory protein	erythromycin/clindamycin resistance			Linco-samid-Nucleo-tidyltrans-ferase	energy-dependent efflux of erythro-mycin	macrolide efflux protein A	probable lysylphos-phatidyl-glycerol synthetase	virginia-mycin A acetyltransf erase	acetyl-transferase inactivating streptogra min A	ATP binding protein, streptogramin-A-resistance		virginia-mycin B hydrolase	bifunctional enzyme Aac/Aph, gentamicin resistance	amino-glycoside adenyl-transferase, tobramycin resistance	3'5'-aminog-lycoside phospho-transferase, neo-/kanamycin resistance	strepto-thricine-acetyl-transferase	dihydro-folate reductase type 1	fusidic acid resistence	mupirocin resistence protein	tetracycline-resistance		chloramphenicol acetyltransferase				
			blaZ	blaI	blaR	ermA	ermB	ermC	linA/ InuA	msrA	mefA	mph(C)	vat(A)	vat(B)	vga(A)	vga(A) (BM 3327)	vgb	aacA-aphD	aadD	aphA3	sat	dfrA	far1	mupA	tetK	tetM	cat	cat (pC221)	cat (pc223)	cat (pMC524)	cat (pSBK203 R)
Prediction for CN1, GenBank CP003979.1	SCCmec IVA (CN1)	CC8 (ST72)	POS	POS	POS	NEG	NEG	POS	NEG	NEG	NEG	NEG	NEG	NEG	NEG	NEG	NEG	NEG	POS	NEG	NEG	NEG	NEG	NEG	NEG	NEG	NEG	NEG	NEG	NEG	NEG
UKD-0900871	SCCmec IVA (CN1)	CC8 (ST72)	POS	POS	POS	NEG	NEG	NEG	NEG	NEG	NEG	NEG	NEG	NEG	NEG	NEG	NEG	NEG	NEG	NEG	NEG	NEG	NEG	NEG	NEG	NEG	NEG	NEG	NEG	NEG	NEG
Prediction for CMFT1723, GenBank HF569096.1	SCCmec V (Bengal Bay)																														
KHDN-10405134	SCCmec V (Bengal Bay)	CC1	POS	POS	POS	NEG	NEG	NEG	NEG	NEG	NEG	NEG	NEG	NEG	NEG	NEG	NEG	POS	NEG	NEG	NEG	NEG	NEG	NEG	POS	NEG	NEG	NEG	NEG	NEG	NEG
UKD-0932405	SCCmec V (Bengal Bay)	CC1 (ST573/772)	POS	POS	POS	NEG	NEG	POS	NEG	POS	NEG	POS	NEG	NEG	NEG	NEG	NEG	POS	NEG	POS	POS	NEG	NEG	NEG	NEG	NEG	NEG	NEG	NEG	NEG	NEG
UKD-1003510	SCCmec V (Bengal Bay)	CC1 (ST573/772)	POS	POS	POS	NEG	NEG	NEG	NEG	POS	NEG	POS	NEG	NEG	NEG	NEG	NEG	POS	NEG	POS	POS	NEG	NEG	NEG	NEG	NEG	NEG	NEG	NEG	NEG	NEG
UKD-1112420	SCCmec V (Bengal Bay)	CC1 (ST573/772)	POS	POS	POS	NEG	NEG	NEG	NEG	POS	NEG	POS	NEG	NEG	NEG	NEG	NEG	POS	NEG	POS	POS	NEG	NEG	NEG	NEG	NEG	NEG	NEG	NEG	NEG	NEG
UKD-1275832	SCCmec V (Bengal Bay)	CC1 (ST573/772)	POS	POS	POS	NEG	NEG	NEG	NEG	POS	NEG	POS	NEG	NEG	NEG	NEG	NEG	POS	NEG	POS	POS	NEG	NEG	NEG	NEG	NEG	NEG	NEG	NEG	NEG	NEG
UKD-13023811	SCCmec V (Bengal Bay)	CC1 (ST573/772)	POS	POS	POS	NEG	NEG	NEG	NEG	POS	NEG	POS	NEG	NEG	NEG	NEG	NEG	POS	NEG	POS	POS	NEG	NEG	NEG	NEG	NEG	NEG	NEG	NEG	NEG	NEG
UKD-14572163	SCCmec V	CC5	POS	POS	POS	NEG	NEG	POS	NEG	NEG	NEG	NEG	NEG	NEG	NEG	NEG	NEG	NEG	NEG	NEG	NEG	NEG	NEG	NEG	POS	POS	NEG	NEG	NEG	NEG	NEG
KHDN-10421133	SCCmec V+fus	CC97	POS	POS	POS	NEG	NEG	NEG	NEG	NEG	NEG	NEG	NEG	NEG	NEG	NEG	NEG	POS	NEG	NEG	NEG	NEG	NEG	NEG	NEG	NEG	NEG	NEG	NEG	NEG	NEG
KHDN-21343541	SCCmec V+fus+tir+ccrAB1	CC1	POS	POS	POS	NEG	NEG	NEG	NEG	NEG	NEG	NEG	NEG	NEG	NEG	NEG	NEG	POS	NEG	POS	POS	NEG	NEG	NEG	NEG	NEG	NEG	NEG	NEG	NEG	NEG
KHDN-10416893	SCCmec V+czrC+speG+ccrAB4	CC22	POS	POS	POS	NEG	NEG	NEG	NEG	NEG	NEG	NEG	NEG	NEG	NEG	NEG	NEG	NEG	NEG	NEG	NEG	NEG	NEG	NEG	NEG	NEG	NEG	NEG	NEG	NEG	NEG
Prediction for GR1, GenBank AJLX	SCCmec VT (GR1)	CC361	POS	POS	POS	NEG	NEG	NEG	NEG	NEG	NEG	NEG	NEG	NEG	NEG	NEG	NEG	POS	NEG	POS	POS	NEG	NEG	NEG	POS	NEG	NEG	NEG	NEG	NEG	NEG
UKD-0422872	SCCmec VT (GR1)	CC152	POS	POS	POS	NEG	NEG	NEG	NEG	NEG	NEG	NEG	NEG	NEG	AMB	NEG	NEG	POS	NEG	NEG	NEG	NEG	NEG	NEG	NEG	NEG	NEG	NEG	NEG	NEG	NEG
Prediction for PM1, GenBank BAFA	SCCmec VT (PM1)	CC59	POS	POS	POS	NEG	POS	NEG	NEG	NEG	NEG	NEG	NEG	NEG	NEG	NEG	NEG	NEG	NEG	POS	POS	NEG	NEG	NEG	POS	NEG	POS	NEG	POS	NEG	AMB
KHDN-000871	SCCmec VT (PM1)	CC45 (agr I)	NEG	NEG	NEG	NEG	NEG	NEG	NEG	NEG	NEG	NEG	NEG	NEG	NEG	NEG	NEG	NEG	NEG	NEG	NEG	NEG	NEG	NEG	NEG	NEG	NEG	NEG	NEG	NEG	NEG
UKD-0725052	SCCmec VT (PM1)	CC59	POS	POS	POS	NEG	POS	NEG	NEG	NEG	NEG	NEG	NEG	NEG	NEG	NEG	NEG	NEG	NEG	POS	POS	NEG	NEG	NEG	POS	NEG	NEG	NEG	NEG	NEG	NEG
UKD-1312138	SCCmec VT (PM1)	CC59	POS	POS	POS	NEG	POS	NEG	NEG	NEG	NEG	NEG	NEG	NEG	NEG	NEG	NEG	NEG	NEG	POS	POS	NEG	NEG	NEG	POS	NEG	POS	NEG	POS	NEG	POS
UKD-Z4728	SCCmec VT (PM1)	CC59	POS	POS	POS	NEG	POS	NEG	NEG	NEG	NEG	NEG	NEG	NEG	NEG	NEG	NEG	NEG	NEG	POS	POS	NEG	NEG	NEG	NEG	NEG	POS	NEG	NEG	NEG	POS
UKD-K0199	SCCmec VT unknown var.1	CC7	POS	POS	POS	NEG	NEG	NEG	NEG	NEG	NEG	NEG	NEG	NEG	NEG	NEG	NEG	NEG	NEG	NEG	NEG	NEG	NEG	NEG	NEG	NEG	NEG	NEG	NEG	NEG	NEG
UKD-15584619	SCCmec VT unknown var.2	CC30	POS	POS	POS	NEG	NEG	NEG	NEG	NEG	NEG	NEG	NEG	NEG	AMB	NEG	NEG	NEG	NEG	NEG	NEG	NEG	NEG	NEG	NEG	NEG	NEG	NEG	NEG	NEG	NEG
UKD-0712568	SCCmec VT unknown var.3	CC45 (agr I)	NEG	NEG	NEG	NEG	NEG	POS	NEG	NEG	NEG	NEG	NEG	NEG	NEG	NEG	NEG	NEG	NEG	NEG	NEG	NEG	NEG	NEG	NEG	NEG	NEG	NEG	NEG	NEG	NEG
KHDN-10420296	SCCmec VT+ACME2	CC45 (agr I)	POS	POS	POS	NEG	NEG	NEG	NEG	NEG	NEG	NEG	NEG	NEG	NEG	NEG	NEG	NEG	NEG	NEG	NEG	NEG	NEG	NEG	NEG	NEG	NEG	NEG	NEG	NEG	NEG
UKD-15577956	SCCmec VT+Cu/czrC+ccrA1	CC398	POS	POS	POS	NEG	NEG	NEG	NEG	NEG	NEG	NEG	NEG	NEG	NEG	NEG	NEG	POS	NEG	NEG	NEG	NEG	NEG	NEG	POS	POS	NEG	NEG	NEG	NEG	NEG
Prediction for S0385, GenBank AM990992	SCCmec VT+czrC (SO385)	CC398	POS	POS	POS	NEG	NEG	NEG	NEG	NEG	NEG	NEG	NEG	NEG	NEG	NEG	NEG	NEG	NEG	NEG	NEG	NEG	NEG	NEG	POS	POS	NEG	NEG	NEG	NEG	NEG
UKD-0628560	SCCmec VT+czrC (SO385)	CC8	NEG	NEG	NEG	NEG	NEG	POS	NEG	NEG	NEG	NEG	NEG	NEG	NEG	NEG	NEG	NEG	NEG	NEG	NEG	NEG	NEG	NEG	POS	NEG	NEG	NEG	NEG	NEG	NEG
UKD-0510018	SCCmec VT+czrC (SO385)	CC398	POS	POS	POS	NEG		NEG	NEG	NEG	NEG	NEG	NEG	NEG	NEG	NEG	NEG	NEG	NEG	NEG	NEG	NEG	NEG	NEG	AMB	POS	NEG	NEG	NEG	NEG	NEG
UKD-0903558	SCCmec VT+czrC (SO385)	CC398	POS	POS	POS	NEG	NEG	NEG	NEG	NEG	NEG	NEG	NEG	NEG	NEG	NEG	NEG	NEG	NEG	NEG	NEG	NEG	NEG	NEG	NEG	POS	NEG	NEG	NEG	NEG	NEG
UKD-0929815	SCCmec VT+czrC (SO385)	CC398	POS	POS	POS	POS	NEG	NEG	NEG	NEG	NEG	NEG	NEG	NEG	NEG	NEG	NEG	NEG	NEG	NEG	NEG	NEG	NEG	NEG	POS	POS	NEG	NEG	NEG	NEG	NEG
UKD-0907762	SCCmec VT+czrC (SO385)	CC398	POS	POS	POS	NEG	NEG	NEG	NEG	NEG	NEG	NEG	NEG	NEG	NEG	NEG	NEG	NEG	NEG	NEG	NEG	NEG	NEG	NEG	POS	POS	NEG	NEG	NEG	NEG	NEG
UKD-1025105	SCCmec VT+czrC (SO385)	CC398	POS	POS	AMB	NEG	NEG	NEG	NEG	NEG	NEG	NEG	NEG	NEG	NEG	POS	NEG	NEG	NEG	NEG	NEG	NEG	NEG	NEG	AMB	POS	NEG	NEG	NEG	NEG	NEG
UKD-11780898	SCCmec VT+czrC (SO385)	CC398	POS	POS	POS	POS	NEG	NEG	NEG	NEG	NEG	NEG	NEG	NEG	AMB	NEG	NEG	NEG	NEG	NEG	NEG	NEG	NEG	NEG	POS	POS	NEG	NEG	NEG	NEG	NEG
UKD-1377009	SCCmec VT+czrC (SO385)	CC398	POS	POS	POS	NEG	NEG	POS	NEG	NEG	NEG	NEG	NEG	NEG	NEG	NEG	NEG	POS	NEG	NEG	NEG	NEG	NEG	NEG	POS	POS	NEG	NEG	NEG	NEG	NEG
UKD-143406	SCCmec VT+czrC (SO385)	CC398	POS	POS	POS	NEG	AMB	POS	NEG	NEG	NEG	NEG	NEG	NEG	NEG	POS	NEG	NEG	NEG	NEG	NEG	NEG	NEG	NEG	POS	POS	NEG	NEG	NEG	NEG	NEG
UKD-1400363	SCCmec VT+czrC (SO385)	CC398	POS	POS	POS	POS	NEG	POS	NEG	NEG	NEG	NEG	NEG	NEG	NEG	NEG	NEG	NEG	NEG	NEG	NEG	NEG	NEG	NEG	POS	POS	NEG	NEG	NEG	NEG	NEG
UKD-14571852	SCCmec VT+czrC (SO385)	CC398	POS	POS	POS	NEG	NEG	POS	NEG	NEG	NEG	NEG	NEG	NEG	NEG	POS	NEG	NEG	NEG	NEG	NEG	NEG	NEG	NEG	POS	POS	NEG	NEG	NEG	NEG	NEG
UKD-14582574	SCCmec VT+czrC (SO385)	CC398	POS	POS	POS	POS	NEG	NEG	NEG	NEG	NEG	NEG	NEG	NEG	NEG	NEG	NEG	NEG	NEG	NEG	NEG	NEG	NEG	NEG	POS	POS	NEG	NEG	NEG	NEG	NEG
UKD-152108	SCCmec VT+czrC (SO385)	CC398	POS	POS	POS	NEG	NEG	NEG	NEG	NEG	NEG	NEG	NEG	NEG	NEG	NEG	NEG	POS	NEG	NEG	NEG	NEG	NEG	NEG	POS	POS	NEG	NEG	NEG	NEG	NEG
UKD-15573321	SCCmec VT+czrC (SO385)	CC398	POS	POS	POS	NEG	NEG	NEG	NEG	NEG	NEG	NEG	NEG	NEG	POS	POS	NEG	NEG	NEG	NEG	NEG	NEG	NEG	NEG	POS	POS	NEG	NEG	NEG	NEG	NEG
UKD-15584034	SCCmec VT+czrC , ydhK-neg. variant	CC398	POS	POS	POS	NEG	NEG	NEG	NEG	NEG	NEG	NEG	NEG	NEG	NEG	NEG	NEG	NEG	POS	NEG	NEG	NEG	NEG	NEG	POS	POS	NEG	NEG	NEG	NEG	NEG
UKD-0932581	SCCmecVT+fus+czrC	CC22	POS	POS	POS	NEG	NEG	NEG	NEG	NEG	NEG	NEG	NEG	NEG	NEG	NEG	NEG	NEG	NEG	NEG	NEG	NEG	NEG	NEG	POS	NEG	NEG	NEG	NEG	NEG	NEG
UKD-1121994	SCCmecVT+fus+czrC	CC22	POS	POS	POS	NEG	NEG	NEG	NEG	NEG	NEG	NEG	NEG	NEG	NEG	NEG	NEG	NEG	NEG	NEG	NEG	NEG	NEG	NEG	NEG	NEG	NEG	NEG	NEG	NEG	NEG
UKD-1272252	SCCmecVT+fus+czrC	CC22	POS	POS	POS	NEG	NEG	NEG	NEG	NEG	NEG	NEG	NEG	NEG	NEG	NEG	NEG	NEG	NEG	NEG	NEG	NEG	NEG	NEG	POS	NEG	NEG	NEG	NEG	NEG	NEG
Prediction for MRSA18	SCCmec VI+fus (MRSA18)																														
UKD-1103380	SCCmec VI+fus (MRSA18)	CC7	NEG	NEG	NEG	NEG	NEG	NEG	NEG	NEG	NEG	NEG	NEG	NEG	NEG	NEG	NEG	NEG	NEG	NEG	NEG	POS	NEG	NEG	NEG	NEG	NEG	NEG	NEG	NEG	NEG
Prediction for M10/0061, GenBank FR823292.1	SCCmec XI (LGA251/M10-61)	CC130																													
Reference strain Dublin-DSH_M10 0061	SCCmec XI (LGA251/M10-61)	CC130	NEG	NEG	NEG	NEG	NEG	NEG	NEG	NEG	NEG	NEG	NEG	NEG	NEG	NEG	NEG	NEG	NEG	NEG	NEG	NEG	NEG	NEG	NEG	NEG	NEG	NEG	NEG	NEG	NEG
Prediction for LGA251, GenBank FR821779.1	SCCmec XI (LGA251/M10-61)	ST425																													
UKD-14015835	SCCmec XI (LGA251/M10-61)	CC130	NEG	NEG	NEG	NEG	NEG	NEG	NEG	NEG	NEG	NEG	NEG	NEG	NEG	NEG	NEG	NEG	NEG	NEG	NEG	NEG	NEG	NEG	NEG	NEG	NEG	NEG	NEG	NEG	NEG
UKD-16573252	SCCmec XI (LGA251/M10-61)	CC130	NEG	NEG	NEG	NEG	NEG	NEG	NEG	NEG	NEG	NEG	NEG	NEG	NEG	NEG	NEG	NEG	NEG	NEG	NEG	NEG	NEG	NEG	NEG	NEG	NEG	NEG	NEG	NEG	NEG
UKD-0217002	PseudoSCCmec, class B mec complex+Hg	CC8	POS	POS	POS	NEG	NEG	NEG	NEG	NEG	NEG	NEG	NEG	NEG	NEG	NEG	NEG	POS	NEG	POS	POS	NEG	NEG	NEG	NEG	POS	NEG	NEG	NEG	NEG	NEG
UKD-0008510	PseudoSCCmec, class B mec complex+Hg	CC8	POS	POS	POS	POS		NEG	NEG	NEG	NEG	NEG	NEG	NEG	NEG	NEG	NEG	POS	NEG	POS	POS	NEG	NEG	NEG	NEG	POS	POS	NEG	NEG	POS	NEG
UKD-0110962	PseudoSCCmec, class B mec complex+Hg	CC8	POS	POS	POS	POS		NEG	NEG	NEG	NEG	NEG	NEG	NEG	NEG	NEG	NEG	POS	NEG	POS	POS	NEG	NEG	NEG	NEG	POS	POS	POS	NEG	POS	NEG
KHDN-10408593	PseudoSCCmec, class B mec complex+Cu/Hg	CC8	POS	POS	POS	NEG	NEG	NEG	NEG	NEG	NEG	NEG	NEG	NEG	NEG	NEG	NEG	NEG	NEG	NEG	NEG	NEG	NEG	NEG	NEG	NEG	NEG	NEG	NEG	NEG	NEG
UKD-0007201	PseudoSCCmec, class B mec complex	CC5	POS	POS	POS	POS	NEG	NEG	NEG	NEG	NEG	NEG	NEG	NEG	NEG	NEG	NEG	POS	NEG	POS	POS	NEG	NEG	NEG	NEG	NEG	NEG	NEG	NEG	NEG	NEG
UKD-0033204	PseudoSCCmec, class B mec complex	CC5	POS	POS	POS	POS	NEG	NEG	NEG	NEG	NEG	NEG	NEG	NEG	NEG	NEG	NEG	POS	NEG	POS	POS	NEG	NEG	NEG	NEG	NEG	NEG	NEG	NEG	NEG	NEG
UKD-0134329	PseudoSCCmec, class B mec complex	CC5	POS	POS	POS	NEG	NEG	NEG	NEG	NEG	NEG	NEG	NEG	NEG	NEG	NEG	NEG	NEG	NEG	NEG	AMB	NEG	NEG	AMB	NEG	NEG	NEG	NEG	NEG	NEG	NEG
UKD-0806946	PseudoSCCmec class C+As/Cu	CC398	POS	POS	POS	POS	NEG	NEG	NEG	NEG	NEG	NEG	NEG	NEG	NEG	NEG	NEG	NEG	NEG	NEG	NEG	NEG	NEG	NEG	NEG	POS	NEG	NEG	NEG	NEG	NEG
UKD-0500610	ACME2	CC8	POS	POS	POS	NEG		POS	NEG	NEG	NEG	NEG	NEG	NEG	NEG	NEG	NEG	NEG	POS	NEG	NEG	NEG	NEG	POS	NEG	NEG	NEG	NEG	NEG	NEG	NEG
UKD-0610438	ACME2	CC8	POS	POS	POS	NEG	NEG	POS	NEG	NEG	NEG	NEG	NEG	NEG	NEG	NEG	NEG	POS	POS	NEG	NEG	NEG	NEG	POS	NEG	NEG	NEG	NEG	NEG	NEG	NEG
KHDN-10409344	ACME2	CC8	NEG	NEG	NEG	NEG	NEG	POS	NEG	NEG	NEG	NEG	NEG	NEG	AMB	NEG	NEG	NEG	NEG	NEG	NEG	NEG	NEG	NEG	NEG	NEG	NEG	NEG	NEG	NEG	NEG
UKD-0322559	ACME2+speG+czrC+ccrAB4	CC8	POS	POS	POS	NEG	NEG	POS	NEG	NEG	NEG	NEG	NEG	NEG	NEG	NEG	NEG	NEG	POS	NEG	NEG	NEG	NEG	NEG	NEG	NEG	NEG	NEG	NEG	NEG	NEG
UKD-0501998	ACME2+speG+czrC+ccrAB4	CC8	POS	POS	POS	NEG		POS	NEG	NEG	NEG	NEG	NEG	NEG	NEG	NEG	NEG	NEG	POS	NEG	NEG	NEG	NEG	NEG	NEG	NEG	NEG	NEG	NEG	NEG	NEG
UKD-0505213	ACME2+speG+czrC+ccrAB4	CC8	POS	POS	POS	NEG		POS	NEG	NEG	NEG	NEG	NEG	NEG	NEG	NEG	NEG	NEG	POS	NEG	NEG	NEG	NEG	NEG	NEG	NEG	NEG	NEG	NEG	NEG	NEG
Prediction for MSSA476, GenBank BX571857.1	fus+tir+ccrAB1 (MSSA476)	CC1	POS	POS	POS	NEG	NEG	NEG	NEG	NEG	NEG	NEG	NEG	NEG	NEG	NEG	NEG	NEG	NEG	NEG	NEG	NEG	NEG	NEG	NEG	NEG	NEG	NEG	NEG	NEG	NEG
Reference strain Sanger 476	fus+tir+ccrAB1 (MSSA476)	CC1	POS	POS	POS	NEG	NEG	NEG	NEG	NEG	NEG	NEG	NEG	NEG	NEG	NEG	NEG	NEG	NEG	NEG	NEG	NEG	NEG	NEG	AMB	NEG	NEG	NEG	NEG	NEG	NEG
UKD-14023914	fus+tir+ccrAB1 (MSSA476)	CC1	NEG	NEG	NEG	NEG	NEG	NEG	NEG	NEG	NEG	NEG	NEG	NEG	NEG	NEG	NEG	NEG	NEG	NEG	NEG	NEG	NEG	NEG	NEG	NEG	NEG	NEG	NEG	NEG	NEG
UKD-K00034	kdp+ccrAB2	CC182	POS	POS	POS	NEG	NEG	NEG	NEG	NEG	NEG	NEG	NEG	NEG	NEG	NEG	NEG	NEG	NEG	NEG	NEG	NEG	NEG	NEG	NEG	NEG	NEG	NEG	NEG	NEG	NEG
UKD-15580369	As+ccrAB4	CC22	POS	POS	POS	NEG	NEG	AMB	NEG	NEG	NEG	NEG	NEG	NEG	NEG	NEG	NEG	NEG	POS	NEG	NEG	NEG	NEG	NEG	NEG	NEG	NEG	NEG	NEG	NEG	NEG
UKD-15570100	ACME3+ccrAB1 (Strain 21342)	CC80	POS	POS	POS	NEG	NEG	NEG	NEG	NEG	NEG	NEG	NEG	NEG	AMB	NEG	NEG	NEG	NEG	NEG	NEG	NEG	NEG	NEG	NEG	NEG	NEG	NEG	NEG	NEG	NEG
UKD-1010678	speG+czrC+ccrAB4	CC8	NEG	NEG	NEG	NEG	NEG	NEG	NEG	NEG	NEG	NEG	NEG	NEG	NEG	NEG	NEG	NEG	NEG	NEG	NEG	NEG	NEG	NEG	NEG	NEG	NEG	NEG	NEG	NEG	NEG
UKD-1008243	speG+czrC+ccrAB4	CC8	POS	POS	POS	NEG	NEG	NEG	NEG	NEG	NEG	NEG	NEG	NEG	NEG	NEG	NEG	NEG	NEG	NEG	NEG	NEG	NEG	NEG	NEG	NEG	NEG	NEG	NEG	NEG	NEG
UKD-1009430	speG+czrC+ccrAB4	CC8	POS	POS	POS	NEG	NEG	NEG	NEG	NEG	NEG	NEG	NEG	NEG	NEG	NEG	NEG	NEG	NEG	NEG	NEG	NEG	NEG	NEG	NEG	NEG	NEG	NEG	NEG	NEG	NEG
UKD-12071828	speG+czrC+ccrAB4	CC22	POS	POS	POS	NEG	NEG	NEG	NEG	NEG	NEG	NEG	NEG	NEG	NEG	NEG	NEG	NEG	NEG	NEG	NEG	NEG	NEG	NEG	NEG	NEG	NEG	NEG	NEG	NEG	NEG
UKD-14011883	speG+czrC+ccrAB4	CC22	POS	POS	POS	NEG	NEG	NEG	NEG	NEG	NEG	NEG	NEG	NEG	NEG	NEG	NEG	NEG	POS	NEG	NEG	NEG	NEG	NEG	NEG	NEG	NEG	NEG	NEG	NEG	NEG
UKD-15008293	speG+czrC+ccrAB4	CC22	POS	POS	POS	NEG	NEG	POS	NEG	NEG	NEG	NEG	NEG	NEG	NEG	NEG	NEG	NEG	POS	NEG	NEG	NEG	NEG	NEG	NEG	NEG	NEG	NEG	NEG	NEG	NEG
UKD-1514877	speG+czrC+ccrAB4	CC22	POS	POS	POS	NEG	NEG	NEG	NEG	NEG	NEG	NEG	NEG	NEG	NEG	NEG	NEG	POS	POS	NEG	NEG	POS	NEG	NEG	NEG	NEG	NEG	NEG	NEG	NEG	NEG
UKD-15580971	speG+czrC+ccrAB4	CC22	POS	POS	POS	NEG	NEG	NEG	NEG	NEG	NEG	NEG	NEG	NEG	NEG	NEG	NEG	NEG	POS	NEG	NEG	NEG	NEG	NEG	NEG	NEG	NEG	NEG	NEG	NEG	NEG
UKD-15582646	speG+czrC+ccrAB4	CC22	POS	POS	POS	NEG	NEG	NEG	NEG	NEG	NEG	NEG	NEG	NEG	NEG	NEG	NEG	NEG	POS	NEG	NEG	NEG	NEG	NEG	NEG	NEG	NEG	NEG	NEG	NEG	NEG

Isolate ID	SCCmec type	Clonal complex	RESISTANCE : MISCELLANEOUS GENES													
			23S rRNA methyltransferase	chloramphenicol/ florfenicol exporter	metallothiol transferase		quaternary ammonium compound resistance protein A	quaternary ammonium compound resistance protein C						vancomycin resistance genes		
			cfr	fexA	fosB	fosB (plasmid)	qacA	qacC	qacC (cons)	qacC (equine)	qacC (SA5)	qacC (Ssap)	qacC (ST94)	vanA	vanB	vanZ
VIRTUAL HYBRIDISATION for COL, GenBank CP000046	*SCCmec I (COL)*	*CC8*	*NEG*	*NEG*	*POS*	*NEG*	*NEG*	*NEG*	*NEG*	*NEG*	*NEG*	*NEG*	*NEG*	*NEG*	*NEG*	*NEG*
**Reference strain COL**	**SCCmec I (COL)**	**CC8**	**NEG**	**NEG**	**POS**	**NEG**	**NEG**	**NEG**	**NEG**	**NEG**	**NEG**	**NEG**	**NEG**	**NEG**	**NEG**	**NEG**
**UKD-0007063**	SCCmec I (COL)	CC5	NEG	NEG	POS	AMB	POS	NEG	NEG	NEG	NEG	NEG	NEG	NEG	NEG	NEG
**UKD-0220759**	SCCmec I (COL)	CC5	NEG	NEG	POS	NEG	POS	NEG	NEG	NEG	NEG	NEG	NEG	NEG	NEG	NEG
**UKD-0308986**	SCCmec I (COL)	CC5	NEG	NEG	POS	NEG	NEG	NEG	NEG	NEG	NEG	NEG	NEG	NEG	NEG	NEG
**UKD-0500009**	SCCmec I (COL)	CC5	NEG	NEG	POS	NEG	POS	NEG	NEG	NEG	NEG	NEG	NEG	NEG	NEG	NEG
**UKD-0508483**	SCCmec I (COL)	CC5	NEG	NEG	POS	NEG	POS	NEG	NEG	NEG	NEG	NEG	NEG	NEG	NEG	NEG
**UKD-0519001**	SCCmec I (COL)	CC5	NEG	NEG	POS	NEG	POS	NEG	NEG	NEG	NEG	NEG	NEG	NEG	NEG	NEG
**UKD-0701876**	SCCmec I (COL)	CC5	NEG	NEG	POS	NEG	POS	NEG	NEG	NEG	NEG	NEG	NEG	NEG	NEG	NEG
**UKD-0708865**	SCCmec I (COL)	CC5	NEG	NEG	POS	NEG	POS	NEG	NEG	NEG	NEG	NEG	NEG	NEG	NEG	NEG
**UKD-1001782**	SCCmec I (COL)	CC5	NEG	NEG	POS	NEG	POS	NEG	NEG	NEG	NEG	NEG	NEG	NEG	NEG	NEG
Prediction for PSP1996, GenBank ANHU	*SCCmec I (PSP1996)*	*CC8*	*NEG*	*NEG*	*POS*	*NEG*	*POS*	*NEG*	*NEG*	*NEG*	*NEG*	*NEG*	*NEG*	*NEG*	*NEG*	*NEG*
**UKD-0261727**	SCCmec I (PSP1996)	CC8	NEG	NEG	POS	NEG	NEG	NEG	NEG	NEG	NEG	NEG	NEG	NEG	NEG	NEG
VIRTUAL HYBRIDISATION for MRSA7 : VIRTUAL HYBRI	*SCCmec I+fus+tirS (Geraldine Clone)*	*CC5*	*NEG*	*NEG*	*POS*	*NEG*	*NEG*	*NEG*	*NEG*	*NEG*	*NEG*	*NEG*	*NEG*	*NEG*	*NEG*	*NEG*
**UKD-1221930**	SCCmec I+fus+tirS (Geraldine Clone)	CC5	NEG	NEG	POS	NEG	NEG	NEG	NEG	NEG	NEG	NEG	NEG	NEG	NEG	NEG
Prediction for N315, GenBank BA000018.3	*SCCmec II (N315)*	*CC5*	*NEG*	*NEG*	*POS*	*NEG*	*NEG*	*NEG*	*NEG*	*NEG*	*NEG*	*NEG*	*NEG*	*NEG*	*NEG*	*NEG*
**Reference strain N315**	**SCCmec II (N315)**	**CC5**	**NEG**	**NEG**	**POS**	**NEG**	**NEG**	**NEG**	**NEG**	**NEG**	**NEG**	**NEG**	**NEG**	**NEG**	**NEG**	**NEG**
Prediction for MRSA252, GenBank BX571856.1	*SCCmec II (N315)*	*CC30*	*NEG*	*NEG*	*POS*	*NEG*	*NEG*	*NEG*	*NEG*	*NEG*	*NEG*	*NEG*	*NEG*	*NEG*	*NEG*	*NEG*
**Reference strain Sanger 252**	**SCCmec II (N315)**	**CC30**	**NEG**	**NEG**	**POS**	**NEG**	**NEG**	**NEG**	**NEG**	**NEG**	**NEG**	**NEG**	**NEG**	**NEG**	**NEG**	**NEG**
**UKD-0230792**	SCCmec II (N315)	CC30	NEG	NEG	POS	NEG	NEG	NEG	NEG	NEG	NEG	NEG	NEG	NEG	NEG	NEG
Prediction for Mu50, GenBank BA000017.4	*SCCmec II (N315)*	*CC5*	*NEG*	*NEG*	*POS*	*NEG*	*POS*	*NEG*	*NEG*	*NEG*	*NEG*	*NEG*	*NEG*	*NEG*	*NEG*	*NEG*
**Reference strain MU50**	**SCCmec II (N315)**	**CC5**	**NEG**	**NEG**	**POS**	**NEG**	**POS**	**NEG**	**NEG**	**NEG**	**NEG**	**NEG**	**NEG**	**NEG**	**NEG**	**NEG**
Prediction for JH1, GenBank CP000736.1	*SCCmec II (JH1/JH9)*	*CC5*	*NEG*	*NEG*	*POS*	*NEG*	*NEG*	*NEG*	*NEG*	*NEG*	*NEG*	*NEG*	*NEG*	*NEG*	*NEG*	*NEG*
Prediction for JH9, GenBank CP000703.1	*SCCmec II (JH1/JH9)*	*CC5*	*NEG*	*NEG*	*POS*	*NEG*	*NEG*	*NEG*	*NEG*	*NEG*	*NEG*	*NEG*	*NEG*	*NEG*	*NEG*	*NEG*
**Reference strain Dublin-DSH_AR07.4_0237**	**SCCmec II (JH1/JH9)**		**NEG**	**NEG**	**POS**	**NEG**	**NEG**	**NEG**	**NEG**	**NEG**	**NEG**	**NEG**	**NEG**	**NEG**	**NEG**	**NEG**
**Reference strain NARSA_382 / USA100**	**SCCmec II (JH1/JH9)**	**CC5**	**NEG**	**NEG**	**POS**	**NEG**	**NEG**	**NEG**	**NEG**	**NEG**	**NEG**	**NEG**	**NEG**	**NEG**	**NEG**	**NEG**
**UKD-0046921**	SCCmec II (JH1/JH9)	CC5	NEG	NEG	POS	NEG	NEG	NEG	NEG	NEG	NEG	NEG	NEG	NEG	NEG	NEG
**UKD-0333415**	SCCmec II (JH1/JH9)	CC5	NEG	NEG	POS	NEG	NEG	NEG	NEG	NEG	NEG	NEG	NEG	NEG	NEG	NEG
**UKD-0341645**	SCCmec II (JH1/JH9)	CC5	NEG	NEG	POS	NEG	NEG	NEG	NEG	NEG	NEG	NEG	NEG	NEG	NEG	NEG
**UKD-0504626**	SCCmec II (JH1/JH9)	CC5	NEG	NEG	POS	NEG	NEG	NEG	NEG	NEG	NEG	NEG	NEG	NEG	NEG	NEG
**UKD-0507581**	SCCmec II (JH1/JH9)	CC5	NEG	NEG	POS	NEG	NEG	NEG	NEG	NEG	NEG	NEG	NEG	NEG	NEG	NEG
**UKD-0507903**	SCCmec II (JH1/JH9)	CC5	NEG	NEG	POS	NEG	NEG	NEG	NEG	NEG	NEG	NEG	NEG	NEG	NEG	NEG
**UKD-0700817**	SCCmec II (JH1/JH9)	CC5	NEG	NEG	POS	NEG	NEG	NEG	NEG	NEG	NEG	NEG	NEG	NEG	NEG	NEG
**UKD-1014561**	SCCmec II (JH1/JH9)	CC5	NEG	NEG	POS	NEG	NEG	NEG	NEG	NEG	NEG	NEG	NEG	NEG	NEG	NEG
**UKD-1037507**	SCCmec II (JH1/JH9)	CC5	NEG	NEG	POS	NEG	NEG	NEG	NEG	NEG	NEG	NEG	NEG	NEG	NEG	NEG
**UKD-1038321**	SCCmec II (JH1/JH9)	CC5	NEG	NEG	POS	NEG	NEG	NEG	NEG	NEG	NEG	NEG	NEG	NEG	NEG	NEG
**UKD-1111608**	SCCmec II (JH1/JH9)	CC5	NEG	NEG	POS	NEG	NEG	NEG	NEG	NEG	NEG	NEG	NEG	NEG	NEG	NEG
**UKD-12753135**	SCCmec II (JH1/JH9)	CC5	NEG	NEG	POS	NEG	NEG	NEG	NEG	NEG	NEG	NEG	NEG	NEG	NEG	NEG
**UKD-1312816**	SCCmec II (JH1/JH9)	CC5	NEG	NEG	POS	NEG	NEG	NEG	NEG	NEG	NEG	NEG	NEG	NEG	NEG	NEG
**UKD-1401623**	SCCmec II (JH1/JH9)	CC5	NEG	NEG	POS	AMB	NEG	NEG	NEG	NEG	NEG	NEG	NEG	NEG	NEG	NEG
**UKD-15571943**	SCCmec II+czrC+ccrA/B-4	CC5	NEG	NEG	POS	NEG	NEG	NEG	NEG	NEG	NEG	NEG	AMB	NEG	NEG	NEG
**UKD-15576888**	SCCmec II+czrC+ccrA/B-4	CC5	NEG	NEG	POS	NEG	NEG	NEG	NEG	NEG	NEG	NEG	AMB	NEG	NEG	NEG
**UKD-14576461**	SCCmec II+speG+czrC+ccrA/B-4	CC5	NEG	NEG	POS	NEG	NEG	NEG	NEG	NEG	NEG	NEG	NEG	NEG	NEG	NEG
**UKD-15581649**	SCCmec II+speG+czrC+ccrA/B-4	CC5	NEG	NEG	POS	NEG	NEG	NEG	NEG	NEG	NEG	NEG	NEG	NEG	NEG	NEG
Prediction for CIGC345D, GenBank AHVO	*SCCmec II A/B/D*	*CC8*	*NEG*	*NEG*	*POS*	*NEG*	*NEG*	*NEG*	*NEG*	*NEG*	*NEG*	*NEG*	*NEG*	*NEG*	*NEG*	*NEG*
**UKD-12K00171**	SCCmec II A/B/D	CC8	NEG	NEG	POS	NEG	NEG	NEG	NEG	NEG	NEG	NEG	NEG	NEG	NEG	NEG
Prediction for JKD6008, GenBank CP002120.1	*SCC [mec III+Cd] (JKD6008)*	*CC8 (ST239)*	*NEG*	*NEG*	*POS*	*NEG*	*POS*	*NEG*	*NEG*	*NEG*	*NEG*	*NEG*	*NEG*	*NEG*	*NEG*	*NEG*
Prediction for JKD6009, GenBank ABSA	*SCC [mec III+Cd] (JKD6008)*	*CC8 (ST239)*	*NEG*	*NEG*	*POS*	*NEG*	*POS*	*NEG*	*NEG*	*NEG*	*NEG*	*NEG*	*NEG*	*NEG*	*NEG*	*NEG*
**Reference strain ATCC_33592**	**SCC [mec III+Cd] (ATCC33592)**	**CC8 (ST239)**	**NEG**	**NEG**	**POS**	**AMB**	**NEG**	**POS**	**POS**	**NEG**	**NEG**	**NEG**	**NEG**	**NEG**	**NEG**	**NEG**
Prediction for PPUKM-775-2009, GenBank AMRE	*SCC [mec III+Cd+ccrC] (PPUKM-775-2009)*	*CC8 (ST239)*	*NEG*	*NEG*	*POS*	*NEG*	*POS*	*NEG*	*NEG*	*NEG*	*NEG*	*NEG*	*NEG*	*NEG*	*NEG*	*NEG*
Prediction for T0131, GenBank CP002643.1	*SCC [mec III+Cd+ccrC] (T0131)*	*CC8 (ST239)*	*NEG*	*NEG*	*POS*	*NEG*	*NEG*	*NEG*	*NEG*	*NEG*	*NEG*	*NEG*	*NEG*	*NEG*	*NEG*	*NEG*
Prediction for XN108, GenBank CP007447.1	*SCC [mec III+Cd+ccrC] (XN108)*	*CC8 (ST239)*	*NEG*	*NEG*	*POS*	*NEG*	*NEG*	*NEG*	*NEG*	*NEG*	*NEG*	*NEG*	*NEG*	*NEG*	*NEG*	*NEG*
Prediction for CN79, GenBank ANCJ	*SCC [mec III+Cd+ccrC] (CN79)*	*CC8 (ST239)*	*NEG*	*NEG*	*POS*	*NEG*	*NEG*	*NEG*	*NEG*	*NEG*	*NEG*	*NEG*	*NEG*	*NEG*	*NEG*	*NEG*
Prediction for 16K, GenBank BABZ	*SCC [mec III+Cd+ccrC] (CN79)*	*CC8 (ST239)*	*NEG*	*NEG*	*POS*	*NEG*	*NEG*	*POS*	*POS*	*NEG*	*NEG*	*NEG*	*NEG*	*NEG*	*NEG*	*NEG*
**UKD-0835987**	SCC [mec III+Cd+ccrC] (CN79)	CC8 (ST239)	NEG	NEG	POS	NEG	NEG	POS	POS	NEG	NEG	NEG	NEG	NEG	NEG	NEG
**UKD-16581769**	SCCmec III+Cd+ccrC	CC8 (ST239)	NEG	NEG	POS	NEG	NEG	NEG	NEG	NEG	NEG	NEG	NEG	NEG	NEG	NEG
Prediction for Bmb9393, GenBank CP005288.1	*SCCmec III+Cd/Hg+ccrC (Bmb9393)*	*CC8 (ST239)*	*NEG*	*NEG*	*POS*	*NEG*	*NEG*	*NEG*	*NEG*	*NEG*	*NEG*	*NEG*	*NEG*	*NEG*	*NEG*	*NEG*
**UKD-15574263**	SCCmec III+Cd/Hg+ccrC (Bmb9393)	CC8 (ST239)	NEG	NEG	POS	NEG	NEG	NEG	NEG	NEG	NEG	NEG	NEG	NEG	NEG	NEG
Prediction for SK1585, GenBank AYLT	*SCCmec III+ccrC+Cd/Hg (SK1585)*	*CC8 (ST239)*	*NEG*	*NEG*	*NEG*	*NEG*	*NEG*	*POS*	*POS*	*NEG*	*NEG*	*NEG*	*NEG*	*NEG*	*NEG*	*NEG*
**UKD-105861**	SCCmec III+ccrC+Cd/Hg (SK1585)	CC8 (ST239)	NEG	NEG	POS	NEG	NEG	NEG	NEG	NEG	NEG	NEG	NEG	NEG	NEG	NEG
**UKD-134572**	SCCmec III+ccrC+Cd/Hg (SK1585)	CC8 (ST239)	NEG	NEG	POS	NEG	NEG	NEG	NEG	NEG	NEG	NEG	NEG	NEG	NEG	NEG
**UKD-139143**	SCCmec III+ccrC+Cd/Hg (SK1585)	CC8 (ST239)	NEG	NEG	POS	NEG	NEG	NEG	NEG	NEG	NEG	NEG	NEG	NEG	NEG	NEG
Prediction for TW20, GenBank FN433596.1	*SCC [mec III+Cd/Hg+ccrC] (TW20)*	*CC8 (ST239)*	*NEG*	*NEG*	*POS*	*NEG*	*POS*	*NEG*	*NEG*	*NEG*	*NEG*	*NEG*	*NEG*	*NEG*	*NEG*	*NEG*
Prediction for ATCC BAA-39, GenBank AEEK	*SCC [mec III+Cd/Hg+ccrC] (TW20)*	*CC8 (ST239)*	*NEG*	*NEG*	*POS*	*NEG*	*NEG*	*NEG*	*NEG*	*NEG*	*NEG*	*NEG*	*NEG*	*NEG*	*NEG*	*NEG*

Isolate ID	SCCmec type	Clonal complex	RESISTANCE : MISCELLANEOUS GENES													
			23S rRNA methyltransferase	chloramphenicol/ florfenicol exporter	metallothiol transferase		quaternary ammonium compound resistance protein A	quaternary ammonium compound resistance protein C						vancomycin resistance genes		
			cfr	fexA	fosB	fosB (plasmid)	qacA	qacC	qacC (cons)	qacC (equine)	qacC (SA5)	qacC (Ssap)	qacC (ST94)	vanA	vanB	vanZ
Prediction for MW2, GenBank BA000033.2	*SCCmec IVa (MW2)*	*CC1*	NEG	NEG	NEG	NEG	NEG	NEG	NEG	NEG	NEG	NEG	NEG	NEG	NEG	NEG
Reference strain MW2	SCCmec IVa (MW2)	CC1	NEG	NEG	NEG	NEG	NEG	NEG	NEG	NEG	NEG	NEG	NEG	NEG	NEG	NEG
Reference strain CA05 (JCSC1968)	SCCmec IVa (MW2)	CC45	NEG	NEG	NEG	NEG	NEG	NEG	NEG	NEG	NEG	NEG	NEG	NEG	NEG	NEG
UKD-0127106	SCCmec IVa (MW2)	CC1	NEG	NEG	NEG	NEG	NEG	NEG	NEG	NEG	NEG	NEG	NEG	NEG	NEG	NEG
UKD-0701446	SCCmec IVa (MW2)	CC1	NEG	NEG	NEG	NEG	NEG	NEG	NEG	NEG	NEG	NEG	NEG	NEG	NEG	NEG
UKD-0947486	SCCmec IVa (MW2)	CC1	NEG	NEG	NEG	NEG	NEG	NEG	NEG	NEG	NEG	NEG	NEG	NEG	NEG	NEG
UKD-1031910	SCCmec IVa (MW2)	CC1	NEG	NEG	NEG	NEG	NEG	NEG	NEG	NEG	NEG	NEG	NEG	NEG	NEG	NEG
UKD-14571328	SCCmec IVa (MW2)	CC1	NEG	NEG	NEG	NEG	POS	NEG	NEG	NEG	NEG	NEG	NEG	NEG	NEG	NEG
UKD-03003012	SCCmec IVa (MW2)	CC5	NEG	NEG	POS	NEG	NEG	NEG	NEG	NEG	NEG	NEG	NEG	NEG	NEG	NEG
UKD-15582582	SCCmec IVa (MW2)	CC6	NEG	NEG	POS	NEG	NEG	NEG	NEG	NEG	NEG	NEG	NEG	NEG	NEG	NEG
UKD-16574230	SCCmec IVa (MW2)	CC6	NEG	NEG	POS	NEG	NEG	NEG	NEG	NEG	NEG	NEG	NEG	NEG	NEG	NEG
UKD-16582560	SCCmec IVa (MW2)	CC6	NEG	NEG	POS	NEG	NEG	NEG	NEG	NEG	NEG	NEG	NEG	NEG	NEG	NEG
UKD-0802855	SCCmec IVa (MW2)	CC7	NEG	NEG	NEG	NEG	NEG	NEG	NEG	NEG	NEG	NEG	NEG	NEG	NEG	NEG
KHDN-10405258	SCCmec IVa (MW2)	CC8	NEG	NEG	POS	NEG	NEG	NEG	NEG	NEG	NEG	NEG	NEG	NEG	NEG	NEG
UKD-15573497	SCCmec IVa (MW2)	CC22	NEG	NEG	NEG	NEG	NEG	NEG	NEG	NEG	NEG	NEG	NEG	NEG	NEG	NEG
UKD-0634729	SCCmec IVa (MW2)	CC30	NEG	NEG	POS	NEG	NEG	NEG	NEG	NEG	NEG	NEG	NEG	NEG	NEG	NEG
UKD-1119771	SCCmec IVa (MW2)	CC30	NEG	NEG	POS	NEG	NEG	NEG	NEG	NEG	NEG	NEG	NEG	NEG	NEG	NEG
KHDN-10411458	SCCmec IVa (MW2)	CC30	NEG	NEG	POS	NEG	NEG	NEG	NEG	NEG	NEG	NEG	NEG	NEG	NEG	NEG
KHDN-398609	SCCmec IVa (MW2)	CC30	NEG	NEG	POS	NEG	NEG	POS	POS	NEG	NEG	AMB	NEG	NEG	NEG	NEG
KHDN-01081998	SCCmec IVa (MW2)	CC30	NEG	NEG	POS	AMB	NEG	NEG	NEG	NEG	NEG	NEG	NEG	NEG	NEG	NEG
KHDN-10399223	SCCmec IVa (MW2)	CC30	NEG	NEG	POS	NEG	NEG	NEG	NEG	NEG	NEG	NEG	NEG	NEG	NEG	NEG
KHDN-W261158	SCCmec IVa (MW2)	CC30	NEG	NEG	POS	NEG	NEG	NEG	NEG	NEG	NEG	NEG	NEG	NEG	NEG	NEG
UKD-0002046	SCCmec IVa (MW2)	CC45 [agr I]	NEG	NEG	NEG	NEG	NEG	NEG	NEG	NEG	NEG	NEG	NEG	NEG	NEG	NEG
UKD-0029618	SCCmec IVa (MW2)	CC45 [agr I]	NEG	NEG	NEG	NEG	NEG	NEG	NEG	NEG	NEG	NEG	NEG	NEG	NEG	NEG
UKD-0043085	SCCmec IVa (MW2)	CC45 [agr I]	NEG	NEG	NEG	NEG	NEG	NEG	NEG	NEG	NEG	NEG	NEG	NEG	NEG	NEG
UKD-0047053	SCCmec IVa (MW2)	CC45 [agr I]	NEG	NEG	NEG	NEG	NEG	NEG	NEG	NEG	NEG	NEG	NEG	NEG	NEG	NEG
UKD-0047429	SCCmec IVa (MW2)	CC45 [agr I]	NEG	NEG	NEG	NEG	NEG	AMB	POS	NEG	NEG	NEG	NEG	NEG	NEG	NEG
UKD-0112465	SCCmec IVa (MW2)	CC45 [agr I]	NEG	NEG	NEG	NEG	NEG	NEG	NEG	NEG	NEG	NEG	NEG	NEG	NEG	NEG
UKD-0103483	SCCmec IVa (MW2)	CC45 [agr I]	NEG	AMB	NEG	NEG	NEG	NEG	NEG	NEG	NEG	NEG	NEG	NEG	NEG	NEG
UKD-0305014	SCCmec IVa (MW2)	CC45 [agr I]	NEG	NEG	NEG	NEG	NEG	NEG	NEG	NEG	NEG	NEG	NEG	NEG	NEG	NEG
UKD-0401119	SCCmec IVa (MW2)	CC45 [agr I]	NEG	NEG	NEG	NEG	NEG	NEG	NEG	NEG	NEG	NEG	NEG	NEG	NEG	NEG
UKD-0537969	SCCmec IVa (MW2)	CC45 [agr I]	NEG	NEG	NEG	NEG	NEG	NEG	NEG	NEG	NEG	NEG	NEG	NEG	NEG	NEG
UKD-0703373	SCCmec IVa (MW2)	CC45 [agr I]	NEG	NEG	NEG	NEG	NEG	NEG	NEG	NEG	NEG	NEG	NEG	NEG	NEG	NEG
UKD-0813653	SCCmec IVa (MW2)	CC45 [agr I]	NEG	NEG	NEG	NEG	NEG	NEG	NEG	NEG	NEG	NEG	NEG	NEG	NEG	NEG
UKD-0839175	SCCmec IVa (MW2)	CC45 [agr I]	NEG	NEG	NEG	NEG	NEG	NEG	NEG	NEG	NEG	NEG	NEG	NEG	NEG	NEG
UKD-1108422	SCCmec IVa (MW2)	CC45 [agr I]	NEG	NEG	NEG	NEG	NEG	NEG	NEG	NEG	NEG	NEG	NEG	NEG	NEG	NEG
UKD-1380645	SCCmec IVa (MW2)	CC45 [agr I]	NEG	NEG	NEG	NEG	NEG	NEG	NEG	NEG	NEG	NEG	NEG	NEG	NEG	NEG
UKD-148016	SCCmec IVa (MW2)	CC45 [agr I]	NEG	NEG	NEG	NEG	NEG	NEG	NEG	NEG	NEG	NEG	NEG	NEG	NEG	NEG
UKD-16571773	SCCmec IVa (MW2)	CC45 [agr I]	NEG	NEG	NEG	NEG	NEG	NEG	NEG	NEG	NEG	NEG	NEG	NEG	NEG	NEG
KHDN-10407738	SCCmec IVa (MW2)	CC45 [ST617]	NEG	NEG	NEG	NEG	POS	NEG	NEG	NEG	NEG	NEG	NEG	NEG	NEG	NEG
UKD-0853670	SCCmec IVa (MW2)	CC88	NEG	NEG	NEG	NEG	NEG	NEG	NEG	NEG	NEG	NEG	NEG	NEG	NEG	NEG
UKD-1417352	SCCmec IVa (MW2)	CC88	NEG	NEG	NEG	NEG	NEG	NEG	NEG	NEG	NEG	NEG	NEG	NEG	NEG	NEG
UKD-15571283	SCCmec IVa (MW2)	CC88	NEG	NEG	NEG	NEG	NEG	NEG	NEG	NEG	NEG	NEG	NEG	NEG	NEG	NEG
KHDN-10406719	SCCmec IVa (MW2)	ST93	NEG	NEG	NEG	NEG	NEG	NEG	NEG	NEG	NEG	NEG	NEG	NEG	NEG	NEG
KHDN-0060294	SCCmec IVa (MW2)	ST93	NEG	NEG	NEG	NEG	NEG	NEG	NEG	NEG	NEG	NEG	NEG	NEG	NEG	NEG
UKD-0734190	SCCmec IVa (MW2)	CC97	NEG	NEG	NEG	NEG	NEG	NEG	NEG	NEG	NEG	NEG	NEG	NEG	NEG	NEG
UKD-13006983	SCCmec IVa (MW2)	CC97	NEG	NEG	NEG	NEG	NEG	NEG	NEG	NEG	NEG	NEG	NEG	NEG	NEG	NEG
Prediction for H131520133	*SCCmec IVa (H131520133)*	*CC152*	NEG	NEG	NEG	NEG	NEG	NEG	NEG	NEG	NEG	NEG	NEG	NEG	NEG	NEG
KHDN-325892	SCCmec IVa (H131520133)	CC30	NEG	NEG	POS	NEG	NEG	NEG	NEG	NEG	NEG	NEG	NEG	NEG	NEG	NEG
UKD-15580207	SCCmec IVa (H131520133)	CC88	NEG	NEG	NEG	NEG	NEG	NEG	NEG	NEG	NEG	NEG	NEG	NEG	NEG	NEG
UKD-0521159	SCCmec Iva	CC8	NEG	NEG	POS	NEG	NEG	NEG	NEG	NEG	NEG	NEG	NEG	NEG	NEG	NEG
Prediction for CMFT503 : HF569113.1	*SCCmec IVa (CMFT503)*															
Prediction for 21343, GenBank AHKV	*SCCmec IVa (CMFT503)*	*CC88*	NEG	NEG	NEG	NEG	NEG	NEG	NEG	NEG	NEG	NEG	NEG	NEG	NEG	NEG
UKD-1420740	SCCmec IVa (CMFT503)	CC22	NEG	NEG	NEG	NEG	NEG	NEG	NEG	NEG	NEG	NEG	NEG	NEG	NEG	NEG
UKD-15575124	SCCmec IVa (CMFT503)	CC22	NEG	NEG	NEG	NEG	NEG	NEG	NEG	NEG	NEG	NEG	NEG	NEG	NEG	NEG
UKD-16584947	SCCmec IVa (CMFT503)	CC22	NEG	NEG	NEG	NEG	NEG	NEG	NEG	NEG	NEG	NEG	NEG	NEG	NEG	NEG
UKD-16575077	SCCmec IVa (CMFT503)	CC88	NEG	NEG	NEG	NEG	NEG	NEG	NEG	NEG	NEG	NEG	NEG	NEG	NEG	NEG
Prediction for TCH1516, GenBank CP000730.1	*SCCmec IVa+ACME1+Cu (USA300)*	*CC8*	NEG	NEG	POS	NEG	NEG	NEG	NEG	NEG	NEG	NEG	NEG	NEG	NEG	NEG
Reference strain USA300-TCH1516	SCCmec IVa+ACME1+Cu (USA300)	CC8	NEG	NEG	POS	NEG	NEG	NEG	NEG	NEG	NEG	NEG	NEG	NEG	NEG	NEG
Prediction for FPR3757, GenBank CP000255.1	*SCCmec IVa+ACME1+Cu (USA300)*	*CC8*	NEG	NEG	POS	NEG	NEG	NEG	NEG	NEG	NEG	NEG	NEG	NEG	NEG	NEG
UKD-0516386	SCCmec IVa+ACME1+Cu (USA300)	CC8	NEG	NEG	POS	NEG	NEG	NEG	NEG	NEG	NEG	NEG	NEG	NEG	NEG	NEG
UKD-0815773	SCCmec IVa+ACME1+Cu (USA300)	CC8	NEG	NEG	POS	NEG	POS	NEG	NEG	NEG	NEG	NEG	NEG	NEG	NEG	NEG
UKD-1414654	SCCmec IVa+ACME1+Cu (USA300)	CC8	NEG	NEG	POS	AMB	NEG	NEG	NEG	NEG	NEG	NEG	NEG	NEG	NEG	NEG
UKD-0408036	SCCmec IVa+ACME2	CC45 [agr I]	NEG	NEG	NEG	NEG	NEG	NEG	NEG	NEG	NEG	NEG	NEG	NEG	NEG	NEG
UKD-0044035	SCCmec IVa+ccrC (Hannover)	CC8	NEG	NEG	POS	NEG	NEG	NEG	NEG	NEG	NEG	NEG	NEG	NEG	NEG	NEG
UKD-0136287	SCCmec IVa+ccrC (Hannover)	CC8	NEG	NEG	POS	NEG	NEG	NEG	NEG	NEG	NEG	NEG	NEG	NEG	NEG	NEG

Isolate ID	SCCmec type	Clonal complex	RESISTANCE : MISCELLANEOUS GENES													
			23S rRNA methyltransferase	chloramphenicol/ florfenicol exporter	metallothiol transferase		quaternary ammonium compound resistance protein A	quaternary ammonium compound resistance protein C						vancomycin resistance genes		
			cfr	fexA	fosB	fosB (plasmid)	qacA	qacC	qacC (cons)	qacC (equine)	qacC (SA5)	qacC (Ssap)	qacC (ST94)	vanA	vanB	vanZ
Prediction for JCSC1978, GenBank AB063173.1	SCCmec IVb/d/i (JCSC1978/6668/4469)															
Reference strain JCSC1978 (8/6-3P)	SCCmec IVb/d/i (JCSC1978/6668/4469)	CC8	NEG	NEG	POS	NEG	NEG	NEG	NEG	NEG	NEG	NEG	NEG	NEG	NEG	NEG
Prediction for JCSC6668, GenBank AB425823.1	SCCmec IVb/d/i (JCSC1978/6668/4469)															
Reference strain JCSC4469	SCCmec IVb/d/i (JCSC1978/6668/4469)	CC5	NEG	NEG	POS	NEG	NEG	NEG	NEG	NEG	NEG	NEG	NEG	NEG	NEG	NEG
UKD-1103429	SCCmec IVb/d/i (JCSC1978/6668/4469)	CC7	NEG	NEG	NEG	NEG	NEG	NEG	NEG	NEG	NEG	NEG	NEG	NEG	NEG	NEG
UKD-1306141	SCCmec IVb/d/i (JCSC1978/6668/4469)	CC7	NEG	NEG	NEG	NEG	NEG	NEG	NEG	NEG	NEG	NEG	NEG	NEG	NEG	NEG
UKD-0500089	SCCmec IVb/d/i (JCSC1978/6668/4469)	CC5	NEG	NEG	POS	NEG	NEG	NEG	NEG	NEG	NEG	NEG	NEG	NEG	NEG	NEG
UKD-0501416	SCCmec IVb/d/i (JCSC1978/6668/4469)	CC5	NEG	NEG	POS	NEG	NEG	NEG	NEG	NEG	NEG	NEG	NEG	NEG	NEG	NEG
UKD-0934574	SCCmec IVb/d/i (JCSC1978/6668/4469)	CC5	NEG	NEG	POS	NEG	NEG	NEG	NEG	NEG	NEG	NEG	NEG	NEG	NEG	NEG
UKD-0746779	SCCmec IVb/d/i (JCSC1978/6668/4469)	CC45 [agr I]	NEG	NEG	NEG	NEG	NEG	NEG	NEG	NEG	NEG	NEG	NEG	NEG	NEG	NEG
UKD-0812518	SCCmec IVb/d/i (JCSC1978/6668/4469)	CC45 [agr I]	NEG	NEG	NEG	NEG	NEG	NEG	NEG	NEG	NEG	NEG	NEG	NEG	NEG	NEG
Prediction for 21209, GenBank AGRP	SCCmec IVb/d/i (21209)	CC8	NEG	NEG	POS	NEG	NEG	NEG	NEG	NEG	NEG	NEG	NEG	NEG	NEG	NEG
UKD-0233579	SCCmec IVb/d/i (21209)	CC8	NEG	NEG	POS	POS	NEG	POS	NEG	POS	POS	NEG	POS	NEG	NEG	NEG
UKD-0504920	SCCmec IVb/d/i (21209)	CC8	NEG	NEG	POS	NEG	POS	POS	NEG	POS	NEG	NEG	NEG	NEG	NEG	NEG
UKD-0725999	SCCmecIV b/d/i+Cu	CC30	NEG	NEG	POS	NEG	NEG	NEG	NEG	NEG	NEG	NEG	NEG	NEG	NEG	NEG
Prediction for IS-105, GenBank AHLR	SCCmec IVc (IS-105)	CC22	NEG	NEG	NEG	NEG	NEG	NEG	NEG	NEG	NEG	NEG	NEG	NEG	NEG	NEG
UKD-0402421	SCCmec IVc (IS-105), merA/B+	CC5	NEG	NEG	POS	NEG	NEG	NEG	NEG	NEG	NEG	NEG	NEG	NEG	NEG	NEG
UKD-0731319	SCCmec IVc (IS-105), merA/B+	CC5	NEG	NEG	POS	NEG	NEG	NEG	NEG	NEG	NEG	NEG	NEG	NEG	NEG	NEG
UKD-0733137	SCCmec IVc (IS-105)	CC8	NEG	NEG	POS	AMB	POS	NEG	NEG	NEG	NEG	NEG	NEG	NEG	NEG	NEG
UKD-0305690	SCCmec IVc (IS-105)	CC22	NEG	NEG	NEG	NEG	NEG	NEG	NEG	NEG	NEG	NEG	NEG	NEG	NEG	NEG
UKD-1424706	SCCmec IVc (IS-105)	CC22	NEG	NEG	NEG	NEG	NEG	NEG	NEG	NEG	NEG	NEG	NEG	NEG	NEG	NEG
Prediction for TCH60, GenBank CP002110.1	SCCmec IVc (TCH60)	CC30	NEG	NEG	POS	NEG	NEG	NEG	NEG	NEG	NEG	NEG	NEG	NEG	NEG	NEG
Reference strain JCSC4788	SCCmec IVc (TCH60)	CC8	NEG	NEG	POS	NEG	NEG	NEG	NEG	NEG	NEG	NEG	NEG	NEG	NEG	NEG
UKD-1318318	SCCmec IVc (TCH60)	CC5	NEG	NEG	POS	NEG	NEG	NEG	NEG	NEG	NEG	NEG	NEG	NEG	NEG	NEG
UKD-14580050	SCCmec IVc (TCH60)	CC5	NEG	NEG	POS	NEG	NEG	NEG	NEG	NEG	NEG	NEG	NEG	NEG	NEG	NEG
UKD-08F01314	SCCmec IVc (TCH60)	CC8	NEG	NEG	POS	NEG	POS	NEG	NEG	NEG	NEG	NEG	NEG	NEG	NEG	NEG
KHDN-50361518	SCCmec IVc (TCH60)	CC8	NEG	NEG	POS	NEG	NEG	NEG	NEG	NEG	NEG	NEG	NEG	NEG	NEG	NEG
UKD-0307912	SCCmec IVc (TCH60)	CC22	NEG	NEG	NEG	NEG	NEG	NEG	NEG	NEG	NEG	NEG	NEG	NEG	NEG	NEG
UKD-0220390	SCCmec IVc (TCH60)	CC45 [agr I]	NEG	NEG	NEG	NEG	NEG	NEG	NEG	NEG	NEG	NEG	NEG	NEG	NEG	NEG
UKD-0311106	SCCmec IVc (TCH60)	CC45 [agr I]	NEG	NEG	NEG	NEG	NEG	NEG	NEG	NEG	NEG	NEG	NEG	NEG	NEG	NEG
UKD-0719855	SCCmec IVc (TCH60)	CC45 [agr I]	NEG	NEG	NEG	NEG	NEG	NEG	NEG	NEG	NEG	NEG	NEG	NEG	NEG	NEG
UKD-0422079	SCCmec IVc (TCH60)	CC45 [agr I]	NEG	NEG	NEG	NEG	NEG	NEG	NEG	NEG	NEG	NEG	NEG	NEG	NEG	NEG
UKD-0409235	SCCmec IVc (TCH60)	CC80	NEG	NEG	NEG	NEG	NEG	NEG	NEG	NEG	NEG	NEG	NEG	NEG	NEG	NEG
UKD-0409807	SCCmec IVc (TCH60)	CC80	NEG	NEG	NEG	NEG	NEG	NEG	NEG	NEG	NEG	NEG	NEG	NEG	NEG	NEG
UKD-0416073	SCCmec IVc (TCH60)	CC80	NEG	NEG	NEG	NEG	NEG	NEG	NEG	NEG	NEG	NEG	NEG	NEG	NEG	NEG
UKD-0431728	SCCmec IVc (TCH60)	CC80	NEG	NEG	NEG	NEG	NEG	NEG	NEG	NEG	NEG	NEG	NEG	NEG	NEG	NEG
UKD-1001508	SCCmec IVc (TCH60)	CC80	NEG	NEG	NEG	NEG	NEG	NEG	NEG	NEG	NEG	NEG	NEG	NEG	NEG	NEG
UKD-1010412	SCCmec IVc (TCH60)	CC80	NEG	NEG	NEG	NEG	NEG	NEG	NEG	NEG	NEG	NEG	NEG	NEG	NEG	NEG
UKD-12753005	SCCmec IVc (TCH60)	CC80	NEG	NEG	NEG	NEG	NEG	NEG	NEG	NEG	NEG	NEG	NEG	NEG	NEG	NEG
UKD-15574809	SCCmec IVc (TCH60)	CC80	NEG	NEG	NEG	NEG	NEG	NEG	NEG	NEG	NEG	NEG	NEG	NEG	NEG	NEG
KHDN-16120291	SCCmec IVc (TCH60)	CC88	NEG	NEG	NEG	NEG	NEG	NEG	NEG	NEG	NEG	NEG	NEG	NEG	NEG	NEG
Prediction for WW2703/97, GenBank ACSW	SCCmec IVc (WW2703/97)	CC30	NEG	NEG	POS	NEG	NEG	NEG	NEG	NEG	NEG	NEG	NEG	NEG	NEG	NEG
UKD-14580088	SCCmec IVc (WW2703/97)	CC398	NEG	NEG	NEG	NEG	NEG	NEG	NEG	NEG	NEG	NEG	NEG	NEG	NEG	NEG
Prediction for SA_ST125_MupR, GenBank ASTH	SCCmec IVc+speG+ccrAB4 (SA_ST125)	CC5	NEG	NEG	POS	NEG	NEG	NEG	NEG	NEG	NEG	NEG	NEG	NEG	NEG	NEG
UKD-0439909	SCCmec IVc+speG+ccrAB4 (SA_ST125)	CC5	NEG	NEG	POS	NEG	NEG	NEG	NEG	NEG	NEG	NEG	NEG	NEG	NEG	NEG
Prediction for MRSA177, GenBank AECP	SCCmec IVc+Cu/Hg (MRSA177)		NEG	NEG	POS	NEG	NEG	NEG	NEG	NEG	NEG	NEG	NEG	NEG	NEG	NEG
UKD-1279734	SCCmec IVc+Cu/Hg (MRSA177)	CC8	NEG	NEG	POS	NEG	NEG	NEG	NEG	NEG	NEG	NEG	NEG	NEG	NEG	NEG
UKD-14580899	SCCmec IVc+Cu/Hg (MRSA177)	CC8	NEG	NEG	POS	AMB	NEG	NEG	NEG	NEG	NEG	NEG	NEG	NEG	NEG	NEG
UKD-16002228	SCCmec IVc+Cu/Hg (MRSA177)	CC8	NEG	NEG	POS	NEG	NEG	NEG	NEG	NEG	NEG	NEG	NEG	NEG	NEG	NEG
Prediction for HO 5096 0412, GenBank HE681097.1	SCCmec IVh/j (HO50960412/JCSC6670)	CC22	NEG	NEG	NEG	NEG	NEG	NEG	NEG	NEG	NEG	NEG	NEG	NEG	NEG	NEG
Prediction for JCSC6670, GenBank AB425824.1	SCCmec IVh/j (HO50960412/JCSC6670)															
Reference strain Dublin_DSH_E1749	SCCmec IVh/j (HO50960412/JCSC6670)		NEG	NEG	NEG	NEG	NEG	NEG	NEG	NEG	NEG	NEG	NEG	NEG	NEG	NEG
UKD-14576248	SCCmec IVh/j (HO50960412/JCSC6670)	CC8	NEG	NEG	POS	NEG	NEG	NEG	NEG	NEG	NEG	NEG	NEG	NEG	NEG	NEG
UKD-0120122	SCCmec IVh/j (HO50960412/JCSC6670)	CC22	NEG	NEG	NEG	NEG	NEG	NEG	NEG	NEG	NEG	NEG	NEG	NEG	NEG	NEG
UKD-0205319	SCCmec IVh/j (HO50960412/JCSC6670)	CC22	NEG	NEG	NEG	NEG	NEG	NEG	NEG	NEG	NEG	NEG	NEG	NEG	NEG	NEG
UKD-0221259	SCCmec IVh/j (HO50960412/JCSC6670)	CC22	NEG	NEG	NEG	NEG	NEG	NEG	NEG	NEG	NEG	NEG	NEG	NEG	NEG	NEG
UKD-0418654	SCCmec IVh/j (HO50960412/JCSC6670)	CC22	NEG	NEG	NEG	NEG	POS	NEG	NEG	NEG	NEG	NEG	NEG	NEG	NEG	NEG
UKD-0429721	SCCmec IVh/j (HO50960412/JCSC6670)	CC22	NEG	NEG	NEG	NEG	NEG	NEG	NEG	NEG	NEG	NEG	NEG	NEG	NEG	NEG
UKD-1116194	SCCmec IVh/j (HO50960412/JCSC6670)	CC22	NEG	NEG	NEG	NEG	NEG	NEG	NEG	NEG	NEG	NEG	NEG	NEG	NEG	NEG
UKD-1122367	SCCmec IVh/j (HO50960412/JCSC6670)	CC22	NEG	NEG	NEG	NEG	NEG	NEG	NEG	NEG	NEG	NEG	NEG	NEG	NEG	NEG
UKD-14012840	SCCmec IVh/j (HO50960412/JCSC6670)	CC22	NEG	NEG	NEG	POS	NEG	NEG	NEG	NEG	NEG	NEG	NEG	NEG	NEG	NEG
UKD-14579833	SCCmec IVh/j (HO50960412/JCSC6670)	CC22	NEG	NEG	NEG	NEG	NEG	NEG	NEG	NEG	NEG	NEG	NEG	NEG	NEG	NEG
UKD-15579198	SCCmec IVh/j (HO50960412/JCSC6670)	CC22	NEG	NEG	NEG	NEG	NEG	NEG	NEG	NEG	NEG	NEG	NEG	NEG	NEG	NEG
UKD-4052193	SCCmec IVh/j (HO50960412/JCSC6670)	CC22	NEG	NEG	NEG	NEG	NEG	NEG	NEG	NEG	NEG	NEG	NEG	NEG	NEG	NEG
UKD-124968	SCCmec IVh/j (HO50960412/JCSC6670)	CC22	NEG	NEG	NEG	NEG	NEG	POS	POS	NEG	NEG	NEG	NEG	NEG	NEG	NEG
UKD-14572546	SCCmec IVh/j (HO50960412/JCSC6670)	CC22	NEG	NEG	NEG	NEG	NEG	NEG	NEG	NEG	NEG	NEG	NEG	NEG	NEG	NEG
UKD-14582287	SCCmec IVh/j (HO50960412/JCSC6670)	CC22	NEG	NEG	NEG	NEG	NEG	NEG	NEG	NEG	NEG	NEG	NEG	NEG	NEG	NEG
UKD-14583376	SCCmec IVh/j (HO50960412/JCSC6670)	CC22	NEG	NEG	NEG	NEG	NEG	NEG	NEG	NEG	NEG	NEG	NEG	NEG	NEG	NEG
UKD-14583496	SCCmec IVh/j (HO50960412/JCSC6670)	CC22	NEG	NEG	NEG	NEG	NEG	NEG	NEG	NEG	NEG	NEG	NEG	NEG	NEG	NEG
UKD-0013324	SCCmec IVh/j (HO50960412/JCSC6670)	CC22	NEG	NEG	NEG	NEG	NEG	NEG	NEG	NEG	NEG	NEG	NEG	NEG	NEG	NEG
UKD-0125329	SCCmec IVh/j (HO50960412/JCSC6670)	CC22	NEG	NEG	NEG	NEG	NEG	NEG	NEG	NEG	NEG	NEG	NEG	NEG	NEG	NEG
UKD-0215498	SCCmec IVh/j (HO50960412/JCSC6670)	CC22	NEG	NEG	NEG	NEG	NEG	NEG	NEG	NEG	NEG	NEG	NEG	NEG	NEG	NEG
Prediction for M08-0126, GenBank FR753166.1	SCCmec IVh/j+ACME2 (M08-0126)	CC22														
UKD-1379483I	SCCmec IVh/j+ACME2 (M08-0126)	CC22	NEG	NEG	NEG	NEG	NEG	POS	POS	NEG	NEG	POS	NEG	NEG	NEG	NEG
UKD-5150204	SCCmec IV+speG+Cu/czrC+ccrAB4	CC22	NEG	NEG	NEG	NEG	POS	NEG	NEG	NEG	NEG	NEG	NEG	NEG	NEG	NEG
UKD-15578423	SCCmec IV+speG+czrC+ccrAB4 var. 1	CC22	NEG	NEG	NEG	NEG	POS	NEG	NEG	NEG	NEG	NEG	NEG	NEG	NEG	NEG
UKD-16582870	SCCmec IV+speG+czrC+ccrAB4 var. 1	CC22	NEG	NEG	NEG	NEG	POS	NEG	NEG	NEG	NEG	NEG	NEG	NEG	NEG	NEG
UKD-15571940	SCCmec IV+speG+czrC+ccrAB4 var. 2	CC22	NEG	NEG	NEG	NEG	NEG	NEG	NEG	NEG	NEG	NEG	NEG	NEG	NEG	NEG

Isolate ID	SCCmec type	Clonal complex	RESISTANCE : MISCELLANEOUS GENES													
			23S rRNA methyltransferase	chloramphenicol/ florfenicol exporter	metallothiol transferase		quaternary ammonium compound resistance protein A	quaternary ammonium compound resistance protein C						vancomycin resistance genes		
			cfr	fexA	fosB	fosB (plasmid)	qacA	qacC	qacC (cons)	qacC (equine)	qacC (SA5)	qacC (Ssap)	qacC (ST94)	vanA	vanB	vanZ
Prediction for CN1, GenBank CP003979.1	*SCCmec IVA (CN1)*	*CC8 (ST72)*	NEG	NEG	POS	NEG	NEG	NEG	NEG	NEG	NEG	NEG	NEG	NEG	NEG	NEG
UKD-0900871	SCCmec IVA (CN1)	CC8 (ST72)	NEG	NEG	POS	NEG	NEG	NEG	NEG	NEG	NEG	NEG	NEG	NEG	NEG	NEG
Prediction for CMFT1723, GenBank HF569096.1	*SCCmec V (Bengal Bay)*															
KHDN-10405134	SCCmec V (Bengal Bay)	CC1	NEG	NEG	NEG	NEG	NEG	NEG	NEG	NEG	NEG	NEG	NEG	NEG	NEG	NEG
UKD-0932405	SCCmec V (Bengal Bay)	CC1 (ST573/772)	NEG	NEG	POS	NEG	NEG	NEG	NEG	NEG	NEG	NEG	NEG	NEG	NEG	NEG
UKD-1003510	SCCmec V (Bengal Bay)	CC1 (ST573/772)	NEG	NEG	POS	NEG	NEG	NEG	NEG	NEG	NEG	NEG	NEG	NEG	NEG	NEG
UKD-1112420	SCCmec V (Bengal Bay)	CC1 (ST573/772)	NEG	NEG	POS	NEG	NEG	NEG	NEG	NEG	NEG	NEG	NEG	NEG	NEG	NEG
UKD-1275832	SCCmec V (Bengal Bay)	CC1 (ST573/772)	NEG	NEG	POS	NEG	NEG	NEG	NEG	NEG	NEG	NEG	NEG	NEG	NEG	NEG
UKD-13023811	SCCmec V (Bengal Bay)	CC1 (ST573/772)	NEG	NEG	POS	NEG	NEG	NEG	NEG	NEG	NEG	NEG	NEG	NEG	NEG	NEG
UKD-14572163	SCCmec V	CC5	NEG	POS	POS	NEG	NEG	NEG	NEG	NEG	NEG	NEG	NEG	NEG	NEG	NEG
KHDN-10421133	SCCmec V+fus	CC97	NEG	NEG	NEG	NEG	NEG	NEG	NEG	NEG	NEG	NEG	NEG	NEG	NEG	NEG
KHDN-21343541	SCCmec V+fus+tir+ccrAB1	CC1	NEG	NEG	NEG	NEG	NEG	NEG	NEG	NEG	NEG	NEG	NEG	NEG	NEG	NEG
KHDN-10416893	SCCmec V+czrC+speG+ccrAB4	CC22	NEG	NEG	NEG	NEG	NEG	NEG	NEG	NEG	NEG	NEG	NEG	NEG	NEG	NEG
Prediction for GR1, GenBank AJLX	*SCCmec VT (GR1)*	*CC361*	NEG	NEG	POS	NEG	NEG	NEG	NEG	NEG	NEG	NEG	NEG	NEG	NEG	NEG
UKD-0422872	SCCmec VT (GR1)	CC152	NEG	NEG	NEG	NEG	NEG	NEG	NEG	NEG	NEG	NEG	NEG	NEG	NEG	NEG
Prediction for PM1, GenBank BAFA	*SCCmec VT (PM1)*	*CC59*	NEG	NEG	NEG	NEG	NEG	NEG	NEG	NEG	NEG	NEG	NEG	NEG	NEG	NEG
KHDN-000871	SCCmec VT (PM1)	CC45 (agr I)	NEG	NEG	NEG	NEG	NEG	NEG	NEG	NEG	NEG	NEG	NEG	NEG	NEG	NEG
UKD-0725052	SCCmec VT (PM1)	CC59	NEG	NEG	NEG	NEG	NEG	NEG	NEG	NEG	NEG	NEG	NEG	NEG	NEG	NEG
UKD-1312138	SCCmec VT (PM1)	CC59	NEG	NEG	NEG	NEG	NEG	NEG	NEG	NEG	NEG	NEG	NEG	NEG	NEG	NEG
UKD-Z4728	SCCmec VT (PM1)	CC59	NEG	NEG	NEG	NEG	NEG	NEG	NEG	NEG	NEG	NEG	NEG	NEG	NEG	NEG
UKD-K0199	SCCmec VT unknown var.1	CC7	NEG	NEG	NEG	NEG	NEG	NEG	NEG	NEG	NEG	NEG	NEG	NEG	NEG	NEG
UKD-15584619	SCCmec VT unknown var.2	CC30	NEG	NEG	POS	NEG	NEG	NEG	NEG	NEG	NEG	NEG	NEG	NEG	NEG	NEG
UKD-0712568	SCCmec VT unknown var.3	CC45 (agr I)	NEG	NEG	NEG	NEG	NEG	NEG	NEG	NEG	NEG	NEG	NEG	NEG	NEG	NEG
KHDN-10420296	SCCmec VT+ACME2	CC45 (agr I)	NEG	NEG	NEG	NEG	NEG	NEG	NEG	NEG	NEG	NEG	NEG	NEG	NEG	NEG
UKD-15577956	SCCmec VT+Cu/czrC+ccrA1	CC398	NEG	NEG	NEG	NEG	NEG	NEG	NEG	NEG	NEG	NEG	NEG	NEG	NEG	NEG
Prediction for S0385, GenBank AM990992	*SCCmec VT+czrC (SO385)*	*CC398*	NEG	NEG	NEG	NEG	NEG	NEG	NEG	NEG	NEG	NEG	NEG	NEG	NEG	NEG
UKD-0628560	SCCmec VT+czrC (SO385)	CC8	NEG	NEG	POS	NEG	NEG	NEG	NEG	NEG	NEG	NEG	NEG	NEG	NEG	NEG
UKD-0510018	SCCmec VT+czrC (SO385)	CC398	NEG	NEG	NEG	NEG	NEG	NEG	NEG	NEG	NEG	NEG	NEG	NEG	NEG	NEG
UKD-0903558	SCCmec VT+czrC (SO385)	CC398	NEG	NEG	NEG	NEG	NEG	NEG	NEG	NEG	NEG	NEG	NEG	NEG	NEG	NEG
UKD-0929815	SCCmec VT+czrC (SO385)	CC398	NEG	NEG	NEG	NEG	NEG	NEG	NEG	NEG	NEG	NEG	NEG	NEG	NEG	NEG
UKD-0907762	SCCmec VT+czrC (SO385)	CC398	NEG	NEG	NEG	NEG	NEG	NEG	NEG	NEG	NEG	NEG	NEG	NEG	NEG	NEG
UKD-1025105	SCCmec VT+czrC (SO385)	CC398	NEG	NEG	NEG	NEG	POS	NEG	NEG	NEG	NEG	NEG	NEG	NEG	NEG	NEG
UKD-11780898	SCCmec VT+czrC (SO385)	CC398	NEG	NEG	NEG	NEG	NEG	NEG	NEG	NEG	NEG	NEG	NEG	NEG	NEG	NEG
UKD-1377009	SCCmec VT+czrC (SO385)	CC398	NEG	NEG	NEG	NEG	NEG	NEG	NEG	NEG	NEG	NEG	NEG	NEG	NEG	NEG
UKD-143406	SCCmec VT+czrC (SO385)	CC398	NEG	NEG	NEG	NEG	NEG	NEG	NEG	NEG	NEG	NEG	NEG	NEG	NEG	NEG
UKD-1400363	SCCmec VT+czrC (SO385)	CC398	NEG	NEG	NEG	NEG	NEG	NEG	NEG	NEG	NEG	NEG	NEG	NEG	NEG	NEG
UKD-14571852	SCCmec VT+czrC (SO385)	CC398	NEG	NEG	POS	NEG	NEG	NEG	NEG	NEG	NEG	NEG	NEG	NEG	NEG	NEG
UKD-14582574	SCCmec VT+czrC (SO385)	CC398	NEG	NEG	NEG	NEG	NEG	NEG	NEG	NEG	NEG	NEG	NEG	NEG	NEG	NEG
UKD-152108	SCCmec VT+czrC (SO385)	CC398	NEG	NEG	NEG	NEG	NEG	NEG	NEG	NEG	NEG	NEG	NEG	NEG	NEG	NEG
UKD-15573321	SCCmec VT+czrC (SO385)	CC398	NEG	NEG	NEG	NEG	NEG	NEG	NEG	NEG	NEG	NEG	NEG	NEG	NEG	NEG
UKD-15584034	SCCmec VT+czrC , ydhK-neg. variant	CC398	NEG	NEG	NEG	NEG	NEG	NEG	NEG	NEG	NEG	NEG	NEG	NEG	NEG	NEG
UKD-0932581	SCCmecVT+fus+czrC	CC22	NEG	NEG	NEG	NEG	NEG	NEG	NEG	NEG	NEG	NEG	NEG	NEG	NEG	NEG
UKD-1121994	SCCmecVT+fus+czrC	CC22	NEG	NEG	NEG	NEG	NEG	NEG	NEG	NEG	NEG	NEG	NEG	NEG	NEG	NEG
UKD-1272252	SCCmecVT+fus+czrC	CC22	NEG	NEG	NEG	NEG	NEG	NEG	NEG	NEG	NEG	NEG	NEG	NEG	NEG	NEG
Prediction for MRSA18	*SCCmec VI+fus (MRSA18)*															
UKD-1103380	SCCmec VI+fus (MRSA18)	CC7	NEG	NEG	NEG	NEG	NEG	NEG	NEG	NEG	NEG	NEG	NEG	NEG	NEG	NEG
Prediction for M10/0061, GenBank FR823292.1	*SCCmec XI (LGA251/M10-61)*	*CC130*														
Reference strain Dublin-DSH_M10 0061	SCCmec XI (LGA251/M10-61)	CC130	NEG	NEG	NEG	NEG	NEG	NEG	NEG	NEG	NEG	NEG	NEG	NEG	NEG	NEG
Prediction for LGA251, GenBank FR821779.1	*SCCmec XI (LGA251/M10-61)*	*ST425*														
UKD-14015835	SCCmec XI (LGA251/M10-61)	CC130	NEG	NEG	NEG	NEG	NEG	NEG	NEG	NEG	NEG	NEG	NEG	NEG	NEG	NEG
UKD-16573252	SCCmec XI (LGA251/M10-61)	CC130	NEG	NEG	NEG	NEG	NEG	NEG	NEG	NEG	NEG		NEG	NEG	NEG	NEG
UKD-0217002	PseudoSCCmec, class B mec complex+Hg	CC8	NEG	NEG	POS	NEG	NEG	NEG	NEG	NEG	NEG	NEG	NEG	NEG	NEG	NEG
UKD-0008510	PseudoSCCmec, class B mec complex+Hg	CC8	NEG	NEG	POS	NEG	NEG	NEG	NEG	NEG	NEG	NEG	NEG	NEG	NEG	NEG
UKD-0110962	PseudoSCCmec, class B mec complex+Hg	CC8	NEG	NEG	POS	AMB	NEG	NEG	NEG	NEG	NEG	NEG	NEG	NEG	NEG	NEG
KHDN-10408593	PseudoSCCmec, class B mec complex+Cu/Hg	CC8	NEG	NEG	POS	NEG	NEG	NEG	NEG	NEG	NEG	NEG	NEG	NEG	NEG	NEG
UKD-0007201	PseudoSCCmec, class B mec complex	CC5	NEG	NEG	POS	NEG	POS	NEG	NEG	NEG	NEG	NEG	NEG	NEG	NEG	NEG
UKD-0033204	PseudoSCCmec, class B mec complex	CC5	NEG	NEG	POS	NEG	POS	NEG	NEG	NEG	NEG	NEG	NEG	NEG	NEG	NEG
UKD-0134329	PseudoSCCmec, class B mec complex	CC5	NEG	NEG	AMB	NEG	POS	NEG	NEG	NEG	NEG	NEG	NEG	NEG	NEG	NEG
UKD-0806946	PseudoSCCmec class C+As/Cu	CC398	NEG	NEG	NEG	NEG	NEG	NEG	NEG	NEG	NEG	NEG	NEG	NEG	NEG	NEG
UKD-0500610	ACME2	CC8	NEG	NEG	POS	NEG	POS	POS	POS	NEG	NEG	NEG	NEG	NEG	NEG	NEG
UKD-0610438	ACME2	CC8	NEG	NEG	POS	NEG	POS	POS	POS	NEG	NEG	NEG	NEG	NEG	NEG	NEG
KHDN-10409344	ACME2	CC8	NEG	NEG	POS	NEG	NEG	NEG	NEG	NEG	NEG	NEG	NEG	NEG	NEG	NEG
UKD-0322559	ACME2+speG+czrC+ccrAB4	CC8	NEG	NEG	POS	NEG	POS	NEG	NEG	NEG	NEG	NEG	NEG	NEG	NEG	NEG
UKD-0501998	ACME2+speG+czrC+ccrAB4	CC8	NEG	NEG	POS	NEG	POS	NEG	NEG	NEG	NEG	NEG	NEG	NEG	NEG	NEG
UKD-0505213	ACME2+speG+czrC+ccrAB4	CC8	NEG	NEG	POS	AMB	POS	NEG	NEG	NEG	NEG	NEG	NEG	NEG	NEG	NEG
Prediction for MSSA476, GenBank BX571857.1	*fus+tir+ccrAB1 (MSSA476)*	*CC1*	NEG	NEG	NEG	NEG	NEG	NEG	NEG	NEG	NEG	NEG	NEG	NEG	NEG	NEG
Reference strain Sanger 476	fus+tir+ccrAB1 (MSSA476)	CC1	NEG	NEG	NEG	NEG	NEG	NEG	NEG	NEG	NEG	NEG	NEG	NEG	NEG	NEG
UKD-14023914	fus+tir+ccrAB1 (MSSA476)	CC1	NEG	NEG	NEG	NEG	NEG	NEG	NEG	NEG	NEG	NEG	NEG	NEG	NEG	NEG
UKD-K00034	kdp+ccrAB2	CC182	NEG	NEG	NEG	NEG	NEG	NEG	NEG	NEG	NEG	NEG	NEG	NEG	NEG	NEG
UKD-15580369	As+ccrAB4	CC22	NEG	NEG	NEG	NEG	POS	POS	POS	NEG	NEG	NEG	NEG	NEG	NEG	NEG
UKD-15570100	ACME3+ccrAB1 (Strain 21342)	CC80	NEG	NEG	NEG	NEG	NEG	NEG	NEG	NEG	NEG	NEG	NEG	NEG	NEG	NEG
UKD-1010678	speG+czrC+ccrAB4	CC8	NEG	NEG	POS	NEG	NEG	NEG	NEG	NEG	NEG	NEG	NEG	NEG	NEG	NEG
UKD-1008243	speG+czrC+ccrAB4	CC8	NEG	NEG	POS	NEG	NEG	NEG	NEG	NEG	NEG	NEG	NEG	NEG	NEG	NEG
UKD-1009430	speG+czrC+ccrAB4	CC8	NEG	NEG	POS	NEG	NEG	NEG	NEG	NEG	NEG	NEG	NEG	NEG	NEG	NEG
UKD-12071828	speG+czrC+ccrAB4	CC22	NEG	NEG	NEG	NEG	NEG	NEG	NEG	NEG	NEG	NEG	NEG	NEG	NEG	NEG
UKD-14011883	speG+czrC+ccrAB4	CC22	NEG	NEG	NEG	NEG	POS	POS	POS	NEG	NEG	NEG	NEG	NEG	NEG	NEG
UKD-15008293	speG+czrC+ccrAB4	CC22	NEG	NEG	NEG	NEG	POS	POS	POS	NEG	NEG	NEG	NEG	NEG	NEG	NEG
UKD-1514877	speG+czrC+ccrAB4	CC22	NEG	NEG	NEG	NEG	POS	POS	POS	NEG	NEG	NEG	NEG	NEG	NEG	NEG
UKD-15580971	speG+czrC+ccrAB4	CC22	NEG	NEG	NEG	NEG	POS	NEG	NEG	NEG	NEG	NEG	NEG	NEG	NEG	NEG
UKD-15582646	speG+czrC+ccrAB4	CC22	NEG	NEG	NEG	NEG	POS	NEG	NEG	NEG	NEG	NEG	NEG	NEG	NEG	NEG

Isolate ID	SCCmec type	Clonal complex	SPECIES MARKER							REGULATORY GENES									VIRULENCE : TOX.SCHOCK.TOXIN			VIRULENCE : ENTEROTOXINS											
			Domain 1 of 23S-rRNA	glyceraldehyde 3-phosphate dehydrogenase, locus 1	katalase A	coagulase	thermostable extracellular nuclease	staphylococcal protein A	IgG-binding protein	staphylococcal accessory regulator A	histidine protein kinase, sae locus	sensor protein	accessory gene regulator alleles				haemolysin delta	toxic shock syndrome toxin 1			Entero-toxin A	Entero-toxin A, allele from strain 320E	Entero-toxin A, allele from strain N315 = Entero-toxin P	Entero-toxin B	Entero-toxin C	Entero-toxin D	Entero-toxin E	Entero-toxin H	Entero-toxin J	Entero-toxin K	Entero-toxin L
			rrnD1 (S. aureus)	gapA	katA	CoA	nuc1	spa	sbi	sarA	saeS	vraS	agrI (total)	agrII (total)	agrIII (total)	agrIV (total)	hld	tst1 (consensus)	tst1 ("human" allele)	tst1 ("bovine" allele)	entA	entA (320E)	entA (N315) / entP	entB	entC	entD	entE	entH	entJ	entK	entL
VIRTUAL HYBRIDISATION for COL, GenBank CP000046	SCCmec I (COL)	CC8	POS	POS	POS	POS	POS	POS	POS	POS	POS	POS	POS	NEG	NEG	NEG	POS	NEG	NEG	NEG	NEG	NEG	NEG	POS	NEG	NEG	NEG	NEG	NEG	POS	NEG
Reference strain COL	SCCmec I (COL)	CC8	POS	POS	POS	POS	POS	POS	POS	POS	POS	POS	POS	NEG	NEG	AMB	POS	NEG	NEG	NEG	NEG	NEG	NEG	POS	NEG	NEG	NEG	NEG	NEG	POS	NEG
UKD-0007063	SCCmec I (COL)	CC5	POS	POS	POS	POS	POS	POS	POS	POS	POS	POS	NEG	POS	NEG	NEG	POS	NEG	NEG	NEG	NEG	NEG	NEG	NEG	NEG	NEG	NEG	NEG	NEG	NEG	NEG
UKD-0220759	SCCmec I (COL)	CC5	POS	POS	POS	POS	POS	POS	POS	POS	POS	POS	NEG	POS	NEG	NEG	POS	NEG	NEG	NEG	POS	NEG	NEG	NEG	NEG	NEG	NEG	NEG	NEG	NEG	NEG
UKD-0308986	SCCmec I (COL)	CC5	POS	POS	POS	POS	POS	POS	POS	POS	POS	POS	NEG	POS	NEG	NEG	POS	NEG	NEG	NEG	POS	NEG	NEG	NEG	NEG	NEG	NEG	NEG	NEG	NEG	NEG
UKD-0500009	SCCmec I (COL)	CC5	POS	POS	POS	POS	POS	POS	POS	POS	POS	POS	NEG	POS	NEG	NEG	POS	NEG	NEG	NEG	POS	NEG	NEG	NEG	NEG	NEG	NEG	NEG	NEG	NEG	NEG
UKD-0508483	SCCmec I (COL)	CC5	POS	POS	POS	POS	POS	POS	POS	POS	POS	POS	NEG	POS	NEG	NEG	POS	NEG	NEG	NEG	POS	NEG	NEG	NEG	NEG	NEG	NEG	NEG	NEG	NEG	NEG
UKD-0519001	SCCmec I (COL)	CC5	POS	POS	POS	POS	POS	POS	POS	POS	POS	POS	NEG	POS	NEG	NEG	POS	NEG	NEG	NEG	POS	AMB	NEG	NEG	NEG	NEG	NEG	NEG	NEG	NEG	NEG
UKD-0701876	SCCmec I (COL)	CC5	POS	POS	POS	POS	POS	POS	POS	POS	POS	POS	NEG	POS	NEG	NEG	POS	NEG	NEG	NEG	POS	NEG	NEG	NEG	NEG	NEG	NEG	NEG	NEG	NEG	NEG
UKD-0708865	SCCmec I (COL)	CC5	POS	POS	POS	POS	POS	POS	POS	POS	POS	POS	NEG	POS	NEG	NEG	POS	NEG	NEG	NEG	POS	NEG	NEG	NEG	NEG	NEG	NEG	NEG	NEG	NEG	NEG
UKD-1001782	SCCmec I (COL)	CC5	POS	POS	POS	POS	POS	POS	POS	POS	POS	POS	NEG	POS	NEG	NEG	POS	NEG	NEG	NEG	POS	NEG	NEG	NEG	NEG	NEG	NEG	NEG	NEG	NEG	NEG
Prediction for PSP1996, GenBank ANHU	SCCmec I (PSP1996)	CC8	POS	POS	POS	POS	POS	POS	POS	AMB	POS	POS	POS	NEG	NEG	NEG	POS	NEG	NEG	NEG	NEG	NEG	NEG	NEG	NEG	NEG	NEG	NEG	NEG	NEG	NEG
UKD-0261727	SCCmec I (PSP1996)	CC8	POS	POS	POS	POS	POS	POS	POS	POS	POS	POS	POS	NEG	NEG	NEG	POS	NEG	NEG	NEG	POS	NEG	NEG	NEG	NEG	NEG	NEG	NEG	NEG	NEG	NEG
VIRTUAL HYBRIDISATION for MRSA7 : VIRTUAL HYBRIL	SCCmec I+fus+tirS (Geraldine Clone)	CC5	POS	POS	POS	POS	POS	POS	POS	POS	POS	POS	NEG	POS	NEG	NEG	POS	POS	POS	NEG	NEG	NEG	NEG	NEG	POS	POS	NEG	NEG	POS	NEG	POS
UKD-1221930	SCCmec I+fus+tirS (Geraldine Clone)	CC5	POS	POS	POS	POS	POS	POS	POS	POS	POS	POS	NEG	POS	NEG	NEG	POS	POS	POS	NEG	NEG	NEG	NEG	NEG	POS	POS	NEG	NEG	POS	NEG	POS
Prediction for N315, GenBank BA000018.3	SCCmec II (N315)	CC5	POS	POS	POS	POS	POS	POS	POS	AMB	POS	POS	NEG	POS	NEG	NEG	POS	POS	POS	NEG	NEG	NEG	POS	NEG	POS	NEG	NEG	NEG	NEG	NEG	POS
Reference strain N315	SCCmec II (N315)	CC5	POS	POS	POS	POS	POS	POS	POS	POS	POS	POS	NEG	POS	NEG	NEG	POS	POS	POS	NEG	NEG	NEG	POS	NEG	POS	NEG	NEG	NEG	NEG	NEG	POS
Prediction for MRSA252, GenBank BX571856.1	SCCmec II (N315)	CC30	POS	POS	POS	POS	POS	POS	POS	AMB	POS	POS	NEG	NEG	POS	NEG	POS	NEG	NEG	NEG	POS	NEG	NEG	NEG	NEG	NEG	NEG	NEG	NEG	NEG	NEG
Reference strain Sanger 252	SCCmec II (N315)	CC30	POS	POS	POS	POS	POS	POS	POS	POS	POS	POS	NEG	NEG	POS	NEG	POS	NEG	NEG	NEG	POS	NEG	NEG	NEG	NEG	NEG	NEG	NEG	NEG	NEG	NEG
UKD-0230792	SCCmec II (N315)	CC30	POS	POS	POS	POS	POS	POS	POS	POS	POS	POS	NEG	NEG	POS	NEG	POS	POS	POS	NEG	POS	NEG	NEG	NEG	NEG	NEG	NEG	NEG	NEG	NEG	NEG
Prediction for Mu50, GenBank BA000017.4	SCCmec II (N315)	CC5	POS	POS	POS	POS	POS	POS	POS	AMB	POS	POS	NEG	POS	NEG	NEG	POS	POS	POS	NEG	POS	NEG	NEG	NEG	POS	NEG	NEG	NEG	NEG	NEG	POS
Reference strain MU50	SCCmec II (N315)	CC5	POS	POS	POS	POS	POS	POS	POS	= NARSA	POS	POS	NEG	POS	NEG	NEG	POS	POS	POS	NEG	POS	NEG	NEG	NEG	POS	NEG	NEG	NEG	NEG	NEG	POS
Prediction for JH1, GenBank CP000736.1	SCCmec II (JH1/JH9)	CC5	POS	POS	POS	POS	POS	POS	POS	AMB	POS	POS	NEG	POS	NEG	NEG	POS	NEG	NEG	NEG	NEG	NEG	NEG	NEG	NEG	NEG	NEG	NEG	NEG	NEG	NEG
Prediction for JH9, GenBank CP000703.1	SCCmec II (JH1/JH9)	CC5	POS	POS	POS	POS	POS	POS	POS	AMB	POS	POS	NEG	POS	NEG	NEG	POS	NEG	NEG	NEG	NEG	NEG	NEG	NEG	NEG	NEG	NEG	NEG	NEG	NEG	NEG
Reference strain Dublin-DSH_AR07.4_0237	SCCmec II (JH1/JH9)		POS	POS	POS	POS	POS	POS	POS	POS	POS	POS	NEG	POS	NEG	NEG	POS	POS	POS	NEG	NEG	NEG	POS	NEG	NEG	POS	NEG	NEG	POS	NEG	NEG
Reference strain NARSA_382 / USA100	SCCmec II (JH1/JH9)	CC5	POS	POS	POS	POS	POS	POS	POS	POS	POS	POS	NEG	POS	NEG	NEG	POS	NEG	NEG	NEG	NEG	NEG	NEG	NEG	NEG	POS	NEG	NEG	POS	NEG	NEG
UKD-0046921	SCCmec II (JH1/JH9)	CC5	POS	POS	POS	POS	POS	POS	POS	POS	POS	POS	NEG	POS	NEG	NEG	POS	NEG	NEG	NEG	NEG	NEG	POS	NEG	NEG	POS	NEG	NEG	POS	NEG	NEG
UKD-0333415	SCCmec II (JH1/JH9)	CC5	POS	POS	POS	POS	POS	POS	POS	POS	POS	POS	NEG	POS	NEG	NEG	POS	NEG	NEG	NEG	NEG	NEG	POS	NEG	NEG	NEG	NEG	NEG	NEG	NEG	NEG
UKD-0341645	SCCmec II (JH1/JH9)	CC5	POS	POS	POS	POS	POS	POS	POS	POS	POS	POS	NEG	POS	NEG	NEG	POS	NEG	NEG	NEG	NEG	NEG	POS	NEG	NEG	POS	NEG	NEG	POS	NEG	NEG
UKD-0504626	SCCmec II (JH1/JH9)	CC5	POS	POS	POS	AMB	POS	POS	POS	POS	POS	POS	NEG	POS	NEG	NEG	AMB	NEG	NEG	NEG	NEG	NEG	POS	NEG	NEG	POS	NEG	NEG	POS	NEG	NEG
UKD-0507581	SCCmec II (JH1/JH9)	CC5	POS	POS	POS	POS	POS	POS	POS	POS	POS	POS	NEG	POS	NEG	NEG	POS	NEG	NEG	NEG	NEG	NEG	POS	NEG	NEG	POS	NEG	NEG	POS	NEG	NEG
UKD-0507903	SCCmec II (JH1/JH9)	CC5	POS	POS	POS	POS	POS	POS	POS	POS	POS	POS	NEG	POS	NEG	NEG	POS	NEG	NEG	NEG	NEG	NEG	POS	NEG	NEG	NEG	NEG	NEG	NEG	NEG	NEG
UKD-0700817	SCCmec II (JH1/JH9)	CC5	POS	POS	POS	POS	POS	POS	POS	POS	POS	POS	NEG	POS	NEG	NEG	POS	NEG	NEG	NEG	NEG	NEG	POS	NEG	NEG	NEG	NEG	NEG	NEG	NEG	NEG
UKD-1014561	SCCmec II (JH1/JH9)	CC5	POS	POS	POS	POS	POS	POS	POS	POS	POS	POS	NEG	POS	NEG	NEG	POS	NEG	NEG	NEG	NEG	NEG	POS	NEG	NEG	NEG	NEG	NEG	NEG	NEG	NEG
UKD-1037507	SCCmec II (JH1/JH9)	CC5	POS	POS	POS	POS	POS	POS	POS	POS	POS	POS	NEG	POS	NEG	NEG	POS	NEG	NEG	NEG	NEG	NEG	NEG	NEG	NEG	POS	NEG	NEG	POS	NEG	NEG
UKD-1038321	SCCmec II (JH1/JH9)	CC5	POS	POS	POS	POS	POS	POS	POS	POS	POS	POS	NEG	POS	NEG	NEG	POS	NEG	NEG	NEG	NEG	NEG	POS	NEG	NEG	POS	NEG	NEG	POS	NEG	NEG
UKD-1111608	SCCmec II (JH1/JH9)	CC5	POS	POS	POS	POS	POS	POS	POS	POS	POS	POS	NEG	POS	NEG	NEG	POS	NEG	NEG	NEG	NEG	NEG	NEG	NEG	NEG	POS	NEG	NEG	POS	NEG	NEG
UKD-12753135	SCCmec II (JH1/JH9)	CC5	POS	POS	POS	POS	POS	POS	POS	POS	POS	POS	NEG	POS	NEG	NEG	POS	NEG	NEG	NEG	NEG	NEG	NEG	NEG	NEG	NEG	NEG	NEG	NEG	NEG	NEG
UKD-1312816	SCCmec II (JH1/JH9)	CC5	POS	POS	POS	POS	POS	POS	POS	POS	POS	POS	NEG	POS	NEG	NEG	POS	NEG	NEG	NEG	NEG	NEG	POS	NEG	NEG	POS	NEG	NEG	POS	NEG	NEG
UKD-1401623	SCCmec II (JH1/JH9)	CC5	POS	POS	POS	POS	POS	POS	POS	POS	POS	POS	NEG	POS	NEG	NEG	POS	NEG	NEG	NEG	NEG	NEG	POS	NEG	NEG	POS	NEG	NEG	POS	NEG	NEG
UKD-15571943	SCCmec II+czrC+ccrA/B-4	CC5	POS	POS	POS	POS	POS	POS	POS	POS	POS	POS	NEG	POS	NEG	NEG	POS	NEG	NEG	NEG	NEG	NEG	POS	NEG	NEG	NEG	NEG	NEG	NEG	NEG	NEG
UKD-15576888	SCCmec II+czrC+ccrA/B-4	CC5	POS	POS	POS	POS	POS	POS	POS	POS	POS	POS	NEG	POS	NEG	NEG	POS	NEG	NEG	NEG	NEG	NEG	POS	NEG	NEG	NEG	NEG	NEG	NEG	NEG	NEG
UKD-14576461	SCCmec II+speG+czrC+ccrA/B-4	CC5	POS	POS	POS	POS	POS	POS	POS	POS	POS	POS	NEG	POS	NEG	NEG	POS	NEG	NEG	NEG	NEG	NEG	POS	NEG	NEG	POS	NEG	NEG	POS	NEG	NEG
UKD-15581649	SCCmec II+speG+czrC+ccrA/B-4	CC5	POS	POS	POS	POS	POS	POS	POS	POS	POS	POS	NEG	POS	NEG	NEG	POS	NEG	NEG	NEG	NEG	NEG	POS	NEG	NEG	POS	NEG	NEG	POS	NEG	NEG
Prediction for CIGC345D, GenBank AHVO	SCCmec II A/B/D	CC8	POS	POS	POS	POS	POS	POS	POS	AMB	POS	POS	POS	NEG	NEG	NEG	POS	NEG	NEG	NEG	NEG	NEG	POS	NEG	NEG	NEG	NEG	NEG	NEG	NEG	NEG
UKD-12K00171	SCCmec II A/B/D	CC8	POS	POS	POS	POS	POS	POS	POS	POS	POS	POS	POS	NEG	NEG	POS	POS	NEG	NEG	NEG	NEG	NEG	POS	NEG	NEG	NEG	NEG	NEG	NEG	NEG	NEG
Prediction for JKD6008, GenBank CP002120.1	SCC [mec III+Cd] (JKD6008)	CC8 (ST239)	POS	POS	POS	POS	POS	POS	POS	AMB	POS	POS	POS	NEG	NEG	NEG	POS	NEG	NEG	NEG	POS	NEG	NEG	NEG	NEG	NEG	NEG	NEG	NEG	POS	NEG
Prediction for JKD6009, GenBank ABSA	SCC [mec III+Cd] (JKD6008)	CC8 (ST239)	NEG	POS	POS	POS	POS	POS	POS	AMB	POS	POS	POS	NEG	NEG	NEG	POS	NEG	NEG	NEG	POS	NEG	NEG	NEG	NEG	NEG	NEG	NEG	NEG	POS	NEG
Reference strain ATCC_33592	SCC [mec III+Cd] (ATCC33592)	CC8 (ST239)	POS	POS	POS	POS	POS	POS	POS	POS	POS	POS	POS	NEG	NEG	POS	POS	NEG	NEG	NEG	POS	NEG	NEG	NEG	NEG	NEG	NEG	NEG	NEG	POS	NEG
Prediction for PPUKM-775-2009, GenBank AMRE	SCC [mec III+Cd+ccrC] (PPUKM-775-2009)	CC8 (ST239)	POS	POS	POS	POS	POS	POS	POS	AMB	POS	POS	POS	NEG	NEG	NEG	POS	NEG	NEG	NEG	POS	NEG	NEG	NEG	NEG	NEG	NEG	NEG	NEG	NEG	NEG
Prediction for T0131, GenBank CP002643.1	SCC [mec III+Cd+ccrC] (T0131)	CC8 (ST239)	POS	POS	POS	POS	POS	POS	POS	AMB	POS	POS	POS	NEG	NEG	NEG	POS	NEG	NEG	NEG	POS	NEG	NEG	NEG	NEG	NEG	NEG	NEG	NEG	POS	NEG
Prediction for XN108, GenBank CP007447.1	SCC [mec III+Cd+ccrC] (XN108)	CC8 (ST239)	POS	POS	POS	POS	POS	POS	POS	AMB	POS	POS	POS	NEG	NEG	NEG	POS	NEG	NEG	NEG	POS	NEG	NEG	NEG	NEG	NEG	NEG	NEG	NEG	POS	NEG
Prediction for CN79, GenBank ANCJ	SCC [mec III+Cd+ccrC] (CN79)	CC8 (ST239)	NEG	POS	POS	POS	POS	POS	POS	AMB	POS	POS	POS	NEG	NEG	NEG	POS	NEG	NEG	NEG	POS	NEG	NEG	NEG	NEG	NEG	NEG	NEG	NEG	POS	NEG
Prediction for 16K, GenBank BABZ	SCC [mec III+Cd+ccrC] (CN79)	CC8 (ST239)	POS	POS	POS	POS	POS	POS	POS	AMB	POS	POS	POS	NEG	NEG	NEG	POS	NEG	NEG	NEG	POS	NEG	NEG	NEG	NEG	NEG	NEG	NEG	NEG	POS	NEG
UKD-0835987	SCC [mec III+Cd+ccrC] (CN79)	CC8 (ST239)	POS	POS	POS	POS	POS	POS	POS	POS	POS	POS	POS	NEG	NEG	NEG	POS	NEG	NEG	NEG	POS	NEG	NEG	NEG	NEG	NEG	NEG	NEG	NEG	POS	NEG
UKD-16581769	SCCmec III+Cd+ccrC	CC8 (ST239)	POS	POS	POS	POS	POS	POS	POS	POS	POS	POS	POS	NEG	NEG	NEG	POS	NEG	NEG	NEG	POS	NEG	NEG	NEG	NEG	NEG	NEG	NEG	NEG	POS	NEG
Prediction for Bmb9393, GenBank CP005288.1	SCCmec III+Cd/Hg+ccrC (Bmb9393)	CC8 (ST239)	POS	POS	POS	POS	POS	POS	POS	AMB	POS	POS	POS	NEG	NEG	NEG	POS	NEG	NEG	NEG	NEG	NEG	NEG	NEG	NEG	NEG	NEG	NEG	NEG	NEG	NEG
UKD-15574263	SCCmec III+Cd/Hg+ccrC (Bmb9393)	CC8 (ST239)	POS	POS	POS	POS	POS	POS	POS	POS	POS	POS	POS	NEG	NEG	AMB	POS	NEG	NEG	NEG	POS	NEG	NEG	NEG	NEG	NEG	NEG	NEG	NEG	NEG	NEG
Prediction for SK1585, GenBank AYLT	SCCmec III+ccrC+Cd/Hg (SK1585)	CC8 (ST239)	POS	POS	POS	POS	POS	POS	POS	AMB	POS	POS	NEG	NEG	NEG	AMB	POS	NEG	NEG	NEG	NEG	NEG	NEG	NEG	NEG	NEG	NEG	NEG	NEG	NEG	NEG
UKD-105861	SCCmec III+ccrC+Cd/Hg (SK1585)	CC8 (ST239)	POS	POS	POS	POS	POS	POS	POS	POS	POS	POS	POS	NEG	NEG	NEG	POS	NEG	NEG	NEG	POS	NEG	NEG	NEG	NEG	NEG	NEG	NEG	NEG	NEG	NEG
UKD-134572	SCCmec III+ccrC+Cd/Hg (SK1585)	CC8 (ST239)	POS	POS	POS	POS	POS	POS	POS	POS	POS	POS	POS	NEG	NEG	NEG	POS	NEG	NEG	NEG	POS	NEG	NEG	NEG	NEG	NEG	NEG	NEG	NEG	NEG	NEG
UKD-139143	SCCmec III+ccrC+Cd/Hg (SK1585)	CC8 (ST239)	POS	POS	POS	POS	POS	POS	POS	POS	POS	POS	POS	NEG	NEG	NEG	POS	NEG	NEG	NEG	POS	NEG	NEG	NEG	NEG	NEG	NEG	NEG	NEG	NEG	NEG
Prediction for TW20, GenBank FN433596.1	SCC [mec III+Cd/Hg+ccrC] (TW20)	CC8 (ST239)	POS	POS	POS	POS	POS	POS	POS	AMB	POS	POS	POS	NEG	NEG	NEG	POS	NEG	NEG	NEG	POS	NEG	NEG	NEG	NEG	NEG	NEG	NEG	NEG	POS	NEG
Prediction for ATCC BAA-39, GenBank AEEK	SCC [mec III+Cd/Hg+ccrC] (TW20)	CC8 (ST239)	POS	POS	POS	POS	POS	POS	POS	AMB	POS	POS	POS	NEG	NEG	NEG	POS	NEG	NEG	NEG	POS	NEG	NEG	NEG	NEG	NEG	NEG	NEG	NEG	POS	NEG

Isolate ID	SCCmec type	Clonal complex	SPECIES MARKER							REGULATORY GENES								VIRULENCE : TOX.SCHOCK.TOXIN			VIRULENCE : ENTEROTOXINS										
			Domain 1 of 23S-rRNA	glyceraldehyde 3-phosphate dehydrogenase, locus 1	katalase A	coagulase	thermostable extracellular nuclease	staphylococcal protein A	IgG-binding protein	staphylococcal accessory regulator A	histidine protein kinase, sae locus	sensor protein	accessory gene regulator alleles				haemolysin delta	toxic shock syndrome toxin 1			Entero-toxin A	Entero-toxin A, allele from strain 320E	Entero-toxin A, allele from strain N315 = Entero-toxin P	Entero-toxin B	Entero-toxin C	Entero-toxin D	Entero-toxin E	Entero-toxin H	Entero-toxin J	Entero-toxin K	Entero-toxin L
			rrnD1 (S. aureus)	gapA	katA	CoA	nuc1	spa	sbi	sarA	saeS	vraS	agrI (total)	agrII (total)	agrIII (total)	agrIV (total)	hld	tst1 (consensus)	tst1 ("human" allele)	tst1 ("bovine" allele)	entA	entA (320E)	entA (N315) / entP	entB	entC	entD	entE	entH	entJ	entK	entL
Prediction for MW2, GenBank BA000033.2	SCCmec IVa (MW2)	CC1	POS	POS	POS	POS	POS	POS	POS	AMB	POS	POS	NEG	NEG	POS	NEG	POS	NEG	NEG	NEG	POS	NEG	NEG	NEG	POS	NEG	NEG	POS	NEG	POS	POS
Reference strain MW2	SCCmec IVa (MW2)	CC1	POS	POS	POS	POS	POS	POS	POS	MW2_R27	POS	POS	NEG	NEG	POS	NEG	POS	NEG	NEG	NEG	POS	NEG	NEG	NEG	POS	NEG	NEG	POS	NEG	POS	POS
Reference strain CA05 (JCSC1968)	SCCmec IVa (MW2)	CC45	POS	POS	POS	POS	POS	POS	POS	POS	POS	POS	POS	NEG	NEG	NEG	POS	NEG	NEG	NEG	NEG	NEG	NEG	NEG	NEG	NEG	NEG	NEG	NEG	NEG	NEG
UKD-0127106	SCCmec IVa (MW2)	CC1	POS	POS	POS	POS	POS	POS	POS	POS	POS	POS	NEG	NEG	POS	NEG	POS	NEG	NEG	NEG	POS	NEG	NEG	NEG	NEG	NEG	NEG	POS	NEG	POS	NEG
UKD-0701446	SCCmec IVa (MW2)	CC1	POS	POS	POS	POS	POS	POS	POS	POS	POS	POS	NEG	NEG	POS	NEG	POS	NEG	NEG	NEG	POS	NEG	NEG	NEG	NEG	NEG	NEG	POS	NEG	POS	NEG
UKD-0947486	SCCmec IVa (MW2)	CC1	POS	POS	POS	POS	POS	POS	POS	POS	POS	POS	NEG	NEG	POS	NEG	POS	NEG	NEG	NEG	NEG	NEG	NEG	NEG	NEG	NEG	NEG	POS	NEG	NEG	NEG
UKD-1031910	SCCmec IVa (MW2)	CC1	POS	POS	POS	POS	POS	POS	POS	POS	POS	POS	NEG	NEG	POS	NEG	POS	NEG	NEG	NEG	NEG	NEG	NEG	NEG	NEG	NEG	NEG	POS	NEG	NEG	NEG
UKD-14571328	SCCmec IVa (MW2)	CC1	POS	POS	POS	POS	POS	POS	POS	POS	POS	POS	NEG	NEG	POS	NEG	POS	NEG	NEG	NEG	NEG	NEG	NEG	NEG	NEG	NEG	NEG	POS	NEG	POS	NEG
UKD-03003012	SCCmec IVa (MW2)	CC5	POS	POS	POS	POS	POS	POS	POS	POS	POS	POS	NEG	POS	NEG	NEG	POS	NEG	NEG	NEG	NEG	NEG	NEG	NEG	NEG	NEG	NEG	NEG	NEG	NEG	NEG
UKD-15582582	SCCmec IVa (MW2)	CC6	POS	POS	POS	POS	POS	POS	POS	POS	POS	POS	POS	NEG	NEG	AMB	POS	NEG	NEG	NEG	POS	NEG	NEG	NEG	NEG	NEG	NEG	NEG	NEG	NEG	NEG
UKD-16574230	SCCmec IVa (MW2)	CC6	POS	POS	POS	POS	POS	POS	POS	POS	POS	POS	POS	NEG	NEG	AMB	POS	NEG	NEG	NEG	POS	NEG	NEG	NEG	NEG	NEG	NEG	NEG	NEG	NEG	NEG
UKD-16582560	SCCmec IVa (MW2)	CC6	POS	POS	POS	POS	POS	POS	POS	POS	POS	POS	POS	NEG	NEG	NEG	POS	NEG	NEG	NEG	POS	NEG	NEG	NEG	NEG	NEG	NEG	NEG	NEG	NEG	NEG
UKD-0802855	SCCmec IVa (MW2)	CC7	POS	POS	POS	POS	POS	POS	POS	POS	POS	POS	POS	NEG	NEG	NEG	POS	NEG	NEG	NEG	NEG	NEG	POS	NEG	NEG	NEG	NEG	NEG	NEG	NEG	NEG
KHDN-10405258	SCCmec IVa (MW2)	CC8	POS	POS	POS	POS	POS	POS	POS	POS	POS	POS	POS	NEG	NEG	AMB	POS	NEG	NEG	NEG	NEG	NEG	NEG	NEG	NEG	NEG	NEG	NEG	NEG	POS	NEG
UKD-15573497	SCCmec IVa (MW2)	CC22	POS	POS	POS	POS	POS	POS	POS	POS	POS	POS	POS	NEG	NEG	POS	POS	POS	POS	NEG	NEG	NEG	NEG	NEG	POS	AMB	NEG	NEG	NEG	NEG	POS
UKD-0634729	SCCmec IVa (MW2)	CC30	POS	POS	POS	POS	POS	POS	POS	POS	POS	POS	NEG	NEG	POS	NEG	POS	NEG	NEG	NEG	NEG	NEG	NEG	NEG	NEG	NEG	NEG	NEG	NEG	NEG	NEG
UKD-1119771	SCCmec IVa (MW2)	CC30	POS	POS	POS	POS	POS	POS	POS	POS	POS	POS	NEG	NEG	POS	NEG	POS	NEG	NEG	NEG	NEG	NEG	NEG	NEG	NEG	NEG	NEG	NEG	NEG	NEG	NEG
KHDN-10411458	SCCmec IVa (MW2)	CC30	POS	POS	POS	POS	POS	POS	POS	POS	POS	POS	NEG	NEG	POS	NEG	POS	NEG	NEG	NEG	NEG	NEG	NEG	NEG	NEG	NEG	NEG	NEG	NEG	NEG	NEG
KHDN-398609	SCCmec IVa (MW2)	CC30	POS	POS	POS	POS	POS	POS	POS	POS	POS	POS	NEG	NEG	POS	NEG	POS	NEG	NEG	NEG	NEG	NEG	NEG	NEG	NEG	NEG	NEG	NEG	NEG	NEG	NEG
KHDN-01081998	SCCmec IVa (MW2)	CC30	POS	POS	POS	POS	POS	POS	POS	POS	POS	POS	NEG	NEG	POS	NEG	POS	NEG	NEG	NEG	NEG	NEG	NEG	NEG	NEG	NEG	NEG	NEG	NEG	NEG	NEG
KHDN-10399223	SCCmec IVa (MW2)	CC30	POS	POS	POS	POS	POS	POS	POS	POS	POS	POS	NEG	NEG	POS	NEG	POS	NEG	NEG	NEG	NEG	NEG	NEG	NEG	NEG	NEG	NEG	NEG	NEG	NEG	NEG
KHDN-W261158	SCCmec IVa (MW2)	CC30	POS	POS	POS	POS	POS	POS	POS	POS	POS	POS	NEG	NEG	POS	NEG	POS	NEG	NEG	NEG	NEG	NEG	NEG	NEG	NEG	NEG	NEG	NEG	NEG	NEG	NEG
UKD-0002046	SCCmec IVa (MW2)	CC45 [agr I]	POS	POS	POS	POS	POS	POS	POS	POS	POS	POS	POS	NEG	NEG	NEG	POS	NEG	NEG	NEG	NEG	NEG	NEG	NEG	NEG	NEG	NEG	NEG	NEG	NEG	NEG
UKD-0029618	SCCmec IVa (MW2)	CC45 [agr I]	POS	POS	POS	POS	POS	POS	POS	POS	POS	POS	POS	NEG	NEG	NEG	POS	NEG	NEG	NEG	NEG	NEG	NEG	NEG	NEG	NEG	NEG	NEG	NEG	NEG	NEG
UKD-0043085	SCCmec IVa (MW2)	CC45 [agr I]	POS	POS	POS	POS	POS	POS	POS	POS	POS	POS	POS	NEG	NEG	NEG	POS	NEG	NEG	NEG	NEG	NEG	NEG	NEG	NEG	NEG	NEG	NEG	NEG	NEG	NEG
UKD-0047053	SCCmec IVa (MW2)	CC45 [agr I]	POS	POS	POS	POS	POS	AMB	POS	POS	POS	POS	AMB	NEG	NEG	NEG	POS	NEG	NEG	NEG	NEG	NEG	NEG	NEG	NEG	NEG	NEG	NEG	NEG	NEG	NEG
UKD-0047429	SCCmec IVa (MW2)	CC45 [agr I]	POS	POS	POS	POS	POS	POS	POS	POS	POS	POS	POS	NEG	NEG	NEG	POS	NEG	NEG	NEG	NEG	NEG	NEG	NEG	NEG	NEG	NEG	NEG	NEG	NEG	NEG
UKD-0112465	SCCmec IVa (MW2)	CC45 [agr I]	POS	POS	POS	POS	POS	POS	POS	POS	POS	POS	POS	NEG	NEG	NEG	POS	NEG	NEG	NEG	NEG	NEG	NEG	NEG	NEG	NEG	NEG	NEG	NEG	NEG	NEG
UKD-0103483	SCCmec IVa (MW2)	CC45 [agr I]	POS	POS	POS	POS	POS	AMB	POS	POS	POS	POS	NEG	NEG	NEG	NEG	POS	NEG	NEG	NEG	NEG	NEG	NEG	NEG	NEG	NEG	NEG	NEG	NEG	NEG	NEG
UKD-0305014	SCCmec IVa (MW2)	CC45 [agr I]	POS	POS	POS	POS	POS	POS	POS	POS	POS	POS	POS	NEG	NEG	NEG	POS	NEG	NEG	NEG	NEG	NEG	NEG	NEG	NEG	NEG	NEG	NEG	NEG	NEG	NEG
UKD-0401119	SCCmec IVa (MW2)	CC45 [agr I]	POS	POS	POS	POS	POS	POS	POS	POS	POS	POS	POS	NEG	NEG	NEG	POS	NEG	NEG	NEG	NEG	NEG	NEG	NEG	NEG	NEG	NEG	NEG	NEG	NEG	NEG
UKD-0537969	SCCmec IVa (MW2)	CC45 [agr I]	POS	POS	POS	POS	POS	POS	POS	POS	POS	POS	POS	NEG	NEG	NEG	POS	NEG	NEG	NEG	NEG	NEG	NEG	NEG	NEG	NEG	NEG	NEG	NEG	NEG	NEG
UKD-0703373	SCCmec IVa (MW2)	CC45 [agr I]	POS	POS	POS	POS	POS	POS	POS	POS	POS	POS	POS	NEG	NEG	NEG	POS	NEG	NEG	NEG	NEG	NEG	NEG	NEG	NEG	NEG	NEG	NEG	NEG	NEG	NEG
UKD-0813653	SCCmec IVa (MW2)	CC45 [agr I]	POS	POS	POS	POS	POS	POS	POS	POS	POS	POS	POS	NEG	NEG	NEG	POS	NEG	NEG	NEG	NEG	NEG	NEG	NEG	NEG	NEG	NEG	NEG	NEG	NEG	NEG
UKD-0839175	SCCmec IVa (MW2)	CC45 [agr I]	POS	POS	POS	POS	POS	POS	POS	POS	POS	POS	POS	NEG	NEG	NEG	POS	NEG	AMB	NEG	NEG	NEG	NEG	NEG	NEG	NEG	NEG	NEG	NEG	NEG	NEG
UKD-1108422	SCCmec IVa (MW2)	CC45 [agr I]	POS	POS	POS	POS	POS	POS	POS	POS	POS	POS	POS	NEG	NEG	NEG	POS	NEG	NEG	NEG	NEG	NEG	NEG	NEG	NEG	NEG	NEG	NEG	NEG	NEG	NEG
UKD-1380645	SCCmec IVa (MW2)	CC45 [agr I]	POS	POS	POS	POS	POS	POS	POS	POS	POS	POS	POS	NEG	NEG	NEG	POS	NEG	NEG	NEG	NEG	NEG	NEG	NEG	POS	NEG	NEG	NEG	NEG	NEG	POS
UKD-148016	SCCmec IVa (MW2)	CC45 [agr I]	POS	POS	POS	POS	POS	POS	POS	POS	POS	POS	POS	NEG	NEG	NEG	POS	NEG	NEG	NEG	NEG	NEG	NEG	NEG	POS	NEG	NEG	NEG	NEG	NEG	POS
UKD-16571773	SCCmec IVa (MW2)	CC45 [agr I]	POS	POS	POS	POS	POS	POS	POS	POS	POS	POS	POS	NEG	NEG	NEG	POS	NEG	NEG	NEG	NEG	NEG	NEG	AMB	POS	NEG	NEG	NEG	NEG	NEG	POS
KHDN-10407738	SCCmec IVa (MW2)	CC45 [ST617]	POS	POS	POS	POS	POS	POS	POS	POS	POS	POS	NEG	NEG	NEG	POS	POS	NEG	NEG	NEG	NEG	NEG	NEG	NEG	NEG	NEG	NEG	NEG	NEG	NEG	NEG
UKD-0853670	SCCmec IVa (MW2)	CC88	POS	POS	POS	POS	POS	POS	POS	POS	POS	POS	NEG	NEG	POS	NEG	POS	NEG	NEG	NEG	NEG	NEG	NEG	AMB	POS	NEG	NEG	NEG	NEG	NEG	POS
UKD-1417352	SCCmec IVa (MW2)	CC88	POS	POS	POS	POS	POS	POS	POS	POS	POS	POS	NEG	NEG	POS	NEG	POS	NEG	NEG	NEG	NEG	NEG	POS	NEG	NEG	NEG	NEG	NEG	NEG	NEG	NEG
UKD-15571283	SCCmec IVa (MW2)	CC88	POS	POS	POS	POS	POS	POS	POS	POS	POS	POS	NEG	NEG	POS	NEG	POS	NEG	NEG	NEG	NEG	NEG	POS	NEG	NEG	POS	NEG	NEG	NEG	NEG	NEG
KHDN-10406719	SCCmec IVa (MW2)	ST93	POS	POS	POS	POS	POS	POS	POS	POS	POS	POS	NEG	NEG	POS	NEG	POS	NEG	NEG	NEG	NEG	NEG	NEG	NEG	NEG	NEG	NEG	NEG	NEG	NEG	NEG
KHDN-0060294	SCCmec IVa (MW2)	ST93	POS	POS	POS	POS	POS	POS	AMB	POS	POS	POS	NEG	NEG	POS	NEG	POS	NEG	NEG	NEG	NEG	NEG	NEG	NEG	NEG	NEG	NEG	NEG	NEG	NEG	NEG
UKD-0734190	SCCmec IVa (MW2)	CC97	POS	POS	POS	POS	POS	POS	POS	POS	POS	POS	POS	NEG	NEG	NEG	POS	NEG	NEG	NEG	NEG	NEG	NEG	NEG	NEG	NEG	NEG	NEG	NEG	NEG	NEG
UKD-13006983	SCCmec IVa (MW2)	CC97	POS	POS	POS	POS	POS	POS	POS	POS	POS	POS	POS	NEG	NEG	POS	POS	NEG	NEG	NEG	NEG	NEG	NEG	NEG	NEG	NEG	NEG	NEG	NEG	NEG	NEG
Prediction for H131520133	SCCmec IVa (H131520133)	CC152	POS	POS	POS	POS	POS	POS	POS	POS	POS	POS	AMB	NEG	NEG	AMB	POS	NEG	NEG	NEG	NEG	NEG	NEG	NEG	NEG	NEG	NEG	NEG	NEG	NEG	NEG
KHDN-325892	SCCmec IVa (H131520133)	CC30	POS	POS	POS	POS	POS	POS	POS	POS	POS	POS	NEG	NEG	POS	NEG	POS	NEG	NEG	NEG	NEG	NEG	NEG	NEG	NEG	NEG	NEG	NEG	NEG	NEG	NEG
UKD-15580207	SCCmec IVa (H131520133)	CC88	POS	POS	POS	POS	POS	POS	POS	POS	POS	POS	NEG	NEG	POS	NEG	POS	NEG	NEG	NEG	NEG	NEG	POS	NEG	NEG	NEG	NEG	NEG	NEG	POS	NEG
UKD-0521159	SCCmec Iva	CC8	POS	POS	POS	POS	POS	POS	POS	POS	POS	POS	POS	NEG	NEG	NEG	POS	NEG	NEG	NEG	NEG	NEG	NEG	POS	NEG	NEG	NEG	NEG	NEG	POS	NEG
Prediction for CMFT503 : HF569113.1	SCCmec IVa (CMFT503)																														
Prediction for 21343, GenBank AHKV	SCCmec IVa (CMFT503)	CC88	POS	POS	POS	POS	POS	POS	POS	AMB	POS	POS	NEG	NEG	POS	NEG	POS	NEG	NEG	NEG	NEG	NEG	NEG	NEG	NEG	NEG	NEG	NEG	NEG	NEG	NEG
UKD-1420740	SCCmec IVa (CMFT503)	CC22	POS	POS	POS	POS	POS	POS	POS	POS	POS	POS	POS	NEG	NEG	POS	POS	NEG	NEG	NEG	NEG	NEG	NEG	NEG	NEG	AMB	NEG	NEG	NEG	NEG	NEG
UKD-15575124	SCCmec IVa (CMFT503)	CC22	POS	POS	POS	POS	POS	POS	POS	POS	POS	POS	POS	NEG	NEG	AMB	POS	POS	POS	NEG	NEG	NEG	NEG	NEG	NEG	NEG	NEG	NEG	NEG	NEG	NEG
UKD-16584947	SCCmec IVa (CMFT503)	CC22	POS	POS	POS	POS	POS	POS	POS	POS	POS	POS	POS	NEG	NEG	NEG	POS	POS	POS	NEG	NEG	NEG	NEG	NEG	NEG	NEG	NEG	NEG	NEG	NEG	NEG
UKD-16575077	SCCmec IVa (CMFT503)	CC88	POS	POS	POS	POS	POS	POS	POS	POS	POS	POS	NEG	NEG	POS	NEG	POS	NEG	NEG	NEG	NEG	NEG	NEG	NEG	NEG	NEG	NEG	NEG	NEG	NEG	NEG
Prediction for TCH1516, GenBank CP000730.1	SCCmec IVa+ACME1+Cu (USA300)	CC8	POS	POS	POS	POS	POS	POS	POS	AMB	POS	POS	POS	NEG	NEG	NEG	POS	NEG	NEG	NEG	NEG	NEG	NEG	NEG	NEG	NEG	NEG	NEG	NEG	POS	NEG
Reference strain USA300-TCH1516	SCCmec IVa+ACME1+Cu (USA300)	CC8	POS	POS	POS	POS	POS	POS	POS	A300-TCH1	POS	POS	POS	NEG	NEG	AMB	POS	NEG	NEG	NEG	NEG	NEG	NEG	NEG	NEG	NEG	NEG	NEG	NEG	POS	NEG
Prediction for FPR3757, GenBank CP000255.1	SCCmec IVa+ACME1+Cu (USA300)	CC8	POS	POS	POS	POS	POS	POS	POS	AMB	POS	POS	POS	NEG	NEG	NEG	POS	NEG	NEG	NEG	NEG	NEG	NEG	NEG	NEG	NEG	NEG	NEG	NEG	POS	NEG
UKD-0516386	SCCmec IVa+ACME1+Cu (USA300)	CC8	POS	POS	POS	POS	POS	POS	POS	POS	POS	POS	POS	NEG	NEG	NEG	POS	NEG	NEG	NEG	NEG	NEG	NEG	NEG	NEG	NEG	NEG	NEG	NEG	POS	NEG
UKD-0815773	SCCmec IVa+ACME1+Cu (USA300)	CC8	POS	POS	POS	POS	POS	POS	POS	POS	POS	POS	POS	NEG	NEG	AMB	POS	NEG	NEG	NEG	NEG	NEG	NEG	NEG	NEG	NEG	NEG	NEG	NEG	POS	NEG
UKD-1414654	SCCmec IVa+ACME1+Cu (USA300)	CC8	POS	POS	POS	POS	POS	POS	POS	POS	POS	POS	POS	NEG	NEG	AMB	POS	NEG	NEG	NEG	NEG	NEG	NEG	NEG	NEG	NEG	NEG	NEG	NEG	POS	NEG
UKD-0408036	SCCmec IVa+ACME2	CC45 [agr I]	POS	POS	POS	POS	POS	POS	POS	POS	POS	POS	POS	NEG	NEG	NEG	POS	NEG	NEG	NEG	NEG	NEG	NEG	NEG	NEG	NEG	NEG	NEG	NEG	NEG	NEG
UKD-0044035	SCCmec IVa+ccrC (Hannover)	CC8	POS	POS	POS	POS	POS	POS	POS	POS	POS	POS	POS	NEG	NEG	NEG	POS	NEG	NEG	NEG	POS	NEG	NEG	POS	NEG	NEG	NEG	NEG	NEG	POS	NEG
UKD-0136287	SCCmec IVa+ccrC (Hannover)	CC8	POS	POS	POS	POS	POS	POS	POS	POS	POS	POS	POS	NEG	NEG	AMB	POS	NEG	NEG	NEG	POS	NEG	NEG	POS	NEG	NEG	NEG	NEG	NEG	POS	NEG

Isolate ID	SCCmec type	Clonal complex	SPECIES MARKER							REGULATORY GENES									VIRULENCE : TOX.SCHOCK.TOXIN			VIRULENCE : ENTEROTOXINS										
			Domain 1 of 23S-rRNA	glyceraldehyde 3-phosphate dehydrogenase, locus 1	katalase A	coagulase	thermostable extracellular nuclease	staphylococcal protein A	IgG-binding protein	staphylococcal accessory regulator A	histidine protein kinase, sae locus	sensor protein	accessory gene regulator alleles				haemolysin delta	toxic shock syndrome toxin 1			Entero-toxin A	Entero-toxin A, allele from strain 320E	Entero-toxin A, allele from strain N315 = Entero-toxin P	Entero-toxin B	Entero-toxin C	Entero-toxin D	Entero-toxin E	Entero-toxin H	Entero-toxin J	Entero-toxin K	Entero-toxin L
			rrnD1 (S. aureus)	gapA	katA	CoA	nuc1	spa	sbi	sarA	saeS	vraS	agrI (total)	agrII (total)	agrIII (total)	agrIV (total)	hld	tst1 (consensus)	tst1 ("human" allele)	tst1 ("bovine" allele)	entA	entA (320E)	entA (N315) / entP	entB	entC	entD	entE	entH	entJ	entK	entL
Prediction for JCSC1978, GenBank AB063173.1	*SCCmec IVb/d/i (JCSC1978/6668/4469)*																														
**Reference strain JCSC1978 (8/6-3P)**	**SCCmec IVb/d/i (JCSC1978/6668/4469)**	**CC8**	**POS**	**POS**	**POS**	**POS**	**POS**	**POS**	**POS**	**POS**	**POS**	**POS**	**POS**	**NEG**	**NEG**	**AMB**	**POS**	**NEG**	**NEG**	**NEG**	**NEG**	**NEG**	**NEG**	**NEG**	**NEG**	**POS**	**NEG**	**NEG**	**POS**	**POS**	**NEG**
Prediction for JCSC6668, GenBank AB425823.1	*SCCmec IVb/d/i (JCSC1978/6668/4469)*																														
**Reference strain JCSC4469**	**SCCmec IVb/d/i (JCSC1978/6668/4469)**	**CC5**	**POS**	**POS**	**POS**	**POS**	**POS**	**POS**	**POS**	**POS**	**POS**	**POS**	**NEG**	**POS**	**NEG**	**NEG**	**POS**	**NEG**	**NEG**	**NEG**	**NEG**	**NEG**	**POS**	**NEG**	**NEG**	**NEG**	**NEG**	**NEG**	**NEG**	**NEG**	**NEG**
**UKD-1103429**	SCCmec IVb/d/i (JCSC1978/6668/4469)	CC7	POS	POS	POS	POS	POS	POS	POS	POS	POS	POS	POS	NEG	NEG	AMB	POS	NEG	NEG	NEG	NEG	NEG	POS	NEG	NEG	NEG	NEG	NEG	NEG	NEG	NEG
**UKD-1306141**	SCCmec IVb/d/i (JCSC1978/6668/4469)	CC7	POS	POS	POS	POS	POS	POS	POS	POS	POS	POS	POS	NEG	NEG	AMB	POS	NEG	NEG	NEG	NEG	NEG	POS	NEG	NEG	NEG	NEG	NEG	NEG	NEG	NEG
**UKD-0500089**	SCCmec IVb/d/i (JCSC1978/6668/4469)	CC5	POS	POS	POS	POS	POS	POS	POS	POS	POS	POS	NEG	POS	NEG	NEG	POS	NEG	NEG	NEG	NEG	NEG	POS	NEG	NEG	POS	NEG	NEG	POS	NEG	NEG
**UKD-0501416**	SCCmec IVb/d/i (JCSC1978/6668/4469)	CC5	POS	POS	POS	POS	POS	POS	POS	POS	POS	POS	NEG	POS	NEG	NEG	POS	NEG	NEG	NEG	NEG	NEG	POS	NEG	NEG	POS	NEG	NEG	POS	NEG	NEG
**UKD-0934574**	SCCmec IVb/d/i (JCSC1978/6668/4469)	CC5	POS	POS	POS	POS	POS	POS	POS	POS	POS	POS	NEG	POS	NEG	NEG	POS	NEG	NEG	NEG	NEG	NEG	POS	NEG	NEG	POS	NEG	NEG	POS	NEG	NEG
**UKD-0746779**	SCCmec IVb/d/i (JCSC1978/6668/4469)	CC45 [agr I]	POS	POS	POS	POS	POS	POS	POS	POS	POS	POS	POS	NEG	NEG	NEG	POS	NEG	NEG	NEG	NEG	NEG	NEG	NEG	NEG	NEG	NEG	NEG	NEG	NEG	NEG
**UKD-0812518**	SCCmec IVb/d/i (JCSC1978/6668/4469)	CC45 [agr I]	POS	POS	POS	POS	POS	POS	POS	POS	POS	POS	POS	NEG	NEG	NEG	POS	NEG	NEG	NEG	NEG	NEG	NEG	NEG	NEG	NEG	NEG	NEG	NEG	NEG	NEG
Prediction for 21209, GenBank AGRP	*SCCmec IVb/d/i (21209)*	*CC8*	*POS*	*POS*	*POS*	*POS*	*POS*	*POS*	*POS*	*AMB*	*POS*	*POS*	*POS*	*NEG*	*NEG*	*NEG*	*POS*	*NEG*	*NEG*	*NEG*	*POS*	*NEG*	*NEG*	*POS*	*NEG*	*NEG*	*NEG*	*NEG*	*NEG*	*POS*	*NEG*
**UKD-0233579**	SCCmec IVb/d/i (21209)	CC8	POS	POS	POS	POS	POS	POS	POS	POS	POS	POS	POS	NEG	NEG	NEG	POS	AMB	NEG	NEG	POS	NEG	NEG	POS	NEG	NEG	NEG	NEG	NEG	POS	NEG
**UKD-0504920**	SCCmec IVb/d/i (21209)	CC8	POS	POS	POS	POS	POS	POS	POS	POS	POS	POS	POS	NEG	NEG	NEG	POS	NEG	NEG	NEG	POS	NEG	NEG	POS	NEG	NEG	NEG	NEG	NEG	POS	NEG
**UKD-0725999**	SCCmecIV b/d/i+Cu	CC30	POS	POS	POS	POS	POS	POS	POS	POS	POS	POS	NEG	NEG	POS	NEG	POS	NEG	NEG	NEG	NEG	NEG	NEG	NEG	NEG	NEG	NEG	NEG	NEG	NEG	NEG
Prediction for IS-105, GenBank AHLR	*SCCmec IVc (IS-105)*	*CC22*	*POS*	*POS*	*POS*	*POS*	*POS*	*POS*	*POS*	*AMB*	*POS*	*POS*	*POS*	*NEG*	*NEG*	*NEG*	*POS*	*NEG*	*NEG*	*NEG*	*NEG*	*NEG*	*NEG*	*NEG*	*NEG*	*NEG*	*NEG*	*NEG*	*NEG*	*NEG*	*NEG*
**UKD-0402421**	SCCmec IVc (IS-105), merA/B+	CC5	POS	POS	POS	POS	POS	POS	POS	POS	POS	POS	NEG	POS	NEG	NEG	POS	NEG	NEG	NEG	NEG	NEG	POS	POS	NEG	POS	NEG	NEG	POS	NEG	NEG
**UKD-0731319**	SCCmec IVc (IS-105), merA/B+	CC5	POS	POS	POS	POS	POS	POS	POS	POS	POS	POS	NEG	POS	NEG	NEG	POS	NEG	NEG	NEG	NEG	NEG	POS	POS	NEG	POS	NEG	NEG	POS	NEG	NEG
**UKD-0733137**	SCCmec IVc (IS-105)	CC8	POS	POS	POS	POS	POS	POS	POS	POS	POS	POS	POS	NEG	NEG	NEG	POS	NEG	NEG	NEG	POS	NEG	NEG	NEG	NEG	POS	NEG	NEG	POS	NEG	NEG
**UKD-0305690**	SCCmec IVc (IS-105)	CC22	POS	POS	POS	POS	POS	POS	POS	POS	POS	POS	POS	NEG	NEG	NEG	POS	NEG	NEG	NEG	NEG	NEG	NEG	NEG	NEG	NEG	NEG	NEG	NEG	NEG	NEG
**UKD-1424706**	SCCmec IVc (IS-105)	CC22	POS	POS	POS	POS	POS	POS	POS	POS	POS	POS	POS	NEG	NEG	AMB	POS	NEG	NEG	NEG	NEG	NEG	NEG	NEG	NEG	NEG	NEG	NEG	NEG	NEG	NEG
Prediction for TCH60, GenBank CP002110.1	*SCCmec IVc (TCH60)*	*CC30*	*POS*	*POS*	*POS*	*POS*	*POS*	*POS*	*POS*	*AMB*	*POS*	*POS*	*NEG*	*NEG*	*POS*	*NEG*	*POS*	*NEG*	*NEG*	*NEG*	*NEG*	*NEG*	*NEG*	*NEG*	*NEG*	*NEG*	*NEG*	*NEG*	*NEG*	*NEG*	*NEG*
**Reference strain JCSC4788**	**SCCmec IVc (TCH60)**	**CC8**	**POS**	**POS**	**POS**	**POS**	**POS**	**POS**	**POS**	**POS**	**POS**	**POS**	**POS**	**NEG**	**NEG**	**NEG**	**POS**	**NEG**	**NEG**	**NEG**	**NEG**	**NEG**	**NEG**	**NEG**	**NEG**	**NEG**	**NEG**	**NEG**	**NEG**	**NEG**	**NEG**
**UKD-1318318**	SCCmec IVc (TCH60)	CC5	POS	POS	POS	POS	POS	POS	POS	POS	POS	POS	NEG	POS	NEG	NEG	POS	NEG	NEG	NEG	NEG	NEG	POS	NEG	NEG	POS	NEG	NEG	POS	NEG	NEG
**UKD-14580050**	SCCmec IVc (TCH60)	CC5	POS	POS	POS	POS	POS	POS	POS	POS	POS	POS	NEG	POS	NEG	NEG	POS	NEG	NEG	NEG	NEG	NEG	POS	NEG	NEG	POS	NEG	NEG	POS	NEG	NEG
**UKD-08F01314**	SCCmec IVc (TCH60)	CC8	POS	POS	POS	POS	NEG	POS	POS	POS	POS	POS	POS	NEG	NEG	NEG	POS	NEG	NEG	NEG	NEG	NEG	NEG	NEG	NEG	NEG	NEG	NEG	NEG	NEG	NEG
**KHDN-50361518**	SCCmec IVc (TCH60)	CC8	POS	POS	POS	POS	POS	POS	POS	POS	POS	POS	POS	NEG	NEG	AMB	POS	NEG	NEG	NEG	POS	NEG	NEG	NEG	NEG	NEG	NEG	NEG	NEG	NEG	NEG
**UKD-0307912**	SCCmec IVc (TCH60)	CC22	POS	POS	POS	POS	POS	POS	POS	POS	POS	POS	POS	NEG	NEG	NEG	POS	NEG	NEG	NEG	NEG	NEG	NEG	NEG	NEG	NEG	NEG	NEG	NEG	NEG	NEG
**UKD-0220390**	SCCmec IVc (TCH60)	CC45 [agr I]	POS	POS	POS	POS	POS	POS	POS	POS	POS	POS	AMB	NEG	NEG	NEG	POS	NEG	NEG	NEG	NEG	NEG	NEG	NEG	POS	NEG	NEG	NEG	NEG	NEG	POS
**UKD-0311106**	SCCmec IVc (TCH60)	CC45 [agr I]	POS	POS	POS	POS	POS	POS	POS	POS	POS	POS	POS	NEG	NEG	NEG	POS	NEG	NEG	NEG	NEG	NEG	NEG	NEG	POS	NEG	NEG	NEG	NEG	NEG	POS
**UKD-0719855**	SCCmec IVc (TCH60)	CC45 [agr I]	POS	POS	POS	POS	POS	POS	POS	POS	POS	POS	POS	NEG	NEG	NEG	POS	NEG	NEG	NEG	NEG	NEG	NEG	NEG	POS	NEG	NEG	NEG	NEG	NEG	POS
**UKD-0422079**	SCCmec IVc (TCH60)	CC45 [agr I]	POS	POS	POS	POS	POS	POS	POS	POS	POS	POS	POS	NEG	NEG	NEG	POS	NEG	NEG	NEG	NEG	NEG	NEG	NEG	POS	NEG	NEG	NEG	NEG	NEG	POS
**UKD-0409235**	SCCmec IVc (TCH60)	CC80	POS	POS	POS	POS	POS	POS	POS	POS	POS	POS	NEG	NEG	POS	NEG	POS	NEG	NEG	NEG	NEG	NEG	NEG	NEG	NEG	NEG	NEG	NEG	NEG	NEG	NEG
**UKD-0409807**	SCCmec IVc (TCH60)	CC80	POS	POS	POS	POS	POS	POS	POS	POS	POS	POS	NEG	NEG	POS	NEG	POS	NEG	NEG	NEG	NEG	NEG	NEG	NEG	NEG	NEG	NEG	NEG	NEG	NEG	NEG
**UKD-0416073**	SCCmec IVc (TCH60)	CC80	POS	POS	POS	POS	POS	POS	POS	POS	POS	POS	NEG	NEG	POS	NEG	POS	NEG	NEG	NEG	NEG	NEG	NEG	NEG	NEG	NEG	NEG	NEG	NEG	NEG	NEG
**UKD-0431728**	SCCmec IVc (TCH60)	CC80	POS	POS	POS	POS	POS	POS	POS	POS	POS	POS	NEG	NEG	POS	NEG	POS	NEG	NEG	NEG	NEG	NEG	NEG	NEG	NEG	NEG	NEG	NEG	NEG	NEG	NEG
**UKD-1001508**	SCCmec IVc (TCH60)	CC80	POS	POS	POS	POS	POS	POS	POS	POS	POS	POS	NEG	NEG	POS	NEG	POS	NEG	NEG	NEG	NEG	NEG	NEG	NEG	NEG	NEG	NEG	NEG	NEG	NEG	NEG
**UKD-1010412**	SCCmec IVc (TCH60)	CC80	POS	POS	POS	POS	POS	POS	POS	POS	POS	POS	NEG	NEG	POS	NEG	POS	NEG	NEG	NEG	NEG	NEG	NEG	NEG	NEG	AMB	NEG	NEG	NEG	NEG	NEG
**UKD-12753005**	SCCmec IVc (TCH60)	CC80	POS	POS	POS	POS	POS	POS	POS	POS	POS	POS	NEG	NEG	POS	NEG	POS	NEG	NEG	NEG	NEG	NEG	NEG	NEG	NEG	NEG	NEG	NEG	NEG	NEG	NEG
**UKD-15574809**	SCCmec IVc (TCH60)	CC80	POS	POS	POS	POS	POS	POS	POS	POS	POS	POS	NEG	NEG	POS	NEG	POS	NEG	NEG	NEG	NEG	NEG	NEG	NEG	NEG	NEG	NEG	NEG	NEG	NEG	NEG
**KHDN-16120291**	SCCmec IVc (TCH60)	CC88	POS	POS	POS	POS	POS	POS	POS	POS	POS	POS	NEG	NEG	POS	NEG	POS	NEG	NEG	NEG	NEG	NEG	POS	NEG	NEG	NEG	NEG	NEG	NEG	NEG	NEG
Prediction for WW2703/97, GenBank ACSW	*SCCmec IVc (WW2703/97)*	*CC30*	*POS*	*POS*	*POS*	*POS*	*POS*	*POS*	*POS*	*AMB*	*POS*	*POS*	*NEG*	*NEG*	*POS*	*NEG*	*POS*	*NEG*	*NEG*	*NEG*	*NEG*	*NEG*	*NEG*	*NEG*	*NEG*	*NEG*	*NEG*	*NEG*	*NEG*	*NEG*	*NEG*
**UKD-14580088**	SCCmec IVc (WW2703/97)	CC398	POS	POS	POS	POS	POS	POS	POS	POS	POS	POS	POS	NEG	NEG	NEG	POS	NEG	NEG	NEG	NEG	NEG	NEG	NEG	NEG	NEG	NEG	NEG	NEG	NEG	NEG
Prediction for SA_ST125_MupR, GenBank ASTH	*SCCmec IVc+speG+ccrAB4 (SA_ST125)*	*CC5*	*POS*	*POS*	*POS*	*POS*	*POS*	*POS*	*POS*	*AMB*	*POS*	*POS*	*NEG*	*POS*	*NEG*	*NEG*	*POS*	*NEG*	*NEG*	*NEG*	*NEG*	*NEG*	*NEG*	*NEG*	*NEG*	*NEG*	*NEG*	*NEG*	*NEG*	*NEG*	*NEG*
**UKD-0439909**	SCCmec IVc+speG+ccrAB4 (SA_ST125)	CC5	POS	POS	POS	POS	POS	POS	POS	POS	POS	POS	NEG	POS	NEG	NEG	POS	NEG	NEG	NEG	NEG	NEG	NEG	NEG	NEG	NEG	NEG	NEG	NEG	NEG	NEG
Prediction for MRSA177, GenBank AECP	*SCCmec IVc+Cu/Hg (MRSA177)*		*POS*	*POS*	*POS*	*POS*	*POS*	*POS*	*POS*	*AMB*	*POS*	*POS*	*POS*	*NEG*	*NEG*	*NEG*	*POS*	*NEG*	*NEG*	*NEG*	*NEG*	*NEG*	*NEG*	*NEG*	*NEG*	*NEG*	*NEG*	*NEG*	*NEG*	*POS*	*NEG*
**UKD-1279734**	SCCmec IVc+Cu/Hg (MRSA177)	CC8	POS	POS	POS	POS	POS	POS	POS	POS	POS	POS	POS	NEG	NEG	POS	POS	NEG	NEG	NEG	NEG	NEG	NEG	NEG	NEG	NEG	NEG	NEG	NEG	NEG	NEG
**UKD-14580899**	SCCmec IVc+Cu/Hg (MRSA177)	CC8	POS	POS	POS	POS	POS	POS	POS	POS	POS	POS	POS	NEG	NEG	POS	POS	NEG	NEG	NEG	NEG	NEG	NEG	NEG	NEG	NEG	NEG	NEG	NEG	POS	NEG
**UKD-16002228**	SCCmec IVc+Cu/Hg (MRSA177)	CC8	POS	POS	POS	POS	POS	POS	POS	POS	POS	POS	POS	NEG	NEG	AMB	POS	NEG	NEG	NEG	NEG	NEG	NEG	NEG	NEG	NEG	NEG	NEG	NEG	NEG	NEG
Prediction for HO 5096 0412, GenBank HE681097.1	*SCCmec IVh/j (HO50960412/JCSC6670)*	*CC22*	*POS*	*POS*	*POS*	*POS*	*POS*	*POS*	*POS*	*POS*	*POS*	*POS*	*POS*	*NEG*	*NEG*	*NEG*	*POS*	*NEG*	*NEG*	*NEG*	*NEG*	*NEG*	*NEG*	*NEG*	*NEG*	*NEG*	*NEG*	*NEG*	*NEG*	*NEG*	*NEG*
Prediction for JCSC6670, GenBank AB425824.1	*SCCmec IVh/j (HO50960412/JCSC6670)*																														
**Reference strain Dublin_DSH_E1749**	**SCCmec IVh/j (HO50960412/JCSC6670)**		**POS**	**POS**	**POS**	**POS**	**POS**	**POS**	**POS**	**POS**	**POS**	**POS**	**POS**	**NEG**	**NEG**	**NEG**	**POS**	**NEG**	**NEG**	**NEG**	**NEG**	**NEG**	**NEG**	**AMB**	**POS**	**NEG**	**NEG**	**NEG**	**NEG**	**NEG**	**POS**
**UKD-14576248**	SCCmec IVh/j (HO50960412/JCSC6670)	CC8	POS	POS	POS	POS	POS	POS	POS	POS	POS	POS	POS	NEG	NEG	AMB	POS	NEG	NEG	NEG	NEG	NEG	NEG	NEG	NEG	NEG	NEG	NEG	NEG	NEG	NEG
**UKD-0120122**	SCCmec IVh/j (HO50960412/JCSC6670)	CC22	POS	POS	POS	POS	POS	POS	POS	POS	POS	POS	POS	NEG	NEG	NEG	POS	NEG	NEG	NEG	NEG	NEG	NEG	NEG	POS	NEG	NEG	NEG	NEG	NEG	POS
**UKD-0205319**	SCCmec IVh/j (HO50960412/JCSC6670)	CC22	POS	POS	POS	POS	POS	POS	POS	POS	POS	POS	POS	NEG	NEG	AMB	POS	NEG	NEG	NEG	NEG	NEG	NEG	NEG	NEG	NEG	NEG	NEG	NEG	NEG	NEG
**UKD-0221259**	SCCmec IVh/j (HO50960412/JCSC6670)	CC22	POS	POS	POS	POS	POS	POS	POS	POS	POS	POS	POS	NEG	NEG	NEG	POS	NEG	NEG	NEG	NEG	NEG	NEG	NEG	POS	NEG	NEG	NEG	NEG	NEG	POS
**UKD-0418654**	SCCmec IVh/j (HO50960412/JCSC6670)	CC22	POS	POS	POS	POS	POS	POS	POS	POS	POS	POS	POS	NEG	NEG	NEG	POS	NEG	NEG	NEG	NEG	NEG	NEG	NEG	NEG	NEG	NEG	NEG	NEG	NEG	NEG
**UKD-0429721**	SCCmec IVh/j (HO50960412/JCSC6670)	CC22	POS	POS	POS	POS	POS	POS	POS	POS	POS	POS	POS	NEG	NEG	NEG	POS	NEG	NEG	NEG	NEG	NEG	NEG	NEG	NEG	NEG	NEG	NEG	NEG	NEG	NEG
**UKD-1116194**	SCCmec IVh/j (HO50960412/JCSC6670)	CC22	POS	POS	POS	POS	POS	POS	POS	POS	POS	POS	POS	NEG	NEG	AMB	POS	NEG	AMB	NEG	NEG	NEG	NEG	AMB	POS	NEG	NEG	NEG	NEG	NEG	POS
**UKD-1122367**	SCCmec IVh/j (HO50960412/JCSC6670)	CC22	POS	POS	POS	POS	POS	POS	POS	POS	POS	POS	POS	NEG	NEG	AMB	POS	NEG	NEG	NEG	NEG	NEG	NEG	NEG	NEG	NEG	NEG	NEG	NEG	NEG	NEG
**UKD-14012840**	SCCmec IVh/j (HO50960412/JCSC6670)	CC22	POS	POS	POS	POS	POS	POS	POS	POS	POS	POS	POS	NEG	NEG	AMB	POS	NEG	NEG	NEG	NEG	NEG	NEG	AMB	POS	NEG	NEG	NEG	NEG	NEG	POS
**UKD-14579833**	SCCmec IVh/j (HO50960412/JCSC6670)	CC22	POS	POS	POS	POS	POS	POS	POS	POS	POS	POS	POS	NEG	NEG	AMB	POS	NEG	NEG	NEG	NEG	NEG	NEG	NEG	NEG	NEG	NEG	NEG	NEG	NEG	NEG
**UKD-15579198**	SCCmec IVh/j (HO50960412/JCSC6670)	CC22	POS	POS	POS	POS	POS	POS	POS	POS	POS	POS	POS	NEG	NEG	POS	POS	NEG	NEG	NEG	NEG	NEG	NEG	AMB	POS	NEG	NEG	NEG	NEG	NEG	POS
**UKD-4052193**	SCCmec IVh/j (HO50960412/JCSC6670)	CC22	POS	POS	POS	POS	POS	POS	POS	POS	POS	POS	POS	NEG	NEG	AMB	POS	NEG	NEG	NEG	NEG	NEG	NEG	NEG	NEG	NEG	NEG	NEG	NEG	NEG	NEG
**UKD-124968**	SCCmec IVh/j (HO50960412/JCSC6670)	CC22	POS	POS	POS	POS	POS	POS	POS	POS	POS	POS	POS	NEG	NEG	AMB	POS	NEG	NEG	NEG	NEG	NEG	NEG	NEG	NEG	NEG	NEG	NEG	NEG	NEG	NEG
**UKD-14572546**	SCCmec IVh/j (HO50960412/JCSC6670)	CC22	POS	POS	POS	POS	POS	POS	POS	POS	POS	POS	POS	NEG	NEG	POS	POS	NEG	NEG	NEG	NEG	NEG	NEG	POS	POS	NEG	NEG	NEG	NEG	NEG	POS
**UKD-14582287**	SCCmec IVh/j (HO50960412/JCSC6670)	CC22	POS	POS	POS	POS	POS	POS	POS	POS	POS	POS	POS	NEG	NEG	POS	POS	NEG	NEG	NEG	NEG	NEG	NEG	NEG	NEG	NEG	NEG	NEG	NEG	NEG	NEG
**UKD-14583376**	SCCmec IVh/j (HO50960412/JCSC6670)	CC22	POS	POS	POS	POS	POS	POS	POS	POS	POS	POS	POS	NEG	NEG	POS	POS	NEG	NEG	NEG	NEG	NEG	NEG	NEG	NEG	NEG	NEG	NEG	NEG	NEG	NEG
**UKD-14583496**	SCCmec IVh/j (HO50960412/JCSC6670)	CC22	POS	POS	POS	POS	POS	POS	POS	POS	POS	POS	POS	NEG	NEG	POS	POS	AMB	AMB	NEG	NEG	NEG	NEG	NEG	NEG	NEG	NEG	NEG	NEG	NEG	NEG
**UKD-0013324**	SCCmec IVh/j (HO50960412/JCSC6670)	CC22	POS	POS	POS	POS	POS	POS	POS	POS	POS	POS	POS	NEG	NEG	NEG	POS	NEG	NEG	NEG	NEG	NEG	NEG	NEG	NEG	NEG	NEG	NEG	NEG	NEG	NEG
**UKD-0125329**	SCCmec IVh/j (HO50960412/JCSC6670)	CC22	POS	POS	POS	POS	POS	POS	POS	POS	POS	POS	POS	NEG	NEG	NEG	POS	NEG	NEG	NEG	NEG	NEG	NEG	NEG	NEG	NEG	NEG	NEG	NEG	NEG	NEG
**UKD-0215498**	SCCmec IVh/j (HO50960412/JCSC6670)	CC22	POS	POS	POS	POS	POS	POS	POS	POS	POS	POS	POS	NEG	NEG	NEG	POS	NEG	NEG	NEG	NEG	NEG	NEG	NEG	NEG	NEG	NEG	NEG	NEG	NEG	NEG
Prediction for M08-0126, GenBank FR753166.1	*SCCmec IVh/j+ACME2 (M08-0126)*	*CC22*																													
**UKD-1379483I**	SCCmec IVh/j+ACME2 (M08-0126)	CC22	POS	POS	POS	POS	POS	POS	POS	POS	POS	POS	POS	NEG	NEG	POS	POS	NEG	AMB	NEG	NEG	NEG	NEG	AMB	POS	NEG	NEG	NEG	NEG	NEG	POS
**UKD-5150204**	SCCmec IV+speG+Cu/czrC+ccrAB4	CC22	POS	POS	POS	POS	POS	POS	POS	POS	POS	POS	POS	NEG	NEG	AMB	POS	NEG	NEG	NEG	NEG	NEG	NEG	NEG	NEG	NEG	NEG	NEG	NEG	NEG	NEG
**UKD-15578423**	SCCmec IV+speG+czrC+ccrAB4 var. 1	CC22	POS	POS	POS	POS	POS	POS	POS	POS	POS	POS	POS	NEG	NEG	AMB	POS	NEG	NEG	NEG	NEG	NEG	NEG	AMB	POS	NEG	NEG	NEG	NEG	NEG	POS
**UKD-16582870**	SCCmec IV+speG+czrC+ccrAB4 var. 1	CC22	POS	POS	POS	POS	POS	POS	POS	POS	POS	POS	POS	NEG	NEG	NEG	POS	NEG	NEG	NEG	NEG	NEG	NEG	NEG	POS	NEG	NEG	NEG	NEG	NEG	POS
**UKD-15571940**	SCCmec IV+speG+czrC+ccrAB4 var. 2	CC22	POS	POS	POS	POS	POS	POS	POS	POS	POS	POS	POS	NEG	NEG	AMB	POS	NEG	NEG	NEG	NEG	NEG	NEG	NEG	NEG	NEG	NEG	NEG	NEG	NEG	NEG

Isolate ID	SCCmec type	Clonal complex	SPECIES MARKER							REGULATORY GENES									VIRULENCE : TOX.SCHOCK.TOXIN			VIRULENCE : ENTEROTOXINS										
			Domain 1 of 23S-rRNA	glyceraldehyde 3-phosphate dehydrogenase, locus 1	katalase A	coagulase	thermostable extracellular nuclease	staphylococcal protein A	IgG-binding protein	staphylococcal accessory regulator A	histidine protein kinase, sae locus	sensor protein	accessory gene regulator alleles				haemolysin delta	toxic shock syndrome toxin 1			Entero-toxin A	Entero-toxin A, allele from strain 320E	Entero-toxin A, allele from strain N315 = Entero-toxin P	Entero-toxin B	Entero-toxin C	Entero-toxin D	Entero-toxin E	Entero-toxin H	Entero-toxin J	Entero-toxin K	Entero-toxin L
			rrnD1 (S. aureus)	gapA	katA	CoA	nuc1	spa	sbi	sarA	saeS	vraS	agrI (total)	agrII (total)	agrIII (total)	agrIV (total)	hld	tst1 (consensus)	tst1 ("human" allele)	tst1 ("bovine" allele)	entA	entA (320E)	entA (N315) / entP	entB	entC	entD	entE	entH	entJ	entK	entL
Prediction for CN1, GenBank CP003979.1	SCCmec IVA (CN1)	CC8 (ST72)	POS	POS	POS	POS	POS	POS	POS	AMB	POS	POS	POS	NEG	NEG	NEG	POS	NEG	NEG	NEG	NEG	NEG	NEG	NEG	NEG	NEG	NEG	NEG	NEG	NEG	NEG
UKD-0900871	SCCmec IVA (CN1)	CC8 (ST72)	POS	POS	POS	POS	POS	POS	POS	POS	POS	POS	POS	NEG	NEG	NEG	POS	NEG	NEG	NEG	NEG	NEG	NEG	NEG	NEG	NEG	NEG	NEG	NEG	NEG	NEG
Prediction for CMFT1723, GenBank HF569096.1	SCCmec V (Bengal Bay)																														
KHDN-10405134	SCCmec V (Bengal Bay)	CC1	POS	POS	POS	POS	POS	POS	POS	POS	POS	POS	NEG	NEG	POS	NEG	POS	POS	POS	NEG	POS	NEG	NEG	NEG	NEG	NEG	NEG	POS	NEG	POS	POS
UKD-0932405	SCCmec V (Bengal Bay)	CC1 (ST573/772)	POS	POS	POS	POS	POS	POS	POS	POS	POS	POS	NEG	POS	NEG	NEG	POS	NEG	NEG	NEG	POS	NEG	NEG	NEG	POS	NEG	NEG	NEG	NEG	NEG	POS
UKD-1003510	SCCmec V (Bengal Bay)	CC1 (ST573/772)	POS	POS	POS	POS	POS	POS	POS	POS	POS	POS	NEG	POS	NEG	NEG	POS	NEG	NEG	NEG	POS	NEG	NEG	NEG	POS	NEG	NEG	NEG	NEG	NEG	POS
UKD-1112420	SCCmec V (Bengal Bay)	CC1 (ST573/772)	POS	POS	POS	POS	POS	POS	POS	POS	POS	POS	NEG	POS	NEG	NEG	POS	NEG	NEG	NEG	POS	NEG	NEG	AMB	POS	NEG	NEG	NEG	NEG	NEG	POS
UKD-1275832	SCCmec V (Bengal Bay)	CC1 (ST573/772)	POS	POS	POS	POS	POS	POS	POS	POS	POS	POS	NEG	POS	NEG	NEG	POS	NEG	NEG	NEG	POS	AMB	NEG	AMB	POS	NEG	NEG	NEG	NEG	NEG	POS
UKD-13023811	SCCmec V (Bengal Bay)	CC1 (ST573/772)	POS	POS	POS	POS	POS	POS	POS	POS	POS	POS	NEG	POS	NEG	NEG	POS	NEG	NEG	NEG	POS	NEG	NEG	NEG	POS	NEG	NEG	NEG	NEG	NEG	POS
UKD-14572163	SCCmec V	CC5	POS	POS	POS	POS	POS	POS	POS	POS	POS	POS	NEG	POS	NEG	NEG	POS	NEG	NEG	NEG	NEG	NEG	POS	NEG	NEG	POS	NEG	NEG	POS	NEG	NEG
KHDN-10421133	SCCmec V+fus	CC97	POS	POS	POS	POS	POS	POS	POS	POS	POS	POS	POS	NEG	NEG	AMB	POS	NEG	NEG	NEG	NEG	NEG	NEG	NEG	NEG	NEG	NEG	NEG	NEG	NEG	NEG
KHDN-21343541	SCCmec V+fus+tir+ccrAB1	CC1	POS	POS	POS	POS	POS	POS	POS	POS	POS	POS	NEG	NEG	POS	NEG	POS	NEG	NEG	NEG	POS	NEG	NEG	NEG	NEG	NEG	NEG	POS	NEG	POS	NEG
KHDN-10416893	SCCmec V+czrC+speG+ccrAB4	CC22	POS	POS	POS	POS	POS	POS	POS	POS	POS	POS	POS	NEG	NEG	AMB	POS	NEG	NEG	NEG	NEG	NEG	NEG	NEG	NEG	NEG	NEG	NEG	NEG	NEG	NEG
Prediction for GR1, GenBank AJLX	SCCmec VT (GR1)	CC361	POS	POS	POS	POS	POS	POS	POS	AMB	POS	POS	POS	NEG	NEG	NEG	POS	NEG	NEG	NEG	NEG	NEG	NEG	NEG	NEG	NEG	NEG	NEG	NEG	NEG	NEG
UKD-0422872	SCCmec VT (GR1)	CC152	POS	POS	POS	POS	POS	POS	POS	POS	POS	POS	POS	NEG	NEG	POS	POS	NEG	NEG	NEG	NEG	NEG	NEG	NEG	NEG	NEG	NEG	NEG	NEG	NEG	NEG
Prediction for PM1, GenBank BAFA	SCCmec VT (PM1)	CC59	POS	POS	POS	POS	POS	POS	POS	AMB	POS	POS	POS	NEG	NEG	NEG	POS	NEG	NEG	NEG	NEG	NEG	NEG	POS	NEG	NEG	NEG	NEG	NEG	POS	NEG
KHDN-000871	SCCmec VT (PM1)	CC45 (agr I)	POS	POS	POS	POS	POS	POS	POS	POS	POS	POS	POS	NEG	NEG	NEG	POS	NEG	NEG	NEG	NEG	NEG	NEG	NEG	POS	NEG	NEG	NEG	NEG	NEG	POS
UKD-0725052	SCCmec VT (PM1)	CC59	POS	POS	POS	POS	POS	POS	POS	POS	POS	POS	POS	NEG	NEG	NEG	POS	NEG	NEG	NEG	NEG	NEG	NEG	POS	NEG	NEG	NEG	NEG	NEG	POS	NEG
UKD-1312138	SCCmec VT (PM1)	CC59	POS	POS	POS	POS	POS	POS	POS	POS	POS	POS	POS	NEG	NEG	POS	POS	NEG	NEG	NEG	NEG	NEG	NEG	POS	NEG	NEG	NEG	NEG	NEG	POS	NEG
UKD-Z4728	SCCmec VT (PM1)	CC59	POS	POS	POS	POS	POS	POS	POS	POS	POS	POS	POS	NEG	NEG	AMB	POS	NEG	NEG	NEG	NEG	NEG	NEG	POS	NEG	NEG	NEG	NEG	NEG	POS	NEG
UKD-K0199	SCCmec VT unknown var.1	CC7	POS	POS	POS	POS	POS	POS	POS	POS	POS	POS	POS	NEG	NEG	NEG	POS	NEG	NEG	NEG	NEG	NEG	POS	NEG	NEG	NEG	NEG	NEG	NEG	NEG	NEG
UKD-15584619	SCCmec VT unknown var.2	CC30	POS	POS	POS	POS	POS	POS	POS	POS	POS	POS	NEG	NEG	POS	NEG	POS	NEG	NEG	NEG	POS	AMB	NEG	NEG	NEG	NEG	NEG	NEG	NEG	NEG	NEG
UKD-0712568	SCCmec VT unknown var.3	CC45 (agr I)	POS	POS	POS	POS	POS	POS	POS	POS	POS	POS	POS	NEG	NEG	NEG	POS	NEG	NEG	NEG	NEG	NEG	NEG	NEG	NEG	NEG	NEG	NEG	NEG	NEG	NEG
KHDN-10420296	SCCmec VT+ACME2	CC45 (agr I)	POS	POS	POS	POS	POS	POS	POS	POS	POS	POS	POS	NEG	NEG	NEG	POS	NEG	NEG	NEG	NEG	NEG	NEG	NEG	POS	NEG	NEG	NEG	NEG	NEG	POS
UKD-15577956	SCCmec VT+Cu/czrC+ccrA1	CC398	POS	POS	POS	POS	POS	POS	POS	POS	POS	POS	POS	NEG	NEG	NEG	POS	NEG	NEG	NEG	NEG	NEG	NEG	NEG	NEG	NEG	NEG	NEG	NEG	NEG	NEG
Prediction for S0385, GenBank AM990992	SCCmec VT+czrC (SO385)	CC398	POS	POS	POS	POS	POS	POS	POS	POS	POS	POS	AMB	NEG	NEG	NEG	POS	NEG	NEG	NEG	NEG	NEG	NEG	NEG	NEG	NEG	NEG	NEG	NEG	NEG	NEG
UKD-0628560	SCCmec VT+czrC (SO385)	CC8	POS	POS	POS	POS	POS	POS	POS	POS	POS	POS	POS	NEG	NEG	NEG	POS	NEG	NEG	NEG	NEG	NEG	NEG	NEG	NEG	NEG	NEG	NEG	NEG	NEG	NEG
UKD-0510018	SCCmec VT+czrC (SO385)	CC398	POS	POS	POS	POS	POS	POS	POS	POS	POS	POS	POS	NEG	NEG	NEG	POS	NEG	NEG	NEG	NEG	NEG	NEG	NEG	NEG	NEG	NEG	NEG	NEG	NEG	NEG
UKD-0903558	SCCmec VT+czrC (SO385)	CC398	POS	POS	POS	POS	POS	POS	POS	POS	POS	POS	POS	NEG	NEG	NEG	POS	NEG	NEG	NEG	NEG	NEG	NEG	NEG	NEG	NEG	NEG	NEG	NEG	NEG	NEG
UKD-0929815	SCCmec VT+czrC (SO385)	CC398	POS	POS	POS	POS	POS	POS	POS	POS	POS	POS	POS	NEG	NEG	NEG	POS	NEG	NEG	NEG	NEG	NEG	NEG	NEG	NEG	NEG	NEG	NEG	NEG	NEG	NEG
UKD-0907762	SCCmec VT+czrC (SO385)	CC398	POS	POS	POS	POS	POS	POS	POS	POS	POS	POS	POS	NEG	NEG	NEG	POS	NEG	NEG	NEG	NEG	NEG	NEG	NEG	NEG	NEG	NEG	NEG	NEG	NEG	NEG
UKD-1025105	SCCmec VT+czrC (SO385)	CC398	POS	POS	POS	POS	POS	POS	POS	POS	POS	POS	AMB	NEG	NEG	NEG	POS	NEG	NEG	NEG	NEG	NEG	NEG	NEG	NEG	NEG	NEG	NEG	NEG	NEG	NEG
UKD-11780898	SCCmec VT+czrC (SO385)	CC398	POS	POS	POS	POS	POS	POS	POS	POS	POS	POS	POS	NEG	NEG	NEG	POS	NEG	NEG	NEG	NEG	NEG	NEG	NEG	NEG	NEG	NEG	NEG	NEG	NEG	NEG
UKD-1377009	SCCmec VT+czrC (SO385)	CC398	POS	POS	POS	POS	POS	POS	POS	POS	POS	POS	POS	NEG	NEG	NEG	POS	NEG	NEG	NEG	NEG	NEG	NEG	NEG	NEG	NEG	NEG	NEG	NEG	NEG	NEG
UKD-143406	SCCmec VT+czrC (SO385)	CC398	POS	POS	POS	POS	POS	POS	POS	POS	POS	POS	POS	NEG	NEG	NEG	POS	NEG	NEG	NEG	NEG	NEG	NEG	NEG	NEG	NEG	NEG	NEG	NEG	NEG	NEG
UKD-1400363	SCCmec VT+czrC (SO385)	CC398	POS	POS	POS	POS	POS	POS	POS	POS	POS	POS	POS	NEG	NEG	NEG	POS	NEG	NEG	NEG	NEG	NEG	NEG	NEG	NEG	NEG	NEG	NEG	NEG	NEG	NEG
UKD-14571852	SCCmec VT+czrC (SO385)	CC398	POS	POS	POS	POS	POS	POS	POS	POS	POS	POS	POS	NEG	NEG	NEG	POS	AMB	NEG	NEG	NEG	NEG	NEG	NEG	NEG	NEG	NEG	NEG	NEG	NEG	NEG
UKD-14582574	SCCmec VT+czrC (SO385)	CC398	POS	POS	POS	POS	POS	POS	POS	POS	POS	POS	POS	NEG	NEG	NEG	POS	NEG	NEG	NEG	NEG	NEG	NEG	NEG	NEG	NEG	NEG	NEG	NEG	NEG	NEG
UKD-152108	SCCmec VT+czrC (SO385)	CC398	POS	POS	POS	POS	POS	POS	POS	POS	POS	POS	POS	NEG	NEG	NEG	POS	NEG	NEG	NEG	NEG	NEG	NEG	NEG	NEG	NEG	NEG	NEG	NEG	NEG	NEG
UKD-15573321	SCCmec VT+czrC (SO385)	CC398	POS	POS	POS	POS	POS	POS	POS	POS	POS	POS	POS	NEG	NEG	NEG	POS	NEG	NEG	NEG	NEG	NEG	NEG	NEG	NEG	NEG	NEG	NEG	NEG	NEG	NEG
UKD-15584034	SCCmec VT+czrC , ydhK-neg. variant	CC398	POS	POS	POS	POS	POS	POS	POS	POS	POS	POS	POS	NEG	NEG	NEG	POS	NEG	NEG	NEG	NEG	NEG	NEG	NEG	NEG	NEG	NEG	NEG	NEG	NEG	NEG
UKD-0932581	SCCmecVT+fus+czrC	CC22	POS	POS	AMB	AMB	POS	POS	POS	POS	POS	POS	POS	NEG	NEG	NEG	POS	NEG	NEG	NEG	NEG	NEG	NEG	NEG	POS	NEG	NEG	NEG	NEG	NEG	POS
UKD-1121994	SCCmecVT+fus+czrC	CC22	POS	POS	POS	POS	POS	POS	POS	POS	POS	POS	POS	NEG	NEG	AMB	POS	NEG	NEG	NEG	NEG	NEG	NEG	AMB	POS	NEG	NEG	NEG	NEG	NEG	POS
UKD-1272252	SCCmecVT+fus+czrC	CC22	POS	POS	POS	POS	POS	POS	POS	POS	POS	POS	POS	NEG	NEG	AMB	POS	NEG	NEG	NEG	NEG	NEG	NEG	AMB	POS	NEG	NEG	NEG	NEG	NEG	POS
Prediction for MRSA18	SCCmec VI+fus (MRSA18)																														
UKD-1103380	SCCmec VI+fus (MRSA18)	CC7	POS	POS	POS	POS	POS	POS	POS	POS	POS	POS	POS	NEG	NEG	NEG	POS	NEG	NEG	NEG	NEG	NEG	POS	NEG	NEG	NEG	NEG	NEG	NEG	NEG	NEG
Prediction for M10/0061, GenBank FR823292.1	SCCmec XI (LGA251/M10-61)	CC130																													
Reference strain Dublin-DSH_M10 0061	SCCmec XI (LGA251/M10-61)	CC130	POS	POS	POS	POS	POS	POS	POS	POS	POS	POS	NEG	NEG	POS	NEG	POS	NEG	NEG	NEG	NEG	NEG	NEG	NEG	NEG	NEG	NEG	NEG	NEG	NEG	NEG
Prediction for LGA251, GenBank FR821779.1	SCCmec XI (LGA251/M10-61)	ST425																													
UKD-14015835	SCCmec XI (LGA251/M10-61)	CC130	POS	POS	POS	POS	POS	POS	POS	POS	POS	POS	NEG	NEG	POS	NEG	POS	NEG	NEG	NEG	NEG	NEG	NEG	NEG	NEG	NEG	NEG	NEG	NEG	NEG	NEG
UKD-16573252	SCCmec XI (LGA251/M10-61)	CC130	POS	POS	POS	POS	POS	POS	POS	POS	POS	POS	NEG	NEG	POS	NEG	POS	NEG	NEG	NEG	NEG	NEG	NEG	NEG	NEG		NEG	NEG		NEG	
UKD-0217002	PseudoSCCmec, class B mec complex+Hg	CC8	POS	POS	POS	POS	POS	POS	POS	POS	POS	POS	POS	NEG	NEG	NEG	POS	NEG	NEG	NEG	NEG	NEG	NEG	POS	NEG	NEG	NEG	NEG	NEG	POS	NEG
UKD-0008510	PseudoSCCmec, class B mec complex+Hg	CC8	POS	POS	POS	POS	POS	POS	POS	POS	POS	POS	POS	NEG	NEG	NEG	POS	NEG	NEG	NEG	POS	NEG	NEG	POS	NEG	NEG	NEG	NEG	NEG	POS	NEG
UKD-0110962	PseudoSCCmec, class B mec complex+Hg	CC8	POS	POS	POS	POS	POS	POS	POS	POS	POS	POS	POS	NEG	NEG	NEG	POS	NEG	NEG	NEG	POS	NEG	NEG	POS	NEG	NEG	NEG	NEG	NEG	POS	NEG
KHDN-10408593	PseudoSCCmec, class B mec complex+Cu/Hg	CC8	POS	POS	POS	POS	POS	POS	POS	POS	POS	POS	POS	NEG	NEG	NEG	POS	NEG	NEG	NEG	NEG	NEG	NEG	NEG	NEG	NEG	NEG	NEG	NEG	POS	NEG
UKD-0007201	PseudoSCCmec, class B mec complex	CC5	POS	POS	POS	POS	POS	POS	POS	POS	POS	POS	NEG	POS	NEG	NEG	POS	NEG	NEG	NEG	NEG	NEG	NEG	NEG	NEG	NEG	NEG	NEG	NEG	NEG	NEG
UKD-0033204	PseudoSCCmec, class B mec complex	CC5	POS	POS	POS	POS	POS	POS	POS	POS	POS	POS	NEG	POS	NEG	NEG	POS	NEG	NEG	NEG	NEG	NEG	NEG	NEG	NEG	NEG	NEG	NEG	NEG	NEG	NEG
UKD-0134329	PseudoSCCmec, class B mec complex	CC5	POS	POS	POS	POS	POS	POS	POS	POS	AMB	POS	NEG	AMB	NEG	NEG	POS	NEG	NEG	NEG	NEG	NEG	NEG	NEG	NEG	NEG	NEG	NEG	NEG	NEG	NEG
UKD-0806946	PseudoSCCmec class C+As/Cu	CC398	POS	POS	POS	POS	POS	POS	POS	POS	POS	POS	POS	NEG	NEG	NEG	POS	NEG	NEG	NEG	NEG	NEG	NEG	NEG	NEG	NEG	NEG	NEG	NEG	NEG	NEG
UKD-0500610	ACME2	CC8	POS	POS	POS	POS	POS	POS	POS	POS	POS	POS	POS	NEG	NEG	NEG	POS	NEG	NEG	NEG	NEG	NEG	NEG	NEG	NEG	POS	NEG	NEG	POS	NEG	NEG
UKD-0610438	ACME2	CC8	POS	POS	POS	POS	POS	POS	POS	POS	POS	POS	POS	NEG	NEG	AMB	POS	NEG	NEG	NEG	NEG	NEG	NEG	NEG	NEG	POS	NEG	NEG	POS	NEG	NEG
KHDN-10409344	ACME2	CC8	POS	POS	POS	POS	POS	POS	POS	POS	POS	POS	POS	NEG	NEG	POS	POS	NEG	NEG	NEG	NEG	NEG	NEG	NEG	NEG	NEG	NEG	NEG	NEG	NEG	NEG
UKD-0322559	ACME2+speG+czrC+ccrAB4	CC8	POS	POS	POS	POS	POS	POS	POS	POS	POS	POS	POS	NEG	NEG	NEG	POS	NEG	NEG	NEG	NEG	NEG	NEG	NEG	NEG	POS	NEG	NEG	POS	NEG	NEG
UKD-0501998	ACME2+speG+czrC+ccrAB4	CC8	POS	POS	POS	POS	POS	POS	POS	POS	POS	POS	POS	NEG	NEG	NEG	POS	NEG	NEG	NEG	NEG	NEG	NEG	NEG	NEG	POS	NEG	NEG	POS	NEG	NEG
UKD-0505213	ACME2+speG+czrC+ccrAB4	CC8	POS	POS	POS	POS	POS	POS	POS	POS	POS	POS	POS	NEG	NEG	NEG	POS	NEG	NEG	NEG	NEG	NEG	NEG	NEG	NEG	POS	NEG	NEG	POS	NEG	NEG
Prediction for MSSA476, GenBank BX571857.1	fus+tir+ccrAB1 (MSSA476)	CC1	POS	POS	POS	POS	POS	POS	POS	AMB	POS	POS	NEG	NEG	POS	NEG	POS	NEG	NEG	NEG	POS	NEG	NEG	NEG	NEG	NEG	NEG	POS	NEG	POS	NEG
Reference strain Sanger 476	fus+tir+ccrAB1 (MSSA476)	CC1	POS	POS	POS	POS	POS	POS	POS	POS	POS	POS	NEG	NEG	POS	NEG	POS	NEG	NEG	NEG	POS	NEG	NEG	NEG	NEG	NEG	NEG	POS	NEG	POS	NEG
UKD-14023914	fus+tir+ccrAB1 (MSSA476)	CC1	POS	POS	POS	POS	POS	POS	POS	POS	POS	POS	NEG	NEG	POS	NEG	POS	NEG	NEG	NEG	POS	NEG	NEG	POS	NEG	NEG	NEG	POS	NEG	POS	NEG
UKD-K00034	kdp+ccrAB2	CC182	POS	POS	POS	POS	POS	POS	POS	POS	POS	POS	POS	NEG	NEG	AMB	POS	NEG	NEG	NEG	NEG	NEG	NEG	NEG	NEG	NEG	NEG	POS	NEG	NEG	NEG
UKD-15580369	As+ccrAB4	CC22	POS	POS	POS	POS	POS	POS	POS	POS	POS	POS	POS	NEG	NEG	POS	POS	NEG	NEG	NEG	NEG	NEG	NEG	AMB	POS	NEG	NEG	NEG	NEG	NEG	POS
UKD-15570100	ACME3+ccrAB1 (Strain 21342)	CC80	POS	POS	POS	POS	POS	POS	POS	POS	POS	POS	NEG	NEG	POS	NEG	POS	NEG	NEG	NEG	NEG	NEG	NEG	NEG	NEG	NEG	NEG	NEG	NEG	NEG	NEG
UKD-1010678	speG+czrC+ccrAB4	CC8	POS	POS	POS	POS	POS	POS	POS	POS	POS	POS	POS	NEG	NEG	NEG	POS	NEG	NEG	NEG	NEG	NEG	NEG	NEG	NEG	NEG	NEG	NEG	NEG	NEG	NEG
UKD-1008243	speG+czrC+ccrAB4	CC8	POS	POS	POS	POS	POS	POS	POS	POS	POS	POS	POS	NEG	NEG	AMB	POS	NEG	NEG	NEG	NEG	NEG	NEG	NEG	NEG	POS	NEG	NEG	POS	NEG	NEG
UKD-1009430	speG+czrC+ccrAB4	CC8	POS	POS	POS	POS	POS	POS	POS	POS	POS	POS	POS	NEG	NEG	NEG	POS	NEG	NEG	NEG	NEG	NEG	NEG	NEG	NEG	POS	NEG	NEG	POS	NEG	NEG
UKD-12071828	speG+czrC+ccrAB4	CC22	POS	POS	POS	POS	POS	POS	POS	POS	POS	POS	POS	NEG	NEG	AMB	POS	NEG	NEG	NEG	NEG	NEG	NEG	AMB	POS	NEG	NEG	NEG	NEG	NEG	POS
UKD-14011883	speG+czrC+ccrAB4	CC22	POS	POS	POS	POS	POS	POS	POS	POS	POS	POS	POS	NEG	NEG	AMB	POS	NEG	NEG	NEG	NEG	NEG	NEG	AMB	POS	NEG	NEG	NEG	NEG	NEG	POS
UKD-15008293	speG+czrC+ccrAB4	CC22	POS	POS	POS	POS	POS	POS	POS	POS	POS	POS	POS	NEG	NEG	AMB	POS	NEG	NEG	NEG	NEG	NEG	NEG	POS	POS	NEG	NEG	NEG	NEG	NEG	POS
UKD-1514877	speG+czrC+ccrAB4	CC22	POS	POS	POS	POS	POS	POS	POS	POS	POS	POS	POS	NEG	NEG	AMB	POS	NEG	NEG	NEG	NEG	NEG	NEG	AMB	POS	NEG	NEG	NEG	NEG	NEG	POS
UKD-15580971	speG+czrC+ccrAB4	CC22	POS	POS	POS	POS	POS	POS	POS	POS	POS	POS	POS	NEG	NEG	AMB	POS	NEG	NEG	NEG	NEG	NEG	NEG	AMB	POS	NEG	NEG	NEG	NEG	NEG	POS
UKD-15582646	speG+czrC+ccrAB4	CC22	POS	POS	POS	POS	POS	POS	POS	POS	POS	POS	POS	NEG	NEG	AMB	POS	NEG	NEG	NEG	NEG	NEG	NEG	AMB	POS	NEG	NEG	NEG	NEG	NEG	POS

Isolate ID	SCCmec type	Clonal complex	VIRULENCE : ENTEROTOXINS										VIRULENCE : HLG AND LEUKOCIDINS													VIRULENCE : HAEMOLYSINS			
			Entero- toxin Q	Entero- toxin R	egc cluster	Entero- toxin G	Entero- toxin I	Entero- toxin M	Entero- toxin N	Entero- toxin O	Entero- toxin U and/or Y	enterotoxin-like protein ORF CM14	haemo-lysin gamma / leuko- cidin, component B	haemolysin gamma / leukocidin, component C		haemolysin gamma, component A	Panton Valentine leukocidin F component	Panton Valentine leukocidin S component	leukocidin from ruminant strains		leukocidin D component	leukocidin E component	leukocidin/ haemolysin toxin family protein			putative membrane protein	haemolysin alpha	putative membrane protein	
			entQ	entR	egc (total)	seg	sei	selm	seln (consensus)	selo	selu	ORF CM14	lukF	lukS	lukS (ST22+ST45)	hlgA	lukF-PV	lukS-PV	lukF-PV (P83)	lukM	lukD	lukE	lukX	lukY	lukY (ST30+ST45)	corB (=hl)	hla	hlIII (cons)	hlIII (other than RF122)
VIRTUAL HYBRIDISATION for COL, GenBank CP000046	SCCmec I (COL)	CC8	POS	NEG	NEG	NEG	NEG	NEG	NEG	NEG	NEG	NEG	POS	POS	AMB	POS	NEG	NEG	NEG	NEG	POS	POS	POS	POS	NEG	POS	POS	POS	POS
Reference strain COL	SCCmec I (COL)	CC8	POS	NEG	NEG	NEG	NEG	NEG	NEG	NEG	NEG	NEG	POS	POS	AMB	POS	NEG	NEG	NEG	NEG	POS	POS	POS	POS	NEG	POS	POS	POS	POS
UKD-0007063	SCCmec I (COL)	CC5	NEG	NEG	POS	POS	POS	POS	POS	POS	POS	NEG	POS	POS	POS	POS	NEG	NEG	AMB	NEG	POS	POS	POS	POS	NEG	POS	POS	POS	POS
UKD-0220759	SCCmec I (COL)	CC5	NEG	NEG	POS	POS	POS	POS	POS	POS	POS	NEG	POS	POS	NEG	POS	NEG	NEG	NEG	NEG	POS	AMB	AMB	POS	NEG	POS	POS	POS	POS
UKD-0308986	SCCmec I (COL)	CC5	NEG	NEG	POS	POS	POS	POS	POS	POS	POS	NEG	POS	POS	POS	POS	NEG	NEG	NEG	NEG	POS	POS	POS	POS	NEG	POS	POS	POS	POS
UKD-0500009	SCCmec I (COL)	CC5	NEG	NEG	POS	POS	POS	POS	POS	POS	POS	NEG	POS	POS	POS	POS	NEG	NEG	NEG	NEG	POS	POS	POS	POS	NEG	POS	POS	POS	POS
UKD-0508483	SCCmec I (COL)	CC5	NEG	NEG	AMB	NEG	NEG	NEG	NEG	POS	NEG	NEG	POS	POS	NEG	POS	NEG	NEG	NEG	NEG	NEG	NEG	POS	POS	NEG	POS	POS	POS	POS
UKD-0519001	SCCmec I (COL)	CC5	NEG	NEG	AMB	NEG	NEG	NEG	NEG	POS	NEG	NEG	POS	POS	AMB	POS	NEG	NEG	NEG	NEG	NEG	NEG	POS	POS	NEG	POS	POS	POS	POS
UKD-0701876	SCCmec I (COL)	CC5	NEG	NEG	AMB	NEG	NEG	NEG	NEG	POS	NEG	NEG	POS	POS	POS	POS	NEG	NEG	NEG	NEG	NEG	NEG	AMB	POS	NEG	POS	POS	POS	POS
UKD-0708865	SCCmec I (COL)	CC5	NEG	NEG	POS	POS	POS	POS	POS	POS	NEG	NEG	POS	POS	NEG	POS	NEG	NEG	NEG	NEG	POS	POS	NEG	POS	NEG	POS	POS	POS	POS
UKD-1001782	SCCmec I (COL)	CC5	NEG	NEG	POS	POS	POS	POS	POS	POS	POS	NEG	POS	POS	AMB	POS	NEG	NEG	NEG	NEG	POS	POS	POS	POS	NEG	POS	POS	POS	POS
Prediction for PSP1996, GenBank ANHU	SCCmec I (PSP1996)	CC8	NEG	NEG	NEG	NEG	NEG	NEG	NEG	NEG	NEG	NEG	POS	POS	AMB	POS	NEG	NEG	NEG	NEG	POS	POS	POS	POS	NEG	POS	POS	POS	POS
UKD-0261727	SCCmec I (PSP1996)	CC8	NEG	NEG	NEG	NEG	NEG	NEG	NEG	NEG	NEG	NEG	POS	POS	AMB	POS	NEG	NEG	NEG	NEG	POS	POS	POS	POS	NEG	POS	POS	POS	POS
VIRTUAL HYBRIDISATION for MRSA7 : VIRTUAL HYBRIL	SCCmec I+fus+tirS (Geraldine Clone)	CC5	NEG	POS	POS	POS	POS	POS	POS	POS	POS	NEG	POS	POS	AMB	POS	NEG	NEG	NEG	NEG	POS	POS	POS	POS	NEG	POS	POS	POS	POS
UKD-1221930	SCCmec I+fus+tirS (Geraldine Clone)	CC5	NEG	POS	POS	POS	POS	POS	POS	POS	POS	NEG	POS	POS	AMB	POS	NEG	NEG	NEG	NEG	POS	POS	POS	POS	NEG	POS	POS	POS	POS
Prediction for N315, GenBank BA000018.3	SCCmec II (N315)	CC5	NEG	NEG	POS	POS	POS	POS	POS	POS	POS	NEG	POS	POS	AMB	POS	NEG	NEG	NEG	NEG	POS	POS	POS	POS	NEG	POS	POS	POS	POS
Reference strain N315	SCCmec II (N315)	CC5	NEG	NEG	POS	POS	POS	POS	POS	POS	POS	NEG	POS	POS	NEG	POS	NEG	NEG	NEG	NEG	POS	POS	POS	POS	NEG	POS	POS	POS	POS
Prediction for MRSA252, GenBank BX571856.1	SCCmec II (N315)	CC30	NEG	NEG	POS	POS	POS	POS	POS	POS	POS	NEG	POS	POS	AMB	POS	NEG	NEG	NEG	NEG	NEG	NEG	POS	NEG	POS	POS	POS	POS	POS
Reference strain Sanger 252	SCCmec II (N315)	CC30	NEG	NEG	POS	POS	POS	POS	POS	POS	POS	NEG	POS	POS	NEG	POS	NEG	NEG	NEG	NEG	NEG	NEG	POS	NEG	POS	POS	POS	POS	POS
UKD-0230792	SCCmec II (N315)	CC30	NEG	NEG	POS	POS	POS	POS	POS	POS	POS	NEG	POS	POS	AMB	POS	NEG	NEG	NEG	NEG	NEG	NEG	POS	NEG	POS	POS	POS	POS	POS
Prediction for Mu50, GenBank BA000017.4	SCCmec II (N315)	CC5	NEG	NEG	POS	POS	POS	POS	POS	POS	POS	NEG	POS	POS	AMB	POS	NEG	NEG	NEG	NEG	POS	POS	POS	POS	NEG	POS	POS	POS	POS
Reference strain MU50	SCCmec II (N315)	CC5	NEG	NEG	POS	POS	POS	POS	POS	POS	POS	NEG	POS	POS	AMB	POS	NEG	NEG	NEG	NEG	POS	POS	POS	POS	NEG	POS	POS	POS	POS
Prediction for JH1, GenBank CP000736.1	SCCmec II (JH1/JH9)	CC5	NEG	NEG	POS	POS	POS	POS	POS	POS	POS	NEG	POS	POS	AMB	POS	NEG	NEG	NEG	NEG	POS	POS	POS	POS	NEG	POS	POS	POS	POS
Prediction for JH9, GenBank CP000703.1	SCCmec II (JH1/JH9)	CC5	NEG	NEG	POS	POS	POS	POS	POS	POS	POS	NEG	POS	POS	AMB	POS	NEG	NEG	NEG	NEG	POS	POS	POS	POS	NEG	POS	POS	POS	POS
Reference strain Dublin-DSH_AR07.4_0237	SCCmec II (JH1/JH9)		NEG	POS	POS	POS	POS	POS	POS	POS	POS	NEG	POS	POS	POS	POS	NEG	NEG	NEG	NEG	NEG	POS	POS	POS	NEG	POS	POS	POS	POS
Reference strain NARSA_382 / USA100	SCCmec II (JH1/JH9)	CC5	NEG	POS	POS	POS	POS	POS	POS	POS	POS	NEG	POS	POS	AMB	POS	NEG	NEG	NEG	NEG	POS	POS	POS	POS	NEG	POS	POS	POS	POS
UKD-0046921	SCCmec II (JH1/JH9)	CC5	NEG	POS	POS	POS	POS	POS	POS	POS	POS	NEG	POS	POS	POS	POS	NEG	NEG	NEG	NEG	POS	POS	POS	POS	NEG	POS	POS	POS	POS
UKD-0333415	SCCmec II (JH1/JH9)	CC5	NEG	NEG	POS	POS	POS	POS	POS	POS	POS	NEG	POS	POS	AMB	POS	NEG	NEG	NEG	NEG	POS	POS	POS	POS	NEG	POS	POS	POS	POS
UKD-0341645	SCCmec II (JH1/JH9)	CC5	NEG	POS	POS	POS	POS	POS	POS	POS	POS	NEG	POS	POS	POS	POS	NEG	NEG	NEG	NEG	POS	POS	POS	POS	NEG	POS	POS	POS	POS
UKD-0504626	SCCmec II (JH1/JH9)	CC5	NEG	AMB	AMB	AMB	POS	POS	NEG	AMB	POS	NEG	POS	AMB	NEG	POS	NEG	NEG	NEG	NEG	POS	POS	NEG	POS	NEG	POS	POS	POS	AMB
UKD-0507581	SCCmec II (JH1/JH9)	CC5	POS	POS	POS	POS	POS	POS	POS	POS	POS	NEG	POS	POS	NEG	POS	NEG	NEG	NEG	NEG	POS	POS	POS	POS	NEG	POS	POS	POS	POS
UKD-0507903	SCCmec II (JH1/JH9)	CC5	AMB	NEG	POS	POS	POS	POS	POS	POS	POS	NEG	POS	POS	NEG	POS	NEG	NEG	NEG	NEG	POS	POS	POS	POS	NEG	POS	POS	POS	POS
UKD-0700817	SCCmec II (JH1/JH9)	CC5	NEG	NEG	POS	POS	POS	POS	POS	POS	POS	NEG	POS	POS	NEG	POS	NEG	NEG	NEG	NEG	POS	POS	AMB	POS	NEG	POS	POS	POS	POS
UKD-1014561	SCCmec II (JH1/JH9)	CC5	NEG	NEG	POS	POS	POS	POS	POS	POS	POS	NEG	POS	POS	POS	POS	NEG	NEG	NEG	NEG	POS	POS	POS	POS	NEG	POS	POS	POS	POS
UKD-1037507	SCCmec II (JH1/JH9)	CC5	NEG	POS	POS	POS	POS	POS	POS	POS	POS	NEG	POS	POS	POS	POS	NEG	NEG	NEG	NEG	POS	POS	POS	POS	NEG	POS	POS	POS	POS
UKD-1038321	SCCmec II (JH1/JH9)	CC5	NEG	AMB	POS	POS	POS	POS	POS	AMB	POS	NEG	POS	POS	NEG	POS	NEG	NEG	NEG	NEG	POS	POS	AMB	POS	NEG	POS	POS	POS	POS
UKD-1111608	SCCmec II (JH1/JH9)	CC5	NEG	POS	POS	POS	POS	POS	POS	POS	POS	NEG	POS	POS	POS	POS	NEG	NEG	NEG	NEG	POS	POS	POS	POS	NEG	POS	POS	POS	POS
UKD-12753135	SCCmec II (JH1/JH9)	CC5	NEG	NEG	POS	POS	POS	POS	POS	POS	POS	NEG	POS	POS	POS	POS	NEG	NEG	NEG	NEG	POS	POS	POS	POS	NEG	POS	POS	POS	POS
UKD-1312816	SCCmec II (JH1/JH9)	CC5	NEG	POS	POS	POS	POS	POS	POS	POS	POS	NEG	POS	POS	POS	POS	NEG	NEG	NEG	NEG	POS	POS	POS	POS	NEG	POS	POS	POS	POS
UKD-1401623	SCCmec II (JH1/JH9)	CC5	NEG	POS	POS	POS	POS	POS	POS	POS	POS	NEG	POS	POS	POS	POS	NEG	NEG	AMB	NEG	POS	POS	POS	POS	NEG	POS	POS	POS	POS
UKD-15571943	SCCmec II+czrC+ccrA/B-4	CC5	NEG	NEG	POS	POS	POS	POS	POS	POS	POS	NEG	POS	POS	POS	POS	NEG	NEG	NEG	NEG	POS	POS	POS	POS	NEG	POS	POS	POS	POS
UKD-15576888	SCCmec II+czrC+ccrA/B-4	CC5	NEG	NEG	POS	POS	POS	POS	POS	POS	POS	NEG	POS	POS	POS	POS	NEG	NEG	NEG	NEG	POS	POS	POS	POS	NEG	POS	POS	POS	POS
UKD-14576461	SCCmec II+speG+czrC+ccrA/B-4	CC5	NEG	POS	POS	POS	POS	POS	POS	POS	POS	NEG	POS	POS	POS	POS	NEG	NEG	AMB	NEG	POS	POS	POS	POS	NEG	POS	POS	POS	POS
UKD-15581649	SCCmec II+speG+czrC+ccrA/B-4	CC5	NEG	POS	POS	POS	POS	POS	POS	POS	POS	NEG	POS	POS	POS	POS	NEG	NEG	NEG	NEG	POS	POS	POS	POS	NEG	POS	POS	POS	POS
Prediction for CIGC345D, GenBank AHVO	SCCmec II A/B/D	CC8	NEG	NEG	NEG	NEG	NEG	NEG	NEG	NEG	NEG	NEG	POS	POS	AMB	POS	NEG	NEG	NEG	NEG	POS	POS	POS	POS	NEG	POS	POS	POS	POS
UKD-12K00171	SCCmec II A/B/D	CC8	NEG	NEG	NEG	NEG	NEG	NEG	NEG	NEG	NEG	NEG	POS	POS	POS	POS	NEG	NEG	NEG	NEG	POS	POS	POS	POS	NEG	POS	POS	POS	POS
Prediction for JKD6008, GenBank CP002120.1	SCC [mec III+Cd] (JKD6008)	CC8 (ST239)	POS	NEG	NEG	NEG	NEG	NEG	NEG	NEG	NEG	NEG	POS	POS	AMB	POS	NEG	NEG	NEG	NEG	NEG	NEG	POS	POS	NEG	POS	POS	POS	POS
Prediction for JKD6009, GenBank ABSA	SCC [mec III+Cd] (JKD6008)	CC8 (ST239)	POS	NEG	NEG	NEG	NEG	NEG	NEG	NEG	NEG	NEG	POS	POS	AMB	POS	NEG	NEG	NEG	NEG	NEG	NEG	POS	POS	NEG	POS	POS	POS	POS
Reference strain ATCC_33592	SCC [mec III+Cd] (ATCC33592)	CC8 (ST239)	POS	NEG	NEG	NEG	NEG	NEG	NEG	NEG	NEG	NEG	POS	POS	POS	POS	NEG	NEG	NEG	NEG	POS	POS	POS	POS	NEG	POS	POS	POS	POS
Prediction for PPUKM-775-2009, GenBank AMRE	SCC [mec III+Cd+ccrC] (PPUKM-775-2009)	CC8 (ST239)	NEG	NEG	NEG	NEG	NEG	NEG	NEG	NEG	NEG	NEG	POS	POS	AMB	POS	NEG	NEG	NEG	NEG	POS	POS	POS	POS	NEG	POS	POS	POS	POS
Prediction for T0131, GenBank CP002643.1	SCC [mec III+Cd+ccrC] (T0131)	CC8 (ST239)	POS	NEG	NEG	NEG	NEG	NEG	NEG	NEG	NEG	NEG	POS	POS	AMB	POS	NEG	NEG	NEG	NEG	POS	POS	POS	POS	NEG	POS	POS	POS	POS
Prediction for XN108, GenBank CP007447.1	SCC [mec III+Cd+ccrC] (XN108)	CC8 (ST239)	POS	NEG	NEG	NEG	NEG	NEG	NEG	NEG	NEG	NEG	POS	POS	AMB	POS	NEG	NEG	NEG	NEG	POS	POS	POS	POS	NEG	POS	POS	POS	POS
Prediction for CN79, GenBank ANCJ	SCC [mec III+Cd+ccrC] (CN79)	CC8 (ST239)	POS	NEG	NEG	NEG	NEG	NEG	NEG	NEG	NEG	NEG	POS	POS	AMB	POS	NEG	NEG	NEG	NEG	POS	POS	POS	POS	NEG	POS	POS	POS	POS
Prediction for 16K, GenBank BABZ	SCC [mec III+Cd+ccrC] (CN79)	CC8 (ST239)	POS	NEG	NEG	NEG	NEG	NEG	NEG	NEG	NEG	NEG	POS	POS	AMB	POS	NEG	NEG	NEG	NEG	POS	POS	POS	POS	NEG	POS	POS	POS	POS
UKD-0835987	SCC [mec III+Cd+ccrC] (CN79)	CC8 (ST239)	POS	NEG		NEG	NEG	NEG	NEG	NEG	NEG	NEG	POS	POS	AMB	POS	NEG	NEG	NEG	NEG	POS	POS	POS	POS	NEG	POS	POS	POS	POS
UKD-16581769	SCCmec III+Cd+ccrC	CC8 (ST239)	POS	NEG	NEG	NEG	NEG	NEG	NEG	NEG	NEG	NEG	POS	POS	NEG	POS	NEG	NEG	NEG	NEG	POS	POS	POS	POS	NEG	POS	POS	POS	POS
Prediction for Bmb9393, GenBank CP005288.1	SCCmec III+Cd/Hg+ccrC (Bmb9393)	CC8 (ST239)	NEG	NEG	NEG	NEG	NEG	NEG	NEG	NEG	NEG	NEG	POS	POS	AMB	POS	NEG	NEG	NEG	NEG	POS	POS	POS	POS	NEG	POS	POS	POS	POS
UKD-15574263	SCCmec III+Cd/Hg+ccrC (Bmb9393)	CC8 (ST239)	NEG	NEG	NEG	NEG	NEG	NEG	NEG	NEG	NEG	NEG	POS	POS	POS	POS	NEG	NEG	NEG	NEG	POS	POS	POS	POS	NEG	POS	POS	POS	POS
Prediction for SK1585, GenBank AYLT	SCCmec III+ccrC+Cd/Hg (SK1585)	CC8 (ST239)	NEG	NEG	POS	POS	POS	POS	POS	POS	POS	NEG	POS	POS	AMB	POS	NEG	NEG	NEG	NEG	NEG	NEG	NEG	NEG	POS	POS	POS	POS	NEG
UKD-105861	SCCmec III+ccrC+Cd/Hg (SK1585)	CC8 (ST239)	NEG	NEG	NEG	NEG	NEG	NEG	NEG	NEG	NEG	NEG	POS	POS	NEG	POS	NEG	NEG	NEG	NEG	POS	POS	POS	POS	NEG	POS	POS	POS	POS
UKD-134572	SCCmec III+ccrC+Cd/Hg (SK1585)	CC8 (ST239)	NEG	NEG	NEG	NEG	NEG	NEG	NEG	NEG	NEG	NEG	POS	POS	NEG	POS	NEG	NEG	NEG	NEG	POS	POS	POS	POS	NEG	POS	POS	POS	POS
UKD-139143	SCCmec III+ccrC+Cd/Hg (SK1585)	CC8 (ST239)	NEG	NEG	NEG	NEG	NEG	NEG	NEG	NEG	NEG	NEG	POS	POS	NEG	POS	NEG	NEG	NEG	NEG	POS	POS	AMB	POS	NEG	POS	POS	POS	POS
Prediction for TW20, GenBank FN433596.1	SCC [mec III+Cd/Hg+ccrC] (TW20)	CC8 (ST239)	POS	NEG	NEG	NEG	NEG	NEG	NEG	NEG	NEG	NEG	POS	POS	AMB	POS	NEG	NEG	NEG	NEG	POS	POS	POS	POS	NEG	POS	POS	POS	POS
Prediction for ATCC BAA-39, GenBank AEEK	SCC [mec III+Cd/Hg+ccrC] (TW20)	CC8 (ST239)	POS	NEG	NEG	NEG	NEG	NEG	NEG	NEG	NEG	NEG	POS	POS	AMB	POS	NEG	NEG	NEG	NEG	POS	POS	POS	POS	NEG	POS	POS	POS	POS

Isolate ID	SCCmec type	Clonal complex	VIRULENCE : ENTEROTOXINS									
			Entero- toxin Q	Entero-toxin R	egc cluster	Entero-toxin G	Entero-toxin I	Entero-toxin M	Entero-toxin N	Entero-toxin O	Entero-toxin U and/or Y	enterotoxin-like protein ORF CM14
			entQ	entR	egc (total)	seg	sei	selm	seln (consensus)	selo	selu	ORF CM14
Prediction for MW2, GenBank BA000033.2	SCCmec IVa (MW2)	CC1	POS	NEG	NEG	NEG	NEG	NEG	NEG	NEG	NEG	NEG
Reference strain MW2	SCCmec IVa (MW2)	CC1	POS	NEG	NEG	NEG	NEG	NEG	NEG	NEG	NEG	NEG
Reference strain CA05 (JCSC1968)	SCCmec IVa (MW2)	CC45	NEG	NEG	AMB	POS	POS	POS	POS	POS	POS	NEG
UKD-0127106	SCCmec IVa (MW2)	CC1	POS	NEG	NEG	NEG	NEG	NEG	NEG	NEG	NEG	NEG
UKD-0701446	SCCmec IVa (MW2)	CC1	POS	NEG	NEG	NEG	NEG	NEG	NEG	NEG	NEG	NEG
UKD-0947486	SCCmec IVa (MW2)	CC1	NEG	NEG	NEG	NEG	NEG	NEG	NEG	NEG	NEG	NEG
UKD-1031910	SCCmec IVa (MW2)	CC1	NEG	NEG	NEG	NEG	NEG	NEG	NEG	NEG	NEG	NEG
UKD-14571328	SCCmec IVa (MW2)	CC1	POS	NEG	NEG	NEG	NEG	NEG	NEG	NEG	NEG	NEG
UKD-03003012	SCCmec IVa (MW2)	CC5	NEG	NEG	POS	POS	POS	POS	POS	POS	POS	NEG
UKD-15582582	SCCmec IVa (MW2)	CC6	NEG	NEG	NEG	NEG	NEG	NEG	NEG	NEG	NEG	NEG
UKD-16574230	SCCmec IVa (MW2)	CC6	NEG	NEG	NEG	NEG	NEG	NEG	NEG	NEG	NEG	NEG
UKD-16582560	SCCmec IVa (MW2)	CC6	NEG	NEG	NEG	NEG	NEG	NEG	NEG	NEG	NEG	NEG
UKD-0802855	SCCmec IVa (MW2)	CC7	NEG	NEG	NEG	NEG	NEG	NEG	NEG	NEG	NEG	NEG
KHDN-10405258	SCCmec IVa (MW2)	CC8	POS	NEG	NEG	NEG	NEG	NEG	NEG	NEG	NEG	NEG
UKD-15573497	SCCmec IVa (MW2)	CC22	NEG	NEG	POS	POS	POS	POS	POS	POS	POS	NEG
UKD-0634729	SCCmec IVa (MW2)	CC30	NEG	NEG	POS	POS	POS	POS	POS	POS	POS	NEG
UKD-1119771	SCCmec IVa (MW2)	CC30	NEG	NEG	POS	POS	POS	POS	POS	POS	POS	NEG
KHDN-10411458	SCCmec IVa (MW2)	CC30	NEG	NEG	POS	POS	POS	POS	POS	POS	POS	NEG
KHDN-398609	SCCmec IVa (MW2)	CC30	NEG	NEG	POS	POS	POS	POS	POS	POS	POS	NEG
KHDN-01081998	SCCmec IVa (MW2)	CC30	NEG	NEG	POS	POS	POS	POS	POS	POS	POS	NEG
KHDN-10399223	SCCmec IVa (MW2)	CC30	NEG	NEG	POS	POS	POS	POS	POS	POS	POS	NEG
KHDN-W261158	SCCmec IVa (MW2)	CC30	NEG	NEG	POS	POS	POS	POS	POS	POS	POS	NEG
UKD-0002046	SCCmec IVa (MW2)	CC45 [agr I]	NEG	NEG	POS	NEG	POS	POS	POS	POS	POS	NEG
UKD-0029618	SCCmec IVa (MW2)	CC45 [agr I]	NEG	NEG	POS	POS	POS	POS	POS	POS	POS	NEG
UKD-0043085	SCCmec IVa (MW2)	CC45 [agr I]	NEG	NEG	POS	POS	POS	POS	POS	POS	POS	NEG
UKD-0047053	SCCmec IVa (MW2)	CC45 [agr I]	NEG	NEG	POS	NEG	POS	POS	POS	POS	POS	NEG
UKD-0047429	SCCmec IVa (MW2)	CC45 [agr I]	NEG	NEG	POS	POS	POS	POS	POS	POS	POS	NEG
UKD-0112465	SCCmec IVa (MW2)	CC45 [agr I]	NEG	NEG	POS	NEG	POS	POS	POS	POS	POS	NEG
UKD-0103483	SCCmec IVa (MW2)	CC45 [agr I]	NEG	NEG	POS	NEG	POS	POS	POS	POS	POS	NEG
UKD-0305014	SCCmec IVa (MW2)	CC45 [agr I]	NEG	NEG	POS	POS	POS	POS	POS	POS	POS	NEG
UKD-0401119	SCCmec IVa (MW2)	CC45 [agr I]	NEG	NEG	POS	NEG	POS	POS	POS	POS	POS	NEG
UKD-0537969	SCCmec IVa (MW2)	CC45 [agr I]	NEG	NEG	POS	NEG	POS	POS	POS	POS	POS	NEG
UKD-0703373	SCCmec IVa (MW2)	CC45 [agr I]	NEG	NEG	POS	NEG	POS	POS	POS	POS	POS	NEG
UKD-0813653	SCCmec IVa (MW2)	CC45 [agr I]	NEG	NEG	POS	POS	POS	POS	POS	POS	POS	NEG
UKD-0839175	SCCmec IVa (MW2)	CC45 [agr I]	NEG	NEG	POS	NEG	POS	POS	POS	POS	POS	NEG
UKD-1108422	SCCmec IVa (MW2)	CC45 [agr I]	NEG	NEG	POS	NEG	POS	POS	POS	POS	POS	NEG
UKD-1380645	SCCmec IVa (MW2)	CC45 [agr I]	NEG	NEG	POS	POS	POS	POS	POS	POS	POS	NEG
UKD-148016	SCCmec IVa (MW2)	CC45 [agr I]	NEG	NEG	POS	POS	POS	POS	POS	POS	POS	NEG
UKD-16571773	SCCmec IVa (MW2)	CC45 [agr I]	NEG	NEG	POS	POS	POS	POS	POS	POS	POS	NEG
KHDN-10407738	SCCmec IVa (MW2)	CC45 [ST617]	NEG	NEG	AMB	NEG	POS	POS	NEG	POS	POS	NEG
UKD-0853670	SCCmec IVa (MW2)	CC88	NEG	NEG	NEG	NEG	NEG	NEG	NEG	NEG	NEG	NEG
UKD-1417352	SCCmec IVa (MW2)	CC88	NEG	NEG	NEG	NEG	NEG	NEG	NEG	NEG	NEG	NEG
UKD-15571283	SCCmec IVa (MW2)	CC88	NEG	NEG	NEG	NEG	NEG	NEG	NEG	NEG	NEG	NEG
KHDN-10406719	SCCmec IVa (MW2)	ST93	NEG	NEG	NEG	NEG	NEG	NEG	NEG	NEG	NEG	POS
KHDN-0060294	SCCmec IVa (MW2)	ST93	NEG	NEG	NEG	NEG	NEG	NEG	NEG	NEG	NEG	POS
UKD-0734190	SCCmec IVa (MW2)	CC97	NEG	NEG	NEG	NEG	NEG	NEG	NEG	NEG	NEG	NEG
UKD-13006983	SCCmec IVa (MW2)	CC97	NEG	NEG	NEG	NEG	NEG	NEG	NEG	NEG	NEG	NEG
Prediction for H131520133	SCCmec IVa (H131520133)	CC152	NEG	NEG	NEG	NEG	NEG	NEG	NEG	NEG	NEG	NEG
KHDN-325892	SCCmec IVa (H131520133)	CC30	NEG	NEG	AMB	POS	POS	POS	POS	POS	POS	NEG
UKD-15580207	SCCmec IVa (H131520133)	CC88	POS	NEG	NEG	NEG	NEG	NEG	NEG	NEG	NEG	NEG
UKD-0521159	SCCmec Iva	CC8	POS	NEG	NEG	NEG	NEG	NEG	NEG	NEG	NEG	NEG
Prediction for CMFT503 : HF569113.1	SCCmec IVa (CMFT503)											
Prediction for 21343, GenBank AHKV	SCCmec IVa (CMFT503)	CC88	NEG	NEG	NEG	NEG	NEG	NEG	NEG	NEG	NEG	NEG
UKD-1420740	SCCmec IVa (CMFT503)	CC22	NEG	NEG	AMB	POS	POS	POS	POS	POS	POS	NEG
UKD-15575124	SCCmec IVa (CMFT503)	CC22	NEG	NEG	POS	POS	POS	POS	POS	POS	POS	NEG
UKD-16584947	SCCmec IVa (CMFT503)	CC22	NEG	NEG	POS	POS	POS	POS	POS	POS	POS	NEG
UKD-16575077	SCCmec IVa (CMFT503)	CC88	NEG	NEG	NEG	NEG	NEG	NEG	NEG	NEG	NEG	NEG
Prediction for TCH1516, GenBank CP000730.1	SCCmec IVa+ACME1+Cu (USA300)	CC8	POS	NEG	NEG	NEG	NEG	NEG	NEG	NEG	NEG	NEG
Reference strain USA300-TCH1516	SCCmec IVa+ACME1+Cu (USA300)	CC8	POS	NEG	NEG	NEG	NEG	NEG	NEG	NEG	NEG	NEG
Prediction for FPR3757, GenBank CP000255.1	SCCmec IVa+ACME1+Cu (USA300)	CC8	POS	NEG	NEG	NEG	NEG	NEG	NEG	NEG	NEG	NEG
UKD-0516386	SCCmec IVa+ACME1+Cu (USA300)	CC8	POS	NEG	NEG	NEG	NEG	NEG	NEG	NEG	NEG	NEG
UKD-0815773	SCCmec IVa+ACME1+Cu (USA300)	CC8	POS	NEG	NEG	NEG	NEG	NEG	NEG	NEG	NEG	NEG
UKD-1414654	SCCmec IVa+ACME1+Cu (USA300)	CC8	POS	NEG	NEG	NEG	NEG	NEG	NEG	NEG	NEG	NEG
UKD-0408036	SCCmec IVa+ACME2	CC45 [agr I]	NEG	NEG	AMB	NEG	POS	POS	POS	POS	POS	NEG
UKD-0044035	SCCmec IVa+ccrC (Hannover)	CC8	POS	NEG	NEG	NEG	NEG	NEG	NEG	NEG	NEG	NEG
UKD-0136287	SCCmec IVa+ccrC (Hannover)	CC8	POS	NEG	NEG	NEG	NEG	NEG	NEG	NEG	NEG	NEG

Isolate ID	VIRULENCE : HLG AND LEUKOCIDINS													VIRULENCE : HAEMOLYSINS			
	haemo-lysin gamma / leuko- cidin, component B	haemolysin gamma / leukocidin, component C		haemolysin gamma, component A	Panton Valentine leukocidin F component	Panton Valentine leukocidin S component	leukocidin from ruminant strains		leukocidin D component	leukocidin E component	leukocidin/ haemolysin toxin family protein			putative membrane protein	haemolysin alpha	putative membrane protein	
	lukF	lukS	lukS (ST22+ST45)	hlgA	lukF-PV	lukS-PV	lukF-PV (P83)	lukM	lukD	lukE	lukX	lukY	lukY (ST30+ST45)	corB (=hl)	hla	hlIII (cons)	hlIII (other than RF122)
Prediction for MW2, GenBank BA000033.2	POS	POS	AMB	POS	POS	POS	NEG	NEG	POS	POS	POS	POS	NEG	POS	POS	POS	POS
Reference strain MW2	POS	POS	NEG	POS	POS	POS	NEG	NEG	POS	POS	POS	POS	NEG	POS	POS	POS	POS
Reference strain CA05 (JCSC1968)	POS	POS	POS	POS	POS	POS	NEG	NEG	NEG	NEG	POS	NEG	POS	POS	POS	POS	NEG
UKD-0127106	POS	POS	AMB	POS	NEG	NEG	NEG	NEG	POS	POS	POS	POS	NEG	POS	POS	POS	POS
UKD-0701446	POS	POS	AMB	POS	NEG	NEG	NEG	NEG	POS	POS	POS	POS	NEG	POS	POS	POS	POS
UKD-0947486	POS	POS	AMB	POS	NEG	NEG	NEG	NEG	POS	POS	POS	POS	NEG	POS	POS	POS	POS
UKD-1031910	POS	POS	AMB	POS	NEG	NEG	NEG	NEG	POS	POS	POS	POS	NEG	POS	POS	POS	POS
UKD-14571328	POS	POS	POS	POS	NEG	NEG	NEG	NEG	POS	POS	POS	POS	NEG	POS	POS	POS	POS
UKD-03003012	POS	POS	POS	POS	NEG	NEG	NEG	NEG	POS	POS	AMB	POS	NEG	POS	POS	POS	POS
UKD-15582582	POS	POS	POS	POS	NEG	NEG	NEG	NEG	POS	POS	POS	POS	NEG	POS	POS	POS	POS
UKD-16574230	POS	POS	POS	POS	NEG	NEG	NEG	NEG	POS	POS	POS	POS	NEG	POS	POS	POS	POS
UKD-16582560	POS	POS	NEG	POS	NEG	NEG	NEG	NEG	POS	POS	POS	POS	NEG	POS	POS	POS	POS
UKD-0802855	POS	NEG	POS	POS	NEG	NEG	NEG	NEG	POS	POS	POS	POS	NEG	POS	POS	POS	POS
KHDN-10405258	POS	POS	POS	POS	POS	POS	NEG	NEG	POS	POS	POS	POS	NEG	POS	POS	POS	POS
UKD-15573497	POS	POS	POS	POS	POS	POS	NEG	NEG	NEG	NEG	POS	POS	NEG	POS	POS	NEG	NEG
UKD-0634729	POS	POS	POS	POS	POS	POS	NEG	NEG	NEG	NEG	POS	NEG	POS	POS	POS	POS	POS
UKD-1119771	POS	POS	POS	POS	POS	POS	NEG	NEG	NEG	NEG	POS	NEG	POS	POS	POS	POS	POS
KHDN-10411458	POS	POS	POS	POS	POS	POS	NEG	NEG	NEG	NEG	POS	NEG	POS	POS	POS	POS	POS
KHDN-398609	POS	POS	POS	POS	POS	POS	NEG	NEG	NEG	NEG	POS	NEG	POS	POS	POS	POS	POS
KHDN-01081998	POS	POS	POS	POS	POS	POS	NEG	NEG	NEG	NEG	POS	NEG	POS	POS	POS	POS	POS
KHDN-10399223	POS	POS	POS	POS	POS	POS	AMB	NEG	NEG	NEG	POS	NEG	POS	POS	POS	POS	POS
KHDN-W261158	POS	POS	AMB	POS	POS	POS	NEG	NEG	NEG	NEG	POS	NEG	POS	POS	POS	POS	POS
UKD-0002046	POS	AMB	POS	POS	NEG	NEG	NEG	NEG	NEG	NEG	POS	NEG	POS	POS	POS	POS	NEG
UKD-0029618	POS	AMB	POS	POS	NEG	NEG	NEG	NEG	NEG	NEG	POS	NEG	POS	POS	POS	POS	NEG
UKD-0043085	POS	POS	POS	POS	NEG	NEG	NEG	NEG	NEG	NEG	POS	NEG	POS	POS	POS	POS	NEG
UKD-0047053	POS	NEG	POS	POS	NEG	NEG	NEG	NEG	NEG	NEG	AMB	NEG	POS	POS	POS	POS	NEG
UKD-0047429	POS	AMB	POS	POS	NEG	NEG	NEG	NEG	NEG	NEG	POS	NEG	POS	POS	POS	POS	NEG
UKD-0112465	POS	NEG	POS	POS	NEG	NEG	NEG	NEG	NEG	NEG	POS	NEG	POS	POS	POS	POS	NEG
UKD-0103483	POS	NEG	POS	POS	NEG	NEG	NEG	NEG	NEG	NEG	POS	NEG	POS	AMB	NEG	POS	NEG
UKD-0305014	POS	POS	POS	POS	NEG	NEG	NEG	NEG	NEG	NEG	POS	NEG	POS	POS	POS	POS	NEG
UKD-0401119	POS	NEG	POS	POS	NEG	NEG	NEG	NEG	NEG	NEG	POS	NEG	POS	POS	POS	POS	NEG
UKD-0537969	POS	NEG	POS	POS	NEG	NEG	NEG	NEG	NEG	NEG	POS	NEG	POS	POS	POS	POS	NEG
UKD-0703373	POS	NEG	POS	POS	NEG	NEG	NEG	NEG	NEG	NEG	AMB	NEG	POS	POS	POS	POS	NEG
UKD-0813653	POS	POS	POS	POS	NEG	NEG	NEG	NEG	NEG	NEG	POS	NEG	POS	POS	POS	POS	NEG
UKD-0839175	POS	AMB	POS	POS	NEG	NEG	NEG	NEG	NEG	NEG	POS	NEG	POS	POS	POS	POS	NEG
UKD-1108422	POS	POS	POS	POS	NEG	NEG	NEG	NEG	NEG	NEG	POS	NEG	POS	POS	POS	POS	NEG
UKD-1380645	POS	POS	POS	POS	NEG	NEG	NEG	NEG	NEG	NEG	POS	NEG	POS	POS	POS	POS	NEG
UKD-148016	POS	POS	POS	POS	NEG	NEG	NEG	NEG	NEG	NEG	POS	NEG	POS	POS	POS	POS	NEG
UKD-16571773	POS	POS	POS	POS	NEG	NEG	NEG	NEG	NEG	NEG	POS	NEG	POS	POS	POS	POS	NEG
KHDN-10407738	POS	POS	POS	POS	NEG	NEG	NEG	NEG	NEG	NEG	POS	NEG	POS	POS	POS	POS	NEG
UKD-0853670	POS	POS	AMB	POS	NEG	NEG	NEG	NEG	POS	POS	AMB	POS	NEG	POS	POS	POS	POS
UKD-1417352	POS	POS	POS	POS	POS	POS	AMB	NEG	POS	POS	POS	POS	NEG	POS	POS	POS	POS
UKD-15571283	POS	POS	POS	POS	POS	POS	AMB	NEG	POS	POS	POS	POS	NEG	POS	POS	POS	POS
KHDN-10406719	NEG	NEG	NEG	POS	POS	POS	NEG	NEG	POS	NEG	POS	POS	NEG	POS	POS	POS	POS
KHDN-0060294	NEG	NEG	NEG	NEG	POS	POS	NEG	NEG	POS	NEG	POS	POS	NEG	POS	POS	POS	POS
UKD-0734190	POS	POS	NEG	POS	NEG	NEG	NEG	NEG	POS	POS	AMB	POS	NEG	POS	POS	POS	POS
UKD-13006983	POS	POS	POS	POS	NEG	NEG	POS	NEG	POS	POS	POS	POS	NEG	POS	POS	POS	POS
Prediction for H131520133	POS	NEG	NEG	NEG	POS	POS	NEG	NEG	AMB	NEG	POS	AMB	NEG	NEG	POS	POS	NEG
KHDN-325892	POS	POS	POS	POS	POS	POS	NEG	NEG	NEG	NEG	POS	NEG	POS	POS	POS	POS	POS
UKD-15580207	POS	POS	POS	POS	POS	POS	NEG	NEG	POS	POS	POS	POS	NEG	POS	POS	POS	POS
UKD-0521159	POS	POS	NEG	POS	NEG	NEG	NEG	NEG	POS	POS	NEG	POS	NEG	POS	POS	POS	POS
Prediction for CMFT503 : HF569113.1																	
Prediction for 21343, GenBank AHKV	POS	POS	AMB	POS	NEG	NEG	NEG	NEG	POS	POS	POS	POS	NEG	POS	POS	POS	POS
UKD-1420740	POS	POS	POS	POS	NEG	NEG	NEG	NEG	NEG	NEG	POS	POS	NEG	POS	POS	AMB	NEG
UKD-15575124	POS	POS	POS	POS	NEG	NEG	NEG	NEG	NEG	NEG	POS	POS	NEG	POS	POS	NEG	NEG
UKD-16584947	POS	NEG	POS	POS	NEG	NEG	NEG	NEG	NEG	NEG	POS	POS	NEG	POS	POS	NEG	NEG
UKD-16575077	POS	POS	POS	POS	NEG	NEG	NEG	NEG	POS	POS	POS	POS	NEG	POS	POS	POS	POS
Prediction for TCH1516, GenBank CP000730.1	POS	POS	AMB	POS	POS	POS	NEG	NEG	POS	POS	POS	POS	NEG	POS	POS	POS	POS
Reference strain USA300-TCH1516	POS	POS	AMB	POS	POS	POS	NEG	NEG	POS	POS	POS	POS	NEG	POS	POS	POS	POS
Prediction for FPR3757, GenBank CP000255.1	POS	POS	AMB	POS	POS	POS	NEG	NEG	POS	POS	POS	POS	NEG	POS	POS	POS	POS
UKD-0516386	POS	POS	NEG	POS	POS	POS	NEG	NEG	POS	POS	NEG	POS	NEG	POS	POS	POS	POS
UKD-0815773	POS	POS	NEG	POS	POS	POS	NEG	NEG	POS	POS	POS	POS	NEG	POS	POS	POS	POS
UKD-1414654	POS	POS	POS	POS	POS	POS	AMB	NEG	POS	POS	POS	POS	NEG	POS	POS	POS	POS
UKD-0408036	POS	POS	POS	POS	NEG	NEG	NEG	NEG	NEG	NEG	POS	NEG	POS	POS	POS	POS	NEG
UKD-0044035	POS	POS	NEG	POS	NEG	NEG	NEG	NEG	POS	POS	NEG	POS	NEG	POS	POS	POS	POS
UKD-0136287	POS	POS	AMB	POS	NEG	NEG	NEG	NEG	POS	POS	POS	POS	NEG	POS	POS	POS	POS

Isolate ID	SCCmec type	Clonal complex	VIRULENCE : ENTEROTOXINS										VIRULENCE : HLG AND LEUKOCIDINS													VIRULENCE : HAEMOLYSINS			
			Entero- toxin Q	Entero- toxin R	egc cluster	Entero- toxin G	Entero- toxin I	Entero- toxin M	Entero- toxin N	Entero- toxin O	Entero- toxin U and/or Y	enterotoxin-like protein ORF CM14	haemo-lysin gamma / leuko- cidin, component B	haemolysin gamma / leukocidin, component C		haemolysin gamma, component A	Panton Valentine leukocidin F component	Panton Valentine leukocidin S component	leukocidin from ruminant strains		leukocidin D component	leukocidin E component	leukocidin/ haemolysin toxin family protein			putative membrane protein	haemolysin alpha	putative membrane protein	
			entQ	entR	egc (total)	seg	sei	selm	seln (consensus)	selo	selu	ORF CM14	lukF	lukS	lukS (ST22+ST45)	hlgA	lukF-PV	lukS-PV	lukF-PV (P83)	lukM	lukD	lukE	lukX	lukY	lukY (ST30+ST45)	corB (=hl)	hla	hlIII (cons)	hlIII (other than RF122)
Prediction for JCSC1978, GenBank AB063173.1	***SCCmec IVb/d/i (JCSC1978/6668/4469)***																												
**Reference strain JCSC1978 (8/6-3P)**	**SCCmec IVb/d/i (JCSC1978/6668/4469)**	**CC8**	**POS**	**POS**	**NEG**	**NEG**	**NEG**	**NEG**	**NEG**	**NEG**	**NEG**	**NEG**	**POS**	**POS**	**POS**	**POS**	**POS**	**POS**	**NEG**	**NEG**	**POS**	**POS**	**POS**	**POS**	**NEG**	**POS**	**POS**	**POS**	**POS**
Prediction for JCSC6668, GenBank AB425823.1	***SCCmec IVb/d/i (JCSC1978/6668/4469)***																												
**Reference strain JCSC4469**	**SCCmec IVb/d/i (JCSC1978/6668/4469)**	**CC5**	**NEG**	**NEG**	**POS**	**POS**	**POS**	**POS**	**POS**	**POS**	**POS**	**NEG**	**POS**	**POS**	**POS**	**POS**	**NEG**	**NEG**	**NEG**	**NEG**	**POS**	**POS**	**POS**	**POS**	**NEG**	**POS**	**POS**	**POS**	**POS**
**UKD-1103429**	SCCmec IVb/d/i (JCSC1978/6668/4469)	CC7	NEG	NEG	NEG	NEG	NEG	NEG	NEG	NEG	NEG	NEG	POS	AMB	POS	POS	NEG	NEG	NEG	NEG	POS	POS	POS	POS	NEG	POS	POS	POS	POS
**UKD-1306141**	SCCmec IVb/d/i (JCSC1978/6668/4469)	CC7	NEG	NEG	NEG	NEG	NEG	NEG	NEG	NEG	NEG	NEG	POS	POS	POS	POS	NEG	NEG	NEG	NEG	POS	POS	POS	POS	NEG	POS	POS	POS	POS
**UKD-0500089**	SCCmec IVb/d/i (JCSC1978/6668/4469)	CC5	NEG	POS	POS	POS	POS	POS	POS	POS	POS	NEG	POS	POS	NEG	POS	NEG	NEG	NEG	NEG	POS	POS	POS	POS	NEG	POS	POS	POS	POS
**UKD-0501416**	SCCmec IVb/d/i (JCSC1978/6668/4469)	CC5	NEG	AMB	POS	POS	POS	POS	POS	NEG	POS	NEG	POS	POS	POS	NEG	NEG	NEG	NEG	NEG	POS	AMB	AMB	NEG	NEG	POS	AMB	POS	POS
**UKD-0934574**	SCCmec IVb/d/i (JCSC1978/6668/4469)	CC5	NEG	NEG	POS	POS	POS	POS	POS	POS	POS	NEG	POS	POS	NEG	POS	NEG	NEG	NEG	NEG	POS	POS	AMB	POS	NEG	POS	POS	POS	POS
**UKD-0746779**	SCCmec IVb/d/i (JCSC1978/6668/4469)	CC45 [agr I]	NEG	NEG	POS	POS	POS	POS	POS	POS	POS	NEG	POS	NEG	POS	POS	NEG	NEG	NEG	NEG	NEG	NEG	POS	NEG	POS	POS	POS	POS	NEG
**UKD-0812518**	SCCmec IVb/d/i (JCSC1978/6668/4469)	CC45 [agr I]	NEG	NEG	POS	POS	POS	POS	POS	POS	POS	NEG	POS	NEG	POS	POS	NEG	NEG	NEG	NEG	NEG	NEG	POS	NEG	POS	POS	POS	POS	NEG
Prediction for 21209, GenBank AGRP	***SCCmec IVb/d/i (21209)***	***CC8***	***POS***	***NEG***	***NEG***	***NEG***	***NEG***	***NEG***	***NEG***	***NEG***	***NEG***	***NEG***	***POS***	***POS***	***AMB***	***POS***	***NEG***	***NEG***	***NEG***	***NEG***	***POS***	***POS***	***POS***	***POS***	***NEG***	***POS***	***POS***	***POS***	***POS***
**UKD-0233579**	SCCmec IVb/d/i (21209)	CC8	POS	NEG	NEG	NEG	NEG	NEG	NEG	NEG	NEG	NEG	POS	POS	POS	POS	NEG	NEG	NEG	NEG	POS	POS	AMB	POS	NEG	POS	POS	POS	POS
**UKD-0504920**	SCCmec IVb/d/i (21209)	CC8	POS	NEG	NEG	NEG	NEG	NEG	NEG	NEG	NEG	NEG	POS	POS	NEG	POS	NEG	NEG	NEG	NEG	POS	POS	POS	POS	NEG	POS	POS	POS	POS
**UKD-0725999**	SCCmecIV b/d/i+Cu	CC30	NEG	NEG	POS	POS	POS	POS	POS	POS	POS	NEG	POS	POS	AMB	POS	POS	POS	NEG	NEG	NEG	NEG	POS	NEG	POS	POS	POS	POS	POS
Prediction for IS-105, GenBank AHLR	***SCCmec IVc (IS-105)***	***CC22***	***NEG***	***NEG***	***POS***	***POS***	***POS***	***POS***	***POS***	***POS***	***POS***	***NEG***	***AMB***	***NEG***	***POS***	***POS***	***POS***	***POS***	***NEG***	***NEG***	***NEG***	***NEG***	***POS***	***POS***	***NEG***	***POS***	***POS***	***NEG***	***NEG***
**UKD-0402421**	SCCmec IVc (IS-105), merA/B+	CC5	NEG	POS	POS	POS	POS	POS	AMB	POS	POS	NEG	POS	POS	POS	POS	NEG	NEG	NEG	NEG	POS	POS	AMB	POS	NEG	POS	POS	POS	POS
**UKD-0731319**	SCCmec IVc (IS-105), merA/B+	CC5	NEG	POS	POS	POS	POS	POS	POS	POS	POS	NEG	POS	POS	AMB	POS	NEG	NEG	NEG	NEG	POS	POS	POS	POS	NEG	POS	POS	POS	POS
**UKD-0733137**	SCCmec IVc (IS-105)	CC8	NEG	POS	NEG	NEG	NEG	NEG	NEG	NEG	NEG	NEG	POS	POS	POS	POS	NEG	NEG	NEG	NEG	POS	POS	POS	POS	NEG	POS	POS	POS	POS
**UKD-0305690**	SCCmec IVc (IS-105)	CC22	NEG	NEG	POS	POS	POS	POS	POS	POS	POS	NEG	NEG	NEG	POS	NEG	NEG	NEG	NEG	NEG	NEG	NEG	POS	POS	NEG	POS	POS	POS	NEG
**UKD-1424706**	SCCmec IVc (IS-105)	CC22	NEG	NEG	AMB	POS	POS	POS	POS	POS	POS	NEG	POS	POS	POS	POS	POS	POS	NEG	NEG	NEG	NEG	POS	POS	NEG	POS	POS	NEG	NEG
Prediction for TCH60, GenBank CP002110.1	***SCCmec IVc (TCH60)***	***CC30***	***NEG***	***NEG***	***POS***	***POS***	***POS***	***POS***	***POS***	***POS***	***POS***	***NEG***	***POS***	***POS***	***AMB***	***POS***	***POS***	***POS***	***NEG***	***NEG***	***NEG***	***NEG***	***POS***	***NEG***	***POS***	***POS***	***POS***	***POS***	***POS***
**Reference strain JCSC4788**	**SCCmec IVc (TCH60)**	**CC8**	**NEG**	**NEG**	**NEG**	**NEG**	**NEG**	**NEG**	**NEG**	**NEG**	**NEG**	**NEG**	**POS**	**POS**	**POS**	**POS**	**NEG**	**NEG**	**NEG**	**NEG**	**POS**	**POS**	**POS**	**POS**	**NEG**	**POS**	**POS**	**POS**	**POS**
**UKD-1318318**	SCCmec IVc (TCH60)	CC5	NEG	POS	POS	POS	POS	POS	POS	POS	POS	NEG	POS	POS	POS	POS	POS	POS	NEG	NEG	POS	POS	POS	POS	NEG	POS	POS	POS	POS
**UKD-14580050**	SCCmec IVc (TCH60)	CC5	NEG	POS	POS	POS	POS	POS	POS	POS	POS	NEG	POS	POS	POS	POS	NEG	NEG	NEG	NEG	POS	POS	POS	POS	NEG	POS	POS	POS	POS
**UKD-08F01314**	SCCmec IVc (TCH60)	CC8	NEG	NEG	NEG	NEG	NEG	NEG	NEG	NEG	NEG	NEG	POS	POS	NEG	POS	NEG	NEG	NEG	NEG	POS	POS	POS	POS	NEG	POS	POS	POS	POS
**KHDN-50361518**	SCCmec IVc (TCH60)	CC8	NEG	NEG	NEG	NEG	NEG	NEG	NEG	NEG	NEG	NEG	POS	POS	POS	POS	NEG	NEG	NEG	NEG	POS	POS	POS	POS	NEG	POS	POS	POS	POS
**UKD-0307912**	SCCmec IVc (TCH60)	CC22	NEG	NEG	POS	POS	POS	POS	POS	POS	POS	NEG	NEG	NEG	POS		NEG	NEG	NEG	NEG		NEG	POS	POS	NEG	POS	POS	NEG	NEG
**UKD-0220390**	SCCmec IVc (TCH60)	CC45 [agr I]	NEG	NEG	POS	POS	POS	POS	POS	AMB	POS	NEG	POS	NEG	POS	POS	NEG	NEG	NEG	NEG	NEG	NEG	POS	NEG	POS	AMB	AMB	POS	NEG
**UKD-0311106**	SCCmec IVc (TCH60)	CC45 [agr I]	NEG	NEG	POS	POS	POS	POS	POS	POS	POS	NEG	POS	POS	POS	POS	NEG	NEG	NEG	NEG	NEG	NEG	POS	NEG	POS	POS	POS	POS	NEG
**UKD-0719855**	SCCmec IVc (TCH60)	CC45 [agr I]	NEG	NEG	POS	POS	POS	POS	POS	POS	POS	NEG	POS	NEG	POS	POS	NEG	NEG	NEG	NEG	NEG	NEG	POS	NEG	POS	POS	POS	POS	NEG
**UKD-0422079**	SCCmec IVc (TCH60)	CC45 [agr I]	NEG	NEG	POS	POS	POS	POS	POS	POS	POS	NEG	POS	NEG	POS	POS	NEG	NEG	NEG	NEG	NEG	NEG	POS	NEG	POS	POS	POS	POS	NEG
**UKD-0409235**	SCCmec IVc (TCH60)	CC80	NEG	NEG	NEG	NEG	NEG	NEG	NEG	NEG	NEG	NEG	POS	POS	POS	POS	POS	POS	NEG	NEG	POS	POS	POS	POS	NEG	POS	POS	POS	POS
**UKD-0409807**	SCCmec IVc (TCH60)	CC80	NEG	NEG	NEG	NEG	NEG	NEG	NEG	NEG	NEG	NEG	POS	POS	POS	POS	POS	POS	NEG	NEG	POS	POS	POS	POS	NEG	POS	POS	POS	POS
**UKD-0416073**	SCCmec IVc (TCH60)	CC80	NEG	NEG	NEG	NEG	NEG	NEG	NEG	NEG	NEG	NEG	POS	POS	NEG	POS	POS	POS	NEG	NEG	POS	POS	POS	POS	NEG	POS	POS	POS	POS
**UKD-0431728**	SCCmec IVc (TCH60)	CC80	NEG	NEG	NEG	NEG	NEG	NEG	NEG	NEG	NEG	NEG	POS	POS	NEG	AMB	POS	POS	NEG	NEG	POS	POS	POS	POS	NEG	POS	POS	POS	POS
**UKD-1001508**	SCCmec IVc (TCH60)	CC80	NEG	NEG	NEG	NEG	NEG	NEG	NEG	NEG	NEG	NEG	POS	POS	AMB	POS	POS	POS	NEG	NEG	POS	POS	POS	POS	NEG	POS	POS	POS	POS
**UKD-1010412**	SCCmec IVc (TCH60)	CC80	NEG	NEG	NEG	NEG	NEG	NEG	NEG	NEG	NEG	NEG	POS	POS	AMB	POS	POS	POS	POS	NEG	POS	POS	POS	POS	NEG	POS	POS	POS	POS
**UKD-12753005**	SCCmec IVc (TCH60)	CC80	NEG	NEG	NEG	NEG	NEG	NEG	NEG	NEG	NEG	NEG	POS	POS	AMB	POS	POS	POS	NEG	NEG	POS	POS	POS	POS	NEG	POS	POS	POS	POS
**UKD-15574809**	SCCmec IVc (TCH60)	CC80	NEG	NEG	NEG	NEG	NEG	NEG	NEG	NEG	NEG	NEG	POS	POS	POS	POS	POS	POS	NEG	NEG	POS	POS	POS	POS	NEG	POS	POS	POS	POS
**KHDN-16120291**	SCCmec IVc (TCH60)	CC88	NEG	NEG	NEG	NEG	NEG	NEG	NEG	NEG	NEG	NEG	POS	POS	NEG	POS	NEG	NEG	NEG	NEG	POS	POS	POS	POS	NEG	POS	POS	POS	POS
Prediction for WW2703/97, GenBank ACSW	***SCCmec IVc (WW2703/97)***	***CC30***	***NEG***	***NEG***	***POS***	***POS***	***POS***	***POS***	***POS***	***POS***	***POS***	***NEG***	***POS***	***POS***	***AMB***	***POS***	***NEG***	***NEG***	***NEG***	***NEG***	***NEG***	***NEG***	***POS***	***NEG***	***POS***	***POS***	***POS***	***POS***	***POS***
**UKD-14580088**	SCCmec IVc (WW2703/97)	CC398	NEG	NEG	NEG	NEG	NEG	NEG	NEG	NEG	NEG	NEG	POS	POS	POS	POS	NEG	NEG	NEG	NEG	NEG	NEG	POS	POS	NEG	POS	POS	POS	POS
Prediction for SA_ST125_MupR, GenBank ASTH	***SCCmec IVc+speG+ccrAB4 (SA_ST125)***	***CC5***	***NEG***	***NEG***	***POS***	***POS***	***POS***	***POS***	***POS***	***POS***	***POS***	***NEG***	***POS***	***POS***	***AMB***	***POS***	***NEG***	***NEG***	***NEG***	***NEG***	***POS***	***POS***	***POS***	***POS***	***NEG***	***POS***	***POS***	***POS***	***POS***
**UKD-0439909**	SCCmec IVc+speG+ccrAB4 (SA_ST125)	CC5	NEG	NEG	POS	POS	POS	POS	POS	POS	POS	NEG	POS	POS	AMB	POS	NEG	NEG	NEG	NEG	POS	POS	POS	POS	NEG	POS	POS	POS	POS
Prediction for MRSA177, GenBank AECP	***SCCmec IVc+Cu/Hg (MRSA177)***		***POS***	***NEG***	***NEG***	***NEG***	***NEG***	***NEG***	***NEG***	***NEG***	***NEG***	***NEG***	***POS***	***POS***	***AMB***	***POS***	***POS***	***POS***	***NEG***	***NEG***	***POS***	***POS***	***POS***	***POS***	***NEG***	***POS***	***POS***	***POS***	***POS***
**UKD-1279734**	SCCmec IVc+Cu/Hg (MRSA177)	CC8	NEG	NEG	NEG	NEG	NEG	NEG	NEG	NEG	NEG	NEG	POS	POS	POS	POS	POS	POS	NEG	NEG	POS	POS	POS	POS	NEG	POS	POS	POS	POS
**UKD-14580899**	SCCmec IVc+Cu/Hg (MRSA177)	CC8	POS	NEG	NEG	NEG	NEG	NEG	NEG	NEG	NEG	NEG	POS	POS	POS	POS	POS	POS	AMB	NEG	POS	POS	POS	POS	NEG	POS	POS	POS	POS
**UKD-16002228**	SCCmec IVc+Cu/Hg (MRSA177)	CC8	NEG	NEG	NEG	NEG	NEG	NEG	NEG	NEG	NEG	NEG	POS	POS	POS	POS	POS	POS	NEG	NEG	POS	POS	POS	POS	NEG	POS	POS	POS	POS
Prediction for HO 5096 0412, GenBank HE681097.1	***SCCmec IVh/j (HO50960412/JCSC6670)***	***CC22***	***NEG***	***NEG***	***POS***	***POS***	***POS***	***POS***	***POS***	***POS***	***POS***	***NEG***	***AMB***	***NEG***	***POS***	***POS***	***NEG***	***NEG***	***NEG***	***NEG***	***NEG***	***NEG***	***POS***	***POS***	***NEG***	***POS***	***POS***	***NEG***	***NEG***
Prediction for JCSC6670, GenBank AB425824.1	***SCCmec IVh/j (HO50960412/JCSC6670)***																												
**Reference strain Dublin_DSH_E1749**	**SCCmec IVh/j (HO50960412/JCSC6670)**		**NEG**	**NEG**	**POS**	**POS**	**POS**	**POS**	**POS**	**POS**	**POS**	**NEG**	**POS**	**POS**	**POS**	**POS**	**NEG**	**NEG**	**NEG**	**NEG**	**NEG**	**NEG**	**POS**	**POS**	**NEG**	**POS**	**POS**	**NEG**	**NEG**
**UKD-14576248**	SCCmec IVh/j (HO50960412/JCSC6670)	CC8	NEG	NEG	NEG	NEG	NEG	NEG	NEG	NEG	NEG	NEG	POS	POS	POS	POS	NEG	NEG	NEG	NEG	POS	POS	POS	POS	NEG	POS	POS	POS	POS
**UKD-0120122**	SCCmec IVh/j (HO50960412/JCSC6670)	CC22	NEG	NEG	POS	AMB	POS	POS	POS	POS	POS	NEG	NEG	NEG	POS	AMB	NEG	NEG	NEG	NEG	NEG	NEG	POS	POS	NEG	POS	NEG	NEG	NEG
**UKD-0205319**	SCCmec IVh/j (HO50960412/JCSC6670)	CC22	NEG	NEG	POS	POS	POS	POS	POS	POS	POS	NEG	POS	AMB	POS	POS	NEG	NEG	NEG	NEG	NEG	NEG	POS	POS	NEG	POS	POS	NEG	NEG
**UKD-0221259**	SCCmec IVh/j (HO50960412/JCSC6670)	CC22	NEG	NEG	POS	POS	POS	POS	POS	POS	POS	NEG	POS	AMB	POS	POS	NEG	NEG	NEG	NEG	NEG	NEG	POS	POS	NEG	POS	POS	NEG	NEG
**UKD-0418654**	SCCmec IVh/j (HO50960412/JCSC6670)	CC22	NEG	NEG	POS	POS	POS	POS	POS	POS	POS	NEG	AMB	AMB	POS	POS	NEG	NEG	NEG	NEG	NEG	NEG	POS	POS	NEG	POS	POS	NEG	NEG
**UKD-0429721**	SCCmec IVh/j (HO50960412/JCSC6670)	CC22	NEG	NEG	POS	POS	POS	POS	POS	AMB	POS	NEG	NEG	NEG	POS	NEG	NEG	NEG	NEG	NEG	NEG	NEG	POS	POS	NEG	POS	NEG	NEG	NEG
**UKD-1116194**	SCCmec IVh/j (HO50960412/JCSC6670)	CC22	NEG	NEG	POS	POS	POS	POS	POS	POS	POS	NEG	POS	POS	POS	POS	NEG	NEG	NEG	NEG	NEG	NEG	POS	POS	NEG	POS	POS	NEG	NEG
**UKD-1122367**	SCCmec IVh/j (HO50960412/JCSC6670)	CC22	NEG	NEG	POS	POS	POS	POS	POS	POS	POS	NEG	POS	POS	POS	POS	NEG	NEG	NEG	NEG	NEG	NEG	POS	POS	NEG	POS	POS	NEG	NEG
**UKD-14012840**	SCCmec IVh/j (HO50960412/JCSC6670)	CC22	NEG	NEG	AMB	POS	POS	POS	POS	POS	POS	NEG	POS	POS	POS	POS	NEG	NEG	NEG	NEG	NEG	NEG	POS	POS	NEG	POS	POS	NEG	NEG
**UKD-14579833**	SCCmec IVh/j (HO50960412/JCSC6670)	CC22	NEG	NEG	POS	POS	POS	POS	POS	POS	POS	NEG	POS	POS	POS	POS	NEG	NEG	NEG	NEG	NEG	NEG	POS	POS	NEG	POS	POS	NEG	NEG
**UKD-15579198**	SCCmec IVh/j (HO50960412/JCSC6670)	CC22	NEG	NEG	POS	POS	POS	POS	POS	POS	POS	NEG	POS	POS	POS	POS	NEG	NEG	NEG	NEG	NEG	NEG	POS	POS	NEG	POS	POS	POS	NEG
**UKD-4052193**	SCCmec IVh/j (HO50960412/JCSC6670)	CC22	NEG	NEG	POS	POS	POS	POS	POS	POS	POS	NEG	POS	POS	POS	POS	NEG	NEG	NEG	NEG	NEG	NEG	POS	POS	NEG	POS	POS	NEG	NEG
**UKD-124968**	SCCmec IVh/j (HO50960412/JCSC6670)	CC22	NEG	NEG	AMB	POS	POS	POS	POS	POS	POS	NEG	POS	POS	POS	POS	NEG	NEG	NEG	NEG	NEG	NEG	POS	POS	NEG	POS	POS	NEG	NEG
**UKD-14572546**	SCCmec IVh/j (HO50960412/JCSC6670)	CC22	NEG	NEG	POS	POS	POS	POS	POS	POS	POS	NEG	POS	POS	POS	POS	NEG	NEG	NEG	NEG	NEG	NEG	POS	POS	NEG	POS	POS	AMB	NEG
**UKD-14582287**	SCCmec IVh/j (HO50960412/JCSC6670)	CC22	NEG	NEG	POS	POS	POS	POS	POS	POS	POS	NEG	POS	POS	POS	POS	NEG	NEG	NEG	NEG	NEG	NEG	POS	POS	NEG	POS	POS	NEG	NEG
**UKD-14583376**	SCCmec IVh/j (HO50960412/JCSC6670)	CC22	NEG	NEG	POS	POS	POS	POS	POS	POS	POS	NEG	POS	POS	POS	POS	NEG	NEG	NEG	NEG	NEG	NEG	POS	POS	NEG	POS	POS	NEG	NEG
**UKD-14583496**	SCCmec IVh/j (HO50960412/JCSC6670)	CC22	NEG	NEG	AMB	POS	POS	POS	POS	POS	POS	NEG	POS	POS	POS	POS	NEG	NEG	NEG	NEG	NEG	NEG	POS	POS	NEG	POS	POS	NEG	NEG
**UKD-0013324**	SCCmec IVh/j (HO50960412/JCSC6670)	CC22	NEG	NEG	POS	POS	POS	POS	POS	POS	POS	NEG	NEG	NEG	POS	AMB	AMB	POS	NEG	NEG	NEG	NEG	AMB	POS	NEG	POS	NEG	NEG	NEG
**UKD-0125329**	SCCmec IVh/j (HO50960412/JCSC6670)	CC22	NEG	NEG	POS	POS	POS	POS	POS	POS	POS	NEG	POS	AMB	POS	POS	POS	POS	NEG	NEG	NEG	NEG	POS	POS	NEG	POS	NEG	NEG	NEG
**UKD-0215498**	SCCmec IVh/j (HO50960412/JCSC6670)	CC22	NEG	NEG	POS	POS	POS	POS	POS	POS	POS	NEG	AMB	NEG	POS	AMB	POS	POS	NEG	NEG	NEG	NEG	POS	POS	NEG	POS	POS	NEG	NEG
Prediction for M08-0126, GenBank FR753166.1	***SCCmec IVh/j+ACME2 (M08-0126)***	***CC22***																											
**UKD-1379483I**	SCCmec IVh/j+ACME2 (M08-0126)	CC22	NEG	NEG	POS	POS	POS	POS	POS	POS	POS	NEG	POS	POS	POS	POS	NEG	NEG	NEG	NEG	NEG	NEG	POS	POS	NEG	POS	POS	AMB	NEG
**UKD-5150204**	SCCmec IV+speG+Cu/czrC+ccrAB4	CC22	NEG	NEG	POS	POS	POS	POS	POS	POS	POS	NEG	POS	POS	POS	POS	NEG	NEG	NEG	NEG	NEG	NEG	POS	POS	NEG	POS	POS	NEG	NEG
**UKD-15578423**	SCCmec IV+speG+czrC+ccrAB4 var. 1	CC22	NEG	NEG	POS	POS	POS	POS	POS	POS	POS	NEG	POS	POS	POS	POS	NEG	NEG	NEG	NEG	NEG	NEG	POS	POS	NEG	POS	POS	NEG	NEG
**UKD-16582870**	SCCmec IV+speG+czrC+ccrAB4 var. 1	CC22	NEG	NEG	POS	POS	POS	POS	POS	POS	POS	NEG	NEG	NEG	POS	POS	NEG	NEG	NEG	NEG	NEG	NEG	POS	POS	NEG	POS	NEG	NEG	NEG
**UKD-15571940**	SCCmec IV+speG+czrC+ccrAB4 var. 2	CC22	NEG	NEG	POS	POS	POS	POS	POS	POS	POS	NEG	POS	POS	POS	POS	NEG	NEG	NEG	NEG	NEG	NEG	POS	POS	NEG	POS	POS	POS	NEG

Isolate ID	SCCmec type	Clonal complex	VIRULENCE : ENTEROTOXINS										VIRULENCE : HLG AND LEUKOCIDINS														VIRULENCE : HAEMOLYSINS			
			Entero- toxin Q	Entero- toxin R	egc cluster	Entero- toxin G	Entero- toxin I	Entero- toxin M	Entero- toxin N	Entero- toxin O	Entero- toxin U and/or Y	enterotoxin-like protein ORF CM14	haemo-lysin gamma / leuko- cidin, component B	haemolysin gamma / leukocidin, component C		haemolysin gamma, component A	Panton Valentine leukocidin F component	Panton Valentine leukocidin S component	leukocidin from ruminant strains		leukocidin D component	leukocidin E component	leukocidin/ haemolysin toxin family protein			putative membrane protein	haemolysin alpha	putative membrane protein	
			entQ	entR	egc (total)	seg	sei	selm	seln (consensus)	selo	selu	ORF CM14	lukF	lukS	lukS (ST22+ST 45)	hlgA	lukF-PV	lukS-PV	lukF-PV (P83)	lukM	lukD	lukE	lukX	lukY	lukY (ST30+ST 45)	corB (=hl)	hla	hlIII (cons)	hlIII (other than RF122)
Prediction for CN1, GenBank CP003979.1	*SCCmec IVA (CN1)*	*CC8 (ST72)*	*NEG*	*NEG*	*POS*	*POS*	*POS*	*POS*	*POS*	*POS*	*POS*	*NEG*	*POS*	*POS*	*AMB*	*POS*	*NEG*	*NEG*	*NEG*	*NEG*	*POS*	*POS*	*POS*	*POS*	*NEG*	*POS*	*POS*	*POS*	*POS*
UKD-0900871	SCCmec IVA (CN1)	CC8 (ST72)	NEG	NEG	POS	POS	POS	POS	POS	POS	POS	NEG	POS	POS	AMB	POS	NEG	NEG	NEG	NEG	POS	POS	POS	POS	NEG	POS	POS	POS	POS
Prediction for CMFT1723, GenBank HF569096.1	*SCCmec V (Bengal Bay)*																												
KHDN-10405134	SCCmec V (Bengal Bay)	CC1	POS	NEG	NEG	NEG	NEG	NEG	NEG	NEG	NEG	NEG	POS	POS	POS	POS	POS	POS	NEG	NEG	POS	POS	POS	POS	NEG	POS	POS	POS	POS
UKD-0932405	SCCmec V (Bengal Bay)	CC1 (ST573/772)	NEG	NEG	POS	POS	POS	POS	POS	POS	POS	POS	POS	POS	AMB	POS	POS	POS	NEG	NEG	NEG	NEG	POS	POS	NEG	POS	POS	POS	POS
UKD-1003510	SCCmec V (Bengal Bay)	CC1 (ST573/772)	NEG	NEG	POS	POS	POS	POS	POS	POS	POS	POS	POS	POS	AMB	POS	POS	POS	NEG	NEG	NEG	NEG	POS	POS	NEG	POS	POS	POS	POS
UKD-1112420	SCCmec V (Bengal Bay)	CC1 (ST573/772)	NEG	NEG	POS	POS	POS	POS	POS	POS	POS	POS	POS	POS	POS	POS	POS	POS	NEG	NEG	NEG	NEG	POS	POS	NEG	POS	POS	POS	POS
UKD-1275832	SCCmec V (Bengal Bay)	CC1 (ST573/772)	NEG	NEG	POS	POS	POS	POS	POS	POS	POS	POS	POS	POS	POS	POS	POS	POS	NEG	NEG	NEG	NEG	POS	POS	NEG	POS	POS	POS	POS
UKD-13023811	SCCmec V (Bengal Bay)	CC1 (ST573/772)	NEG	NEG	AMB	POS	POS	POS	POS	POS	POS	POS	POS	POS	POS	POS	POS	POS	NEG	NEG	NEG	NEG	POS	POS	NEG	POS	POS	POS	POS
UKD-14572163	SCCmec V	CC5	NEG	POS	POS	POS	POS	POS	POS	POS	POS	NEG	POS	POS	POS	POS	NEG	NEG	AMB	NEG	POS	POS	POS	POS	NEG	POS	POS	POS	POS
KHDN-10421133	SCCmec V+fus	CC97	NEG	NEG	NEG	NEG	NEG	NEG	NEG	NEG	NEG	NEG	POS	POS	POS	POS	NEG	NEG	NEG	NEG	POS	POS	POS	POS	NEG	POS	POS	POS	POS
KHDN-21343541	SCCmec V+fus+tir+ccrAB1	CC1	POS	NEG	NEG	NEG	NEG	NEG	NEG	NEG	NEG	NEG	POS	POS	POS	POS	POS	POS	NEG	NEG	POS	POS	POS	POS	NEG	POS	POS	POS	POS
KHDN-10416893	SCCmec V+czrC+speG+ccrAB4	CC22	NEG	NEG	POS	POS	POS	POS	POS	POS	POS	NEG	POS	POS	POS	POS	NEG	NEG	NEG	NEG	NEG	NEG	POS	POS	NEG	POS	POS	NEG	NEG
Prediction for GR1, GenBank AJLX	*SCCmec VT (GR1)*	*CC361*	*NEG*	*NEG*	*POS*	*POS*	*POS*	*POS*	*POS*	*POS*	*POS*	*NEG*	*POS*	*POS*	*AMB*	*POS*	*NEG*	*NEG*	*NEG*	*NEG*	*POS*	*POS*	*POS*	*POS*	*NEG*	*POS*	*POS*	*POS*	*POS*
UKD-0422872	SCCmec VT (GR1)	CC152	NEG	NEG	NEG	NEG	NEG	NEG	NEG	NEG	NEG	NEG	POS	NEG	NEG	POS	POS	POS	AMB	NEG	POS	NEG	AMB	POS	NEG	NEG	POS	POS	NEG
Prediction for PM1, GenBank BAFA	*SCCmec VT (PM1)*	*CC59*	*POS*	*NEG*	*NEG*	*NEG*	*NEG*	*NEG*	*NEG*	*NEG*	*NEG*	*NEG*	*POS*	*POS*	*AMB*	*POS*	*POS*	*POS*	*NEG*	*NEG*	*NEG*	*NEG*	*POS*	*AMB*	*NEG*	*POS*	*POS*	*POS*	*NEG*
KHDN-000871	SCCmec VT (PM1)	CC45 (agr I)	NEG	NEG	POS	POS	POS	POS	POS	POS	POS	NEG	POS	POS	POS	POS	NEG	NEG	NEG	NEG	NEG	NEG	POS	NEG	POS	POS	POS	POS	NEG
UKD-0725052	SCCmec VT (PM1)	CC59	POS	NEG	NEG	NEG	NEG	NEG	NEG	NEG	NEG	NEG	POS	POS	AMB	POS	POS	POS	NEG	NEG	NEG	NEG	POS	POS	NEG	POS	POS	POS	NEG
UKD-1312138	SCCmec VT (PM1)	CC59	POS	NEG	NEG	NEG	NEG	NEG	NEG	NEG	NEG	NEG	POS	POS	POS	POS	POS	POS	NEG	NEG	NEG	NEG	POS	POS	NEG	POS	POS	POS	NEG
UKD-Z4728	SCCmec VT (PM1)	CC59	POS	NEG	NEG	NEG	NEG	NEG	NEG	NEG	NEG	NEG	POS	POS	POS	POS	POS	POS	NEG	NEG	NEG	NEG	POS	POS	NEG	POS	POS	POS	NEG
UKD-K0199	SCCmec VT unknown var.1	CC7	NEG	NEG	NEG	NEG	NEG	NEG	NEG	NEG	NEG	NEG	POS	NEG	POS	POS	NEG	NEG	NEG	NEG	POS	POS	POS	POS	NEG	POS	POS	POS	POS
UKD-15584619	SCCmec VT unknown var.2	CC30	NEG	NEG	POS	POS	POS	POS	POS	POS	POS	NEG	POS	POS	POS	POS	NEG	NEG	NEG	NEG	NEG	NEG	POS	NEG	POS	POS	POS	POS	POS
UKD-0712568	SCCmec VT unknown var.3	CC45 (agr I)	NEG	NEG	POS	POS	POS	POS	POS	POS	POS	NEG	POS	NEG	POS	POS	NEG	NEG	NEG	NEG	NEG	NEG	POS	NEG	POS	POS	POS	POS	NEG
KHDN-10420296	SCCmec VT+ACME2	CC45 (agr I)	NEG	NEG	AMB	POS	POS	POS	POS	POS	POS	NEG	POS	POS	POS	POS	NEG	NEG	NEG	NEG	NEG	NEG	POS	NEG	POS	POS	POS	POS	NEG
UKD-15577956	SCCmec VT+Cu/czrC+ccrA1	CC398	NEG	NEG	NEG	NEG	NEG	NEG	NEG	NEG	NEG	NEG	POS	POS	POS	POS	NEG	NEG	NEG	NEG	NEG	NEG	POS	POS	NEG	POS	POS	POS	POS
Prediction for S0385, GenBank AM990992	*SCCmec VT+czrC (SO385)*	*CC398*	*NEG*	*NEG*	*NEG*	*NEG*	*NEG*	*NEG*	*NEG*	*NEG*	*NEG*	*NEG*	*POS*	*POS*	*AMB*	*POS*	*NEG*	*NEG*	*NEG*	*NEG*	*NEG*	*NEG*	*POS*	*AMB*	*NEG*	*POS*	*POS*	*POS*	*POS*
UKD-0628560	SCCmec VT+czrC (SO385)	CC8	NEG	NEG	NEG	NEG	NEG	NEG	NEG	NEG	NEG	NEG	POS	POS	NEG	POS	NEG	NEG	NEG	NEG	POS	POS	POS	POS	NEG	POS	POS	POS	POS
UKD-0510018	SCCmec VT+czrC (SO385)	CC398	NEG	NEG	NEG	NEG	NEG	NEG	NEG	NEG	NEG	NEG	POS	POS	NEG	POS	NEG	NEG	NEG	NEG	NEG	NEG	NEG	POS	NEG	POS	POS	POS	POS
UKD-0903558	SCCmec VT+czrC (SO385)	CC398	NEG	NEG	NEG	NEG	NEG	NEG	NEG	NEG	NEG	NEG	POS	POS	NEG	POS	NEG	NEG	NEG	NEG	NEG	NEG	NEG	POS	NEG	POS	POS	POS	POS
UKD-0929815	SCCmec VT+czrC (SO385)	CC398	NEG	NEG	NEG	NEG	NEG	NEG	NEG	NEG	NEG	NEG	POS	POS	AMB	POS	NEG	NEG	NEG	NEG	NEG	NEG	POS	POS	NEG	POS	POS	POS	POS
UKD-0907762	SCCmec VT+czrC (SO385)	CC398	NEG	NEG	NEG	NEG	NEG	NEG	NEG	NEG	NEG	NEG	POS	POS	NEG	POS	NEG	NEG	NEG	NEG	NEG	NEG	AMB	POS	NEG	POS	POS	POS	POS
UKD-1025105	SCCmec VT+czrC (SO385)	CC398	NEG	NEG	NEG	NEG	NEG	NEG	NEG	NEG	NEG	NEG	POS	POS	AMB	AMB	NEG	NEG	NEG	NEG	NEG	NEG	AMB	AMB	NEG	POS	POS	POS	POS
UKD-11780898	SCCmec VT+czrC (SO385)	CC398	NEG	NEG	NEG	NEG	NEG	NEG	NEG	NEG	NEG	NEG	POS	POS	POS	POS	NEG	NEG	NEG	NEG	NEG	NEG	POS	POS	NEG	POS	POS	POS	POS
UKD-1377009	SCCmec VT+czrC (SO385)	CC398	NEG	NEG	NEG	NEG	NEG	NEG	NEG	NEG	NEG	NEG	POS	POS	POS	POS	NEG	NEG	NEG	NEG	NEG	NEG	POS	POS	NEG	POS	POS	POS	POS
UKD-143406	SCCmec VT+czrC (SO385)	CC398	NEG	NEG	NEG	NEG	NEG	NEG	NEG	NEG	NEG	NEG	POS	POS	POS	POS	NEG	NEG	NEG	NEG	NEG	NEG	POS	POS	NEG	POS	POS	POS	POS
UKD-1400363	SCCmec VT+czrC (SO385)	CC398	NEG	NEG	NEG	NEG	NEG	NEG	NEG	NEG	NEG	NEG	POS	POS	POS	POS	NEG	NEG	NEG	NEG	NEG	NEG	POS	POS	NEG	POS	POS	POS	POS
UKD-14571852	SCCmec VT+czrC (SO385)	CC398	NEG	NEG	NEG	NEG	NEG	NEG	NEG	NEG	NEG	NEG	POS	POS	POS	POS	NEG	NEG	NEG	NEG	NEG	NEG	POS	POS	NEG	POS	POS	POS	POS
UKD-14582574	SCCmec VT+czrC (SO385)	CC398	NEG	NEG	NEG	NEG	NEG	NEG	NEG	NEG	NEG	NEG	POS	POS	POS	POS	NEG	NEG	NEG	NEG	NEG	NEG	POS	POS	NEG	POS	POS	POS	POS
UKD-152108	SCCmec VT+czrC (SO385)	CC398	NEG	NEG	NEG	NEG	NEG	NEG	NEG	NEG	NEG	NEG	POS	POS	POS	POS	NEG	NEG	NEG	NEG	NEG	NEG	POS	POS	NEG	POS	POS	POS	POS
UKD-15573321	SCCmec VT+czrC (SO385)	CC398	NEG	NEG	NEG	NEG	NEG	NEG	NEG	NEG	NEG	NEG	POS	POS	POS	POS	NEG	NEG	NEG	NEG	NEG	NEG	POS	POS	NEG	POS	POS	POS	POS
UKD-15584034	SCCmec VT+czrC , ydhK-neg. variant	CC398	NEG	NEG	NEG	NEG	NEG	NEG	NEG	NEG	NEG	NEG	POS	POS	POS	POS	NEG	NEG	NEG	NEG	NEG	NEG	POS	POS	NEG	POS	POS	POS	POS
UKD-0932581	SCCmecVT+fus+czrC	CC22	NEG	NEG	POS	POS	POS	POS	POS	POS	POS	NEG	NEG	NEG	POS	AMB	NEG	NEG	NEG	NEG	NEG	NEG	POS	POS	NEG	POS	AMB	NEG	NEG
UKD-1121994	SCCmecVT+fus+czrC	CC22	NEG	NEG	POS	POS	POS	POS	POS	POS	POS	NEG	POS	POS	POS	POS	NEG	NEG	NEG	NEG	NEG	NEG	POS	POS	NEG	POS	POS	NEG	NEG
UKD-1272252	SCCmecVT+fus+czrC	CC22	NEG	NEG	POS	POS	POS	POS	POS	POS	POS	NEG	POS	POS	POS	POS	NEG	NEG	NEG	NEG	NEG	NEG	POS	POS	NEG	POS	POS	NEG	NEG
Prediction for MRSA18	*SCCmec VI+fus (MRSA18)*																												
UKD-1103380	SCCmec VI+fus (MRSA18)	CC7	NEG	NEG	NEG	NEG	NEG	NEG	NEG	NEG	NEG	NEG	POS	NEG	POS	POS	NEG	NEG	NEG	NEG	POS	POS	POS	POS	NEG	POS	POS	POS	POS
Prediction for M10/0061, GenBank FR823292.1	*SCCmec XI (LGA251/M10-61)*	*CC130*																											
**Reference strain Dublin-DSH_M10 0061**	**SCCmec XI (LGA251/M10-61)**	**CC130**	**NEG**	**NEG**	**NEG**	**NEG**	**NEG**	**NEG**	**NEG**	**NEG**	**NEG**	**NEG**	**POS**	**POS**	**POS**	**POS**	**NEG**	**NEG**	**NEG**	**NEG**	**POS**	**AMB**	**POS**	**POS**	**NEG**	**POS**	**POS**	**POS**	**NEG**
Prediction for LGA251, GenBank FR821779.1	*SCCmec XI (LGA251/M10-61)*	*ST425*																											
UKD-14015835	SCCmec XI (LGA251/M10-61)	CC130	NEG	NEG	NEG	NEG	NEG	NEG	NEG	NEG	NEG	NEG	POS	POS	POS	POS	NEG	NEG	NEG	NEG	POS	NEG	POS	POS	NEG	POS	POS	POS	NEG
UKD-16573252	SCCmec XI (LGA251/M10-61)	CC130	NEG	NEG	NEG	NEG	NEG	NEG	NEG	NEG	NEG	NEG	POS	POS	POS	POS	NEG	NEG	NEG	NEG	POS	NEG	POS	POS	NEG	POS	POS	POS	NEG
UKD-0217002	PseudoSCCmec, class B mec complex+Hg	CC8	POS	NEG	NEG	NEG	NEG	NEG	NEG	NEG	NEG	NEG	POS	POS	NEG	POS	NEG	NEG	NEG	NEG	POS	POS	POS	POS	NEG	POS	POS	POS	POS
UKD-0008510	PseudoSCCmec, class B mec complex+Hg	CC8	POS	NEG	NEG	NEG	NEG	NEG	NEG	NEG	NEG	NEG	POS	POS	NEG	POS	NEG	NEG	NEG	NEG	POS	POS	POS	POS	NEG	POS	POS	POS	POS
UKD-0110962	PseudoSCCmec, class B mec complex+Hg	CC8	POS	NEG	NEG	NEG	NEG	NEG	NEG	NEG	NEG	NEG	POS	POS	POS	POS	NEG	NEG	NEG	NEG	POS	POS	POS	POS	NEG	POS	POS	POS	POS
KHDN-10408593	PseudoSCCmec, class B mec complex+Cu/Hg	CC8	POS	NEG	NEG	NEG	NEG	NEG	NEG	NEG	NEG	NEG	POS	POS	POS	POS	POS	POS	NEG	NEG	POS	POS	POS	POS	NEG	POS	POS	POS	POS
UKD-0007201	PseudoSCCmec, class B mec complex	CC5	NEG	NEG	POS	POS	POS	POS	POS	POS	POS	NEG	POS	POS	AMB	POS	NEG	NEG	NEG	NEG	POS	POS	POS	POS	NEG	POS	POS	POS	POS
UKD-0033204	PseudoSCCmec, class B mec complex	CC5	NEG	NEG	POS	POS	POS	POS	POS	POS	POS	NEG	POS	POS	AMB	POS	NEG	NEG	NEG	NEG	POS	POS	POS	POS	NEG	POS	POS	POS	POS
UKD-0134329	PseudoSCCmec, class B mec complex	CC5	NEG	NEG	POS	NEG	AMB	AMB	POS	NEG	AMB	NEG	AMB	AMB	NEG	AMB	NEG	NEG	NEG	NEG	POS	NEG	AMB	NEG	NEG	AMB	NEG	AMB	AMB
UKD-0806946	PseudoSCCmec class C+As/Cu	CC398	NEG	NEG	NEG	NEG	NEG	NEG	NEG	NEG	NEG	NEG	POS	POS	AMB	POS	NEG	NEG	NEG	NEG	NEG	NEG	AMB	POS	NEG	POS	POS	POS	POS
UKD-0500610	ACME2	CC8	NEG	POS	NEG	NEG	NEG	NEG	NEG	NEG	NEG	NEG	POS	POS	POS	POS	NEG	NEG	NEG	NEG	POS	POS	AMB	POS	NEG	POS	POS	POS	POS
UKD-0610438	ACME2	CC8	NEG	POS	NEG	NEG	NEG	NEG	NEG	NEG	NEG	NEG	POS	POS	POS	POS	NEG	NEG	NEG	NEG	POS	POS	POS	POS	NEG	POS	POS	POS	POS
KHDN-10409344	ACME2	CC8	NEG	NEG	NEG	NEG	NEG	NEG	NEG	NEG	NEG	NEG	POS	POS	POS	POS	NEG	NEG	NEG	NEG	POS	POS	POS	POS	NEG	POS	POS	POS	POS
UKD-0322559	ACME2+speG+czrC+ccrAB4	CC8	NEG	POS	NEG	NEG	NEG	NEG	NEG	NEG	NEG	NEG	POS	POS	AMB	POS	NEG	NEG	NEG	NEG	POS	POS	POS	POS	NEG	POS	POS	POS	POS
UKD-0501998	ACME2+speG+czrC+ccrAB4	CC8	NEG	POS	NEG	NEG	NEG	NEG	NEG	NEG	NEG	NEG	POS	POS	AMB	POS	NEG	NEG	NEG	NEG	POS	POS	NEG	POS	NEG	POS	POS	POS	POS
UKD-0505213	ACME2+speG+czrC+ccrAB4	CC8	NEG	POS	NEG	NEG	NEG	NEG	NEG	NEG	NEG	NEG	POS	POS	POS	POS	NEG	NEG	AMB	NEG	POS	POS	POS	POS	NEG	POS	POS	POS	POS
Prediction for MSSA476, GenBank BX571857.1	*fus+tir+ccrAB1 (MSSA476)*	*CC1*	*POS*	*NEG*	*NEG*	*NEG*	*NEG*	*NEG*	*NEG*	*NEG*	*NEG*	*NEG*	*POS*	*POS*	*AMB*	*POS*	*NEG*	*NEG*	*NEG*	*NEG*	*POS*	*POS*	*POS*	*POS*	*NEG*	*POS*	*POS*	*POS*	*POS*
**Reference strain Sanger 476**	**fus+tir+ccrAB1 (MSSA476)**	**CC1**	**POS**	**NEG**	**NEG**	**NEG**	**NEG**	**NEG**	**NEG**	**NEG**	**NEG**	**NEG**	**POS**	**POS**	**AMB**	**POS**	**NEG**	**NEG**	**NEG**	**NEG**	**POS**	**POS**	**POS**	**POS**	**NEG**	**POS**	**POS**	**POS**	**POS**
UKD-14023914	fus+tir+ccrAB1 (MSSA476)	CC1	POS	NEG	NEG	NEG	NEG	NEG	NEG	NEG	NEG	NEG	POS	POS	POS	POS	NEG	NEG	NEG	NEG	POS	POS	POS	POS	NEG	POS	POS	POS	POS
UKD-K00034	kdp+ccrAB2	CC182	NEG	NEG	POS	POS	POS	POS	POS	POS	POS	NEG	POS	POS	AMB	POS	NEG	NEG	NEG	NEG	NEG	NEG	POS	POS	NEG	POS	POS	POS	NEG
UKD-15580369	As+ccrAB4	CC22	NEG	NEG	POS	POS	POS	POS	POS	POS	POS	NEG	POS	POS	POS	POS	NEG	NEG	NEG	NEG	NEG	NEG	POS	POS	NEG	POS	POS	NEG	NEG
UKD-15570100	ACME3+ccrAB1 (Strain 21342)	CC80	NEG	NEG	NEG	NEG	NEG	NEG	NEG	NEG	NEG	POS	POS	POS	POS	POS	POS	POS	AMB	NEG	POS	POS	POS	POS	NEG	POS	NEG	POS	POS
UKD-1010678	speG+czrC+ccrAB4	CC8	NEG	NEG	NEG	NEG	NEG	NEG	NEG	NEG	NEG	NEG	POS	POS	NEG	POS	NEG	NEG	NEG	NEG	POS	POS	POS	POS	NEG	POS	POS	POS	POS
UKD-1008243	speG+czrC+ccrAB4	CC8	NEG	POS	NEG	NEG	NEG	NEG	NEG	NEG	NEG	NEG	POS	POS	POS	POS	NEG	NEG	NEG	NEG	POS	POS	POS	POS	NEG	POS	POS	POS	POS
UKD-1009430	speG+czrC+ccrAB4	CC8	NEG	POS	NEG	NEG	NEG	NEG	NEG	NEG	NEG	NEG	POS	POS	NEG	POS	NEG	NEG	NEG	NEG	POS	POS	POS	POS	NEG	POS	POS	POS	POS
UKD-12071828	speG+czrC+ccrAB4	CC22	NEG	NEG	POS	POS	POS	POS	POS	POS	POS	NEG	POS	POS	POS	POS	NEG	NEG	NEG	NEG	NEG	NEG	POS	POS	NEG	POS	POS	NEG	NEG
UKD-14011883	speG+czrC+ccrAB4	CC22	NEG	NEG	POS	POS	POS	POS	POS	POS	POS	NEG	POS	POS	POS	POS	NEG	NEG	NEG	NEG	NEG	NEG	POS	POS	NEG	POS	POS	NEG	NEG
UKD-15008293	speG+czrC+ccrAB4	CC22	NEG	NEG	POS	POS	POS	POS	POS	POS	POS	NEG	POS	POS	POS	POS	NEG	NEG	NEG	NEG	NEG	NEG	POS	POS	NEG	POS	POS	NEG	NEG
UKD-1514877	speG+czrC+ccrAB4	CC22	NEG	NEG	POS	POS	POS	POS	POS	POS	POS	NEG	POS	POS	POS	POS	NEG	NEG	NEG	NEG	NEG	NEG	POS	POS	NEG	POS	POS	NEG	NEG
UKD-15580971	speG+czrC+ccrAB4	CC22	NEG	NEG	POS	POS	POS	POS	POS	POS	POS	NEG	POS	POS	POS	POS	NEG	NEG	NEG	NEG	NEG	NEG	POS	POS	NEG	POS	POS	AMB	NEG
UKD-15582646	speG+czrC+ccrAB4	CC22	NEG	NEG	POS	POS	POS	POS	POS	POS	POS	NEG	POS	POS	POS	POS	NEG	NEG	NEG	NEG	NEG	NEG	POS	POS	NEG	POS	POS	NEG	NEG

Isolate ID	SCCmec type	Clonal complex	VIRULENCE : HAEMOLYSIN BETA AND HLB-CONV PHAGES							VIRULENCE : OTHER FACTORS						VIRULENCE : PROTEASES									
			haemolysin beta				staphylo-kinase	chemotaxis-inhibiting protein (CHIPS)	Staphyl. Comple-ment inhibitor	exfoliative toxins			epidermal cell differentiation inhibitors			aureolysin			serin-protease A	serin-protease B	serin-protease E	glutamylen dopeptidase	staphopain B, protease	staphopain A (staphylopain A), protease	
			hlb-probe 1	hlb-probe 2	hlb-probe 3	un-truncated hlb	sak	chp	scn	etA	etB	etD	edinA	edinB	edinC	aur (cons)	aur (Other than MRSA252)	aur (MRSA252)	splA	splB	splE	sspA	sspB	sspP (cons)	sspP (other than ST93)
VIRTUAL HYBRIDISATION for COL, GenBank CP000046	*SCCmec I (COL)*	*CC8*	*POS*	*POS*	*POS*	*POS*	*NEG*	*NEG*	*NEG*	*NEG*	*NEG*	*NEG*	*NEG*	*NEG*	*NEG*	*POS*	*POS*	*NEG*	*POS*	*POS*	*POS*	*POS*	*POS*	*POS*	*POS*
Reference strain COL	SCCmec I (COL)	CC8	POS	POS	POS	POS	NEG	NEG	NEG	NEG	NEG	NEG	NEG	NEG	NEG	POS	POS	NEG	POS	POS	POS	POS	POS	POS	POS
UKD-0007063	SCCmec I (COL)	CC5	POS	POS	POS	AMB	POS	POS	POS	NEG	NEG	NEG	NEG	NEG	NEG	POS	POS	NEG	POS	POS	NEG	POS	POS	POS	POS
UKD-0220759	SCCmec I (COL)	CC5	AMB	POS	AMB	NEG	POS	NEG	POS	NEG	NEG	NEG	NEG	NEG	NEG	AMB	POS	NEG	POS	POS	NEG	POS	POS	POS	POS
UKD-0308986	SCCmec I (COL)	CC5	POS	POS	AMB	NEG	POS	AMB	POS	NEG	NEG	NEG	NEG	NEG	NEG	POS	POS	NEG	POS	POS	NEG	POS	POS	POS	POS
UKD-0500009	SCCmec I (COL)	CC5	POS	POS	POS	NEG	POS	NEG	POS	NEG	NEG	NEG	NEG	NEG	NEG	POS	POS	NEG	POS	POS	NEG	POS	POS	POS	POS
UKD-0508483	SCCmec I (COL)	CC5	POS	POS	POS	NEG	POS	NEG	POS	NEG	NEG	NEG	NEG	NEG	NEG	POS	POS	NEG	POS	POS	NEG	POS	POS	POS	POS
UKD-0519001	SCCmec I (COL)	CC5	AMB	POS	AMB	NEG	POS	NEG	POS	NEG	NEG	NEG	NEG	NEG	NEG	AMB	POS	NEG	POS	POS	NEG	POS	POS	POS	POS
UKD-0701876	SCCmec I (COL)	CC5	POS	POS	POS	NEG	POS	NEG	POS	NEG	NEG	NEG	NEG	NEG	NEG	POS	POS	NEG	POS	POS	NEG	POS	POS	POS	POS
UKD-0708865	SCCmec I (COL)	CC5	NEG	POS	NEG	NEG	POS	NEG	AMB	NEG	NEG	NEG	NEG	NEG	NEG	NEG	NEG	NEG	POS	POS	NEG	POS	POS	POS	AMB
UKD-1001782	SCCmec I (COL)	CC5	POS	POS	POS	AMB	POS	AMB	POS	NEG	NEG	NEG	NEG	NEG	NEG	POS	POS	NEG	POS	POS	NEG	POS	POS	POS	POS
Prediction for PSP1996, GenBank ANHU	*SCCmec I (PSP1996)*	*CC8*	*POS*	*POS*	*POS*	*POS*	*NEG*	*NEG*	*POS*	*NEG*	*NEG*	*NEG*	*NEG*	*NEG*	*NEG*	*POS*	*POS*	*NEG*	*POS*	*POS*	*POS*	*POS*	*POS*	*POS*	*POS*
UKD-0261727	SCCmec I (PSP1996)	CC8	POS	POS	POS	NEG	POS	POS	POS	NEG	NEG	NEG	NEG	NEG	NEG	POS	POS	NEG	POS	POS	POS	POS	POS	POS	POS
VIRTUAL HYBRIDISATION for MRSA7 : VIRTUAL HYBRID	*SCCmec I+fus+tirS (Geraldine Clone)*	*CC5*	*POS*	*POS*	*AMB*	*POS*	*POS*	*POS*	*POS*	*NEG*	*NEG*	*NEG*	*NEG*	*NEG*	*NEG*	*POS*	*POS*	*NEG*	*POS*	*POS*	*NEG*	*POS*	*POS*	*POS*	*POS*
UKD-1221930	SCCmec I+fus+tirS (Geraldine Clone)	CC5	POS	POS	POS	AMB	POS	POS	POS	NEG	NEG	NEG	NEG	NEG	NEG	POS	POS	NEG	POS	POS	NEG	POS	POS	POS	POS
Prediction for N315, GenBank BA000018.3	*SCCmec II (N315)*	*CC5*	*POS*	*POS*	*AMB*	*POS*	*POS*	*POS*	*POS*	*NEG*	*NEG*	*NEG*	*NEG*	*NEG*	*NEG*	*POS*	*POS*	*NEG*	*POS*	*POS*	*NEG*	*POS*	*POS*	*POS*	*POS*
Reference strain N315	SCCmec II (N315)	CC5	AMB	POS	NEG	NEG	POS	POS	POS	NEG	NEG	NEG	NEG	NEG	NEG	POS	POS	NEG	POS	POS	NEG	POS	POS	POS	POS
Prediction for MRSA252, GenBank BX571856.1	*SCCmec II (N315)*	*CC30*	*POS*	*AMB*	*AMB*	*POS*	*POS*	*POS*	*POS*	*NEG*	*NEG*	*NEG*	*NEG*	*NEG*	*NEG*	*POS*	*NEG*	*POS*	*NEG*	*NEG*	*POS*	*POS*	*POS*	*POS*	*POS*
Reference strain Sanger 252	SCCmec II (N315)	CC30	AMB	AMB	NEG	NEG	POS	POS	POS	NEG	NEG	NEG	NEG	NEG	NEG	POS	NEG	POS	NEG	NEG	POS	POS	POS	POS	POS
UKD-0230792	SCCmec II (N315)	CC30	POS	POS	AMB	NEG	POS	POS	POS	NEG	NEG	NEG	NEG	NEG	NEG	POS	NEG	POS	NEG	NEG	POS	POS	POS	POS	POS
Prediction for Mu50, GenBank BA000017.4	*SCCmec II (N315)*	*CC5*	*POS*	*POS*	*AMB*	*POS*	*POS*	*NEG*	*POS*	*NEG*	*NEG*	*NEG*	*NEG*	*NEG*	*NEG*	*POS*	*POS*	*NEG*	*POS*	*POS*	*NEG*	*POS*	*POS*	*POS*	*POS*
Reference strain MU50	SCCmec II (N315)	CC5	POS	POS	POS	NEG	POS	NEG	POS	NEG	NEG	NEG	NEG	NEG	NEG	POS	POS	NEG	POS	POS	NEG	POS	POS	POS	POS
Prediction for JH1, GenBank CP000736.1	*SCCmec II (JH1/JH9)*	*CC5*	*POS*	*POS*	*AMB*	*POS*	*POS*	*POS*	*POS*	*NEG*	*NEG*	*NEG*	*NEG*	*NEG*	*NEG*	*POS*	*POS*	*NEG*	*POS*	*POS*	*NEG*	*POS*	*POS*	*POS*	*POS*
Prediction for JH9, GenBank CP000703.1	*SCCmec II (JH1/JH9)*	*CC5*	*POS*	*POS*	*AMB*	*POS*	*POS*	*POS*	*POS*	*NEG*	*NEG*	*NEG*	*NEG*	*NEG*	*NEG*	*POS*	*POS*	*NEG*	*POS*	*POS*	*NEG*	*POS*	*POS*	*POS*	*POS*
Reference strain Dublin-DSH_AR07.4_0237	SCCmec II (JH1/JH9)		POS	POS	POS	POS	POS	POS	POS	NEG	NEG	NEG	NEG	NEG	NEG	POS	POS	NEG	POS	POS	NEG	POS	POS	POS	POS
Reference strain NARSA_382 / USA100	SCCmec II (JH1/JH9)	CC5	POS	POS	POS	NEG	POS	POS	POS	NEG	NEG	NEG	NEG	NEG	NEG	POS	POS	NEG	POS	POS	NEG	POS	POS	POS	POS
UKD-0046921	SCCmec II (JH1/JH9)	CC5	POS	POS	POS	NEG	POS	POS	POS	NEG	NEG	NEG	NEG	NEG	NEG	POS	POS	NEG	POS	POS	NEG	POS	POS	POS	POS
UKD-0333415	SCCmec II (JH1/JH9)	CC5	POS	POS	AMB	NEG	POS	POS	POS	NEG	NEG	NEG	NEG	NEG	NEG	POS	POS	NEG	POS	POS	NEG	POS	POS	POS	POS
UKD-0341645	SCCmec II (JH1/JH9)	CC5	POS	POS	POS	NEG	POS	POS	POS	NEG	NEG	NEG	NEG	NEG	NEG	POS	POS	NEG	POS	POS	NEG	POS	POS	POS	POS
UKD-0504626	SCCmec II (JH1/JH9)	CC5	NEG	POS	NEG	NEG	AMB	POS	POS	NEG	NEG	NEG	NEG	NEG	NEG	POS	POS	NEG	POS	POS	NEG	POS	POS	POS	POS
UKD-0507581	SCCmec II (JH1/JH9)	CC5	POS	POS	POS	NEG	POS	POS	POS	NEG	NEG	NEG	NEG	NEG	NEG	POS	POS	NEG	POS	POS	NEG	POS	POS	POS	POS
UKD-0507903	SCCmec II (JH1/JH9)	CC5	POS	POS	AMB	NEG	POS	POS	POS	NEG	NEG	NEG	NEG	NEG	NEG	POS	POS	NEG	POS	POS	NEG	POS	POS	POS	POS
UKD-0700817	SCCmec II (JH1/JH9)	CC5	NEG	POS	NEG	NEG	POS	POS	POS	NEG	NEG	NEG	NEG	NEG	NEG	AMB	POS	NEG	POS	POS	NEG	POS	POS	POS	POS
UKD-1014561	SCCmec II (JH1/JH9)	CC5	POS	POS	POS	NEG	POS	POS	POS	NEG	NEG	NEG	NEG	NEG	NEG	POS	POS	NEG	POS	POS	NEG	POS	POS	POS	POS
UKD-1037507	SCCmec II (JH1/JH9)	CC5	POS	AMB	POS	NEG	POS	NEG	POS	NEG	NEG	NEG	NEG	NEG	NEG	POS	POS	NEG	POS	POS	NEG	POS	POS	POS	POS
UKD-1038321	SCCmec II (JH1/JH9)	CC5	AMB	POS	NEG	NEG	POS	POS	POS	NEG	NEG	NEG	NEG	NEG	NEG	POS	POS	NEG	POS	POS	NEG	POS	POS	POS	POS
UKD-1111608	SCCmec II (JH1/JH9)	CC5	POS	POS	POS	NEG	POS	POS	POS	NEG	NEG	NEG	NEG	NEG	NEG	POS	POS	NEG	POS	POS	NEG	POS	POS	POS	POS
UKD-12753135	SCCmec II (JH1/JH9)	CC5	POS	POS	POS	POS	POS	NEG	NEG	NEG	NEG	NEG	NEG	NEG	NEG	POS	POS	NEG	POS	POS	NEG	POS	POS	POS	POS
UKD-1312816	SCCmec II (JH1/JH9)	CC5	POS	POS	POS	NEG	POS	POS	POS	NEG	NEG	NEG	NEG	NEG	NEG	POS	POS	NEG	POS	POS	NEG	POS	POS	POS	POS
UKD-1401623	SCCmec II (JH1/JH9)	CC5	POS	POS	POS	AMB	POS	POS	POS	NEG	NEG	NEG	NEG	NEG	NEG	POS	POS	NEG	POS	POS	NEG	POS	POS	POS	POS
UKD-15571943	SCCmec II+czrC+ccrA/B-4	CC5	POS	POS	POS	POS	POS	POS	POS	NEG	NEG	NEG	NEG	NEG	NEG	POS	POS	NEG	POS	POS	NEG	POS	POS	POS	POS
UKD-15576888	SCCmec II+czrC+ccrA/B-4	CC5	POS	POS	POS	POS	POS	POS	POS	NEG	NEG	NEG	NEG	NEG	NEG	POS	POS	NEG	POS	POS	NEG	POS	POS	POS	POS
UKD-14576461	SCCmec II+speG+czrC+ccrA/B-4	CC5	POS	POS	POS	AMB	POS	POS	POS	NEG	NEG	NEG	NEG	NEG	NEG	POS	POS	NEG	POS	POS	NEG	POS	POS	POS	POS
UKD-15581649	SCCmec II+speG+czrC+ccrA/B-4	CC5	POS	POS	POS	POS	POS	POS	POS	NEG	NEG	NEG	NEG	NEG	NEG	POS	POS	NEG	POS	POS	NEG	POS	POS	POS	POS
Prediction for CIGC345D, GenBank AHVO	*SCCmec II A/B/D*	*CC8*	*POS*	*POS*	*POS*	*POS*	*POS*	*NEG*	*POS*	*NEG*	*NEG*	*NEG*	*NEG*	*NEG*	*NEG*	*POS*	*POS*	*NEG*	*NEG*	*POS*	*POS*	*POS*	*POS*	*POS*	*POS*
UKD-12K00171	SCCmec II A/B/D	CC8	POS	POS	POS	POS	POS	NEG	POS	NEG	NEG	NEG	NEG	NEG	NEG	POS	POS	NEG	NEG	POS	POS	POS	POS	POS	POS
Prediction for JKD6008, GenBank CP002120.1	*SCC [mec III+Cd] (JKD6008)*	*CC8 (ST239)*	*POS*	*POS*	*POS*	*POS*	*POS*	*NEG*	*POS*	*NEG*	*NEG*	*NEG*	*NEG*	*NEG*	*NEG*	*POS*	*NEG*	*POS*	*NEG*	*NEG*	*NEG*	*POS*	*POS*	*POS*	*POS*
Prediction for JKD6009, GenBank ABSA	*SCC [mec III+Cd] (JKD6008)*	*CC8 (ST239)*	*POS*	*POS*	*POS*	*POS*	*POS*	*NEG*	*POS*	*NEG*	*NEG*	*NEG*	*NEG*	*NEG*	*NEG*	*POS*	*NEG*	*POS*	*NEG*	*NEG*	*NEG*	*POS*	*POS*	*POS*	*POS*
Reference strain ATCC_33592	SCC [mec III+Cd] (ATCC33592)	CC8 (ST239)	POS	POS	POS	AMB	POS	NEG	POS	NEG	NEG	NEG	NEG	NEG	NEG	POS	NEG	POS	POS	POS	POS	POS	POS	POS	POS
Prediction for PPUKM-775-2009, GenBank AMRE	*SCC [mec III+Cd+ccrC] (PPUKM-775-2009)*	*CC8 (ST239)*	*POS*	*POS*	*POS*	*POS*	*POS*	*NEG*	*POS*	*NEG*	*NEG*	*NEG*	*NEG*	*NEG*	*NEG*	*POS*	*NEG*	*POS*	*POS*	*POS*	*POS*	*POS*	*POS*	*POS*	*POS*
Prediction for T0131, GenBank CP002643.1	*SCC [mec III+Cd+ccrC] (T0131)*	*CC8 (ST239)*	*POS*	*POS*	*POS*	*POS*	*POS*	*NEG*	*POS*	*NEG*	*NEG*	*NEG*	*NEG*	*NEG*	*NEG*	*POS*	*NEG*	*POS*	*POS*	*POS*	*NEG*	*POS*	*POS*	*POS*	*POS*
Prediction for XN108, GenBank CP007447.1	*SCC [mec III+Cd+ccrC] (XN108)*	*CC8 (ST239)*	*POS*	*POS*	*POS*	*POS*	*POS*	*NEG*	*POS*	*NEG*	*NEG*	*NEG*	*NEG*	*NEG*	*NEG*	*POS*	*NEG*	*POS*	*POS*	*POS*	*POS*	*POS*	*POS*	*POS*	*POS*
Prediction for CN79, GenBank ANCJ	*SCC [mec III+Cd+ccrC] (CN79)*	*CC8 (ST239)*	*POS*	*POS*	*POS*	*POS*	*POS*	*NEG*	*POS*	*NEG*	*NEG*	*NEG*	*NEG*	*NEG*	*NEG*	*POS*	*NEG*	*POS*	*POS*	*POS*	*NEG*	*POS*	*POS*	*POS*	*POS*
Prediction for 16K, GenBank BABZ	*SCC [mec III+Cd+ccrC] (CN79)*	*CC8 (ST239)*	*POS*	*POS*	*POS*	*POS*	*POS*	*NEG*	*POS*	*NEG*	*NEG*	*NEG*	*NEG*	*NEG*	*NEG*	*POS*	*NEG*	*POS*	*POS*	*POS*	*NEG*	*POS*	*POS*	*POS*	*POS*
UKD-0835987	SCC [mec III+Cd+ccrC] (CN79)	CC8 (ST239)	POS	POS	AMB	POS	NEG	NEG	NEG	NEG	NEG	NEG	NEG	NEG	NEG	POS	NEG	POS	POS	POS	NEG	POS	POS	POS	POS
UKD-16581769	SCCmec III+Cd+ccrC	CC8 (ST239)	POS	POS	POS	POS	NEG	NEG	NEG	NEG	NEG	NEG	NEG	NEG	NEG	POS	NEG	POS	POS	POS	NEG	POS	POS	POS	POS
Prediction for Bmb9393, GenBank CP005288.1	*SCCmec III+Cd/Hg+ccrC (Bmb9393)*	*CC8 (ST239)*	*POS*	*POS*	*POS*	*POS*	*POS*	*POS*	*POS*	*NEG*	*NEG*	*NEG*	*NEG*	*NEG*	*NEG*	*POS*	*NEG*	*POS*	*POS*	*POS*	*POS*	*POS*	*POS*	*POS*	*POS*
UKD-15574263	SCCmec III+Cd/Hg+ccrC (Bmb9393)	CC8 (ST239)	POS	POS	POS	AMB	POS	NEG	POS	NEG	NEG	NEG	NEG	NEG	NEG	POS	NEG	POS	POS	POS	POS	POS	POS	POS	POS
Prediction for SK1585, GenBank AYLT	*SCCmec III+ccrC+Cd/Hg (SK1585)*	*CC8 (ST239)*	*NEG*	*NEG*	*NEG*	*NEG*	*POS*	*POS*	*POS*	*NEG*	*NEG*	*NEG*	*NEG*	*NEG*	*NEG*	*POS*	*NEG*	*POS*	*NEG*	*NEG*	*NEG*	*POS*	*POS*	*POS*	*POS*
UKD-105861	SCCmec III+ccrC+Cd/Hg (SK1585)	CC8 (ST239)	POS	POS	POS	NEG	POS	NEG	POS	NEG	NEG	NEG	NEG	NEG	NEG	POS	NEG	POS	POS	POS	POS	POS	POS	POS	POS
UKD-134572	SCCmec III+ccrC+Cd/Hg (SK1585)	CC8 (ST239)	POS	POS	POS	NEG	POS	NEG	POS	NEG	NEG	NEG	NEG	NEG	NEG	NEG	NEG	POS	POS	POS	POS	POS	POS	POS	POS
UKD-139143	SCCmec III+ccrC+Cd/Hg (SK1585)	CC8 (ST239)	POS	POS	POS	NEG	POS	NEG	POS	NEG	NEG	NEG	NEG	NEG	NEG	POS	NEG	POS	POS	POS	POS	POS	POS	POS	POS
Prediction for TW20, GenBank FN433596.1	*SCC [mec III+Cd/Hg+ccrC] (TW20)*	*CC8 (ST239)*	*POS*	*POS*	*POS*	*POS*	*POS*	*NEG*	*POS*	*NEG*	*NEG*	*NEG*	*NEG*	*NEG*	*NEG*	*POS*	*NEG*	*POS*	*POS*	*POS*	*POS*	*POS*	*POS*	*POS*	*POS*
Prediction for ATCC BAA-39, GenBank AEEK	*SCC [mec III+Cd/Hg+ccrC] (TW20)*	*CC8 (ST239)*	*POS*	*POS*	*POS*	*POS*	*POS*	*NEG*	*POS*	*NEG*	*NEG*	*NEG*	*NEG*	*NEG*	*NEG*	*POS*	*NEG*	*POS*	*POS*	*POS*	*POS*	*POS*	*POS*	*POS*	*POS*

Isolate ID	SCCmec type	Clonal complex	VIRULENCE : HAEMOLYSIN BETA AND HLB-CONV PHAGES							VIRULENCE : OTHER FACTORS						VIRULENCE : PROTEASES									
			haemolysin beta				staphylo-kinase	chemotaxis-inhibiting protein (CHIPS)	Staphyl. Comple-ment inhibitor	exfoliative toxins			epidermal cell differentiation inhibitors			aureolysin			serin-protease A	serin-protease B	serin-protease E	glutamylendopeptidase	staphopain B, protease	staphopain A (staphylopain A), protease	
			hlb-probe 1	hlb-probe 2	hlb-probe 3	un-truncated hlb	sak	chp	scn	etA	etB	etD	edinA	edinB	edinC	aur (cons)	aur (Other than MRSA252)	aur (MRSA252)	splA	splB	splE	sspA	sspB	sspP (cons)	sspP (other than ST93)
Prediction for MW2, GenBank BA000033.2	*SCCmec IVa (MW2)*	*CC1*	POS	POS	POS	POS	POS	NEG	POS	NEG	NEG	NEG	NEG	NEG	NEG	POS	POS	NEG	POS	POS	NEG	POS	POS	POS	POS
Reference strain MW2	SCCmec IVa (MW2)	CC1	AMB	POS	AMB	NEG	POS	NEG	POS	NEG	NEG	NEG	NEG	NEG	NEG	POS	POS	NEG	POS	POS	NEG	POS	POS	POS	POS
Reference strain CA05 (JCSC1968)	SCCmec IVa (MW2)	CC45	POS	POS	NEG	NEG	POS	POS	POS	NEG	NEG	NEG	NEG	NEG	NEG	POS	NEG	POS	NEG	NEG	NEG	POS	POS	POS	POS
UKD-0127106	SCCmec IVa (MW2)	CC1	POS	POS	POS	NEG	POS	NEG	POS	NEG	NEG	NEG	NEG	NEG	NEG	POS	POS	NEG	POS	POS	POS	POS	POS	POS	POS
UKD-0701446	SCCmec IVa (MW2)	CC1	POS	POS	POS	NEG	POS	NEG	POS	NEG	NEG	NEG	NEG	NEG	NEG	POS	POS	NEG	POS	POS	POS	POS	POS	POS	POS
UKD-0947486	SCCmec IVa (MW2)	CC1	POS	POS	POS	NEG	POS	NEG	POS	NEG	NEG	NEG	NEG	NEG	NEG	POS	POS	NEG	POS	POS	POS	POS	POS	POS	POS
UKD-1031910	SCCmec IVa (MW2)	CC1	POS	POS	POS	NEG	POS	NEG	POS	NEG	NEG	NEG	NEG	NEG	NEG	POS	POS	NEG	POS	POS	NEG	POS	POS	POS	POS
UKD-14571328	SCCmec IVa (MW2)	CC1	POS	POS	POS	NEG	POS	NEG	POS	NEG	NEG	NEG	NEG	NEG	NEG	POS	POS	NEG	POS	POS	NEG	POS	POS	POS	POS
UKD-03003012	SCCmec IVa (MW2)	CC5	POS	POS	POS	AMB	POS	POS	POS	NEG	NEG	NEG	NEG	NEG	NEG	POS	POS	NEG	POS	POS	NEG	POS	POS	POS	POS
UKD-15582582	SCCmec IVa (MW2)	CC6	POS	POS	POS	POS	POS	NEG	POS	NEG	NEG	NEG	NEG	NEG	NEG	POS	POS	NEG	POS	POS	POS	POS	POS	POS	POS
UKD-16574230	SCCmec IVa (MW2)	CC6	POS	POS	POS	AMB	POS	NEG	POS	NEG	NEG	NEG	NEG	NEG	NEG	POS	POS	NEG	POS	POS	POS	POS	POS	POS	POS
UKD-16582560	SCCmec IVa (MW2)	CC6	AMB	POS	NEG	NEG	POS	NEG	POS	NEG	NEG	NEG	NEG	NEG	NEG	POS	POS	NEG	POS	POS	POS	POS	POS	POS	POS
UKD-0802855	SCCmec IVa (MW2)	CC7	AMB	POS	AMB	NEG	POS	NEG	POS	NEG	NEG	NEG	NEG	NEG	NEG	POS	POS	NEG	POS	POS	POS	POS	POS	POS	POS
KHDN-10405258	SCCmec IVa (MW2)	CC8	POS	POS	POS	NEG	POS	POS	POS	NEG	NEG	NEG	NEG	NEG	NEG	POS	POS	NEG	POS	POS	POS	POS	POS	POS	POS
UKD-15573497	SCCmec IVa (MW2)	CC22	POS	POS	POS	POS	POS	POS	POS	NEG	NEG	NEG	NEG	NEG	NEG	POS	NEG	POS	NEG	NEG	NEG	POS	POS	POS	POS
UKD-0634729	SCCmec IVa (MW2)	CC30	POS	POS	POS	NEG	POS	POS	POS	NEG	NEG	NEG	NEG	NEG	NEG	POS	NEG	POS	NEG	NEG	POS	POS	POS	POS	POS
UKD-1119771	SCCmec IVa (MW2)	CC30	POS	POS	POS	AMB	POS	POS	POS	NEG	NEG	NEG	NEG	NEG	NEG	POS	NEG	POS	NEG	NEG	POS	POS	POS	POS	POS
KHDN-10411458	SCCmec IVa (MW2)	CC30	POS	POS	POS	NEG	POS	POS	POS	NEG	NEG	NEG	NEG	NEG	NEG	POS	NEG	POS	NEG	NEG	POS	POS	POS	POS	POS
KHDN-398609	SCCmec IVa (MW2)	CC30	POS	POS	POS	AMB	POS	POS	POS	NEG	NEG	NEG	NEG	NEG	NEG	POS	NEG	POS	NEG	NEG	POS	POS	POS	POS	POS
KHDN-01081998	SCCmec IVa (MW2)	CC30	POS	POS	POS	AMB	POS	POS	POS	NEG	NEG	NEG	NEG	NEG	NEG	POS	NEG	POS	NEG	NEG	POS	POS	POS	POS	POS
KHDN-10399223	SCCmec IVa (MW2)	CC30	POS	POS	POS	AMB	POS	POS	POS	NEG	NEG	NEG	NEG	NEG	NEG	POS	NEG	POS	NEG	NEG	POS	POS	POS	POS	POS
KHDN-W261158	SCCmec IVa (MW2)	CC30	POS	POS	POS	AMB	POS	POS	POS	NEG	NEG	NEG	NEG	NEG	NEG	POS	NEG	POS	NEG	NEG	POS	POS	POS	POS	POS
UKD-0002046	SCCmec IVa (MW2)	CC45 [agr I]	POS	POS	NEG	NEG	POS	POS	POS	NEG	NEG	NEG	NEG	NEG	NEG	POS	NEG	POS	NEG	NEG	NEG	POS	POS	POS	POS
UKD-0029618	SCCmec IVa (MW2)	CC45 [agr I]	AMB	AMB	NEG	NEG	POS	POS	POS	NEG	NEG	NEG	NEG	NEG	NEG	AMB	NEG	POS	NEG	NEG	NEG	POS	POS	POS	POS
UKD-0043085	SCCmec IVa (MW2)	CC45 [agr I]	POS	POS	NEG	NEG	POS	POS	POS	NEG	NEG	NEG	NEG	NEG	NEG	AMB	NEG	POS	NEG	NEG	NEG	POS	POS	POS	POS
UKD-0047053	SCCmec IVa (MW2)	CC45 [agr I]	NEG	NEG	NEG	NEG	POS	POS	POS	NEG	NEG	NEG	NEG	NEG	NEG	NEG	NEG	POS	NEG	NEG	NEG	POS	POS	POS	POS
UKD-0047429	SCCmec IVa (MW2)	CC45 [agr I]	AMB	POS	NEG	NEG	POS	POS	POS	NEG	NEG	NEG	NEG	NEG	NEG	AMB	NEG	POS	NEG	NEG	NEG	POS	POS	POS	POS
UKD-0112465	SCCmec IVa (MW2)	CC45 [agr I]	NEG	AMB	NEG	NEG	POS	POS	POS	NEG	NEG	NEG	NEG	NEG	NEG	AMB	NEG	POS	NEG	NEG	NEG	POS	POS	POS	POS
UKD-0103483	SCCmec IVa (MW2)	CC45 [agr I]	NEG	NEG	NEG	NEG	POS	POS	POS	NEG	NEG	NEG	NEG	NEG	NEG	NEG	NEG	POS	NEG	NEG	NEG	POS	POS	POS	POS
UKD-0305014	SCCmec IVa (MW2)	CC45 [agr I]	POS	POS	NEG	NEG	POS	POS	POS	NEG	NEG	NEG	NEG	NEG	NEG	POS	NEG	POS	NEG	NEG	NEG	POS	POS	POS	POS
UKD-0401119	SCCmec IVa (MW2)	CC45 [agr I]	AMB	AMB	NEG	NEG	POS	POS	POS	NEG	NEG	NEG	NEG	NEG	NEG	AMB	NEG	POS	NEG	NEG	NEG	POS	POS	POS	POS
UKD-0537969	SCCmec IVa (MW2)	CC45 [agr I]	POS	POS	NEG	NEG	POS	POS	POS	NEG	NEG	NEG	NEG	NEG	NEG	POS	NEG	POS	NEG	NEG	NEG	POS	POS	POS	POS
UKD-0703373	SCCmec IVa (MW2)	CC45 [agr I]	NEG	NEG	NEG	NEG	POS	POS	POS	NEG	NEG	NEG	NEG	NEG	NEG	NEG	NEG	POS	NEG	NEG	NEG	POS	POS	POS	POS
UKD-0813653	SCCmec IVa (MW2)	CC45 [agr I]	POS	POS	NEG	NEG	POS	POS	POS	NEG	NEG	NEG	NEG	NEG	NEG	POS	NEG	POS	NEG	NEG	NEG	POS	POS	POS	POS
UKD-0839175	SCCmec IVa (MW2)	CC45 [agr I]	POS	POS	NEG	AMB	POS	POS	POS	NEG	NEG	NEG	NEG	NEG	NEG	POS	NEG	POS	NEG	NEG	NEG	POS	POS	POS	POS
UKD-1108422	SCCmec IVa (MW2)	CC45 [agr I]	POS	POS	NEG	NEG	POS	POS	POS	NEG	NEG	NEG	NEG	NEG	NEG	POS	NEG	POS	NEG	NEG	NEG	POS	POS	POS	POS
UKD-1380645	SCCmec IVa (MW2)	CC45 [agr I]	NEG	NEG	NEG	NEG	POS	POS	POS	NEG	NEG	NEG	NEG	NEG	NEG	POS	NEG	POS	NEG	NEG	NEG	POS	POS	POS	POS
UKD-148016	SCCmec IVa (MW2)	CC45 [agr I]	NEG	NEG	NEG	NEG	POS	POS	POS	NEG	NEG	NEG	NEG	NEG	NEG	POS	NEG	POS	NEG	NEG	NEG	POS	POS	POS	POS
UKD-16571773	SCCmec IVa (MW2)	CC45 [agr I]	NEG	NEG	NEG	POS	POS	POS	POS	NEG	NEG	NEG	NEG	NEG	NEG	POS	NEG	POS	NEG	NEG	NEG	POS	POS	POS	POS
KHDN-10407738	SCCmec IVa (MW2)	CC45 [ST617]	NEG	NEG	NEG	NEG	POS	POS	POS	NEG	NEG	NEG	NEG	NEG	NEG	POS	POS	NEG	NEG	NEG	NEG	POS	POS	POS	POS
UKD-0853670	SCCmec IVa (MW2)	CC88	POS	POS	POS	NEG	POS	AMB	POS	NEG	NEG	NEG	NEG	NEG	NEG	POS	POS	NEG	POS	POS	NEG	POS	POS	POS	POS
UKD-1417352	SCCmec IVa (MW2)	CC88	POS	POS	POS	POS	POS	POS	POS	NEG	NEG	NEG	NEG	NEG	NEG	POS	POS	NEG	POS	POS	NEG	POS	POS	POS	POS
UKD-15571283	SCCmec IVa (MW2)	CC88	POS	POS	POS	POS	POS	POS	POS	NEG	NEG	NEG	NEG	NEG	NEG	POS	POS	NEG	POS	POS	NEG	POS	POS	POS	POS
KHDN-10406719	SCCmec IVa (MW2)	ST93	POS	POS	POS	POS	POS	POS	POS	NEG	NEG	NEG	NEG	NEG	NEG	POS	POS	NEG	POS	NEG	POS	POS	POS	POS	POS
KHDN-0060294	SCCmec IVa (MW2)	ST93	POS	POS	POS	NEG	POS	POS	POS	NEG	NEG	NEG	NEG	NEG	NEG	POS	POS	NEG	POS	NEG	POS	POS	POS	POS	NEG
UKD-0734190	SCCmec IVa (MW2)	CC97	POS	POS	POS	NEG	POS	NEG	POS	NEG	NEG	NEG	NEG	NEG	NEG	POS	POS	NEG	POS	POS	POS	POS	POS	POS	POS
UKD-13006983	SCCmec IVa (MW2)	CC97	POS	POS	POS	POS	POS	NEG	POS	NEG	NEG	NEG	NEG	NEG	NEG	POS	POS	NEG	POS	POS	POS	POS	POS	POS	POS
Prediction for H131520133	*SCCmec IVa (H131520133)*	*CC152*	AMB	NEG	POS	AMB	POS	NEG	POS	NEG	NEG	NEG	NEG	POS	NEG	POS	POS	NEG	NEG	NEG	NEG	NEG	POS	POS	POS
KHDN-325892	SCCmec IVa (H131520133)	CC30	POS	POS	POS	AMB	POS	POS	POS	NEG	NEG	NEG	NEG	NEG	NEG	POS	NEG	POS	NEG	NEG	POS	POS	POS	POS	POS
UKD-15580207	SCCmec IVa (H131520133)	CC88	POS	POS	POS	POS	POS	POS	POS	NEG	NEG	NEG	NEG	NEG	NEG	POS	POS	NEG	POS	POS	NEG	POS	POS	POS	POS
UKD-0521159	SCCmec Iva	CC8	POS	POS	POS	NEG	POS	NEG	POS	NEG	NEG	NEG	NEG	NEG	NEG	POS	POS	NEG	POS	POS	POS	POS	POS	POS	POS
Prediction for CMFT503 : HF569113.1	*SCCmec IVa (CMFT503)*																								
Prediction for 21343, GenBank AHKV	*SCCmec IVa (CMFT503)*	*CC88*	POS	POS	POS	POS	POS	POS	POS	POS	NEG	NEG	NEG	NEG	NEG	POS	POS	NEG	POS	POS	NEG	POS	POS	POS	POS
UKD-1420740	SCCmec IVa (CMFT503)	CC22	POS	POS	POS	POS	POS	POS	POS	NEG	NEG	NEG	NEG	NEG	NEG	POS	NEG	POS	NEG	NEG	NEG	POS	POS	POS	POS
UKD-15575124	SCCmec IVa (CMFT503)	CC22	POS	POS	POS	AMB	POS	POS	POS	NEG	NEG	NEG	NEG	NEG	NEG	POS	NEG	POS	NEG	NEG	NEG	POS	POS	POS	POS
UKD-16584947	SCCmec IVa (CMFT503)	CC22	POS	POS	POS	NEG	POS	POS	POS	NEG	NEG	NEG	NEG	NEG	NEG	POS	NEG	POS	NEG	NEG	NEG	POS	POS	POS	POS
UKD-16575077	SCCmec IVa (CMFT503)	CC88	POS	POS	POS	NEG	POS	POS	POS	NEG	NEG	NEG	NEG	NEG	NEG	POS	POS	NEG	POS	POS	NEG	POS	POS	POS	POS
Prediction for TCH1516, GenBank CP000730.1	*SCCmec IVa+ACME1+Cu (USA300)*	*CC8*	POS	POS	POS	POS	POS	POS	POS	NEG	NEG	NEG	NEG	NEG	NEG	POS	POS	NEG	POS	POS	POS	POS	POS	POS	POS
Reference strain USA300-TCH1516	SCCmec IVa+ACME1+Cu (USA300)	CC8	POS	POS	POS	NEG	POS	POS	POS	NEG	NEG	NEG	NEG	NEG	NEG	POS	POS	NEG	POS	POS	POS	POS	POS	POS	POS
Prediction for FPR3757, GenBank CP000255.1	*SCCmec IVa+ACME1+Cu (USA300)*	*CC8*	POS	POS	POS	POS	POS	POS	POS	NEG	NEG	NEG	NEG	NEG	NEG	POS	POS	NEG	POS	POS	POS	POS	POS	POS	POS
UKD-0516386	SCCmec IVa+ACME1+Cu (USA300)	CC8	POS	POS	POS	NEG	POS	POS	POS	NEG	NEG	NEG	NEG	NEG	NEG	AMB	AMB	NEG	POS	POS	POS	POS	POS	POS	POS
UKD-0815773	SCCmec IVa+ACME1+Cu (USA300)	CC8	POS	POS	POS	NEG	POS	POS	POS	NEG	NEG	NEG	NEG	NEG	NEG	POS	POS	NEG	POS	POS	POS	POS	POS	POS	POS
UKD-1414654	SCCmec IVa+ACME1+Cu (USA300)	CC8	POS	POS	POS	AMB	POS	POS	POS	NEG	NEG	NEG	NEG	NEG	NEG	POS	POS	NEG	POS	POS	POS	POS	POS	POS	POS
UKD-0408036	SCCmec IVa+ACME2	CC45 [agr I]	POS	POS	NEG	NEG	POS	POS	POS	NEG	NEG	NEG	NEG	NEG	NEG	POS	NEG	POS	NEG	NEG	NEG	POS	POS	POS	POS
UKD-0044035	SCCmec IVa+ccrC (Hannover)	CC8	POS	POS	POS	NEG	POS	NEG	POS	NEG	NEG	NEG	NEG	NEG	NEG	POS	POS	NEG	POS	POS	POS	POS	POS	POS	POS
UKD-0136287	SCCmec IVa+ccrC (Hannover)	CC8	POS	POS	POS	NEG	POS	NEG	POS	NEG	NEG	NEG	NEG	NEG	NEG	POS	POS	NEG	POS	POS	POS	POS	POS	POS	POS

Isolate ID	SCCmec type	Clonal complex	VIRULENCE : HAEMOLYSIN BETA AND HLB-CONV PHAGES							VIRULENCE : OTHER FACTORS						VIRULENCE : PROTEASES									
			haemolysin beta				staphylo-kinase	chemotaxis-inhibiting protein (CHIPS)	Staphyl. Comple-ment inhibitor	exfoliative toxins			epidermal cell differentiation inhibitors			aureolysin			serin-protease A	serin-protease B	serin-protease E	glutamylen dopeptidase	staphopain B, protease	staphopain A (staphylopain A), protease	
			hlb-probe 1	hlb-probe 2	hlb-probe 3	un-truncated hlb	sak	chp	scn	etA	etB	etD	edinA	edinB	edinC	aur (cons)	aur (Other than MRSA252)	aur (MRSA252)	splA	splB	splE	sspA	sspB	sspP (cons)	sspP (other than ST93)
Prediction for JCSC1978, GenBank AB063173.1	***SCCmec IVb/d/i (JCSC1978/6668/4469)***																								
**Reference strain JCSC1978 (8/6-3P)**	**SCCmec IVb/d/i (JCSC1978/6668/4469)**	**CC8**	**POS**	**POS**	**POS**	**POS**	**NEG**	**NEG**	**NEG**	**NEG**	**NEG**	**NEG**	**NEG**	**NEG**	**NEG**	**POS**	**POS**	**NEG**	**POS**	**POS**	**POS**	**POS**	**POS**	**POS**	**POS**
Prediction for JCSC6668, GenBank AB425823.1	***SCCmec IVb/d/i (JCSC1978/6668/4469)***																								
**Reference strain JCSC4469**	**SCCmec IVb/d/i (JCSC1978/6668/4469)**	**CC5**	**POS**	**POS**	**POS**	**AMB**	**POS**	**POS**	**POS**	**NEG**	**NEG**	**NEG**	**NEG**	**NEG**	**NEG**	**POS**	**POS**	**NEG**	**POS**	**POS**	**NEG**	**POS**	**POS**	**POS**	**POS**
**UKD-1103429**	SCCmec IVb/d/i (JCSC1978/6668/4469)	CC7	POS	POS	POS	NEG	POS	NEG	POS	NEG	NEG	NEG	NEG	NEG	NEG	POS	POS	NEG	POS	POS	POS	POS	POS	POS	POS
**UKD-1306141**	SCCmec IVb/d/i (JCSC1978/6668/4469)	CC7	POS	POS	POS	AMB	POS	NEG	POS	NEG	NEG	NEG	NEG	NEG	NEG	POS	POS	NEG	POS	POS	POS	POS	POS	POS	POS
**UKD-0500089**	SCCmec IVb/d/i (JCSC1978/6668/4469)	CC5	POS	POS	POS	NEG	POS	POS	POS	NEG	NEG	NEG	NEG	NEG	NEG	POS	POS	NEG	POS	POS	NEG	POS	POS	POS	POS
**UKD-0501416**	SCCmec IVb/d/i (JCSC1978/6668/4469)	CC5	NEG	POS	NEG	NEG	POS	POS	POS	NEG	NEG	NEG	NEG	NEG	NEG	POS	POS	NEG	POS	POS	NEG	POS	POS	POS	POS
**UKD-0934574**	SCCmec IVb/d/i (JCSC1978/6668/4469)	CC5	AMB	POS	NEG	NEG	POS	POS	POS	NEG	NEG	NEG	NEG	NEG	NEG	POS	POS	NEG	POS	POS	NEG	POS	POS	POS	POS
**UKD-0746779**	SCCmec IVb/d/i (JCSC1978/6668/4469)	CC45 [agr I]	AMB	AMB	NEG	NEG	POS	POS	POS	NEG	NEG	NEG	NEG	NEG	NEG	AMB	NEG	POS	NEG	NEG	NEG	POS	POS	POS	POS
**UKD-0812518**	SCCmec IVb/d/i (JCSC1978/6668/4469)	CC45 [agr I]	AMB	POS	NEG	NEG	POS	POS	POS	NEG	NEG	NEG	NEG	NEG	NEG	NEG	NEG	POS	NEG	NEG	NEG	POS	POS	POS	POS
Prediction for 21209, GenBank AGRP	***SCCmec IVb/d/i (21209)***	***CC8***	***POS***	***POS***	***POS***	***POS***	***POS***	***NEG***	***POS***	***NEG***	***NEG***	***NEG***	***NEG***	***NEG***	***NEG***	***POS***	***POS***	***NEG***	***POS***	***POS***	***POS***	***POS***	***POS***	***POS***	***POS***
**UKD-0233579**	SCCmec IVb/d/i (21209)	CC8	POS	POS	POS	NEG	POS	NEG	POS	NEG	NEG	NEG	NEG	NEG	NEG	POS	POS	NEG	POS	POS	POS	POS	POS	POS	POS
**UKD-0504920**	SCCmec IVb/d/i (21209)	CC8	AMB	POS	AMB	POS	NEG	NEG	NEG	NEG	NEG	NEG	NEG	NEG	NEG	POS	POS	NEG	POS	POS	POS	POS	POS	POS	POS
**UKD-0725999**	SCCmecIV b/d/i+Cu	CC30	POS	POS	POS	NEG	POS	NEG	POS	NEG	NEG	NEG	NEG	NEG	NEG	POS	NEG	POS	NEG	NEG	POS	POS	POS	POS	POS
Prediction for IS-105, GenBank AHLR	***SCCmec IVc (IS-105)***	***CC22***	***POS***	***POS***	***POS***	***POS***	***POS***	***POS***	***POS***	***NEG***	***NEG***	***NEG***	***NEG***	***NEG***	***NEG***	***POS***	***NEG***	***POS***	***NEG***	***NEG***	***NEG***	***POS***	***POS***	***POS***	***POS***
**UKD-0402421**	SCCmec IVc (IS-105), merA/B+	CC5	POS	POS	POS	NEG	POS	POS	POS	NEG	NEG	NEG	NEG	NEG	NEG	POS	POS	NEG	POS	POS	NEG	POS	POS	POS	POS
**UKD-0731319**	SCCmec IVc (IS-105), merA/B+	CC5	POS	POS	POS	NEG	POS	POS	POS	NEG	NEG	NEG	NEG	NEG	NEG	POS	POS	NEG	POS	POS	NEG	POS	POS	POS	POS
**UKD-0733137**	SCCmec IVc (IS-105)	CC8	POS	POS	POS	NEG	POS	NEG	POS	NEG	NEG	NEG	NEG	NEG	NEG	POS	POS	NEG	POS	POS	POS	POS	POS	POS	POS
**UKD-0305690**	SCCmec IVc (IS-105)	CC22	POS	POS	POS	NEG	POS	POS	POS	NEG	NEG	NEG	NEG	NEG	NEG	POS	NEG	POS	NEG	NEG	NEG	POS	POS	POS	POS
**UKD-1424706**	SCCmec IVc (IS-105)	CC22	POS	POS	POS	AMB	POS	POS	POS	NEG	NEG	NEG	NEG	NEG	NEG	POS	NEG	POS	NEG	NEG	NEG	POS	POS	POS	POS
Prediction for TCH60, GenBank CP002110.1	***SCCmec IVc (TCH60)***	***CC30***	***NEG***	***NEG***	***NEG***	***POS***	***POS***	***POS***	***POS***	***NEG***	***NEG***	***NEG***	***NEG***	***NEG***	***NEG***	***POS***	***NEG***	***POS***	***NEG***	***NEG***	***POS***	***POS***	***POS***	***POS***	***POS***
**Reference strain JCSC4788**	**SCCmec IVc (TCH60)**	**CC8**	**POS**	**POS**	**POS**	**AMB**	**POS**	**NEG**	**POS**	**NEG**	**NEG**	**NEG**	**NEG**	**NEG**	**NEG**	**POS**	**POS**	**NEG**	**POS**	**POS**	**POS**	**POS**	**POS**	**POS**	**POS**
**UKD-1318318**	SCCmec IVc (TCH60)	CC5	POS	POS	POS	NEG	POS	NEG	POS	NEG	NEG	NEG	NEG	NEG	NEG	POS	POS	NEG	POS	POS	NEG	POS	POS	POS	POS
**UKD-14580050**	SCCmec IVc (TCH60)	CC5	POS	POS	POS	NEG	POS	NEG	POS	NEG	NEG	NEG	NEG	NEG	NEG	POS	POS	NEG	POS	POS	NEG	POS	POS	POS	POS
**UKD-08F01314**	SCCmec IVc (TCH60)	CC8	AMB	POS	NEG	POS	NEG	NEG	NEG	NEG	NEG	NEG	NEG	NEG	NEG	POS	POS	NEG	POS	POS	AMB	POS	POS	POS	POS
**KHDN-50361518**	SCCmec IVc (TCH60)	CC8	POS	POS	POS	NEG	POS	NEG	POS	NEG	NEG	NEG	NEG	NEG	NEG	POS	POS	NEG	POS	POS	POS	POS	POS	POS	POS
**UKD-0307912**	SCCmec IVc (TCH60)	CC22	POS	POS	POS	NEG	POS	POS	POS	NEG	NEG	NEG	NEG	NEG	NEG	POS	NEG	POS	NEG	NEG	NEG	POS	POS	POS	POS
**UKD-0220390**	SCCmec IVc (TCH60)	CC45 [agr I]	NEG	NEG	NEG	NEG	POS	POS	POS	NEG	NEG	NEG	NEG	NEG	NEG	NEG	NEG	POS	NEG	NEG	NEG	POS	POS	POS	POS
**UKD-0311106**	SCCmec IVc (TCH60)	CC45 [agr I]	NEG	NEG	NEG	NEG	POS	POS	POS	NEG	NEG	NEG	NEG	NEG	NEG	POS	NEG	POS	NEG	NEG	NEG	POS	POS	POS	POS
**UKD-0719855**	SCCmec IVc (TCH60)	CC45 [agr I]	NEG	NEG	NEG	NEG	POS	POS	POS	NEG	NEG	NEG	NEG	NEG	NEG	NEG	NEG	POS	NEG	NEG	NEG	POS	POS	POS	POS
**UKD-0422079**	SCCmec IVc (TCH60)	CC45 [agr I]	AMB	POS	NEG	NEG	POS	POS	POS	NEG	NEG	NEG	NEG	NEG	NEG	NEG	NEG	POS	NEG	NEG	NEG	POS	POS	POS	POS
**UKD-0409235**	SCCmec IVc (TCH60)	CC80	POS	POS	POS	NEG	POS	NEG	POS	NEG	NEG	POS	NEG	POS	NEG	POS	POS	NEG	POS	POS	NEG	POS	POS	POS	POS
**UKD-0409807**	SCCmec IVc (TCH60)	CC80	POS	POS	POS	AMB	POS	NEG	POS	NEG	NEG	POS	NEG	POS	NEG	POS	POS	NEG	POS	POS	NEG	POS	POS	POS	POS
**UKD-0416073**	SCCmec IVc (TCH60)	CC80	AMB	POS	NEG	NEG	POS	NEG	POS	NEG	NEG	POS	NEG	POS	NEG	POS	POS	NEG	POS	POS	NEG	POS	POS	POS	POS
**UKD-0431728**	SCCmec IVc (TCH60)	CC80	NEG	POS	NEG	NEG	POS	NEG	POS	NEG	NEG	POS	NEG	POS	NEG	POS	POS	NEG	POS	POS	NEG	POS	POS	POS	POS
**UKD-1001508**	SCCmec IVc (TCH60)	CC80	POS	POS	POS	AMB	POS	NEG	POS	NEG	NEG	POS	NEG	POS	NEG	POS	POS	NEG	POS	POS	NEG	POS	POS	POS	POS
**UKD-1010412**	SCCmec IVc (TCH60)	CC80	AMB	AMB	AMB	AMB	POS	NEG	POS	NEG	NEG	POS	NEG	POS	NEG	POS	POS	NEG	POS	POS	NEG	POS	POS	POS	POS
**UKD-12753005**	SCCmec IVc (TCH60)	CC80	POS	POS	POS	AMB	POS	NEG	POS	NEG	NEG	POS	NEG	POS	NEG	POS	POS	NEG	POS	POS	NEG	POS	POS	POS	POS
**UKD-15574809**	SCCmec IVc (TCH60)	CC80	POS	POS	POS	NEG	POS	NEG	POS	NEG	NEG	POS	NEG	POS	NEG	POS	POS	NEG	POS	POS	NEG	POS	POS	POS	POS
**KHDN-16120291**	SCCmec IVc (TCH60)	CC88	POS	POS	POS	NEG	POS	NEG	POS	NEG	NEG	POS	NEG	POS	NEG	POS	POS	NEG	POS	POS	NEG	POS	POS	POS	POS
Prediction for WW2703/97, GenBank ACSW	***SCCmec IVc (WW2703/97)***	***CC30***	***POS***	***POS***	***POS***	***POS***	***POS***	***NEG***	***POS***	***NEG***	***NEG***	***NEG***	***NEG***	***NEG***	***NEG***	***POS***	***NEG***	***POS***	***NEG***	***NEG***	***POS***	***POS***	***POS***	***POS***	***POS***
**UKD-14580088**	SCCmec IVc (WW2703/97)	CC398	NEG	NEG	NEG	POS	NEG	NEG	NEG	NEG	NEG	NEG	NEG	NEG	NEG	POS	NEG	POS	NEG	NEG	NEG	POS	POS	POS	POS
Prediction for SA_ST125_MupR, GenBank ASTH	***SCCmec IVc+speG+ccrAB4 (SA_ST125)***	***CC5***	***POS***	***POS***	***AMB***	***POS***	***NEG***	***NEG***	***NEG***	***NEG***	***NEG***	***NEG***	***NEG***	***NEG***	***NEG***	***POS***	***POS***	***NEG***	***POS***	***POS***	***NEG***	***POS***	***POS***	***POS***	***POS***
**UKD-0439909**	SCCmec IVc+speG+ccrAB4 (SA_ST125)	CC5	POS	POS	AMB	AMB	POS	NEG	POS	NEG	NEG	NEG	NEG	NEG	NEG	POS	POS	NEG	POS	POS	NEG	POS	POS	POS	POS
Prediction for MRSA177, GenBank AECP	***SCCmec IVc+Cu/Hg (MRSA177)***		***POS***	***POS***	***POS***	***POS***	***POS***	***POS***	***POS***	***NEG***	***NEG***	***NEG***	***NEG***	***NEG***	***NEG***	***POS***	***POS***	***NEG***	***POS***	***POS***	***POS***	***POS***	***POS***	***POS***	***POS***
**UKD-1279734**	SCCmec IVc+Cu/Hg (MRSA177)	CC8	POS	POS	POS	POS	POS	POS	POS	NEG	NEG	NEG	NEG	NEG	NEG	POS	POS	NEG	POS	POS	POS	POS	POS	POS	POS
**UKD-14580899**	SCCmec IVc+Cu/Hg (MRSA177)	CC8	POS	POS	POS	POS	NEG	NEG	NEG	NEG	NEG	NEG	NEG	NEG	NEG	POS	POS	NEG	POS	POS	POS	POS	POS	POS	POS
**UKD-16002228**	SCCmec IVc+Cu/Hg (MRSA177)	CC8	POS	POS	POS	POS	NEG	NEG	NEG	NEG	NEG	NEG	NEG	NEG	NEG	POS	POS	NEG	POS	POS	POS	POS	POS	POS	POS
Prediction for HO 5096 0412, GenBank HE681097.1	***SCCmec IVh/j (HO50960412/JCSC6670)***	***CC22***	***POS***	***POS***	***POS***	***POS***	***POS***	***POS***	***POS***	***NEG***	***NEG***	***NEG***	***NEG***	***NEG***	***NEG***	***POS***	***NEG***	***POS***	***NEG***	***NEG***	***NEG***	***POS***	***POS***	***POS***	***POS***
Prediction for JCSC6670, GenBank AB425824.1	***SCCmec IVh/j (HO50960412/JCSC6670)***																								
**Reference strain Dublin_DSH_E1749**	**SCCmec IVh/j (HO50960412/JCSC6670)**		**POS**	**POS**	**POS**	**NEG**	**POS**	**POS**	**POS**	**NEG**	**NEG**	**NEG**	**NEG**	**NEG**	**NEG**	**POS**	**NEG**	**POS**	**NEG**	**NEG**	**NEG**	**POS**	**POS**	**POS**	**POS**
**UKD-14576248**	SCCmec IVh/j (HO50960412/JCSC6670)	CC8	POS	POS	POS	NEG	POS	NEG	POS	NEG	NEG	NEG	NEG	NEG	NEG	POS	POS	NEG	POS	POS	POS	POS	POS	POS	POS
**UKD-0120122**	SCCmec IVh/j (HO50960412/JCSC6670)	CC22	POS	POS	NEG	NEG	POS	POS	POS	NEG	NEG	NEG	NEG	NEG	NEG	POS	NEG	POS	NEG	NEG	NEG	POS	POS	POS	POS
**UKD-0205319**	SCCmec IVh/j (HO50960412/JCSC6670)	CC22	POS	POS	POS	NEG	POS	POS	POS	NEG	NEG	NEG	NEG	NEG	NEG	POS	NEG	POS	NEG	NEG	NEG	POS	POS	POS	POS
**UKD-0221259**	SCCmec IVh/j (HO50960412/JCSC6670)	CC22	POS	POS	POS	NEG	POS	POS	POS	NEG	NEG	NEG	NEG	NEG	NEG	AMB	NEG	POS	NEG	NEG	NEG	POS	POS	POS	POS
**UKD-0418654**	SCCmec IVh/j (HO50960412/JCSC6670)	CC22	POS	POS	POS	NEG	POS	POS	POS	NEG	NEG	NEG	NEG	NEG	NEG	POS	NEG	POS	NEG	NEG	NEG	POS	POS	POS	POS
**UKD-0429721**	SCCmec IVh/j (HO50960412/JCSC6670)	CC22	POS	POS	NEG	NEG	POS	POS	POS	NEG	NEG	NEG	NEG	NEG	NEG	POS	NEG	POS	NEG	NEG	NEG	POS	POS	POS	POS
**UKD-1116194**	SCCmec IVh/j (HO50960412/JCSC6670)	CC22	POS	POS	POS	POS	NEG	AMB	NEG	NEG	NEG	NEG	NEG	NEG	NEG	POS	NEG	POS	NEG	NEG	NEG	POS	POS	POS	POS
**UKD-1122367**	SCCmec IVh/j (HO50960412/JCSC6670)	CC22	POS	POS	POS	POS	POS	POS	POS	NEG	NEG	NEG	NEG	NEG	NEG	POS	NEG	POS	NEG	NEG	NEG	POS	POS	POS	POS
**UKD-14012840**	SCCmec IVh/j (HO50960412/JCSC6670)	CC22	POS	POS	POS	NEG	POS	POS	POS	NEG	NEG	NEG	NEG	NEG	NEG	POS	NEG	POS	NEG	NEG	NEG	POS	POS	POS	POS
**UKD-14579833**	SCCmec IVh/j (HO50960412/JCSC6670)	CC22	POS	POS	POS	NEG	POS	POS	POS	NEG	NEG	NEG	NEG	NEG	NEG	POS	NEG	POS	NEG	NEG	NEG	POS	POS	POS	POS
**UKD-15579198**	SCCmec IVh/j (HO50960412/JCSC6670)	CC22	POS	POS	POS	POS	POS	POS	POS	NEG	NEG	NEG	NEG	NEG	NEG	POS	NEG	POS	NEG	NEG	NEG	POS	POS	POS	POS
**UKD-4052193**	SCCmec IVh/j (HO50960412/JCSC6670)	CC22	POS	POS	POS	POS	POS	POS	POS	NEG	NEG	NEG	NEG	NEG	NEG	POS	NEG	POS	NEG	NEG	NEG	POS	POS	POS	POS
**UKD-124968**	SCCmec IVh/j (HO50960412/JCSC6670)	CC22	POS	POS	POS	AMB	POS	POS	POS	NEG	NEG	NEG	NEG	NEG	NEG	POS	NEG	POS	NEG	NEG	NEG	POS	POS	POS	POS
**UKD-14572546**	SCCmec IVh/j (HO50960412/JCSC6670)	CC22	POS	POS	POS	AMB	POS	POS	POS	NEG	NEG	NEG	NEG	NEG	NEG	POS	NEG	POS	NEG	NEG	NEG	POS	POS	POS	POS
**UKD-14582287**	SCCmec IVh/j (HO50960412/JCSC6670)	CC22	POS	POS	POS	AMB	POS	POS	POS	NEG	NEG	NEG	NEG	NEG	NEG	POS	NEG	POS	NEG	NEG	NEG	POS	POS	POS	POS
**UKD-14583376**	SCCmec IVh/j (HO50960412/JCSC6670)	CC22	POS	POS	POS	POS	POS	POS	POS	NEG	NEG	NEG	NEG	NEG	NEG	POS	NEG	POS	NEG	NEG	NEG	POS	POS	POS	POS
**UKD-14583496**	SCCmec IVh/j (HO50960412/JCSC6670)	CC22	POS	POS	POS	POS	POS	POS	POS	NEG	NEG	NEG	NEG	NEG	NEG	POS	NEG	POS	NEG	NEG	NEG	POS	POS	POS	POS
**UKD-0013324**	SCCmec IVh/j (HO50960412/JCSC6670)	CC22	POS	POS	AMB	NEG	POS	POS	POS	NEG	NEG	NEG	NEG	NEG	NEG	POS	NEG	POS	NEG	NEG	NEG	POS	POS	POS	POS
**UKD-0125329**	SCCmec IVh/j (HO50960412/JCSC6670)	CC22	POS	POS	POS	NEG	POS	POS	POS	NEG	NEG	NEG	NEG	NEG	NEG	POS	NEG	POS	NEG	NEG	NEG	POS	POS	POS	POS
**UKD-0215498**	SCCmec IVh/j (HO50960412/JCSC6670)	CC22	POS	POS	POS	NEG	POS	POS	POS	NEG	NEG	NEG	NEG	NEG	NEG	POS	NEG	POS	NEG	NEG	NEG	POS	POS	POS	POS
Prediction for M08-0126, GenBank FR753166.1	***SCCmec IVh/j+ACME2 (M08-0126)***	***CC22***																							
**UKD-1379483I**	SCCmec IVh/j+ACME2 (M08-0126)	CC22	POS	POS	POS	POS	NEG	AMB	NEG	NEG	NEG	NEG	NEG	NEG	NEG	POS	NEG	POS	NEG	NEG	NEG	POS	POS	POS	POS
**UKD-5150204**	SCCmec IV+speG+Cu/czrC+ccrAB4	CC22	POS	POS	POS	POS	NEG	NEG	NEG	NEG	NEG	NEG	NEG	NEG	NEG	POS	NEG	POS	NEG	NEG	NEG	POS	POS	POS	POS
**UKD-15578423**	SCCmec IV+speG+czrC+ccrAB4 var. 1	CC22	POS	POS	POS	POS	POS	POS	POS	NEG	NEG	NEG	NEG	NEG	NEG	POS	NEG	POS	NEG	NEG	NEG	POS	POS	POS	POS
**UKD-16582870**	SCCmec IV+speG+czrC+ccrAB4 var. 1	CC22	POS	POS	POS	NEG	POS	POS	POS	NEG	NEG	NEG	NEG	NEG	NEG	POS	NEG	POS	NEG	NEG	NEG	POS	POS	POS	POS
**UKD-15571940**	SCCmec IV+speG+czrC+ccrAB4 var. 2	CC22	POS	POS	POS	POS	POS	POS	POS	NEG	NEG	NEG	NEG	NEG	NEG	POS	NEG	POS	NEG	NEG	NEG	POS	POS	POS	POS

Isolate ID	SCCmec type	Clonal complex	VIRULENCE : HAEMOLYSIN BETA AND HLB-CONV PHAGES							VIRULENCE : OTHER FACTORS						VIRULENCE : PROTEASES									
			haemolysin beta				staphylo-kinase	chemotaxis-inhibiting protein (CHIPS)	Staphyl. Comple-ment inhibitor	exfoliative toxins			epidermal cell differentiation inhibitors			aureolysin			serin-protease A	serin-protease B	serin-protease E	glutamylendopeptidase	staphopain B, protease	staphopain A (staphylopain A), protease	
			hlb-probe 1	hlb-probe 2	hlb-probe 3	un-truncated hlb	sak	chp	scn	etA	etB	etD	edinA	edinB	edinC	aur (cons)	aur (Other than MRSA252)	aur (MRSA252)	splA	splB	splE	sspA	sspB	sspP (cons)	sspP (other than ST93)
Prediction for CN1, GenBank CP003979.1	*SCCmec IVA (CN1)*	*CC8 (ST72)*	POS	POS	POS	POS	POS	POS	POS	NEG	NEG	NEG	NEG	NEG	NEG	POS	POS	NEG	POS	POS	NEG	POS	POS	POS	POS
UKD-0900871	SCCmec IVA (CN1)	CC8 (ST72)	POS	POS	POS	NEG	POS	POS	POS	NEG	NEG	NEG	NEG	NEG	NEG	POS	POS	NEG	POS	POS	NEG	POS	POS	POS	POS
Prediction for CMFT1723, GenBank HF569096.1	*SCCmec V (Bengal Bay)*																								
KHDN-10405134	SCCmec V (Bengal Bay)	CC1	POS	POS	POS	POS	POS	NEG	POS	NEG	NEG	NEG	NEG	NEG	NEG	POS	POS	NEG	POS	POS	POS	POS	POS	POS	POS
UKD-0932405	SCCmec V (Bengal Bay)	CC1 (ST573/772)	NEG	NEG	AMB	NEG	NEG	NEG	POS	NEG	NEG	NEG	NEG	NEG	NEG	POS	POS	NEG	NEG	NEG	NEG	POS	POS	POS	POS
UKD-1003510	SCCmec V (Bengal Bay)	CC1 (ST573/772)	NEG	NEG	POS	NEG	NEG	NEG	POS	NEG	NEG	NEG	NEG	NEG	NEG	POS	POS	NEG	NEG	NEG	NEG	POS	POS	POS	POS
UKD-1112420	SCCmec V (Bengal Bay)	CC1 (ST573/772)	NEG	NEG	AMB	AMB	NEG	NEG	POS	NEG	NEG	NEG	NEG	NEG	NEG	POS	POS	NEG	NEG	NEG	NEG	POS	POS	POS	POS
UKD-1275832	SCCmec V (Bengal Bay)	CC1 (ST573/772)	AMB	NEG	POS	POS	NEG	NEG	POS	NEG	NEG	NEG	NEG	NEG	NEG	POS	POS	NEG	NEG	NEG	NEG	POS	POS	POS	POS
UKD-13023811	SCCmec V (Bengal Bay)	CC1 (ST573/772)	NEG	NEG	POS	NEG	NEG	NEG	POS	NEG	NEG	NEG	NEG	NEG	NEG	POS	POS	NEG	NEG	NEG	NEG	POS	POS	POS	POS
UKD-14572163	SCCmec V	CC5	POS	POS	POS	NEG	POS	NEG	POS	NEG	NEG	NEG	NEG	NEG	NEG	POS	POS	NEG	POS	POS	NEG	POS	POS	POS	POS
KHDN-10421133	SCCmec V+fus	CC97	POS	POS	POS	POS	POS	NEG	POS	NEG	NEG	NEG	NEG	NEG	NEG	POS	POS	NEG	POS	POS	POS	POS	POS	POS	POS
KHDN-21343541	SCCmec V+fus+tir+ccrAB1	CC1	POS	POS	POS	AMB	POS	NEG	POS	NEG	NEG	NEG	NEG	NEG	NEG	POS	POS	NEG	POS	POS	POS	POS	POS	POS	POS
KHDN-10416893	SCCmec V+czrC+speG+ccrAB4	CC22	POS	POS	POS	NEG	POS	POS	POS	NEG	NEG	NEG	NEG	NEG	NEG	POS	NEG	POS	NEG	NEG	NEG	POS	POS	POS	POS
Prediction for GR1, GenBank AJLX	*SCCmec VT (GR1)*	*CC361*	POS	POS	AMB	POS	POS	NEG	POS	NEG	NEG	NEG	NEG	NEG	NEG	POS	POS	NEG	NEG	NEG	NEG	POS	POS	POS	POS
UKD-0422872	SCCmec VT (GR1)	CC152	AMB	AMB	POS	POS	POS	NEG	POS	NEG	NEG	NEG	NEG	POS	NEG	POS	POS	NEG	NEG	NEG	NEG	POS	POS	POS	POS
Prediction for PM1, GenBank BAFA	*SCCmec VT (PM1)*	*CC59*	AMB	NEG	AMB	AMB	NEG	POS	POS	NEG	NEG	NEG	NEG	NEG	NEG	POS	POS	NEG	NEG	NEG	NEG	AMB	POS	POS	POS
KHDN-000871	SCCmec VT (PM1)	CC45 (agr I)	NEG	NEG	NEG	POS	NEG	NEG	NEG	NEG	NEG	NEG	NEG	NEG	NEG	POS	NEG	POS	NEG	NEG	NEG	POS	POS	POS	POS
UKD-0725052	SCCmec VT (PM1)	CC59	NEG	NEG	NEG	POS	NEG	POS	POS	NEG	NEG	NEG	NEG	NEG	NEG	POS	AMB	NEG	NEG	NEG	NEG	POS	POS	POS	POS
UKD-1312138	SCCmec VT (PM1)	CC59	POS	NEG	POS	POS	NEG	POS	POS	NEG	NEG	NEG	NEG	NEG	NEG	POS	POS	NEG	NEG	NEG	NEG	POS	POS	POS	POS
UKD-Z4728	SCCmec VT (PM1)	CC59	POS	NEG	POS	POS	NEG	POS	POS	NEG	NEG	NEG	NEG	NEG	NEG	POS	POS	NEG	NEG	NEG	NEG	POS	POS	POS	POS
UKD-K0199	SCCmec VT unknown var.1	CC7	POS	POS	POS	NEG	POS	NEG	POS	NEG	NEG	NEG	NEG	NEG	NEG	POS	POS	NEG	POS	POS	POS	POS	POS	POS	POS
UKD-15584619	SCCmec VT unknown var.2	CC30	POS	POS	POS	POS	POS	POS	POS	NEG	NEG	NEG	NEG	NEG	NEG	POS	NEG	POS	NEG	NEG	POS	POS	POS	POS	POS
UKD-0712568	SCCmec VT unknown var.3	CC45 (agr I)	AMB	AMB	NEG	NEG	POS	POS	POS	NEG	NEG	NEG	NEG	NEG	NEG	NEG	NEG	POS	NEG	NEG	NEG	POS	POS	POS	POS
KHDN-10420296	SCCmec VT+ACME2	CC45 (agr I)	NEG	NEG	NEG	NEG	POS	POS	POS	NEG	NEG	NEG	NEG	NEG	NEG	POS	NEG	POS	NEG	NEG	NEG	POS	POS	POS	POS
UKD-15577956	SCCmec VT+Cu/czrC+ccrA1	CC398	NEG	NEG	NEG	POS	NEG	NEG	NEG	NEG	NEG	NEG	NEG	NEG	NEG	POS	NEG	POS	NEG	NEG	NEG	POS	POS	POS	POS
Prediction for S0385, GenBank AM990992	*SCCmec VT+czrC (SO385)*	*CC398*	NEG	NEG	NEG	NEG	NEG	NEG	NEG	NEG	NEG	NEG	NEG	NEG	NEG	POS	NEG	POS	NEG	NEG	NEG	POS	POS	POS	POS
UKD-0628560	SCCmec VT+czrC (SO385)	CC8	POS	POS	POS	NEG	POS	NEG	POS	NEG	NEG	NEG	NEG	NEG	NEG	POS	POS	NEG	POS	POS	POS	POS	POS	POS	POS
UKD-0510018	SCCmec VT+czrC (SO385)	CC398	NEG	NEG	NEG	NEG	NEG	NEG	NEG	NEG	NEG	NEG	NEG	NEG	NEG	NEG	NEG	POS	NEG	NEG	NEG	POS	POS	POS	POS
UKD-0903558	SCCmec VT+czrC (SO385)	CC398	NEG	NEG	NEG	POS	NEG	NEG	NEG	NEG	NEG	NEG	NEG	NEG	NEG	AMB	NEG	POS	NEG	NEG	NEG	POS	POS	POS	POS
UKD-0929815	SCCmec VT+czrC (SO385)	CC398	NEG	NEG	NEG	POS	NEG	NEG	NEG	NEG	NEG	NEG	NEG	NEG	NEG	POS	NEG	POS	NEG	NEG	NEG	POS	POS	POS	POS
UKD-0907762	SCCmec VT+czrC (SO385)	CC398	NEG	NEG	NEG	POS	NEG	NEG	NEG	NEG	NEG	NEG	NEG	NEG	NEG	POS	NEG	POS	NEG	NEG	NEG	POS	POS	POS	POS
UKD-1025105	SCCmec VT+czrC (SO385)	CC398	NEG	NEG	NEG	AMB	NEG	NEG	NEG	NEG	NEG	NEG	NEG	NEG	NEG	POS	NEG	POS	NEG	NEG	NEG	POS	POS	POS	POS
UKD-11780898	SCCmec VT+czrC (SO385)	CC398	NEG	NEG	NEG	POS	NEG	NEG	NEG	NEG	NEG	NEG	NEG	NEG	NEG	POS	NEG	POS	NEG	NEG	NEG	POS	POS	POS	POS
UKD-1377009	SCCmec VT+czrC (SO385)	CC398	NEG	NEG	NEG	POS	NEG	NEG	NEG	NEG	NEG	NEG	NEG	NEG	NEG	POS	NEG	POS	NEG	NEG	NEG	POS	POS	POS	POS
UKD-143406	SCCmec VT+czrC (SO385)	CC398	NEG	NEG	NEG	POS	POS	POS	POS	NEG	NEG	NEG	NEG	NEG	NEG	POS	NEG	POS	NEG	NEG	NEG	POS	POS	POS	POS
UKD-1400363	SCCmec VT+czrC (SO385)	CC398	NEG	NEG	NEG	POS	NEG	NEG	NEG	NEG	NEG	NEG	NEG	NEG	NEG	POS	NEG	POS	NEG	NEG	NEG	POS	POS	POS	POS
UKD-14571852	SCCmec VT+czrC (SO385)	CC398	NEG	NEG	NEG	POS	POS	POS	POS	NEG	NEG	NEG	NEG	NEG	NEG	POS	NEG	POS	NEG	NEG	NEG	POS	POS	POS	POS
UKD-14582574	SCCmec VT+czrC (SO385)	CC398	NEG	NEG	NEG	POS	NEG	NEG	NEG	NEG	NEG	NEG	NEG	NEG	NEG	POS	NEG	POS	NEG	NEG	NEG	POS	POS	POS	POS
UKD-152108	SCCmec VT+czrC (SO385)	CC398	NEG	NEG	NEG	POS	NEG	NEG	NEG	NEG	NEG	NEG	NEG	NEG	NEG	POS	NEG	POS	NEG	NEG	NEG	POS	POS	POS	POS
UKD-15573321	SCCmec VT+czrC (SO385)	CC398	NEG	NEG	NEG	POS	NEG	NEG	NEG	NEG	NEG	NEG	NEG	NEG	NEG	POS	NEG	POS	NEG	NEG	NEG	POS	POS	POS	POS
UKD-15584034	SCCmec VT+czrC , ydhK-neg. variant	CC398	NEG	NEG	NEG	POS	NEG	NEG	NEG	NEG	NEG	NEG	NEG	NEG	NEG	POS	NEG	POS	NEG	NEG	NEG	POS	POS	POS	POS
UKD-0932581	SCCmecVT+fus+czrC	CC22	POS	POS	POS	NEG	POS	POS	POS	NEG	NEG	NEG	NEG	NEG	NEG	POS	NEG	POS	NEG	NEG	NEG	POS	POS	POS	POS
UKD-1121994	SCCmecVT+fus+czrC	CC22	POS	POS	POS	POS	NEG	NEG	NEG	NEG	NEG	NEG	NEG	NEG	NEG	POS	NEG	POS	NEG	NEG	NEG	POS	POS	POS	POS
UKD-1272252	SCCmecVT+fus+czrC	CC22	POS	POS	POS	POS	POS	POS	POS	NEG	NEG	NEG	NEG	NEG	NEG	POS	NEG	POS	NEG	NEG	NEG	POS	POS	POS	POS
Prediction for MRSA18	*SCCmec VI+fus (MRSA18)*																								
UKD-1103380	SCCmec VI+fus (MRSA18)	CC7	AMB	POS	NEG	NEG	POS	NEG	POS	NEG	NEG	NEG	NEG	NEG	NEG	POS	POS	NEG	POS	POS	POS	POS	POS	POS	POS
Prediction for M10/0061, GenBank FR823292.1	*SCCmec XI (LGA251/M10-61)*	*CC130*																							
**Reference strain Dublin-DSH_M10 0061**	**SCCmec XI (LGA251/M10-61)**	**CC130**	**POS**	**POS**	**POS**	**POS**	**NEG**	**NEG**	**NEG**	**NEG**	**NEG**	**NEG**	**NEG**	**POS**	**NEG**	**POS**	**POS**	**NEG**	**POS**	**POS**	**POS**	**POS**	**POS**	**POS**	**POS**
Prediction for LGA251, GenBank FR821779.1	*SCCmec XI (LGA251/M10-61)*	*ST425*																							
UKD-14015835	SCCmec XI (LGA251/M10-61)	CC130	POS	POS	POS	POS	NEG	NEG	NEG	NEG	NEG	NEG	NEG	POS	NEG	POS	POS	NEG	POS	POS	POS	POS	POS	POS	POS
UKD-16573252	SCCmec XI (LGA251/M10-61)	CC130	POS	POS	POS	POS	NEG	NEG	NEG	NEG	NEG	NEG	NEG	POS	NEG	POS	POS	NEG	POS	POS	NEG	POS	POS	POS	POS
UKD-0217002	PseudoSCCmec, class B mec complex+Hg	CC8	POS	POS	AMB	POS	NEG	NEG	NEG	NEG	NEG	NEG	NEG	NEG	NEG	POS	POS	NEG	POS	POS	POS	POS	POS	POS	POS
UKD-0008510	PseudoSCCmec, class B mec complex+Hg	CC8	POS	POS	POS	NEG	POS	NEG	POS	NEG	NEG	NEG	NEG	NEG	NEG	POS	POS	NEG	POS	POS	POS	POS	POS	POS	POS
UKD-0110962	PseudoSCCmec, class B mec complex+Hg	CC8	POS	POS	POS	NEG	POS	NEG	POS	NEG	NEG	NEG	NEG	NEG	NEG	POS	POS	NEG	POS	POS	POS	POS	POS	POS	POS
KHDN-10408593	PseudoSCCmec, class B mec complex+Cu/Hg	CC8	POS	POS	POS	NEG	POS	POS	POS	NEG	NEG	NEG	NEG	NEG	NEG	POS	POS	NEG	POS	POS	POS	POS	POS	POS	POS
UKD-0007201	PseudoSCCmec, class B mec complex	CC5	POS	POS	POS	NEG	POS	POS	POS	NEG	NEG	NEG	NEG	NEG	NEG	POS	POS	NEG	POS	POS	NEG	POS	POS	POS	POS
UKD-0033204	PseudoSCCmec, class B mec complex	CC5	POS	POS	AMB	NEG	POS	POS	POS	NEG	NEG	NEG	NEG	NEG	NEG	POS	POS	NEG	POS	POS	NEG	POS	POS	POS	POS
UKD-0134329	PseudoSCCmec, class B mec complex	CC5	NEG	POS	NEG	NEG	POS	POS	POS	NEG	NEG	NEG	NEG	NEG	NEG	POS	AMB	NEG	POS	AMB	NEG	POS	POS	AMB	AMB
UKD-0806946	PseudoSCCmec class C+As/Cu	CC398	NEG	NEG	NEG	NEG	NEG	NEG	NEG	NEG	NEG	NEG	NEG	NEG	NEG	POS	NEG	POS	NEG	NEG	NEG	POS	POS	POS	POS
UKD-0500610	ACME2	CC8	POS	POS	POS	NEG	POS	NEG	POS	NEG	NEG	NEG	NEG	NEG	NEG	POS	POS	NEG	POS	POS	AMB	POS	POS	POS	POS
UKD-0610438	ACME2	CC8	POS	POS	POS	POS	POS	NEG	POS	NEG	NEG	NEG	NEG	NEG	NEG	POS	POS	NEG	POS	POS	POS	POS	POS	POS	POS
KHDN-10409344	ACME2	CC8	POS	POS	POS	AMB	POS	POS	POS	NEG	NEG	NEG	NEG	NEG	NEG	POS	POS	NEG	POS	POS	POS	POS	POS	POS	POS
UKD-0322559	ACME2+speG+czrC+ccrAB4	CC8	POS	POS	AMB	POS	NEG	NEG	NEG	NEG	NEG	NEG	NEG	NEG	NEG	POS	POS	NEG	POS	POS	AMB	POS	POS	POS	POS
UKD-0501998	ACME2+speG+czrC+ccrAB4	CC8	POS	POS	POS	NEG	POS	NEG	POS	NEG	NEG	NEG	NEG	NEG	NEG	POS	POS	NEG	POS	POS	NEG	POS	POS	POS	POS
UKD-0505213	ACME2+speG+czrC+ccrAB4	CC8	POS	POS	POS	NEG	POS	NEG	POS	NEG	NEG	NEG	NEG	NEG	NEG	POS	POS	NEG	POS	POS	POS	POS	POS	POS	POS
Prediction for MSSA476, GenBank BX571857.1	*fus+tir+ccrAB1 (MSSA476)*	*CC1*	POS	POS	POS	POS	POS	NEG	POS	NEG	NEG	NEG	NEG	NEG	NEG	POS	POS	NEG	POS	POS	NEG	POS	POS	POS	POS
**Reference strain Sanger 476**	**fus+tir+ccrAB1 (MSSA476)**	**CC1**	**AMB**	**POS**	**AMB**	**NEG**	**POS**	**NEG**	**POS**	**NEG**	**NEG**	**NEG**	**NEG**	**NEG**	**NEG**	**POS**	**POS**	**NEG**	**POS**	**POS**	**NEG**	**POS**	**POS**	**POS**	**POS**
UKD-14023914	fus+tir+ccrAB1 (MSSA476)	CC1	POS	POS	POS	NEG	POS	NEG	POS	NEG	NEG	NEG	NEG	NEG	NEG	POS	POS	NEG	POS	POS	POS	POS	POS	POS	POS
UKD-K00034	kdp+ccrAB2	CC182	POS	POS	POS	NEG	POS	POS	POS	NEG	NEG	NEG	NEG	NEG	NEG	POS	POS	NEG	NEG	NEG	NEG	POS	POS	POS	POS
UKD-15580369	As+ccrAB4	CC22	POS	POS	POS	NEG	POS	POS	POS	NEG	NEG	NEG	NEG	NEG	NEG	POS	NEG	POS	NEG	NEG	NEG	POS	POS	POS	POS
UKD-15570100	ACME3+ccrAB1 (Strain 21342)	CC80	POS	POS	POS	POS	POS	NEG	POS	NEG	NEG	NEG	NEG	NEG	NEG	POS	POS	NEG	POS	POS	NEG	POS	POS	POS	POS
UKD-1010678	speG+czrC+ccrAB4	CC8	POS	POS	POS	NEG	POS	NEG	POS	NEG	NEG	NEG	NEG	NEG	NEG	POS	POS	NEG	POS	POS	POS	POS	POS	POS	POS
UKD-1008243	speG+czrC+ccrAB4	CC8	POS	POS	POS	NEG	POS	NEG	POS	NEG	NEG	NEG	NEG	NEG	NEG	POS	POS	NEG	POS	POS	POS	POS	POS	POS	POS
UKD-1009430	speG+czrC+ccrAB4	CC8	POS	POS	POS	NEG	POS	NEG	POS	NEG	NEG	NEG	NEG	NEG	NEG	POS	POS	NEG	POS	POS	POS	POS	POS	POS	POS
UKD-12071828	speG+czrC+ccrAB4	CC22	POS	POS	POS	NEG	POS	POS	POS	NEG	NEG	NEG	NEG	NEG	NEG	POS	NEG	POS	NEG	NEG	NEG	POS	POS	POS	POS
UKD-14011883	speG+czrC+ccrAB4	CC22	POS	POS	POS	NEG	POS	POS	POS	NEG	NEG	NEG	NEG	NEG	NEG	POS	NEG	POS	NEG	NEG	NEG	POS	POS	POS	POS
UKD-15008293	speG+czrC+ccrAB4	CC22	POS	POS	POS	NEG	POS	POS	POS	NEG	NEG	NEG	NEG	NEG	NEG	POS	NEG	POS	NEG	NEG	NEG	POS	POS	POS	POS
UKD-1514877	speG+czrC+ccrAB4	CC22	POS	POS	POS	POS	POS	POS	POS	NEG	NEG	NEG	NEG	NEG	NEG	POS	NEG	POS	NEG	NEG	NEG	POS	POS	POS	POS
UKD-15580971	speG+czrC+ccrAB4	CC22	POS	POS	POS	POS	POS	POS	POS	NEG	NEG	NEG	NEG	NEG	NEG	POS	NEG	POS	NEG	NEG	NEG	POS	POS	POS	POS
UKD-15582646	speG+czrC+ccrAB4	CC22	POS	POS	POS	POS	POS	POS	POS	NEG	NEG	NEG	NEG	NEG	NEG	POS	NEG	POS	NEG	NEG	NEG	POS	POS	POS	POS

Isolate ID	SCCmec type	Clonal complex	VIRULENCE : STAPHYLOCOCCAL SUPERANTIGEN/ENTEROTOXIN-LIKE GENES (SET/SSL)																			
			staphyl. exotoxin-like protein	staphylococcal superantigen-like protein 1						staphylococcal superantigen-like protein 2		staphylococcal superantigen-like protein 3			staphylococcal superantigen-like protein 4		staphylococcal superantigen-like protein 5				staphylococcal superantigen-like protein 6	
			setC / selX	set6-var1_11	set6-var2_11	set6-var1_12	set6-var2_12	set6-var4_11	ssl01-RF122	ssl02/set7	ssl02/set7 (MRSA252)	ssl03/set8_probe1	ssl03/set8_probe2	ssl03/set8 (MRSA252, SAR0424)	ssl04/set9	ssl04/set9 (MRSA252, SAR0425)	ssl05/set3_probe1	ssl05/set3 (RF122, probe-611)	ssl05/set3_probe2 (612)	ssl05/set3 (MRSA252)	ssl06/set21	ssl06 (NCTC8325+MW2)
VIRTUAL HYBRIDISATION for COL, GenBank CP000046	*SCCmec I (COL)*	*CC8*	POS	POS	NEG	AMB	NEG	POS	NEG	POS	AMB	POS	POS	NEG	POS	NEG	NEG	NEG	NEG	NEG	NEG	NEG
Reference strain COL	SCCmec I (COL)	CC8	POS	POS	NEG	POS	NEG	POS	NEG	POS	NEG	POS	POS	NEG	POS	NEG	NEG	NEG	NEG	NEG	NEG	AMB
UKD-0007063	SCCmec I (COL)	CC5	POS	POS	POS	POS	AMB	POS	NEG	POS	AMB	POS	POS	NEG	POS	AMB	POS	AMB	POS	NEG	NEG	AMB
UKD-0220759	SCCmec I (COL)	CC5	POS	POS	NEG	POS	NEG	POS	NEG	POS	NEG	POS	POS	NEG	POS	NEG	POS	POS	NEG	NEG	NEG	NEG
UKD-0308986	SCCmec I (COL)	CC5	POS	POS	NEG	POS	NEG	POS	NEG	POS	AMB	POS	POS	NEG	POS	AMB	POS	AMB	POS	NEG	NEG	NEG
UKD-0500009	SCCmec I (COL)	CC5	POS	POS	NEG	POS	NEG	POS	NEG	POS	NEG	POS	POS	NEG	POS	NEG	POS	AMB	POS	NEG	NEG	NEG
UKD-0508483	SCCmec I (COL)	CC5	POS	POS	NEG	POS	NEG	POS	NEG	POS	NEG	POS	POS	NEG	POS	NEG	POS	NEG	POS	NEG	NEG	NEG
UKD-0519001	SCCmec I (COL)	CC5	POS	POS	AMB	POS	NEG	POS	NEG	POS	AMB	POS	POS	NEG	POS	NEG	POS	AMB	POS	NEG	NEG	NEG
UKD-0701876	SCCmec I (COL)	CC5	POS	POS	NEG	POS	NEG	POS	NEG	POS	AMB	POS	POS	NEG	POS	NEG	POS	AMB	POS	NEG	NEG	POS
UKD-0708865	SCCmec I (COL)	CC5	POS	POS	NEG	POS	NEG	POS	NEG	POS	NEG	POS	POS	NEG	POS	NEG	POS	NEG	POS	NEG	NEG	NEG
UKD-1001782	SCCmec I (COL)	CC5	POS	POS	NEG	POS	NEG	POS	NEG	POS	AMB	POS	POS	NEG	POS	AMB	POS	AMB	POS	NEG	NEG	NEG
Prediction for PSP1996, GenBank ANHU	*SCCmec I (PSP1996)*	*CC8*	POS	POS	NEG	AMB	NEG	POS	NEG	POS	AMB	POS	POS	NEG	POS	NEG	POS	NEG	POS	NEG	POS	POS
UKD-0261727	SCCmec I (PSP1996)	CC8	POS	POS	NEG	NEG	NEG	POS	NEG	POS	NEG	POS	POS	NEG	POS	NEG	POS	AMB	POS	NEG	POS	POS
VIRTUAL HYBRIDISATION for MRSA7 : VIRTUAL HYBRII	*SCCmec I+fus+tirS (Geraldine Clone)*	*CC5*	POS	POS	NEG	POS	NEG	AMB	NEG	POS	AMB	POS	POS	NEG	POS	NEG	POS	NEG	POS	NEG	NEG	NEG
UKD-1221930	SCCmec I+fus+tirS (Geraldine Clone)	CC5	POS	POS	NEG	POS	NEG	POS	NEG	POS	AMB	POS	POS	NEG	POS	AMB	POS	AMB	POS	NEG	NEG	NEG
Prediction for N315, GenBank BA000018.3	*SCCmec II (N315)*	*CC5*	POS	POS	NEG	POS	NEG	AMB	NEG	POS	AMB	POS	POS	NEG	POS	NEG	POS	NEG	POS	NEG	NEG	NEG
Reference strain N315	SCCmec II (N315)	CC5	POS	POS	NEG	POS	NEG	POS	NEG	POS	NEG	POS	POS	NEG	POS	NEG	POS	NEG	POS	NEG	NEG	NEG
Prediction for MRSA252, GenBank BX571856.1	*SCCmec II (N315)*	*CC30*	NEG	NEG	POS	POS	NEG	AMB	NEG	AMB	POS	NEG	NEG	POS	NEG	POS	NEG	NEG	NEG	POS	NEG	NEG
Reference strain Sanger 252	SCCmec II (N315)	CC30	NEG						NEG	NEG	POS	NEG	NEG	POS	NEG	POS	NEG	NEG	NEG	POS	NEG	NEG
UKD-0230792	SCCmec II (N315)	CC30	NEG	NEG	POS	POS	NEG	POS	NEG	AMB	POS	NEG	NEG	POS	NEG	POS	NEG	NEG	NEG	POS	NEG	AMB
Prediction for Mu50, GenBank BA000017.4	*SCCmec II (N315)*	*CC5*	POS	POS	NEG	POS	NEG	AMB	NEG	POS	AMB	POS	POS	NEG	NEG	NEG	POS	NEG	POS	NEG	NEG	NEG
Reference strain MU50	SCCmec II (N315)	CC5	POS	POS	NEG	POS	NEG	POS	NEG	POS	NEG	POS	POS	NEG	NEG	NEG	POS	NEG	POS	NEG	NEG	NEG
Prediction for JH1, GenBank CP000736.1	*SCCmec II (JH1/JH9)*	*CC5*	POS	POS	NEG	POS	NEG	AMB	NEG	POS	AMB	POS	POS	NEG	POS	NEG	POS	NEG	POS	NEG	NEG	NEG
Prediction for JH9, GenBank CP000703.1	*SCCmec II (JH1/JH9)*	*CC5*	POS	POS	NEG	POS	NEG	AMB	NEG	POS	AMB	POS	POS	NEG	POS	NEG	POS	NEG	POS	NEG	NEG	NEG
Reference strain Dublin-DSH_AR07.4_0237	SCCmec II (JH1/JH9)		POS	POS	NEG	POS	NEG	POS	NEG	POS	AMB	POS	POS	NEG	POS	AMB	POS	AMB	POS	NEG	NEG	AMB
Reference strain NARSA_382 / USA100	SCCmec II (JH1/JH9)	CC5	POS	POS	NEG	POS	NEG	POS	NEG	POS	AMB	POS	POS	NEG	POS	NEG	POS	AMB	POS	NEG	NEG	NEG
UKD-0046921	SCCmec II (JH1/JH9)	CC5	POS	POS	NEG	POS	NEG	POS	NEG	POS	AMB	POS	POS	NEG	POS	NEG	POS	AMB	POS	NEG	NEG	NEG
UKD-0333415	SCCmec II (JH1/JH9)	CC5	POS	POS	NEG	POS	NEG	POS	NEG	POS	AMB	POS	POS	NEG	POS	NEG	POS	AMB	POS	NEG	NEG	NEG
UKD-0341645	SCCmec II (JH1/JH9)	CC5	POS	POS	NEG	POS	NEG	POS	NEG	POS	AMB	POS	POS	NEG	POS	NEG	POS	AMB	POS	NEG	NEG	NEG
UKD-0504626	SCCmec II (JH1/JH9)	CC5	POS	POS	NEG	POS	NEG	POS	NEG	POS	NEG	AMB	POS	NEG	POS	NEG	POS	NEG	POS	NEG	NEG	NEG
UKD-0507581	SCCmec II (JH1/JH9)	CC5	POS	POS	NEG	POS	NEG	POS	NEG	POS	NEG	POS	POS	NEG	POS	NEG	POS	NEG	POS	NEG	NEG	NEG
UKD-0507903	SCCmec II (JH1/JH9)	CC5	POS	POS	NEG	POS	NEG	POS	NEG	POS	NEG	POS	POS	NEG	POS	NEG	POS	NEG	POS	NEG	NEG	NEG
UKD-0700817	SCCmec II (JH1/JH9)	CC5	POS	POS	NEG	POS	NEG	POS	NEG	POS	AMB	POS	POS	NEG	POS	NEG	POS	NEG	POS	NEG	NEG	NEG
UKD-1014561	SCCmec II (JH1/JH9)	CC5	POS	POS	NEG	POS	NEG	POS	NEG	POS	AMB	POS	POS	NEG	POS	NEG	POS	AMB	POS	NEG	NEG	NEG
UKD-1037507	SCCmec II (JH1/JH9)	CC5	POS	POS	AMB	POS	AMB	POS	NEG	AMB	POS	POS	POS	NEG	POS	NEG	POS	AMB	POS	NEG	NEG	NEG
UKD-1038321	SCCmec II (JH1/JH9)	CC5	POS	POS	NEG	POS	NEG	AMB	NEG	POS	NEG	POS	POS	NEG	POS	NEG	POS	NEG	POS	NEG	NEG	NEG
UKD-1111608	SCCmec II (JH1/JH9)	CC5	POS	POS	NEG	POS	NEG	POS	NEG	POS	AMB	POS	POS	NEG	POS	NEG	POS	AMB	POS	NEG	NEG	NEG
UKD-12753135	SCCmec II (JH1/JH9)	CC5	POS	POS	NEG	POS	NEG	POS	NEG	POS	AMB	POS	POS	NEG	POS	AMB	POS	AMB	POS	NEG	NEG	NEG
UKD-1312816	SCCmec II (JH1/JH9)	CC5	POS	POS	NEG	POS	NEG	POS	NEG	POS	AMB	POS	POS	NEG	POS	NEG	POS	AMB	POS	NEG	NEG	NEG
UKD-1401623	SCCmec II (JH1/JH9)	CC5	POS	POS	NEG	POS	NEG	POS	NEG	POS	AMB	POS	POS	NEG	POS	AMB	POS	POS	AMB	NEG	NEG	POS
UKD-15571943	SCCmec II+czrC+ccrA/B-4	CC5	POS	POS	POS	POS	POS	POS	NEG	POS	AMB	POS	POS	NEG	POS	POS	POS	POS	AMB	NEG	NEG	POS
UKD-15576888	SCCmec II+czrC+ccrA/B-4	CC5	POS	POS	POS	POS	AMB	POS	NEG	POS	AMB	POS	POS	NEG	POS	AMB	POS	AMB	POS	NEG	NEG	POS
UKD-14576461	SCCmec II+speG+czrC+ccrA/B-4	CC5	POS	POS	NEG	POS	NEG	POS	NEG	POS	AMB	POS	POS	NEG	POS	AMB	POS	AMB	POS	NEG	NEG	POS
UKD-15581649	SCCmec II+speG+czrC+ccrA/B-4	CC5	POS	POS	NEG	POS	NEG	POS	NEG	POS	AMB	POS	POS	NEG	POS	NEG	POS	AMB	POS	NEG	NEG	AMB
Prediction for CIGC345D, GenBank AHVO	*SCCmec II A/B/D*	*CC8*	POS	POS	NEG	AMB	NEG	POS	NEG	POS	AMB	POS	POS	NEG	POS	NEG	POS	NEG	POS	NEG	POS	POS
UKD-12K00171	SCCmec II A/B/D	CC8	POS	POS	NEG	POS	NEG	POS	NEG	POS	AMB	POS	POS	NEG	POS	NEG	POS	POS	AMB	NEG	POS	POS
Prediction for JKD6008, GenBank CP002120.1	*SCC [mec III+Cd] (JKD6008)*	*CC8 (ST239)*	POS	POS	NEG	AMB	NEG	POS	NEG	POS	AMB	POS	POS	NEG	POS	NEG	POS	NEG	POS	NEG	POS	POS
Prediction for JKD6009, GenBank ABSA	*SCC [mec III+Cd] (JKD6008)*	*CC8 (ST239)*	POS	POS	NEG	AMB	NEG	POS	NEG	POS	AMB	POS	POS	NEG	POS	NEG	POS	NEG	POS	NEG	POS	NEG
Reference strain ATCC_33592	SCC [mec III+Cd] (ATCC33592)	CC8 (ST239)	POS	POS	NEG	POS	NEG	POS	NEG	POS	AMB	POS	POS	NEG	POS	NEG	POS	POS	POS	NEG	POS	POS
Prediction for PPUKM-775-2009, GenBank AMRE	*SCC [mec III+Cd+ccrC] (PPUKM-775-2009)*	*CC8 (ST239)*	POS	POS	NEG	AMB	NEG	POS	NEG	POS	AMB	POS	POS	NEG	POS	NEG	POS	NEG	POS	NEG	POS	POS
Prediction for T0131, GenBank CP002643.1	*SCC [mec III+Cd+ccrC] (T0131)*	*CC8 (ST239)*	POS	POS	NEG	AMB	NEG	POS	NEG	POS	AMB	POS	POS	NEG	POS	NEG	POS	NEG	POS	NEG	POS	POS
Prediction for XN108, GenBank CP007447.1	*SCC [mec III+Cd+ccrC] (XN108)*	*CC8 (ST239)*	POS	POS	NEG	AMB	NEG	POS	NEG	POS	AMB	POS	POS	NEG	POS	NEG	POS	NEG	POS	NEG	POS	POS
Prediction for CN79, GenBank ANCJ	*SCC [mec III+Cd+ccrC] (CN79)*	*CC8 (ST239)*	POS	POS	NEG	AMB	NEG	POS	NEG	POS	AMB	POS	POS	NEG	POS	NEG	POS	NEG	POS	NEG	POS	POS
Prediction for 16K, GenBank BABZ	*SCC [mec III+Cd+ccrC] (CN79)*	*CC8 (ST239)*	POS	POS	NEG	AMB	NEG	POS	NEG	POS	AMB	POS	POS	NEG	POS	NEG	POS	NEG	POS	NEG	POS	POS
UKD-0835987	SCC [mec III+Cd+ccrC] (CN79)	CC8 (ST239)	POS	POS	NEG	AMB	NEG	POS	NEG	POS	NEG	POS	POS	NEG	POS	NEG	POS	AMB	POS	NEG	AMB	POS
UKD-16581769	SCCmec III+Cd+ccrC	CC8 (ST239)	POS	POS	NEG	NEG	NEG	POS	NEG	POS	NEG	POS	POS	NEG	POS	NEG	POS	NEG	POS	NEG	POS	POS
Prediction for Bmb9393, GenBank CP005288.1	*SCCmec III+Cd/Hg+ccrC (Bmb9393)*	*CC8 (ST239)*	POS	POS	NEG	AMB	NEG	POS	NEG	POS	AMB	POS	POS	NEG	POS	NEG	POS	NEG	POS	NEG	POS	POS
UKD-15574263	SCCmec III+Cd/Hg+ccrC (Bmb9393)	CC8 (ST239)	POS	POS	NEG	POS	NEG	POS	NEG	POS	AMB	POS	POS	NEG	POS	NEG	POS	AMB	POS	NEG	POS	POS
Prediction for SK1585, GenBank AYLT	*SCCmec III+ccrC+Cd/Hg (SK1585)*	*CC8 (ST239)*	POS	POS	NEG	AMB	NEG	POS	NEG	POS	AMB	POS	POS	NEG	POS	NEG	POS	NEG	POS	NEG	POS	POS
UKD-105861	SCCmec III+ccrC+Cd/Hg (SK1585)	CC8 (ST239)	POS	POS	NEG	NEG	NEG	POS	NEG	POS	NEG	POS	POS	NEG	POS	NEG	POS	POS	NEG	NEG	POS	POS
UKD-134572	SCCmec III+ccrC+Cd/Hg (SK1585)	CC8 (ST239)	POS						NEG	POS	NEG	POS	POS	NEG	POS	NEG	POS	NEG	POS	NEG	NEG	POS
UKD-139143	SCCmec III+ccrC+Cd/Hg (SK1585)	CC8 (ST239)	POS	POS	NEG	NEG	NEG	POS	NEG	POS	NEG	POS	POS	NEG	POS	NEG	POS	NEG	POS	NEG	POS	POS
Prediction for TW20, GenBank FN433596.1	*SCC [mec III+Cd/Hg+ccrC] (TW20)*	*CC8 (ST239)*	POS	POS	NEG	AMB	NEG	POS	NEG	POS	AMB	POS	POS	NEG	POS	NEG	POS	NEG	POS	NEG	POS	POS
Prediction for ATCC BAA-39, GenBank AEEK	*SCC [mec III+Cd/Hg+ccrC] (TW20)*	*CC8 (ST239)*	POS	POS	NEG	AMB	NEG	POS	NEG	POS	AMB	POS	POS	NEG	POS	NEG	POS	NEG	POS	NEG	POS	POS

Isolate ID	SCCmec type	Clonal complex	VIRULENCE : STAPHYLOCOCCAL SUPERANTIGEN/ENTEROTOXIN-LIKE GENES (SET/SSL)																			
			staphyl. exotoxin-like protein	staphylococcal superantigen-like protein 1						staphylococcal superantigen-like protein 2		staphylococcal superantigen-like protein 3			staphylococcal superantigen-like protein 4		staphylococcal superantigen-like protein 5				staphylococcal superantigen-like protein 6	
			setC / selX	set6-var1_11	set6-var2_11	set6-var1_12	set6-var2_12	set6-var4_11	ssl01-RF122	ssl02/set7	ssl02/set7 (MRSA252)	ssl03/set8_probe1	ssl03/set8_probe2	ssl03/set8 (MRSA252, SAR0424)	ssl04/set9	ssl04/set9 (MRSA252, SAR0425)	ssl05/set3_probe1	ssl05/set3 (RF122, probe-611)	ssl05/set3_probe2 (612)	ssl05/set3 (MRSA252)	ssl06/set21	ssl06 (NCTC8325+MW2)
Prediction for MW2, GenBank BA000033.2	*SCCmec IVa (MW2)*	*CC1*	*POS*	*NEG*	*POS*	*NEG*	*POS*	*NEG*	*NEG*	*POS*	*AMB*	*POS*	*POS*	*NEG*	*POS*	*NEG*	*POS*	*NEG*	*POS*	*NEG*	*POS*	*POS*
**Reference strain MW2**	**SCCmec IVa (MW2)**	**CC1**	**POS**	**NEG**	**POS**	**NEG**	**POS**	**NEG**	**NEG**	**POS**	**NEG**	**POS**	**POS**	**NEG**	**POS**	**NEG**	**POS**	**NEG**	**POS**	**NEG**	**POS**	**POS**
**Reference strain CA05 (JCSC1968)**	**SCCmec IVa (MW2)**	**CC45**	**POS**	**NEG**	**POS**	**POS**	**NEG**	**POS**	**NEG**	**NEG**	**POS**	**NEG**	**NEG**	**NEG**	**NEG**	**POS**	**NEG**	**NEG**	**NEG**	**POS**	**NEG**	**NEG**
**UKD-0127106**	SCCmec IVa (MW2)	CC1	POS	NEG	POS	NEG	POS	NEG	NEG	POS	NEG	POS	POS	NEG	POS	NEG	POS	AMB	POS	NEG	POS	POS
**UKD-0701446**	SCCmec IVa (MW2)	CC1	POS	NEG	POS	NEG	POS	NEG	NEG	POS	AMB	POS	POS	NEG	POS	NEG	POS	AMB	POS	NEG	POS	POS
**UKD-0947486**	SCCmec IVa (MW2)	CC1	POS	NEG	POS	NEG	POS	NEG	NEG	POS	NEG	POS	POS	NEG	POS	NEG	POS	NEG	POS	NEG	POS	POS
**UKD-1031910**	SCCmec IVa (MW2)	CC1	POS	NEG	POS	NEG	POS	NEG	NEG	POS	NEG	POS	POS	NEG	POS	NEG	POS	AMB	POS	NEG	POS	POS
**UKD-14571328**	SCCmec IVa (MW2)	CC1	POS	NEG	POS	NEG	POS	NEG	NEG	POS	AMB	POS	POS	NEG	NEG	NEG	POS	AMB	POS	NEG	POS	POS
**UKD-03003012**	SCCmec IVa (MW2)	CC5	POS						NEG	POS	NEG	POS	POS	NEG	POS	NEG	POS	POS	POS	NEG	NEG	AMB
**UKD-15582582**	SCCmec IVa (MW2)	CC6	POS	NEG	NEG	POS	NEG	POS	POS	POS	AMB	POS	POS	NEG	POS	NEG	POS	AMB	POS	AMB	NEG	POS
**UKD-16574230**	SCCmec IVa (MW2)	CC6	POS	NEG	NEG	POS	NEG	POS	POS	POS	AMB	POS	POS	NEG	POS	AMB	POS	AMB	POS	POS	NEG	AMB
**UKD-16582560**	SCCmec IVa (MW2)	CC6	POS	NEG	NEG	NEG	NEG	POS	POS	POS	NEG	POS	POS	NEG	POS	NEG	POS	POS	NEG	NEG	NEG	NEG
**UKD-0802855**	SCCmec IVa (MW2)	CC7	POS	POS	NEG	NEG	POS	NEG	NEG	POS	NEG	AMB	AMB	NEG	POS	NEG	POS	NEG	POS	NEG	POS	POS
**KHDN-10405258**	SCCmec IVa (MW2)	CC8	POS	POS	NEG	POS	NEG	POS	NEG	POS	AMB	POS	POS	NEG	POS	NEG	POS	AMB	POS	NEG	POS	POS
**UKD-15573497**	SCCmec IVa (MW2)	CC22	POS	NEG	POS	POS	NEG	NEG	NEG	AMB	POS	NEG	AMB	POS	NEG	POS	POS	POS	NEG	POS	NEG	POS
**UKD-0634729**	SCCmec IVa (MW2)	CC30	NEG						NEG	AMB	POS	NEG	NEG	POS	AMB	POS	NEG	POS	NEG	POS	NEG	POS
**UKD-1119771**	SCCmec IVa (MW2)	CC30	NEG	NEG	POS	POS	NEG	POS	NEG	NEG	POS	NEG	AMB	POS	NEG	POS	NEG	NEG	NEG	POS	NEG	POS
**KHDN-10411458**	SCCmec IVa (MW2)	CC30	NEG	NEG	POS	POS	NEG	POS	NEG	AMB	POS	NEG	NEG	POS	NEG	POS	NEG	NEG	NEG	POS	NEG	AMB
**KHDN-398609**	SCCmec IVa (MW2)	CC30	NEG	NEG	POS	POS	NEG	POS	NEG	NEG	POS	NEG	NEG	POS	NEG	POS	NEG	NEG	NEG	POS	NEG	AMB
**KHDN-01081998**	SCCmec IVa (MW2)	CC30	NEG	NEG	POS	POS	NEG	POS	NEG	AMB	POS	NEG	AMB	POS	NEG	POS	NEG	POS	NEG	POS	NEG	POS
**KHDN-10399223**	SCCmec IVa (MW2)	CC30	NEG	NEG	POS	POS	NEG	POS	NEG	NEG	POS	NEG	NEG	POS	NEG	POS	NEG	NEG	NEG	POS	NEG	AMB
**KHDN-W261158**	SCCmec IVa (MW2)	CC30	NEG	NEG	POS	POS	NEG	POS	NEG	NEG	POS	NEG	NEG	POS	NEG	POS	NEG	NEG	NEG	POS	NEG	NEG
**UKD-0002046**	SCCmec IVa (MW2)	CC45 [agr I]	POS	NEG	POS	POS	NEG	POS	NEG	AMB	POS	NEG	NEG	NEG	NEG	POS	NEG	NEG	NEG	POS	NEG	AMB
**UKD-0029618**	SCCmec IVa (MW2)	CC45 [agr I]	AMB	NEG	POS	AMB	NEG	AMB	NEG	AMB	POS	NEG	NEG	NEG	NEG	AMB	NEG	NEG	NEG	POS	NEG	NEG
**UKD-0043085**	SCCmec IVa (MW2)	CC45 [agr I]	AMB	NEG	POS	AMB	NEG	POS	NEG	AMB	POS	NEG	NEG	NEG	NEG	AMB	NEG	NEG	NEG	POS	NEG	NEG
**UKD-0047053**	SCCmec IVa (MW2)	CC45 [agr I]	POS						NEG	NEG	POS	NEG	NEG	NEG	NEG	POS	NEG	NEG	NEG	POS	NEG	NEG
**UKD-0047429**	SCCmec IVa (MW2)	CC45 [agr I]	AMB	NEG	POS	NEG	NEG	AMB	NEG	NEG	POS	NEG	NEG	NEG	NEG	AMB	NEG	NEG	NEG	POS	NEG	NEG
**UKD-0112465**	SCCmec IVa (MW2)	CC45 [agr I]	NEG	NEG	POS	NEG	NEG	AMB	NEG	NEG	POS	NEG	NEG	NEG	NEG	AMB	NEG	NEG	NEG	POS	NEG	NEG
**UKD-0103483**	SCCmec IVa (MW2)	CC45 [agr I]	NEG	NEG	AMB	NEG	NEG	NEG	NEG	NEG	POS	NEG	NEG	NEG	NEG	POS	NEG	NEG	NEG	POS	NEG	NEG
**UKD-0305014**	SCCmec IVa (MW2)	CC45 [agr I]	POS	NEG	POS	AMB	NEG	POS	NEG	AMB	POS	NEG	NEG	NEG	NEG	POS	NEG	NEG	NEG	POS	NEG	AMB
**UKD-0401119**	SCCmec IVa (MW2)	CC45 [agr I]	NEG	NEG	POS	NEG	NEG	AMB	NEG	NEG	POS	NEG	NEG	NEG	NEG	AMB	NEG	NEG	NEG	POS	NEG	NEG
**UKD-0537969**	SCCmec IVa (MW2)	CC45 [agr I]	AMB	NEG	POS	NEG	NEG	POS	NEG	NEG	POS	NEG	NEG	NEG	NEG	NEG	NEG	NEG	NEG	AMB	NEG	AMB
**UKD-0703373**	SCCmec IVa (MW2)	CC45 [agr I]	POS						NEG	NEG	POS	NEG	NEG	NEG	NEG	NEG	NEG	NEG	NEG	NEG	NEG	NEG
**UKD-0813653**	SCCmec IVa (MW2)	CC45 [agr I]	POS	NEG	POS	AMB	NEG	POS	NEG	AMB	POS	NEG	NEG	NEG	NEG	AMB	NEG	NEG	NEG	POS	NEG	AMB
**UKD-0839175**	SCCmec IVa (MW2)	CC45 [agr I]	POS	NEG	POS	POS	NEG	POS	NEG	AMB	POS	NEG	NEG	NEG	NEG	AMB	NEG	NEG	NEG	POS	NEG	POS
**UKD-1108422**	SCCmec IVa (MW2)	CC45 [agr I]	POS	NEG	POS	POS	NEG	POS	NEG	AMB	POS	NEG	NEG	NEG	NEG	POS	NEG	NEG	NEG	POS	NEG	POS
**UKD-1380645**	SCCmec IVa (MW2)	CC45 [agr I]	POS	NEG	POS	POS	NEG	POS	NEG	NEG	POS	NEG	NEG	NEG	NEG	POS	NEG	NEG	NEG	POS	NEG	AMB
**UKD-148016**	SCCmec IVa (MW2)	CC45 [agr I]	POS	NEG	POS	POS	NEG	POS	NEG	AMB	POS	NEG	NEG	NEG	NEG	POS	NEG	NEG	NEG	POS	NEG	POS
**UKD-16571773**	SCCmec IVa (MW2)	CC45 [agr I]	POS	NEG	POS	POS	NEG	POS	NEG	AMB	POS	NEG	NEG	NEG	NEG	POS	NEG	NEG	NEG	POS	NEG	POS
**KHDN-10407738**	SCCmec IVa (MW2)	CC45 [ST617]	POS	NEG	POS	POS	NEG	POS	NEG	AMB	POS	NEG	NEG	NEG	NEG	POS	NEG	NEG	NEG	POS	NEG	POS
**UKD-0853670**	SCCmec IVa (MW2)	CC88	POS	NEG	POS	NEG	NEG	POS	NEG	POS	NEG	POS	POS	NEG	POS	NEG	NEG	AMB	POS	NEG	AMB	POS
**UKD-1417352**	SCCmec IVa (MW2)	CC88	POS	NEG	POS	POS	NEG	POS	POS	POS	AMB	POS	POS	NEG	POS	POS	NEG	POS	POS	NEG	POS	POS
**UKD-15571283**	SCCmec IVa (MW2)	CC88	POS	NEG	POS	POS	NEG	POS	POS	POS	AMB	POS	POS	NEG	POS	POS	NEG	AMB	POS	NEG	POS	POS
**KHDN-10406719**	SCCmec IVa (MW2)	ST93	POS	NEG	POS	NEG	POS	NEG	POS	NEG	NEG	NEG	NEG	NEG	AMB	POS	NEG	POS	AMB	NEG	POS	POS
**KHDN-0060294**	SCCmec IVa (MW2)	ST93	POS	NEG	NEG	NEG	POS	NEG	POS	NEG	NEG	NEG	NEG	NEG	NEG	NEG	NEG	NEG	NEG	NEG	POS	AMB
**UKD-0734190**	SCCmec IVa (MW2)	CC97	POS						POS	POS	NEG	NEG	NEG	NEG	POS	NEG	POS	POS	NEG	NEG	NEG	NEG
**UKD-13006983**	SCCmec IVa (MW2)	CC97	POS	NEG	NEG	POS	NEG	POS	POS	POS	AMB	POS	POS	NEG	POS	POS	POS	AMB	POS	POS	NEG	POS
Prediction for H131520133	*SCCmec IVa (H131520133)*	*CC152*	*NEG*	*NEG*	*NEG*	*NEG*	*POS*	*NEG*	*POS*	*AMB*	*AMB*	*NEG*	*NEG*	*NEG*	*NEG*	*NEG*	*NEG*	*NEG*	*NEG*	*NEG*	*NEG*	*NEG*
**KHDN-325892**	SCCmec IVa (H131520133)	CC30	NEG	NEG	POS	POS	NEG	POS	NEG	AMB	POS	NEG	POS	POS	AMB	POS	NEG	NEG	NEG	POS	NEG	POS
**UKD-15580207**	SCCmec IVa (H131520133)	CC88	POS	NEG	POS	POS	NEG	POS	POS	POS	AMB	POS	POS	NEG	POS	POS	NEG	AMB	POS	NEG	POS	POS
**UKD-0521159**	SCCmec Iva	CC8	POS						NEG	POS	NEG	POS	POS	NEG	POS	NEG	POS	AMB	POS	NEG	NEG	POS
Prediction for CMFT503 : HF569113.1	*SCCmec IVa (CMFT503)*																					
Prediction for 21343, GenBank AHKV	*SCCmec IVa (CMFT503)*	*CC88*	*POS*	*NEG*	*AMB*	*AMB*	*NEG*	*POS*	*NEG*	*POS*	*AMB*	*POS*	*POS*	*NEG*	*POS*	*NEG*	*NEG*	*NEG*	*POS*	*NEG*	*POS*	*POS*
**UKD-1420740**	SCCmec IVa (CMFT503)	CC22	POS	NEG	POS	POS	NEG	POS	AMB	AMB	POS	POS	POS	POS	NEG	POS	POS	POS	NEG	POS	NEG	POS
**UKD-15575124**	SCCmec IVa (CMFT503)	CC22	POS	NEG	POS	POS	NEG	NEG	POS	AMB	POS	NEG	NEG	POS	NEG	POS	POS	POS	NEG	POS	NEG	AMB
**UKD-16584947**	SCCmec IVa (CMFT503)	CC22	POS	NEG	POS	POS	NEG	NEG	NEG	POS	AMB	NEG	NEG	NEG	NEG	POS	POS	NEG	NEG	POS	NEG	NEG
**UKD-16575077**	SCCmec IVa (CMFT503)	CC88	POS	NEG	POS	POS	NEG	POS	NEG	POS	NEG	POS	POS	NEG	POS	NEG	NEG	AMB	POS	NEG	POS	POS
Prediction for TCH1516, GenBank CP000730.1	*SCCmec IVa+ACME1+Cu (USA300)*	*CC8*	*POS*	*POS*	*NEG*	*AMB*	*NEG*	*POS*	*NEG*	*POS*	*AMB*	*POS*	*POS*	*NEG*	*POS*	*NEG*	*POS*	*NEG*	*POS*	*NEG*	*POS*	*POS*
Reference strain USA300-TCH1516	SCCmec IVa+ACME1+Cu (USA300)	CC8	POS	POS	NEG	AMB	NEG	POS	NEG	POS	NEG	POS	POS	NEG	POS	NEG	POS	AMB	POS	NEG	POS	POS
Prediction for FPR3757, GenBank CP000255.1	*SCCmec IVa+ACME1+Cu (USA300)*	*CC8*	*POS*	*POS*	*NEG*	*AMB*	*NEG*	*POS*	*NEG*	*POS*	*AMB*	*POS*	*POS*	*NEG*	*POS*	*NEG*	*POS*	*NEG*	*POS*	*NEG*	*POS*	*POS*
**UKD-0516386**	SCCmec IVa+ACME1+Cu (USA300)	CC8	POS						AMB	POS	NEG	POS	POS	NEG	POS	NEG	POS	AMB	POS	NEG	NEG	POS
**UKD-0815773**	SCCmec IVa+ACME1+Cu (USA300)	CC8	POS						NEG	POS	AMB	POS	POS	NEG	POS	NEG	POS	NEG	POS	NEG	POS	POS
**UKD-1414654**	SCCmec IVa+ACME1+Cu (USA300)	CC8	POS	POS	NEG	POS	NEG	POS	NEG	POS	AMB	POS	POS	NEG	POS	NEG	POS	AMB	POS	NEG	POS	POS
**UKD-0408036**	SCCmec IVa+ACME2	CC45 [agr I]	POS	NEG	POS	POS	NEG	POS	NEG	AMB	POS	NEG	NEG	NEG	NEG	POS	NEG	NEG	NEG	POS	NEG	POS
**UKD-0044035**	SCCmec IVa+ccrC (Hannover)	CC8	POS						NEG	POS	NEG	POS	POS	NEG	POS	NEG	POS	NEG	POS	NEG	NEG	POS
**UKD-0136287**	SCCmec IVa+ccrC (Hannover)	CC8	POS	POS	NEG	AMB	NEG	POS	NEG	POS	AMB	POS	POS	NEG	POS	NEG	POS	POS	NEG	NEG	POS	POS

Isolate ID	SCCmec type	Clonal complex	VIRULENCE : STAPHYLOCOCCAL SUPERANTIGEN/ENTEROTOXIN-LIKE GENES (SET/SSL)																			
			staphyl. exotoxin-like protein	staphylococcal superantigen-like protein 1						staphylococcal superantigen-like protein 2		staphylococcal superantigen-like protein 3			staphylococcal superantigen-like protein 4		staphylococcal superantigen-like protein 5				staphylococcal superantigen-like protein 6	
			setC / selX	set6-var1_11	set6-var2_11	set6-var1_12	set6-var2_12	set6-var4_11	ssl01-RF122	ssl02/set 7	ssl02/set 7 (MRSA252)	ssl03/set 8_probe 1	ssl03/set 8_probe 2	ssl03/set 8 (MRSA252, SAR0424)	ssl04/set 9	ssl04/set 9 (MRSA252, SAR0425)	ssl05/set 3_probe 1	ssl05/set 3 (RF122, probe-611)	ssl05/set 3_probe 2 (612)	ssl05/set 3 (MRSA252)	ssl06/set 21	ssl06 (NCTC8325+MW2)
Prediction for JCSC1978, GenBank AB063173.1	*SCCmec IVb/d/i (JCSC1978/6668/4469)*																					
Reference strain JCSC1978 (8/6-3P)	SCCmec IVb/d/i (JCSC1978/6668/4469)	CC8	POS	POS	NEG	POS	NEG	POS	NEG	POS	AMB	POS	POS	NEG	POS	NEG	POS	AMB	POS	NEG	POS	POS
Prediction for JCSC6668, GenBank AB425823.1	*SCCmec IVb/d/i (JCSC1978/6668/4469)*																					
Reference strain JCSC4469	SCCmec IVb/d/i (JCSC1978/6668/4469)	CC5	POS	POS	NEG	POS	NEG	POS	NEG	POS	AMB	POS	POS	NEG	POS	AMB	POS	AMB	POS	NEG	NEG	AMB
UKD-1103429	SCCmec IVb/d/i (JCSC1978/6668/4469)	CC7	POS	POS	NEG	NEG	POS	NEG	NEG	POS	AMB	POS	POS	NEG	POS	NEG	POS	NEG	POS	NEG	POS	POS
UKD-1306141	SCCmec IVb/d/i (JCSC1978/6668/4469)	CC7	POS	POS	NEG	NEG	POS	NEG	NEG	POS	AMB	POS	POS	NEG	POS	AMB	POS	NEG	POS	NEG	POS	POS
UKD-0500089	SCCmec IVb/d/i (JCSC1978/6668/4469)	CC5	POS						NEG	POS	AMB	POS	POS	NEG	POS	NEG	POS	NEG	POS	NEG	NEG	NEG
UKD-0501416	SCCmec IVb/d/i (JCSC1978/6668/4469)	CC5	POS	POS	NEG	POS	NEG	NEG	NEG	POS	NEG	POS	POS	NEG	NEG	NEG	AMB	NEG	POS	NEG	NEG	NEG
UKD-0934574	SCCmec IVb/d/i (JCSC1978/6668/4469)	CC5	POS	POS	NEG	POS	NEG	POS	NEG	POS	NEG	POS	POS	NEG	POS	NEG	POS	NEG	POS	NEG	NEG	NEG
UKD-0746779	SCCmec IVb/d/i (JCSC1978/6668/4469)	CC45 [agr I]	POS						NEG	POS	POS	NEG	NEG	NEG	NEG	POS	NEG	NEG	NEG	POS	NEG	POS
UKD-0812518	SCCmec IVb/d/i (JCSC1978/6668/4469)	CC45 [agr I]	POS	NEG	POS	NEG	NEG	AMB	NEG	NEG	POS	NEG	NEG	NEG	NEG	POS	NEG	NEG	NEG	POS	NEG	NEG
Prediction for 21209, GenBank AGRP	*SCCmec IVb/d/i (21209)*	*CC8*	*POS*	*POS*	*NEG*	*AMB*	*NEG*	*POS*	*NEG*	*POS*	*AMB*	*POS*	*POS*	*NEG*	*POS*	*NEG*	*POS*	*NEG*	*POS*	*NEG*	*POS*	*POS*
UKD-0233579	SCCmec IVb/d/i (21209)	CC8	POS						NEG	POS	NEG	POS	POS	NEG	POS	NEG	POS	AMB	POS	NEG	NEG	POS
UKD-0504920	SCCmec IVb/d/i (21209)	CC8	POS						NEG	POS	NEG	POS	POS	NEG	POS	NEG	POS	NEG	POS	NEG	NEG	POS
UKD-0725999	SCCmecIV b/d/i+Cu	CC30	NEG	NEG	POS	POS	NEG	POS	NEG	NEG	POS	NEG	NEG	POS	NEG	POS	NEG	NEG	NEG	POS	NEG	AMB
Prediction for IS-105, GenBank AHLR	*SCCmec IVc (IS-105)*	*CC22*	*POS*	*NEG*	*POS*	*POS*	*NEG*	*NEG*	*NEG*	*AMB*	*AMB*	*NEG*	*NEG*	*POS*	*NEG*	*AMB*	*POS*	*NEG*	*NEG*	*POS*	*NEG*	*NEG*
UKD-0402421	SCCmec IVc (IS-105), merA/B+	CC5	POS	POS	NEG	POS	NEG	POS	NEG	POS	NEG	POS	POS	NEG	POS	NEG	POS	POS	POS	NEG	NEG	NEG
UKD-0731319	SCCmec IVc (IS-105), merA/B+	CC5	POS						NEG	POS	POS	POS	POS	NEG	POS	NEG	POS	AMB	POS	NEG	NEG	NEG
UKD-0733137	SCCmec IVc (IS-105)	CC8	POS	POS	NEG	NEG	NEG	POS	NEG	POS	NEG	POS	POS	NEG	POS	NEG	POS	NEG	POS	NEG	AMB	POS
UKD-0305690	SCCmec IVc (IS-105)	CC22	POS	NEG	POS	NEG	NEG	NEG	NEG	POS	AMB	NEG	NEG	NEG	NEG	POS	POS	NEG	NEG	POS	NEG	NEG
UKD-1424706	SCCmec IVc (IS-105)	CC22	POS	NEG	POS	POS	NEG	NEG	NEG	AMB	POS	NEG	NEG	POS	NEG	POS	POS	POS	NEG	POS	NEG	AMB
Prediction for TCH60, GenBank CP002110.1	*SCCmec IVc (TCH60)*	*CC30*	*NEG*	*NEG*	*POS*	*POS*	*NEG*	*AMB*	*NEG*	*AMB*	*POS*	*NEG*	*NEG*	*POS*	*NEG*	*POS*	*NEG*	*NEG*	*NEG*	*POS*	*NEG*	*NEG*
Reference strain JCSC4788	SCCmec IVc (TCH60)	CC8	POS	POS	NEG	POS	NEG	POS	NEG	POS	AMB	POS	POS	NEG	POS	NEG	POS	AMB	POS	NEG	POS	POS
UKD-1318318	SCCmec IVc (TCH60)	CC5	POS	POS	NEG	POS	NEG	POS	NEG	POS	AMB	POS	POS	NEG	POS	NEG	POS	AMB	POS	NEG	NEG	NEG
UKD-14580050	SCCmec IVc (TCH60)	CC5	POS	POS	NEG	POS	NEG	POS	NEG	POS	AMB	POS	POS	NEG	POS	AMB	POS	AMB	POS	NEG	NEG	NEG
UKD-08F01314	SCCmec IVc (TCH60)	CC8	POS						NEG	POS	NEG	POS	POS	NEG	POS	NEG	POS	NEG	POS	NEG	AMB	POS
KHDN-50361518	SCCmec IVc (TCH60)	CC8	POS	POS	NEG	POS	NEG	POS	NEG	POS	AMB	POS	POS	NEG	POS	NEG	POS	POS	AMB	NEG	POS	POS
UKD-0307912	SCCmec IVc (TCH60)	CC22	POS						NEG	POS	POS	NEG	NEG	POS	NEG	POS	POS	AMB	NEG	POS	NEG	NEG
UKD-0220390	SCCmec IVc (TCH60)	CC45 [agr I]	NEG	NEG	POS	NEG	NEG	NEG	NEG	NEG	POS	NEG	NEG	NEG	NEG	POS	NEG	NEG	NEG	POS	NEG	NEG
UKD-0311106	SCCmec IVc (TCH60)	CC45 [agr I]	POS	NEG	POS	POS	NEG	POS	NEG	AMB	POS	NEG	NEG	NEG	NEG	POS	NEG	NEG	NEG	POS	NEG	POS
UKD-0719855	SCCmec IVc (TCH60)	CC45 [agr I]	POS						NEG	AMB	POS	NEG	NEG	NEG	NEG	POS	NEG	NEG	NEG	POS	NEG	POS
UKD-0422079	SCCmec IVc (TCH60)	CC45 [agr I]	AMB	NEG	POS	NEG	NEG	AMB	NEG	NEG	POS	NEG	NEG	NEG	NEG	POS	NEG	NEG	NEG	POS	NEG	NEG
UKD-0409235	SCCmec IVc (TCH60)	CC80	POS	NEG	NEG	POS	NEG	POS	POS	POS	NEG	POS	POS	NEG	POS	NEG	POS	POS	AMB	NEG	NEG	NEG
UKD-0409807	SCCmec IVc (TCH60)	CC80	POS	NEG	NEG	POS	NEG	POS	POS	POS	NEG	POS	POS	NEG	POS	AMB	POS	AMB	POS	NEG	NEG	POS
UKD-0416073	SCCmec IVc (TCH60)	CC80	POS						POS	POS	NEG	POS	POS	NEG	POS	NEG	POS	POS	POS	NEG	NEG	NEG
UKD-0431728	SCCmec IVc (TCH60)	CC80	POS	NEG	NEG	NEG	NEG	POS	POS	POS	NEG	AMB	POS	NEG	POS	NEG	POS	POS	NEG	NEG	NEG	NEG
UKD-1001508	SCCmec IVc (TCH60)	CC80	POS	NEG	POS	POS	NEG	POS	POS	POS	AMB	POS	POS	NEG	POS	AMB	POS	AMB	POS	AMB	NEG	NEG
UKD-1010412	SCCmec IVc (TCH60)	CC80	POS	NEG	NEG	POS	NEG	POS	AMB	POS	AMB	POS	AMB	NEG	POS	NEG	POS	POS	NEG	AMB	NEG	AMB
UKD-12753005	SCCmec IVc (TCH60)	CC80	POS	NEG	NEG	POS	NEG	POS	POS	POS	AMB	POS	POS	NEG	POS	NEG	POS	AMB	POS	NEG	NEG	NEG
UKD-15574809	SCCmec IVc (TCH60)	CC80	POS	NEG	NEG	POS	NEG	POS	POS	POS	AMB	POS	POS	NEG	POS	NEG	POS	POS	AMB	NEG	NEG	NEG
KHDN-16120291	SCCmec IVc (TCH60)	CC88	POS	NEG	NEG	NEG	NEG	POS	NEG	POS	NEG	POS	POS	NEG	POS	NEG	NEG	NEG	NEG	POS	POS	POS
Prediction for WW2703/97, GenBank ACSW	*SCCmec IVc (WW2703/97)*	*CC30*	*NEG*	*NEG*	*POS*	*POS*	*NEG*	*AMB*	*NEG*	*AMB*	*POS*	*NEG*	*NEG*	*POS*	*NEG*	*POS*	*NEG*	*NEG*	*NEG*	*POS*	*NEG*	*NEG*
UKD-14580088	SCCmec IVc (WW2703/97)	CC398	POS	POS	NEG	POS	NEG	POS	NEG	NEG	POS	NEG	NEG	NEG	NEG	POS	NEG	NEG	NEG	POS	NEG	NEG
Prediction for SA_ST125_MupR, GenBank ASTH	*SCCmec IVc+speG+ccrAB4 (SA_ST125)*	*CC5*	*POS*	*POS*	*NEG*	*POS*	*NEG*	*AMB*	*NEG*	*POS*	*AMB*	*POS*	*POS*	*NEG*	*POS*	*NEG*	*POS*	*NEG*	*POS*	*NEG*	*NEG*	*NEG*
UKD-0439909	SCCmec IVc+speG+ccrAB4 (SA_ST125)	CC5	POS						NEG	POS	NEG	POS	POS	NEG	POS	NEG	POS	NEG	POS	NEG	NEG	NEG
Prediction for MRSA177, GenBank AECP	*SCCmec IVc+Cu/Hg (MRSA177)*		*POS*	*POS*	*NEG*	*AMB*	*NEG*	*POS*	*NEG*	*POS*	*AMB*	*POS*	*POS*	*NEG*	*POS*	*NEG*	*POS*	*NEG*	*POS*	*NEG*	*POS*	*POS*
UKD-1279734	SCCmec IVc+Cu/Hg (MRSA177)	CC8	POS	POS	NEG	POS	NEG	POS	NEG	POS	AMB	POS	POS	NEG	POS	NEG	POS	POS	AMB	NEG	POS	POS
UKD-14580899	SCCmec IVc+Cu/Hg (MRSA177)	CC8	POS	POS	NEG	POS	NEG	POS	NEG	POS	AMB	POS	POS	NEG	POS	NEG	POS	POS	AMB	NEG	POS	POS
UKD-16002228	SCCmec IVc+Cu/Hg (MRSA177)	CC8	POS	POS	NEG	POS	NEG	POS	NEG	POS	AMB	POS	POS	NEG	POS	NEG	POS	AMB	POS	NEG	POS	POS
Prediction for HO 5096 0412, GenBank HE681097.1	*SCCmec IVh/j (HO50960412/JCSC6670)*	*CC22*	*POS*	*NEG*	*POS*	*POS*	*NEG*	*NEG*	*NEG*	*AMB*	*AMB*	*NEG*	*NEG*	*POS*	*NEG*	*AMB*	*POS*	*NEG*	*NEG*	*POS*	*NEG*	*NEG*
Prediction for JCSC6670, GenBank AB425824.1	*SCCmec IVh/j (HO50960412/JCSC6670)*																					
Reference strain Dublin_DSH_E1749	SCCmec IVh/j (HO50960412/JCSC6670)		POS	NEG	POS	POS	NEG	NEG	NEG	POS	AMB	NEG	NEG	POS	NEG	POS	POS	NEG	NEG	POS	NEG	NEG
UKD-14576248	SCCmec IVh/j (HO50960412/JCSC6670)	CC8	POS	POS	NEG	POS	NEG	POS	NEG	POS	AMB	POS	POS	NEG	POS	NEG	POS	AMB	POS	NEG	POS	POS
UKD-0120122	SCCmec IVh/j (HO50960412/JCSC6670)	CC22	POS	NEG	POS	POS	NEG	NEG	NEG	AMB	POS	NEG	NEG	NEG	NEG	POS	POS	NEG	NEG	POS	NEG	NEG
UKD-0205319	SCCmec IVh/j (HO50960412/JCSC6670)	CC22	POS	NEG	POS	POS	NEG	NEG	NEG	AMB	POS	NEG	NEG	AMB	NEG	AMB	POS	NEG	NEG	POS	NEG	NEG
UKD-0221259	SCCmec IVh/j (HO50960412/JCSC6670)	CC22	POS	NEG	POS	POS	NEG	NEG	NEG	AMB	POS	NEG	NEG	NEG	NEG	POS	POS	NEG	NEG	POS	NEG	NEG
UKD-0418654	SCCmec IVh/j (HO50960412/JCSC6670)	CC22	POS	NEG	POS	NEG	NEG	NEG	NEG	AMB	POS	NEG	NEG	NEG	NEG	POS	POS	NEG	NEG	POS	NEG	NEG
UKD-0429721	SCCmec IVh/j (HO50960412/JCSC6670)	CC22	POS	NEG	POS	NEG	NEG	NEG	NEG	NEG	POS	NEG	NEG	NEG	NEG	POS	AMB	NEG	NEG	POS	NEG	NEG
UKD-1116194	SCCmec IVh/j (HO50960412/JCSC6670)	CC22	POS	NEG	POS	POS	NEG	NEG	NEG	AMB	POS	NEG	NEG	POS	NEG	POS	NEG	NEG	NEG	POS	NEG	POS
UKD-1122367	SCCmec IVh/j (HO50960412/JCSC6670)	CC22	POS	NEG	POS	POS	NEG	NEG	NEG	AMB	POS	NEG	NEG	POS	NEG	POS	NEG	NEG	NEG	POS	NEG	POS
UKD-14012840	SCCmec IVh/j (HO50960412/JCSC6670)	CC22	POS	NEG	POS	POS	NEG	AMB	NEG	POS	AMB	NEG	NEG	POS	NEG	POS	POS	POS	NEG	POS	NEG	POS
UKD-14579833	SCCmec IVh/j (HO50960412/JCSC6670)	CC22	POS	NEG	POS	POS	NEG	NEG	NEG	AMB	POS	NEG	NEG	POS	NEG	POS	POS	POS	NEG	POS	NEG	AMB
UKD-15579198	SCCmec IVh/j (HO50960412/JCSC6670)	CC22	POS	NEG	POS	POS	NEG	NEG	NEG	AMB	POS	POS	NEG	POS	NEG	POS	POS	POS	NEG	POS	NEG	POS
UKD-4052193	SCCmec IVh/j (HO50960412/JCSC6670)	CC22	POS	NEG	POS	POS	NEG	NEG	NEG	POS	AMB	AMB	NEG	POS	NEG	POS	POS	POS	NEG	POS	NEG	POS
UKD-124968	SCCmec IVh/j (HO50960412/JCSC6670)	CC22	POS	NEG	POS	POS	NEG	NEG	NEG	AMB	POS	NEG	NEG	AMB	NEG	POS	POS	POS	NEG	POS	NEG	NEG
UKD-14572546	SCCmec IVh/j (HO50960412/JCSC6670)	CC22	POS	NEG	POS	POS	NEG	AMB	AMB	AMB	POS	POS	AMB	POS	NEG	POS	POS	POS	NEG	POS	NEG	POS
UKD-14582287	SCCmec IVh/j (HO50960412/JCSC6670)	CC22	POS	NEG	POS	POS	NEG	POS	AMB	AMB	POS	POS	POS	POS	NEG	POS	POS	POS	NEG	POS	NEG	POS
UKD-14583376	SCCmec IVh/j (HO50960412/JCSC6670)	CC22	POS	NEG	POS	POS	NEG	POS	AMB	AMB	POS	POS	POS	POS	NEG	POS	POS	POS	NEG	POS	NEG	POS
UKD-14583496	SCCmec IVh/j (HO50960412/JCSC6670)	CC22	POS	NEG	POS	POS	NEG	POS	POS	AMB	POS	POS	AMB	POS	NEG	POS	POS	POS	NEG	POS	NEG	POS
UKD-0013324	SCCmec IVh/j (HO50960412/JCSC6670)	CC22	POS	NEG	POS	POS	NEG	NEG	NEG	AMB	POS	NEG	NEG	AMB	NEG	POS	POS	NEG	NEG	POS	NEG	NEG
UKD-0125329	SCCmec IVh/j (HO50960412/JCSC6670)	CC22	POS	NEG	POS	AMB	NEG	NEG	NEG	AMB	POS	NEG	NEG	NEG	NEG	AMB	POS	NEG	NEG	POS	NEG	NEG
UKD-0215498	SCCmec IVh/j (HO50960412/JCSC6670)	CC22	POS	NEG	POS	POS	NEG	NEG	NEG	AMB	POS	NEG	NEG	NEG	NEG	POS	POS	NEG	NEG	POS	NEG	NEG
Prediction for M08-0126, GenBank FR753166.1	*SCCmec IVh/j+ACME2 (M08-0126)*	*CC22*																				
UKD-1379483I	SCCmec IVh/j+ACME2 (M08-0126)	CC22	POS	NEG	POS	POS	NEG	NEG	NEG	AMB	POS	AMB	NEG	POS	NEG	POS	POS	POS	NEG	POS	NEG	NEG
UKD-5150204	SCCmec IV+speG+Cu/czrC+ccrAB4	CC22	POS	NEG	POS	POS	NEG	AMB	NEG	AMB	POS	AMB	NEG	POS	NEG	POS	POS	POS	NEG	POS	NEG	POS
UKD-15578423	SCCmec IV+speG+czrC+ccrAB4 var. 1	CC22	POS	NEG	POS	POS	NEG	NEG	NEG	AMB	POS	AMB	NEG	POS	NEG	POS	POS	POS	NEG	POS	NEG	POS
UKD-16582870	SCCmec IV+speG+czrC+ccrAB4 var. 1	CC22	POS	NEG	POS	POS	NEG	NEG	NEG	POS	AMB	NEG	NEG	NEG	NEG	POS	NEG	NEG	NEG	POS	NEG	NEG
UKD-15571940	SCCmec IV+speG+czrC+ccrAB4 var. 2	CC22	POS	NEG	POS	POS	NEG	NEG	NEG	AMB	POS	NEG	NEG	POS	NEG	POS	POS	POS	NEG	POS	NEG	POS

Isolate ID	SCCmec type	Clonal complex	VIRULENCE : STAPHYLOCOCCAL SUPERANTIGEN/ENTEROTOXIN-LIKE GENES (SET/SSL)																			
			staphyl. exotoxin-like protein	staphylococcal superantigen-like protein 1						staphylococcal superantigen-like protein 2		staphylococcal superantigen-like protein 3			staphylococcal superantigen-like protein 4		staphylococcal superantigen-like protein 5				staphylococcal superantigen-like protein 6	
			setC / selX	set6-var1_11	set6-var2_11	set6-var1_12	set6-var2_12	set6-var4_11	ssl01-RF122	ssl02/set 7	ssl02/set 7 (MRSA252)	ssl03/set 8_probe 1	ssl03/set 8_probe 2	ssl03/set 8 (MRSA252, SAR0424)	ssl04/set 9	ssl04/set 9 (MRSA252, SAR0425)	ssl05/set 3_probe 1	ssl05/set 3 (RF122, probe-611)	ssl05/set 3_probe 2 (612)	ssl05/set 3 (MRSA252)	ssl06/set 21	ssl06 (NCTC8325+MW2)
Prediction for CN1, GenBank CP003979.1	*SCCmec IVA (CN1)*	*CC8 (ST72)*	*POS*	*POS*	*NEG*	*NEG*	*NEG*	*NEG*	*NEG*	*POS*	*AMB*	*POS*	*POS*	*NEG*	*POS*	*NEG*	*POS*	*POS*	*NEG*	*NEG*	*NEG*	*NEG*
**UKD-0900871**	SCCmec IVA (CN1)	CC8 (ST72)	POS	POS	NEG	NEG	NEG	NEG	NEG	POS	NEG	POS	POS	NEG	POS	NEG	POS	POS	AMB	NEG	NEG	NEG
Prediction for CMFT1723, GenBank HF569096.1	*SCCmec V (Bengal Bay)*																					
**KHDN-10405134**	SCCmec V (Bengal Bay)	CC1	POS	NEG	POS	NEG	POS	NEG	NEG	POS	NEG	POS	POS	NEG	POS	NEG	POS	NEG	POS	NEG	POS	POS
**UKD-0932405**	SCCmec V (Bengal Bay)	CC1 (ST573/772)	POS	POS	NEG	NEG	NEG	POS	NEG	POS	NEG	POS	POS	NEG	POS	NEG	POS	POS	AMB	NEG	NEG	NEG
**UKD-1003510**	SCCmec V (Bengal Bay)	CC1 (ST573/772)	POS	POS	NEG	POS	NEG	POS	NEG	POS	AMB	POS	POS	NEG	POS	AMB	POS	AMB	POS	AMB	NEG	NEG
**UKD-1112420**	SCCmec V (Bengal Bay)	CC1 (ST573/772)	POS	POS	NEG	POS	NEG	POS	NEG	POS	AMB	POS	POS	NEG	POS	AMB	AMB	AMB	POS	NEG	NEG	NEG
**UKD-1275832**	SCCmec V (Bengal Bay)	CC1 (ST573/772)	POS	POS	NEG	POS	NEG	POS	NEG	POS	AMB	POS	POS	NEG	POS	AMB	POS	AMB	POS	NEG	NEG	AMB
**UKD-13023811**	SCCmec V (Bengal Bay)	CC1 (ST573/772)	POS	POS	NEG	POS	NEG	POS	NEG	POS	AMB	POS	POS	NEG	POS	NEG	POS	AMB	POS	NEG	NEG	NEG
**UKD-14572163**	SCCmec V	CC5	POS	POS	NEG	POS	NEG	POS	NEG	POS	AMB	POS	POS	NEG	POS	AMB	POS	AMB	POS	NEG	NEG	POS
**KHDN-10421133**	SCCmec V+fus	CC97	POS	NEG	NEG	NEG	NEG	AMB	POS	POS	AMB	POS	POS	NEG	POS	AMB	POS	AMB	POS	AMB	NEG	POS
**KHDN-21343541**	SCCmec V+fus+tir+ccrAB1	CC1	POS	NEG	POS	NEG	POS	NEG	NEG	POS	AMB	POS	POS	NEG	POS	NEG	POS	AMB	POS	NEG	POS	POS
**KHDN-10416893**	SCCmec V+czrC+speG+ccrAB4	CC22	POS	NEG	POS	POS	NEG	NEG	NEG	AMB	POS	NEG	NEG	POS	NEG	POS	POS	NEG	NEG	POS	POS	NEG
Prediction for GR1, GenBank AJLX	*SCCmec VT (GR1)*	*CC361*	*POS*	*NEG*	*POS*	*NEG*	*POS*	*NEG*	*NEG*	*POS*	*NEG*	*POS*	*POS*	*NEG*	*POS*	*NEG*	*POS*	*POS*	*NEG*	*NEG*	*NEG*	*NEG*
**UKD-0422872**	SCCmec VT (GR1)	CC152	NEG	NEG	NEG	NEG	POS	NEG	POS	AMB	POS	NEG	NEG	NEG	NEG	NEG	NEG	NEG	NEG	NEG	NEG	NEG
Prediction for PM1, GenBank BAFA	*SCCmec VT (PM1)*	*CC59*	*POS*	*POS*	*NEG*	*POS*	*NEG*	*AMB*	*NEG*	*POS*	*NEG*	*POS*	*AMB*	*NEG*	*POS*	*NEG*	*POS*	*POS*	*NEG*	*NEG*	*NEG*	*NEG*
**KHDN-000871**	SCCmec VT (PM1)	CC45 (agr I)	POS	NEG	POS	POS	NEG	POS	NEG	NEG	POS	NEG	NEG	NEG	NEG	POS	NEG	NEG	NEG	POS	NEG	POS
**UKD-0725052**	SCCmec VT (PM1)	CC59	POS						NEG	NEG	NEG	POS	POS	NEG	POS	NEG	POS	POS	NEG	NEG	NEG	NEG
**UKD-1312138**	SCCmec VT (PM1)	CC59	POS	POS	AMB	POS	NEG	POS	NEG	POS	NEG	POS	POS	NEG	POS	AMB	POS	AMB	POS	POS	NEG	NEG
**UKD-Z4728**	SCCmec VT (PM1)	CC59	POS	POS	NEG	POS	NEG	POS	NEG	POS	NEG	POS	POS	NEG	POS	AMB	POS	AMB	POS	NEG	NEG	AMB
**UKD-K0199**	SCCmec VT unknown var.1	CC7	POS	POS	NEG	NEG	AMB	NEG	NEG	POS	NEG	POS	POS	NEG	POS	NEG	POS	NEG	POS	NEG	POS	POS
**UKD-15584619**	SCCmec VT unknown var.2	CC30	NEG	NEG	POS	POS	NEG	POS	NEG	AMB	POS	NEG	POS	POS	AMB	POS	NEG	POS	NEG	POS	NEG	POS
**UKD-0712568**	SCCmec VT unknown var.3	CC45 (agr I)	POS	NEG	POS	NEG	NEG	POS	NEG	NEG	POS	NEG	NEG	NEG	NEG	POS	NEG	NEG	NEG	POS	NEG	NEG
**KHDN-10420296**	SCCmec VT+ACME2	CC45 (agr I)	POS	NEG	POS	POS	NEG	POS	NEG	AMB	POS	NEG	NEG	NEG	NEG	POS	NEG	NEG	NEG	POS	NEG	POS
**UKD-15577956**	SCCmec VT+Cu/czrC+ccrA1	CC398	POS	POS	NEG	POS	NEG	POS	NEG	AMB	POS	NEG	NEG	NEG	NEG	POS	NEG	NEG	NEG	POS	NEG	NEG
Prediction for S0385, GenBank AM990992	*SCCmec VT+czrC (SO385)*	*CC398*	*POS*	*POS*	*NEG*	*POS*	*NEG*	*AMB*	*NEG*	*AMB*	*POS*	*NEG*	*NEG*	*NEG*	*NEG*	*POS*	*NEG*	*NEG*	*NEG*	*POS*	*NEG*	*NEG*
**UKD-0628560**	SCCmec VT+czrC (SO385)	CC8	POS						NEG	POS	AMB	POS	POS	NEG	POS	NEG	POS	POS	POS	NEG	AMB	POS
**UKD-0510018**	SCCmec VT+czrC (SO385)	CC398	POS						NEG	NEG	POS	NEG	NEG	NEG	NEG	POS	NEG	NEG	NEG	POS	NEG	NEG
**UKD-0903558**	SCCmec VT+czrC (SO385)	CC398	POS	POS	NEG	POS	NEG	AMB	NEG	NEG	POS	NEG	NEG	NEG	NEG	POS	NEG	NEG	NEG	POS	NEG	NEG
**UKD-0929815**	SCCmec VT+czrC (SO385)	CC398	POS	POS	NEG	POS	NEG	AMB	NEG	NEG	POS	NEG	NEG	NEG	NEG	POS	NEG	NEG	NEG	POS	NEG	AMB
**UKD-0907762**	SCCmec VT+czrC (SO385)	CC398	POS	POS	NEG	POS	NEG	POS	NEG	NEG	POS	NEG	NEG	NEG	NEG	POS	NEG	NEG	NEG	POS	NEG	NEG
**UKD-1025105**	SCCmec VT+czrC (SO385)	CC398	POS	POS	NEG	POS	NEG	NEG	NEG	NEG	POS	NEG	NEG	NEG	NEG	AMB	NEG	NEG	NEG	POS	NEG	NEG
**UKD-11780898**	SCCmec VT+czrC (SO385)	CC398	POS	POS	NEG	POS	NEG	POS	NEG	NEG	POS	NEG	NEG	NEG	NEG	POS	NEG	NEG	NEG	POS	NEG	POS
**UKD-1377009**	SCCmec VT+czrC (SO385)	CC398	POS	POS	NEG	POS	NEG	POS	NEG	AMB	POS	NEG	NEG	NEG	NEG	POS	NEG	NEG	NEG	POS	NEG	AMB
**UKD-143406**	SCCmec VT+czrC (SO385)	CC398	POS	POS	NEG	POS	NEG	POS	NEG	AMB	POS	NEG	NEG	NEG	POS	POS	NEG	POS	NEG	POS	NEG	POS
**UKD-1400363**	SCCmec VT+czrC (SO385)	CC398	POS	POS	NEG	POS	NEG	POS	NEG	AMB	POS	NEG	NEG	NEG	NEG	POS	NEG	NEG	NEG	POS	NEG	NEG
**UKD-14571852**	SCCmec VT+czrC (SO385)	CC398	POS	POS	NEG	POS	NEG	POS	NEG	AMB	POS	NEG	NEG	NEG	NEG	POS	NEG	POS	NEG	POS	NEG	POS
**UKD-14582574**	SCCmec VT+czrC (SO385)	CC398	POS	POS	NEG	POS	NEG	POS	NEG	AMB	POS	NEG	NEG	NEG	NEG	POS	NEG	POS	NEG	POS	NEG	AMB
**UKD-152108**	SCCmec VT+czrC (SO385)	CC398	POS	POS	NEG	POS	NEG	POS	NEG	AMB	POS	NEG	NEG	NEG	NEG	POS	NEG	NEG	NEG	POS	NEG	NEG
**UKD-15573321**	SCCmec VT+czrC (SO385)	CC398	POS	POS	NEG	POS	NEG	POS	NEG	AMB	POS	NEG	NEG	NEG	NEG	POS	NEG	POS	NEG	POS	NEG	POS
**UKD-15584034**	SCCmec VT+czrC , ydhK-neg. variant	CC398	POS	POS	NEG	POS	NEG	POS	NEG	NEG	POS	NEG	NEG	NEG	NEG	POS	NEG	NEG	NEG	POS	NEG	NEG
**UKD-0932581**	SCCmecVT+fus+czrC	CC22	POS	NEG	NEG	POS	NEG	NEG	NEG	AMB	POS	NEG	NEG	NEG	NEG	POS	POS	NEG	NEG	POS	NEG	NEG
**UKD-1121994**	SCCmecVT+fus+czrC	CC22	POS	NEG	POS	POS	NEG	NEG	NEG	AMB	POS	NEG	NEG	POS	NEG	POS	AMB	AMB	NEG	POS	NEG	POS
**UKD-1272252**	SCCmecVT+fus+czrC	CC22	POS	NEG	POS	POS	NEG	AMB	NEG	AMB	POS	NEG	NEG	POS	NEG	POS	POS	NEG	NEG	POS	NEG	AMB
Prediction for MRSA18	*SCCmec VI+fus (MRSA18)*																					
**UKD-1103380**	SCCmec VI+fus (MRSA18)	CC7	POS	POS	NEG	NEG	AMB	NEG	NEG	POS	NEG	POS	POS	NEG	POS	NEG	POS	NEG	POS	NEG	POS	POS
Prediction for M10/0061, GenBank FR823292.1	*SCCmec XI (LGA251/M10-61)*	*CC130*																				
**Reference strain Dublin-DSH_M10 0061**	**SCCmec XI (LGA251/M10-61)**	**CC130**	**POS**	**POS**	**NEG**	**POS**	**NEG**	**POS**	**NEG**	**POS**	**NEG**	**POS**	**POS**	**NEG**	**POS**	**NEG**	**NEG**	**AMB**	**POS**	**NEG**	**NEG**	**NEG**
Prediction for LGA251, GenBank FR821779.1	*SCCmec XI (LGA251/M10-61)*	*ST425*																				
**UKD-14015835**	SCCmec XI (LGA251/M10-61)	CC130	POS	POS	NEG	POS	NEG	POS	NEG	POS	NEG	POS	POS	NEG	POS	NEG	NEG	AMB	POS	NEG	NEG	NEG
**UKD-16573252**	SCCmec XI (LGA251/M10-61)	CC130	POS	POS	NEG	POS	NEG	NEG	NEG	POS	NEG	POS	POS	NEG	POS	AMB	NEG	POS	POS	NEG	NEG	NEG
**UKD-0217002**	PseudoSCCmec, class B mec complex+Hg	CC8	POS	POS	NEG	NEG	NEG	POS	NEG	POS	NEG	POS	POS	NEG	POS	NEG	POS	AMB	POS	NEG	POS	POS
**UKD-0008510**	PseudoSCCmec, class B mec complex+Hg	CC8	POS						NEG	POS	NEG	POS	POS	NEG	POS	NEG	POS	NEG	POS	NEG	NEG	POS
**UKD-0110962**	PseudoSCCmec, class B mec complex+Hg	CC8	POS						NEG	POS	POS	POS	POS	NEG	POS	NEG	POS	AMB	POS	NEG	NEG	POS
**KHDN-10408593**	PseudoSCCmec, class B mec complex+Cu/Hg	CC8	POS	POS	NEG	AMB	NEG	POS	NEG	POS	AMB	POS	POS	NEG	POS	NEG	POS	AMB	POS	NEG	POS	POS
**UKD-0007201**	PseudoSCCmec, class B mec complex	CC5	POS	POS	NEG	POS	NEG	POS	NEG	POS	AMB	POS	POS	NEG	POS	AMB	POS	AMB	POS	NEG	NEG	NEG
**UKD-0033204**	PseudoSCCmec, class B mec complex	CC5	POS	POS	NEG	POS	NEG	POS	NEG	POS	AMB	POS	POS	NEG	POS	NEG	POS	AMB	POS	NEG	NEG	NEG
**UKD-0134329**	PseudoSCCmec, class B mec complex	CC5	POS	POS	NEG	POS	NEG	NEG	NEG	POS	NEG	POS	AMB	NEG	POS	NEG	AMB	NEG	POS	NEG	NEG	NEG
**UKD-0806946**	PseudoSCCmec class C+As/Cu	CC398	POS						NEG	NEG	POS	NEG	NEG	NEG	NEG	POS	NEG	NEG	NEG	POS	NEG	NEG
**UKD-0500610**	ACME2	CC8	POS						NEG	POS	NEG	POS	POS	NEG	POS	NEG	POS	AMB	POS	NEG	NEG	POS
**UKD-0610438**	ACME2	CC8	POS	POS	NEG	POS	NEG	POS	NEG	POS	AMB	POS	POS	NEG	POS	NEG	POS	POS	POS	NEG	POS	POS
**KHDN-10409344**	ACME2	CC8	POS	POS	NEG	POS	NEG	POS	NEG	POS	AMB	POS	POS	NEG	POS	NEG	POS	NEG	POS	NEG	POS	POS
**UKD-0322559**	ACME2+speG+czrC+ccrAB4	CC8	POS	POS	NEG	AMB	NEG	POS	NEG	POS	NEG	POS	POS	NEG	POS	NEG	POS	NEG	POS	NEG	POS	POS
**UKD-0501998**	ACME2+speG+czrC+ccrAB4	CC8	POS						NEG	POS	NEG	POS	POS	NEG	POS	NEG	POS	AMB	POS	NEG	NEG	POS
**UKD-0505213**	ACME2+speG+czrC+ccrAB4	CC8	POS						NEG	POS	AMB	POS	POS	NEG	POS	NEG	POS	AMB	POS	NEG	NEG	POS
Prediction for MSSA476, GenBank BX571857.1	*fus+tir+ccrAB1 (MSSA476)*	*CC1*	*POS*	*NEG*	*POS*	*NEG*	*POS*	*NEG*	*NEG*	*POS*	*AMB*	*POS*	*POS*	*NEG*	*POS*	*NEG*	*POS*	*NEG*	*POS*	*NEG*	*POS*	*POS*
**Reference strain Sanger 476**	**fus+tir+ccrAB1 (MSSA476)**	**CC1**	**POS**	**NEG**	**POS**	**NEG**	**POS**	**NEG**	**NEG**	**POS**	**NEG**	**POS**	**POS**	**NEG**	**POS**	**NEG**	**POS**	**NEG**	**POS**	**NEG**	**POS**	**POS**
**UKD-14023914**	fus+tir+ccrAB1 (MSSA476)	CC1	POS	NEG	POS	NEG	POS	NEG	NEG	POS	AMB	POS	POS	NEG	POS	NEG	POS	AMB	POS	NEG	POS	POS
**UKD-K00034**	kdp+ccrAB2	CC182	POS	NEG	POS	NEG	POS	NEG	NEG	POS	NEG	POS	POS	NEG	POS	NEG	NEG	POS	NEG	NEG	NEG	NEG
**UKD-15580369**	As+ccrAB4	CC22	POS	NEG	POS	POS	NEG	NEG	NEG	AMB	POS	NEG	NEG	POS	NEG	POS	POS	POS	NEG	POS	NEG	AMB
**UKD-15570100**	ACME3+ccrAB1 (Strain 21342)	CC80	POS	NEG	NEG	POS	NEG	POS	POS	POS	AMB	POS	POS	NEG	POS	AMB	POS	AMB	POS	NEG	NEG	POS
**UKD-1010678**	speG+czrC+ccrAB4	CC8	POS	POS	NEG	NEG	NEG	POS	NEG	POS	NEG	POS	POS	NEG	POS	NEG	POS	NEG	POS	NEG	POS	POS
**UKD-1008243**	speG+czrC+ccrAB4	CC8	POS	POS	NEG	POS	NEG	POS	NEG	POS	AMB	POS	POS	NEG	POS	NEG	POS	AMB	POS	NEG	POS	POS
**UKD-1009430**	speG+czrC+ccrAB4	CC8	POS	POS	NEG	NEG	NEG	POS	NEG	POS	NEG	POS	POS	NEG	POS	NEG	POS	NEG	POS	NEG	POS	POS
**UKD-12071828**	speG+czrC+ccrAB4	CC22	POS	NEG	POS	POS	NEG	NEG	NEG	AMB	POS	NEG	NEG	AMB	NEG	POS	POS	NEG	NEG	POS	NEG	NEG
**UKD-14011883**	speG+czrC+ccrAB4	CC22	POS	NEG	POS	POS	NEG	NEG	NEG	AMB	POS	NEG	NEG	POS	NEG	POS	POS	POS	NEG	POS	NEG	AMB
**UKD-15008293**	speG+czrC+ccrAB4	CC22	POS	NEG	POS	POS	NEG	NEG	NEG	AMB	POS	NEG	NEG	POS	NEG	POS	POS	NEG	NEG	POS	NEG	NEG
**UKD-1514877**	speG+czrC+ccrAB4	CC22	POS	NEG	POS	POS	NEG	NEG	NEG	POS	POS	NEG	NEG	POS	NEG	POS	POS	NEG	NEG	POS	NEG	NEG
**UKD-15580971**	speG+czrC+ccrAB4	CC22	POS	NEG	POS	POS	NEG	NEG	NEG	POS	AMB	AMB	NEG	POS	NEG	POS	POS	AMB	NEG	POS	NEG	AMB
**UKD-15582646**	speG+czrC+ccrAB4	CC22	POS	NEG	POS	POS	NEG	NEG	NEG	POS	POS	NEG	NEG	POS	NEG	POS	POS	NEG	NEG	POS	NEG	NEG

VIRULENCE : STAPHYLOCOCCAL SUPERANTIGEN/ENTEROTOXIN-LIKE GENES (SET/SSL)

Isolate ID	SCCmec type	Clonal complex	staphylococcal superantigen-like protein 7			staphylococcal superantigen-like protein 8		staphylococcal superantigen-like protein 9			staphylococcal superantigen-like protein 10			staphylococcal superantigene-like protein 11				staphylococcal exotoxin-like protein, second locus				
			ssl07/set 1	ssl07/set 1 (MRSA252)	ssl07/set 1 (AF188836)	ssl08/set 12_probe 1	ssl08/set 12_probe 2	ssl09/set 5_probe 1	ssl09/set 5_probe 2	ssl09/set 5 (MRSA252)	ssl10/set 4	ssl10 (RF122)	ssl10/set 4 (MRSA252)	ssl11/set 2 (COL)	ssl11+set 2(Mu50+N315)	ssl11+set 2(MW2+MSSA476)	ssl11/set 2 (MRSA252)	setB3	setB3 (MRSA252)	setB2	setB2 (MRSA252)	setB1
VIRTUAL HYBRIDISATION for COL, GenBank CP000046	*SCCmec I (COL)*	*CC8*	*NEG*	*NEG*	*NEG*	*NEG*	*NEG*	*POS*	*POS*	*NEG*	*POS*	*NEG*	*NEG*	*POS*	*NEG*	*NEG*	*NEG*	*POS*	*NEG*	*POS*	*NEG*	*POS*
**Reference strain COL**	**SCCmec I (COL)**	**CC8**	**NEG**	**NEG**	**NEG**	**NEG**	**NEG**	**POS**	**POS**	**NEG**	**POS**	**NEG**	**NEG**	**POS**	**NEG**	**NEG**	**NEG**	**POS**	**NEG**	**POS**	**NEG**	**POS**
**UKD-0007063**	SCCmec I (COL)	CC5	POS	AMB	AMB	POS	POS	POS	POS	NEG	POS	AMB	AMB	NEG	POS	NEG	NEG	POS	NEG	POS	NEG	POS
**UKD-0220759**	SCCmec I (COL)	CC5	POS	AMB	NEG	POS	POS	POS	POS	NEG	POS	NEG	NEG	NEG	POS	NEG	NEG	POS	NEG	POS	NEG	POS
**UKD-0308986**	SCCmec I (COL)	CC5	POS	AMB	AMB	POS	POS	POS	POS	NEG	POS	NEG	AMB	NEG	POS	NEG	NEG	POS	NEG	POS	NEG	POS
**UKD-0500009**	SCCmec I (COL)	CC5	POS	POS	NEG	POS	POS	POS	POS	NEG	POS	NEG	NEG	NEG	POS	NEG	NEG	POS	NEG	POS	NEG	POS
**UKD-0508483**	SCCmec I (COL)	CC5	POS	NEG	NEG	POS	POS	POS	NEG	NEG	POS	NEG	NEG	NEG	POS	NEG	NEG	POS	NEG	POS	NEG	POS
**UKD-0519001**	SCCmec I (COL)	CC5	AMB	POS	AMB	POS	POS	POS	NEG	NEG	POS	NEG	AMB	NEG	POS	NEG	NEG	POS	NEG	POS	NEG	POS
**UKD-0701876**	SCCmec I (COL)	CC5	POS	AMB	AMB	POS	POS	POS	POS	NEG	POS	AMB	NEG	NEG	POS	NEG	NEG	POS	NEG	POS	NEG	POS
**UKD-0708865**	SCCmec I (COL)	CC5	POS	AMB	NEG	POS	AMB	POS	NEG	NEG	POS	NEG	NEG	NEG	POS	NEG	NEG	POS	NEG	POS	NEG	POS
**UKD-1001782**	SCCmec I (COL)	CC5	AMB	POS	AMB	POS	POS	POS	POS	NEG	POS	AMB	AMB	NEG	POS	NEG	NEG	POS	NEG	POS	NEG	POS
Prediction for PSP1996, GenBank ANHU	*SCCmec I (PSP1996)*	*CC8*	*POS*	*NEG*	*NEG*	*POS*	*POS*	*POS*	*POS*	*NEG*	*POS*	*NEG*	*NEG*	*POS*	*NEG*	*NEG*	*NEG*	*POS*	*NEG*	*POS*	*NEG*	*POS*
**UKD-0261727**	SCCmec I (PSP1996)	CC8	POS	AMB	NEG	POS	POS	POS	POS	NEG	POS	NEG	NEG	POS	NEG	NEG	NEG	POS	NEG	POS	NEG	POS
VIRTUAL HYBRIDISATION for MRSA7 : VIRTUAL HYBRII	*SCCmec I+fus+tirS (Geraldine Clone)*	*CC5*	*POS*	*NEG*	*NEG*	*POS*	*POS*	*POS*	*POS*	*NEG*	*POS*	*NEG*	*NEG*	*NEG*	*POS*	*NEG*	*NEG*	*POS*	*NEG*	*POS*	*NEG*	*POS*
**UKD-1221930**	SCCmec I+fus+tirS (Geraldine Clone)	CC5	POS	AMB	NEG	POS	POS	POS	POS	NEG	POS	NEG	AMB	NEG	POS	NEG	NEG	POS	NEG	POS	NEG	POS
Prediction for N315, GenBank BA000018.3	*SCCmec II (N315)*	*CC5*	*POS*	*NEG*	*NEG*	*POS*	*POS*	*POS*	*POS*	*NEG*	*POS*	*NEG*	*NEG*	*NEG*	*POS*	*NEG*	*NEG*	*POS*	*NEG*	*POS*	*NEG*	*POS*
**Reference strain N315**	**SCCmec II (N315)**	**CC5**	**POS**	**NEG**	**NEG**	**POS**	**POS**	**POS**	**POS**	**NEG**	**POS**	**NEG**	**NEG**	**NEG**	**POS**	**NEG**	**NEG**	**POS**	**NEG**	**POS**	**NEG**	**POS**
Prediction for MRSA252, GenBank BX571856.1	*SCCmec II (N315)*	*CC30*	*NEG*	*POS*	*NEG*	*NEG*	*NEG*	*NEG*	*NEG*	*POS*	*NEG*	*NEG*	*POS*	*NEG*	*NEG*	*NEG*	*POS*	*NEG*	*POS*	*NEG*	*POS*	*AMB*
**Reference strain Sanger 252**	**SCCmec II (N315)**	**CC30**	**NEG**	**POS**	**NEG**	**NEG**	**NEG**	**NEG**	**NEG**	**POS**	**NEG**	**NEG**	**POS**	**NEG**	**NEG**	**NEG**	**POS**	**NEG**	**POS**	**NEG**	**POS**	**AMB**
**UKD-0230792**	SCCmec II (N315)	CC30	NEG	POS	AMB	NEG	NEG	NEG	NEG	POS	AMB	NEG	POS	NEG	NEG	NEG	POS	NEG	POS	NEG	POS	POS
Prediction for Mu50, GenBank BA000017.4	*SCCmec II (N315)*	*CC5*	*POS*	*NEG*	*NEG*	*POS*	*POS*	*POS*	*POS*	*NEG*	*POS*	*NEG*	*NEG*	*NEG*	*POS*	*NEG*	*NEG*	*POS*	*NEG*	*POS*	*NEG*	*POS*
**Reference strain MU50**	**SCCmec II (N315)**	**CC5**	**POS**	**NEG**	**NEG**	**POS**	**POS**	**POS**	**POS**	**NEG**	**POS**	**NEG**	**NEG**	**NEG**	**POS**	**NEG**	**NEG**	**POS**	**NEG**	**POS**	**NEG**	**POS**
Prediction for JH1, GenBank CP000736.1	*SCCmec II (JH1/JH9)*	*CC5*	*POS*	*NEG*	*NEG*	*POS*	*POS*	*POS*	*POS*	*NEG*	*POS*	*NEG*	*NEG*	*NEG*	*POS*	*NEG*	*NEG*	*POS*	*NEG*	*POS*	*NEG*	*POS*
Prediction for JH9, GenBank CP000703.1	*SCCmec II (JH1/JH9)*	*CC5*	*POS*	*NEG*	*NEG*	*POS*	*POS*	*POS*	*POS*	*NEG*	*POS*	*NEG*	*NEG*	*NEG*	*POS*	*NEG*	*NEG*	*POS*	*NEG*	*POS*	*NEG*	*POS*
**Reference strain Dublin-DSH_AR07.4_0237**	**SCCmec II (JH1/JH9)**		**POS**	**AMB**	**AMB**	**POS**	**POS**	**POS**	**POS**	**NEG**	**POS**	**NEG**	**AMB**	**NEG**	**POS**	**NEG**	**NEG**	**POS**	**NEG**	**POS**	**NEG**	**POS**
**Reference strain NARSA_382 / USA100**	**SCCmec II (JH1/JH9)**	**CC5**	**POS**	**AMB**	**AMB**	**POS**	**POS**	**POS**	**POS**	**NEG**	**POS**	**NEG**	**AMB**	**NEG**	**POS**	**NEG**	**NEG**	**POS**	**NEG**	**POS**	**NEG**	**POS**
**UKD-0046921**	SCCmec II (JH1/JH9)	CC5	POS	AMB	AMB	POS	POS	POS	POS	NEG	POS	AMB	AMB	NEG	POS	NEG	NEG	POS	NEG	POS	NEG	POS
**UKD-0333415**	SCCmec II (JH1/JH9)	CC5	POS	AMB	NEG	POS	POS	POS	POS	NEG	POS	NEG	AMB	NEG	POS	NEG	NEG	POS	NEG	POS	NEG	POS
**UKD-0341645**	SCCmec II (JH1/JH9)	CC5	POS	AMB	NEG	POS	POS	POS	POS	NEG	POS	NEG	AMB	NEG	POS	NEG	NEG	POS	NEG	POS	NEG	POS
**UKD-0504626**	SCCmec II (JH1/JH9)	CC5	POS	NEG	NEG	POS	POS	NEG	NEG	NEG	POS	NEG	NEG	NEG	POS	NEG	NEG	POS	NEG	POS	NEG	POS
**UKD-0507581**	SCCmec II (JH1/JH9)	CC5	POS	NEG	NEG	POS	POS	POS	POS	NEG	POS	NEG	NEG	NEG	POS	NEG	NEG	POS	NEG	POS	NEG	POS
**UKD-0507903**	SCCmec II (JH1/JH9)	CC5	POS	AMB	NEG	POS	POS	POS	POS	NEG	POS	NEG	NEG	NEG	POS	NEG	NEG	POS	NEG	POS	NEG	POS
**UKD-0700817**	SCCmec II (JH1/JH9)	CC5	POS	AMB	AMB	POS	POS	POS	NEG	NEG	POS	NEG	NEG	NEG	POS	NEG	NEG	POS	NEG	POS	NEG	POS
**UKD-1014561**	SCCmec II (JH1/JH9)	CC5	POS	AMB	NEG	POS	POS	POS	POS	NEG	POS	NEG	NEG	NEG	POS	NEG	NEG	POS	NEG	POS	NEG	POS
**UKD-1037507**	SCCmec II (JH1/JH9)	CC5	AMB	POS	AMB	POS	POS	POS	POS	NEG	POS	AMB	AMB	NEG	POS	NEG	NEG	POS	NEG	POS	NEG	POS
**UKD-1038321**	SCCmec II (JH1/JH9)	CC5	POS	AMB	NEG	POS	POS	POS	POS	NEG	POS	NEG	NEG	NEG	POS	NEG	NEG	POS	NEG	POS	NEG	POS
**UKD-1111608**	SCCmec II (JH1/JH9)	CC5	POS	AMB	NEG	POS	POS	POS	POS	NEG	POS	NEG	AMB	NEG	POS	NEG	NEG	POS	NEG	POS	NEG	POS
**UKD-12753135**	SCCmec II (JH1/JH9)	CC5	AMB	POS	AMB	POS	POS	POS	POS	NEG	POS	AMB	AMB	NEG	POS	NEG	NEG	POS	NEG	POS	NEG	POS
**UKD-1312816**	SCCmec II (JH1/JH9)	CC5	POS	AMB	NEG	POS	POS	POS	POS	NEG	POS	NEG	AMB	NEG	POS	NEG	NEG	POS	NEG	POS	NEG	POS
**UKD-1401623**	SCCmec II (JH1/JH9)	CC5	POS	AMB	AMB	POS	POS	POS	POS	NEG	POS	AMB	AMB	NEG	POS	NEG	NEG	POS	NEG	POS	NEG	POS
**UKD-15571943**	SCCmec II+czrC+ccrA/B-4	CC5	AMB	POS	AMB	POS	POS	POS	POS	NEG	POS	AMB	AMB	NEG	POS	NEG	NEG	POS	NEG	POS	NEG	POS
**UKD-15576888**	SCCmec II+czrC+ccrA/B-4	CC5	POS	AMB	AMB	POS	POS	POS	POS	NEG	POS	AMB	AMB	NEG	POS	NEG	NEG	POS	NEG	POS	NEG	POS
**UKD-14576461**	SCCmec II+speG+czrC+ccrA/B-4	CC5	POS	AMB	AMB	POS	POS	POS	POS	NEG	POS	AMB	AMB	NEG	POS	NEG	NEG	POS	NEG	POS	NEG	POS
**UKD-15581649**	SCCmec II+speG+czrC+ccrA/B-4	CC5	POS	AMB	AMB	POS	POS	POS	POS	NEG	POS	AMB	AMB	NEG	POS	NEG	NEG	POS	NEG	POS	NEG	POS
Prediction for CIGC345D, GenBank AHVO	*SCCmec II A/B/D*	*CC8*	*POS*	*NEG*	*NEG*	*POS*	*POS*	*POS*	*POS*	*NEG*	*POS*	*NEG*	*NEG*	*POS*	*NEG*	*NEG*	*NEG*	*POS*	*NEG*	*POS*	*NEG*	*POS*
**UKD-12K00171**	SCCmec II A/B/D	CC8	AMB	POS	AMB	POS	POS	POS	POS	NEG	POS	NEG	AMB	POS	NEG	NEG	NEG	POS	NEG	POS	NEG	POS
Prediction for JKD6008, GenBank CP002120.1	*SCC [mec III+Cd] (JKD6008)*	*CC8 (ST239)*	*POS*	*NEG*	*NEG*	*POS*	*POS*	*POS*	*POS*	*NEG*	*POS*	*NEG*	*NEG*	*POS*	*NEG*	*NEG*	*NEG*	*POS*	*NEG*	*POS*	*NEG*	*POS*
Prediction for JKD6009, GenBank ABSA	*SCC [mec III+Cd] (JKD6008)*	*CC8 (ST239)*	*POS*	*NEG*	*NEG*	*POS*	*POS*	*POS*	*POS*	*NEG*	*POS*	*NEG*	*NEG*	*POS*	*NEG*	*NEG*	*NEG*	*POS*	*NEG*	*POS*	*NEG*	*POS*
**Reference strain ATCC_33592**	**SCC [mec III+Cd] (ATCC33592)**	**CC8 (ST239)**	**POS**	**POS**	**AMB**	**POS**	**POS**	**POS**	**POS**	**NEG**	**POS**	**NEG**	**AMB**	**POS**	**NEG**	**NEG**	**NEG**	**POS**	**NEG**	**POS**	**NEG**	**POS**
Prediction for PPUKM-775-2009, GenBank AMRE	*SCC [mec III+Cd+ccrC] (PPUKM-775-2009)*	*CC8 (ST239)*	*POS*	*NEG*	*NEG*	*POS*	*POS*	*POS*	*POS*	*NEG*	*POS*	*NEG*	*NEG*	*POS*	*NEG*	*NEG*	*NEG*	*POS*	*NEG*	*POS*	*NEG*	*POS*
Prediction for T0131, GenBank CP002643.1	*SCC [mec III+Cd+ccrC] (T0131)*	*CC8 (ST239)*	*POS*	*NEG*	*NEG*	*POS*	*POS*	*POS*	*POS*	*NEG*	*POS*	*NEG*	*NEG*	*POS*	*NEG*	*NEG*	*NEG*	*POS*	*NEG*	*POS*	*NEG*	*POS*
Prediction for XN108, GenBank CP007447.1	*SCC [mec III+Cd+ccrC] (XN108)*	*CC8 (ST239)*	*POS*	*NEG*	*NEG*	*POS*	*POS*	*POS*	*POS*	*NEG*	*POS*	*NEG*	*NEG*	*POS*	*NEG*	*NEG*	*NEG*	*POS*	*NEG*	*POS*	*NEG*	*POS*
Prediction for CN79, GenBank ANCJ	*SCC [mec III+Cd+ccrC] (CN79)*	*CC8 (ST239)*	*POS*	*NEG*	*NEG*	*POS*	*POS*	*POS*	*POS*	*NEG*	*POS*	*NEG*	*NEG*	*POS*	*NEG*	*NEG*	*NEG*	*POS*	*NEG*	*POS*	*NEG*	*POS*
Prediction for 16K, GenBank BABZ	*SCC [mec III+Cd+ccrC] (CN79)*	*CC8 (ST239)*	*POS*	*NEG*	*NEG*	*POS*	*POS*	*POS*	*POS*	*NEG*	*POS*	*NEG*	*NEG*	*POS*	*NEG*	*NEG*	*NEG*	*POS*	*NEG*	*POS*	*NEG*	*POS*
**UKD-0835987**	SCC [mec III+Cd+ccrC] (CN79)	CC8 (ST239)	POS	AMB	AMB	POS	POS	POS	AMB	NEG	POS	NEG	NEG	POS	NEG	NEG	NEG	POS	NEG	POS	NEG	POS
**UKD-16581769**	SCCmec III+Cd+ccrC	CC8 (ST239)	POS	NEG	NEG	POS	POS	POS	POS	NEG	POS	NEG	NEG	POS	NEG	NEG	NEG	POS	NEG	POS	NEG	POS
Prediction for Bmb9393, GenBank CP005288.1	*SCCmec III+Cd/Hg+ccrC (Bmb9393)*	*CC8 (ST239)*	*POS*	*NEG*	*NEG*	*POS*	*POS*	*POS*	*POS*	*NEG*	*POS*	*NEG*	*NEG*	*POS*	*NEG*	*NEG*	*NEG*	*POS*	*NEG*	*POS*	*NEG*	*POS*
**UKD-15574263**	SCCmec III+Cd/Hg+ccrC (Bmb9393)	CC8 (ST239)	POS	AMB	AMB	POS	POS	POS	POS	NEG	POS	AMB	AMB	POS	NEG	NEG	NEG	POS	NEG	POS	NEG	POS
Prediction for SK1585, GenBank AYLT	*SCCmec III+ccrC+Cd/Hg (SK1585)*	*CC8 (ST239)*	*POS*	*NEG*	*NEG*	*POS*	*POS*	*POS*	*POS*	*NEG*	*POS*	*NEG*	*NEG*	*POS*	*NEG*	*NEG*	*NEG*	*NEG*	*POS*	*NEG*	*NEG*	*NEG*
**UKD-105861**	SCCmec III+ccrC+Cd/Hg (SK1585)	CC8 (ST239)	POS	AMB	NEG	POS	POS	POS	POS	NEG	POS	NEG	NEG	POS	NEG	NEG	NEG	POS	NEG	POS	NEG	POS
**UKD-134572**	SCCmec III+ccrC+Cd/Hg (SK1585)	CC8 (ST239)	POS	AMB	NEG	POS	POS	POS	NEG	NEG	POS	NEG	NEG	POS	NEG	NEG	NEG	POS	NEG	POS	NEG	POS
**UKD-139143**	SCCmec III+ccrC+Cd/Hg (SK1585)	CC8 (ST239)	POS	AMB	NEG	POS	POS	POS	POS	NEG	POS	NEG	NEG	POS	NEG	NEG	NEG	POS	NEG	POS	NEG	POS
Prediction for TW20, GenBank FN433596.1	*SCC [mec III+Cd/Hg+ccrC] (TW20)*	*CC8 (ST239)*	*POS*	*NEG*	*NEG*	*POS*	*POS*	*POS*	*POS*	*NEG*	*POS*	*NEG*	*NEG*	*POS*	*NEG*	*NEG*	*NEG*	*POS*	*NEG*	*POS*	*NEG*	*POS*
Prediction for ATCC BAA-39, GenBank AEEK	*SCC [mec III+Cd/Hg+ccrC] (TW20)*	*CC8 (ST239)*	*POS*	*NEG*	*NEG*	*POS*	*POS*	*POS*	*POS*	*NEG*	*POS*	*NEG*	*NEG*	*POS*	*NEG*	*NEG*	*NEG*	*POS*	*NEG*	*POS*	*NEG*	*POS*

Isolate ID	SCCmec type	Clonal complex	VIRULENCE : STAPHYLOCOCCAL SUPERANTIGEN/ENTEROTOXIN-LIKE GENES (SET/SSL)																			
			staphylococcal superantigen-like protein 7			staphylococcal superantigen-like protein 8		staphylococcal superantigen-like protein 9			staphylococcal superantigen-like protein 10			staphylococcal superantigene-like protein 11				staphylococcal exotoxin-like protein, second locus				
			ssl07/set1	ssl07/set1 (MRSA252)	ssl07/set1 (AF188836)	ssl08/set12_probe1	ssl08/set12_probe2	ssl09/set5_probe1	ssl09/set5_probe2	ssl09/set5 (MRSA252)	ssl10/set4	ssl10 (RF122)	ssl10/set4 (MRSA252)	ssl11/set2 (COL)	ssl11+set2(Mu50+N315)	ssl11+set2(MW2+MSSA476)	ssl11/set2 (MRSA252)	setB3	setB3 (MRSA252)	setB2	setB2 (MRSA252)	setB1
Prediction for MW2, GenBank BA000033.2	*SCCmec IVa (MW2)*	*CC1*	*POS*	*NEG*	*NEG*	*POS*	*POS*	*POS*	*POS*	*NEG*	*POS*	*NEG*	*NEG*	*NEG*	*NEG*	*POS*	*NEG*	*POS*	*NEG*	*POS*	*NEG*	*POS*
**Reference strain MW2**	**SCCmec IVa (MW2)**	**CC1**	**POS**	**NEG**	**NEG**	**POS**	**POS**	**POS**	**POS**	**NEG**	**POS**	**NEG**	**NEG**	**NEG**	**NEG**	**POS**	**NEG**	**POS**	**NEG**	**POS**	**NEG**	**POS**
**Reference strain CA05 (JCSC1968)**	**SCCmec IVa (MW2)**	**CC45**	**NEG**	**NEG**	**NEG**	**NEG**	**NEG**	**NEG**	**NEG**	**POS**	**NEG**	**NEG**	**POS**	**NEG**	**NEG**	**NEG**	**NEG**	**NEG**	**POS**	**NEG**	**POS**	**NEG**
**UKD-0127106**	SCCmec IVa (MW2)	CC1	POS	AMB	AMB	POS	POS	POS	AMB	NEG	POS	NEG	NEG	NEG	NEG	POS	NEG	POS	NEG	POS	NEG	POS
**UKD-0701446**	SCCmec IVa (MW2)	CC1	POS	AMB	AMB	POS	POS	POS	POS	NEG	POS	AMB	AMB	NEG	NEG	POS	NEG	POS	NEG	POS	NEG	POS
**UKD-0947486**	SCCmec IVa (MW2)	CC1	POS	AMB	NEG	POS	POS	POS	POS	NEG	POS	NEG	AMB	NEG	NEG	POS	NEG	POS	NEG	POS	NEG	POS
**UKD-1031910**	SCCmec IVa (MW2)	CC1	AMB	POS	NEG	POS	POS	POS	POS	NEG	POS	NEG	AMB	NEG	NEG	POS	NEG	POS	NEG	POS	NEG	POS
**UKD-14571328**	SCCmec IVa (MW2)	CC1	AMB	POS	AMB	POS	POS	POS	POS	NEG	POS	NEG	AMB	NEG	NEG	POS	NEG	POS	NEG	POS	NEG	POS
**UKD-03003012**	SCCmec IVa (MW2)	CC5	POS	POS	NEG	POS	POS	POS	POS	NEG	POS	NEG	NEG	AMB	POS	NEG	NEG	POS	NEG	POS	NEG	POS
**UKD-15582582**	SCCmec IVa (MW2)	CC6	POS	NEG	NEG	POS	POS	POS	POS	NEG	POS	AMB	AMB	NEG	POS	NEG	NEG	POS	NEG	POS	NEG	POS
**UKD-16574230**	SCCmec IVa (MW2)	CC6	POS	AMB	AMB	POS	POS	POS	POS	NEG	POS	AMB	AMB	NEG	POS	NEG	NEG	POS	NEG	POS	NEG	POS
**UKD-16582560**	SCCmec IVa (MW2)	CC6	POS	NEG	NEG	POS	POS	POS	POS	NEG	AMB	POS	NEG	NEG	POS	NEG	NEG	POS	NEG	POS	NEG	POS
**UKD-0802855**	SCCmec IVa (MW2)	CC7	POS	AMB	NEG	POS	POS	POS	POS	NEG	POS	NEG	NEG	NEG	NEG	NEG	NEG	POS	NEG	POS	NEG	POS
**KHDN-10405258**	SCCmec IVa (MW2)	CC8	POS	AMB	NEG	POS	POS	POS	POS	NEG	POS	NEG	AMB	POS	NEG	NEG	NEG	POS	NEG	POS	NEG	POS
**UKD-15573497**	SCCmec IVa (MW2)	CC22	AMB	POS	AMB	NEG	NEG	POS	POS	NEG	POS	NEG	AMB	NEG	NEG	NEG	NEG	NEG	NEG	NEG	NEG	POS
**UKD-0634729**	SCCmec IVa (MW2)	CC30	AMB	POS	AMB	NEG	NEG	NEG	NEG	POS	NEG	NEG	POS	NEG	NEG	NEG	POS	NEG	POS	NEG	POS	POS
**UKD-1119771**	SCCmec IVa (MW2)	CC30	AMB	POS	AMB	NEG	NEG	NEG	NEG	POS	AMB	NEG	POS	NEG	NEG	NEG	POS	NEG	POS	NEG	POS	POS
**KHDN-10411458**	SCCmec IVa (MW2)	CC30	NEG	POS	AMB	NEG	NEG	NEG	NEG	POS	AMB	NEG	POS	NEG	NEG	NEG	POS	NEG	POS	NEG	POS	POS
**KHDN-398609**	SCCmec IVa (MW2)	CC30	AMB	POS	AMB	NEG	NEG	NEG	NEG	POS	POS	NEG	AMB	NEG	NEG	NEG	POS	NEG	POS	NEG	POS	POS
**KHDN-01081998**	SCCmec IVa (MW2)	CC30	AMB	POS	AMB	NEG	NEG	NEG	NEG	POS	AMB	NEG	POS	NEG	NEG	NEG	POS	NEG	POS	NEG	POS	POS
**KHDN-10399223**	SCCmec IVa (MW2)	CC30	NEG	POS	AMB	NEG	NEG	NEG	NEG	POS	POS	NEG	AMB	NEG	NEG	NEG	POS	NEG	POS	NEG	POS	POS
**KHDN-W261158**	SCCmec IVa (MW2)	CC30	NEG	POS	NEG	NEG	NEG	NEG	NEG	POS	AMB	NEG	POS	NEG	NEG	NEG	POS	NEG	POS	NEG	POS	POS
**UKD-0002046**	SCCmec IVa (MW2)	CC45 [agr I]	NEG	NEG	NEG	NEG	NEG	NEG	NEG	POS	POS	NEG	AMB	NEG	NEG	NEG	NEG	NEG	POS	NEG	POS	NEG
**UKD-0029618**	SCCmec IVa (MW2)	CC45 [agr I]	NEG	NEG	NEG	NEG	NEG	NEG	NEG	POS	AMB	NEG	POS	NEG	NEG	NEG	NEG	NEG	POS	NEG	NEG	NEG
**UKD-0043085**	SCCmec IVa (MW2)	CC45 [agr I]	NEG	NEG	NEG	NEG	NEG	NEG	NEG	POS	AMB	NEG	POS	NEG	NEG	NEG	NEG	NEG	POS	NEG	NEG	NEG
**UKD-0047053**	SCCmec IVa (MW2)	CC45 [agr I]	NEG	NEG	NEG	NEG	NEG	NEG	NEG	POS	NEG	NEG	POS	NEG	NEG	NEG	NEG	NEG	POS	NEG	NEG	POS
**UKD-0047429**	SCCmec IVa (MW2)	CC45 [agr I]	NEG	NEG	NEG	NEG	NEG	NEG	NEG	POS	AMB	NEG	POS	NEG	NEG	NEG	NEG	NEG	POS	NEG	NEG	NEG
**UKD-0112465**	SCCmec IVa (MW2)	CC45 [agr I]	NEG	NEG	NEG	NEG	NEG	NEG	NEG	POS	AMB	NEG	POS	NEG	NEG	NEG	NEG	NEG	POS	NEG	NEG	NEG
**UKD-0103483**	SCCmec IVa (MW2)	CC45 [agr I]	NEG	NEG	NEG	NEG	NEG	NEG	NEG	POS	AMB	NEG	POS	NEG	NEG	NEG	NEG	NEG	POS	NEG	NEG	NEG
**UKD-0305014**	SCCmec IVa (MW2)	CC45 [agr I]	NEG	NEG	NEG	NEG	NEG	NEG	NEG	POS	AMB	NEG	POS	NEG	NEG	NEG	NEG	NEG	POS	NEG	NEG	NEG
**UKD-0401119**	SCCmec IVa (MW2)	CC45 [agr I]	NEG	NEG	NEG	NEG	NEG	NEG	NEG	POS	POS	NEG	AMB	NEG	NEG	NEG	NEG	NEG	POS	NEG	NEG	NEG
**UKD-0537969**	SCCmec IVa (MW2)	CC45 [agr I]	NEG	NEG	NEG	NEG	NEG	NEG	NEG	POS	AMB	NEG	POS	NEG	NEG	NEG	NEG	NEG	POS	NEG	NEG	NEG
**UKD-0703373**	SCCmec IVa (MW2)	CC45 [agr I]	NEG	NEG	NEG	NEG	NEG	NEG	NEG	POS	NEG	NEG	POS	NEG	NEG	NEG	NEG	NEG	POS	NEG	NEG	NEG
**UKD-0813653**	SCCmec IVa (MW2)	CC45 [agr I]	NEG	NEG	NEG	NEG	NEG	NEG	NEG	POS	AMB	NEG	POS	NEG	NEG	NEG	NEG	NEG	POS	NEG	NEG	NEG
**UKD-0839175**	SCCmec IVa (MW2)	CC45 [agr I]	NEG	NEG	NEG	NEG	NEG	NEG	NEG	POS	AMB	NEG	POS	NEG	NEG	NEG	NEG	NEG	POS	NEG	AMB	AMB
**UKD-1108422**	SCCmec IVa (MW2)	CC45 [agr I]	NEG	NEG	NEG	NEG	NEG	NEG	NEG	POS	AMB	NEG	POS	NEG	NEG	NEG	NEG	NEG	POS	NEG	POS	POS
**UKD-1380645**	SCCmec IVa (MW2)	CC45 [agr I]	NEG	NEG	POS	NEG	NEG	NEG	NEG	POS	POS	NEG	POS	NEG	NEG	NEG	NEG	NEG	POS	NEG	POS	NEG
**UKD-148016**	SCCmec IVa (MW2)	CC45 [agr I]	AMB	AMB	POS	NEG	NEG	NEG	NEG	POS	AMB	AMB	POS	NEG	NEG	NEG	NEG	NEG	POS	NEG	POS	AMB
**UKD-16571773**	SCCmec IVa (MW2)	CC45 [agr I]	NEG	AMB	POS	NEG	NEG	NEG	NEG	POS	AMB	NEG	POS	NEG	NEG	NEG	NEG	NEG	POS	NEG	POS	POS
**KHDN-10407738**	SCCmec IVa (MW2)	CC45 [ST617]	NEG	AMB	POS	NEG	NEG	NEG	NEG	POS	AMB	NEG	POS	NEG	NEG	NEG	NEG	NEG	POS	NEG	POS	NEG
**UKD-0853670**	SCCmec IVa (MW2)	CC88	POS	AMB	AMB	POS	POS	POS	POS	NEG	POS	NEG	AMB	NEG	NEG	NEG	AMB	POS	NEG	POS	NEG	POS
**UKD-1417352**	SCCmec IVa (MW2)	CC88	POS	POS	AMB	POS	POS	POS	POS	NEG	POS	NEG	AMB	NEG	NEG	NEG	POS	POS	NEG	POS	NEG	POS
**UKD-15571283**	SCCmec IVa (MW2)	CC88	AMB	POS	AMB	POS	POS	POS	POS	NEG	POS	AMB	AMB	NEG	NEG	NEG	POS	POS	NEG	POS	NEG	POS
**KHDN-10406719**	SCCmec IVa (MW2)	ST93	NEG	NEG	NEG	POS	POS	NEG	NEG	NEG	AMB	NEG	POS	NEG	NEG	NEG	NEG	NEG	POS	NEG	AMB	NEG
**KHDN-0060294**	SCCmec IVa (MW2)	ST93	NEG	NEG	NEG	POS	POS	NEG	NEG	NEG	NEG	NEG	POS	NEG	NEG	NEG	NEG	NEG	POS	NEG	NEG	NEG
**UKD-0734190**	SCCmec IVa (MW2)	CC97	POS	AMB	NEG	POS	POS	POS	POS	NEG	POS	NEG	NEG	NEG	NEG	NEG	NEG	POS	NEG	POS	NEG	POS
**UKD-13006983**	SCCmec IVa (MW2)	CC97	AMB	POS	AMB	POS	POS	POS	POS	NEG	POS	AMB	AMB	NEG	NEG	NEG	NEG	POS	NEG	POS	NEG	POS
Prediction for H131520133	*SCCmec IVa (H131520133)*	*CC152*	*NEG*	*NEG*	*POS*	*NEG*	*NEG*	*NEG*	*NEG*	*NEG*	*NEG*	*NEG*	*NEG*	*NEG*	*NEG*	*NEG*	*NEG*	*POS*	*NEG*	*POS*	*NEG*	*POS*
**KHDN-325892**	SCCmec IVa (H131520133)	CC30	AMB	POS	AMB	NEG	NEG	NEG	NEG	POS	AMB	NEG	POS	NEG	NEG	NEG	POS	NEG	POS	NEG	POS	POS
**UKD-15580207**	SCCmec IVa (H131520133)	CC88	AMB	POS	AMB	POS	POS	POS	POS	NEG	POS	AMB	AMB	NEG	NEG	NEG	POS	POS	NEG	POS	NEG	POS
**UKD-0521159**	SCCmec Iva	CC8	POS	AMB	NEG	POS	POS	POS	NEG	NEG	POS	NEG	NEG	POS	NEG	NEG	NEG	POS	NEG	POS	NEG	POS
Prediction for CMFT503 : HF569113.1	*SCCmec IVa (CMFT503)*																					
Prediction for 21343, GenBank AHKV	*SCCmec IVa (CMFT503)*	*CC88*	*POS*	*NEG*	*NEG*	*POS*	*POS*	*POS*	*POS*	*NEG*	*POS*	*NEG*	*NEG*	*NEG*	*NEG*	*NEG*	*AMB*	*POS*	*NEG*	*POS*	*NEG*	*POS*
**UKD-1420740**	SCCmec IVa (CMFT503)	CC22	AMB	POS	AMB	NEG	NEG	POS	POS	NEG	POS	NEG	AMB	NEG	NEG	NEG	NEG	NEG	NEG	POS	NEG	POS
**UKD-15575124**	SCCmec IVa (CMFT503)	CC22	AMB	AMB	POS	NEG	NEG	POS	POS	NEG	AMB	NEG	POS	NEG	NEG	NEG	NEG	NEG	NEG	NEG	NEG	POS
**UKD-16584947**	SCCmec IVa (CMFT503)	CC22	AMB	AMB	POS	NEG	NEG	POS	POS	NEG	NEG	NEG	NEG	NEG	NEG	NEG	NEG	NEG	NEG	NEG	NEG	NEG
**UKD-16575077**	SCCmec IVa (CMFT503)	CC88	POS	AMB	NEG	POS	POS	POS	POS	NEG	POS	NEG	AMB	NEG	NEG	NEG	NEG	POS	NEG	POS	NEG	POS
Prediction for TCH1516, GenBank CP000730.1	*SCCmec IVa+ACME1+Cu (USA300)*	*CC8*	*POS*	*NEG*	*NEG*	*POS*	*POS*	*POS*	*POS*	*NEG*	*POS*	*NEG*	*NEG*	*POS*	*NEG*	*NEG*	*NEG*	*POS*	*NEG*	*POS*	*NEG*	*POS*
Reference strain USA300-TCH1516	SCCmec IVa+ACME1+Cu (USA300)	CC8	POS	AMB	NEG	POS	POS	POS	POS	NEG	POS	NEG	AMB	POS	NEG	NEG	NEG	POS	NEG	POS	NEG	POS
Prediction for FPR3757, GenBank CP000255.1	*SCCmec IVa+ACME1+Cu (USA300)*	*CC8*	*POS*	*NEG*	*NEG*	*POS*	*POS*	*POS*	*POS*	*NEG*	*POS*	*NEG*	*NEG*	*POS*	*NEG*	*NEG*	*NEG*	*POS*	*NEG*	*POS*	*NEG*	*POS*
**UKD-0516386**	SCCmec IVa+ACME1+Cu (USA300)	CC8	POS	AMB	NEG	POS	POS	POS	NEG	NEG	POS	NEG	NEG	POS	AMB	NEG	NEG	POS	NEG	POS	NEG	POS
**UKD-0815773**	SCCmec IVa+ACME1+Cu (USA300)	CC8	POS	AMB	NEG	POS	POS	POS	POS	NEG	POS	NEG	NEG	POS	NEG	NEG	NEG	POS	NEG	POS	NEG	POS
**UKD-1414654**	SCCmec IVa+ACME1+Cu (USA300)	CC8	POS	AMB	AMB	POS	POS	POS	POS	NEG	POS	AMB	AMB	POS	NEG	NEG	NEG	POS	NEG	POS	NEG	POS
**UKD-0408036**	SCCmec IVa+ACME2	CC45 [agr I]	NEG	NEG	NEG	NEG	NEG	NEG	NEG	POS	AMB	AMB	POS	NEG	NEG	NEG	NEG	NEG	POS	NEG	AMB	AMB
**UKD-0044035**	SCCmec IVa+ccrC (Hannover)	CC8	POS	AMB	NEG	POS	POS	POS	NEG	NEG	POS	NEG	NEG	POS	NEG	NEG	NEG	POS	NEG	POS	NEG	POS
**UKD-0136287**	SCCmec IVa+ccrC (Hannover)	CC8	POS	AMB	NEG	POS	POS	POS	POS	NEG	POS	NEG	AMB	POS	NEG	NEG	NEG	POS	NEG	POS	NEG	POS

Isolate ID	SCCmec type	Clonal complex	VIRULENCE : STAPHYLOCOCCAL SUPERANTIGEN/ENTEROTOXIN-LIKE GENES (SET/SSL)																			
			staphylococcal superantigen-like protein 7			staphylococcal superantigen-like protein 8		staphylococcal superantigen-like protein 9			staphylococcal superantigen-like protein 10			staphylococcal superantigene-like protein 11				staphylococcal exotoxin-like protein, second locus				
			ssl07/set 1	ssl07/set 1 (MRSA252)	ssl07/set 1 (AF188836)	ssl08/set 12_probe 1	ssl08/set 12_probe 2	ssl09/set 5_probe 1	ssl09/set 5_probe 2	ssl09/set 5 (MRSA252)	ssl10/set 4	ssl10 (RF122)	ssl10/set 4 (MRSA252)	ssl11/set 2 (COL)	ssl11+set 2(Mu50+ N315)	ssl11+set 2(MW2+ MSSA476)	ssl11/set 2 (MRSA252)	setB3	setB3 (MRSA252)	setB2	setB2 (MRSA252)	setB1
Prediction for JCSC1978, GenBank AB063173.1	***SCCmec IVb/d/i (JCSC1978/6668/4469)***																					
**Reference strain JCSC1978 (8/6-3P)**	**SCCmec IVb/d/i (JCSC1978/6668/4469)**	**CC8**	**POS**	**AMB**	**NEG**	**POS**	**POS**	**POS**	**POS**	**NEG**	**POS**	**AMB**	**AMB**	**POS**	**NEG**	**NEG**	**NEG**	**POS**	**NEG**	**POS**	**NEG**	**POS**
Prediction for JCSC6668, GenBank AB425823.1	***SCCmec IVb/d/i (JCSC1978/6668/4469)***																					
**Reference strain JCSC4469**	**SCCmec IVb/d/i (JCSC1978/6668/4469)**	**CC5**	**POS**	**AMB**	**AMB**	**POS**	**POS**	**POS**	**POS**	**NEG**	**POS**	**AMB**	**AMB**	**NEG**	**POS**	**NEG**	**NEG**	**POS**	**NEG**	**POS**	**NEG**	**POS**
**UKD-1103429**	SCCmec IVb/d/i (JCSC1978/6668/4469)	CC7	POS	AMB	NEG	POS	POS	POS	POS	NEG	POS	NEG	AMB	NEG	NEG	NEG	NEG	POS	NEG	POS	NEG	POS
**UKD-1306141**	SCCmec IVb/d/i (JCSC1978/6668/4469)	CC7	AMB	POS	AMB	POS	POS	POS	POS	NEG	POS	AMB	AMB	NEG	NEG	NEG	NEG	POS	NEG	POS	NEG	POS
**UKD-0500089**	SCCmec IVb/d/i (JCSC1978/6668/4469)	CC5	POS	AMB	NEG	POS	POS	POS	NEG	NEG	POS	NEG	NEG	NEG	POS	NEG	NEG	POS	NEG	POS	NEG	POS
**UKD-0501416**	SCCmec IVb/d/i (JCSC1978/6668/4469)	CC5	POS	NEG	NEG	POS	POS	NEG	AMB	NEG	POS	NEG	NEG	NEG	POS	NEG	NEG	AMB	NEG	AMB	NEG	AMB
**UKD-0934574**	SCCmec IVb/d/i (JCSC1978/6668/4469)	CC5	POS	AMB	NEG	POS	POS	POS	POS	NEG	POS	NEG	NEG	NEG	POS	NEG	NEG	POS	NEG	POS	NEG	POS
**UKD-0746779**	SCCmec IVb/d/i (JCSC1978/6668/4469)	CC45 [agr I]	NEG	NEG	NEG	NEG	NEG	NEG	NEG	POS	NEG	NEG	POS	NEG	NEG	NEG	NEG	NEG	POS	NEG	POS	NEG
**UKD-0812518**	SCCmec IVb/d/i (JCSC1978/6668/4469)	CC45 [agr I]	NEG	NEG	NEG	NEG	NEG	NEG	NEG	POS	AMB	NEG	POS	NEG	NEG	NEG	NEG	NEG	POS	NEG	NEG	NEG
Prediction for 21209, GenBank AGRP	***SCCmec IVb/d/i (21209)***	***CC8***	***POS***	***NEG***	***NEG***	***POS***	***POS***	***POS***	***POS***	***NEG***	***POS***	***NEG***	***NEG***	***POS***	***NEG***	***NEG***	***NEG***	***POS***	***NEG***	***POS***	***NEG***	***POS***
**UKD-0233579**	SCCmec IVb/d/i (21209)	CC8	POS	AMB	NEG	POS	POS	POS	POS	NEG	POS	AMB	NEG	POS	NEG	NEG	NEG	POS	NEG	POS	NEG	POS
**UKD-0504920**	SCCmec IVb/d/i (21209)	CC8	POS	AMB	AMB	POS	POS	POS	NEG	NEG	POS	NEG	NEG	POS	NEG	NEG	NEG	POS	NEG	POS	NEG	POS
**UKD-0725999**	SCCmecIV b/d/i+Cu	CC30	NEG	POS	NEG	NEG	NEG	NEG	NEG	POS	NEG	NEG	POS	NEG	NEG	NEG	POS	NEG	POS	NEG	POS	NEG
Prediction for IS-105, GenBank AHLR	***SCCmec IVc (IS-105)***	***CC22***	***NEG***	***NEG***	***POS***	***NEG***	***NEG***	***POS***	***POS***	***NEG***	***NEG***	***NEG***	***POS***	***NEG***	***NEG***	***NEG***	***NEG***	***NEG***	***NEG***	***AMB***	***NEG***	***POS***
**UKD-0402421**	SCCmec IVc (IS-105), merA/B+	CC5	POS	AMB	NEG	POS	POS	POS	POS	NEG	POS	NEG	NEG	NEG	POS	NEG	NEG	POS	NEG	POS	NEG	POS
**UKD-0731319**	SCCmec IVc (IS-105), merA/B+	CC5	POS	AMB	AMB	POS	POS	POS	POS	NEG	AMB	AMB	POS	NEG	POS	NEG	NEG	POS	NEG	POS	NEG	POS
**UKD-0733137**	SCCmec IVc (IS-105)	CC8	POS	AMB	NEG	POS	POS	POS	POS	NEG	POS	AMB	NEG	POS	NEG	NEG	NEG	POS	NEG	POS	NEG	POS
**UKD-0305690**	SCCmec IVc (IS-105)	CC22	NEG	AMB	POS	NEG	NEG	AMB	POS	NEG	NEG	NEG	NEG	NEG	NEG	NEG	NEG	NEG	NEG	NEG	NEG	NEG
**UKD-1424706**	SCCmec IVc (IS-105)	CC22	AMB	POS	AMB	NEG	NEG	POS	POS	NEG	POS	NEG	AMB	NEG	NEG	NEG	NEG	NEG	NEG	POS	NEG	POS
Prediction for TCH60, GenBank CP002110.1	***SCCmec IVc (TCH60)***	***CC30***	***NEG***	***POS***	***NEG***	***NEG***	***NEG***	***NEG***	***NEG***	***POS***	***NEG***	***NEG***	***POS***	***NEG***	***NEG***	***NEG***	***POS***	***NEG***	***POS***	***NEG***	***POS***	***AMB***
**Reference strain JCSC4788**	**SCCmec IVc (TCH60)**	**CC8**	**POS**	**AMB**	**NEG**	**POS**	**POS**	**POS**	**POS**	**NEG**	**POS**	**AMB**	**AMB**	**POS**	**NEG**	**NEG**	**NEG**	**POS**	**NEG**	**POS**	**NEG**	**POS**
**UKD-1318318**	SCCmec IVc (TCH60)	CC5	POS	AMB	NEG	POS	POS	POS	POS	NEG	POS	NEG	AMB	NEG	POS	NEG	NEG	POS	NEG	POS	NEG	POS
**UKD-14580050**	SCCmec IVc (TCH60)	CC5	POS	AMB	AMB	POS	POS	POS	POS	NEG	POS	AMB	AMB	NEG	POS	NEG	NEG	POS	NEG	POS	NEG	POS
**UKD-08F01314**	SCCmec IVc (TCH60)	CC8	POS	AMB	NEG	POS	POS	POS	AMB	NEG	POS	NEG	NEG	POS	NEG	NEG	NEG	POS	NEG	POS	NEG	POS
**KHDN-50361518**	SCCmec IVc (TCH60)	CC8	AMB	POS	AMB	POS	POS	POS	POS	NEG	POS	AMB	AMB	POS	NEG	NEG	NEG	POS	NEG	POS	NEG	POS
**UKD-0307912**	SCCmec IVc (TCH60)	CC22	AMB	AMB	POS	NEG	NEG		POS	NEG	NEG	NEG		NEG	NEG	NEG	NEG	NEG	NEG		NEG	
**UKD-0220390**	SCCmec IVc (TCH60)	CC45 [agr I]	NEG	NEG	NEG	NEG	NEG	NEG	NEG	POS	AMB	NEG	POS	NEG	NEG	NEG	NEG	NEG	POS	NEG	NEG	NEG
**UKD-0311106**	SCCmec IVc (TCH60)	CC45 [agr I]	NEG	AMB	POS	NEG	NEG	NEG	NEG	POS	AMB	AMB	POS	NEG	NEG	NEG	NEG	NEG	POS	NEG	AMB	NEG
**UKD-0719855**	SCCmec IVc (TCH60)	CC45 [agr I]	NEG	NEG	NEG	NEG	NEG	NEG	NEG	POS	NEG	NEG	POS	NEG	NEG	NEG	NEG	NEG	POS	NEG	AMB	POS
**UKD-0422079**	SCCmec IVc (TCH60)	CC45 [agr I]	NEG	NEG	NEG	NEG	NEG	NEG	NEG	POS	AMB	NEG	POS	NEG	NEG	NEG	NEG	NEG	POS	NEG	NEG	NEG
**UKD-0409235**	SCCmec IVc (TCH60)	CC80	POS	AMB	NEG	POS	POS	POS	POS	NEG	POS	NEG	AMB	NEG	NEG	NEG	NEG	POS	NEG	POS	NEG	POS
**UKD-0409807**	SCCmec IVc (TCH60)	CC80	POS	AMB	NEG	POS	POS	POS	POS	NEG	POS	AMB	NEG	NEG	NEG	NEG	NEG	POS	NEG	POS	NEG	POS
**UKD-0416073**	SCCmec IVc (TCH60)	CC80	POS	NEG	NEG	POS	POS	POS	POS	NEG	POS	NEG	NEG	NEG	NEG	NEG	NEG	POS	NEG	POS	NEG	POS
**UKD-0431728**	SCCmec IVc (TCH60)	CC80	POS	NEG	NEG	POS	POS	POS	POS	NEG	POS	NEG	NEG	NEG	NEG	NEG	NEG	POS	NEG	POS	NEG	POS
**UKD-1001508**	SCCmec IVc (TCH60)	CC80	AMB	POS	AMB	POS	POS	POS	POS	NEG	POS	AMB	AMB	NEG	NEG	NEG	NEG	POS	NEG	POS	NEG	POS
**UKD-1010412**	SCCmec IVc (TCH60)	CC80	AMB	POS	AMB	POS	POS	POS	POS	NEG	POS	AMB	AMB	NEG	NEG	AMB	NEG	POS	NEG	POS	NEG	POS
**UKD-12753005**	SCCmec IVc (TCH60)	CC80	AMB	POS	AMB	POS	POS	POS	POS	NEG	POS	AMB	AMB	NEG	NEG	NEG	NEG	POS	NEG	POS	NEG	POS
**UKD-15574809**	SCCmec IVc (TCH60)	CC80	POS	AMB	AMB	POS	POS	POS	POS	NEG	POS	AMB	AMB	NEG	NEG	NEG	NEG	POS	NEG	POS	NEG	POS
**KHDN-16120291**	SCCmec IVc (TCH60)	CC88	POS	AMB	NEG	POS	POS	POS	POS	NEG	POS	NEG	NEG	NEG	NEG	NEG	NEG	POS	NEG	POS	NEG	POS
Prediction for WW2703/97, GenBank ACSW	***SCCmec IVc (WW2703/97)***	***CC30***	***NEG***	***POS***	***NEG***	***NEG***	***NEG***	***NEG***	***NEG***	***POS***	***NEG***	***NEG***	***POS***	***NEG***	***NEG***	***NEG***	***POS***	***NEG***	***POS***	***NEG***	***POS***	***AMB***
**UKD-14580088**	SCCmec IVc (WW2703/97)	CC398	AMB	AMB	POS	NEG	NEG	NEG	NEG	POS	AMB	NEG	POS	NEG	NEG	NEG	POS	NEG	POS	NEG	POS	POS
Prediction for SA_ST125_MupR, GenBank ASTH	***SCCmec IVc+speG+ccrAB4 (SA_ST125)***	***CC5***	***POS***	***NEG***	***NEG***	***POS***	***POS***	***POS***	***POS***	***NEG***	***POS***	***NEG***	***NEG***	***NEG***	***POS***	***NEG***	***NEG***	***POS***	***NEG***	***POS***	***NEG***	***POS***
**UKD-0439909**	SCCmec IVc+speG+ccrAB4 (SA_ST125)	CC5	POS	AMB	AMB	POS	POS	POS	POS	NEG	POS	NEG	NEG	NEG	POS	NEG	NEG	POS	NEG	POS	NEG	POS
Prediction for MRSA177, GenBank AECP	***SCCmec IVc+Cu/Hg (MRSA177)***		***POS***	***NEG***	***NEG***	***POS***	***POS***	***POS***	***POS***	***NEG***	***POS***	***NEG***	***NEG***	***POS***	***NEG***	***NEG***	***NEG***	***POS***	***NEG***	***POS***	***NEG***	***POS***
**UKD-1279734**	SCCmec IVc+Cu/Hg (MRSA177)	CC8	AMB	POS	AMB	POS	POS	POS	POS	NEG	POS	AMB	AMB	POS	NEG	NEG	NEG	POS	NEG	POS	NEG	POS
**UKD-14580899**	SCCmec IVc+Cu/Hg (MRSA177)	CC8	AMB	POS	AMB	POS	POS	POS	POS	NEG	POS	AMB	AMB	POS	NEG	NEG	NEG	POS	NEG	POS	NEG	POS
**UKD-16002228**	SCCmec IVc+Cu/Hg (MRSA177)	CC8	POS	AMB	AMB	POS	POS	POS	POS	NEG	POS	AMB	AMB	POS	NEG	NEG	NEG	POS	NEG	POS	NEG	POS
Prediction for HO 5096 0412, GenBank HE681097.1	***SCCmec IVh/j (HO50960412/JCSC6670)***	***CC22***	***NEG***	***NEG***	***POS***	***NEG***	***NEG***	***POS***	***POS***	***NEG***	***NEG***	***NEG***	***POS***	***NEG***	***NEG***	***NEG***	***NEG***	***NEG***	***NEG***	***AMB***	***NEG***	***POS***
Prediction for JCSC6670, GenBank AB425824.1	***SCCmec IVh/j (HO50960412/JCSC6670)***																					
**Reference strain Dublin_DSH_E1749**	**SCCmec IVh/j (HO50960412/JCSC6670)**		**AMB**	**AMB**	**POS**	**NEG**	**NEG**	**POS**	**POS**	**NEG**	**NEG**	**NEG**	**NEG**	**NEG**	**NEG**	**NEG**	**NEG**	**NEG**	**NEG**	**NEG**	**NEG**	**NEG**
**UKD-14576248**	SCCmec IVh/j (HO50960412/JCSC6670)	CC8	POS	NEG	NEG	POS	POS	POS	POS	NEG	POS	AMB	AMB	POS	NEG	NEG	NEG	POS	NEG	POS	NEG	POS
**UKD-0120122**	SCCmec IVh/j (HO50960412/JCSC6670)	CC22	NEG	NEG	POS	NEG	NEG	POS	POS	NEG	NEG	NEG	NEG	NEG	NEG	NEG	NEG	NEG	NEG	NEG	NEG	NEG
**UKD-0205319**	SCCmec IVh/j (HO50960412/JCSC6670)	CC22	AMB	AMB	POS	NEG	NEG	POS	POS	NEG	POS	NEG	NEG	NEG	NEG	NEG	NEG	NEG	NEG	NEG	NEG	NEG
**UKD-0221259**	SCCmec IVh/j (HO50960412/JCSC6670)	CC22	AMB	AMB	POS	NEG	NEG	POS	POS	NEG	NEG	NEG	NEG	NEG	NEG	NEG	NEG	NEG	NEG	NEG	NEG	NEG
**UKD-0418654**	SCCmec IVh/j (HO50960412/JCSC6670)	CC22	AMB	AMB	POS	NEG	NEG	POS	POS	NEG	NEG	NEG	NEG	NEG	NEG	NEG	NEG	NEG	NEG	NEG	NEG	NEG
**UKD-0429721**	SCCmec IVh/j (HO50960412/JCSC6670)	CC22	NEG	NEG	POS	NEG	NEG	NEG	NEG	NEG	NEG	NEG	NEG	NEG	NEG	NEG	NEG	NEG	NEG	NEG	NEG	NEG
**UKD-1116194**	SCCmec IVh/j (HO50960412/JCSC6670)	CC22	AMB	POS	AMB	NEG	NEG	POS	POS	NEG	AMB	NEG	POS	NEG	NEG	NEG	NEG	NEG	NEG	AMB	NEG	POS
**UKD-1122367**	SCCmec IVh/j (HO50960412/JCSC6670)	CC22	AMB	POS	AMB	NEG	NEG	POS	POS	NEG	AMB	NEG	POS	NEG	NEG	NEG	NEG	NEG	NEG	AMB	NEG	POS
**UKD-14012840**	SCCmec IVh/j (HO50960412/JCSC6670)	CC22	AMB	POS	AMB	NEG	NEG	POS	POS	NEG	POS	NEG	AMB	NEG	NEG	NEG	NEG	NEG	NEG	NEG	NEG	AMB
**UKD-14579833**	SCCmec IVh/j (HO50960412/JCSC6670)	CC22	AMB	POS	AMB	NEG	NEG	POS	POS	NEG	POS	NEG	NEG	NEG	NEG	NEG	NEG	NEG	NEG	NEG	NEG	NEG
**UKD-15579198**	SCCmec IVh/j (HO50960412/JCSC6670)	CC22	POS	AMB	AMB	NEG	NEG	POS	POS	NEG	POS	AMB	AMB	NEG	NEG	NEG	NEG	NEG	NEG	NEG	NEG	POS
**UKD-4052193**	SCCmec IVh/j (HO50960412/JCSC6670)	CC22	POS	AMB	AMB	NEG	NEG	POS	POS	NEG	AMB	AMB	POS	NEG	NEG	NEG	NEG	NEG	NEG	AMB	NEG	POS
**UKD-124968**	SCCmec IVh/j (HO50960412/JCSC6670)	CC22	AMB	POS	AMB	NEG	NEG	POS	POS	NEG	POS	NEG	AMB	NEG	NEG	NEG	NEG	NEG	NEG	NEG	NEG	AMB
**UKD-14572546**	SCCmec IVh/j (HO50960412/JCSC6670)	CC22	AMB	POS	AMB	NEG	NEG	POS	POS	NEG	POS	AMB	AMB	NEG	NEG	NEG	NEG	NEG	NEG	NEG	NEG	POS
**UKD-14582287**	SCCmec IVh/j (HO50960412/JCSC6670)	CC22	POS	AMB	AMB	NEG	NEG	POS	POS	NEG	POS	NEG	AMB	NEG	NEG	NEG	NEG	NEG	NEG	NEG	NEG	POS
**UKD-14583376**	SCCmec IVh/j (HO50960412/JCSC6670)	CC22	POS	AMB	AMB	NEG	NEG	POS	POS	AMB	POS	NEG	AMB	NEG	NEG	NEG	NEG	NEG	NEG	AMB	NEG	POS
**UKD-14583496**	SCCmec IVh/j (HO50960412/JCSC6670)	CC22	AMB	POS	AMB	NEG	NEG	POS	POS	POS	POS	NEG	AMB	NEG	NEG	AMB	NEG	NEG	NEG	NEG	NEG	POS
**UKD-0013324**	SCCmec IVh/j (HO50960412/JCSC6670)	CC22	NEG	AMB	POS	NEG	NEG	POS	POS	NEG	NEG	NEG	NEG	NEG	NEG	NEG	NEG	NEG	NEG	NEG	NEG	NEG
**UKD-0125329**	SCCmec IVh/j (HO50960412/JCSC6670)	CC22	AMB	AMB	POS	NEG	NEG	POS	POS	NEG	NEG	NEG	NEG	NEG	NEG	NEG	NEG	NEG	NEG	NEG	NEG	NEG
**UKD-0215498**	SCCmec IVh/j (HO50960412/JCSC6670)	CC22	AMB	AMB	POS	NEG	NEG	POS	POS	NEG	NEG	NEG	NEG	NEG	NEG	NEG	NEG	NEG	NEG	NEG	NEG	NEG
Prediction for M08-0126, GenBank FR753166.1	***SCCmec IVh/j+ACME2 (M08-0126)***	***CC22***																				
**UKD-1379483I**	SCCmec IVh/j+ACME2 (M08-0126)	CC22	AMB	POS	AMB	NEG	NEG	POS	POS	NEG	POS	AMB	AMB	NEG	NEG	NEG	NEG	NEG	NEG	AMB	NEG	POS
**UKD-5150204**	SCCmec IV+speG+Cu/czrC+ccrAB4	CC22	AMB	POS	AMB	NEG	NEG	POS	POS	NEG	POS	NEG	AMB	NEG	NEG	NEG	NEG	NEG	NEG	AMB	NEG	POS
**UKD-15578423**	SCCmec IV+speG+czrC+ccrAB4 var. 1	CC22	AMB	AMB	POS	NEG	NEG	POS	POS	NEG	POS	NEG	AMB	NEG	NEG	NEG	NEG	NEG	NEG	NEG	NEG	POS
**UKD-16582870**	SCCmec IV+speG+czrC+ccrAB4 var. 1	CC22	NEG	AMB	POS	NEG	NEG	POS	POS	NEG	NEG	NEG	NEG	NEG	NEG	NEG	NEG	NEG	NEG	NEG	NEG	NEG
**UKD-15571940**	SCCmec IV+speG+czrC+ccrAB4 var. 2	CC22	AMB	POS	AMB	NEG	NEG	POS	POS	NEG	POS	AMB	AMB	NEG	NEG	AMB	NEG	NEG	NEG	NEG	NEG	POS

Isolate ID	SCCmec type	Clonal complex	VIRULENCE : STAPHYLOCOCCAL SUPERANTIGEN/ENTEROTOXIN-LIKE GENES (SET/SSL)																			
			staphylococcal superantigen-like protein 7			staphylococcal superantigen-like protein 8		staphylococcal superantigen-like protein 9			staphylococcal superantigen-like protein 10			staphylococcal superantigene-like protein 11				staphylococcal exotoxin-like protein, second locus				
			ssl07/set1	ssl07/set1 (MRSA252)	ssl07/set1 (AF188836)	ssl08/set12_probe1	ssl08/set12_probe2	ssl09/set5_probe1	ssl09/set5_probe2	ssl09/set5 (MRSA252)	ssl10/set4	ssl10 (RF122)	ssl10/set4 (MRSA252)	ssl11/set2 (COL)	ssl11+set2(Mu50+N315)	ssl11+set2(MW2+MSSA476)	ssl11/set2 (MRSA252)	setB3	setB3 (MRSA252)	setB2	setB2 (MRSA252)	setB1
Prediction for CN1, GenBank CP003979.1	*SCCmec IVA (CN1)*	*CC8 (ST72)*	*POS*	*NEG*	*NEG*	*POS*	*POS*	*POS*	*POS*	*NEG*	*POS*	*NEG*	*NEG*	*NEG*	*NEG*	*NEG*	*NEG*	*POS*	*NEG*	*POS*	*NEG*	*POS*
UKD-0900871	SCCmec IVA (CN1)	CC8 (ST72)	POS	AMB	NEG	POS	POS	POS	POS	NEG	POS	NEG	NEG	NEG	NEG	NEG	NEG	POS	NEG	POS	NEG	POS
Prediction for CMFT1723, GenBank HF569096.1	*SCCmec V (Bengal Bay)*																					
KHDN-10405134	SCCmec V (Bengal Bay)	CC1	POS	AMB	NEG	POS	POS	POS	POS	NEG	POS	NEG	NEG	NEG	NEG	POS	NEG	POS	NEG	POS	NEG	POS
UKD-0932405	SCCmec V (Bengal Bay)	CC1 (ST573/772)	POS	NEG	NEG	POS	POS	POS	POS	NEG	POS	NEG	NEG	NEG	NEG	NEG	NEG	POS	NEG	POS	NEG	POS
UKD-1003510	SCCmec V (Bengal Bay)	CC1 (ST573/772)	POS	NEG	AMB	POS	POS	POS	POS	NEG	POS	AMB	AMB	NEG	NEG	NEG	NEG	POS	NEG	POS	NEG	POS
UKD-1112420	SCCmec V (Bengal Bay)	CC1 (ST573/772)	POS	NEG	NEG	POS	POS	POS	POS	NEG	POS	AMB	AMB	NEG	NEG	NEG	NEG	POS	NEG	POS	NEG	POS
UKD-1275832	SCCmec V (Bengal Bay)	CC1 (ST573/772)	POS	NEG	AMB	POS	POS	POS	POS	NEG	POS	AMB	AMB	NEG	NEG	NEG	NEG	POS	NEG	POS	NEG	POS
UKD-13023811	SCCmec V (Bengal Bay)	CC1 (ST573/772)	POS	NEG	NEG	POS	POS	POS	POS	NEG	POS	NEG	AMB	NEG	NEG	NEG	NEG	POS	NEG	POS	NEG	POS
UKD-14572163	SCCmec V	CC5	POS	AMB	AMB	POS	POS	POS	POS	NEG	POS	AMB	AMB	NEG	POS	NEG	NEG	POS	NEG	POS	NEG	POS
KHDN-10421133	SCCmec V+fus	CC97	AMB	POS	AMB	POS	POS	POS	POS	NEG	POS	AMB	AMB	NEG	NEG	NEG	NEG	POS	NEG	POS	NEG	POS
KHDN-21343541	SCCmec V+fus+tir+ccrAB1	CC1	AMB	POS	AMB	POS	POS	POS	POS	NEG	POS	AMB	AMB	NEG	NEG	POS	NEG	POS	NEG	POS	NEG	POS
KHDN-10416893	SCCmec V+czrC+speG+ccrAB4	CC22	AMB	AMB	POS	NEG	NEG	POS	POS	NEG	POS	NEG	NEG	NEG	NEG	NEG	NEG	NEG	NEG	NEG	NEG	NEG
Prediction for GR1, GenBank AJLX	*SCCmec VT (GR1)*	*CC361*	*POS*	*NEG*	*NEG*	*POS*	*POS*	*POS*	*POS*	*NEG*	*POS*	*NEG*	*NEG*	*NEG*	*NEG*	*NEG*	*NEG*	*POS*	*NEG*	*POS*	*NEG*	*POS*
UKD-0422872	SCCmec VT (GR1)	CC152	NEG	NEG	NEG	NEG	NEG	NEG	NEG	NEG	POS	NEG	AMB	NEG	NEG	NEG	NEG	POS	NEG	POS	NEG	POS
Prediction for PM1, GenBank BAFA	*SCCmec VT (PM1)*	*CC59*	*POS*	*NEG*	*NEG*	*POS*	*POS*	*POS*	*POS*	*NEG*	*NEG*	*POS*	*NEG*	*NEG*	*NEG*	*NEG*	*NEG*	*POS*	*NEG*	*POS*	*NEG*	*POS*
KHDN-000871	SCCmec VT (PM1)	CC45 (agr I)	NEG	AMB	POS	NEG	NEG	NEG	NEG	POS	POS	NEG	POS	NEG	NEG	NEG	NEG	NEG	POS	NEG	POS	AMB
UKD-0725052	SCCmec VT (PM1)	CC59	POS	NEG	NEG	POS	POS	AMB	POS	NEG	POS	POS	NEG	NEG	NEG	NEG	NEG	POS	NEG	POS	NEG	POS
UKD-1312138	SCCmec VT (PM1)	CC59	AMB	POS	AMB	POS	POS	POS	POS	NEG	POS	AMB	AMB	NEG	NEG	NEG	NEG	POS	NEG	POS	NEG	POS
UKD-Z4728	SCCmec VT (PM1)	CC59	POS	AMB	AMB	POS	POS	POS	POS	NEG	POS	AMB	NEG	NEG	NEG	NEG	NEG	POS	NEG	POS	NEG	POS
UKD-K0199	SCCmec VT unknown var.1	CC7	POS	AMB	NEG	POS	POS	POS	POS	NEG	POS	NEG	NEG	NEG	NEG	NEG	NEG	POS	NEG	POS	NEG	POS
UKD-15584619	SCCmec VT unknown var.2	CC30	AMB	POS	AMB	NEG	NEG	NEG	NEG	POS	AMB	AMB	POS	NEG	NEG	NEG	POS	NEG	POS	NEG	POS	POS
UKD-0712568	SCCmec VT unknown var.3	CC45 (agr I)	NEG	NEG	NEG	NEG	NEG	NEG	NEG	POS	AMB	NEG	POS	NEG	NEG	NEG	POS	NEG	POS	NEG	AMB	NEG
KHDN-10420296	SCCmec VT+ACME2	CC45 (agr I)	NEG	AMB	POS	NEG	NEG	NEG	NEG	POS	AMB	NEG	POS	NEG	NEG	NEG	NEG	NEG	POS	NEG	POS	POS
UKD-15577956	SCCmec VT+Cu/czrC+ccrA1	CC398	AMB	POS	AMB	NEG	NEG	NEG	NEG	POS	AMB	NEG	POS	NEG	NEG	NEG	POS	NEG	POS	NEG	POS	POS
Prediction for S0385, GenBank AM990992	*SCCmec VT+czrC (SO385)*	*CC398*	*NEG*	*NEG*	*POS*	*NEG*	*NEG*	*NEG*	*NEG*	*POS*	*NEG*	*NEG*	*POS*	*NEG*	*NEG*	*NEG*	*POS*	*NEG*	*POS*	*NEG*	*POS*	*AMB*
UKD-0628560	SCCmec VT+czrC (SO385)	CC8	POS	NEG	NEG	POS	POS	POS	AMB	NEG	POS	NEG	NEG	POS	NEG	NEG	NEG	POS	NEG	POS	NEG	POS
UKD-0510018	SCCmec VT+czrC (SO385)	CC398	AMB	AMB	POS	NEG	NEG	NEG	NEG	NEG	NEG	NEG	POS	NEG	NEG	NEG	POS	NEG	POS	NEG	POS	AMB
UKD-0903558	SCCmec VT+czrC (SO385)	CC398	AMB	AMB	POS	NEG	NEG	NEG	NEG	POS	AMB	NEG	POS	NEG	NEG	NEG	AMB	NEG	POS	NEG	POS	POS
UKD-0929815	SCCmec VT+czrC (SO385)	CC398	AMB	AMB	POS	NEG	NEG	NEG	NEG	POS	AMB	NEG	POS	NEG	NEG	NEG	AMB	NEG	POS	NEG	POS	POS
UKD-0907762	SCCmec VT+czrC (SO385)	CC398	AMB	AMB	POS	NEG	NEG	NEG	NEG	POS	AMB	NEG	POS	NEG	NEG	NEG	POS	NEG	POS	NEG	POS	POS
UKD-1025105	SCCmec VT+czrC (SO385)	CC398	NEG	AMB	POS	NEG	NEG	NEG	NEG	AMB	AMB	NEG	POS	NEG	NEG	NEG	NEG	NEG	POS	NEG	POS	POS
UKD-11780898	SCCmec VT+czrC (SO385)	CC398	AMB	POS	AMB	NEG	NEG	NEG	NEG	POS	AMB	NEG	POS	NEG	NEG	NEG	NEG	NEG	POS	NEG	POS	POS
UKD-1377009	SCCmec VT+czrC (SO385)	CC398	AMB	POS	AMB	NEG	NEG	NEG	NEG	POS	AMB	NEG	POS	NEG	NEG	NEG	POS	NEG	POS	NEG	POS	POS
UKD-143406	SCCmec VT+czrC (SO385)	CC398	AMB	POS	AMB	NEG	NEG	NEG	NEG	POS	AMB	NEG	POS	NEG	NEG	NEG	POS	NEG	POS	NEG	POS	POS
UKD-1400363	SCCmec VT+czrC (SO385)	CC398	AMB	AMB	POS	NEG	NEG	NEG	NEG	POS	POS	NEG	AMB	NEG	NEG	NEG	POS	NEG	POS	NEG	POS	POS
UKD-14571852	SCCmec VT+czrC (SO385)	CC398	AMB	POS	AMB	NEG	NEG	NEG	NEG	POS	AMB	AMB	POS	NEG	NEG	NEG	POS	NEG	POS	NEG	POS	POS
UKD-14582574	SCCmec VT+czrC (SO385)	CC398	AMB	POS	AMB	NEG	NEG	NEG	NEG	POS	AMB	NEG	POS	NEG	NEG	NEG	POS	NEG	POS	NEG	POS	POS
UKD-152108	SCCmec VT+czrC (SO385)	CC398	AMB	POS	AMB	NEG	NEG	NEG	NEG	POS	AMB	NEG	POS	NEG	NEG	NEG	POS	NEG	POS	NEG	POS	POS
UKD-15573321	SCCmec VT+czrC (SO385)	CC398	AMB	POS	AMB	NEG	NEG	NEG	NEG	POS	AMB	NEG	POS	NEG	NEG	NEG	POS	NEG	POS	NEG	POS	POS
UKD-15584034	SCCmec VT+czrC , ydhK-neg. variant	CC398	AMB	POS	AMB	NEG	NEG	NEG	NEG	POS	AMB	NEG	POS	NEG	NEG	NEG	POS	NEG	POS	NEG	POS	POS
UKD-0932581	SCCmecVT+fus+czrC	CC22	NEG	AMB	POS	NEG	NEG	POS	POS	NEG	NEG	NEG	NEG	NEG	NEG	NEG	NEG	NEG	NEG	NEG	NEG	NEG
UKD-1121994	SCCmecVT+fus+czrC	CC22	AMB	POS	AMB	NEG	NEG	POS	POS	NEG	POS	NEG	AMB	NEG	NEG	NEG	NEG	NEG	NEG	NEG	NEG	POS
UKD-1272252	SCCmecVT+fus+czrC	CC22	AMB	POS	AMB	NEG	NEG	POS	POS	NEG	AMB	NEG	POS	NEG	NEG	NEG	NEG	NEG	NEG	POS	NEG	POS
Prediction for MRSA18	*SCCmec VI+fus (MRSA18)*																					
UKD-1103380	SCCmec VI+fus (MRSA18)	CC7	POS	AMB	NEG	POS	POS	POS	POS	NEG	POS	NEG	NEG	NEG	NEG	NEG	NEG	POS	NEG	POS	NEG	POS
Prediction for M10/0061, GenBank FR823292.1	*SCCmec XI (LGA251/M10-61)*	*CC130*																				
**Reference strain Dublin-DSH_M10 0061**	**SCCmec XI (LGA251/M10-61)**	**CC130**	**POS**	**AMB**	**NEG**	**POS**	**POS**	**POS**	**POS**	**NEG**	**POS**	**AMB**	**AMB**	**NEG**	**NEG**	**NEG**	**NEG**	**POS**	**NEG**	**POS**	**NEG**	**POS**
Prediction for LGA251, GenBank FR821779.1	*SCCmec XI (LGA251/M10-61)*	*ST425*																				
UKD-14015835	SCCmec XI (LGA251/M10-61)	CC130	POS	AMB	AMB	POS	POS	POS	POS	NEG	POS	AMB	NEG	NEG	NEG	NEG	NEG	POS	NEG	POS	NEG	POS
UKD-16573252	SCCmec XI (LGA251/M10-61)	CC130	POS	POS	NEG	POS	POS	POS	POS	NEG	POS	AMB	NEG	NEG	NEG	NEG	NEG	POS	NEG	POS	NEG	POS
UKD-0217002	PseudoSCCmec, class B mec complex+Hg	CC8	POS	AMB	NEG	POS	POS	POS	POS	NEG	POS	NEG	NEG	POS	NEG	NEG	NEG	POS	NEG	POS	NEG	POS
UKD-0008510	PseudoSCCmec, class B mec complex+Hg	CC8	POS	NEG	NEG	POS	POS	POS	POS	NEG	POS	NEG	NEG	POS	NEG	NEG	NEG	POS	NEG	POS	NEG	POS
UKD-0110962	PseudoSCCmec, class B mec complex+Hg	CC8	POS	AMB	AMB	POS	POS	POS	POS	NEG	NEG	AMB	NEG	POS	NEG	NEG	NEG	POS	NEG	POS	NEG	POS
KHDN-10408593	PseudoSCCmec, class B mec complex+Cu/Hg	CC8	POS	AMB	NEG	POS	POS	POS	POS	NEG	POS	NEG	AMB	POS	NEG	NEG	NEG	POS	NEG	POS	NEG	POS
UKD-0007201	PseudoSCCmec, class B mec complex	CC5	POS	AMB	AMB	POS	POS	POS	POS	NEG	POS	AMB	AMB	NEG	POS	NEG	NEG	POS	NEG	POS	NEG	POS
UKD-0033204	PseudoSCCmec, class B mec complex	CC5	POS	AMB	AMB	POS	POS	POS	POS	NEG	POS	NEG	AMB	NEG	POS	NEG	NEG	POS	NEG	POS	NEG	POS
UKD-0134329	PseudoSCCmec, class B mec complex	CC5	POS	NEG	NEG	POS	POS	NEG	AMB	NEG	POS	NEG	NEG	NEG	POS	NEG	NEG	AMB	NEG	AMB	NEG	AMB
UKD-0806946	PseudoSCCmec class C+As/Cu	CC398	AMB	AMB	POS	NEG	NEG	NEG	NEG	AMB	AMB	NEG	POS	NEG	NEG	NEG	NEG	NEG	POS	NEG	POS	AMB
UKD-0500610	ACME2	CC8	POS	NEG	NEG	POS	POS	POS	POS	NEG	POS	NEG	NEG	POS	NEG	NEG	NEG	POS	NEG	POS	NEG	POS
UKD-0610438	ACME2	CC8	POS	NEG	NEG	POS	POS	POS	POS	NEG	POS	AMB	AMB	POS	NEG	NEG	NEG	POS	NEG	POS	NEG	POS
KHDN-10409344	ACME2	CC8	POS	NEG	NEG	POS	POS	POS	POS	NEG	POS	NEG	AMB	POS	NEG	NEG	NEG	POS	NEG	POS	NEG	POS
UKD-0322559	ACME2+speG+czrC+ccrAB4	CC8	POS	NEG	NEG	POS	POS	POS	POS	NEG	POS	NEG	NEG	POS	NEG	NEG	NEG	POS	NEG	POS	NEG	POS
UKD-0501998	ACME2+speG+czrC+ccrAB4	CC8	POS	NEG	NEG	POS	POS	POS	POS	NEG	POS	NEG	NEG	POS	NEG	NEG	NEG	POS	NEG	POS	NEG	POS
UKD-0505213	ACME2+speG+czrC+ccrAB4	CC8	POS	NEG	NEG	POS	POS	POS	POS	NEG	POS	POS	NEG	POS	NEG	NEG	NEG	POS	NEG	POS	NEG	AMB
Prediction for MSSA476, GenBank BX571857.1	*fus+tir+ccrAB1 (MSSA476)*	*CC1*	*POS*	*NEG*	*NEG*	*POS*	*POS*	*POS*	*POS*	*NEG*	*POS*	*NEG*	*NEG*	*NEG*	*NEG*	*POS*	*NEG*	*POS*	*NEG*	*POS*	*NEG*	*POS*
**Reference strain Sanger 476**	**fus+tir+ccrAB1 (MSSA476)**	**CC1**	**POS**	**NEG**	**NEG**	**POS**	**POS**	**POS**	**POS**	**NEG**	**POS**	**NEG**	**NEG**	**NEG**	**NEG**	**POS**	**NEG**	**POS**	**NEG**	**POS**	**NEG**	**POS**
UKD-14023914	fus+tir+ccrAB1 (MSSA476)	CC1	AMB	POS	AMB	POS	POS	POS	POS	NEG	POS	AMB	AMB	NEG	NEG	POS	NEG	POS	NEG	POS	NEG	POS
UKD-K00034	kdp+ccrAB2	CC182	POS	POS	NEG	POS	POS	POS	AMB	NEG	POS	POS	NEG	NEG	NEG	NEG	POS	POS	NEG	POS	NEG	POS
UKD-15580369	As+ccrAB4	CC22	AMB	POS	AMB	NEG	NEG	POS	POS	NEG	POS	NEG	AMB	NEG	NEG	NEG	NEG	NEG	NEG	NEG	NEG	NEG
UKD-15570100	ACME3+ccrAB1 (Strain 21342)	CC80	AMB	POS	AMB	POS	POS	POS	POS	NEG	POS	AMB	AMB	NEG	NEG	NEG	NEG	POS	NEG	POS	NEG	POS
UKD-1010678	speG+czrC+ccrAB4	CC8	POS	NEG	NEG	POS	POS	POS	POS	NEG	POS	NEG	NEG	POS	NEG	NEG	NEG	POS	NEG	POS	NEG	POS
UKD-1008243	speG+czrC+ccrAB4	CC8	POS	NEG	NEG	POS	POS	POS	POS	NEG	POS	NEG	AMB	POS	NEG	NEG	NEG	POS	NEG	POS	NEG	POS
UKD-1009430	speG+czrC+ccrAB4	CC8	POS	NEG	NEG	POS	POS	POS	POS	NEG	POS	NEG	AMB	POS	NEG	NEG	NEG	POS	NEG	POS	NEG	POS
UKD-12071828	speG+czrC+ccrAB4	CC22	AMB	POS	POS	NEG	NEG	POS	POS	NEG	POS	NEG	AMB	NEG	NEG	NEG	NEG	NEG	NEG	AMB	NEG	AMB
UKD-14011883	speG+czrC+ccrAB4	CC22	AMB	POS	AMB	NEG	NEG	POS	POS	NEG	POS	NEG	AMB	NEG	NEG	NEG	NEG	NEG	NEG	NEG	NEG	AMB
UKD-15008293	speG+czrC+ccrAB4	CC22	AMB	AMB	POS	NEG	NEG	POS	POS	NEG	POS	NEG	NEG	NEG	NEG	NEG	NEG	NEG	NEG	NEG	NEG	AMB
UKD-1514877	speG+czrC+ccrAB4	CC22	AMB	POS	POS	NEG	NEG	POS	POS	NEG	POS	NEG	AMB	NEG	NEG	NEG	NEG	NEG	NEG	NEG	NEG	POS
UKD-15580971	speG+czrC+ccrAB4	CC22	AMB	POS	POS	NEG	NEG	POS	POS	NEG	POS	NEG	POS	NEG	NEG	NEG	NEG	NEG	NEG	NEG	NEG	POS
UKD-15582646	speG+czrC+ccrAB4	CC22	AMB	POS	POS	NEG	NEG	POS	POS	NEG	POS	NEG	AMB	NEG	NEG	NEG	NEG	NEG	NEG	NEG	NEG	POS

Isolate ID	SCCmec type	Clonal complex	CAPSULE-ASSOCIATED GENES			BIOFILM-ASSOCIATED GENES				ADHESION FACTORS / GENES ENCODING MICROBIAL SURFACE COMPONENTS RECOGNIZING ADHESIVE MATRIX MOLECULES (MSCRAMM GENES)																		
			Capsule type 1	Capsule type 5	Capsule type 8	intercellular adhesion protein A	intercellular adhesion protein C	biofilm PIA synthesis protein D	surface protein involved in biofilm formation	bone sialoprotein-binding protein							clumping factor A					clumping factor B					collagen-binding adhesin	cell wall associated fibronectin-binding protein
			cap 1	cap 5	cap 8	icaA	icaC	icaD	bap	bbp	bbp (cons)	bbp (COL+MW2)	bbp (MRSA252)	bbp (Mu50)	bbp (RF122)	bbp (ST45)	clfA	clfA (cons)	clfA (COL+RF122)	clfA (MRSA252)	clfA (Mu50+MW2)	clfB	clfB (cons)	clfB (COL+Mu50)	clfB (MW2)	clfB (RF122)	cna	ebh (cons)
VIRTUAL HYBRIDISATION for COL, GenBank CP000046	SCCmec I (COL)	CC8	NEG	POS	NEG	POS	POS	POS	NEG	POS	POS	POS	NEG	NEG	NEG	NEG	POS	POS	POS	NEG	AMB	POS	POS	POS	NEG	NEG	NEG	POS
Reference strain COL	SCCmec I (COL)	CC8	NEG	POS	NEG	POS	POS	POS	NEG	POS	POS	POS	NEG	NEG	NEG	NEG	POS	POS	POS	NEG	POS	POS	POS	POS	NEG	NEG	NEG	POS
UKD-0007063	SCCmec I (COL)	CC5	AMB	POS	NEG	POS	POS	POS	NEG	POS	POS	AMB	NEG	POS	NEG	AMB	POS	POS	NEG	NEG	POS	POS	POS	POS	NEG	NEG	NEG	POS
UKD-0220759	SCCmec I (COL)	CC5	NEG	POS	NEG	POS	POS	POS	NEG	POS	POS	NEG	NEG	POS	NEG	NEG	POS	POS	NEG	NEG	POS	POS	POS	POS	NEG	NEG	NEG	POS
UKD-0308986	SCCmec I (COL)	CC5	NEG	POS	NEG	POS	POS	POS	NEG	POS	POS	AMB	NEG	POS	NEG	AMB	POS	POS	NEG	NEG	POS	POS	POS	POS	NEG	NEG	NEG	POS
UKD-0500009	SCCmec I (COL)	CC5	NEG	POS	NEG	POS	POS	POS	NEG	POS	POS	NEG	NEG	POS	NEG	NEG	NEG	NEG	NEG	NEG	NEG	POS	POS	POS	NEG	NEG	NEG	POS
UKD-0508483	SCCmec I (COL)	CC5	NEG	POS	NEG	POS	POS	POS	NEG	POS	POS	NEG	NEG	POS	NEG	NEG	POS	POS	NEG	NEG	POS	POS	POS	POS	NEG	NEG	NEG	POS
UKD-0519001	SCCmec I (COL)	CC5	NEG	POS	NEG	POS	POS	POS	NEG	POS	POS	NEG	NEG	POS	NEG	NEG	POS	POS	NEG	NEG	POS	POS	POS	POS	NEG	NEG	NEG	POS
UKD-0701876	SCCmec I (COL)	CC5	NEG	POS	NEG	POS	POS	POS	NEG	NEG	NEG	NEG	NEG	NEG	NEG	NEG	POS	POS	NEG	NEG	POS	POS	POS	POS	NEG	NEG	NEG	POS
UKD-0708865	SCCmec I (COL)	CC5	NEG	POS	NEG	POS	AMB	AMB	NEG	NEG	AMB	NEG	NEG	AMB	NEG	NEG	POS	POS	NEG	NEG	POS	POS	POS	AMB	NEG	NEG	NEG	AMB
UKD-1001782	SCCmec I (COL)	CC5	NEG	POS	NEG	POS	POS	POS	NEG	POS	NEG	NEG	NEG	NEG	NEG	POS	POS	POS	NEG	NEG	POS	POS	POS	POS	NEG	NEG	NEG	POS
Prediction for PSP1996, GenBank ANHU	SCCmec I (PSP1996)	CC8	NEG	POS	NEG	POS	POS	POS	NEG	NEG	NEG	NEG	NEG	NEG	NEG	NEG	POS	POS	POS	NEG	AMB	POS	POS	POS	NEG	NEG	NEG	POS
UKD-0261727	SCCmec I (PSP1996)	CC8	NEG	POS	NEG	POS	POS	POS	NEG	NEG	NEG	NEG	NEG	NEG	NEG	NEG	POS	POS	POS	AMB	AMB	POS	POS	POS	NEG	NEG	NEG	POS
VIRTUAL HYBRIDISATION for MRSA7 : VIRTUAL HYBRII	SCCmec I+fus+tirS (Geraldine Clone)	CC5	NEG	POS	NEG	POS	POS	POS	NEG	POS	POS	NEG	NEG	POS	NEG	NEG	POS	POS	NEG	NEG	POS	POS	POS	POS	NEG	NEG	NEG	POS
UKD-1221930	SCCmec I+fus+tirS (Geraldine Clone)	CC5	NEG	POS	NEG	POS	POS	POS	NEG	POS	POS	NEG	NEG	POS	NEG	NEG	POS	POS	NEG	NEG	POS	POS	POS	POS	NEG	NEG	NEG	POS
Prediction for N315, GenBank BA000018.3	SCCmec II (N315)	CC5	NEG	POS	NEG	POS	POS	POS	NEG	POS	POS	NEG	NEG	POS	NEG	NEG	POS	POS	NEG	NEG	POS	POS	POS	POS	NEG	NEG	NEG	POS
Reference strain N315	SCCmec II (N315)	CC5	NEG	POS	NEG	POS	POS	POS	NEG	POS	POS	NEG	NEG	POS	NEG	NEG	POS	POS	NEG	NEG	POS	POS	POS	POS	NEG	NEG	NEG	POS
Prediction for MRSA252, GenBank BX571856.1	SCCmec II (N315)	CC30	NEG	NEG	POS	POS	POS	POS	NEG	POS	POS	NEG	POS	NEG	NEG	NEG	POS	POS	AMB	POS	NEG	POS	POS	NEG	NEG	NEG	POS	POS
Reference strain Sanger 252	SCCmec II (N315)	CC30	NEG	NEG	POS	POS	POS	POS	NEG	POS	POS	NEG	POS	NEG	NEG	NEG	POS	POS	NEG	POS	NEG	POS	POS	NEG	NEG	NEG	POS	POS
UKD-0230792	SCCmec II (N315)	CC30	NEG	NEG	POS	POS	POS	POS	NEG	POS	POS	NEG	POS	NEG	NEG	NEG	POS	POS	AMB	POS	NEG	POS	POS	NEG	NEG	POS	POS	POS
Prediction for Mu50, GenBank BA000017.4	SCCmec II (N315)	CC5	NEG	POS	NEG	POS	POS	POS	NEG	POS	POS	NEG	NEG	POS	NEG	NEG	POS	POS	NEG	NEG	POS	POS	POS	POS	NEG	NEG	NEG	POS
Reference strain MU50	SCCmec II (N315)	CC5	NEG	POS	NEG	POS	POS	POS	NEG	POS	POS	NEG	NEG	POS	NEG	NEG	POS	POS	NEG	NEG	POS	POS	POS	POS	NEG	NEG	NEG	POS
Prediction for JH1, GenBank CP000736.1	SCCmec II (JH1/JH9)	CC5	NEG	POS	NEG	POS	POS	POS	NEG	POS	POS	NEG	NEG	POS	NEG	NEG	POS	POS	NEG	NEG	POS	POS	POS	POS	NEG	NEG	NEG	POS
Prediction for JH9, GenBank CP000703.1	SCCmec II (JH1/JH9)	CC5	NEG	POS	NEG	POS	POS	POS	NEG	POS	POS	NEG	NEG	POS	NEG	NEG	POS	POS	NEG	NEG	POS	POS	POS	POS	NEG	NEG	NEG	POS
Reference strain Dublin-DSH_AR07.4_0237	SCCmec II (JH1/JH9)		NEG	POS	NEG	POS	POS	POS	NEG	POS	POS	AMB	NEG	POS	NEG	NEG	POS	POS	NEG	NEG	POS	POS	POS	POS	NEG	NEG	NEG	POS
Reference strain NARSA_382 / USA100	SCCmec II (JH1/JH9)	CC5	NEG	POS	NEG	POS	POS	POS	NEG	POS	POS	NEG	NEG	POS	NEG	NEG	POS	POS	NEG	NEG	POS	POS	POS	POS	NEG	NEG	NEG	POS
UKD-0046921	SCCmec II (JH1/JH9)	CC5	NEG	POS	NEG	POS	POS	POS	NEG	POS	POS	NEG	NEG	POS	NEG	NEG	POS	POS	NEG	NEG	POS	POS	POS	POS	NEG	NEG	NEG	POS
UKD-0333415	SCCmec II (JH1/JH9)	CC5	NEG	POS	NEG	POS	POS	POS	NEG	POS	POS	NEG	NEG	POS	NEG	NEG	POS	POS	NEG	NEG	POS	POS	POS	POS	NEG	NEG	NEG	POS
UKD-0341645	SCCmec II (JH1/JH9)	CC5	NEG	POS	NEG	POS	POS	POS	NEG	POS	POS	NEG	NEG	POS	NEG	NEG	POS	POS	NEG	NEG	POS	POS	POS	POS	NEG	NEG	NEG	POS
UKD-0504626	SCCmec II (JH1/JH9)	CC5	NEG	POS	NEG	POS	POS	POS	NEG	POS	POS	NEG	NEG	POS	NEG	NEG	POS	POS	NEG	NEG	POS	POS	POS	POS	NEG	NEG	NEG	POS
UKD-0507581	SCCmec II (JH1/JH9)	CC5	NEG	POS	NEG	POS	POS	POS	NEG	POS	POS	NEG	NEG	POS	NEG	NEG	POS	POS	NEG	NEG	POS	POS	POS	POS	NEG	NEG	NEG	POS
UKD-0507903	SCCmec II (JH1/JH9)	CC5	NEG	POS	NEG	POS	POS	POS	NEG	POS	POS	NEG	NEG	POS	NEG	NEG	POS	POS	NEG	NEG	POS	POS	POS	POS	NEG	NEG	NEG	POS
UKD-0700817	SCCmec II (JH1/JH9)	CC5	NEG	POS	NEG	POS	POS	POS	NEG	POS	POS	NEG	NEG	POS	NEG	NEG	POS	POS	NEG	NEG	POS	POS	POS	POS	NEG	NEG	NEG	POS
UKD-1014561	SCCmec II (JH1/JH9)	CC5	NEG	POS	NEG	POS	POS	POS	NEG	POS	POS	NEG	NEG	POS	NEG	NEG	POS	POS	NEG	NEG	POS	POS	POS	POS	NEG	NEG	NEG	POS
UKD-1037507	SCCmec II (JH1/JH9)	CC5	NEG	POS	NEG	POS	POS	POS	NEG	POS	POS	AMB	NEG	POS	NEG	NEG	POS	POS	NEG	NEG	POS	POS	POS	POS	NEG	NEG	NEG	POS
UKD-1038321	SCCmec II (JH1/JH9)	CC5	NEG	POS	NEG	POS	POS	POS	NEG	POS	POS	NEG	NEG	POS	NEG	NEG	POS	POS	NEG	NEG	POS	POS	POS	POS	NEG	NEG	NEG	POS
UKD-1111608	SCCmec II (JH1/JH9)	CC5	NEG	POS	NEG	POS	POS	POS	NEG	POS	POS	NEG	NEG	POS	NEG	NEG	POS	POS	NEG	NEG	POS	POS	POS	POS	NEG	NEG	NEG	POS
UKD-12753135	SCCmec II (JH1/JH9)	CC5	NEG	POS	NEG	POS	POS	POS	NEG	POS	POS	AMB	NEG	POS	NEG	NEG	POS	POS	NEG	NEG	POS	POS	POS	POS	NEG	NEG	NEG	POS
UKD-1312816	SCCmec II (JH1/JH9)	CC5	NEG	POS	NEG	POS	POS	POS	NEG	POS	POS	NEG	NEG	POS	NEG	NEG	POS	POS	NEG	NEG	POS	POS	POS	POS	NEG	NEG	NEG	POS
UKD-1401623	SCCmec II (JH1/JH9)	CC5	NEG	POS	NEG	POS	POS	POS	NEG	POS	POS	AMB	NEG	POS	NEG	NEG	POS	POS	NEG	NEG	POS	POS	POS	POS	NEG	NEG	NEG	POS
UKD-15571943	SCCmec II+czrC+ccrA/B-4	CC5	NEG	POS	NEG	POS	POS	POS	NEG	NEG	AMB	NEG	NEG	NEG	NEG	NEG	POS	POS	NEG	NEG	POS	POS	POS	POS	NEG	NEG	NEG	POS
UKD-15576888	SCCmec II+czrC+ccrA/B-4	CC5	NEG	POS	NEG	POS	POS	POS	NEG	NEG	NEG	NEG	NEG	NEG	NEG	NEG	POS	POS	NEG	NEG	POS	POS	POS	POS	NEG	NEG	NEG	POS
UKD-14576461	SCCmec II+speG+czrC+ccrA/B-4	CC5	NEG	POS	NEG	POS	POS	POS	NEG	NEG	NEG	NEG	NEG	NEG	NEG	NEG	POS	POS	NEG	NEG	POS	POS	POS	POS	NEG	NEG	NEG	POS
UKD-15581649	SCCmec II+speG+czrC+ccrA/B-4	CC5	NEG	POS	NEG	POS	POS	POS	NEG	NEG	NEG	NEG	NEG	NEG	NEG	NEG	POS	POS	NEG	NEG	POS	POS	POS	POS	NEG	NEG	NEG	POS
Prediction for CIGC345D, GenBank AHVO	SCCmec II A/B/D	CC8	NEG	POS	NEG	POS	POS	POS	NEG	POS	POS	POS	NEG	NEG	NEG	NEG	POS	POS	POS	NEG	AMB	POS	POS	POS	NEG	NEG	NEG	POS
UKD-12K00171	SCCmec II A/B/D	CC8	NEG	POS	NEG	POS	POS	POS	NEG	POS	POS	POS	NEG	NEG	NEG	NEG	POS	POS	AMB	POS	AMB	POS	POS	POS	NEG	NEG	NEG	POS
Prediction for JKD6008, GenBank CP002120.1	SCC [mec III+Cd] (JKD6008)	CC8 (ST239)	NEG	NEG	POS	POS	POS	POS	NEG	POS	POS	POS	NEG	NEG	NEG	NEG	POS	POS	POS	NEG	AMB	POS	POS	NEG	NEG	NEG	POS	POS
Prediction for JKD6009, GenBank ABSA	SCC [mec III+Cd] (JKD6008)	CC8 (ST239)	NEG	NEG	POS	POS	POS	POS	NEG	POS	POS	POS	NEG	NEG	NEG	NEG	POS	POS	POS	NEG	AMB	POS	POS	NEG	NEG	NEG	POS	POS
Reference strain ATCC_33592	SCC [mec III+Cd] (ATCC33592)	CC8 (ST239)	NEG	NEG	POS	POS	POS	POS	NEG	POS	POS	POS	NEG	AMB	NEG	NEG	POS	POS	POS	AMB	AMB	POS	POS	NEG	NEG	POS	POS	POS
Prediction for PPUKM-775-2009, GenBank AMRE	SCC [mec III+Cd+ccrC] (PPUKM-775-2009)	CC8 (ST239)	NEG	NEG	POS	POS	POS	POS	NEG	POS	POS	POS	NEG	NEG	NEG	NEG	POS	POS	POS	NEG	AMB	POS	POS	NEG	NEG	NEG	POS	POS
Prediction for T0131, GenBank CP002643.1	SCC [mec III+Cd+ccrC] (T0131)	CC8 (ST239)	NEG	NEG	POS	POS	POS	POS	NEG	POS	POS	POS	NEG	NEG	NEG	NEG	POS	POS	POS	NEG	AMB	POS	POS	NEG	NEG	NEG	POS	POS
Prediction for XN108, GenBank CP007447.1	SCC [mec III+Cd+ccrC] (XN108)	CC8 (ST239)	NEG	NEG	POS	POS	POS	POS	NEG	POS	POS	POS	NEG	NEG	NEG	NEG	POS	POS	POS	NEG	AMB	POS	POS	NEG	NEG	NEG	POS	POS
Prediction for CN79, GenBank ANCJ	SCC [mec III+Cd+ccrC] (CN79)	CC8 (ST239)	NEG	NEG	POS	POS	POS	POS	NEG	POS	POS	POS	NEG	NEG	NEG	NEG	POS	POS	POS	NEG	AMB	POS	POS	NEG	NEG	NEG	POS	POS
Prediction for 16K, GenBank BABZ	SCC [mec III+Cd+ccrC] (CN79)	CC8 (ST239)	NEG	NEG	POS	POS	POS	POS	NEG	POS	POS	POS	NEG	NEG	NEG	NEG	POS	POS	POS	NEG	AMB	POS	POS	NEG	NEG	NEG	POS	POS
UKD-0835987	SCC [mec III+Cd+ccrC] (CN79)	CC8 (ST239)	NEG	NEG	POS	POS	POS	POS	NEG	POS	POS	POS	NEG	NEG	NEG	NEG	POS	POS	POS	AMB	AMB	POS	POS	NEG	NEG	AMB	POS	POS
UKD-16581769	SCCmec III+Cd+ccrC	CC8 (ST239)	NEG	NEG	POS	POS	POS	POS	NEG	POS	POS	POS	NEG	NEG	NEG	NEG	POS	POS	POS	NEG	NEG	POS	POS	NEG	NEG	NEG	POS	POS
Prediction for Bmb9393, GenBank CP005288.1	SCCmec III+Cd/Hg+ccrC (Bmb9393)	CC8 (ST239)	NEG	NEG	POS	POS	POS	POS	NEG	POS	POS	POS	NEG	NEG	NEG	NEG	POS	POS	POS	NEG	AMB	POS	POS	NEG	NEG	NEG	POS	POS
UKD-15574263	SCCmec III+Cd/Hg+ccrC (Bmb9393)	CC8 (ST239)	NEG	NEG	POS	POS	POS	POS	NEG	POS	POS	POS	NEG	NEG	NEG	NEG	POS	POS	POS	AMB	AMB	POS	POS	NEG	NEG	POS	POS	POS
Prediction for SK1585, GenBank AYLT	SCCmec III+ccrC+Cd/Hg (SK1585)	CC8 (ST239)	NEG	NEG	POS	POS	POS	POS	NEG	NEG	NEG	NEG	NEG	NEG	NEG	NEG	POS	POS	AMB	NEG	POS	POS	POS	NEG	NEG	NEG	POS	POS
UKD-105861	SCCmec III+ccrC+Cd/Hg (SK1585)	CC8 (ST239)	NEG	NEG	POS	POS	POS	POS	NEG	POS	POS	POS	NEG	NEG	NEG	NEG	POS	POS	POS	AMB	AMB	POS	POS	NEG	NEG	NEG	POS	POS
UKD-134572	SCCmec III+ccrC+Cd/Hg (SK1585)	CC8 (ST239)	NEG	NEG	POS	POS	POS	POS	NEG	POS	POS	POS	NEG	NEG	NEG	NEG	POS	POS	POS	AMB	POS	POS	POS	NEG	NEG	NEG	POS	POS
UKD-139143	SCCmec III+ccrC+Cd/Hg (SK1585)	CC8 (ST239)	NEG	NEG	POS	POS	POS	POS	NEG	POS	POS	POS	NEG	NEG	NEG	NEG	POS	POS	POS	NEG	AMB	POS	POS	NEG	NEG	NEG	POS	POS
Prediction for TW20, GenBank FN433596.1	SCC [mec III+Cd/Hg+ccrC] (TW20)	CC8 (ST239)	NEG	NEG	POS	POS	POS	POS	NEG	POS	POS	POS	NEG	NEG	NEG	NEG	POS	POS	POS	NEG	AMB	POS	POS	NEG	NEG	NEG	POS	POS
Prediction for ATCC BAA-39, GenBank AEEK	SCC [mec III+Cd/Hg+ccrC] (TW20)	CC8 (ST239)	NEG	NEG	POS	POS	POS	POS	NEG	POS	POS	POS	NEG	NEG	NEG	NEG	POS	POS	POS	NEG	AMB	POS	POS	NEG	NEG	NEG	POS	POS

Isolate ID	SCCmec type	Clonal complex	CAPSULE-ASSOCIATED GENES			BIOFILM-ASSOCIATED GENES				ADHAESION FACTORS / GENES ENCODING MICROBIAL SURFACE COMPONENTS RECOGNIZING ADHESIVE MATRIX MOLECULES (MSCRAMM GENES)																		
			Capsule type 1	Capsule type 5	Capsule type 8	intercellular adhesion protein A	intercellular adhesion protein C	biofilm PIA synthesis protein D	surface protein involved in biofilm formation	bone sialoprotein-binding protein							clumping factor A					clumping factor B					collagen-binding adhesin	cell wall associated fibronectin-binding protein
			cap 1	cap 5	cap 8	icaA	icaC	icaD	bap	bbp	bbp (cons)	bbp (COL+MW2)	bbp (MRSA252)	bbp (Mu50)	bbp (RF122)	bbp (ST45)	clfA	clfA (cons)	clfA (COL+RF122)	clfA (MRSA252)	clfA (Mu50+MW2)	clfB	clfB (cons)	clfB (COL+Mu50)	clfB (MW2)	clfB (RF122)	cna	ebh (cons)
Prediction for MW2, GenBank BA000033.2	***SCCmec IVa (MW2)***	***CC1***	***NEG***	***NEG***	***POS***	***POS***	***POS***	***POS***	***NEG***	***POS***	***POS***	***POS***	***NEG***	***NEG***	***NEG***	***NEG***	***POS***	***POS***	***NEG***	***NEG***	***POS***	***POS***	***POS***	***NEG***	***POS***	***AMB***	***POS***	***POS***
**Reference strain MW2**	**SCCmec IVa (MW2)**	**CC1**	**NEG**	**NEG**	**POS**	**POS**	**POS**	**POS**	**NEG**	**POS**	**POS**	**POS**	**NEG**	**NEG**	**NEG**	**NEG**	**POS**	**POS**	**NEG**	**NEG**	**POS**	**POS**	**POS**	**NEG**	**POS**	**POS**	**POS**	**POS**
**Reference strain CA05 (JCSC1968)**	**SCCmec IVa (MW2)**	**CC45**	**NEG**	**NEG**	**POS**	**POS**	**POS**	**POS**	**NEG**	**POS**	**POS**	**NEG**	**NEG**	**AMB**	**NEG**	**POS**	**POS**	**POS**	**AMB**	**NEG**	**POS**	**POS**	**POS**	**NEG**	**POS**	**AMB**	**POS**	**POS**
**UKD-0127106**	SCCmec IVa (MW2)	CC1	NEG	NEG	POS	POS	POS	POS	NEG	POS	POS	POS	NEG	NEG	NEG	NEG	POS	POS	NEG	NEG	POS	POS	POS	NEG	POS	POS	POS	POS
**UKD-0701446**	SCCmec IVa (MW2)	CC1	NEG	NEG	POS	POS	POS	POS	NEG	POS	POS	POS	NEG	NEG	NEG	NEG	POS	POS	NEG	NEG	POS	POS	POS	NEG	POS	AMB	POS	POS
**UKD-0947486**	SCCmec IVa (MW2)	CC1	NEG	NEG	POS	POS	POS	POS	NEG	POS	POS	POS	NEG	NEG	NEG	NEG	POS	POS	NEG	NEG	POS	POS	POS	NEG	POS	AMB	POS	POS
**UKD-1031910**	SCCmec IVa (MW2)	CC1	NEG	NEG	POS	POS	POS	POS	NEG	POS	POS	POS	NEG	AMB	NEG	NEG	POS	POS	NEG	NEG	POS	POS	POS	NEG	POS	AMB	POS	POS
**UKD-14571328**	SCCmec IVa (MW2)	CC1	NEG	NEG	POS	POS	POS	POS	NEG	POS	POS	POS	NEG	AMB	NEG	NEG	POS	POS	NEG	NEG	POS	POS	POS	NEG	AMB	POS	POS	POS
**UKD-03003012**	SCCmec IVa (MW2)	CC5	NEG	POS	NEG	POS	POS	POS	NEG	POS	POS	NEG	NEG	POS	NEG	NEG	POS	POS	NEG	NEG	POS	POS	POS	POS	NEG	NEG	NEG	POS
**UKD-15582582**	SCCmec IVa (MW2)	CC6	NEG	NEG	POS	POS	POS	POS	NEG	POS	POS	POS	NEG	AMB	NEG	AMB	POS	POS	NEG	NEG	POS	POS	POS	NEG	NEG	POS	POS	POS
**UKD-16574230**	SCCmec IVa (MW2)	CC6	NEG	NEG	POS	POS	POS	POS	NEG	POS	POS	POS	NEG	AMB	NEG	NEG	POS	POS	NEG	NEG	POS	POS	POS	NEG	NEG	POS	POS	POS
**UKD-16582560**	SCCmec IVa (MW2)	CC6	NEG	NEG	POS	POS	POS	POS	NEG	POS	POS	POS	NEG	NEG	NEG	NEG	POS	POS	NEG	NEG	POS	POS	POS	NEG	NEG	NEG	POS	POS
**UKD-0802855**	SCCmec IVa (MW2)	CC7	NEG	NEG	POS	POS	POS	POS	NEG	POS	POS	POS	POS	NEG	NEG	NEG	POS	POS	POS	NEG	POS	POS	POS	NEG	NEG	NEG	NEG	POS
**KHDN-10405258**	SCCmec IVa (MW2)	CC8	NEG	POS	NEG	POS	POS	POS	NEG	POS	POS	POS	NEG	AMB	NEG	NEG	POS	POS	POS	AMB	POS	POS	POS	POS	NEG	NEG	NEG	POS
**UKD-15573497**	SCCmec IVa (MW2)	CC22	NEG	POS	NEG	POS	POS	POS	NEG	POS	POS	NEG	NEG	POS	NEG	AMB	POS	POS	NEG	NEG	POS	POS	POS	NEG	NEG	POS	POS	NEG
**UKD-0634729**	SCCmec IVa (MW2)	CC30	NEG	NEG	POS	POS	POS	POS	NEG	POS	POS	AMB	POS	NEG	NEG	NEG	POS	POS	POS	POS	AMB	POS	POS	NEG	NEG	POS	POS	POS
**UKD-1119771**	SCCmec IVa (MW2)	CC30	NEG	NEG	POS	POS	POS	POS	NEG	POS	POS	AMB	POS	NEG	NEG	NEG	POS	POS	POS	AMB	NEG	POS	POS	NEG	NEG	POS	POS	POS
**KHDN-10411458**	SCCmec IVa (MW2)	CC30	NEG	NEG	POS	POS	POS	POS	NEG	POS	POS	AMB	POS	NEG	NEG	NEG	POS	POS	AMB	POS	NEG	POS	POS	NEG	NEG	POS	POS	POS
**KHDN-398609**	SCCmec IVa (MW2)	CC30	NEG	NEG	POS	POS	POS	POS	NEG	POS	POS	AMB	POS	NEG	NEG	NEG	POS	POS	POS	AMB	AMB	POS	POS	NEG	NEG	POS	POS	POS
**KHDN-01081998**	SCCmec IVa (MW2)	CC30	NEG	NEG	POS	POS	POS	POS	NEG	POS	POS	AMB	POS	NEG	NEG	NEG	POS	POS	POS	AMB	NEG	POS	POS	NEG	NEG	POS	POS	POS
**KHDN-10399223**	SCCmec IVa (MW2)	CC30	NEG	NEG	POS	POS	POS	POS	NEG	POS	POS	NEG	POS	NEG	NEG	NEG	POS	POS	POS	AMB	NEG	POS	POS	NEG	NEG	POS	POS	POS
**KHDN-W261158**	SCCmec IVa (MW2)	CC30	NEG	NEG	POS	POS	POS	POS	NEG	POS	POS	NEG	POS	NEG	NEG	NEG	POS	POS	POS	AMB	NEG	POS	POS	NEG	NEG	POS	POS	POS
**UKD-0002046**	SCCmec IVa (MW2)	CC45 [agr I]	NEG	NEG	POS	POS	POS	POS	NEG	POS	POS	NEG	NEG	NEG	NEG	POS	POS	POS	AMB	NEG	POS	POS	POS	NEG	AMB	POS	POS	POS
**UKD-0029618**	SCCmec IVa (MW2)	CC45 [agr I]	NEG	NEG	POS	POS	POS	POS	NEG	POS	POS	NEG	NEG	NEG	NEG	POS	POS	POS	AMB	NEG	POS	POS	POS	NEG	POS	AMB	POS	POS
**UKD-0043085**	SCCmec IVa (MW2)	CC45 [agr I]	NEG	NEG	POS	POS	POS	POS	NEG	POS	POS	NEG	NEG	NEG	NEG	POS	POS	POS	AMB	NEG	POS	POS	POS	NEG	POS	AMB	POS	POS
**UKD-0047053**	SCCmec IVa (MW2)	CC45 [agr I]	NEG	NEG	POS	POS	POS	POS	NEG	POS	POS	NEG	NEG	NEG	NEG	POS	POS	POS	NEG	NEG	POS	POS	POS	NEG	POS	POS	POS	POS
**UKD-0047429**	SCCmec IVa (MW2)	CC45 [agr I]	NEG	NEG	POS	POS	POS	POS	NEG	POS	POS	NEG	NEG	NEG	NEG	POS	POS	POS	AMB	NEG	POS	POS	POS	NEG	POS	AMB	POS	POS
**UKD-0112465**	SCCmec IVa (MW2)	CC45 [agr I]	NEG	NEG	POS	POS	POS	POS	NEG	POS	POS	NEG	NEG	NEG	NEG	POS	POS	POS	AMB	NEG	POS	POS	POS	NEG	POS	AMB	POS	POS
**UKD-0103483**	SCCmec IVa (MW2)	CC45 [agr I]	NEG	NEG	POS	POS	POS	POS	NEG	POS	POS	NEG	NEG	NEG	NEG	POS	POS	POS	NEG	NEG	POS	POS	POS	NEG	POS	AMB	POS	POS
**UKD-0305014**	SCCmec IVa (MW2)	CC45 [agr I]	NEG	NEG	POS	POS	POS	POS	NEG	POS	POS	NEG	NEG	NEG	NEG	POS	POS	POS	AMB	NEG	POS	POS	POS	NEG	POS	AMB	POS	POS
**UKD-0401119**	SCCmec IVa (MW2)	CC45 [agr I]	NEG	NEG	POS	POS	POS	POS	NEG	POS	POS	NEG	NEG	NEG	NEG	POS	POS	POS	AMB	NEG	POS	POS	POS	NEG	POS	AMB	POS	POS
**UKD-0537969**	SCCmec IVa (MW2)	CC45 [agr I]	NEG	NEG	POS	POS	POS	POS	NEG	POS	POS	NEG	NEG	NEG	NEG	POS	POS	POS	AMB	NEG	POS	POS	POS	NEG	POS	AMB	POS	POS
**UKD-0703373**	SCCmec IVa (MW2)	CC45 [agr I]	NEG	NEG	POS	POS	POS	POS	NEG	POS	POS	NEG	NEG	NEG	NEG	POS	POS	POS	NEG	NEG	POS	POS	POS	NEG	POS	POS	POS	POS
**UKD-0813653**	SCCmec IVa (MW2)	CC45 [agr I]	NEG	NEG	POS	POS	POS	POS	NEG	POS	POS	NEG	NEG	NEG	NEG	POS	POS	POS	AMB	NEG	POS	POS	POS	NEG	POS	AMB	POS	POS
**UKD-0839175**	SCCmec IVa (MW2)	CC45 [agr I]	NEG	NEG	POS	POS	POS	POS	NEG	POS	POS	AMB	NEG	AMB	NEG	POS	POS	POS	POS	AMB	AMB	POS	POS	NEG	POS	AMB	POS	POS
**UKD-1108422**	SCCmec IVa (MW2)	CC45 [agr I]	NEG	NEG	POS	POS	POS	POS	NEG	POS	POS	AMB	NEG	AMB	AMB	POS	POS	POS	POS	NEG	AMB	POS	POS	NEG	POS	AMB	POS	POS
**UKD-1380645**	SCCmec IVa (MW2)	CC45 [agr I]	NEG	NEG	POS	POS	POS	POS	NEG	POS	POS	NEG	NEG	NEG	NEG	POS	POS	POS	AMB	NEG	POS	POS	POS	NEG	POS	AMB	POS	POS
**UKD-148016**	SCCmec IVa (MW2)	CC45 [agr I]	NEG	NEG	POS	POS	POS	POS	NEG	POS	POS	AMB	NEG	AMB	AMB	POS	POS	POS	POS	AMB	AMB	POS	POS	NEG	POS	AMB	POS	POS
**UKD-16571773**	SCCmec IVa (MW2)	CC45 [agr I]	NEG	NEG	POS	POS	POS	POS	NEG	POS	POS	AMB	NEG	AMB	NEG	POS	POS	POS	AMB	NEG	POS	POS	POS	NEG	POS	AMB	POS	POS
**KHDN-10407738**	SCCmec IVa (MW2)	CC45 [ST617]	NEG	NEG	POS	POS	POS	POS	NEG	POS	POS	NEG	NEG	AMB	NEG	POS	POS	POS	AMB	NEG	POS	POS	POS	POS	NEG	NEG	NEG	POS
**UKD-0853670**	SCCmec IVa (MW2)	CC88	NEG	NEG	POS	POS	POS	POS	NEG	POS	POS	NEG	NEG	POS	NEG	NEG	POS	POS	NEG	NEG	POS	POS	POS	NEG	NEG	NEG	NEG	POS
**UKD-1417352**	SCCmec IVa (MW2)	CC88	NEG	NEG	POS	POS	POS	POS	NEG	POS	POS	NEG	NEG	POS	NEG	NEG	POS	POS	NEG	NEG	POS	POS	POS	NEG	NEG	POS	NEG	POS
**UKD-15571283**	SCCmec IVa (MW2)	CC88	NEG	NEG	POS	POS	POS	POS	NEG	POS	POS	AMB	NEG	POS	NEG	NEG	POS	POS	NEG	AMB	POS	POS	POS	NEG	AMB	POS	NEG	POS
**KHDN-10406719**	SCCmec IVa (MW2)	ST93	NEG	NEG	POS	POS	POS	POS	NEG	POS	POS	NEG	NEG	POS	NEG	NEG	POS	POS	NEG	NEG	POS	POS	POS	NEG	NEG	POS	NEG	POS
**KHDN-0060294**	SCCmec IVa (MW2)	ST93	NEG	NEG	POS	POS	POS	POS	NEG	POS	POS	NEG	NEG	POS	NEG	NEG	POS	POS	NEG	NEG	POS	POS	POS	NEG	NEG	POS	NEG	NEG
**UKD-0734190**	SCCmec IVa (MW2)	CC97	NEG	POS	NEG	POS	POS	POS	NEG	NEG	NEG	NEG	NEG	NEG	NEG	NEG	POS	POS	POS	NEG	POS	NEG	AMB	NEG	NEG	NEG	NEG	POS
**UKD-13006983**	SCCmec IVa (MW2)	CC97	NEG	POS	NEG	POS	POS	POS	NEG	NEG	NEG	NEG	NEG	NEG	NEG	NEG	POS	POS	AMB	POS	AMB	POS	POS	NEG	NEG	POS	NEG	POS
Prediction for H131520133	***SCCmec IVa (H131520133)***	***CC152***	***NEG***	***POS***	***NEG***	***POS***	***NEG***	***POS***	***NEG***	***POS***	***POS***	***POS***	***NEG***	***NEG***	***NEG***	***NEG***	***POS***	***POS***	***POS***	***NEG***	***AMB***	***POS***	***POS***	***NEG***	***AMB***	***AMB***	***POS***	***POS***
**KHDN-325892**	SCCmec IVa (H131520133)	CC30	NEG	NEG	POS	POS	POS	POS	NEG	POS	POS	AMB	POS	NEG	NEG	NEG	POS	POS	POS	AMB	NEG	POS	POS	NEG	NEG	NEG	POS	POS
**UKD-15580207**	SCCmec IVa (H131520133)	CC88	NEG	NEG	POS	POS	POS	POS	NEG	POS	POS	AMB	NEG	POS	NEG	NEG	POS	POS	NEG	NEG	POS	POS	POS	NEG	NEG	POS	NEG	POS
**UKD-0521159**	SCCmec Iva	CC8	NEG	POS	NEG	POS	POS	POS	NEG	POS	POS	POS	NEG	NEG	NEG	NEG	POS	POS	POS	POS	POS	POS	POS	POS	NEG	NEG	NEG	POS
Prediction for CMFT503 : HF569113.1	***SCCmec IVa (CMFT503)***		***NEG***																									
Prediction for 21343, GenBank AHKV	***SCCmec IVa (CMFT503)***	***CC88***	***NEG***	***NEG***	***POS***	***POS***	***POS***	***POS***	***NEG***	***POS***	***POS***	***NEG***	***NEG***	***POS***	***NEG***	***NEG***	***POS***	***POS***	***NEG***	***NEG***	***POS***	***POS***	***POS***	***NEG***	***NEG***	***NEG***	***NEG***	***POS***
**UKD-1420740**	SCCmec IVa (CMFT503)	CC22	NEG	POS	NEG	POS	POS	POS	NEG	POS	POS	NEG	NEG	POS	AMB	AMB	POS	POS	NEG	AMB	POS	POS	POS	NEG	AMB	POS	POS	NEG
**UKD-15575124**	SCCmec IVa (CMFT503)	CC22	NEG	POS	NEG	POS	POS	POS	NEG	POS	POS	NEG	NEG	POS	NEG	NEG	POS	POS	NEG	NEG	POS	POS	POS	NEG	NEG	POS	POS	NEG
**UKD-16584947**	SCCmec IVa (CMFT503)	CC22	NEG	POS	NEG	POS	POS	POS	NEG	POS	POS	NEG	NEG	NEG	NEG	NEG	POS	POS	NEG	NEG	POS	POS	POS	NEG	NEG	POS	POS	NEG
**UKD-16575077**	SCCmec IVa (CMFT503)	CC88	NEG	NEG	POS	POS	POS	POS	NEG	POS	POS	NEG	NEG	POS	NEG	NEG	POS	POS	NEG	NEG	POS	POS	POS	NEG	NEG	POS	NEG	POS
Prediction for TCH1516, GenBank CP000730.1	***SCCmec IVa+ACME1+Cu (USA300)***	***CC8***	***NEG***	***POS***	***NEG***	***POS***	***POS***	***POS***	***NEG***	***POS***	***POS***	***POS***	***NEG***	***NEG***	***NEG***	***NEG***	***POS***	***POS***	***POS***	***NEG***	***AMB***	***POS***	***POS***	***POS***	***NEG***	***NEG***	***NEG***	***POS***
**Reference strain USA300-TCH1516**	SCCmec IVa+ACME1+Cu (USA300)	CC8	NEG	POS	NEG	POS	POS	POS	NEG	POS	POS	POS	NEG	NEG	NEG	NEG	POS	POS	POS	AMB	AMB	POS	POS	POS	NEG	NEG	NEG	POS
Prediction for FPR3757, GenBank CP000255.1	***SCCmec IVa+ACME1+Cu (USA300)***	***CC8***	***NEG***	***POS***	***NEG***	***POS***	***POS***	***POS***	***NEG***	***POS***	***POS***	***POS***	***NEG***	***NEG***	***NEG***	***NEG***	***POS***	***POS***	***POS***	***NEG***	***AMB***	***POS***	***POS***	***POS***	***NEG***	***NEG***	***NEG***	***POS***
**UKD-0516386**	SCCmec IVa+ACME1+Cu (USA300)	CC8	NEG	POS	NEG	POS	POS	POS	NEG	POS	POS	POS	NEG	NEG	NEG	NEG	POS	POS	POS	POS	POS	POS	POS	POS	NEG	NEG	NEG	POS
**UKD-0815773**	SCCmec IVa+ACME1+Cu (USA300)	CC8	NEG	POS	NEG	POS	POS	POS	NEG	POS	POS	POS	NEG	NEG	NEG	NEG	POS	POS	POS	AMB	AMB	POS	POS	POS	NEG	NEG	NEG	POS
**UKD-1414654**	SCCmec IVa+ACME1+Cu (USA300)	CC8	NEG	POS	NEG	POS	POS	POS	NEG	POS	POS	POS	NEG	AMB	NEG	NEG	POS	POS	AMB	AMB	POS	POS	POS	POS	NEG	NEG	NEG	POS
**UKD-0408036**	SCCmec IVa+ACME2	CC45 [agr I]	NEG	NEG	POS	POS	POS	POS	NEG	POS	POS	AMB	NEG	NEG	NEG	POS	POS	POS	POS	AMB	AMB	POS	POS	NEG	POS	AMB	POS	POS
**UKD-0044035**	SCCmec IVa+ccrC (Hannover)	CC8	NEG	POS	NEG	POS	POS	POS	NEG	NEG	NEG	NEG	NEG	NEG	NEG	NEG	POS	POS	POS	AMB	POS	POS	POS	POS	NEG	NEG	NEG	POS
**UKD-0136287**	SCCmec IVa+ccrC (Hannover)	CC8	NEG	POS	NEG	POS	POS	POS	NEG	NEG	NEG	NEG	NEG	NEG	NEG	NEG	POS	POS	POS	AMB	AMB	POS	POS	POS	NEG	NEG	NEG	POS

Isolate ID	SCCmec type	Clonal complex	CAPSULE-ASSOCIATED GENES			BIOFILM-ASSOCIATED GENES				ADHESION FACTORS / GENES ENCODING MICROBIAL SURFACE COMPONENTS RECOGNIZING ADHESIVE MATRIX MOLECULES (MSCRAMM GENES)																		
			Capsule type 1	Capsule type 5	Capsule type 8	intercellular adhesion protein A	intercellular adhesion protein C	biofilm PIA synthesis protein D	surface protein involved in biofilm formation	bone sialoprotein-binding protein							clumping factor A					clumping factor B					collagen-binding adhesin	cell wall associated fibronectin-binding protein
			cap 1	cap 5	cap 8	icaA	icaC	icaD	bap	bbp	bbp (cons)	bbp (COL+MW2)	bbp (MRSA252)	bbp (Mu50)	bbp (RF122)	bbp (ST45)	clfA	clfA (cons)	clfA (COL+RF122)	clfA (MRSA252)	clfA (Mu50+MW2)	clfB	clfB (cons)	clfB (COL+Mu50)	clfB (MW2)	clfB (RF122)	cna	ebh (cons)
Prediction for JCSC1978, GenBank AB063173.1	SCCmec IVb/d/i (JCSC1978/6668/4469)		NEG																									
Reference strain JCSC1978 (8/6-3P)	SCCmec IVb/d/i (JCSC1978/6668/4469)	CC8	NEG	POS	NEG	POS	POS	POS	NEG	POS	POS	POS	NEG	AMB	NEG	NEG	POS	POS	POS	AMB	AMB	POS	POS	POS	NEG	NEG	NEG	POS
Prediction for JCSC6668, GenBank AB425823.1	SCCmec IVb/d/i (JCSC1978/6668/4469)		NEG																									
Reference strain JCSC4469	SCCmec IVb/d/i (JCSC1978/6668/4469)	CC5	NEG	POS	NEG	POS	POS	POS	NEG	POS	POS	AMB	NEG	POS	NEG	NEG	POS	POS	NEG	NEG	POS	POS	POS	POS	NEG	NEG	NEG	POS
UKD-1103429	SCCmec IVb/d/i (JCSC1978/6668/4469)	CC7	NEG	NEG	POS	POS	POS	POS	NEG	POS	POS	POS	AMB	NEG	NEG	NEG	POS	POS	POS	AMB	AMB	POS	POS	NEG	NEG	POS	NEG	POS
UKD-1306141	SCCmec IVb/d/i (JCSC1978/6668/4469)	CC7	NEG	NEG	POS	POS	POS	POS	NEG	POS	POS	POS	AMB	NEG	NEG	NEG	POS	POS	AMB	POS	AMB	POS	POS	NEG	NEG	POS	NEG	POS
UKD-0500089	SCCmec IVb/d/i (JCSC1978/6668/4469)	CC5	NEG	POS	NEG	POS	POS	POS	NEG	POS	POS	NEG	NEG	POS	NEG	NEG	POS	POS	NEG	NEG	POS	POS	POS	POS	NEG	NEG	NEG	POS
UKD-0501416	SCCmec IVb/d/i (JCSC1978/6668/4469)	CC5	NEG	POS	NEG	POS	POS	POS	NEG	POS	AMB	NEG	NEG	POS	NEG	NEG	POS	POS	NEG	NEG	POS	POS	POS	POS	NEG	NEG	NEG	POS
UKD-0934574	SCCmec IVb/d/i (JCSC1978/6668/4469)	CC5	NEG	POS	NEG	POS	POS	POS	NEG	POS	POS	NEG	NEG	POS	NEG	NEG	POS	POS	NEG	NEG	POS	POS	POS	POS	NEG	NEG	NEG	POS
UKD-0746779	SCCmec IVb/d/i (JCSC1978/6668/4469)	CC45 [agr I]	NEG	NEG	POS	POS	POS	POS	NEG	POS	POS	AMB	NEG	NEG	NEG	POS	POS	POS	POS	NEG	POS	POS	POS	NEG	POS	POS	POS	POS
UKD-0812518	SCCmec IVb/d/i (JCSC1978/6668/4469)	CC45 [agr I]	NEG	NEG	POS	POS	POS	POS	NEG	POS	POS	NEG	NEG	NEG	NEG	POS	POS	POS	NEG	NEG	POS	POS	POS	NEG	POS	AMB	POS	POS
Prediction for 21209, GenBank AGRP	SCCmec IVb/d/i (21209)	CC8	NEG	POS	NEG	POS	POS	POS	NEG	POS	POS	POS	NEG	NEG	NEG	NEG	POS	POS	POS	NEG	AMB	POS	POS	POS	NEG	NEG	NEG	POS
UKD-0233579	SCCmec IVb/d/i (21209)	CC8	NEG	POS	NEG	POS	POS	POS	NEG	POS	POS	POS	NEG	POS	POS	AMB	POS	POS	POS	POS	POS	POS	POS	POS	NEG	NEG	NEG	POS
UKD-0504920	SCCmec IVb/d/i (21209)	CC8	NEG	POS	NEG	POS	POS	POS	NEG	POS	POS	POS	NEG	NEG	NEG	NEG	POS	POS	POS	AMB	POS	POS	POS	POS	NEG	NEG	NEG	POS
UKD-0725999	SCCmecIV b/d/i+Cu	CC30	NEG	NEG	POS	POS	POS	POS	NEG	POS	POS	NEG	POS	NEG	NEG	NEG	POS	POS	POS	POS	NEG	POS	POS	NEG	NEG	AMB	POS	POS
Prediction for IS-105, GenBank AHLR	SCCmec IVc (IS-105)	CC22	NEG	POS	NEG	POS	POS	POS	NEG	POS	POS	NEG	NEG	NEG	NEG	NEG	POS	POS	NEG	NEG	POS	POS	POS	NEG	NEG	NEG	POS	NEG
UKD-0402421	SCCmec IVc (IS-105), merA/B+	CC5	NEG	POS	NEG	POS	POS	POS	NEG	POS	POS	NEG	NEG	POS	NEG	NEG	POS	POS	NEG	NEG	POS	POS	POS	POS	NEG	NEG	NEG	POS
UKD-0731319	SCCmec IVc (IS-105), merA/B+	CC5	NEG	POS	NEG	POS	POS	POS	NEG	POS	POS	NEG	NEG	POS	NEG	NEG	POS	POS	NEG	NEG	POS	POS	POS	POS	NEG	NEG	NEG	POS
UKD-0733137	SCCmec IVc (IS-105)	CC8	NEG	POS	NEG	POS	POS	POS	NEG	POS	POS	POS	NEG	NEG	NEG	NEG	POS	POS	POS	POS	POS	POS	POS	POS	NEG	NEG	NEG	POS
UKD-0305690	SCCmec IVc (IS-105)	CC22	NEG	POS	NEG	POS	POS	POS	NEG	POS	POS	NEG	NEG	NEG	NEG	NEG	POS	POS	NEG	NEG	POS	POS	POS	NEG	NEG	NEG	POS	NEG
UKD-1424706	SCCmec IVc (IS-105)	CC22	NEG	POS	NEG	POS	POS	POS	NEG	POS	POS	NEG	NEG	POS	NEG	AMB	POS	POS	NEG	NEG	POS	POS	POS	NEG	NEG	POS	POS	NEG
Prediction for TCH60, GenBank CP002110.1	SCCmec IVc (TCH60)	CC30	NEG	NEG	POS	POS	POS	POS	NEG	POS	POS	NEG	POS	NEG	NEG	NEG	POS	POS	AMB	POS	NEG	POS	POS	NEG	NEG	NEG	POS	POS
Reference strain JCSC4788	SCCmec IVc (TCH60)	CC8	NEG	POS	NEG	POS	POS	POS	NEG	NEG	NEG	NEG	NEG	NEG	NEG	NEG	POS	POS	POS	AMB	AMB	POS	POS	POS	NEG	NEG	NEG	POS
UKD-1318318	SCCmec IVc (TCH60)	CC5	NEG	POS	NEG	POS	POS	POS	NEG	POS	POS	NEG	NEG	POS	NEG	NEG	POS	POS	NEG	NEG	POS	POS	POS	POS	NEG	NEG	NEG	POS
UKD-14580050	SCCmec IVc (TCH60)	CC5	NEG	POS	NEG	POS	POS	POS	NEG	POS	POS	AMB	NEG	POS	NEG	NEG	POS	POS	NEG	NEG	POS	POS	POS	POS	NEG	NEG	NEG	POS
UKD-08F01314	SCCmec IVc (TCH60)	CC8	NEG	POS	NEG	POS	POS	POS	NEG	POS	POS	POS	NEG	NEG	NEG	NEG	NEG	NEG	NEG	NEG	NEG	POS	POS	POS	NEG	NEG	NEG	POS
KHDN-50361518	SCCmec IVc (TCH60)	CC8	NEG	POS	NEG	POS	POS	POS	NEG	POS	POS	POS	NEG	AMB	NEG	NEG	POS	POS	AMB	AMB	POS	POS	POS	POS	NEG	NEG	NEG	POS
UKD-0307912	SCCmec IVc (TCH60)	CC22	NEG	POS	NEG	POS	POS	POS	NEG	POS	POS	NEG	NEG	NEG	NEG	NEG	POS	POS	NEG	NEG	POS	POS	POS	NEG	NEG	POS	POS	NEG
UKD-0220390	SCCmec IVc (TCH60)	CC45 [agr I]	NEG	NEG	POS	POS	POS	POS	NEG	POS	POS	NEG	NEG	NEG	NEG	POS	POS	POS	NEG	NEG	POS	POS	POS	NEG	POS	AMB	POS	POS
UKD-0311106	SCCmec IVc (TCH60)	CC45 [agr I]	NEG	NEG	POS	POS	POS	POS	NEG	POS	NEG	NEG	NEG	NEG	NEG	POS	POS	POS	POS	NEG	AMB	POS	POS	NEG	POS	AMB	POS	POS
UKD-0719855	SCCmec IVc (TCH60)	CC45 [agr I]	NEG	NEG	POS	POS	POS	POS	NEG	POS	POS	NEG	NEG	NEG	NEG	POS	POS	POS	NEG	NEG	POS	POS	POS	NEG	POS	POS	POS	POS
UKD-0422079	SCCmec IVc (TCH60)	CC45 [agr I]	NEG	NEG	POS	POS	POS	POS	NEG	POS	POS	NEG	NEG	NEG	NEG	POS	POS	POS	AMB	NEG	POS	POS	POS	NEG	POS	AMB	POS	POS
UKD-0409235	SCCmec IVc (TCH60)	CC80	NEG	NEG	POS	POS	POS	POS	NEG	POS	POS	NEG	NEG	POS	NEG	NEG	POS	POS	NEG	NEG	POS	POS	POS	POS	NEG	NEG	NEG	POS
UKD-0409807	SCCmec IVc (TCH60)	CC80	NEG	NEG	POS	POS	POS	POS	NEG	POS	POS	AMB	NEG	POS	NEG	NEG	POS	POS	NEG	NEG	POS	POS	POS	POS	NEG	NEG	NEG	POS
UKD-0416073	SCCmec IVc (TCH60)	CC80	NEG	NEG	POS	POS	POS	POS	NEG	POS	POS	NEG	NEG	POS	NEG	NEG	POS	POS	NEG	NEG	POS	POS	POS	POS	NEG	NEG	NEG	POS
UKD-0431728	SCCmec IVc (TCH60)	CC80	NEG	NEG	POS	POS	POS	POS	NEG	POS	POS	NEG	NEG	POS	NEG	NEG	POS	POS	NEG	NEG	POS	POS	POS	POS	NEG	NEG	NEG	POS
UKD-1001508	SCCmec IVc (TCH60)	CC80	NEG	NEG	POS	POS	POS	POS	NEG	POS	POS	AMB	NEG	POS	NEG	NEG	POS	POS	NEG	NEG	POS	POS	POS	POS	NEG	NEG	NEG	POS
UKD-1010412	SCCmec IVc (TCH60)	CC80	NEG	NEG	POS	AMB	AMB	AMB	NEG	POS	POS	AMB	NEG	POS	NEG	AMB	POS	POS	NEG	NEG	POS	POS	POS	POS	NEG	NEG	NEG	POS
UKD-12753005	SCCmec IVc (TCH60)	CC80	NEG	NEG	POS	POS	POS	POS	NEG	POS	POS	NEG	NEG	POS	NEG	NEG	POS	POS	NEG	NEG	POS	POS	POS	POS	NEG	NEG	NEG	POS
UKD-15574809	SCCmec IVc (TCH60)	CC80	NEG	NEG	POS	POS	POS	POS	NEG	POS	POS	NEG	NEG	POS	NEG	NEG	POS	POS	NEG	NEG	POS	POS	POS	POS	NEG	NEG	NEG	POS
KHDN-16120291	SCCmec IVc (TCH60)	CC88	NEG	NEG	POS	POS	POS	POS	NEG	POS	POS	NEG	NEG	POS	NEG	NEG	POS	POS	NEG	NEG	POS	POS	POS	NEG	NEG	NEG	NEG	POS
Prediction for WW2703/97, GenBank ACSW	SCCmec IVc (WW2703/97)	CC30	NEG	NEG	POS	POS	POS	POS	NEG	POS	POS	NEG	POS	NEG	NEG	NEG	POS	POS	AMB	POS	NEG	POS	POS	NEG	NEG	NEG	POS	POS
UKD-14580088	SCCmec IVc (WW2703/97)	CC398	NEG	POS	NEG	POS	POS	POS	NEG	POS	POS	NEG	NEG	NEG	NEG	POS	POS	POS	POS	AMB	AMB	POS	POS	NEG	POS	AMB	POS	POS
Prediction for SA_ST125_MupR, GenBank ASTH	SCCmec IVc+speG+ccrAB4 (SA_ST125)	CC5	NEG	POS	NEG	POS	POS	POS	NEG	POS	POS	NEG	NEG	POS	NEG	NEG	POS	POS	NEG	NEG	POS	POS	POS	POS	NEG	NEG	NEG	POS
UKD-0439909	SCCmec IVc+speG+ccrAB4 (SA_ST125)	CC5	NEG	POS	NEG	POS	POS	POS	NEG	NEG	NEG	NEG	NEG	NEG	NEG	NEG	POS	POS	NEG	NEG	POS	POS	POS	POS	NEG	NEG	NEG	POS
Prediction for MRSA177, GenBank AECP	SCCmec IVc+Cu/Hg (MRSA177)		NEG	POS	NEG	POS	POS	POS	NEG	NEG	NEG	NEG	NEG	NEG	NEG	NEG	POS	POS	POS	NEG	AMB	POS	POS	POS	NEG	NEG	NEG	POS
UKD-1279734	SCCmec IVc+Cu/Hg (MRSA177)	CC8	NEG	POS	NEG	POS	POS	POS	NEG	POS	POS	POS	NEG	AMB	NEG	NEG	POS	POS	AMB	POS	AMB	POS	POS	POS	NEG	NEG	NEG	POS
UKD-14580899	SCCmec IVc+Cu/Hg (MRSA177)	CC8	NEG	POS	NEG	POS	POS	POS	NEG	POS	POS	POS	NEG	AMB	NEG	NEG	POS	POS	AMB	POS	AMB	POS	POS	POS	NEG	NEG	NEG	POS
UKD-16002228	SCCmec IVc+Cu/Hg (MRSA177)	CC8	NEG	POS	NEG	POS	POS	POS	NEG	NEG	NEG	NEG	NEG	NEG	NEG	NEG	POS	POS	AMB	AMB	POS	POS	POS	POS	NEG	NEG	NEG	POS
Prediction for HO 5096 0412, GenBank HE681097.1	SCCmec IVh/j (HO50960412/JCSC6670)	CC22	NEG	POS	NEG	POS	POS	POS	NEG	POS	POS	NEG	NEG	NEG	NEG	NEG	POS	POS	NEG	NEG	POS	POS	POS	NEG	NEG	NEG	POS	NEG
Prediction for JCSC6670, GenBank AB425824.1	SCCmec IVh/j (HO50960412/JCSC6670)		NEG																									
Reference strain Dublin_DSH_E1749	SCCmec IVh/j (HO50960412/JCSC6670)		NEG	POS	NEG	POS	POS	POS	NEG	POS	POS	NEG	NEG	POS	NEG	NEG	POS	POS	NEG	NEG	POS	POS	POS	NEG	NEG	POS	POS	NEG
UKD-14576248	SCCmec IVh/j (HO50960412/JCSC6670)	CC8	NEG	POS	NEG	POS	POS	POS	NEG	POS	POS	POS	NEG	AMB	NEG	NEG	POS	POS	POS	AMB	AMB	POS	POS	POS	NEG	NEG	NEG	POS
UKD-0120122	SCCmec IVh/j (HO50960412/JCSC6670)	CC22	NEG	POS	NEG	POS	POS	POS	NEG	POS	POS	NEG	NEG	NEG	NEG	NEG	POS	POS	NEG	NEG	POS	POS	POS	NEG	NEG	NEG	POS	NEG
UKD-0205319	SCCmec IVh/j (HO50960412/JCSC6670)	CC22	NEG	POS	NEG	POS	POS	POS	NEG	POS	POS	NEG	NEG	NEG	NEG	NEG	POS	POS	NEG	NEG	POS	POS	POS	NEG	NEG	POS	POS	NEG
UKD-0221259	SCCmec IVh/j (HO50960412/JCSC6670)	CC22	NEG	POS	NEG	POS	POS	POS	NEG	POS	POS	NEG	NEG	NEG	NEG	NEG	POS	POS	NEG	NEG	POS	POS	POS	NEG	NEG	POS	POS	NEG
UKD-0418654	SCCmec IVh/j (HO50960412/JCSC6670)	CC22	NEG	POS	NEG	POS	POS	POS	NEG	POS	POS	NEG	NEG	NEG	NEG	NEG	POS	POS	NEG	NEG	POS	POS	POS	NEG	NEG	POS	POS	NEG
UKD-0429721	SCCmec IVh/j (HO50960412/JCSC6670)	CC22	NEG	POS	NEG	POS	AMB	POS	NEG	POS	POS	NEG	NEG	NEG	NEG	NEG	POS	POS	NEG	NEG	POS	POS	POS	NEG	NEG	NEG	POS	NEG
UKD-1116194	SCCmec IVh/j (HO50960412/JCSC6670)	CC22	NEG	POS	NEG	POS	POS	POS	NEG	NEG	NEG	NEG	NEG	NEG	NEG	NEG	POS	POS	NEG	NEG	POS	POS	POS	NEG	NEG	POS	POS	NEG
UKD-1122367	SCCmec IVh/j (HO50960412/JCSC6670)	CC22	NEG	POS	NEG	POS	POS	POS	NEG	POS	POS	NEG	NEG	POS	NEG	NEG	POS	POS	NEG	NEG	POS	POS	POS	NEG	NEG	POS	POS	NEG
UKD-14012840	SCCmec IVh/j (HO50960412/JCSC6670)	CC22	NEG	POS	NEG	POS	POS	POS	NEG	POS	POS	NEG	NEG	POS	NEG	NEG	POS	POS	NEG	NEG	POS	POS	POS	NEG	NEG	POS	POS	NEG
UKD-14579833	SCCmec IVh/j (HO50960412/JCSC6670)	CC22	NEG	POS	NEG	POS	POS	POS	NEG	POS	POS	NEG	NEG	POS	NEG	NEG	POS	POS	NEG	NEG	POS	POS	POS	NEG	NEG	POS	POS	NEG
UKD-15579198	SCCmec IVh/j (HO50960412/JCSC6670)	CC22	NEG	POS	NEG	POS	POS	POS	NEG	POS	POS	NEG	NEG	POS	AMB	AMB	POS	POS	NEG	NEG	POS	POS	POS	NEG	NEG	POS	POS	NEG
UKD-4052193	SCCmec IVh/j (HO50960412/JCSC6670)	CC22	NEG	POS	NEG	POS	POS	POS	NEG	POS	POS	NEG	NEG	POS	NEG	NEG	POS	POS	NEG	NEG	POS	POS	POS	NEG	NEG	POS	POS	NEG
UKD-124968	SCCmec IVh/j (HO50960412/JCSC6670)	CC22	NEG	POS	NEG	POS	POS	POS	NEG	POS	POS	NEG	NEG	POS	NEG	NEG	POS	POS	NEG	NEG	POS	POS	POS	NEG	NEG	POS	POS	NEG
UKD-14572546	SCCmec IVh/j (HO50960412/JCSC6670)	CC22	NEG	POS	NEG	POS	POS	POS	NEG	POS	POS	NEG	NEG	POS	AMB	AMB	POS	POS	NEG	AMB	POS	POS	POS	NEG	AMB	POS	POS	NEG
UKD-14582287	SCCmec IVh/j (HO50960412/JCSC6670)	CC22	NEG	POS	NEG	POS	POS	POS	NEG	POS	POS	NEG	NEG	POS	AMB	AMB	POS	POS	NEG	AMB	POS	POS	POS	AMB	AMB	POS	POS	NEG
UKD-14583376	SCCmec IVh/j (HO50960412/JCSC6670)	CC22	NEG	POS	NEG	POS	POS	POS	NEG	POS	POS	NEG	NEG	POS	AMB	NEG	POS	POS	NEG	AMB	POS	POS	POS	NEG	AMB	POS	POS	NEG
UKD-14583496	SCCmec IVh/j (HO50960412/JCSC6670)	CC22	NEG	POS	NEG	POS	POS	POS	NEG	POS	POS	AMB	NEG	POS	AMB	AMB	POS	POS	NEG	AMB	POS	POS	POS	AMB	AMB	POS	POS	NEG
UKD-0013324	SCCmec IVh/j (HO50960412/JCSC6670)	CC22	NEG	POS	NEG	POS	POS	POS	NEG	POS	POS	NEG	NEG	NEG	NEG	NEG	POS	POS	NEG	NEG	POS	POS	POS	NEG	NEG	NEG	POS	NEG
UKD-0125329	SCCmec IVh/j (HO50960412/JCSC6670)	CC22	NEG	POS	NEG	POS	POS	POS	NEG	POS	POS	NEG	NEG	NEG	NEG	NEG	POS	POS	NEG	NEG	POS	POS	POS	NEG	NEG	POS	POS	NEG
UKD-0215498	SCCmec IVh/j (HO50960412/JCSC6670)	CC22	NEG	POS	NEG	POS	POS	POS	NEG	POS	POS	NEG	NEG	NEG	NEG	NEG	POS	POS	NEG	NEG	POS	POS	POS	NEG	NEG	NEG	POS	NEG
Prediction for M08-0126, GenBank FR753166.1	SCCmec IVh/j+ACME2 (M08-0126)	CC22	NEG																									
UKD-1379483I	SCCmec IVh/j+ACME2 (M08-0126)	CC22	NEG	POS	NEG	POS	POS	POS	NEG	POS	POS	NEG	NEG	POS	NEG	AMB	POS	POS	NEG	NEG	POS	POS	POS	NEG	NEG	POS	POS	NEG
UKD-5150204	SCCmec IV+speG+Cu/czrC+ccrAB4	CC22	NEG	POS	NEG	POS	POS	POS	NEG	POS	POS	NEG	NEG	POS	AMB	NEG	POS	POS	NEG	NEG	POS	POS	POS	NEG	NEG	POS	POS	NEG
UKD-15578423	SCCmec IV+speG+czrC+ccrAB4 var. 1	CC22	NEG	POS	NEG	POS	POS	POS	NEG	POS	POS	NEG	NEG	POS	NEG	NEG	POS	POS	NEG	NEG	POS	POS	POS	NEG	NEG	POS	POS	NEG
UKD-16582870	SCCmec IV+speG+czrC+ccrAB4 var. 1	CC22	NEG	POS	NEG	POS	POS	POS	NEG	POS	POS	NEG	NEG	NEG	NEG	NEG	POS	POS	NEG	NEG	POS	POS	POS	NEG	NEG	NEG	POS	NEG
UKD-15571940	SCCmec IV+speG+czrC+ccrAB4 var. 2	CC22	NEG	POS	NEG	POS	POS	POS	NEG	POS	POS	NEG	NEG	POS	NEG	NEG	POS	POS	NEG	NEG	POS	POS	POS	NEG	NEG	POS	POS	NEG

Isolate ID	SCCmec type	Clonal complex	CAPSULE-ASSOCIATED GENES			BIOFILM-ASSOCIATED GENES				ADHESION FACTORS / GENES ENCODING MICROBIAL SURFACE COMPONENTS RECOGNIZING ADHESIVE MATRIX MOLECULES (MSCRAMM GENES)																		
			Capsule type 1	Capsule type 5	Capsule type 8	intercellular adhesion protein A	intercellular adhesion protein C	biofilm PIA synthesis protein D	surface protein involved in biofilm formation	bone sialoprotein-binding protein							clumping factor A					clumping factor B					collagen-binding adhesin	cell wall associated fibronectin-binding protein
			cap 1	cap 5	cap 8	icaA	icaC	icaD	bap	bbp	bbp (cons)	bbp (COL+MW2)	bbp (MRSA252)	bbp (Mu50)	bbp (RF122)	bbp (ST45)	clfA	clfA (cons)	clfA (COL+RF122)	clfA (MRSA252)	clfA (Mu50+MW2)	clfB	clfB (cons)	clfB (COL+Mu50)	clfB (MW2)	clfB (RF122)	cna	ebh (cons)
Prediction for CN1, GenBank CP003979.1	***SCCmec IVA (CN1)***	***CC8 (ST72)***	***NEG***	***POS***	***NEG***	***POS***	***POS***	***POS***	***NEG***	***POS***	***POS***	***NEG***	***NEG***	***POS***	***NEG***	***NEG***	***POS***	***POS***	***POS***	***NEG***	***AMB***	***POS***	***POS***	***NEG***	***NEG***	***NEG***	***NEG***	***NEG***
**UKD-0900871**	SCCmec IVA (CN1)	CC8 (ST72)	NEG	POS	NEG	POS	POS	POS	NEG	POS	POS	NEG	NEG	POS	NEG	NEG	POS	POS	POS	AMB	AMB	POS	POS	NEG	NEG	NEG	NEG	POS
Prediction for CMFT1723, GenBank HF569096.1	***SCCmec V (Bengal Bay)***		***NEG***																									
**KHDN-10405134**	SCCmec V (Bengal Bay)	CC1	NEG	NEG	POS	POS	POS	POS	NEG	POS	POS	POS	NEG	AMB	NEG	NEG	POS	POS	NEG	NEG	POS	POS	POS	NEG	POS	AMB	POS	POS
**UKD-0932405**	SCCmec V (Bengal Bay)	CC1 (ST573/772)	NEG	POS	NEG	POS	POS	POS	NEG	POS	POS	NEG	NEG	POS	NEG	NEG	POS	POS	NEG	NEG	POS	POS	POS	NEG	NEG	NEG	POS	POS
**UKD-1003510**	SCCmec V (Bengal Bay)	CC1 (ST573/772)	NEG	POS	NEG	POS	POS	POS	NEG	POS	POS	AMB	NEG	POS	NEG	NEG	POS	POS	NEG	NEG	POS	POS	POS	NEG	NEG	POS	POS	POS
**UKD-1112420**	SCCmec V (Bengal Bay)	CC1 (ST573/772)	NEG	POS	NEG	POS	POS	POS	NEG	POS	POS	AMB	NEG	POS	NEG	NEG	POS	POS	NEG	NEG	POS	POS	POS	NEG	NEG	POS	POS	POS
**UKD-1275832**	SCCmec V (Bengal Bay)	CC1 (ST573/772)	NEG	POS	NEG	POS	POS	POS	NEG	POS	POS	AMB	NEG	POS	NEG	NEG	POS	POS	NEG	NEG	POS	POS	POS	NEG	NEG	POS	POS	POS
**UKD-13023811**	SCCmec V (Bengal Bay)	CC1 (ST573/772)	NEG	POS	NEG	POS	POS	POS	NEG	POS	POS	NEG	NEG	POS	NEG	AMB	POS	POS	NEG	NEG	POS	POS	POS	NEG	NEG	POS	POS	POS
**UKD-14572163**	SCCmec V	CC5	NEG	POS	NEG	POS	POS	POS	NEG	POS	POS	AMB	NEG	POS	NEG	NEG	POS	POS	NEG	NEG	POS	POS	POS	POS	NEG	NEG	NEG	POS
**KHDN-10421133**	SCCmec V+fus	CC97	NEG	POS	NEG	POS	POS	POS	NEG	POS	POS	AMB	NEG	POS	NEG	NEG	POS	POS	AMB	AMB	POS	POS	POS	NEG	NEG	POS	NEG	POS
**KHDN-21343541**	SCCmec V+fus+tir+ccrAB1	CC1	NEG	NEG	POS	POS	POS	POS	NEG	POS	POS	POS	NEG	AMB	NEG	NEG	POS	POS	NEG	NEG	POS	POS	POS	NEG	POS	AMB	POS	POS
**KHDN-10416893**	SCCmec V+czrC+speG+ccrAB4	CC22	NEG	POS	NEG	POS	POS	POS	NEG	POS	POS	NEG	NEG	POS	NEG	NEG	POS	POS	NEG	NEG	POS	POS	POS	NEG	NEG	POS	POS	NEG
Prediction for GR1, GenBank AJLX	***SCCmec VT (GR1)***	***CC361***	***NEG***	***NEG***	***POS***	***POS***	***POS***	***POS***	***NEG***	***POS***	***POS***	***POS***	***NEG***	***NEG***	***NEG***	***AMB***	***POS***	***POS***	***NEG***	***NEG***	***POS***	***POS***	***POS***	***POS***	***NEG***	***NEG***	***NEG***	***POS***
**UKD-0422872**	SCCmec VT (GR1)	CC152	NEG	POS	NEG	POS	NEG	POS	NEG	POS	POS	POS	NEG	NEG	NEG	AMB	POS	POS	POS	AMB	AMB	POS	POS	NEG	POS	AMB	POS	POS
Prediction for PM1, GenBank BAFA	***SCCmec VT (PM1)***	***CC59***	***NEG***	***NEG***	***POS***	***POS***	***POS***	***POS***	***NEG***	***POS***	***POS***	***NEG***	***NEG***	***NEG***	***NEG***	***NEG***	***POS***	***POS***	***POS***	***NEG***	***AMB***	***POS***	***POS***	***NEG***	***AMB***	***POS***	***NEG***	***POS***
**KHDN-000871**	SCCmec VT (PM1)	CC45 (agr I)	NEG	NEG	POS	POS	POS	POS	NEG	NEG	NEG	NEG	NEG	NEG	NEG	NEG	POS	POS	POS	NEG	POS	POS	POS	NEG	POS	AMB	POS	POS
**UKD-0725052**	SCCmec VT (PM1)	CC59	NEG	NEG	POS	POS	POS	POS	NEG	POS	POS	NEG	NEG	NEG	NEG	NEG	POS	POS	POS	NEG	POS	POS	POS	NEG	POS	POS	NEG	POS
**UKD-1312138**	SCCmec VT (PM1)	CC59	NEG	NEG	POS	POS	POS	POS	NEG	POS	POS	AMB	NEG	POS	NEG	NEG	POS	POS	POS	AMB	AMB	POS	POS	NEG	POS	AMB	NEG	POS
**UKD-Z4728**	SCCmec VT (PM1)	CC59	NEG	NEG	POS	POS	POS	POS	NEG	POS	POS	POS	NEG	AMB	NEG	NEG	POS	POS	AMB	AMB	POS	POS	POS	NEG	POS	AMB	NEG	POS
**UKD-K0199**	SCCmec VT unknown var.1	CC7	NEG	NEG	POS	POS	POS	POS	NEG	POS	POS	POS	AMB	NEG	NEG	NEG	POS	POS	POS	AMB	AMB	POS	POS	NEG	NEG	NEG	NEG	POS
**UKD-15584619**	SCCmec VT unknown var.2	CC30	NEG	NEG	POS	POS	POS	POS	NEG	POS	POS	AMB	POS	NEG	NEG	NEG	POS	POS	AMB	POS	AMB	POS	POS	NEG	NEG	POS	POS	POS
**UKD-0712568**	SCCmec VT unknown var.3	CC45 (agr I)	NEG	NEG	POS	POS	POS	POS	NEG	POS	POS	NEG	NEG	NEG	NEG	POS	POS	POS	NEG	NEG	POS	POS	POS	NEG	POS	POS	POS	POS
**KHDN-10420296**	SCCmec VT+ACME2	CC45 (agr I)	NEG	NEG	POS	POS	POS	POS	NEG	POS	POS	AMB	NEG	AMB	NEG	POS	POS	POS	AMB	NEG	POS	POS	POS	NEG	AMB	POS	POS	POS
**UKD-15577956**	SCCmec VT+Cu/czrC+ccrA1	CC398	NEG	POS	NEG	POS	POS	POS	NEG	POS	POS	AMB	NEG	AMB	NEG	POS	POS	POS	POS	AMB	AMB	POS	POS	NEG	POS	AMB	POS	POS
Prediction for S0385, GenBank AM990992	***SCCmec VT+czrC (SO385)***	***CC398***	***NEG***	***POS***	***NEG***	***POS***	***POS***	***POS***	***NEG***	***NEG***	***NEG***	***NEG***	***NEG***	***NEG***	***NEG***	***NEG***	***POS***	***POS***	***POS***	***NEG***	***AMB***	***POS***	***POS***	***NEG***	***AMB***	***AMB***	***POS***	***POS***
**UKD-0628560**	SCCmec VT+czrC (SO385)	CC8	NEG	POS	NEG	POS	POS	POS	NEG	POS	POS	POS	NEG	NEG	NEG	NEG	POS	POS	POS	AMB	AMB	POS	POS	POS	NEG	NEG	NEG	POS
**UKD-0510018**	SCCmec VT+czrC (SO385)	CC398	NEG	POS	NEG	POS	POS	POS	NEG	POS	POS	NEG	NEG	NEG	NEG	POS	POS	POS	POS	NEG	POS	POS	POS	NEG	POS	POS	POS	POS
**UKD-0903558**	SCCmec VT+czrC (SO385)	CC398	NEG	POS	NEG	POS	POS	POS	NEG	POS	POS	NEG	NEG	NEG	NEG	POS	POS	POS	AMB	AMB	POS	POS	POS	NEG	AMB	POS	POS	POS
**UKD-0929815**	SCCmec VT+czrC (SO385)	CC398	NEG	POS	NEG	POS	POS	POS	NEG	POS	POS	NEG	NEG	NEG	NEG	POS	POS	POS	POS	AMB	AMB	POS	POS	NEG	POS	AMB	POS	POS
**UKD-0907762**	SCCmec VT+czrC (SO385)	CC398	NEG	POS	NEG	POS	POS	POS	NEG	POS	POS	NEG	NEG	NEG	NEG	POS	POS	POS	POS	NEG	AMB	POS	POS	NEG	AMB	POS	POS	POS
**UKD-1025105**	SCCmec VT+czrC (SO385)	CC398	NEG	POS	NEG	POS	POS	POS	NEG	POS	POS	NEG	NEG	NEG	NEG	POS	POS	POS	POS	AMB	AMB	POS	POS	NEG	POS	AMB	POS	POS
**UKD-11780898**	SCCmec VT+czrC (SO385)	CC398	NEG	POS	NEG	POS	POS	POS	NEG	POS	POS	AMB	NEG	AMB	NEG	POS	POS	POS	AMB	AMB	POS	POS	POS	NEG	POS	AMB	POS	POS
**UKD-1377009**	SCCmec VT+czrC (SO385)	CC398	NEG	POS	NEG	POS	POS	POS	NEG	POS	POS	NEG	NEG	AMB	NEG	POS	POS	POS	POS	AMB	AMB	POS	POS	NEG	POS	AMB	POS	POS
**UKD-143406**	SCCmec VT+czrC (SO385)	CC398	NEG	POS	NEG	POS	POS	POS	NEG	POS	POS	AMB	NEG	AMB	AMB	POS	POS	POS	POS	AMB	AMB	POS	POS	NEG	POS	AMB	POS	POS
**UKD-1400363**	SCCmec VT+czrC (SO385)	CC398	NEG	POS	NEG	POS	POS	POS	NEG	POS	POS	AMB	NEG	AMB	NEG	POS	POS	POS	POS	AMB	AMB	POS	POS	NEG	POS	AMB	POS	POS
**UKD-14571852**	SCCmec VT+czrC (SO385)	CC398	NEG	POS	NEG	POS	POS	POS	NEG	POS	POS	AMB	NEG	AMB	NEG	POS	POS	POS	AMB	POS	AMB	POS	POS	NEG	POS	AMB	POS	POS
**UKD-14582574**	SCCmec VT+czrC (SO385)	CC398	NEG	POS	NEG	POS	POS	POS	NEG	POS	POS	NEG	NEG	AMB	NEG	POS	POS	POS	POS	AMB	AMB	POS	POS	NEG	POS	AMB	POS	POS
**UKD-152108**	SCCmec VT+czrC (SO385)	CC398	NEG	POS	NEG	POS	POS	POS	NEG	POS	POS	AMB	NEG	AMB	NEG	POS	POS	POS	AMB	AMB	POS	POS	POS	NEG	POS	AMB	POS	POS
**UKD-15573321**	SCCmec VT+czrC (SO385)	CC398	NEG	POS	NEG	POS	POS	POS	NEG	POS	POS	AMB	NEG	AMB	NEG	POS	POS	POS	POS	AMB	AMB	POS	POS	NEG	POS	AMB	POS	POS
**UKD-15584034**	SCCmec VT+czrC , ydhK-neg. variant	CC398	NEG	POS	NEG	POS	POS	POS	NEG	POS	POS	AMB	NEG	AMB	NEG	POS	POS	POS	POS	AMB	AMB	POS	POS	NEG	POS	AMB	POS	POS
**UKD-0932581**	SCCmecVT+fus+czrC	CC22	NEG	POS	NEG	POS	POS	POS	NEG	POS	POS	NEG	NEG	NEG	NEG	NEG	POS	POS	NEG	NEG	POS	POS	POS	NEG	NEG	NEG	POS	NEG
**UKD-1121994**	SCCmecVT+fus+czrC	CC22	NEG	POS	NEG	POS	POS	POS	NEG	POS	POS	NEG	NEG	POS	NEG	NEG	POS	POS	NEG	NEG	POS	POS	POS	NEG	NEG	POS	POS	NEG
**UKD-1272252**	SCCmecVT+fus+czrC	CC22	NEG	POS	NEG	POS	POS	POS	NEG	POS	POS	NEG	NEG	POS	NEG	NEG	POS	POS	NEG	NEG	POS	POS	POS	NEG	NEG	POS	POS	NEG
Prediction for MRSA18	***SCCmec VI+fus (MRSA18)***		***NEG***																									
**UKD-1103380**	SCCmec VI+fus (MRSA18)	CC7	NEG	NEG	POS	POS	POS	POS	NEG	POS	POS	POS	AMB	NEG	NEG	NEG	POS	POS	POS	NEG	AMB	POS	POS	NEG	NEG	NEG	NEG	POS
Prediction for M10/0061, GenBank FR823292.1	***SCCmec XI (LGA251/M10-61)***	***CC130***	***NEG***																									
**Reference strain Dublin-DSH_M10 0061**	**SCCmec XI (LGA251/M10-61)**	**CC130**	**NEG**	**NEG**	**POS**	**POS**	**POS**	**POS**	**NEG**	**POS**	**POS**	**NEG**	**NEG**	**POS**	**NEG**	**AMB**	**POS**	**POS**	**POS**	**AMB**	**AMB**	**POS**	**POS**	**NEG**	**AMB**	**POS**	**NEG**	**POS**
Prediction for LGA251, GenBank FR821779.1	***SCCmec XI (LGA251/M10-61)***	***ST425***	***NEG***																									
**UKD-14015835**	SCCmec XI (LGA251/M10-61)	CC130	NEG	NEG	POS	POS	POS	POS	NEG	POS	POS	NEG	NEG	POS	NEG	NEG	POS	POS	POS	AMB	AMB	POS	POS	NEG	AMB	POS	NEG	POS
**UKD-16573252**	SCCmec XI (LGA251/M10-61)	CC130	NEG	NEG	POS	POS	POS	POS	NEG	POS	POS	AMB	NEG	POS	NEG	NEG	POS	POS	POS	AMB	POS	POS	POS	NEG	POS	POS	NEG	POS
**UKD-0217002**	PseudoSCCmec, class B mec complex+Hg	CC8	NEG	POS	NEG	POS	POS	POS	NEG	NEG	NEG	NEG	NEG	NEG	NEG	NEG	POS	POS	POS	AMB	AMB	POS	POS	POS	NEG	NEG	NEG	POS
**UKD-0008510**	PseudoSCCmec, class B mec complex+Hg	CC8	NEG	POS	NEG	POS	POS	POS	NEG	NEG	NEG	NEG	NEG	NEG	NEG	NEG	POS	POS	POS	NEG	POS	POS	POS	POS	NEG	NEG	NEG	POS
**UKD-0110962**	PseudoSCCmec, class B mec complex+Hg	CC8	NEG	POS	NEG	POS	POS	POS	NEG	NEG	NEG	NEG	NEG	NEG	NEG	NEG	POS	POS	POS	POS	POS	POS	POS	POS	NEG	NEG	NEG	POS
**KHDN-10408593**	PseudoSCCmec, class B mec complex+Cu/Hg	CC8	NEG	POS	NEG	POS	POS	POS	NEG	POS	POS	POS	NEG	NEG	NEG	NEG	POS	POS	POS	AMB	AMB	POS	POS	POS	NEG	NEG	NEG	POS
**UKD-0007201**	PseudoSCCmec, class B mec complex	CC5	NEG	POS	NEG	POS	POS	POS	NEG	POS	POS	NEG	NEG	POS	NEG	NEG	POS	POS	NEG	NEG	POS	POS	POS	POS	NEG	NEG	NEG	POS
**UKD-0033204**	PseudoSCCmec, class B mec complex	CC5	NEG	POS	NEG	POS	POS	POS	NEG	POS	POS	NEG	NEG	POS	NEG	NEG	POS	POS	NEG	NEG	POS	POS	POS	POS	NEG	NEG	NEG	POS
**UKD-0134329**	PseudoSCCmec, class B mec complex	CC5	NEG	POS	NEG	POS	AMB	POS	NEG	POS	POS	NEG	NEG	POS	NEG	NEG	POS	POS	NEG	NEG	POS	POS	POS	POS	NEG	NEG	NEG	AMB
**UKD-0806946**	PseudoSCCmec class C+As/Cu	CC398	NEG	POS	NEG	POS	POS	POS	NEG	POS	POS	NEG	NEG	NEG	NEG	POS	POS	POS	POS	AMB	POS	POS	POS	NEG	POS	AMB	POS	POS
**UKD-0500610**	ACME2	CC8	NEG	POS	NEG	POS	POS	POS	NEG	POS	POS	POS	NEG	NEG	NEG	NEG	POS	POS	POS	POS	POS	POS	POS	POS	NEG	NEG	NEG	POS
**UKD-0610438**	ACME2	CC8	NEG	POS	NEG	POS	POS	POS	NEG	POS	POS	POS	NEG	AMB	NEG	NEG	POS	POS	POS	AMB	AMB	POS	POS	POS	NEG	NEG	NEG	POS
**KHDN-10409344**	ACME2	CC8	NEG	POS	NEG	POS	POS	POS	NEG	POS	POS	POS	NEG	AMB	NEG	NEG	POS	POS	AMB	POS	POS	POS	POS	POS	NEG	NEG	NEG	POS
**UKD-0322559**	ACME2+speG+czrC+ccrAB4	CC8	NEG	POS	NEG	POS	POS	POS	NEG	POS	POS	POS	NEG	NEG	NEG	NEG	POS	POS	POS	NEG	POS	POS	POS	POS	NEG	NEG	NEG	POS
**UKD-0501998**	ACME2+speG+czrC+ccrAB4	CC8	NEG	POS	NEG	POS	POS	POS	NEG	POS	POS	POS	NEG	NEG	NEG	NEG	POS	POS	POS	POS	POS	POS	POS	POS	NEG	NEG	NEG	POS
**UKD-0505213**	ACME2+speG+czrC+ccrAB4	CC8	NEG	POS	NEG	POS	POS	POS	NEG	POS	POS	POS	NEG	NEG	NEG	NEG	POS	POS	POS	POS	POS	POS	POS	POS	NEG	NEG	NEG	POS
Prediction for MSSA476, GenBank BX571857.1	***fus+tir+ccrAB1 (MSSA476)***	***CC1***	***NEG***	***NEG***	***POS***	***POS***	***POS***	***POS***	***NEG***	***POS***	***POS***	***POS***	***NEG***	***NEG***	***NEG***	***NEG***	***POS***	***POS***	***NEG***	***NEG***	***POS***	***POS***	***POS***	***NEG***	***POS***	***AMB***	***POS***	***POS***
**Reference strain Sanger 476**	**fus+tir+ccrAB1 (MSSA476)**	**CC1**	**NEG**	**NEG**	**POS**	**POS**	**POS**	**POS**	**NEG**	**POS**	**POS**	**POS**	**NEG**	**NEG**	**NEG**	**NEG**	**POS**	**POS**	**NEG**	**NEG**	**POS**	**POS**	**POS**	**NEG**	**POS**	**AMB**	**POS**	**POS**
**UKD-14023914**	fus+tir+ccrAB1 (MSSA476)	CC1	NEG	NEG	POS	POS	POS	POS	NEG	NEG	NEG	NEG	NEG	NEG	NEG	NEG	POS	POS	NEG	NEG	POS	POS	POS	NEG	POS	AMB	POS	POS
**UKD-K00034**	kdp+ccrAB2	CC182	NEG	POS	NEG	POS	POS	POS	NEG	NEG	NEG	NEG	NEG	NEG	NEG	NEG	POS	POS	POS	AMB	POS	POS	POS	NEG	NEG	AMB	POS	POS
**UKD-15580369**	As+ccrAB4	CC22	NEG	POS	NEG	POS	POS	POS	NEG	POS	POS	NEG	NEG	POS	NEG	NEG	POS	POS	NEG	NEG	POS	POS	POS	NEG	NEG	POS	POS	NEG
**UKD-15570100**	ACME3+ccrAB1 (Strain 21342)	CC80	NEG	NEG	POS	POS	POS	POS	NEG	POS	POS	AMB	NEG	POS	NEG	NEG	POS	POS	NEG	NEG	POS	POS	POS	POS	NEG	NEG	NEG	POS
**UKD-1010678**	speG+czrC+ccrAB4	CC8	NEG	POS	NEG	POS	POS	POS	NEG	POS	POS	POS	NEG	NEG	NEG	NEG	POS	POS	POS	AMB	AMB	POS	POS	POS	NEG	NEG	NEG	POS
**UKD-1008243**	speG+czrC+ccrAB4	CC8	NEG	POS	NEG	POS	POS	POS	NEG	POS	POS	POS	NEG	NEG	NEG	NEG	POS	POS	POS	AMB	AMB	POS	POS	POS	NEG	NEG	NEG	POS
**UKD-1009430**	speG+czrC+ccrAB4	CC8	NEG	POS	NEG	POS	POS	POS	NEG	POS	POS	POS	NEG	NEG	NEG	NEG	POS	POS	POS	AMB	AMB	POS	POS	POS	NEG	NEG	NEG	POS
**UKD-12071828**	speG+czrC+ccrAB4	CC22	NEG	POS	NEG	POS	POS	POS	NEG	POS	POS	NEG	NEG	AMB	NEG	NEG	POS	POS	NEG	NEG	POS	POS	POS	NEG	NEG	AMB	POS	NEG
**UKD-14011883**	speG+czrC+ccrAB4	CC22	NEG	POS	NEG	POS	POS	POS	NEG	POS	POS	NEG	NEG	POS	NEG	NEG	POS	POS	NEG	NEG	POS	POS	POS	NEG	NEG	POS	POS	NEG
**UKD-15008293**	speG+czrC+ccrAB4	CC22	NEG	POS	NEG	POS	POS	POS	NEG	POS	POS	NEG	NEG	POS	NEG	NEG	POS	POS	NEG	NEG	POS	POS	POS	NEG	NEG	POS	POS	NEG
**UKD-1514877**	speG+czrC+ccrAB4	CC22	NEG	POS	NEG	POS	POS	POS	NEG	POS	POS	NEG	NEG	POS	NEG	NEG	POS	POS	NEG	NEG	POS	POS	POS	NEG	NEG	POS	POS	NEG
**UKD-15580971**	speG+czrC+ccrAB4	CC22	NEG	POS	NEG	POS	POS	POS	NEG	POS	POS	NEG	NEG	POS	NEG	NEG	POS	POS	NEG	NEG	POS	POS	POS	NEG	NEG	POS	POS	NEG
**UKD-15582646**	speG+czrC+ccrAB4	CC22	NEG	POS	NEG	POS	POS	POS	NEG	POS	POS	NEG	NEG	POS	NEG	NEG	POS	POS	NEG	NEG	POS	POS	POS	NEG	NEG	POS	POS	NEG

Isolate ID	SCCmec type	Clonal complex	ebpS	ebpS_probe 612	ebpS_probe 614	ebpS (01-1111)	ebpS (COL)	eno	efb / fib	efb / fib (MRSA252)	fnbA	fnbA (cons)	fnbA (COL)	fnbA (MRSA252)	fnbA (Mu50+MW2)	fnbA (RF122)	fnbB	fnbB (COL)	fnbB (COL+Mu50+MW2)	fnbB (Mu50)	fnbB (MW2)	fnbB (ST15)	fnbB (ST45-2)	map	map (COL)	map (MRSA252)	map (Mu50+MW2)	sasG	sasG (COL+Mu50)	sasG (MW2)	sasG (OtherThan252+122
			ADHAESION FACTORS / GENES ENCODING MICROBIAL SURFACE COMPONENTS RECOGNIZING ADHESIVE MATRIX MOLECULES (MSCRAMM GENES)																												
			cell surface elastin binding protein					enolase	fibrinogen binding protein (19 kDa)		fibronectin-binding protein A						fibronectin-binding protein B							Major histocompatibility complex class II analog protein (=Extracellular adherence protein, eap)				Staphylococcus aureus surface protein G			
VIRTUAL HYBRIDISATION for COL, GenBank CP000046	SCCmec I (COL)	CC8	POS	POS	POS	NEG	POS	POS	POS	NEG	POS	POS	POS	NEG	NEG	NEG	POS	AMB	AMB	NEG	NEG	NEG	NEG	POS	POS	NEG	NEG	POS	POS	NEG	POS
Reference strain COL	SCCmec I (COL)	CC8	POS	POS	POS	NEG	POS	POS	POS	NEG	POS	POS	POS	NEG	NEG	NEG	POS	POS	POS	NEG	NEG	NEG	NEG	POS	POS	NEG	NEG	POS	POS	NEG	POS
UKD-0007063	SCCmec I (COL)	CC5	POS	POS	POS	NEG	NEG	POS	POS	NEG	POS	POS	NEG	NEG	POS	NEG	NEG	NEG	NEG	NEG	NEG	NEG	NEG	POS	POS	AMB	POS	POS	POS	AMB	POS
UKD-0220759	SCCmec I (COL)	CC5	POS	AMB	POS	NEG	NEG	POS	POS	NEG	POS	POS	NEG	NEG	POS	NEG	NEG	NEG	NEG	NEG	NEG	NEG	NEG	POS	NEG	NEG	POS	POS	POS	NEG	POS
UKD-0308986	SCCmec I (COL)	CC5	POS	POS	POS	NEG	NEG	POS	POS	NEG	POS	POS	NEG	NEG	POS	NEG	NEG	NEG	NEG	NEG	NEG	NEG	NEG	POS	POS	NEG	POS	POS	POS	NEG	POS
UKD-0500009	SCCmec I (COL)	CC5	POS	POS	POS	NEG	NEG	POS	POS	NEG	POS	POS	NEG	NEG	POS	NEG	NEG	NEG	NEG	NEG	NEG	NEG	NEG	POS	AMB	NEG	POS	POS	POS	NEG	POS
UKD-0508483	SCCmec I (COL)	CC5	POS	POS	POS	NEG	NEG	POS	POS	NEG	POS	POS	NEG	NEG	POS	NEG	NEG	NEG	NEG	NEG	NEG	NEG	NEG	POS	NEG	NEG	POS	POS	POS	NEG	POS
UKD-0519001	SCCmec I (COL)	CC5	POS	POS	POS	NEG	NEG	POS	POS	NEG	POS	POS	NEG	NEG	POS	NEG	NEG	NEG	NEG	NEG	NEG	NEG	NEG	POS	NEG	NEG	POS	POS	POS	NEG	POS
UKD-0701876	SCCmec I (COL)	CC5	POS	POS	POS	NEG	NEG	POS	POS	NEG	POS	POS	NEG	NEG	POS	NEG	NEG	NEG	NEG	NEG	NEG	NEG	NEG	POS	POS	NEG	POS	POS	POS	NEG	POS
UKD-0708865	SCCmec I (COL)	CC5	POS	AMB	POS	NEG	NEG	POS	POS	NEG	POS	POS	NEG	NEG	AMB	NEG	NEG	NEG	NEG	NEG	NEG	NEG	NEG	POS	NEG	NEG	POS	POS	POS	NEG	POS
UKD-1001782	SCCmec I (COL)	CC5	POS	POS	POS	NEG	NEG	POS	POS	NEG	POS	POS	NEG	NEG	POS	NEG	NEG	NEG	NEG	NEG	NEG	NEG	NEG	POS	POS	NEG	POS	POS	POS	NEG	POS
Prediction for PSP1996, GenBank ANHU	SCCmec I (PSP1996)	CC8	POS	POS	POS	NEG	POS	POS	POS	NEG	POS	POS	POS	NEG	NEG	NEG	POS	AMB	AMB	NEG	NEG	NEG	NEG	POS	POS	NEG	NEG	POS	POS	NEG	POS
UKD-0261727	SCCmec I (PSP1996)	CC8	POS	POS	POS	NEG	POS	POS	POS	NEG	POS	POS	POS	NEG	NEG	NEG	POS	POS	AMB	NEG	NEG	NEG	NEG	POS	POS	NEG	NEG	POS	POS	NEG	POS
VIRTUAL HYBRIDISATION for MRSA7 : VIRTUAL HYBRII	SCCmec I+fus+tirS (Geraldine Clone)	CC5	POS	POS	POS	NEG	NEG	POS	POS	NEG	POS	POS	NEG	NEG	POS	NEG	POS	NEG	AMB	AMB	NEG	NEG	NEG	POS	AMB	NEG	POS	POS	POS	NEG	POS
UKD-1221930	SCCmec I+fus+tirS (Geraldine Clone)	CC5	POS	POS	POS	NEG	NEG	POS	POS	NEG	POS	POS	NEG	NEG	POS	NEG	POS	NEG	AMB	POS	NEG	AMB	NEG	POS	POS	NEG	POS	POS	POS	NEG	POS
Prediction for N315, GenBank BA000018.3	SCCmec II (N315)	CC5	POS	POS	POS	NEG	NEG	POS	POS	NEG	POS	POS	NEG	NEG	POS	NEG	POS	NEG	AMB	AMB	NEG	NEG	NEG	POS	AMB	NEG	POS	POS	POS	NEG	POS
Reference strain N315	SCCmec II (N315)	CC5	POS	POS	POS	NEG	NEG	POS	POS	NEG	POS	POS	NEG	NEG	POS	NEG	POS	NEG	POS	POS	NEG	NEG	NEG	POS	AMB	NEG	POS	POS	POS	NEG	POS
Prediction for MRSA252, GenBank BX571856.1	SCCmec II (N315)	CC30	POS	POS	POS	NEG	NEG	POS	NEG	POS	POS	POS	NEG	POS	NEG	NEG	NEG	NEG	NEG	NEG	NEG	NEG	NEG	POS	NEG	POS	NEG	NEG	NEG	NEG	NEG
Reference strain Sanger 252	SCCmec II (N315)	CC30	POS	POS	POS	NEG	NEG	POS	NEG	POS	POS	POS	NEG	POS	NEG	NEG	NEG	NEG	NEG	NEG	NEG	NEG	NEG	POS	NEG	POS	NEG	NEG	NEG	NEG	NEG
UKD-0230792	SCCmec II (N315)	CC30	POS	POS	POS	NEG	NEG	POS	NEG	POS	POS	POS	NEG	POS	NEG	NEG	NEG	NEG	NEG	NEG	NEG	NEG	NEG	POS	NEG	POS	NEG	NEG	NEG	NEG	NEG
Prediction for Mu50, GenBank BA000017.4	SCCmec II (N315)	CC5	POS	POS	POS	NEG	NEG	POS	POS	NEG	POS	POS	NEG	NEG	POS	NEG	POS	NEG	AMB	AMB	NEG	NEG	NEG	POS	AMB	NEG	POS	POS	POS	NEG	POS
Reference strain MU50	SCCmec II (N315)	CC5	POS	POS	POS	NEG	NEG	POS	POS	NEG	POS	POS	NEG	NEG	POS	NEG	POS	NEG	POS	POS	NEG	NEG	NEG	POS	POS	NEG	POS	POS	POS	NEG	POS
Prediction for JH1, GenBank CP000736.1	SCCmec II (JH1/JH9)	CC5	POS	POS	POS	NEG	NEG	POS	POS	NEG	POS	POS	NEG	NEG	POS	NEG	POS	NEG	AMB	AMB	NEG	NEG	NEG	POS	AMB	NEG	POS	POS	POS	NEG	POS
Prediction for JH9, GenBank CP000703.1	SCCmec II (JH1/JH9)	CC5	POS	POS	POS	NEG	NEG	POS	POS	NEG	POS	POS	NEG	NEG	POS	NEG	POS	NEG	AMB	AMB	NEG	NEG	NEG	POS	AMB	NEG	POS	POS	POS	NEG	POS
Reference strain Dublin-DSH_AR07.4_0237	SCCmec II (JH1/JH9)		POS	POS	POS	NEG	NEG	POS	POS	NEG	POS	POS	NEG	NEG	POS	NEG	POS	NEG	AMB	POS	NEG	AMB	NEG	POS	POS	NEG	POS	POS	POS	NEG	POS
Reference strain NARSA_382 / USA100	SCCmec II (JH1/JH9)	CC5	POS	POS	POS	NEG	NEG	POS	POS	NEG	POS	POS	NEG	NEG	POS	NEG	POS	NEG	POS	AMB	NEG	NEG	NEG	POS	POS	NEG	POS	POS	POS	NEG	POS
UKD-0046921	SCCmec II (JH1/JH9)	CC5	POS	POS	POS	NEG	NEG	POS	POS	NEG	POS	POS	NEG	NEG	POS	NEG	POS	NEG	AMB	POS	NEG	NEG	NEG	POS	POS	NEG	POS	POS	POS	NEG	POS
UKD-0333415	SCCmec II (JH1/JH9)	CC5	POS	POS	POS	NEG	NEG	POS	POS	NEG	POS	POS	NEG	NEG	POS	NEG	POS	NEG	POS	AMB	NEG	AMB	NEG	POS	POS	NEG	POS	POS	POS	NEG	POS
UKD-0341645	SCCmec II (JH1/JH9)	CC5	POS	POS	POS	NEG	NEG	POS	POS	NEG	POS	POS	NEG	NEG	POS	NEG	POS	NEG	POS	AMB	NEG	AMB	NEG	POS	POS	NEG	POS	POS	POS	NEG	POS
UKD-0504626	SCCmec II (JH1/JH9)	CC5	POS	POS	POS	NEG	NEG	POS	POS	NEG	POS	POS	NEG	NEG	POS	NEG	POS	NEG	POS	POS	NEG	NEG	NEG	POS	NEG	NEG	POS	POS	POS	NEG	POS
UKD-0507581	SCCmec II (JH1/JH9)	CC5	POS	POS	POS	NEG	NEG	POS	POS	AMB	POS	POS	NEG	NEG	POS	AMB	POS	NEG	POS	POS	NEG	NEG	NEG	POS	NEG	NEG	POS	POS	POS	NEG	POS
UKD-0507903	SCCmec II (JH1/JH9)	CC5	POS	POS	POS	NEG	NEG	POS	POS	NEG	POS	POS	NEG	NEG	POS	AMB	POS	NEG	POS	POS	NEG	NEG	NEG	POS	NEG	NEG	POS	POS	POS	NEG	POS
UKD-0700817	SCCmec II (JH1/JH9)	CC5	POS	AMB	POS	NEG	NEG	POS	POS	NEG	POS	POS	NEG	NEG	POS	NEG	POS	NEG	POS	POS	NEG	NEG	NEG	POS	NEG	NEG	POS	POS	POS	NEG	POS
UKD-1014561	SCCmec II (JH1/JH9)	CC5	POS	POS	POS	NEG	NEG	POS	POS	NEG	POS	POS	NEG	NEG	POS	NEG	POS	NEG	AMB	POS	NEG	NEG	NEG	POS	AMB	NEG	POS	POS	POS	NEG	POS
UKD-1037507	SCCmec II (JH1/JH9)	CC5	POS	POS	POS	NEG	NEG	POS	POS	NEG	POS	POS	NEG	NEG	POS	NEG	POS	NEG	AMB	POS	NEG	AMB	NEG	POS	POS	NEG	POS	POS	POS	NEG	POS
UKD-1038321	SCCmec II (JH1/JH9)	CC5	POS	POS	POS	NEG	NEG	POS	POS	NEG	POS	POS	NEG	NEG	POS	NEG	POS	NEG	POS	AMB	NEG	NEG	NEG	POS	NEG	NEG	POS	POS	POS	NEG	POS
UKD-1111608	SCCmec II (JH1/JH9)	CC5	POS	POS	POS	NEG	NEG	POS	POS	NEG	POS	POS	NEG	NEG	POS	NEG	POS	NEG	AMB	POS	NEG	AMB	NEG	POS	POS	NEG	POS	POS	POS	NEG	POS
UKD-12753135	SCCmec II (JH1/JH9)	CC5	POS	POS	POS	NEG	NEG	POS	POS	NEG	POS	POS	NEG	NEG	POS	NEG	POS	NEG	AMB	POS	NEG	AMB	NEG	POS	POS	NEG	POS	POS	POS	NEG	POS
UKD-1312816	SCCmec II (JH1/JH9)	CC5	POS	POS	POS	NEG	NEG	POS	POS	NEG	POS	POS	NEG	NEG	POS	NEG	POS	NEG	AMB	POS	NEG	AMB	NEG	POS	POS	NEG	POS	POS	POS	NEG	POS
UKD-1401623	SCCmec II (JH1/JH9)	CC5	POS	POS	POS	NEG	NEG	POS	POS	POS	POS	POS	NEG	NEG	POS	NEG	POS	NEG	AMB	POS	NEG	AMB	NEG	POS	POS	NEG	POS	POS	POS	NEG	POS
UKD-15571943	SCCmec II+czrC+ccrA/B-4	CC5	POS	POS	POS	NEG	AMB	POS	POS	NEG	POS	POS	NEG	NEG	POS	NEG	POS	NEG	AMB	POS	NEG	AMB	NEG	POS	POS	POS	POS	POS	POS	NEG	POS
UKD-15576888	SCCmec II+czrC+ccrA/B-4	CC5	POS	POS	POS	NEG	NEG	POS	POS	NEG	POS	POS	NEG	NEG	POS	NEG	POS	NEG	AMB	POS	NEG	AMB	NEG	POS	POS	NEG	POS	POS	POS	NEG	POS
UKD-14576461	SCCmec II+speG+czrC+ccrA/B-4	CC5	POS	POS	POS	NEG	NEG	POS	POS	NEG	POS	POS	NEG	NEG	POS	NEG	NEG	NEG	NEG	NEG	NEG	NEG	NEG	POS	POS	NEG	POS	POS	POS	NEG	POS
UKD-15581649	SCCmec II+speG+czrC+ccrA/B-4	CC5	POS	POS	POS	NEG	NEG	POS	POS	NEG	POS	POS	NEG	NEG	POS	NEG	NEG	NEG	NEG	NEG	NEG	NEG	NEG	POS	POS	NEG	POS	POS	POS	NEG	POS
Prediction for CIGC345D, GenBank AHVO	SCCmec II A/B/D	CC8	POS	POS	POS	NEG	POS	POS	POS	NEG	POS	POS	POS	NEG	NEG	NEG	POS	AMB	AMB	NEG	NEG	NEG	NEG	POS	POS	NEG	NEG	POS	POS	NEG	POS
UKD-12K00171	SCCmec II A/B/D	CC8	POS	POS	POS	NEG	POS	POS	POS	NEG	POS	POS	POS	NEG	NEG	NEG	POS	AMB	AMB	POS	NEG	NEG	NEG	POS	POS	NEG	POS	POS	POS	NEG	POS
Prediction for JKD6008, GenBank CP002120.1	SCC [mec III+Cd] (JKD6008)	CC8 (ST239)	POS	POS	POS	NEG	POS	POS	POS	NEG	POS	POS	POS	NEG	NEG	NEG	POS	AMB	AMB	NEG	NEG	NEG	NEG	POS	POS	NEG	NEG	POS	POS	NEG	POS
Prediction for JKD6009, GenBank ABSA	SCC [mec III+Cd] (JKD6008)	CC8 (ST239)	POS	POS	POS	NEG	POS	POS	POS	NEG	POS	POS	POS	NEG	NEG	NEG	POS	AMB	AMB	NEG	NEG	NEG	NEG	POS	POS	NEG	NEG	POS	POS	NEG	POS
Reference strain ATCC_33592	SCC [mec III+Cd] (ATCC33592)	CC8 (ST239)	AMB	POS	POS	NEG	POS	POS	POS	NEG	POS	POS	POS	NEG	NEG	NEG	POS	POS	AMB	AMB	NEG	NEG	NEG	POS	POS	NEG	NEG	POS	POS	NEG	POS
Prediction for PPUKM-775-2009, GenBank AMRE	SCC [mec III+Cd+ccrC] (PPUKM-775-2009)	CC8 (ST239)	POS	POS	POS	NEG	POS	POS	POS	NEG	POS	POS	POS	NEG	NEG	NEG	POS	AMB	AMB	NEG	NEG	NEG	NEG	POS	POS	NEG	NEG	POS	POS	NEG	POS
Prediction for T0131, GenBank CP002643.1	SCC [mec III+Cd+ccrC] (T0131)	CC8 (ST239)	POS	POS	POS	NEG	POS	POS	POS	NEG	POS	POS	POS	NEG	NEG	NEG	NEG	NEG	NEG	NEG	NEG	NEG	NEG	POS	POS	NEG	NEG	POS	POS	NEG	POS
Prediction for XN108, GenBank CP007447.1	SCC [mec III+Cd+ccrC] (XN108)	CC8 (ST239)	POS	POS	POS	NEG	POS	POS	POS	NEG	POS	POS	POS	NEG	NEG	NEG	POS	AMB	AMB	NEG	NEG	NEG	NEG	POS	POS	NEG	NEG	POS	POS	NEG	POS
Prediction for CN79, GenBank ANCJ	SCC [mec III+Cd+ccrC] (CN79)	CC8 (ST239)	POS	POS	POS	NEG	POS	POS	POS	NEG	POS	POS	POS	NEG	NEG	NEG	NEG	NEG	NEG	NEG	NEG	NEG	NEG	POS	POS	NEG	NEG	POS	POS	NEG	POS
Prediction for 16K, GenBank BABZ	SCC [mec III+Cd+ccrC] (CN79)	CC8 (ST239)	POS	POS	POS	NEG	POS	POS	POS	NEG	POS	POS	POS	NEG	NEG	NEG	NEG	NEG	NEG	NEG	NEG	NEG	NEG	POS	POS	NEG	NEG	POS	POS	NEG	POS
UKD-0835987	SCC [mec III+Cd+ccrC] (CN79)	CC8 (ST239)	POS	POS	POS	NEG	POS	POS	POS	NEG	POS	POS	POS	NEG	NEG	NEG	NEG	NEG	NEG	NEG	NEG	NEG	NEG	POS	POS	NEG	NEG	POS	POS	NEG	POS
UKD-16581769	SCCmec III+Cd+ccrC	CC8 (ST239)	POS	POS	POS	NEG	POS	POS	POS	NEG	POS	POS	POS	NEG	NEG	NEG	NEG	NEG	NEG	NEG	NEG	NEG	NEG	POS	POS	NEG	NEG	POS	POS	NEG	POS
Prediction for Bmb9393, GenBank CP005288.1	SCCmec III+Cd/Hg+ccrC (Bmb9393)	CC8 (ST239)	POS	POS	POS	NEG	POS	POS	POS	NEG	POS	POS	POS	NEG	NEG	NEG	POS	AMB	AMB	NEG	NEG	NEG	NEG	POS	POS	NEG	NEG	POS	POS	NEG	POS
UKD-15574263	SCCmec III+Cd/Hg+ccrC (Bmb9393)	CC8 (ST239)	POS	POS	POS	NEG	POS	POS	POS	NEG	POS	POS	POS	NEG	NEG	NEG	POS	POS	AMB	AMB	NEG	NEG	NEG	POS	POS	NEG	AMB	POS	POS	NEG	POS
Prediction for SK1585, GenBank AYLT	SCCmec III+ccrC+Cd/Hg (SK1585)	CC8 (ST239)	POS	NEG	POS	POS	NEG	POS	NEG	NEG	POS	POS	POS	NEG	NEG	NEG	POS	AMB	AMB	NEG	NEG	NEG	NEG	POS	NEG	POS	NEG	POS	POS	NEG	POS
UKD-105861	SCCmec III+ccrC+Cd/Hg (SK1585)	CC8 (ST239)	POS	POS	POS	NEG	POS	POS	POS	NEG	NEG	NEG	NEG	NEG	NEG	NEG	POS	POS	AMB	NEG	NEG	NEG	NEG	POS	POS	NEG	NEG	POS	POS	NEG	POS
UKD-134572	SCCmec III+ccrC+Cd/Hg (SK1585)	CC8 (ST239)	AMB	AMB	POS	NEG	POS	POS	POS	NEG	NEG	NEG	NEG	NEG	NEG	NEG	POS	POS	POS	NEG	NEG	NEG	NEG	POS	POS	NEG	NEG	POS	POS	NEG	POS
UKD-139143	SCCmec III+ccrC+Cd/Hg (SK1585)	CC8 (ST239)	POS	POS	POS	NEG	POS	POS	POS	NEG	NEG	NEG	NEG	NEG	NEG	NEG	POS	POS	AMB	NEG	NEG	NEG	NEG	POS	POS	NEG	NEG	POS	POS	NEG	POS
Prediction for TW20, GenBank FN433596.1	SCC [mec III+Cd/Hg+ccrC] (TW20)	CC8 (ST239)	POS	POS	POS	NEG	POS	POS	POS	NEG	POS	POS	POS	NEG	NEG	NEG	POS	AMB	AMB	NEG	NEG	NEG	NEG	POS	POS	NEG	NEG	POS	POS	NEG	POS
Prediction for ATCC BAA-39, GenBank AEEK	SCC [mec III+Cd/Hg+ccrC] (TW20)	CC8 (ST239)	POS	POS	POS	NEG	POS	POS	POS	NEG	POS	POS	POS	NEG	NEG	NEG	POS	AMB	AMB	NEG	NEG	NEG	NEG	POS	POS	NEG	NEG	POS	POS	NEG	POS

Isolate ID	SCCmec type	Clonal complex	ADHAESION FACTORS / GENES ENCODING MICROBIAL SURFACE COMPONENTS RECOGNIZING ADHESIVE MATRIX MOLECULES (MSCRAMM GENES)																												
			cell surface elastin binding protein					enolase	fibrinogen binding protein (19 kDa)		fibronectin-binding protein A						fibronectin-binding protein B							Major histocompatibility complex class II analog protein (=Extracellular adherence protein, eap)				Staphylococcus aureus surface protein G			
			ebpS	ebpS_probe 612	ebpS_probe 614	ebpS (01-1111)	ebpS (COL)	eno	efb / fib	efb / fib (MRSA252)	fnbA	fnbA (cons)	fnbA (COL)	fnbA (MRSA252)	fnbA (Mu50+MW2)	fnbA (RF122)	fnbB	fnbB (COL)	fnbB (COL+Mu50+MW2)	fnbB (Mu50)	fnbB (MW2)	fnbB (ST15)	fnbB (ST45-2)	map	map (COL)	map (MRSA252)	map (Mu50+MW2)	sasG	sasG (COL+Mu50)	sasG (MW2)	sasG (OtherThan252+122)
Prediction for MW2, GenBank BA000033.2	*SCCmec IVa (MW2)*	*CC1*	POS	POS	POS	NEG	NEG	POS	POS	NEG	POS	POS	NEG	NEG	POS	NEG	POS	NEG	AMB	NEG	AMB	NEG	NEG	POS	AMB	NEG	POS	POS	NEG	POS	POS
Reference strain MW2	SCCmec IVa (MW2)	CC1	POS	POS	POS	NEG	NEG	POS	POS	NEG	POS	POS	NEG	NEG	POS	NEG	POS	NEG	POS	NEG	POS	NEG	NEG	NEG	AMB	NEG	AMB	POS	NEG	POS	POS
Reference strain CA05 (JCSC1968)	SCCmec IVa (MW2)	CC45	POS	NEG	POS	POS	NEG	POS	NEG	POS	POS	POS	NEG	NEG	POS	NEG	POS	NEG	POS	AMB	NEG	AMB	NEG	POS	NEG	POS	NEG	NEG	NEG	NEG	NEG
UKD-0127106	SCCmec IVa (MW2)	CC1	POS	POS	POS	NEG	NEG	POS	POS	NEG	POS	POS	NEG	NEG	POS	NEG	POS	NEG	POS	NEG	POS	NEG	NEG	POS	NEG	NEG	POS	POS	NEG	POS	POS
UKD-0701446	SCCmec IVa (MW2)	CC1	POS	POS	POS	NEG	NEG	POS	POS	NEG	POS	POS	NEG	NEG	POS	NEG	POS	NEG	AMB	NEG	POS	NEG	NEG	POS	NEG	NEG	POS	POS	NEG	POS	POS
UKD-0947486	SCCmec IVa (MW2)	CC1	POS	POS	POS	NEG	NEG	POS	POS	NEG	POS	POS	NEG	NEG	POS	NEG	POS	NEG	AMB	NEG	POS	NEG	NEG	POS	POS	NEG	POS	POS	NEG	POS	POS
UKD-1031910	SCCmec IVa (MW2)	CC1	POS	POS	POS	NEG	NEG	POS	POS	NEG	POS	POS	NEG	NEG	POS	NEG	POS	NEG	AMB	NEG	POS	NEG	NEG	NEG	NEG	NEG	NEG	POS	NEG	POS	POS
UKD-14571328	SCCmec IVa (MW2)	CC1	POS	POS	POS	NEG	NEG	POS	POS	NEG	POS	POS	NEG	NEG	POS	NEG	POS	NEG	AMB	NEG	POS	NEG	NEG	NEG	NEG	NEG	NEG	POS	NEG	POS	POS
UKD-03003012	SCCmec IVa (MW2)	CC5	POS	POS	POS	NEG	NEG	POS	POS	NEG	POS	POS	NEG	NEG	POS	NEG	POS	NEG	POS	POS	NEG	NEG	NEG	POS	AMB	NEG	POS	POS	POS	NEG	POS
UKD-15582582	SCCmec IVa (MW2)	CC6	POS	POS	POS	NEG	NEG	POS	POS	NEG	POS	POS	NEG	POS	NEG	NEG	POS	NEG	AMB	POS	NEG	NEG	NEG	POS	POS	NEG	POS	POS	NEG	POS	POS
UKD-16574230	SCCmec IVa (MW2)	CC6	POS	POS	POS	NEG	NEG	POS	POS	NEG	POS	POS	NEG	POS	NEG	NEG	POS	NEG	AMB	POS	NEG	NEG	NEG	POS	AMB	NEG	POS	POS	NEG	POS	POS
UKD-16582560	SCCmec IVa (MW2)	CC6	POS	AMB	POS	NEG	NEG	POS	POS	NEG	POS	POS	NEG	POS	NEG	NEG	POS	NEG	AMB	POS	NEG	NEG	NEG	POS	NEG	NEG	POS	POS	NEG	POS	POS
UKD-0802855	SCCmec IVa (MW2)	CC7	POS	POS	POS	NEG	POS	POS	POS	NEG	POS	POS	NEG	NEG	NEG	NEG	POS	NEG	POS	NEG	NEG	NEG	POS	POS	NEG	NEG	POS	NEG	NEG	NEG	NEG
KHDN-10405258	SCCmec IVa (MW2)	CC8	POS	POS	POS	NEG	POS	POS	POS	NEG	POS	POS	POS	NEG	NEG	NEG	POS	POS	AMB	AMB	NEG	NEG	NEG	POS	POS	NEG	NEG	POS	POS	NEG	POS
UKD-15573497	SCCmec IVa (MW2)	CC22	POS	POS	POS	NEG	POS	POS	NEG	POS	POS	POS	NEG	NEG	POS	NEG	POS	NEG	AMB	NEG	POS	NEG	NEG	POS	AMB	NEG	POS	POS	NEG	POS	POS
UKD-0634729	SCCmec IVa (MW2)	CC30	POS	POS	POS	NEG	NEG	POS	NEG	POS	POS	POS	NEG	POS	NEG	NEG	POS	NEG	POS	POS	NEG	NEG	NEG	POS	AMB	POS	AMB	NEG	NEG	NEG	NEG
UKD-1119771	SCCmec IVa (MW2)	CC30	POS	POS	POS	NEG	NEG	POS	NEG	POS	POS	POS	NEG	POS	NEG	NEG	POS	NEG	AMB	POS	NEG	NEG	NEG	POS	NEG	POS	NEG	NEG	NEG	NEG	NEG
KHDN-10411458	SCCmec IVa (MW2)	CC30	POS	POS	POS	NEG	NEG	POS	NEG	POS	POS	POS	NEG	POS	NEG	NEG	POS	NEG	AMB	POS	NEG	NEG	NEG	POS	NEG	POS	NEG	NEG	NEG	NEG	NEG
KHDN-398609	SCCmec IVa (MW2)	CC30	POS	POS	POS	NEG	NEG	POS	NEG	POS	POS	POS	NEG	POS	NEG	NEG	POS	NEG	AMB	POS	NEG	NEG	NEG	POS	NEG	POS	NEG	NEG	NEG	NEG	NEG
KHDN-01081998	SCCmec IVa (MW2)	CC30	POS	POS	POS	NEG	NEG	POS	NEG	POS	POS	POS	NEG	POS	NEG	NEG	POS	NEG	AMB	POS	NEG	NEG	NEG	POS	NEG	POS	NEG	NEG	NEG	NEG	NEG
KHDN-10399223	SCCmec IVa (MW2)	CC30	POS	POS	POS	NEG	NEG	POS	NEG	POS	POS	POS	NEG	POS	NEG	NEG	POS	NEG	AMB	POS	NEG	NEG	NEG	POS	NEG	POS	NEG	NEG	NEG	NEG	NEG
KHDN-W261158	SCCmec IVa (MW2)	CC30	POS	POS	POS	NEG	NEG	POS	NEG	POS	POS	POS	NEG	POS	NEG	NEG	POS	NEG	AMB	POS	NEG	NEG	NEG	POS	NEG	POS	NEG	NEG	NEG	NEG	NEG
UKD-0002046	SCCmec IVa (MW2)	CC45 [agr I]	POS	NEG	POS	POS	NEG	POS	NEG	POS	POS	POS	NEG	NEG	POS	NEG	POS	NEG	AMB	POS	NEG	NEG	NEG	POS	NEG	POS	NEG	NEG	NEG	NEG	NEG
UKD-0029618	SCCmec IVa (MW2)	CC45 [agr I]	POS	NEG	POS	POS	NEG	POS	NEG	POS	POS	POS	NEG	NEG	POS	NEG	POS	NEG	POS	AMB	NEG	NEG	NEG	POS	NEG	POS	NEG	NEG	NEG	NEG	NEG
UKD-0043085	SCCmec IVa (MW2)	CC45 [agr I]	POS	NEG	POS	POS	NEG	POS	NEG	POS	POS	POS	NEG	NEG	POS	NEG	POS	NEG	POS	AMB	NEG	NEG	NEG	POS	NEG	POS	NEG	NEG	NEG	NEG	NEG
UKD-0047053	SCCmec IVa (MW2)	CC45 [agr I]	POS	NEG	POS	POS	NEG	POS	NEG	POS	POS	POS	NEG	NEG	POS	NEG	POS	NEG	POS	POS	NEG	NEG	NEG	POS	NEG	POS	NEG	NEG	NEG	NEG	NEG
UKD-0047429	SCCmec IVa (MW2)	CC45 [agr I]	POS	NEG	POS	POS	NEG	POS	NEG	POS	POS	POS	NEG	NEG	POS	NEG	POS	NEG	POS	AMB	NEG	AMB	NEG	POS	NEG	POS	NEG	NEG	NEG	NEG	NEG
UKD-0112465	SCCmec IVa (MW2)	CC45 [agr I]	POS	NEG	POS	POS	NEG	POS	NEG	POS	POS	POS	NEG	NEG	POS	NEG	POS	NEG	POS	AMB	NEG	NEG	NEG	POS	NEG	POS	NEG	NEG	NEG	NEG	NEG
UKD-0103483	SCCmec IVa (MW2)	CC45 [agr I]	POS	NEG	POS	POS	NEG	POS	NEG	AMB	POS	POS	NEG	NEG	POS	NEG	POS	NEG	AMB	POS	NEG	NEG	NEG	POS	NEG	POS	NEG	NEG	NEG	NEG	NEG
UKD-0305014	SCCmec IVa (MW2)	CC45 [agr I]	POS	NEG	POS	POS	NEG	POS	NEG	POS	POS	POS	NEG	NEG	POS	NEG	POS	NEG	POS	AMB	NEG	AMB	NEG	POS	NEG	POS	NEG	NEG	NEG	NEG	NEG
UKD-0401119	SCCmec IVa (MW2)	CC45 [agr I]	POS	NEG	POS	POS	NEG	POS	NEG	POS	POS	POS	NEG	NEG	POS	NEG	POS	NEG	POS	AMB	NEG	NEG	NEG	POS	NEG	POS	NEG	NEG	NEG	NEG	NEG
UKD-0537969	SCCmec IVa (MW2)	CC45 [agr I]	POS	NEG	POS	AMB	NEG	POS	NEG	POS	POS	POS	NEG	NEG	POS	NEG	POS	NEG	AMB	POS	NEG	NEG	NEG	POS	NEG	POS	NEG	NEG	NEG	NEG	NEG
UKD-0703373	SCCmec IVa (MW2)	CC45 [agr I]	POS	NEG	POS	AMB	NEG	POS	NEG	POS	POS	POS	NEG	NEG	POS	NEG	NEG	NEG	AMB	AMB	NEG	NEG	NEG	POS	NEG	POS	NEG	NEG	NEG	NEG	NEG
UKD-0813653	SCCmec IVa (MW2)	CC45 [agr I]	POS	NEG	POS	POS	NEG	POS	NEG	POS	POS	POS	NEG	NEG	POS	NEG	POS	NEG	POS	AMB	NEG	AMB	NEG	POS	NEG	POS	NEG	NEG	NEG	NEG	NEG
UKD-0839175	SCCmec IVa (MW2)	CC45 [agr I]	POS	NEG	POS	POS	POS	POS	NEG	POS	POS	POS	NEG	NEG	POS	NEG	POS	NEG	POS	AMB	NEG	AMB	NEG	POS	AMB	POS	POS	NEG	NEG	NEG	NEG
UKD-1108422	SCCmec IVa (MW2)	CC45 [agr I]	POS	NEG	POS	POS	NEG	POS	NEG	POS	POS	POS	NEG	NEG	POS	NEG	POS	NEG	AMB	AMB	NEG	POS	NEG	POS	NEG	POS	NEG	NEG	NEG	NEG	NEG
UKD-1380645	SCCmec IVa (MW2)	CC45 [agr I]	POS	NEG	POS	POS	NEG	POS	NEG	POS	POS	POS	NEG	NEG	POS	NEG	NEG	NEG	NEG	NEG	NEG	NEG	NEG	POS	NEG	POS	NEG	NEG	NEG	NEG	NEG
UKD-148016	SCCmec IVa (MW2)	CC45 [agr I]	POS	NEG	POS	POS	NEG	POS	NEG	POS	POS	POS	NEG	NEG	POS	NEG	NEG	NEG	NEG	NEG	NEG	NEG	NEG	POS	POS	POS	POS	NEG	NEG	NEG	NEG
UKD-16571773	SCCmec IVa (MW2)	CC45 [agr I]	POS	NEG	POS	POS	AMB	POS	NEG	POS	POS	POS	NEG	NEG	POS	NEG	NEG	NEG	NEG	NEG	NEG	NEG	NEG	POS	NEG	POS	NEG	NEG	NEG	NEG	NEG
KHDN-10407738	SCCmec IVa (MW2)	CC45 [ST617]	POS	NEG	POS	POS	NEG	POS	NEG	POS	POS	POS	NEG	POS	NEG	NEG	POS	NEG	AMB	NEG	NEG	NEG	POS	POS	NEG	POS	NEG	POS	NEG	POS	POS
UKD-0853670	SCCmec IVa (MW2)	CC88	POS	POS	POS	NEG	POS	POS	POS	NEG	POS	POS	NEG	NEG	NEG	NEG	POS	NEG	POS	AMB	NEG	NEG	NEG	POS	NEG	NEG	POS	POS	NEG	POS	POS
UKD-1417352	SCCmec IVa (MW2)	CC88	POS	POS	POS	NEG	POS	POS	POS	NEG	POS	POS	NEG	NEG	NEG	NEG	POS	NEG	AMB	POS	NEG	NEG	NEG	POS	POS	NEG	POS	POS	NEG	POS	POS
UKD-15571283	SCCmec IVa (MW2)	CC88	POS	POS	POS	NEG	POS	POS	POS	POS	POS	POS	NEG	NEG	NEG	NEG	POS	NEG	AMB	POS	NEG	NEG	NEG	POS	POS	POS	POS	POS	NEG	POS	POS
KHDN-10406719	SCCmec IVa (MW2)	ST93	POS	POS	POS	NEG	POS	POS	NEG	POS	POS	POS	NEG	NEG	NEG	NEG	POS	NEG	AMB	POS	NEG	NEG	NEG	NEG	NEG	NEG	NEG	NEG	NEG	NEG	NEG
KHDN-0060294	SCCmec IVa (MW2)	ST93	POS	POS	POS	NEG	NEG	POS	NEG	POS	POS	POS	NEG	NEG	NEG	NEG	POS	NEG	POS	AMB	NEG	NEG	NEG	NEG	NEG	NEG	NEG	NEG	NEG	NEG	NEG
UKD-0734190	SCCmec IVa (MW2)	CC97	POS	POS	POS	NEG	NEG	POS	POS	NEG	POS	POS	NEG	NEG	NEG	NEG	POS	NEG	POS	POS	NEG	NEG	NEG	POS	POS	NEG	POS	POS	POS	NEG	POS
UKD-13006983	SCCmec IVa (MW2)	CC97	POS	POS	POS	NEG	NEG	POS	POS	AMB	POS	POS	NEG	NEG	NEG	NEG	POS	NEG	AMB	POS	NEG	NEG	NEG	POS	POS	POS	POS	POS	POS	NEG	POS
Prediction for H131520133	*SCCmec IVa (H131520133)*	*CC152*	POS	POS	POS	NEG	NEG	POS	NEG	NEG	POS	POS	NEG	NEG	NEG	NEG	POS	NEG	AMB	AMB	NEG	NEG	NEG	NEG	NEG	NEG	NEG	NEG	NEG	NEG	NEG
KHDN-325892	SCCmec IVa (H131520133)	CC30	POS	POS	POS	NEG	NEG	POS	NEG	POS	POS	POS	NEG	POS	NEG	NEG	POS	NEG	AMB	POS	NEG	NEG	NEG	POS	NEG	POS	NEG	NEG	NEG	NEG	NEG
UKD-15580207	SCCmec IVa (H131520133)	CC88	POS	POS	POS	NEG	POS	POS	POS	AMB	POS	POS	NEG	NEG	NEG	NEG	POS	NEG	AMB	POS	NEG	NEG	NEG	POS	POS	NEG	POS	POS	NEG	POS	POS
UKD-0521159	SCCmec Iva	CC8	POS	POS	POS	NEG	POS	POS	POS	NEG	POS	POS	POS	NEG	NEG	NEG	POS	POS	POS	NEG	NEG	NEG	NEG	POS	POS	NEG	NEG	POS	POS	NEG	POS
Prediction for CMFT503 : HF569113.1	*SCCmec IVa (CMFT503)*																														
Prediction for 21343, GenBank AHKV	*SCCmec IVa (CMFT503)*	*CC88*	POS	POS	POS	NEG	POS	POS	POS	NEG	POS	POS	NEG	NEG	NEG	NEG	POS	NEG	POS	AMB	NEG	NEG	NEG	POS	AMB	NEG	POS	POS	NEG	POS	POS
UKD-1420740	SCCmec IVa (CMFT503)	CC22	POS	POS	POS	NEG	POS	POS	NEG	POS	POS	POS	NEG	NEG	POS	NEG	POS	NEG	NEG	NEG	POS	NEG	NEG	POS	POS	AMB	POS	POS	NEG	POS	POS
UKD-15575124	SCCmec IVa (CMFT503)	CC22	POS	POS	POS	NEG	POS	POS	NEG	POS	POS	POS	NEG	NEG	POS	NEG	NEG	NEG	NEG	NEG	NEG	NEG	NEG	POS	NEG	NEG	POS	POS	NEG	POS	POS
UKD-16584947	SCCmec IVa (CMFT503)	CC22	POS	POS	POS	NEG	POS	POS	NEG	POS	POS	POS	NEG	NEG	POS	NEG	NEG	NEG	NEG	NEG	NEG	NEG	NEG	POS	NEG	NEG	POS	POS	NEG	POS	POS
UKD-16575077	SCCmec IVa (CMFT503)	CC88	POS	POS	POS	NEG	POS	POS	POS	NEG	POS	POS	NEG	NEG	NEG	NEG	POS	NEG	AMB	POS	NEG	NEG	NEG	POS	NEG	NEG	POS	POS	NEG	POS	POS
Prediction for TCH1516, GenBank CP000730.1	*SCCmec IVa+ACME1+Cu (USA300)*	*CC8*	POS	POS	POS	NEG	POS	POS	POS	NEG	POS	POS	POS	NEG	NEG	NEG	POS	AMB	AMB	NEG	NEG	NEG	NEG	POS	POS	NEG	NEG	POS	POS	NEG	POS
Reference strain USA300-TCH1516	SCCmec IVa+ACME1+Cu (USA300)	CC8	POS	POS	POS	NEG	POS	POS	POS	NEG	POS	POS	POS	NEG	NEG	NEG	POS	POS	AMB	AMB	NEG	NEG	NEG	POS	POS	NEG	AMB	POS	POS	NEG	POS
Prediction for FPR3757, GenBank CP000255.1	*SCCmec IVa+ACME1+Cu (USA300)*	*CC8*	POS	POS	POS	NEG	POS	POS	POS	NEG	POS	POS	POS	NEG	NEG	NEG	POS	AMB	AMB	NEG	NEG	NEG	NEG	POS	POS	NEG	NEG	POS	POS	NEG	POS
UKD-0516386	SCCmec IVa+ACME1+Cu (USA300)	CC8	POS	POS	POS	NEG	POS	POS	POS	NEG	POS	POS	POS	NEG	NEG	NEG	POS	POS	POS	NEG	NEG	NEG	NEG	POS	POS	NEG	NEG	POS	POS	NEG	POS
UKD-0815773	SCCmec IVa+ACME1+Cu (USA300)	CC8	POS	POS	POS	NEG	POS	POS	POS	NEG	POS	POS	POS	NEG	NEG	NEG	POS	AMB	POS	NEG	NEG	NEG	NEG	POS	POS	NEG	NEG	POS	POS	NEG	POS
UKD-1414654	SCCmec IVa+ACME1+Cu (USA300)	CC8	POS	POS	POS	NEG	POS	POS	POS	AMB	POS	POS	POS	NEG	NEG	NEG	POS	POS	AMB	AMB	NEG	NEG	NEG	POS	POS	NEG	POS	POS	POS	NEG	POS
UKD-0408036	SCCmec IVa+ACME2	CC45 [agr I]	POS	NEG	POS	POS	NEG	POS	NEG	POS	POS	POS	NEG	NEG	POS	NEG	POS	NEG	POS	AMB	NEG	AMB	NEG	POS	NEG	POS	NEG	NEG	NEG	NEG	NEG
UKD-0044035	SCCmec IVa+ccrC (Hannover)	CC8	POS	POS	POS	NEG	POS	POS	POS	NEG	POS	POS	POS	NEG	NEG	NEG	POS	POS	POS	NEG	NEG	NEG	NEG	POS	POS	NEG	NEG	POS	POS	NEG	POS
UKD-0136287	SCCmec IVa+ccrC (Hannover)	CC8	POS	POS	POS	NEG	POS	POS	POS	NEG	POS	POS	POS	NEG	NEG	NEG	POS	POS	AMB	AMB	NEG	NEG	NEG	POS	POS	NEG	NEG	POS	POS	NEG	POS

Isolate ID	SCCmec type	Clonal complex	ADHAESION FACTORS / GENES ENCODING MICROBIAL SURFACE COMPONENTS RECOGNIZING ADHESIVE MATRIX MOLECULES (MSCRAMM GENES)																												
			cell surface elastin binding protein					enolase	fibrinogen binding protein (19 kDa)		fibronectin-binding protein A						fibronectin-binding protein B							Major histocompatibility complex class II analog protein (=Extracellular adherence protein, eap)				Staphylococcus aureus surface protein G			
			ebpS	ebpS_probe 612	ebpS_probe 614	ebpS (01-1111)	ebpS (COL)	eno	efb / fib	efb / fib (MRSA252)	fnbA	fnbA (cons)	fnbA (COL)	fnbA (MRSA252)	fnbA (Mu50+MW2)	fnbA (RF122)	fnbB	fnbB (COL)	fnbB (COL+Mu50+MW2)	fnbB (Mu50)	fnbB (MW2)	fnbB (ST15)	fnbB (ST45-2)	map	map (COL)	map (MRSA252)	map (Mu50+MW2)	sasG	sasG (COL+Mu50)	sasG (MW2)	sasG (OtherThan252+122)
Prediction for JCSC1978, GenBank AB063173.1	*SCCmec IVb/d/i (JCSC1978/6668/4469)*																														
Reference strain JCSC1978 (8/6-3P)	SCCmec IVb/d/i (JCSC1978/6668/4469)	CC8	POS	POS	POS	NEG	POS	POS	POS	NEG	POS	POS	POS	NEG	NEG	NEG	POS	POS	AMB	AMB	NEG	NEG	NEG	POS	POS	NEG	AMB	POS	POS	NEG	POS
Prediction for JCSC6668, GenBank AB425823.1	*SCCmec IVb/d/i (JCSC1978/6668/4469)*																														
Reference strain JCSC4469	SCCmec IVb/d/i (JCSC1978/6668/4469)	CC5	POS	POS	POS	NEG	NEG	POS	POS	NEG	POS	POS	NEG	NEG	POS	NEG	POS	NEG	AMB	POS	NEG	AMB	NEG	POS	POS	NEG	POS	POS	POS	NEG	POS
UKD-1103429	SCCmec IVb/d/i (JCSC1978/6668/4469)	CC7	POS	POS	POS	NEG	POS	POS	POS	NEG	POS	POS	NEG	NEG	NEG	NEG	POS	NEG	AMB	NEG	NEG	NEG	POS	POS	NEG	NEG	POS	NEG	NEG	NEG	NEG
UKD-1306141	SCCmec IVb/d/i (JCSC1978/6668/4469)	CC7	POS	POS	POS	NEG	POS	POS	POS	NEG	POS	POS	NEG	NEG	NEG	NEG	POS	NEG	AMB	NEG	NEG	NEG	POS	POS	AMB	NEG	POS	NEG	NEG	NEG	NEG
UKD-0500089	SCCmec IVb/d/i (JCSC1978/6668/4469)	CC5	POS	POS	POS	NEG	NEG	POS	POS	NEG	POS	POS	NEG	NEG	POS	NEG	POS	NEG	POS	POS	NEG	NEG	NEG	POS	NEG	NEG	POS	POS	POS	NEG	POS
UKD-0501416	SCCmec IVb/d/i (JCSC1978/6668/4469)	CC5	POS	AMB	POS	NEG	NEG	POS	POS	NEG	POS	POS	NEG	NEG	POS	NEG	POS	NEG	POS	NEG	NEG	NEG	NEG	POS	NEG	NEG	POS	POS	POS	NEG	POS
UKD-0934574	SCCmec IVb/d/i (JCSC1978/6668/4469)	CC5	POS	POS	POS	NEG	NEG	POS	POS	NEG	POS	POS	NEG	NEG	POS	NEG	POS	NEG	POS	AMB	NEG	NEG	NEG	POS	NEG	NEG	POS	POS	POS	NEG	POS
UKD-0746779	SCCmec IVb/d/i (JCSC1978/6668/4469)	CC45 [agr I]	POS	NEG	POS	POS	AMB	POS	NEG	POS	POS	POS	NEG	NEG	POS	NEG	POS	NEG	POS	POS	NEG	POS	NEG	POS	AMB	POS	AMB	NEG	NEG	NEG	NEG
UKD-0812518	SCCmec IVb/d/i (JCSC1978/6668/4469)	CC45 [agr I]	POS	NEG	POS	POS	NEG	POS	NEG	POS	POS	POS	NEG	NEG	POS	NEG	POS	NEG	POS	AMB	NEG	NEG	NEG	POS	NEG	POS	NEG	NEG	NEG	NEG	NEG
Prediction for 21209, GenBank AGRP	*SCCmec IVb/d/i (21209)*	*CC8*	*POS*	*POS*	*POS*	*NEG*	*POS*	*POS*	*POS*	*NEG*	*POS*	*POS*	*POS*	*NEG*	*NEG*	*NEG*	*POS*	*AMB*	*AMB*	*NEG*	*NEG*	*NEG*	*NEG*	*POS*	*POS*	*NEG*	*NEG*	*POS*	*POS*	*NEG*	*POS*
UKD-0233579	SCCmec IVb/d/i (21209)	CC8	POS	POS	POS	NEG	POS	POS	POS	AMB	POS	POS	POS	NEG	NEG	NEG	POS	POS	POS	NEG	NEG	NEG	NEG	POS	POS	NEG	POS	POS	POS	AMB	POS
UKD-0504920	SCCmec IVb/d/i (21209)	CC8	POS	POS	POS	NEG	POS	POS	POS	NEG	POS	POS	POS	NEG	NEG	NEG	POS	POS	POS	NEG	NEG	NEG	NEG	POS	POS	NEG	NEG	POS	POS	NEG	POS
UKD-0725999	SCCmecIV b/d/i+Cu	CC30	POS	POS	POS	NEG	NEG	POS	NEG	POS	POS	POS	NEG	POS	NEG	NEG	POS	NEG	POS	POS	NEG	NEG	NEG	POS	NEG	POS	NEG	NEG	NEG	NEG	NEG
Prediction for IS-105, GenBank AHLR	*SCCmec IVc (IS-105)*	*CC22*	*POS*	*POS*	*POS*	*NEG*	*POS*	*POS*	*NEG*	*POS*	*POS*	*POS*	*NEG*	*NEG*	*POS*	*NEG*	*POS*	*NEG*	*AMB*	*NEG*	*AMB*	*NEG*	*NEG*	*POS*	*NEG*	*NEG*	*POS*	*POS*	*NEG*	*POS*	*POS*
UKD-0402421	SCCmec IVc (IS-105), merA/B+	CC5	POS	POS	POS	NEG	NEG	POS	POS	NEG	POS	POS	NEG	NEG	POS	NEG	POS	NEG	POS	POS	NEG	NEG	NEG	POS	AMB	NEG	POS	POS	POS	NEG	POS
UKD-0731319	SCCmec IVc (IS-105), merA/B+	CC5	POS	POS	POS	NEG	NEG	POS	POS	NEG	POS	POS	NEG	NEG	POS	NEG	POS	NEG	POS	POS	NEG	NEG	NEG	POS	POS	NEG	POS	POS	POS	NEG	POS
UKD-0733137	SCCmec IVc (IS-105)	CC8	POS	POS	POS	NEG	POS	POS	POS	NEG	POS	POS	POS	NEG	NEG	NEG	NEG	NEG	NEG	NEG	NEG	NEG	NEG	POS	POS	NEG	AMB	POS	POS	NEG	POS
UKD-0305690	SCCmec IVc (IS-105)	CC22	POS	POS	POS	NEG	POS	POS	NEG	POS	POS	POS	NEG	NEG	POS	NEG	POS	NEG	AMB	NEG	POS	NEG	NEG	POS	NEG	NEG	POS	POS	NEG	POS	POS
UKD-1424706	SCCmec IVc (IS-105)	CC22	POS	POS	POS	NEG	POS	POS	NEG	POS	POS	POS	NEG	NEG	POS	NEG	POS	NEG	AMB	NEG	POS	NEG	NEG	POS	AMB	NEG	POS	POS	NEG	POS	POS
Prediction for TCH60, GenBank CP002110.1	*SCCmec IVc (TCH60)*	*CC30*	*POS*	*POS*	*POS*	*NEG*	*NEG*	*POS*	*NEG*	*POS*	*POS*	*POS*	*NEG*	*POS*	*NEG*	*NEG*	*POS*	*NEG*	*AMB*	*AMB*	*NEG*	*NEG*	*NEG*	*POS*	*NEG*	*POS*	*NEG*	*NEG*	*NEG*	*NEG*	*NEG*
Reference strain JCSC4788	SCCmec IVc (TCH60)	CC8	POS	POS	POS	NEG	POS	POS	POS	NEG	POS	POS	POS	NEG	NEG	NEG	POS	POS	AMB	AMB	NEG	NEG	NEG	POS	POS	NEG	NEG	POS	POS	NEG	POS
UKD-1318318	SCCmec IVc (TCH60)	CC5	POS	POS	POS	NEG	NEG	POS	POS	NEG	POS	POS	NEG	NEG	POS	NEG	POS	NEG	AMB	POS	NEG	NEG	NEG	POS	POS	NEG	POS	POS	POS	NEG	POS
UKD-14580050	SCCmec IVc (TCH60)	CC5	POS	POS	POS	NEG	NEG	POS	POS	NEG	POS	POS	NEG	NEG	POS	NEG	POS	NEG	AMB	POS	NEG	AMB	NEG	POS	POS	NEG	POS	POS	POS	NEG	POS
UKD-08F01314	SCCmec IVc (TCH60)	CC8	POS	POS	POS	NEG	POS	POS	POS	NEG	POS	POS	POS	NEG	NEG	NEG	POS	AMB	POS	NEG	NEG	NEG	NEG	POS	POS	NEG	NEG	POS	POS	NEG	POS
KHDN-50361518	SCCmec IVc (TCH60)	CC8	POS	POS	POS	NEG	POS	POS	POS	NEG	POS	POS	POS	NEG	NEG	NEG	POS	POS	AMB	AMB	NEG	NEG	NEG	POS	POS	NEG	NEG	POS	POS	NEG	POS
UKD-0307912	SCCmec IVc (TCH60)	CC22	POS	POS	POS	NEG	POS	POS	NEG	POS	POS	POS	NEG	NEG	POS	NEG	POS	NEG	POS	AMB	POS	NEG	NEG	POS	NEG	NEG	POS	POS	NEG	POS	POS
UKD-0220390	SCCmec IVc (TCH60)	CC45 [agr I]	POS	NEG	POS	POS	NEG	POS	NEG	AMB	POS	POS	NEG	NEG	POS	NEG	POS	NEG	POS	AMB	NEG	NEG	NEG	POS	NEG	POS	NEG	NEG	NEG	NEG	NEG
UKD-0311106	SCCmec IVc (TCH60)	CC45 [agr I]	POS	NEG	POS	POS	NEG	POS	NEG	POS	POS	POS	NEG	NEG	POS	NEG	POS	NEG	AMB	POS	NEG	AMB	NEG	POS	NEG	POS	NEG	NEG	NEG	NEG	NEG
UKD-0719855	SCCmec IVc (TCH60)	CC45 [agr I]	POS	NEG	POS	AMB	NEG	POS	NEG	POS	POS	POS	NEG	NEG	POS	NEG	POS	NEG	POS	POS	NEG	NEG	NEG	POS	NEG	POS	NEG	NEG	NEG	NEG	NEG
UKD-0422079	SCCmec IVc (TCH60)	CC45 [agr I]	POS	NEG	POS	POS	NEG	POS	NEG	POS	POS	POS	NEG	NEG	POS	NEG	POS	NEG	POS	AMB	NEG	NEG	NEG	POS	NEG	POS	NEG	NEG	NEG	NEG	NEG
UKD-0409235	SCCmec IVc (TCH60)	CC80	POS	POS	POS	NEG	NEG	POS	POS	NEG	POS	POS	NEG	NEG	NEG	NEG	POS	NEG	AMB	NEG	POS	NEG	NEG	POS	NEG	NEG	POS	POS	NEG	POS	POS
UKD-0409807	SCCmec IVc (TCH60)	CC80	POS	POS	POS	NEG	NEG	POS	POS	AMB	POS	POS	NEG	NEG	NEG	NEG	POS	NEG	AMB	NEG	POS	NEG	NEG	POS	POS	AMB	POS	POS	NEG	POS	POS
UKD-0416073	SCCmec IVc (TCH60)	CC80	AMB	POS	POS	NEG	NEG	POS	POS	NEG	POS	POS	NEG	NEG	NEG	NEG	POS	NEG	POS	NEG	POS	NEG	NEG	POS	AMB	NEG	POS	POS	NEG	POS	POS
UKD-0431728	SCCmec IVc (TCH60)	CC80	POS	POS	POS	NEG	NEG	POS	POS	NEG	POS	POS	NEG	NEG	NEG	NEG	POS	NEG	AMB	NEG	POS	NEG	NEG	POS	NEG	NEG	POS	POS	NEG	POS	POS
UKD-1001508	SCCmec IVc (TCH60)	CC80	POS	POS	POS	NEG	NEG	POS	POS	NEG	POS	POS	NEG	NEG	NEG	NEG	POS	NEG	AMB	NEG	POS	NEG	NEG	POS	POS	NEG	POS	POS	NEG	POS	POS
UKD-1010412	SCCmec IVc (TCH60)	CC80	POS	POS	POS	NEG	NEG	POS	POS	NEG	POS	POS	NEG	NEG	NEG	NEG	POS	NEG	AMB	NEG	POS	NEG	NEG	POS	AMB	AMB	POS	POS	NEG	POS	POS
UKD-12753005	SCCmec IVc (TCH60)	CC80	POS	POS	POS	NEG	NEG	POS	POS	NEG	POS	POS	NEG	NEG	NEG	NEG	POS	NEG	AMB	NEG	POS	NEG	NEG	POS	AMB	NEG	POS	POS	NEG	POS	POS
UKD-15574809	SCCmec IVc (TCH60)	CC80	POS	POS	POS	NEG	NEG	POS	POS	NEG	POS	POS	NEG	NEG	NEG	NEG	POS	NEG	AMB	NEG	POS	NEG	NEG	POS	NEG	NEG	POS	POS	NEG	POS	POS
KHDN-16120291	SCCmec IVc (TCH60)	CC88	POS	POS	POS	NEG	POS	POS	POS	NEG	POS	POS	NEG	NEG	NEG	NEG	POS	NEG	POS	AMB	NEG	NEG	NEG	POS	NEG	NEG	POS	POS	NEG	POS	POS
Prediction for WW2703/97, GenBank ACSW	*SCCmec IVc (WW2703/97)*	*CC30*	*POS*	*POS*	*POS*	*NEG*	*NEG*	*POS*	*NEG*	*POS*	*POS*	*POS*	*NEG*	*POS*	*NEG*	*NEG*	*NEG*	*NEG*	*NEG*	*NEG*	*NEG*	*NEG*	*NEG*	*POS*	*NEG*	*POS*	*NEG*	*NEG*	*NEG*	*NEG*	*NEG*
UKD-14580088	SCCmec IVc (WW2703/97)	CC398	POS	NEG	POS	POS	POS	POS	NEG	POS	AMB	POS	NEG	NEG	NEG	NEG	POS	NEG	POS	NEG	NEG	NEG	NEG	POS	POS	POS	NEG	NEG	NEG	NEG	NEG
Prediction for SA_ST125_MupR, GenBank ASTH	*SCCmec IVc+speG+ccrAB4 (SA_ST125)*	*CC5*	*POS*	*POS*	*POS*	*NEG*	*NEG*	*POS*	*POS*	*NEG*	*POS*	*POS*	*NEG*	*NEG*	*POS*	*NEG*	*POS*	*NEG*	*AMB*	*AMB*	*NEG*	*NEG*	*NEG*	*POS*	*AMB*	*NEG*	*POS*	*POS*	*POS*	*NEG*	*POS*
UKD-0439909	SCCmec IVc+speG+ccrAB4 (SA_ST125)	CC5	POS	POS	POS	NEG	NEG	POS	POS	NEG	POS	POS	NEG	NEG	POS	NEG	POS	NEG	POS	POS	NEG	NEG	NEG	POS	NEG	NEG	POS	POS	POS	NEG	POS
Prediction for MRSA177, GenBank AECP	*SCCmec IVc+Cu/Hg (MRSA177)*		*POS*	*POS*	*POS*	*NEG*	*POS*	*POS*	*POS*	*NEG*	*POS*	*POS*	*POS*	*NEG*	*NEG*	*NEG*	*POS*	*AMB*	*AMB*	*NEG*	*NEG*	*NEG*	*NEG*	*POS*	*POS*	*NEG*	*NEG*	*POS*	*POS*	*NEG*	*POS*
UKD-1279734	SCCmec IVc+Cu/Hg (MRSA177)	CC8	POS	POS	POS	NEG	POS	POS	POS	NEG	POS	POS	POS	NEG	NEG	NEG	POS	POS	AMB	AMB	NEG	NEG	NEG	POS	POS	NEG	POS	POS	POS	NEG	POS
UKD-14580899	SCCmec IVc+Cu/Hg (MRSA177)	CC8	POS	POS	POS	NEG	POS	POS	POS	AMB	POS	POS	POS	NEG	NEG	NEG	POS	POS	AMB	AMB	NEG	NEG	NEG	POS	POS	NEG	POS	POS	POS	NEG	POS
UKD-16002228	SCCmec IVc+Cu/Hg (MRSA177)	CC8	POS	POS	POS	NEG	POS	POS	POS	NEG	POS	POS	POS	NEG	NEG	NEG	POS	POS	AMB	AMB	NEG	NEG	NEG	POS	POS	NEG	POS	POS	POS	NEG	POS
Prediction for HO 5096 0412, GenBank HE681097.1	*SCCmec IVh/j (HO50960412/JCSC6670)*	*CC22*	*POS*	*POS*	*POS*	*NEG*	*POS*	*POS*	*NEG*	*POS*	*POS*	*POS*	*NEG*	*NEG*	*POS*	*NEG*	*NEG*	*NEG*	*NEG*	*NEG*	*NEG*	*NEG*	*NEG*	*POS*	*NEG*	*NEG*	*POS*	*POS*	*NEG*	*POS*	*POS*
Prediction for JCSC6670, GenBank AB425824.1	*SCCmec IVh/j (HO50960412/JCSC6670)*																														
Reference strain Dublin_DSH_E1749	SCCmec IVh/j (HO50960412/JCSC6670)		POS	POS	POS	NEG	POS	POS	NEG	POS	POS	POS	NEG	NEG	POS	NEG	NEG	NEG	NEG	NEG	NEG	NEG	NEG	POS	NEG	NEG	POS	POS	NEG	POS	POS
UKD-14576248	SCCmec IVh/j (HO50960412/JCSC6670)	CC8	POS	POS	POS	NEG	POS	POS	POS	NEG	POS	POS	POS	NEG	NEG	NEG	POS	POS	AMB	AMB	NEG	NEG	NEG	POS	POS	NEG	POS	POS	POS	NEG	POS
UKD-0120122	SCCmec IVh/j (HO50960412/JCSC6670)	CC22	POS	NEG	POS	NEG	POS	POS	NEG	AMB	POS	POS	NEG	NEG	POS	NEG	NEG	NEG	NEG	NEG	NEG	NEG	NEG	POS	NEG	NEG	POS	POS	NEG	POS	POS
UKD-0205319	SCCmec IVh/j (HO50960412/JCSC6670)	CC22	POS	POS	POS	NEG	POS	POS	NEG	POS	POS	POS	NEG	NEG	POS	NEG	NEG	NEG	NEG	NEG	NEG	NEG	NEG	POS	NEG	NEG	POS	POS	NEG	POS	POS
UKD-0221259	SCCmec IVh/j (HO50960412/JCSC6670)	CC22	POS	POS	POS	NEG	POS	POS	NEG	POS	POS	POS	NEG	NEG	POS	NEG	NEG	NEG	NEG	NEG	NEG	NEG	NEG	NEG	NEG	NEG	NEG	POS	NEG	POS	POS
UKD-0418654	SCCmec IVh/j (HO50960412/JCSC6670)	CC22	POS	POS	POS	NEG	POS	POS	NEG	POS	POS	POS	NEG	NEG	POS	NEG	NEG	NEG	NEG	NEG	NEG	NEG	NEG	POS	NEG	NEG	POS	POS	NEG	POS	POS
UKD-0429721	SCCmec IVh/j (HO50960412/JCSC6670)	CC22	POS	POS	POS	NEG	AMB	POS	NEG	POS	POS	POS	NEG	NEG	POS	NEG	NEG	NEG	NEG	NEG	NEG	NEG	NEG	NEG	NEG	NEG	NEG	POS	NEG	POS	POS
UKD-1116194	SCCmec IVh/j (HO50960412/JCSC6670)	CC22	POS	POS	POS	NEG	POS	POS	NEG	POS	POS	POS	NEG	NEG	POS	NEG	NEG	NEG	NEG	NEG	NEG	NEG	NEG	POS	NEG	NEG	POS	POS	NEG	POS	POS
UKD-1122367	SCCmec IVh/j (HO50960412/JCSC6670)	CC22	POS	POS	POS	NEG	POS	POS	NEG	POS	POS	POS	NEG	NEG	POS	NEG	NEG	NEG	NEG	NEG	NEG	NEG	NEG	POS	NEG	NEG	POS	POS	NEG	POS	POS
UKD-14012840	SCCmec IVh/j (HO50960412/JCSC6670)	CC22	POS	POS	POS	NEG	POS	POS	NEG	POS	POS	POS	NEG	NEG	POS	NEG	NEG	NEG	NEG	NEG	NEG	NEG	NEG	POS	NEG	NEG	POS	POS	NEG	POS	POS
UKD-14579833	SCCmec IVh/j (HO50960412/JCSC6670)	CC22	POS	POS	POS	NEG	POS	POS	NEG	POS	POS	POS	NEG	NEG	POS	NEG	NEG	NEG	NEG	NEG	NEG	NEG	NEG	POS	NEG	NEG	POS	POS	NEG	POS	POS
UKD-15579198	SCCmec IVh/j (HO50960412/JCSC6670)	CC22	POS	POS	POS	NEG	POS	POS	NEG	POS	POS	POS	NEG	NEG	POS	NEG	NEG	NEG	NEG	NEG	NEG	NEG	NEG	POS	AMB	NEG	POS	POS	NEG	POS	POS
UKD-4052193	SCCmec IVh/j (HO50960412/JCSC6670)	CC22	POS	POS	POS	NEG	POS	POS	NEG	POS	POS	POS	NEG	NEG	POS	NEG	AMB	NEG	NEG	POS	NEG	NEG	NEG	POS	NEG	NEG	POS	POS	NEG	POS	POS
UKD-124968	SCCmec IVh/j (HO50960412/JCSC6670)	CC22	POS	POS	POS	NEG	POS	POS	NEG	POS	POS	POS	NEG	NEG	POS	NEG	NEG	NEG	NEG	NEG	NEG	NEG	NEG	POS	NEG	NEG	POS	POS	NEG	POS	POS
UKD-14572546	SCCmec IVh/j (HO50960412/JCSC6670)	CC22	POS	POS	POS	NEG	POS	POS	NEG	POS	POS	POS	NEG	NEG	POS	NEG	POS	NEG	NEG	NEG	POS	NEG	NEG	POS	POS	NEG	POS	POS	NEG	POS	POS
UKD-14582287	SCCmec IVh/j (HO50960412/JCSC6670)	CC22	POS	POS	POS	NEG	POS	POS	NEG	POS	POS	POS	NEG	NEG	POS	NEG	POS	NEG	NEG	NEG	POS	NEG	NEG	POS	NEG	NEG	POS	POS	NEG	POS	POS
UKD-14583376	SCCmec IVh/j (HO50960412/JCSC6670)	CC22	POS	POS	POS	NEG	POS	POS	NEG	POS	POS	POS	NEG	NEG	POS	NEG	POS	NEG	NEG	NEG	POS	NEG	NEG	POS	POS	NEG	POS	POS	NEG	POS	POS
UKD-14583496	SCCmec IVh/j (HO50960412/JCSC6670)	CC22	POS	POS	POS	NEG	POS	POS	NEG	POS	POS	POS	NEG	NEG	POS	NEG	POS	NEG	NEG	NEG	POS	NEG	NEG	POS	POS	NEG	POS	POS	NEG	POS	POS
UKD-0013324	SCCmec IVh/j (HO50960412/JCSC6670)	CC22	POS	AMB	POS	NEG	POS	POS	NEG	POS	POS	POS	NEG	NEG	POS	NEG	POS	NEG	AMB	NEG	POS	NEG	NEG	POS	NEG	NEG	POS	POS	NEG	POS	POS
UKD-0125329	SCCmec IVh/j (HO50960412/JCSC6670)	CC22	POS	POS	POS	NEG	POS	POS	NEG	POS	POS	POS	NEG	NEG	POS	NEG	POS	NEG	AMB	NEG	POS	NEG	NEG	POS	NEG	NEG	POS	POS	NEG	POS	POS
UKD-0215498	SCCmec IVh/j (HO50960412/JCSC6670)	CC22	POS	POS	POS	NEG	POS	POS	NEG	POS	POS	POS	NEG	NEG	POS	NEG	POS	NEG	AMB	NEG	POS	NEG	NEG	NEG	NEG	NEG	NEG	POS	NEG	POS	POS
Prediction for M08-0126, GenBank FR753166.1	*SCCmec IVh/j+ACME2 (M08-0126)*	*CC22*																													
UKD-1379483I	SCCmec IVh/j+ACME2 (M08-0126)	CC22	POS	POS	POS	NEG	POS	POS	NEG	POS	POS	POS	NEG	NEG	POS	NEG	NEG	NEG	NEG	NEG	NEG	NEG	NEG	POS	AMB	NEG	POS	POS	NEG	POS	POS
UKD-5150204	SCCmec IV+speG+Cu/czrC+ccrAB4	CC22	POS	POS	POS	NEG	POS	POS	NEG	POS	POS	POS	NEG	NEG	POS	NEG	POS	NEG	AMB	NEG	POS	NEG	NEG	POS	AMB	NEG	POS	POS	NEG	POS	POS
UKD-15578423	SCCmec IV+speG+czrC+ccrAB4 var. 1	CC22	POS	POS	POS	NEG	POS	POS	NEG	POS	POS	POS	NEG	NEG	POS	NEG	NEG	NEG	NEG	NEG	NEG	NEG	NEG	POS	NEG	NEG	POS	POS	NEG	POS	POS
UKD-16582870	SCCmec IV+speG+czrC+ccrAB4 var. 1	CC22	POS	POS	POS	NEG	POS	POS	NEG	POS	POS	POS	NEG	NEG	POS	NEG	NEG	NEG	NEG	NEG	NEG	NEG	NEG	POS	NEG	NEG	POS	POS	NEG	POS	POS
UKD-15571940	SCCmec IV+speG+czrC+ccrAB4 var. 2	CC22	POS	POS	POS	NEG	POS	POS	NEG	POS	POS	POS	NEG	NEG	POS	NEG	NEG	NEG	NEG	NEG	NEG	NEG	NEG	POS	NEG	NEG	POS	POS	NEG	POS	POS

Isolate ID	SCCmec type	Clonal complex	ADHAESION FACTORS / GENES ENCODING MICROBIAL SURFACE COMPONENTS RECOGNIZING ADHESIVE MATRIX MOLECULES (MSCRAMM GENES)																												
			cell surface elastin binding protein					enolase	fibrinogen binding protein (19 kDa)		fibronectin-binding protein A						fibronectin-binding protein B							Major histocompatibility complex class II analog protein (=Extracellular adherence protein, eap)				Staphylococcus aureus surface protein G			
			ebpS	ebpS_probe 612	ebpS_probe 614	ebpS (01-1111)	ebpS (COL)	eno	efb / fib	efb / fib (MRSA252)	fnbA	fnbA (cons)	fnbA (COL)	fnbA (MRSA252)	fnbA (Mu50+MW2)	fnbA (RF122)	fnbB	fnbB (COL)	fnbB (COL+Mu50+MW2)	fnbB (Mu50)	fnbB (MW2)	fnbB (ST15)	fnbB (ST45-2)	map	map (COL)	map (MRSA252)	map (Mu50+MW2)	sasG	sasG (COL+Mu50)	sasG (MW2)	sasG (OtherThan252+122)
Prediction for CN1, GenBank CP003979.1	*SCCmec IVA (CN1)*	*CC8 (ST72)*	*POS*	*POS*	*POS*	*NEG*	*NEG*	*POS*	*POS*	*NEG*	*POS*	*POS*	*POS*	*NEG*	*NEG*	*NEG*	*POS*	*NEG*	*POS*	*NEG*	*NEG*	*NEG*	*NEG*	*POS*	*AMB*	*NEG*	*POS*	*POS*	*NEG*	*POS*	*POS*
UKD-0900871	SCCmec IVA (CN1)	CC8 (ST72)	POS	POS	POS	NEG	NEG	POS	POS	NEG	POS	POS	POS	NEG	NEG	NEG	POS	NEG	POS	AMB	NEG	NEG	NEG	POS	NEG	NEG	POS	POS	NEG	POS	POS
Prediction for CMFT1723, GenBank HF569096.1	*SCCmec V (Bengal Bay)*																														
KHDN-10405134	SCCmec V (Bengal Bay)	CC1	POS	POS	POS	NEG	NEG	POS	POS	NEG	POS	POS	NEG	NEG	POS	NEG	POS	NEG	AMB	NEG	POS	NEG	NEG	POS	NEG	NEG	POS	POS	NEG	POS	POS
UKD-0932405	SCCmec V (Bengal Bay)	CC1 (ST573/772)	POS	POS	POS	NEG	NEG	POS	POS	NEG	POS	POS	NEG	NEG	POS	NEG	POS	NEG	POS	NEG	NEG	AMB	NEG	POS	NEG	NEG	POS	POS	NEG	POS	POS
UKD-1003510	SCCmec V (Bengal Bay)	CC1 (ST573/772)	POS	POS	POS	NEG	NEG	POS	POS	AMB	POS	POS	NEG	NEG	POS	NEG	POS	NEG	AMB	AMB	NEG	POS	NEG	POS	AMB	NEG	POS	POS	NEG	POS	POS
UKD-1112420	SCCmec V (Bengal Bay)	CC1 (ST573/772)	POS	POS	POS	NEG	NEG	POS	POS	NEG	POS	POS	NEG	NEG	POS	NEG	POS	NEG	AMB	AMB	NEG	POS	NEG	POS	POS	NEG	POS	POS	NEG	POS	POS
UKD-1275832	SCCmec V (Bengal Bay)	CC1 (ST573/772)	POS	POS	POS	NEG	NEG	POS	POS	NEG	POS	POS	NEG	NEG	POS	NEG	POS	NEG	AMB	AMB	NEG	POS	NEG	POS	POS	AMB	POS	POS	NEG	POS	POS
UKD-13023811	SCCmec V (Bengal Bay)	CC1 (ST573/772)	POS	POS	POS	NEG	NEG	POS	POS	NEG	POS	POS	NEG	NEG	POS	NEG	POS	NEG	POS	AMB	NEG	AMB	NEG	POS	AMB	NEG	POS	POS	NEG	POS	POS
UKD-14572163	SCCmec V	CC5	POS	POS	POS	NEG	NEG	POS	POS	POS	POS	POS	NEG	NEG	POS	NEG	POS	NEG	AMB	POS	NEG	AMB	NEG	POS	POS	NEG	POS	POS	POS	NEG	POS
KHDN-10421133	SCCmec V+fus	CC97	POS	POS	POS	NEG	NEG	POS	POS	NEG	POS	POS	NEG	NEG	NEG	NEG	POS	NEG	AMB	POS	NEG	NEG	NEG	POS	POS	AMB	POS	POS	POS	NEG	POS
KHDN-21343541	SCCmec V+fus+tir+ccrAB1	CC1	POS	POS	POS	NEG	NEG	POS	POS	NEG	POS	POS	NEG	NEG	POS	NEG	POS	NEG	AMB	NEG	POS	NEG	NEG	POS	AMB	NEG	POS	POS	NEG	POS	POS
KHDN-10416893	SCCmec V+czrC+speG+ccrAB4	CC22	POS	POS	POS	NEG	POS	POS	NEG	POS	POS	POS	NEG	NEG	POS	NEG	NEG	NEG	NEG	NEG	NEG	NEG	NEG	POS	NEG	NEG	POS	POS	NEG	POS	POS
Prediction for GR1, GenBank AJLX	*SCCmec VT (GR1)*	*CC361*	*POS*	*POS*	*POS*	*NEG*	*POS*	*POS*	*POS*	*NEG*	*POS*	*POS*	*NEG*	*NEG*	*NEG*	*NEG*	*POS*	*NEG*	*POS*	*NEG*	*NEG*	*AMB*	*NEG*	*POS*	*AMB*	*NEG*	*POS*	*POS*	*NEG*	*POS*	*POS*
UKD-0422872	SCCmec VT (GR1)	CC152	POS	POS	POS	NEG	NEG	POS	NEG	POS	POS	POS	NEG	NEG	POS	NEG	POS	NEG	AMB	POS	NEG	NEG	NEG	NEG	NEG	NEG	NEG	NEG	NEG	AMB	NEG
Prediction for PM1, GenBank BAFA	*SCCmec VT (PM1)*	*CC59*	*POS*	*POS*	*POS*	*NEG*	*POS*	*POS*	*POS*	*NEG*	*POS*	*POS*	*NEG*	*POS*	*NEG*	*NEG*	*POS*	*NEG*	*POS*	*NEG*	*NEG*	*NEG*	*NEG*	*POS*	*POS*	*NEG*	*NEG*	*POS*	*NEG*	*POS*	*POS*
KHDN-000871	SCCmec VT (PM1)	CC45 (agr I)	POS	NEG	POS	POS	NEG	POS	NEG	POS	POS	POS	NEG	NEG	POS	NEG	POS	NEG	POS	AMB	NEG	AMB	NEG	POS	NEG	POS	NEG	NEG	NEG	NEG	NEG
UKD-0725052	SCCmec VT (PM1)	CC59	POS	POS	AMB	NEG	POS	POS	POS	NEG	POS	POS	NEG	POS	NEG	NEG	POS	NEG	POS	AMB	NEG	NEG	NEG	POS	POS	NEG	NEG	POS	NEG	POS	POS
UKD-1312138	SCCmec VT (PM1)	CC59	POS	POS	POS	NEG	POS	POS	POS	AMB	POS	POS	NEG	POS	NEG	NEG	POS	NEG	POS	AMB	NEG	NEG	NEG	POS	POS	NEG	POS	POS	NEG	POS	POS
UKD-Z4728	SCCmec VT (PM1)	CC59	POS	POS	POS	NEG	POS	POS	POS	NEG	POS	POS	NEG	POS	NEG	NEG	POS	NEG	POS	AMB	NEG	NEG	NEG	POS	POS	NEG	POS	POS	NEG	POS	POS
UKD-K0199	SCCmec VT unknown var.1	CC7	POS	POS	POS	NEG	POS	POS	POS	NEG	POS	POS	NEG	NEG	POS	NEG	POS	NEG	AMB	NEG	NEG	NEG	POS	POS	NEG	NEG	POS	NEG	NEG	NEG	NEG
UKD-15584619	SCCmec VT unknown var.2	CC30	POS	POS	POS	NEG	NEG	POS	NEG	POS	POS	POS	NEG	POS	NEG	NEG	NEG	NEG	NEG	NEG	NEG	NEG	NEG	POS	NEG	POS	POS	NEG	NEG	NEG	NEG
UKD-0712568	SCCmec VT unknown var.3	CC45 (agr I)	POS	NEG	POS	POS	NEG	POS	NEG	POS	POS	POS	NEG	NEG	POS	NEG	POS	NEG	POS	POS	NEG	NEG	NEG	POS	NEG	POS	NEG	NEG	NEG	NEG	NEG
KHDN-10420296	SCCmec VT+ACME2	CC45 (agr I)	POS	NEG	POS	POS	NEG	POS	NEG	POS	POS	POS	NEG	NEG	POS	NEG	POS	NEG	POS	AMB	NEG	AMB	NEG	POS	AMB	POS	AMB	NEG	NEG	NEG	NEG
UKD-15577956	SCCmec VT+Cu/czrC+ccrA1	CC398	POS	NEG	POS	POS	POS	POS	NEG	POS	POS	POS	NEG	NEG	NEG	NEG	POS	NEG	POS	AMB	NEG	NEG	NEG	POS	POS	POS	AMB	NEG	NEG	AMB	NEG
Prediction for S0385, GenBank AM990992	*SCCmec VT+czrC (SO385)*	*CC398*	*POS*	*NEG*	*POS*	*POS*	*POS*	*AMB*	*NEG*	*POS*	*NEG*	*AMB*	*NEG*	*NEG*	*NEG*	*NEG*	*POS*	*NEG*	*POS*	*NEG*	*NEG*	*NEG*	*NEG*	*NEG*	*NEG*	*AMB*	*NEG*	*NEG*	*NEG*	*NEG*	*NEG*
UKD-0628560	SCCmec VT+czrC (SO385)	CC8	POS	POS	POS	NEG	POS	POS	POS	NEG	POS	POS	POS	NEG	NEG	NEG	POS	POS	POS	NEG	NEG	NEG	NEG	POS	POS	NEG	NEG	POS	POS	NEG	POS
UKD-0510018	SCCmec VT+czrC (SO385)	CC398	POS	NEG	POS	AMB	POS	POS	NEG	POS	AMB	AMB	NEG	NEG	NEG	NEG	POS	NEG	POS	NEG	NEG	NEG	NEG	POS	POS	POS	NEG	NEG	NEG	NEG	NEG
UKD-0903558	SCCmec VT+czrC (SO385)	CC398	POS	NEG	POS	POS	POS	POS	NEG	POS	POS	POS	NEG	NEG	NEG	NEG	POS	NEG	POS	NEG	NEG	NEG	NEG	POS	POS	POS	NEG	NEG	NEG	NEG	NEG
UKD-0929815	SCCmec VT+czrC (SO385)	CC398	POS	NEG	POS	POS	POS	POS	NEG	POS	POS	POS	NEG	NEG	NEG	NEG	POS	NEG	POS	NEG	NEG	NEG	NEG	POS	POS	POS	NEG	NEG	NEG	NEG	NEG
UKD-0907762	SCCmec VT+czrC (SO385)	CC398	POS	NEG	POS	AMB	POS	POS	NEG	POS	POS	POS	NEG	NEG	NEG	NEG	POS	NEG	POS	NEG	NEG	NEG	NEG	POS	POS	POS	NEG	NEG	NEG	NEG	NEG
UKD-1025105	SCCmec VT+czrC (SO385)	CC398	POS	NEG	POS	POS	POS	POS	NEG	POS	POS	POS	NEG	NEG	NEG	NEG	POS	NEG	POS	NEG	NEG	NEG	NEG	POS	POS	POS	NEG	NEG	NEG	NEG	NEG
UKD-11780898	SCCmec VT+czrC (SO385)	CC398	POS	NEG	POS	POS	POS	POS	NEG	POS	POS	POS	NEG	NEG	NEG	NEG	POS	NEG	POS	AMB	NEG	AMB	NEG	POS	POS	POS	AMB	NEG	NEG	NEG	NEG
UKD-1377009	SCCmec VT+czrC (SO385)	CC398	POS	NEG	POS	POS	POS	POS	NEG	POS	POS	POS	NEG	NEG	NEG	NEG	POS	NEG	POS	AMB	NEG	NEG	NEG	POS	POS	POS	NEG	NEG	NEG	NEG	NEG
UKD-143406	SCCmec VT+czrC (SO385)	CC398	POS	NEG	POS	POS	POS	POS	NEG	POS	POS	POS	NEG	NEG	NEG	NEG	POS	NEG	POS	AMB	NEG	AMB	NEG	POS	POS	POS	POS	NEG	NEG	AMB	NEG
UKD-1400363	SCCmec VT+czrC (SO385)	CC398	POS	NEG	POS	POS	POS	POS	NEG	POS	POS	POS	NEG	NEG	NEG	NEG	POS	NEG	POS	NEG	NEG	NEG	NEG	POS	POS	POS	NEG	NEG	NEG	NEG	NEG
UKD-14571852	SCCmec VT+czrC (SO385)	CC398	POS	NEG	POS	POS	POS	POS	NEG	POS	POS	POS	NEG	NEG	NEG	NEG	POS	NEG	POS	AMB	NEG	AMB	NEG	POS	POS	POS	POS	NEG	NEG	AMB	NEG
UKD-14582574	SCCmec VT+czrC (SO385)	CC398	POS	NEG	POS	POS	POS	POS	NEG	POS	POS	POS	NEG	NEG	NEG	NEG	POS	NEG	POS	AMB	NEG	NEG	NEG	POS	POS	POS	AMB	NEG	NEG	NEG	NEG
UKD-152108	SCCmec VT+czrC (SO385)	CC398	POS	NEG	POS	POS	POS	POS	NEG	POS	POS	POS	NEG	NEG	NEG	NEG	POS	NEG	POS	AMB	NEG	NEG	NEG	POS	POS	POS	NEG	NEG	NEG	NEG	NEG
UKD-15573321	SCCmec VT+czrC (SO385)	CC398	POS	NEG	POS	POS	POS	POS	NEG	POS	POS	POS	NEG	NEG	NEG	NEG	POS	NEG	POS	AMB	NEG	AMB	NEG	POS	POS	POS	POS	NEG	NEG	NEG	NEG
UKD-15584034	SCCmec VT+czrC , ydhK-neg. variant	CC398	POS	NEG	POS	POS	POS	POS	NEG	POS	POS	POS	NEG	NEG	NEG	NEG	POS	NEG	POS	AMB	NEG	NEG	NEG	POS	POS	POS	NEG	NEG	NEG	NEG	NEG
UKD-0932581	SCCmecVT+fus+czrC	CC22	POS	POS	POS	NEG	POS	POS	NEG	POS	POS	POS	NEG	NEG	POS	NEG	POS	NEG	AMB	NEG	POS	NEG	NEG	POS	NEG	NEG	POS	POS	NEG	POS	POS
UKD-1121994	SCCmecVT+fus+czrC	CC22	POS	POS	POS	NEG	POS	POS	NEG	POS	POS	POS	NEG	NEG	POS	NEG	POS	NEG	AMB	NEG	POS	NEG	NEG	POS	NEG	NEG	POS	POS	NEG	POS	POS
UKD-1272252	SCCmecVT+fus+czrC	CC22	POS	POS	POS	NEG	POS	POS	NEG	POS	POS	POS	NEG	NEG	POS	NEG	POS	NEG	AMB	NEG	POS	NEG	NEG	POS	NEG	NEG	POS	POS	NEG	POS	POS
Prediction for MRSA18	*SCCmec VI+fus (MRSA18)*																														
UKD-1103380	SCCmec VI+fus (MRSA18)	CC7	POS	POS	POS	NEG	POS	POS	POS	NEG	POS	POS	NEG	NEG	NEG	NEG	POS	NEG	AMB	NEG	NEG	NEG	POS	POS	NEG	NEG	POS	NEG	NEG	NEG	NEG
Prediction for M10/0061, GenBank FR823292.1	*SCCmec XI (LGA251/M10-61)*	*CC130*																													
**Reference strain Dublin-DSH_M10 0061**	**SCCmec XI (LGA251/M10-61)**	**CC130**	**POS**	**POS**	**POS**	**NEG**	**NEG**	**POS**	**POS**	**NEG**	**POS**	**POS**	**NEG**	**NEG**	**NEG**	**NEG**	**POS**	**NEG**	**POS**	**AMB**	**NEG**	**NEG**	**NEG**	**POS**	**POS**	**NEG**	**AMB**	**NEG**	**NEG**	**NEG**	**NEG**
Prediction for LGA251, GenBank FR821779.1	*SCCmec XI (LGA251/M10-61)*	*ST425*																													
UKD-14015835	SCCmec XI (LGA251/M10-61)	CC130	POS	POS	POS	NEG	NEG	POS	POS	NEG	POS	POS	NEG	NEG	NEG	NEG	POS	NEG	POS	NEG	NEG	NEG	NEG	POS	POS	NEG	POS	NEG	NEG	NEG	NEG
UKD-16573252	SCCmec XI (LGA251/M10-61)	CC130	POS	POS	POS	NEG	NEG	POS	POS	NEG	POS	POS	NEG	NEG	NEG	NEG	POS	NEG	POS	NEG	NEG	NEG	NEG	POS	POS	NEG	POS	NEG	NEG	NEG	
UKD-0217002	PseudoSCCmec, class B mec complex+Hg	CC8	POS	POS	POS	NEG	POS	POS	POS	NEG	POS	POS	POS	NEG	NEG	NEG	POS	AMB	POS	NEG	NEG	NEG	NEG	POS	POS	NEG	NEG	POS	POS	NEG	POS
UKD-0008510	PseudoSCCmec, class B mec complex+Hg	CC8	POS	POS	POS	NEG	POS	POS	POS	NEG	POS	POS	POS	NEG	NEG	NEG	POS	POS	POS	NEG	NEG	NEG	NEG	POS	POS	NEG	NEG	POS	POS	NEG	POS
UKD-0110962	PseudoSCCmec, class B mec complex+Hg	CC8	POS	POS	POS	NEG	POS	POS	POS	NEG	POS	POS	POS	NEG	NEG	NEG	POS	POS	POS	POS	NEG	NEG	NEG	POS	POS	NEG	AMB	POS	POS	NEG	POS
KHDN-10408593	PseudoSCCmec, class B mec complex+Cu/Hg	CC8	POS	POS	POS	NEG	POS	POS	POS	NEG	POS	POS	POS	NEG	NEG	NEG	POS	POS	AMB	NEG	NEG	NEG	NEG	POS	POS	NEG	NEG	POS	POS	NEG	POS
UKD-0007201	PseudoSCCmec, class B mec complex	CC5	POS	POS	POS	NEG	NEG	POS	POS	NEG	POS	POS	NEG	NEG	POS	NEG	NEG	NEG	NEG	NEG	NEG	NEG	NEG	POS	POS	NEG	POS	POS	POS	NEG	POS
UKD-0033204	PseudoSCCmec, class B mec complex	CC5	POS	POS	POS	NEG	NEG	POS	POS	NEG	POS	POS	NEG	NEG	POS	NEG	NEG	NEG	NEG	NEG	NEG	NEG	NEG	POS	AMB	NEG	POS	POS	POS	NEG	POS
UKD-0134329	PseudoSCCmec, class B mec complex	CC5	NEG	NEG	AMB	NEG	NEG	POS	AMB	NEG	POS	POS	NEG	NEG	POS	NEG	NEG	NEG	NEG	NEG	NEG	NEG	NEG	NEG	AMB	NEG	AMB	POS	AMB	NEG	POS
UKD-0806946	PseudoSCCmec class C+As/Cu	CC398	POS	NEG	POS	POS	POS	POS	NEG	POS	POS	POS	NEG	NEG	NEG	NEG	POS	NEG	POS	AMB	NEG	NEG	NEG	POS	POS	POS	NEG	NEG	NEG	NEG	NEG
UKD-0500610	ACME2	CC8	POS	POS	POS	NEG	POS	POS	POS	NEG	POS	POS	POS	NEG	NEG	NEG	POS	POS	POS	AMB	NEG	NEG	NEG	POS	POS	NEG	AMB	POS	POS	NEG	POS
UKD-0610438	ACME2	CC8	POS	POS	POS	NEG	POS	POS	POS	NEG	POS	POS	POS	NEG	NEG	NEG	POS	POS	AMB	AMB	NEG	NEG	NEG	POS	POS	NEG	POS	POS	POS	NEG	POS
KHDN-10409344	ACME2	CC8	POS	POS	POS	NEG	POS	POS	POS	NEG	POS	POS	POS	NEG	NEG	NEG	POS	POS	AMB	NEG	NEG	NEG	NEG	POS	POS	NEG	AMB	POS	POS	NEG	POS
UKD-0322559	ACME2+speG+czrC+ccrAB4	CC8	POS	POS	POS	NEG	POS	POS	POS	NEG	POS	POS	POS	NEG	NEG	NEG	POS	POS	POS	NEG	NEG	NEG	NEG	POS	POS	NEG	NEG	POS	POS	NEG	POS
UKD-0501998	ACME2+speG+czrC+ccrAB4	CC8	POS	POS	POS	NEG	POS	POS	POS	NEG	POS	POS	POS	NEG	NEG	NEG	POS	POS	POS	AMB	NEG	NEG	NEG	POS	POS	NEG	NEG	POS	POS	NEG	POS
UKD-0505213	ACME2+speG+czrC+ccrAB4	CC8	POS	POS	POS	NEG	POS	POS	POS	NEG	POS	POS	POS	NEG	NEG	NEG	POS	POS	POS	POS	NEG	NEG	NEG	POS	POS	NEG	AMB	POS	POS	NEG	POS
Prediction for MSSA476, GenBank BX571857.1	*fus+tir+ccrAB1 (MSSA476)*	*CC1*	*POS*	*POS*	*POS*	*NEG*	*NEG*	*POS*	*POS*	*NEG*	*POS*	*POS*	*NEG*	*NEG*	*POS*	*NEG*	*POS*	*NEG*	*AMB*	*NEG*	*AMB*	*NEG*	*NEG*	*POS*	*AMB*	*NEG*	*POS*	*POS*	*NEG*	*POS*	*POS*
**Reference strain Sanger 476**	**fus+tir+ccrAB1 (MSSA476)**	**CC1**	**POS**	**POS**	**POS**	**NEG**	**NEG**	**POS**	**POS**	**NEG**	**POS**	**POS**	**NEG**	**NEG**	**POS**	**NEG**	**POS**	**NEG**	**AMB**	**NEG**	**POS**	**NEG**	**NEG**	**POS**	**NEG**	**NEG**	**POS**	**POS**	**NEG**	**POS**	**POS**
UKD-14023914	fus+tir+ccrAB1 (MSSA476)	CC1	POS	POS	POS	NEG	NEG	POS	POS	NEG	POS	POS	NEG	NEG	POS	NEG	POS	NEG	AMB	NEG	POS	NEG	NEG	POS	NEG	NEG	POS	POS	NEG	POS	POS
UKD-K00034	kdp+ccrAB2	CC182	POS	NEG	POS	POS	NEG	POS	POS	NEG	POS	POS	POS	NEG	NEG	NEG	NEG	NEG	NEG	NEG	NEG	NEG	NEG	POS	POS	NEG	NEG	POS	NEG	POS	POS
UKD-15580369	As+ccrAB4	CC22	POS	POS	POS	NEG	POS	POS	NEG	POS	POS	POS	NEG	NEG	POS	NEG	NEG	NEG	NEG	NEG	NEG	NEG	NEG	POS	NEG	NEG	POS	POS	NEG	POS	POS
UKD-15570100	ACME3+ccrAB1 (Strain 21342)	CC80	POS	POS	POS	NEG	NEG	POS	POS	NEG	POS	POS	NEG	NEG	NEG	NEG	POS	NEG	AMB	NEG	POS	NEG	NEG	POS	POS	AMB	POS	POS	NEG	POS	POS
UKD-1010678	speG+czrC+ccrAB4	CC8	POS	POS	POS	NEG	POS	POS	POS	NEG	POS	POS	POS	NEG	NEG	NEG	POS	AMB	POS	NEG	NEG	NEG	NEG	POS	POS	NEG	NEG	POS	POS	NEG	POS
UKD-1008243	speG+czrC+ccrAB4	CC8	POS	POS	POS	NEG	POS	POS	POS	NEG	POS	POS	POS	NEG	NEG	NEG	POS	AMB	POS	AMB	NEG	NEG	NEG	POS	POS	NEG	AMB	POS	POS	NEG	POS
UKD-1009430	speG+czrC+ccrAB4	CC8	POS	POS	POS	NEG	POS	POS	POS	NEG	POS	POS	POS	NEG	NEG	NEG	POS	POS	AMB	NEG	NEG	NEG	NEG	POS	POS	NEG	NEG	POS	POS	NEG	POS
UKD-12071828	speG+czrC+ccrAB4	CC22	POS	POS	POS	NEG	POS	POS	NEG	POS	POS	POS	NEG	NEG	POS	NEG	NEG	NEG	NEG	NEG	NEG	NEG	NEG	POS	NEG	NEG	POS	POS	NEG	POS	POS
UKD-14011883	speG+czrC+ccrAB4	CC22	POS	POS	POS	NEG	POS	POS	NEG	POS	POS	POS	NEG	NEG	POS	NEG	NEG	NEG	NEG	NEG	NEG	NEG	NEG	POS	NEG	NEG	POS	POS	NEG	POS	POS
UKD-15008293	speG+czrC+ccrAB4	CC22	POS	POS	POS	NEG	POS	POS	NEG	POS	POS	POS	NEG	NEG	POS	NEG	NEG	NEG	NEG	NEG	NEG	NEG	NEG	POS	NEG	NEG	POS	POS	NEG	POS	POS
UKD-1514877	speG+czrC+ccrAB4	CC22	POS	POS	POS	NEG	POS	POS	NEG	POS	POS	POS	NEG	NEG	POS	NEG	NEG	NEG	NEG	NEG	NEG	NEG	NEG	POS	NEG	NEG	POS	POS	NEG	POS	POS
UKD-15580971	speG+czrC+ccrAB4	CC22	POS	POS	POS	NEG	POS	POS	NEG	POS	POS	POS	NEG	NEG	POS	NEG	NEG	NEG	NEG	NEG	NEG	NEG	NEG	POS	NEG	NEG	POS	POS	NEG	POS	POS
UKD-15582646	speG+czrC+ccrAB4	CC22	POS	POS	POS	NEG	POS	POS	NEG	POS	POS	POS	NEG	NEG	POS	NEG	NEG	NEG	NEG	NEG	NEG	NEG	NEG	POS	NEG	NEG	POS	POS	NEG	POS	POS

Isolate ID	SCCmec type	Clonal complex	ADHAESION FACTORS / GENES ENCODING MICROBIAL SURFACE COMPONENTS RECOGNIZING ADHESIVE MATRIX MOLECULES (MSCRAMM GENES)																		IMMUNOD.AG.B		DEFENSIN RESIST.		TRANSFERRIN BINDING PROT		
			Ser-Asp rich fibrinogen-/bone sialoprotein-binding protein C							Ser-Asp rich fibrinogen-/bone sialoprotein-binding protein D					van Willebrand factor binding protein						immunodominant antigen B		defensin resistance protein		transferrin-binding protein		
			sdrC	sdrC (cons)	sdrC (B1)	sdrC (COL)	sdrC (Mu50)	sdrC (MW2+MRSA252+RF122)	sdrC (OtherThan252+RF122)	sdrD	sdrD (cons)	sdrD (COL+MW2)	sdrD (Mu50)	sdrD (other)	vwb	vwb (cons)	vwb (COL+MW2)	vwb (MRSA252)	vwb (Mu50)	vwb (RF122)	isaB	isaB (MRSA252)	mprF (COL+MW2)	mprF (Mu50+252)	isdA (cons)	isdA (MRSA252)	isdA (Other Than MRSA252)
VIRTUAL HYBRIDISATION for COL, GenBank CP000046	SCCmec I (COL)	CC8	POS	POS	NEG	POS	NEG	NEG	POS	POS	POS	POS	NEG	NEG	POS	POS	POS	NEG	NEG	NEG	POS	NEG	POS	NEG	POS	NEG	POS
Reference strain COL	SCCmec I (COL)	CC8	POS	POS	NEG	POS	NEG	NEG	POS	POS	POS	POS	NEG	NEG	POS	POS	POS	NEG	NEG	NEG	POS	POS	POS	NEG	POS	NEG	POS
UKD-0007063	SCCmec I (COL)	CC5	POS	POS	POS	NEG	POS	NEG	POS	POS	POS	NEG	POS	NEG	POS	POS	NEG	NEG	POS	NEG	POS	AMB	POS	AMB	POS	AMB	POS
UKD-0220759	SCCmec I (COL)	CC5	POS	POS	NEG	NEG	POS	NEG	POS	POS	POS	NEG	AMB	NEG	POS	POS	NEG	NEG	POS	NEG	POS	AMB	NEG	POS	POS	NEG	POS
UKD-0308986	SCCmec I (COL)	CC5	POS	POS	NEG	NEG	POS	NEG	POS	POS	POS	NEG	POS	NEG	POS	POS	NEG	NEG	POS	NEG	POS	AMB	AMB	POS	POS	AMB	POS
UKD-0500009	SCCmec I (COL)	CC5	POS	POS	NEG	NEG	POS	NEG	POS	POS	POS	NEG	POS	NEG	POS	POS	NEG	NEG	POS	NEG	POS	POS	POS	POS	POS	NEG	POS
UKD-0508483	SCCmec I (COL)	CC5	POS	POS	NEG	NEG	POS	NEG	POS	NEG	NEG	NEG	NEG	NEG	POS	POS	NEG	NEG	POS	NEG	POS	POS	POS	POS	POS	NEG	POS
UKD-0519001	SCCmec I (COL)	CC5	POS	POS	NEG	NEG	POS	NEG	POS	POS	POS	NEG	POS	NEG	POS	POS	NEG	NEG	POS	NEG	POS	AMB	AMB	POS	POS	NEG	POS
UKD-0701876	SCCmec I (COL)	CC5	POS	POS	NEG	NEG	POS	POS	POS	POS	POS	NEG	POS	NEG	POS	POS	NEG	NEG	POS	NEG	POS	AMB	POS	AMB	POS	AMB	POS
UKD-0708865	SCCmec I (COL)	CC5	POS	POS	NEG	NEG	POS	NEG	POS	POS	POS	NEG	NEG	NEG	POS	POS	NEG	NEG	POS	NEG	POS	NEG	NEG	NEG	AMB	NEG	AMB
UKD-1001782	SCCmec I (COL)	CC5	POS	POS	NEG	NEG	POS	NEG	POS	POS	POS	NEG	POS	NEG	POS	POS	NEG	NEG	POS	NEG	POS	AMB	POS	AMB	POS	NEG	POS
Prediction for PSP1996, GenBank ANHU	SCCmec I (PSP1996)	CC8	POS	POS	NEG	POS	NEG	NEG	POS	POS	POS	POS	NEG	NEG	POS	POS	POS	NEG	NEG	NEG	POS	NEG	POS	NEG	POS	NEG	POS
UKD-0261727	SCCmec I (PSP1996)	CC8	POS	POS	NEG	POS	NEG	NEG	POS	POS	POS	POS	NEG	NEG	POS	POS	POS	NEG	NEG	NEG	POS	AMB	POS	AMB	POS	NEG	POS
VIRTUAL HYBRIDISATION for MRSA7 : VIRTUAL HYBRII	SCCmec I+fus+tirS (Geraldine Clone)	CC5	POS	POS	NEG	NEG	POS	NEG	POS	POS	POS	NEG	POS	NEG	POS	POS	NEG	NEG	POS	NEG	POS	NEG	NEG	POS	POS	NEG	POS
UKD-1221930	SCCmec I+fus+tirS (Geraldine Clone)	CC5	POS	POS	AMB	NEG	POS	NEG	POS	POS	POS	NEG	POS	NEG	POS	POS	NEG	NEG	POS	NEG	POS	AMB	POS	AMB	POS	AMB	POS
Prediction for N315, GenBank BA000018.3	SCCmec II (N315)	CC5	POS	POS	NEG	NEG	POS	NEG	POS	POS	POS	NEG	POS	NEG	POS	POS	NEG	NEG	POS	NEG	POS	NEG	NEG	POS	POS	NEG	POS
Reference strain N315	SCCmec II (N315)	CC5	POS	POS	NEG	NEG	POS	NEG	POS	POS	POS	NEG	POS	NEG	POS	POS	NEG	NEG	POS	NEG	POS	POS	NEG	POS	POS	NEG	POS
Prediction for MRSA252, GenBank BX571856.1	SCCmec II (N315)	CC30	POS	POS	NEG	NEG	NEG	POS	NEG	NEG	NEG	NEG	NEG	NEG	POS	POS	NEG	POS	NEG	NEG	NEG	POS	NEG	POS	POS	POS	NEG
Reference strain Sanger 252	SCCmec II (N315)	CC30	POS	POS	NEG	NEG	NEG	POS	NEG	NEG	NEG	NEG	NEG	NEG	POS	POS	NEG	POS	NEG	NEG	NEG	POS	NEG	POS	POS	POS	NEG
UKD-0230792	SCCmec II (N315)	CC30	POS	POS	NEG	NEG	NEG	NEG	NEG	POS	POS	NEG	NEG	POS	POS	POS	NEG	POS	NEG	NEG	NEG	POS	AMB	POS	POS	POS	NEG
Prediction for Mu50, GenBank BA000017.4	SCCmec II (N315)	CC5	POS	POS	NEG	NEG	POS	NEG	POS	POS	POS	NEG	POS	NEG	POS	POS	NEG	NEG	POS	NEG	POS	NEG	NEG	POS	POS	NEG	POS
Reference strain MU50	SCCmec II (N315)	CC5	POS	POS	NEG	NEG	POS	NEG	POS	POS	POS	NEG	POS	NEG	POS	POS	NEG	NEG	POS	NEG	POS	AMB	NEG	POS	POS	NEG	POS
Prediction for JH1, GenBank CP000736.1	SCCmec II (JH1/JH9)	CC5	POS	POS	NEG	NEG	POS	NEG	POS	POS	POS	NEG	POS	NEG	POS	POS	NEG	NEG	POS	NEG	POS	NEG	NEG	POS	POS	NEG	POS
Prediction for JH9, GenBank CP000703.1	SCCmec II (JH1/JH9)	CC5	POS	POS	NEG	NEG	POS	NEG	POS	POS	POS	NEG	POS	NEG	POS	POS	NEG	NEG	POS	NEG	POS	NEG	NEG	POS	POS	NEG	POS
Reference strain Dublin-DSH_AR07.4_0237	SCCmec II (JH1/JH9)		POS	POS	NEG	NEG	POS	NEG	POS	POS	POS	NEG	POS	NEG	POS	POS	NEG	NEG	POS	NEG	AMB	POS	AMB	POS	POS	AMB	POS
Reference strain NARSA_382 / USA100	SCCmec II (JH1/JH9)	CC5	POS	POS	NEG	NEG	POS	NEG	POS	POS	POS	NEG	POS	NEG	POS	POS	NEG	NEG	POS	NEG	POS	AMB	AMB	POS	POS	NEG	POS
UKD-0046921	SCCmec II (JH1/JH9)	CC5	POS	POS	NEG	NEG	POS	NEG	POS	POS	POS	NEG	POS	NEG	POS	POS	NEG	NEG	POS	NEG	POS	AMB	AMB	POS	POS	NEG	POS
UKD-0333415	SCCmec II (JH1/JH9)	CC5	POS	POS	NEG	NEG	POS	NEG	POS	POS	POS	NEG	POS	NEG	POS	POS	NEG	NEG	POS	NEG	POS	AMB	AMB	POS	POS	NEG	POS
UKD-0341645	SCCmec II (JH1/JH9)	CC5	POS	POS	NEG	NEG	POS	NEG	POS	POS	POS	NEG	POS	NEG	POS	POS	NEG	NEG	POS	NEG	POS	AMB	AMB	POS	POS	NEG	POS
UKD-0504626	SCCmec II (JH1/JH9)	CC5	POS	POS	NEG	NEG	POS	NEG	POS	POS	POS	NEG	POS	NEG	POS	POS	NEG	NEG	POS	NEG	POS	AMB	NEG	POS	POS	NEG	POS
UKD-0507581	SCCmec II (JH1/JH9)	CC5	POS	POS	NEG	NEG	POS	NEG	POS	POS	POS	NEG	POS	NEG	POS	POS	NEG	NEG	POS	NEG	AMB	POS	AMB	POS	POS	NEG	POS
UKD-0507903	SCCmec II (JH1/JH9)	CC5	POS	POS	NEG	NEG	POS	NEG	POS	POS	POS	NEG	POS	NEG	POS	POS	NEG	NEG	POS	NEG	AMB	POS	AMB	POS	POS	NEG	POS
UKD-0700817	SCCmec II (JH1/JH9)	CC5	POS	POS	NEG	NEG	POS	NEG	POS	POS	POS	NEG	POS	NEG	POS	POS	NEG	NEG	POS	NEG	POS	AMB	NEG	POS	POS	NEG	POS
UKD-1014561	SCCmec II (JH1/JH9)	CC5	POS	POS	NEG	NEG	POS	NEG	POS	POS	POS	NEG	POS	NEG	POS	POS	NEG	NEG	POS	NEG	POS	AMB	AMB	POS	POS	NEG	POS
UKD-1037507	SCCmec II (JH1/JH9)	CC5	POS	POS	AMB	NEG	POS	NEG	POS	POS	POS	NEG	POS	NEG	POS	POS	NEG	NEG	POS	NEG	POS	AMB	POS	AMB	POS	AMB	POS
UKD-1038321	SCCmec II (JH1/JH9)	CC5	POS	POS	NEG	NEG	POS	NEG	POS	POS	POS	NEG	POS	NEG	POS	POS	NEG	NEG	POS	NEG	POS	AMB	AMB	POS	POS	NEG	POS
UKD-1111608	SCCmec II (JH1/JH9)	CC5	POS	POS	NEG	NEG	POS	NEG	POS	POS	POS	NEG	POS	NEG	POS	POS	NEG	NEG	POS	NEG	POS	AMB	AMB	POS	POS	AMB	POS
UKD-12753135	SCCmec II (JH1/JH9)	CC5	POS	POS	NEG	NEG	POS	AMB	POS	POS	POS	NEG	POS	NEG	POS	POS	NEG	NEG	POS	NEG	POS	AMB	POS	AMB	POS	NEG	POS
UKD-1312816	SCCmec II (JH1/JH9)	CC5	POS	POS	NEG	NEG	POS	NEG	POS	POS	POS	NEG	POS	NEG	POS	POS	NEG	NEG	POS	NEG	POS	AMB	AMB	POS	POS	NEG	POS
UKD-1401623	SCCmec II (JH1/JH9)	CC5	POS	POS	AMB	NEG	POS	POS	POS	POS	POS	NEG	POS	NEG	POS	POS	NEG	NEG	POS	NEG	AMB	POS	POS	AMB	POS	AMB	POS
UKD-15571943	SCCmec II+czrC+ccrA/B-4	CC5	POS	POS	POS	NEG	POS	POS	POS	POS	POS	NEG	POS	NEG	POS	POS	NEG	NEG	POS	NEG	POS	AMB	POS	AMB	POS	AMB	POS
UKD-15576888	SCCmec II+czrC+ccrA/B-4	CC5	POS	POS	AMB	NEG	POS	POS	POS	POS	POS	NEG	POS	NEG	POS	POS	NEG	NEG	POS	NEG	POS	AMB	POS	AMB	POS	AMB	POS
UKD-14576461	SCCmec II+speG+czrC+ccrA/B-4	CC5	POS	POS	NEG	NEG	POS	POS	POS	POS	POS	NEG	POS	NEG	POS	POS	NEG	NEG	POS	NEG	AMB	POS	POS	AMB	POS	AMB	POS
UKD-15581649	SCCmec II+speG+czrC+ccrA/B-4	CC5	POS	POS	NEG	NEG	POS	AMB	POS	POS	POS	NEG	POS	NEG	POS	POS	NEG	NEG	POS	NEG	POS	AMB	POS	AMB	POS	AMB	POS
Prediction for CIGC345D, GenBank AHVO	SCCmec II A/B/D	CC8	POS	POS	NEG	POS	NEG	NEG	POS	POS	POS	POS	NEG	NEG	POS	POS	POS	NEG	NEG	NEG	POS	NEG	POS	NEG	POS	NEG	POS
UKD-12K00171	SCCmec II A/B/D	CC8	POS	POS	AMB	POS	NEG	NEG	POS	POS	POS	POS	NEG	NEG	POS	POS	POS	NEG	NEG	NEG	AMB	POS	AMB	POS	POS	AMB	POS
Prediction for JKD6008, GenBank CP002120.1	SCC [mec III+Cd] (JKD6008)	CC8 (ST239)	POS	POS	NEG	POS	NEG	NEG	POS	POS	POS	POS	NEG	NEG	POS	POS	POS	NEG	NEG	NEG	NEG	POS	POS	NEG	POS	NEG	POS
Prediction for JKD6009, GenBank ABSA	SCC [mec III+Cd] (JKD6008)	CC8 (ST239)	POS	POS	NEG	POS	NEG	NEG	POS	POS	POS	POS	NEG	NEG	POS	POS	POS	NEG	NEG	NEG	NEG	POS	POS	NEG	POS	NEG	POS
Reference strain ATCC_33592	SCC [mec III+Cd] (ATCC33592)	CC8 (ST239)	POS	POS	NEG	POS	NEG	NEG	POS	POS	POS	POS	NEG	NEG	POS	POS	POS	NEG	NEG	NEG	NEG	POS	POS	AMB	POS	NEG	POS
Prediction for PPUKM-775-2009, GenBank AMRE	SCC [mec III+Cd+ccrC] (PPUKM-775-2009)	CC8 (ST239)	POS	POS	NEG	POS	NEG	NEG	POS	POS	POS	POS	NEG	NEG	POS	POS	POS	NEG	NEG	NEG	NEG	POS	POS	NEG	POS	NEG	POS
Prediction for T0131, GenBank CP002643.1	SCC [mec III+Cd+ccrC] (T0131)	CC8 (ST239)	POS	POS	NEG	POS	NEG	NEG	POS	POS	POS	POS	NEG	NEG	POS	POS	POS	NEG	NEG	NEG	NEG	POS	POS	NEG	POS	NEG	POS
Prediction for XN108, GenBank CP007447.1	SCC [mec III+Cd+ccrC] (XN108)	CC8 (ST239)	POS	POS	NEG	POS	NEG	NEG	POS	POS	POS	POS	NEG	NEG	POS	POS	POS	NEG	NEG	NEG	NEG	POS	POS	NEG	POS	NEG	POS
Prediction for CN79, GenBank ANCJ	SCC [mec III+Cd+ccrC] (CN79)	CC8 (ST239)	POS	POS	NEG	POS	NEG	NEG	POS	POS	POS	POS	NEG	NEG	POS	POS	POS	NEG	NEG	NEG	NEG	POS	POS	NEG	POS	NEG	POS
Prediction for 16K, GenBank BABZ	SCC [mec III+Cd+ccrC] (CN79)	CC8 (ST239)	POS	POS	NEG	POS	NEG	NEG	POS	POS	POS	POS	NEG	NEG	POS	POS	POS	NEG	NEG	NEG	NEG	POS	POS	NEG	POS	NEG	POS
UKD-0835987	SCC [mec III+Cd+ccrC] (CN79)	CC8 (ST239)	POS	POS	NEG	POS	NEG	NEG	POS	POS	POS	POS	NEG	NEG	POS	POS	POS	NEG	NEG	NEG	NEG	POS	POS	AMB	POS	NEG	POS
UKD-16581769	SCCmec III+Cd+ccrC	CC8 (ST239)	POS	POS	NEG	POS	NEG	NEG	POS	POS	POS	POS	NEG	NEG	POS	POS	POS	NEG	NEG	NEG	NEG	POS	POS	NEG	POS	NEG	POS
Prediction for Bmb9393, GenBank CP005288.1	SCCmec III+Cd/Hg+ccrC (Bmb9393)	CC8 (ST239)	POS	POS	NEG	POS	NEG	NEG	POS	POS	POS	POS	NEG	NEG	POS	POS	POS	NEG	NEG	NEG	NEG	POS	POS	NEG	POS	NEG	POS
UKD-15574263	SCCmec III+Cd/Hg+ccrC (Bmb9393)	CC8 (ST239)	POS	POS	NEG	POS	NEG	NEG	POS	POS	POS	POS	NEG	NEG	POS	POS	POS	NEG	NEG	NEG	NEG	POS	POS	AMB	POS	AMB	POS
Prediction for SK1585, GenBank AYLT	SCCmec III+ccrC+Cd/Hg (SK1585)	CC8 (ST239)	POS	POS	NEG	POS	NEG	NEG	POS	POS	POS	POS	NEG	NEG	POS	POS	NEG	NEG	NEG	NEG	NEG	POS	NEG	POS	POS	POS	NEG
UKD-105861	SCCmec III+ccrC+Cd/Hg (SK1585)	CC8 (ST239)	POS	POS	NEG	POS	NEG	NEG	POS	POS	POS	POS	NEG	NEG	POS	POS	POS	NEG	NEG	NEG	NEG	POS	POS	AMB	POS	NEG	POS
UKD-134572	SCCmec III+ccrC+Cd/Hg (SK1585)	CC8 (ST239)	POS	POS	NEG	POS	NEG	NEG	POS	POS	POS	AMB	NEG	NEG	POS	POS	POS	NEG	NEG	NEG	NEG	POS	POS	NEG	POS	NEG	POS
UKD-139143	SCCmec III+ccrC+Cd/Hg (SK1585)	CC8 (ST239)	POS	POS	NEG	POS	NEG	NEG	POS	POS	POS	POS	NEG	NEG	POS	POS	POS	NEG	NEG	NEG	NEG	POS	POS	AMB	POS	NEG	POS
Prediction for TW20, GenBank FN433596.1	SCC [mec III+Cd/Hg+ccrC] (TW20)	CC8 (ST239)	POS	POS	NEG	POS	NEG	NEG	POS	POS	POS	POS	NEG	NEG	POS	POS	POS	NEG	NEG	NEG	NEG	POS	POS	NEG	POS	NEG	POS
Prediction for ATCC BAA-39, GenBank AEEK	SCC [mec III+Cd/Hg+ccrC] (TW20)	CC8 (ST239)	POS	POS	NEG	POS	NEG	NEG	POS	POS	POS	POS	NEG	NEG	POS	POS	POS	NEG	NEG	NEG	NEG	POS	POS	NEG	POS	NEG	POS

Isolate ID	SCCmec type	Clonal complex	ADHAESION FACTORS / GENES ENCODING MICROBIAL SURFACE COMPONENTS RECOGNIZING ADHESIVE MATRIX MOLECULES (MSCRAMM GENES)																		IMMUNOD.AG.B		DEFENSIN RESIST.		TRANSFERRIN BINDING PROT		
			Ser-Asp rich fibrinogen-/bone sialoprotein-binding protein C							Ser-Asp rich fibrinogen-/bone sialoprotein-binding protein D					van Willebrand factor binding protein						immunodominant antigen B		defensin resistance protein		transferrin-binding protein		
			sdrC	sdrC (cons)	sdrC (B1)	sdrC (COL)	sdrC (Mu50)	sdrC (MW2+MRSA252+RF122)	sdrC (OtherThan252+RF122)	sdrD	sdrD (cons)	sdrD (COL+MW2)	sdrD (Mu50)	sdrD (other)	vwb	vwb (cons)	vwb (COL+MW2)	vwb (MRSA252)	vwb (Mu50)	vwb (RF122)	isaB	isaB (MRSA252)	mprF (COL+MW2)	mprF (Mu50+252)	isdA (cons)	isdA (MRSA252)	isdA (Other Than MRSA252)
Prediction for MW2, GenBank BA000033.2	*SCCmec IVa (MW2)*	*CC1*	***POS***	***POS***	***NEG***	***NEG***	***NEG***	***POS***	***POS***	***POS***	***POS***	***POS***	***NEG***	***NEG***	***POS***	***POS***	***POS***	***NEG***	***NEG***	***NEG***	***POS***	***NEG***	***POS***	***NEG***	***POS***	***NEG***	***POS***
Reference strain MW2	SCCmec IVa (MW2)	CC1	**POS**	**POS**	**NEG**	**NEG**	**NEG**	**POS**	**POS**	**POS**	**POS**	**POS**	**NEG**	**NEG**	**POS**	**POS**	**POS**	**NEG**	**NEG**	**NEG**	**POS**	**NEG**	**POS**	**NEG**	**POS**	**NEG**	**POS**
Reference strain CA05 (JCSC1968)	SCCmec IVa (MW2)	CC45	**POS**	**POS**	**POS**	**NEG**	**NEG**	**NEG**	**NEG**	**POS**	**POS**	**POS**	**NEG**	**NEG**	**POS**	**POS**	**NEG**	**POS**	**NEG**	**NEG**	**NEG**	**POS**	**AMB**	**POS**	**POS**	**POS**	**AMB**
**UKD-0127106**	SCCmec IVa (MW2)	CC1	POS	POS	NEG	NEG	NEG	POS	POS	POS	POS	POS	NEG	NEG	POS	POS	POS	NEG	NEG	NEG	POS	AMB	POS	AMB	POS	NEG	POS
**UKD-0701446**	SCCmec IVa (MW2)	CC1	POS	POS	NEG	NEG	NEG	POS	POS	POS	POS	POS	NEG	NEG	POS	POS	POS	NEG	NEG	NEG	POS	AMB	POS	AMB	POS	NEG	POS
**UKD-0947486**	SCCmec IVa (MW2)	CC1	POS	POS	NEG	NEG	NEG	POS	POS	POS	POS	POS	NEG	NEG	POS	POS	POS	NEG	NEG	NEG	POS	AMB	POS	AMB	POS	NEG	POS
**UKD-1031910**	SCCmec IVa (MW2)	CC1	POS	POS	NEG	NEG	NEG	POS	POS	POS	POS	POS	NEG	NEG	POS	POS	POS	NEG	NEG	NEG	POS	AMB	POS	AMB	POS	NEG	POS
**UKD-14571328**	SCCmec IVa (MW2)	CC1	POS	POS	NEG	NEG	NEG	POS	POS	POS	POS	POS	NEG	NEG	POS	POS	POS	NEG	NEG	NEG	AMB	POS	POS	AMB	POS	NEG	POS
**UKD-03003012**	SCCmec IVa (MW2)	CC5	POS	POS	NEG	NEG	POS	AMB	POS	POS	POS	NEG	POS	NEG	POS	POS	NEG	NEG	POS	NEG	POS	POS	POS	POS	POS	NEG	POS
**UKD-15582582**	SCCmec IVa (MW2)	CC6	POS	POS	NEG	POS	NEG	NEG	POS	POS	POS	NEG	POS	NEG	POS	POS	NEG	POS	NEG	NEG	POS	AMB	POS	AMB	POS	AMB	POS
**UKD-16574230**	SCCmec IVa (MW2)	CC6	POS	POS	NEG	POS	NEG	NEG	POS	POS	POS	NEG	POS	NEG	POS	POS	NEG	POS	NEG	NEG	AMB	POS	POS	AMB	POS	AMB	POS
**UKD-16582560**	SCCmec IVa (MW2)	CC6	POS	POS	NEG	POS	NEG	NEG	POS	POS	POS	NEG	POS	NEG	POS	POS	NEG	POS	NEG	NEG	POS	AMB	POS	NEG	POS	NEG	POS
**UKD-0802855**	SCCmec IVa (MW2)	CC7	POS	POS	NEG	POS	NEG	NEG	POS	POS	POS	NEG	POS	NEG	POS	POS	NEG	POS	NEG	NEG	POS	AMB	POS	NEG	POS	NEG	POS
**KHDN-10405258**	SCCmec IVa (MW2)	CC8	POS	POS	NEG	POS	NEG	NEG	POS	POS	POS	POS	NEG	NEG	POS	POS	POS	NEG	NEG	NEG	AMB	POS	POS	AMB	POS	NEG	POS
**UKD-15573497**	SCCmec IVa (MW2)	CC22	POS	POS	NEG	NEG	POS	POS	POS	POS	POS	NEG	NEG	POS	POS	POS	NEG	NEG	NEG	POS	AMB	POS	POS	AMB	POS	AMB	POS
**UKD-0634729**	SCCmec IVa (MW2)	CC30	POS	POS	NEG	NEG	AMB	POS	NEG	POS	POS	NEG	NEG	POS	POS	POS	NEG	POS	NEG	NEG	NEG	POS	AMB	POS	POS	POS	NEG
**UKD-1119771**	SCCmec IVa (MW2)	CC30	POS	POS	NEG	NEG	NEG	AMB	NEG	POS	POS	NEG	NEG	POS	POS	POS	NEG	POS	NEG	NEG	AMB	POS	POS	AMB	POS	POS	NEG
**KHDN-10411458**	SCCmec IVa (MW2)	CC30	POS	POS	NEG	NEG	NEG	NEG	NEG	POS	POS	NEG	NEG	POS	POS	POS	NEG	POS	NEG	NEG	NEG	POS	AMB	POS	POS	POS	NEG
**KHDN-398609**	SCCmec IVa (MW2)	CC30	POS	POS	NEG	NEG	AMB	POS	NEG	POS	POS	NEG	NEG	POS	POS	POS	NEG	POS	NEG	NEG	NEG	POS	POS	AMB	POS	POS	NEG
**KHDN-01081998**	SCCmec IVa (MW2)	CC30	POS	POS	NEG	NEG	NEG	AMB	NEG	POS	POS	NEG	NEG	POS	POS	POS	NEG	POS	NEG	NEG	AMB	POS	POS	AMB	POS	POS	AMB
**KHDN-10399223**	SCCmec IVa (MW2)	CC30	POS	POS	NEG	NEG	NEG	AMB	NEG	POS	POS	NEG	NEG	POS	POS	POS	NEG	POS	NEG	NEG	NEG	POS	AMB	POS	POS	POS	NEG
**KHDN-W261158**	SCCmec IVa (MW2)	CC30	POS	POS	NEG	NEG	NEG	AMB	NEG	POS	POS	NEG	NEG	POS	POS	POS	NEG	POS	NEG	NEG	NEG	POS	POS	AMB	POS	POS	NEG
**UKD-0002046**	SCCmec IVa (MW2)	CC45 [agr I]	POS	POS	POS	NEG	NEG	AMB	NEG	POS	POS	POS	NEG	NEG	POS	POS	NEG	POS	NEG	NEG	NEG	POS	NEG	POS	POS	POS	POS
**UKD-0029618**	SCCmec IVa (MW2)	CC45 [agr I]	POS	POS	POS	NEG	NEG	NEG	NEG	POS	POS	POS	NEG	NEG	POS	NEG	NEG	POS	NEG	NEG	NEG	POS	NEG	POS	POS	POS	AMB
**UKD-0043085**	SCCmec IVa (MW2)	CC45 [agr I]	POS	POS	POS	NEG	NEG	NEG	NEG	POS	POS	POS	NEG	NEG	POS	NEG	NEG	POS	NEG	NEG	NEG	POS	NEG	POS	POS	POS	AMB
**UKD-0047053**	SCCmec IVa (MW2)	CC45 [agr I]	POS	POS	POS	NEG	NEG	NEG	NEG	POS	POS	NEG	NEG	NEG	POS	NEG	NEG	POS	NEG	NEG	NEG	POS	NEG	NEG	POS	POS	NEG
**UKD-0047429**	SCCmec IVa (MW2)	CC45 [agr I]	POS	POS	POS	NEG	NEG	NEG	NEG	POS	POS	POS	NEG	NEG	POS	NEG	NEG	POS	NEG	NEG	NEG	POS	NEG	POS	POS	POS	AMB
**UKD-0112465**	SCCmec IVa (MW2)	CC45 [agr I]	POS	POS	POS	NEG	NEG	NEG	NEG	POS	POS	POS	NEG	NEG	POS	NEG	NEG	POS	NEG	NEG	NEG	POS	NEG	NEG	POS	POS	AMB
**UKD-0103483**	SCCmec IVa (MW2)	CC45 [agr I]	POS	POS	POS	NEG	NEG	NEG	NEG	POS	POS	POS	NEG	NEG	POS	NEG	NEG	POS	NEG	NEG	NEG	POS	NEG	NEG	POS	POS	NEG
**UKD-0305014**	SCCmec IVa (MW2)	CC45 [agr I]	POS	POS	POS	NEG	NEG	NEG	NEG	POS	POS	POS	NEG	NEG	POS	NEG	NEG	POS	NEG	NEG	NEG	POS	AMB	POS	POS	POS	AMB
**UKD-0401119**	SCCmec IVa (MW2)	CC45 [agr I]	POS	POS	POS	NEG	NEG	NEG	NEG	POS	POS	POS	NEG	NEG	POS	NEG	NEG	POS	NEG	NEG	NEG	POS	NEG	POS	POS	POS	AMB
**UKD-0537969**	SCCmec IVa (MW2)	CC45 [agr I]	POS	POS	POS	NEG	NEG	NEG	NEG	POS	POS	POS	NEG	NEG	POS	AMB	NEG	POS	NEG	NEG	NEG	POS	AMB	POS	POS	POS	NEG
**UKD-0703373**	SCCmec IVa (MW2)	CC45 [agr I]	POS	POS	POS	NEG	NEG	NEG	NEG	POS	POS	NEG	NEG	NEG	POS	NEG	NEG	POS	NEG	NEG	NEG	POS	NEG	NEG	POS	POS	NEG
**UKD-0813653**	SCCmec IVa (MW2)	CC45 [agr I]	POS	POS	POS	NEG	NEG	NEG	NEG	POS	POS	POS	NEG	NEG	POS	NEG	NEG	POS	NEG	NEG	NEG	POS	NEG	POS	POS	POS	AMB
**UKD-0839175**	SCCmec IVa (MW2)	CC45 [agr I]	POS	POS	POS	AMB	NEG	POS	NEG	POS	POS	POS	NEG	NEG	POS	POS	NEG	POS	POS	NEG	AMB	POS	AMB	POS	POS	POS	AMB
**UKD-1108422**	SCCmec IVa (MW2)	CC45 [agr I]	POS	POS	POS	NEG	NEG	NEG	NEG	POS	POS	POS	NEG	NEG	POS	POS	NEG	POS	NEG	NEG	AMB	POS	POS	AMB	POS	AMB	POS
**UKD-1380645**	SCCmec IVa (MW2)	CC45 [agr I]	POS	POS	POS	NEG	NEG	AMB	NEG	POS	POS	POS	NEG	NEG	POS	NEG	NEG	POS	NEG	NEG	NEG	POS	NEG	POS	POS	POS	NEG
**UKD-148016**	SCCmec IVa (MW2)	CC45 [agr I]	POS	POS	POS	NEG	NEG	POS	NEG	POS	POS	POS	NEG	NEG	POS	POS	NEG	POS	NEG	NEG	AMB	POS	AMB	POS	POS	POS	AMB
**UKD-16571773**	SCCmec IVa (MW2)	CC45 [agr I]	POS	POS	POS	NEG	NEG	POS	NEG	NEG	AMB	NEG	NEG	NEG	POS	POS	NEG	POS	POS	NEG	AMB	POS	AMB	POS	POS	POS	AMB
**KHDN-10407738**	SCCmec IVa (MW2)	CC45 [ST617]	POS	POS	POS	NEG	NEG	AMB	NEG	POS	POS	NEG	NEG	POS	POS	POS	NEG	NEG	NEG	NEG	POS	AMB	AMB	POS	POS	POS	AMB
**UKD-0853670**	SCCmec IVa (MW2)	CC88	POS	POS	NEG	NEG	POS	POS	POS	POS	POS	NEG	NEG	POS	POS	POS	NEG	NEG	POS	NEG	POS	AMB	POS	AMB	POS	NEG	POS
**UKD-1417352**	SCCmec IVa (MW2)	CC88	POS	POS	NEG	NEG	POS	POS	POS	POS	POS	NEG	NEG	POS	POS	POS	NEG	NEG	POS	NEG	POS	AMB	POS	AMB	POS	NEG	POS
**UKD-15571283**	SCCmec IVa (MW2)	CC88	POS	POS	NEG	NEG	POS	POS	POS	POS	POS	NEG	NEG	POS	POS	POS	NEG	NEG	POS	NEG	POS	AMB	POS	AMB	POS	AMB	POS
**KHDN-10406719**	SCCmec IVa (MW2)	ST93	POS	POS	NEG	POS	NEG	NEG	NEG	POS	POS	NEG	NEG	POS	POS	POS	NEG	NEG	NEG	NEG	POS	AMB	AMB	POS	POS	AMB	POS
**KHDN-0060294**	SCCmec IVa (MW2)	ST93	POS	POS	NEG	POS	NEG	NEG	NEG	POS	POS	NEG	NEG	POS	POS	POS	NEG	NEG	NEG	NEG	POS	AMB	NEG	NEG	POS	NEG	POS
**UKD-0734190**	SCCmec IVa (MW2)	CC97	POS	POS	NEG	NEG	POS	AMB	POS	NEG	NEG	NEG	NEG	NEG	POS	POS	NEG	NEG	NEG	POS	POS	AMB	POS	NEG	POS	NEG	POS
**UKD-13006983**	SCCmec IVa (MW2)	CC97	POS	POS	POS	NEG	POS	POS	POS	POS	POS	NEG	POS	NEG	POS	POS	NEG	NEG	NEG	POS	AMB	POS	AMB	POS	POS	AMB	POS
Prediction for H131520133	*SCCmec IVa (H131520133)*	*CC152*	***NEG***	***NEG***	***NEG***	***NEG***	***NEG***	***NEG***	***NEG***	***POS***	***POS***	***POS***	***NEG***	***NEG***	***POS***	***POS***	***NEG***	***NEG***	***NEG***	***NEG***	***POS***	***NEG***	***POS***	***NEG***	***AMB***	***NEG***	***NEG***
**KHDN-325892**	SCCmec IVa (H131520133)	CC30	POS	POS	NEG	NEG	NEG	POS	NEG	POS	POS	NEG	NEG	POS	POS	POS	NEG	NEG	NEG	NEG	NEG	POS	POS	AMB	POS	POS	AMB
**UKD-15580207**	SCCmec IVa (H131520133)	CC88	POS	POS	NEG	NEG	POS	POS	POS	POS	POS	NEG	NEG	POS	POS	POS	NEG	NEG	POS	NEG	POS	AMB	POS	AMB	POS	AMB	POS
**UKD-0521159**	SCCmec Iva	CC8	POS	POS	NEG	POS	NEG	NEG	POS	POS	POS	POS	NEG	NEG	POS	POS	POS	NEG	NEG	NEG	POS	POS	POS	AMB	POS	NEG	POS
Prediction for CMFT503 : HF569113.1	*SCCmec IVa (CMFT503)*																										
Prediction for 21343, GenBank AHKV	*SCCmec IVa (CMFT503)*	*CC88*	***POS***	***POS***	***NEG***	***NEG***	***POS***	***NEG***	***POS***	***POS***	***POS***	***NEG***	***NEG***	***POS***	***POS***	***POS***	***NEG***	***NEG***	***POS***	***NEG***	***POS***	***NEG***	***POS***	***NEG***	***POS***	***NEG***	***POS***
**UKD-1420740**	SCCmec IVa (CMFT503)	CC22	POS	POS	NEG	NEG	POS	POS	POS	POS	POS	NEG	NEG	POS	POS	POS	NEG	NEG	NEG	POS	AMB	POS	POS	AMB	POS	AMB	POS
**UKD-15575124**	SCCmec IVa (CMFT503)	CC22	POS	POS	NEG	NEG	POS	NEG	POS	POS	POS	NEG	NEG	POS	POS	POS	NEG	NEG	NEG	POS	NEG	POS	POS	AMB	POS	AMB	POS
**UKD-16584947**	SCCmec IVa (CMFT503)	CC22	POS	POS	NEG	NEG	POS	NEG	POS	POS	POS	NEG	NEG	POS	POS	POS	NEG	NEG	NEG	POS	NEG	POS	AMB	POS	POS	NEG	POS
**UKD-16575077**	SCCmec IVa (CMFT503)	CC88	POS	POS	NEG	NEG	POS	NEG	POS	POS	POS	NEG	NEG	POS	POS	POS	NEG	NEG	POS	NEG	AMB	POS	POS	AMB	POS	NEG	POS
Prediction for TCH1516, GenBank CP000730.1	*SCCmec IVa+ACME1+Cu (USA300)*	*CC8*	***POS***	***POS***	***NEG***	***POS***	***NEG***	***NEG***	***POS***	***POS***	***POS***	***POS***	***NEG***	***NEG***	***POS***	***POS***	***POS***	***NEG***	***NEG***	***NEG***	***POS***	***NEG***	***POS***	***NEG***	***POS***	***NEG***	***POS***
Reference strain USA300-TCH1516	SCCmec IVa+ACME1+Cu (USA300)	CC8	POS	POS	NEG	POS	NEG	NEG	POS	POS	POS	POS	NEG	NEG	POS	POS	POS	NEG	NEG	NEG	POS	AMB	POS	AMB	POS	NEG	POS
Prediction for FPR3757, GenBank CP000255.1	*SCCmec IVa+ACME1+Cu (USA300)*	*CC8*	***POS***	***POS***	***NEG***	***POS***	***NEG***	***NEG***	***POS***	***POS***	***POS***	***POS***	***NEG***	***NEG***	***POS***	***POS***	***POS***	***NEG***	***NEG***	***NEG***	***POS***	***NEG***	***POS***	***NEG***	***POS***	***NEG***	***POS***
**UKD-0516386**	SCCmec IVa+ACME1+Cu (USA300)	CC8	POS	POS	NEG	POS	NEG	NEG	POS	POS	POS	AMB	NEG	NEG	POS	POS	POS	NEG	NEG	NEG	POS	POS	POS	NEG	POS	NEG	POS
**UKD-0815773**	SCCmec IVa+ACME1+Cu (USA300)	CC8	POS	POS	NEG	POS	NEG	NEG	POS	POS	POS	POS	NEG	NEG	POS	POS	POS	NEG	NEG	NEG	POS	AMB	POS	NEG	POS	NEG	POS
**UKD-1414654**	SCCmec IVa+ACME1+Cu (USA300)	CC8	POS	POS	NEG	POS	NEG	AMB	POS	POS	POS	POS	NEG	NEG	POS	POS	POS	NEG	NEG	NEG	AMB	POS	POS	AMB	POS	AMB	POS
**UKD-0408036**	SCCmec IVa+ACME2	CC45 [agr I]	POS	POS	POS	NEG	NEG		NEG	POS	POS	POS	NEG	NEG	POS	POS	NEG	POS	NEG	NEG	NEG	POS	AMB	POS	POS	POS	AMB
**UKD-0044035**	SCCmec IVa+ccrC (Hannover)	CC8	AMB	POS	NEG	POS	NEG	NEG	POS	POS	POS	POS	NEG	NEG	POS	POS	POS	NEG	NEG	NEG	POS	POS	POS	NEG	POS	NEG	POS
**UKD-0136287**	SCCmec IVa+ccrC (Hannover)	CC8	POS	POS	NEG	POS	NEG	NEG	POS	POS	POS	POS	NEG	NEG	POS	POS	POS	NEG	NEG	NEG	POS	AMB	POS	AMB	POS	NEG	POS

Isolate ID	SCCmec type	Clonal complex	ADHAESION FACTORS / GENES ENCODING MICROBIAL SURFACE COMPONENTS RECOGNIZING ADHESIVE MATRIX MOLECULES (MSCRAMM GENES)																		IMMUNOD.AG.B		DEFENSIN RESIST.		TRANSFERRIN BINDING PROT		
			Ser-Asp rich fibrinogen-/bone sialoprotein-binding protein C							Ser-Asp rich fibrinogen-/bone sialoprotein-binding protein D					van Willebrand factor binding protein						immunodominant antigen B		defensin resistance protein		transferrin-binding protein		
			sdrC	sdrC (cons)	sdrC (B1)	sdrC (COL)	sdrC (Mu50)	sdrC (MW2+MRSA252+RF122)	sdrC (OtherThan252+RF122)	sdrD	sdrD (cons)	sdrD (COL+MW2)	sdrD (Mu50)	sdrD (other)	vwb	vwb (cons)	vwb (COL+MW2)	vwb (MRSA252)	vwb (Mu50)	vwb (RF122)	isaB	isaB (MRSA252)	mprF (COL+MW2)	mprF (Mu50+252)	isdA (cons)	isdA (MRSA252)	isdA (Other Than MRSA252)
Prediction for JCSC1978, GenBank AB063173.1	*SCCmec IVb/d/i (JCSC1978/6668/4469)*																										
Reference strain JCSC1978 (8/6-3P)	SCCmec IVb/d/i (JCSC1978/6668/4469)	CC8	POS	POS	NEG	POS	NEG	NEG	POS	POS	POS	POS	NEG	NEG	POS	POS	POS	NEG	NEG	NEG	AMB	POS	POS	AMB	POS	AMB	POS
Prediction for JCSC6668, GenBank AB425823.1	*SCCmec IVb/d/i (JCSC1978/6668/4469)*																										
Reference strain JCSC4469	SCCmec IVb/d/i (JCSC1978/6668/4469)	CC5	POS	POS	NEG	NEG	POS	NEG	POS	POS	POS	NEG	POS	NEG	POS	POS	NEG	NEG	POS	NEG	AMB	POS	AMB	POS	POS	AMB	POS
UKD-1103429	SCCmec IVb/d/i (JCSC1978/6668/4469)	CC7	POS	POS	NEG	POS	NEG	NEG	POS	POS	POS	NEG	POS	NEG	POS	POS	NEG	NEG	NEG	NEG	POS	AMB	POS	AMB	POS	AMB	POS
UKD-1306141	SCCmec IVb/d/i (JCSC1978/6668/4469)	CC7	POS	POS	NEG	POS	NEG	NEG	POS	POS	POS	NEG	POS	NEG	POS	POS	NEG	NEG	AMB	NEG	AMB	POS	AMB	POS	POS	AMB	POS
UKD-0500089	SCCmec IVb/d/i (JCSC1978/6668/4469)	CC5	POS	POS	NEG	NEG	NEG	NEG	POS	POS	POS	NEG	POS	NEG	POS	POS	NEG	NEG	POS	NEG	POS	POS	POS	POS	POS	NEG	POS
UKD-0501416	SCCmec IVb/d/i (JCSC1978/6668/4469)	CC5	POS	POS	NEG	NEG	POS	NEG	POS	NEG	AMB	NEG	AMB	NEG	POS	POS	NEG	NEG	POS	NEG	POS	AMB	NEG	POS	POS	NEG	POS
UKD-0934574	SCCmec IVb/d/i (JCSC1978/6668/4469)	CC5	POS	POS	NEG	NEG	POS	NEG	POS	NEG	NEG	NEG	NEG	NEG	POS	POS	NEG	NEG	POS	NEG	POS	AMB	NEG	POS	POS	NEG	POS
UKD-0746779	SCCmec IVb/d/i (JCSC1978/6668/4469)	CC45 [agr I]	POS	POS	POS	NEG	NEG	AMB	NEG	POS	POS	POS	NEG	NEG	POS	AMB	NEG	POS	NEG	NEG	NEG	POS	NEG	NEG	POS	POS	POS
UKD-0812518	SCCmec IVb/d/i (JCSC1978/6668/4469)	CC45 [agr I]	POS	POS	POS	NEG	NEG	NEG	NEG	POS	POS	POS	NEG	NEG	POS	NEG	NEG	POS	NEG	NEG	NEG	POS	NEG	POS	POS	POS	NEG
Prediction for 21209, GenBank AGRP	*SCCmec IVb/d/i (21209)*	*CC8*	*POS*	*POS*	*NEG*	*POS*	*NEG*	*NEG*	*POS*	*POS*	*POS*	*POS*	*NEG*	*NEG*	*POS*	*POS*	*POS*	*NEG*	*NEG*	*NEG*	*POS*	*NEG*	*POS*	*NEG*	*POS*	*NEG*	*POS*
UKD-0233579	SCCmec IVb/d/i (21209)	CC8	POS	POS	NEG	POS	AMB	POS	POS	POS	POS	POS	NEG	NEG	POS	POS	POS	NEG	NEG	NEG	POS	AMB	POS	AMB	POS	POS	POS
UKD-0504920	SCCmec IVb/d/i (21209)	CC8	POS	POS	NEG	POS	NEG	NEG	POS	POS	POS	AMB	NEG	NEG	POS	POS	POS	NEG	NEG	NEG	POS	AMB	POS	AMB	POS	NEG	POS
UKD-0725999	SCCmecIV b/d/i+Cu	CC30	POS	POS	NEG	NEG	NEG	NEG	NEG	POS	POS	NEG	NEG	POS	POS	POS	NEG	POS	NEG	NEG	NEG	POS	AMB	POS	POS	POS	NEG
Prediction for IS-105, GenBank AHLR	*SCCmec IVc (IS-105)*	*CC22*	*POS*	*POS*	*NEG*	*NEG*	*POS*	*NEG*	*POS*	*POS*	*POS*	*NEG*	*NEG*	*POS*	*POS*	*POS*	*NEG*	*NEG*	*NEG*	*POS*	*NEG*	*POS*	*NEG*	*POS*	*POS*	*NEG*	*POS*
UKD-0402421	SCCmec IVc (IS-105), merA/B+	CC5	POS	POS	NEG	NEG	POS	NEG	POS	POS	POS	NEG	POS	NEG	POS	POS	NEG	NEG	POS	NEG	POS	POS	POS	POS	POS	NEG	POS
UKD-0731319	SCCmec IVc (IS-105), merA/B+	CC5	POS	POS	NEG	NEG	POS	NEG	POS	POS	POS	NEG	POS	NEG	POS	POS	NEG	NEG	POS	NEG	POS	AMB	AMB	POS	POS	AMB	POS
UKD-0733137	SCCmec IVc (IS-105)	CC8	POS	POS	NEG	POS	NEG	AMB	POS	POS	POS	POS	NEG	NEG	POS	POS	POS	NEG	NEG	NEG	POS	AMB	POS	POS	POS	AMB	POS
UKD-0305690	SCCmec IVc (IS-105)	CC22	POS	POS	NEG	NEG	POS	NEG	POS	POS	POS	NEG	NEG	POS	POS	POS	NEG	NEG	NEG	POS	NEG	POS	AMB	POS	POS	NEG	POS
UKD-1424706	SCCmec IVc (IS-105)	CC22	POS	POS	NEG	NEG	POS	NEG	POS	POS	POS	NEG	NEG	POS	POS	POS	NEG	NEG	NEG	POS	NEG	POS	AMB	POS	POS	AMB	POS
Prediction for TCH60, GenBank CP002110.1	*SCCmec IVc (TCH60)*	*CC30*	*POS*	*POS*	*NEG*	*NEG*	*NEG*	*POS*	*NEG*	*POS*	*POS*	*NEG*	*NEG*	*POS*	*POS*	*POS*	*NEG*	*POS*	*NEG*	*NEG*	*NEG*	*POS*	*NEG*	*POS*	*POS*	*POS*	*NEG*
Reference strain JCSC4788	SCCmec IVc (TCH60)	CC8	POS	POS	NEG	POS	NEG	NEG	POS	POS	POS	POS	NEG	NEG	POS	POS	POS	NEG	NEG	NEG	AMB	POS	POS	AMB	POS	NEG	POS
UKD-1318318	SCCmec IVc (TCH60)	CC5	POS	POS	NEG	NEG	POS	NEG	POS	POS	POS	NEG	POS	NEG	POS	POS	NEG	NEG	POS	NEG	POS	AMB	AMB	POS	POS	NEG	POS
UKD-14580050	SCCmec IVc (TCH60)	CC5	POS	POS	NEG	NEG	POS	NEG	POS	POS	POS	NEG	POS	NEG	POS	POS	NEG	NEG	POS	NEG	AMB	POS	POS	AMB	POS	NEG	POS
UKD-08F01314	SCCmec IVc (TCH60)	CC8	POS	POS	NEG	POS	NEG	NEG	POS	POS	POS	NEG	NEG	NEG	NEG	NEG	NEG	NEG	NEG	NEG	POS	AMB	POS	NEG	POS	NEG	POS
KHDN-50361518	SCCmec IVc (TCH60)	CC8	POS	POS	NEG	POS	NEG	NEG	POS	POS	POS	POS	NEG	NEG	POS	POS	POS	NEG	NEG	NEG	AMB	POS	POS	AMB	POS	AMB	POS
UKD-0307912	SCCmec IVc (TCH60)	CC22	POS	POS	NEG	NEG	POS	NEG	POS	POS	POS	NEG	NEG	POS	POS	POS	NEG	NEG	NEG	POS	NEG	POS	POS	POS	POS	POS	POS
UKD-0220390	SCCmec IVc (TCH60)	CC45 [agr I]	POS	POS	POS	NEG	NEG	NEG	NEG	POS	POS	POS	NEG	NEG	POS	NEG	NEG	POS	NEG	NEG	NEG	POS	NEG	NEG	POS	POS	NEG
UKD-0311106	SCCmec IVc (TCH60)	CC45 [agr I]	POS	POS	POS	NEG	NEG	NEG	NEG	POS	POS	POS	NEG	NEG	POS	AMB	NEG	POS	NEG	NEG	NEG	POS	AMB	POS	POS	POS	AMB
UKD-0719855	SCCmec IVc (TCH60)	CC45 [agr I]	POS	POS	POS	NEG	NEG	AMB	NEG	POS	POS	POS	NEG	NEG	POS	NEG	NEG	POS	NEG	NEG	NEG	POS	NEG	NEG	POS	POS	NEG
UKD-0422079	SCCmec IVc (TCH60)	CC45 [agr I]	POS	POS	POS	NEG	NEG	NEG	NEG	POS	POS	POS	NEG	NEG	POS	NEG	NEG	POS	NEG	NEG	NEG	POS	NEG	NEG	POS	POS	AMB
UKD-0409235	SCCmec IVc (TCH60)	CC80	POS	POS	NEG	POS	NEG	NEG	POS	POS	POS	NEG	NEG	POS	POS	POS	NEG	POS	NEG	NEG	AMB	POS	AMB	POS	POS	NEG	POS
UKD-0409807	SCCmec IVc (TCH60)	CC80	POS	POS	NEG	POS	NEG	POS	POS	POS	POS	NEG	NEG	POS	POS	POS	NEG	POS	NEG	NEG	POS	AMB	POS	AMB	POS	AMB	POS
UKD-0416073	SCCmec IVc (TCH60)	CC80	POS	POS	NEG	POS	NEG	NEG	POS	POS	POS	NEG	NEG	POS	POS	POS	NEG	POS	NEG	NEG	POS	NEG	NEG	POS	POS	NEG	POS
UKD-0431728	SCCmec IVc (TCH60)	CC80	POS	POS	NEG	POS	NEG	NEG	POS	POS	POS	NEG	NEG	POS	POS	POS	NEG	POS	NEG	NEG	POS	AMB	NEG	POS	POS	NEG	POS
UKD-1001508	SCCmec IVc (TCH60)	CC80	POS	POS	NEG	POS	NEG	AMB	POS	POS	POS	NEG	NEG	POS	POS	POS	NEG	POS	NEG	NEG	POS	AMB	POS	AMB	POS	AMB	POS
UKD-1010412	SCCmec IVc (TCH60)	CC80	POS	POS	NEG	POS	NEG	AMB	POS	POS	POS	NEG	NEG	POS	POS	POS	NEG	POS	NEG	NEG	POS	AMB	POS	AMB	AMB	NEG	POS
UKD-12753005	SCCmec IVc (TCH60)	CC80	POS	POS	NEG	POS	NEG	NEG	POS	POS	POS	NEG	NEG	POS	POS	POS	NEG	POS	NEG	NEG	POS	AMB	POS	AMB	POS	NEG	POS
UKD-15574809	SCCmec IVc (TCH60)	CC80	POS	POS	NEG	POS	NEG	NEG	POS	POS	POS	NEG	NEG	POS	POS	POS	NEG	POS	NEG	NEG	POS	AMB	AMB	POS	POS	NEG	POS
KHDN-16120291	SCCmec IVc (TCH60)	CC88	POS	POS	NEG	NEG	POS	POS	POS	POS	POS	NEG	NEG	POS	POS	POS	NEG	NEG	POS	NEG	POS	AMB	POS	AMB	POS	NEG	POS
Prediction for WW2703/97, GenBank ACSW	*SCCmec IVc (WW2703/97)*	*CC30*	*POS*	*POS*	*NEG*	*NEG*	*NEG*	*POS*	*NEG*	*POS*	*POS*	*NEG*	*NEG*	*POS*	*POS*	*POS*	*NEG*	*POS*	*NEG*	*NEG*	*NEG*	*POS*	*NEG*	*POS*	*POS*	*POS*	*NEG*
UKD-14580088	SCCmec IVc (WW2703/97)	CC398	POS	POS	NEG	NEG	NEG	AMB	NEG	POS	POS	NEG	NEG	POS	POS	POS	NEG	NEG	NEG	NEG	NEG	POS	NEG	AMB	POS	POS	NEG
Prediction for SA_ST125_MupR, GenBank ASTH	*SCCmec IVc+speG+ccrAB4 (SA_ST125)*	*CC5*	*POS*	*POS*	*NEG*	*NEG*	*POS*	*NEG*	*POS*	*POS*	*POS*	*NEG*	*POS*	*NEG*	*POS*	*POS*	*NEG*	*NEG*	*POS*	*NEG*	*POS*	*NEG*	*POS*	*POS*	*POS*	*NEG*	*POS*
UKD-0439909	SCCmec IVc+speG+ccrAB4 (SA_ST125)	CC5	POS	POS	NEG	NEG	POS	NEG	POS	NEG	NEG	NEG	NEG	NEG	POS	POS	NEG	NEG	POS	NEG	POS	NEG	NEG	POS	POS	NEG	POS
Prediction for MRSA177, GenBank AECP	*SCCmec IVc+Cu/Hg (MRSA177)*		*POS*	*POS*	*NEG*	*POS*	*NEG*	*NEG*	*POS*	*POS*	*POS*	*POS*	*NEG*	*NEG*	*POS*	*POS*	*POS*	*NEG*	*NEG*	*NEG*	*POS*	*NEG*	*POS*	*NEG*	*POS*	*NEG*	*POS*
UKD-1279734	SCCmec IVc+Cu/Hg (MRSA177)	CC8	POS	POS	AMB	POS	NEG	NEG	POS	POS	POS	POS	NEG	NEG	POS	POS	POS	NEG	NEG	NEG	POS	AMB	AMB	POS	POS	AMB	POS
UKD-14580899	SCCmec IVc+Cu/Hg (MRSA177)	CC8	POS	POS	NEG	POS	NEG	AMB	POS	POS	POS	POS	NEG	NEG	POS	POS	POS	NEG	NEG	NEG	POS	AMB	AMB	POS	POS	AMB	POS
UKD-16002228	SCCmec IVc+Cu/Hg (MRSA177)	CC8	POS	POS	NEG	POS	NEG	AMB	POS	NEG	NEG	NEG	NEG	NEG	POS	POS	POS	NEG	NEG	NEG	POS	AMB	AMB	POS	POS	AMB	POS
Prediction for HO 5096 0412, GenBank HE681097.1	*SCCmec IVh/j (HO50960412/JCSC6670)*	*CC22*	*POS*	*POS*	*NEG*	*NEG*	*POS*	*NEG*	*POS*	*POS*	*POS*	*NEG*	*NEG*	*POS*	*POS*	*POS*	*NEG*	*NEG*	*NEG*	*POS*	*NEG*	*POS*	*NEG*	*POS*	*POS*	*NEG*	*POS*
Prediction for JCSC6670, GenBank AB425824.1	*SCCmec IVh/j (HO50960412/JCSC6670)*																										
Reference strain Dublin_DSH_E1749	SCCmec IVh/j (HO50960412/JCSC6670)		POS	POS	NEG	NEG	POS	NEG	POS	POS	POS	NEG	NEG	POS	POS	POS	NEG	NEG	NEG	POS	NEG	POS	AMB	POS	POS	NEG	POS
UKD-14576248	SCCmec IVh/j (HO50960412/JCSC6670)	CC8	POS	POS	NEG	POS	NEG	NEG	POS	NEG	NEG	NEG	NEG	NEG	POS	POS	POS	NEG	NEG	NEG	POS	AMB	POS	AMB	POS	AMB	POS
UKD-0120122	SCCmec IVh/j (HO50960412/JCSC6670)	CC22	POS	POS	NEG	NEG	POS	NEG	POS	POS	POS	NEG	NEG	POS	POS	AMB	NEG	NEG	NEG	POS	NEG	POS	NEG	POS	POS	NEG	POS
UKD-0205319	SCCmec IVh/j (HO50960412/JCSC6670)	CC22	POS	POS	NEG	NEG	POS	NEG	POS	POS	POS	NEG	NEG	POS	POS	POS	NEG	NEG	NEG	POS	NEG	POS	AMB	POS	POS	NEG	POS
UKD-0221259	SCCmec IVh/j (HO50960412/JCSC6670)	CC22	POS	POS	NEG	NEG	POS	NEG	POS	POS	POS	NEG	NEG	POS	POS	POS	NEG	NEG	NEG	POS	NEG	POS	AMB	POS	POS	NEG	POS
UKD-0418654	SCCmec IVh/j (HO50960412/JCSC6670)	CC22	POS	POS	NEG	NEG	POS	NEG	POS	POS	POS	NEG	NEG	POS	POS	POS	NEG	NEG	NEG	POS	NEG	POS	AMB	POS	POS	NEG	POS
UKD-0429721	SCCmec IVh/j (HO50960412/JCSC6670)	CC22	POS	POS	NEG	NEG	POS	NEG	POS	POS	POS	NEG	NEG	AMB	POS	POS	NEG	NEG	NEG	POS	NEG	POS	AMB	POS	POS	NEG	POS
UKD-1116194	SCCmec IVh/j (HO50960412/JCSC6670)	CC22	POS	POS	NEG	NEG	POS	NEG	POS	POS	POS	NEG	NEG	POS	POS	POS	NEG	NEG	NEG	POS	AMB	POS	POS	AMB	POS	NEG	POS
UKD-1122367	SCCmec IVh/j (HO50960412/JCSC6670)	CC22	POS	POS	NEG	NEG	POS	NEG	POS	POS	POS	NEG	NEG	POS	POS	POS	NEG	NEG	NEG	POS	AMB	POS	POS	AMB	POS	AMB	POS
UKD-14012840	SCCmec IVh/j (HO50960412/JCSC6670)	CC22	POS	POS	NEG	NEG	POS	NEG	POS	POS	POS	NEG	NEG	POS	POS	POS	NEG	NEG	NEG	POS	NEG	POS	AMB	POS	POS	AMB	POS
UKD-14579833	SCCmec IVh/j (HO50960412/JCSC6670)	CC22	POS	POS	NEG	NEG	POS	AMB	POS	POS	POS	NEG	NEG	POS	POS	POS	NEG	NEG	NEG	POS	NEG	POS	POS	AMB	POS	AMB	POS
UKD-15579198	SCCmec IVh/j (HO50960412/JCSC6670)	CC22	POS	POS	NEG	NEG	POS	AMB	POS	POS	POS	NEG	NEG	POS	POS	POS	NEG	NEG	NEG	POS	AMB	POS	POS	AMB	POS	AMB	POS
UKD-4052193	SCCmec IVh/j (HO50960412/JCSC6670)	CC22	POS	POS	AMB	NEG	POS	AMB	POS	POS	POS	NEG	NEG	POS	POS	POS	NEG	NEG	NEG	POS	AMB	POS	POS	AMB	POS	AMB	POS
UKD-124968	SCCmec IVh/j (HO50960412/JCSC6670)	CC22	POS	POS	NEG	NEG	POS	AMB	POS	POS	POS	NEG	NEG	POS	POS	POS	NEG	NEG	NEG	POS	AMB	POS	POS	AMB	POS	AMB	POS
UKD-14572546	SCCmec IVh/j (HO50960412/JCSC6670)	CC22	POS	POS	NEG	NEG	POS	POS	POS	POS	POS	NEG	NEG	POS	POS	POS	NEG	NEG	NEG	POS	AMB	POS	POS	AMB	POS	AMB	POS
UKD-14582287	SCCmec IVh/j (HO50960412/JCSC6670)	CC22	POS	POS	NEG	NEG	POS	POS	POS	POS	POS	NEG	NEG	POS	POS	POS	NEG	NEG	NEG	POS	AMB	POS	POS	AMB	POS	AMB	POS
UKD-14583376	SCCmec IVh/j (HO50960412/JCSC6670)	CC22	POS	POS	NEG	NEG	POS	POS	POS	POS	POS	NEG	NEG	POS	POS	POS	NEG	NEG	NEG	POS	AMB	POS	POS	AMB	POS	AMB	POS
UKD-14583496	SCCmec IVh/j (HO50960412/JCSC6670)	CC22	POS	POS	AMB	NEG	POS	POS	POS	POS	POS	NEG	NEG	POS	POS	POS	NEG	NEG	NEG	POS	AMB	POS	POS	AMB	POS	AMB	POS
UKD-0013324	SCCmec IVh/j (HO50960412/JCSC6670)	CC22	POS	POS	NEG	NEG	POS	NEG	POS	POS	POS	NEG	NEG	POS	POS	POS	NEG	NEG	NEG	POS	NEG	POS	NEG	POS	POS	NEG	POS
UKD-0125329	SCCmec IVh/j (HO50960412/JCSC6670)	CC22	POS	POS	NEG	NEG	POS	NEG	POS	POS	POS	NEG	NEG	POS	POS	POS	NEG	NEG	NEG	POS	NEG	POS	AMB	POS	POS	NEG	POS
UKD-0215498	SCCmec IVh/j (HO50960412/JCSC6670)	CC22	POS	POS	NEG	NEG	POS	NEG	POS	POS	POS	NEG	NEG	POS	POS	POS	NEG	NEG	NEG	POS	NEG	POS	AMB	POS	POS	NEG	POS
Prediction for M08-0126, GenBank FR753166.1	*SCCmec IVh/j+ACME2 (M08-0126)*	*CC22*																									
UKD-1379483I	SCCmec IVh/j+ACME2 (M08-0126)	CC22	POS	POS	NEG	NEG	POS	NEG	POS	POS	POS	NEG	NEG	POS	POS	POS	NEG	NEG	NEG	POS	AMB	POS	POS	AMB	POS	AMB	POS
UKD-5150204	SCCmec IV+speG+Cu/czrC+ccrAB4	CC22	POS	POS	NEG	NEG	POS	POS	POS	POS	POS	NEG	NEG	POS	POS	POS	NEG	NEG	NEG	POS	AMB	POS	POS	AMB	POS	AMB	POS
UKD-15578423	SCCmec IV+speG+czrC+ccrAB4 var. 1	CC22	POS	POS		NEG	POS	POS	POS	POS	POS	NEG	NEG	POS	POS	POS	NEG	NEG	NEG	POS	AMB	POS	AMB	POS	POS	AMB	POS
UKD-16582870	SCCmec IV+speG+czrC+ccrAB4 var. 1	CC22	POS	POS	NEG	NEG	POS	NEG	POS	POS	POS	NEG	NEG	POS	POS	POS	NEG	NEG	NEG	POS	NEG	POS	AMB	POS	POS	NEG	POS
UKD-15571940	SCCmec IV+speG+czrC+ccrAB4 var. 2	CC22	POS	POS	NEG	NEG	POS	NEG	POS	POS	POS	NEG	NEG	POS	POS	POS	NEG	NEG	NEG	POS	AMB	POS	POS	AMB	POS	AMB	POS

Isolate ID	SCCmec type	Clonal complex	ADHAESION FACTORS / GENES ENCODING MICROBIAL SURFACE COMPONENTS RECOGNIZING ADHESIVE MATRIX MOLECULES (MSCRAMM GENES)																		IMMUNOD.AG.B		DEFENSIN RESIST.		TRANSFERRIN BINDING PROT		
			Ser-Asp rich fibrinogen-/bone sialoprotein-binding protein C							Ser-Asp rich fibrinogen-/bone sialoprotein-binding protein D					van Willebrand factor binding protein						immunodominant antigen B		defensin resistance protein		transferrin-binding protein		
			sdrC	sdrC (cons)	sdrC (B1)	sdrC (COL)	sdrC (Mu50)	sdrC (MW2+MRSA252+RF122)	sdrC (OtherThan252+RF122)	sdrD	sdrD (cons)	sdrD (COL+MW2)	sdrD (Mu50)	sdrD (other)	vwb	vwb (cons)	vwb (COL+MW2)	vwb (MRSA252)	vwb (Mu50)	vwb (RF122)	isaB	isaB (MRSA252)	mprF (COL+MW2)	mprF (Mu50+252)	isdA (cons)	isdA (MRSA252)	isdA (Other Than MRSA252)
Prediction for CN1, GenBank CP003979.1	*SCCmec IVA (CN1)*	*CC8 (ST72)*	*POS*	*POS*	*NEG*	*NEG*	*POS*	*NEG*	*POS*	*POS*	*POS*	*NEG*	*POS*	*NEG*	*POS*	*POS*	*NEG*	*NEG*	*NEG*	*NEG*	*POS*	*NEG*	*POS*	*NEG*	*POS*	*NEG*	*POS*
UKD-0900871	SCCmec IVA (CN1)	CC8 (ST72)	POS	POS	NEG	NEG	POS	POS	POS	POS	POS	NEG	POS	NEG	POS	POS	NEG	NEG	NEG	NEG	POS	AMB	POS	AMB	POS	NEG	POS
Prediction for CMFT1723, GenBank HF569096.1	*SCCmec V (Bengal Bay)*																										
KHDN-10405134	SCCmec V (Bengal Bay)	CC1	POS	POS	NEG	NEG	NEG	POS	POS	POS	POS	POS	NEG	NEG	POS	POS	POS	NEG	NEG	NEG	AMB	POS	POS	AMB	POS	NEG	POS
UKD-0932405	SCCmec V (Bengal Bay)	CC1 (ST573/772)	POS	POS	NEG	NEG	POS	AMB	POS	POS	POS	NEG	NEG	POS	POS	POS	NEG	NEG	NEG	NEG	POS	AMB	POS	AMB	POS	NEG	POS
UKD-1003510	SCCmec V (Bengal Bay)	CC1 (ST573/772)	POS	POS	NEG	NEG	POS	POS	POS	POS	POS	NEG	NEG	POS	POS	POS	NEG	NEG	NEG	NEG	AMB	POS	POS	AMB	POS	AMB	POS
UKD-1112420	SCCmec V (Bengal Bay)	CC1 (ST573/772)	POS	POS	NEG	NEG	POS	POS	POS	POS	POS	NEG	NEG	POS	POS	POS	NEG	NEG	NEG	NEG	POS	AMB	POS	AMB	POS	NEG	POS
UKD-1275832	SCCmec V (Bengal Bay)	CC1 (ST573/772)	POS	POS	NEG	NEG	POS	POS	POS	POS	POS	NEG	NEG	POS	POS	POS	NEG	NEG	NEG	NEG	AMB	POS	AMB	POS	POS	AMB	POS
UKD-13023811	SCCmec V (Bengal Bay)	CC1 (ST573/772)	POS	POS	NEG	NEG	POS	POS	POS	POS	POS	NEG	NEG	POS	POS	POS	NEG	NEG	NEG	NEG	AMB	POS	POS	AMB	POS	NEG	POS
UKD-14572163	SCCmec V	CC5	POS	POS	NEG	NEG	POS	POS	POS	NEG	NEG	NEG	NEG	NEG	POS	POS	NEG	NEG	POS	NEG	AMB	POS	POS	AMB	POS	AMB	POS
KHDN-10421133	SCCmec V+fus	CC97	POS	POS	NEG	NEG	POS	POS	POS	POS	POS	NEG	POS	NEG	POS	POS	NEG	NEG	NEG	POS	POS	AMB	POS	AMB	POS	AMB	POS
KHDN-21343541	SCCmec V+fus+tir+ccrAB1	CC1	POS	POS	NEG	NEG	NEG	POS	POS	POS	POS	POS	NEG	NEG	POS	POS	POS	NEG	NEG	NEG	POS	AMB	AMB	POS	POS	AMB	POS
KHDN-10416893	SCCmec V+czrC+speG+ccrAB4	CC22	POS	POS	NEG	NEG	POS	NEG	POS	POS	POS	NEG	NEG	POS	POS	POS	NEG	NEG	NEG	POS	NEG	POS	AMB	POS	POS	AMB	POS
Prediction for GR1, GenBank AJLX	*SCCmec VT (GR1)*	*CC361*	*POS*	*POS*	*NEG*	*POS*	*NEG*	*NEG*	*POS*	*POS*	*POS*	*NEG*	*NEG*	*POS*	*POS*	*POS*	*NEG*	*NEG*	*POS*	*NEG*	*POS*	*NEG*	*POS*	*NEG*	*POS*	*NEG*	*POS*
UKD-0422872	SCCmec VT (GR1)	CC152	NEG	NEG	NEG	NEG	NEG	NEG	NEG	POS	POS	NEG	NEG	NEG	POS	POS	NEG	NEG	NEG	NEG	AMB	POS	POS	NEG	POS	NEG	NEG
Prediction for PM1, GenBank BAFA	*SCCmec VT (PM1)*	*CC59*	*POS*	*POS*	*NEG*	*NEG*	*NEG*	*POS*	*NEG*	*POS*	*POS*	*NEG*	*AMB*	*NEG*	*POS*	*POS*	*NEG*	*NEG*	*NEG*	*NEG*	*POS*	*NEG*	*POS*	*NEG*	*POS*	*NEG*	*POS*
KHDN-000871	SCCmec VT (PM1)	CC45 (agr I)	POS	POS	POS	NEG	NEG	POS	NEG	POS	POS	POS	NEG	NEG	POS	POS	NEG	POS	NEG	NEG	NEG	POS	AMB	POS	POS	POS	AMB
UKD-0725052	SCCmec VT (PM1)	CC59	POS	POS	NEG	NEG	NEG	AMB	NEG	POS	POS	NEG	NEG	NEG	POS	POS	NEG	NEG	NEG	NEG	POS	NEG	AMB	NEG	POS	NEG	POS
UKD-1312138	SCCmec VT (PM1)	CC59	POS	POS	NEG	NEG	NEG	POS	NEG	POS	POS	NEG	POS	NEG	POS	POS	NEG	NEG	NEG	NEG	POS	AMB	POS	AMB	POS	AMB	POS
UKD-Z4728	SCCmec VT (PM1)	CC59	POS	POS	NEG	NEG	NEG	POS	NEG	POS	POS	NEG	AMB	NEG	POS	POS	NEG	NEG	NEG	NEG	POS	AMB	POS	AMB	POS	AMB	POS
UKD-K0199	SCCmec VT unknown var.1	CC7	POS	POS	NEG	POS	NEG	NEG	NEG	POS	POS	NEG	POS	NEG	POS	POS	NEG	NEG	NEG	NEG	AMB	POS	POS	AMB	POS	NEG	POS
UKD-15584619	SCCmec VT unknown var.2	CC30	POS	POS	NEG	NEG	NEG	POS	NEG	POS	POS	NEG	NEG	POS	POS	POS	NEG	POS	AMB	NEG	AMB	POS	POS	AMB	POS	POS	AMB
UKD-0712568	SCCmec VT unknown var.3	CC45 (agr I)	POS	POS	POS	NEG	NEG	NEG	NEG	POS	POS	POS	NEG	NEG	POS	NEG	NEG	POS	NEG	NEG	NEG	POS	NEG	AMB	POS	POS	NEG
KHDN-10420296	SCCmec VT+ACME2	CC45 (agr I)	POS	POS	POS	NEG	NEG	POS	NEG	POS	POS	POS	NEG	NEG	POS	POS	NEG	POS	NEG	NEG	AMB	POS	AMB	POS	POS	POS	AMB
UKD-15577956	SCCmec VT+Cu/czrC+ccrA1	CC398	POS	POS	NEG	NEG	POS	POS	NEG	POS	POS	NEG	AMB	NEG	POS	POS	NEG	NEG	NEG	NEG	AMB	POS	AMB	POS	POS	POS	AMB
Prediction for S0385, GenBank AM990992	*SCCmec VT+czrC (SO385)*	*CC398*	*POS*	*POS*	*NEG*	*NEG*	*NEG*	*NEG*	*NEG*	*POS*	*POS*	*NEG*	*AMB*	*NEG*	*POS*	*POS*	*NEG*	*NEG*	*NEG*	*NEG*	*NEG*	*POS*	*NEG*	*POS*	*POS*	*POS*	*NEG*
UKD-0628560	SCCmec VT+czrC (SO385)	CC8	POS	POS	NEG	POS	NEG	NEG	POS	POS	POS	POS	NEG	NEG	POS	POS	POS	NEG	NEG	NEG	POS	AMB	POS	AMB	POS	NEG	POS
UKD-0510018	SCCmec VT+czrC (SO385)	CC398	POS	POS	NEG	NEG	NEG	NEG	NEG	POS	POS	NEG	NEG	NEG	POS	POS	NEG	NEG	NEG	NEG	NEG	POS	NEG	NEG	POS	AMB	NEG
UKD-0903558	SCCmec VT+czrC (SO385)	CC398	POS	POS	NEG	NEG	NEG	NEG	NEG	POS	POS	NEG	NEG	NEG	POS	POS	NEG	NEG	NEG	NEG	NEG	POS	NEG	NEG	POS	POS	NEG
UKD-0929815	SCCmec VT+czrC (SO385)	CC398	POS	POS	NEG	NEG	AMB	AMB	NEG	POS	POS	NEG	NEG	NEG	POS	POS	NEG	NEG	NEG	NEG	NEG	POS	NEG	NEG	POS	POS	NEG
UKD-0907762	SCCmec VT+czrC (SO385)	CC398	POS	POS	NEG	NEG	NEG	NEG	NEG	POS	POS	NEG	NEG	NEG	POS	POS	NEG	NEG	NEG	NEG	NEG	POS	NEG	POS	POS	POS	NEG
UKD-1025105	SCCmec VT+czrC (SO385)	CC398	POS	POS	NEG	NEG	NEG	NEG	NEG	POS	POS	NEG	NEG	NEG	POS	POS	NEG	NEG	NEG	NEG	NEG	POS	NEG	NEG	POS	POS	NEG
UKD-11780898	SCCmec VT+czrC (SO385)	CC398	POS	POS	NEG	NEG	POS	POS	NEG	POS	POS	NEG	POS	NEG	POS	POS	NEG	NEG	NEG	NEG	AMB	POS	AMB	POS	POS	POS	AMB
UKD-1377009	SCCmec VT+czrC (SO385)	CC398	POS	POS	NEG	NEG	POS	POS	NEG	POS	POS	NEG	NEG	NEG	POS	POS	NEG	NEG	NEG	NEG	NEG	POS	NEG	POS	POS	POS	AMB
UKD-143406	SCCmec VT+czrC (SO385)	CC398	POS	POS	NEG	NEG	POS	POS	NEG	POS	POS	NEG	POS	NEG	POS	POS	NEG	NEG	NEG	NEG	AMB	POS	AMB	POS	POS	POS	AMB
UKD-1400363	SCCmec VT+czrC (SO385)	CC398	POS	POS	NEG	NEG	POS	POS	NEG	POS	POS	NEG	NEG	NEG	POS	POS	NEG	NEG	NEG	NEG	NEG	POS	NEG	POS	POS	POS	AMB
UKD-14571852	SCCmec VT+czrC (SO385)	CC398	POS	POS	NEG	NEG	POS	POS	NEG	POS	POS	NEG	POS	NEG	POS	POS	NEG	NEG	NEG	NEG	AMB	POS	NEG	POS	POS	POS	AMB
UKD-14582574	SCCmec VT+czrC (SO385)	CC398	POS	POS	NEG	NEG	POS	POS	NEG	POS	POS	NEG	NEG	NEG	POS	POS	NEG	NEG	NEG	NEG	NEG	POS	NEG	NEG	POS	POS	AMB
UKD-152108	SCCmec VT+czrC (SO385)	CC398	POS	POS	NEG	NEG	POS	POS	NEG	POS	POS	NEG	AMB	NEG	POS	POS	NEG	NEG	NEG	NEG	NEG	POS	AMB	POS	POS	POS	AMB
UKD-15573321	SCCmec VT+czrC (SO385)	CC398	POS	POS	NEG	NEG	POS	POS	NEG	POS	POS	NEG	POS	NEG	POS	POS	NEG	NEG	NEG	NEG	AMB	POS	AMB	POS	POS	POS	AMB
UKD-15584034	SCCmec VT+czrC , ydhK-neg. variant	CC398	POS	POS	NEG	NEG	POS	POS	NEG	POS	POS	NEG	POS	NEG	POS	POS	NEG	NEG	NEG	NEG	NEG	POS	AMB	POS	POS	POS	AMB
UKD-0932581	SCCmecVT+fus+czrC	CC22	POS	POS	NEG	NEG	POS	NEG	POS	POS	POS	NEG	NEG	POS	POS	POS	NEG	NEG	NEG	POS	NEG	POS	NEG	POS	POS	NEG	POS
UKD-1121994	SCCmecVT+fus+czrC	CC22	POS	POS	NEG	NEG	POS	NEG	POS	POS	POS	NEG	NEG	POS	POS	POS	NEG	NEG	NEG	POS	NEG	POS	POS	AMB	POS	AMB	POS
UKD-1272252	SCCmecVT+fus+czrC	CC22	POS	POS	NEG	NEG	POS	NEG	POS	POS	POS	NEG	NEG	POS	POS	POS	NEG	NEG	NEG	POS	NEG	POS	POS	AMB	POS	AMB	POS
Prediction for MRSA18	*SCCmec VI+fus (MRSA18)*																										
UKD-1103380	SCCmec VI+fus (MRSA18)	CC7	POS	POS	NEG	POS	NEG	NEG	POS	POS	POS	NEG	POS	NEG	POS	POS	NEG	NEG	NEG	NEG	POS	AMB	POS	NEG	POS	NEG	POS
Prediction for M10/0061, GenBank FR823292.1	*SCCmec XI (LGA251/M10-61)*	*CC130*																									
**Reference strain Dublin-DSH_M10 0061**	**SCCmec XI (LGA251/M10-61)**	**CC130**	**POS**	**POS**	**NEG**	**POS**	**NEG**	**NEG**	**NEG**	**POS**	**POS**	**NEG**	**AMB**	**NEG**	**POS**	**POS**	**NEG**	**NEG**	**NEG**	**NEG**	**AMB**	**POS**	**POS**	**AMB**	**POS**	**NEG**	**POS**
Prediction for LGA251, GenBank FR821779.1	*SCCmec XI (LGA251/M10-61)*	*ST425*																									
UKD-14015835	SCCmec XI (LGA251/M10-61)	CC130	POS	POS	NEG	POS	NEG	NEG	NEG	POS	POS	NEG	NEG	NEG	POS	POS	NEG	NEG	NEG	NEG	AMB	POS	POS	AMB	POS	AMB	POS
UKD-16573252	SCCmec XI (LGA251/M10-61)	CC130	POS	POS	NEG	POS	NEG		NEG	POS	POS	NEG	AMB	NEG	POS	POS	NEG	NEG	NEG	NEG	AMB	POS	POS	POS	POS	NEG	POS
UKD-0217002	PseudoSCCmec, class B mec complex+Hg	CC8	POS	POS	NEG	POS	NEG	NEG	POS	POS	POS	POS	NEG	NEG	POS	POS	POS	NEG	NEG	NEG	POS	AMB	POS	AMB	POS	NEG	POS
UKD-0008510	PseudoSCCmec, class B mec complex+Hg	CC8	POS	POS	NEG	POS	NEG	NEG	POS	POS	POS	POS	NEG	NEG	POS	POS	POS	NEG	NEG	NEG	POS	NEG	POS	NEG	POS	NEG	POS
UKD-0110962	PseudoSCCmec, class B mec complex+Hg	CC8	POS	POS	NEG	POS	NEG	AMB	POS	POS	POS	POS	NEG	NEG	POS	POS	POS	NEG	NEG	NEG	POS	AMB	POS	AMB	POS	NEG	POS
KHDN-10408593	PseudoSCCmec, class B mec complex+Cu/Hg	CC8	POS	POS	NEG	POS	NEG	NEG	POS	POS	POS	POS	NEG	NEG	POS	POS	POS	NEG	NEG	NEG	AMB	POS	POS	AMB	POS	NEG	POS
UKD-0007201	PseudoSCCmec, class B mec complex	CC5	POS	POS	NEG	NEG	POS	NEG	POS	POS	POS	NEG	POS	NEG	POS	POS	NEG	NEG	POS	NEG	POS	AMB	AMB	POS	POS	NEG	POS
UKD-0033204	PseudoSCCmec, class B mec complex	CC5	POS	POS	NEG	NEG	POS	NEG	POS	POS	POS	NEG	POS	NEG	POS	POS	NEG	NEG	POS	NEG	POS	AMB	AMB	POS	POS	NEG	POS
UKD-0134329	PseudoSCCmec, class B mec complex	CC5	POS	AMB	NEG	NEG	POS	NEG	POS	NEG	AMB	NEG	AMB	NEG	POS	AMB	NEG	NEG	POS	NEG	POS	AMB	NEG	POS	POS	NEG	POS
UKD-0806946	PseudoSCCmec class C+As/Cu	CC398	POS	POS	NEG	NEG	POS	POS	NEG	POS	POS	NEG	NEG	NEG	POS	POS	NEG	NEG	NEG	NEG	NEG	POS	NEG	NEG	POS	POS	NEG
UKD-0500610	ACME2	CC8	POS	POS	NEG	POS	NEG	NEG	POS	POS	POS	POS	NEG	NEG	POS	POS	POS	NEG	NEG	NEG	POS	AMB	POS	AMB	POS	NEG	POS
UKD-0610438	ACME2	CC8	AMB	POS	NEG	POS	NEG	NEG	POS	NEG	NEG	NEG	NEG	NEG	POS	POS	POS	NEG	NEG	NEG	POS	AMB	POS	AMB	POS	NEG	POS
KHDN-10409344	ACME2	CC8	AMB	POS	AMB	POS	NEG	NEG	POS	POS	POS	POS	NEG	NEG	POS	POS	POS	NEG	NEG	NEG	POS	AMB	POS	POS	POS	NEG	POS
UKD-0322559	ACME2+speG+czrC+ccrAB4	CC8	POS	POS	NEG	POS	NEG	NEG	POS	POS	POS	POS	NEG	NEG	POS	POS	POS	NEG	NEG	NEG	POS	POS	POS	AMB	POS	NEG	POS
UKD-0501998	ACME2+speG+czrC+ccrAB4	CC8	AMB	POS	NEG	POS	NEG	NEG	POS	POS	POS	POS	NEG	NEG	POS	POS	POS	NEG	NEG	NEG	POS	AMB	POS	AMB	POS	NEG	POS
UKD-0505213	ACME2+speG+czrC+ccrAB4	CC8	POS	POS	NEG	POS	NEG	AMB	POS	POS	POS	POS	NEG	NEG	POS	POS	POS	NEG	NEG	NEG	POS	AMB	POS	AMB	POS	POS	POS
Prediction for MSSA476, GenBank BX571857.1	*fus+tir+ccrAB1 (MSSA476)*	*CC1*	*POS*	*POS*	*NEG*	*NEG*	*NEG*	*POS*	*POS*	*POS*	*POS*	*POS*	*NEG*	*NEG*	*POS*	*POS*	*POS*	*NEG*	*NEG*	*NEG*	*POS*	*NEG*	*POS*	*NEG*	*POS*	*NEG*	*POS*
**Reference strain Sanger 476**	**fus+tir+ccrAB1 (MSSA476)**	**CC1**	**POS**	**POS**	**NEG**	**NEG**	**NEG**	**POS**	**POS**	**POS**	**POS**	**POS**	**NEG**	**NEG**	**POS**	**POS**	**POS**	**NEG**	**NEG**	**NEG**	**POS**	**AMB**	**POS**	**NEG**	**POS**	**NEG**	**POS**
UKD-14023914	fus+tir+ccrAB1 (MSSA476)	CC1	POS	POS	NEG	NEG	NEG	POS	POS	NEG	NEG	NEG	NEG	NEG	POS	POS	POS	NEG	NEG	NEG	POS	AMB	POS	AMB	POS	AMB	POS
UKD-K00034	kdp+ccrAB2	CC182	POS	POS	NEG	NEG	NEG	NEG	NEG	NEG	NEG	NEG	NEG	NEG	AMB	POS	NEG	NEG	NEG	NEG	POS	POS	NEG	AMB	POS	NEG	POS
UKD-15580369	As+ccrAB4	CC22	POS	POS	NEG	NEG	POS	POS	POS	NEG	NEG	NEG	NEG	NEG	POS	POS	NEG	NEG	NEG	POS	AMB	POS	POS	AMB	POS	AMB	POS
UKD-15570100	ACME3+ccrAB1 (Strain 21342)	CC80	POS	POS	NEG	POS	NEG	POS	POS	POS	POS	NEG	NEG	POS	POS	POS	NEG	POS	NEG	NEG	POS	AMB	POS	AMB	POS	AMB	POS
UKD-1010678	speG+czrC+ccrAB4	CC8	POS	POS	NEG	POS	NEG	NEG	POS	POS	POS	POS	NEG	NEG	POS	POS	POS	NEG	NEG	NEG	POS	AMB	POS	AMB	POS	NEG	POS
UKD-1008243	speG+czrC+ccrAB4	CC8	POS	POS	NEG	POS	NEG	NEG	POS	POS	POS	POS	NEG	NEG	POS	POS	POS	NEG	NEG	NEG	POS	AMB	POS	AMB	POS	NEG	POS
UKD-1009430	speG+czrC+ccrAB4	CC8	POS	POS	NEG	POS	NEG	NEG	POS	POS	POS	POS	NEG	NEG	POS	POS	POS	NEG	NEG	NEG	POS	AMB	POS	AMB	POS	NEG	POS
UKD-12071828	speG+czrC+ccrAB4	CC22	POS	POS	NEG	NEG	POS	NEG	POS	POS	POS	NEG	NEG	POS	POS	POS	NEG	NEG	NEG	POS	NEG	POS	POS	POS	POS	NEG	POS
UKD-14011883	speG+czrC+ccrAB4	CC22	POS	POS	NEG	NEG	POS	NEG	POS	NEG	NEG	NEG	NEG	NEG	POS	POS	NEG	NEG	NEG	POS	NEG	POS	POS	AMB	POS	AMB	POS
UKD-15008293	speG+czrC+ccrAB4	CC22	POS	POS	NEG	NEG	POS	NEG	POS	NEG	NEG	NEG	NEG	NEG	POS	POS	NEG	NEG	NEG	POS	NEG	POS	POS	POS	POS	AMB	POS
UKD-1514877	speG+czrC+ccrAB4	CC22	POS	POS	NEG	NEG	POS	NEG	POS	NEG	NEG	NEG	NEG	NEG	POS	POS	NEG	NEG	NEG	POS	NEG	POS	POS	POS	POS	NEG	POS
UKD-15580971	speG+czrC+ccrAB4	CC22	POS	POS	NEG	NEG	POS	NEG	POS	NEG	NEG	NEG	NEG	NEG	POS	POS	NEG	NEG	NEG	POS	NEG	POS	POS	AMB	POS	AMB	POS
UKD-15582646	speG+czrC+ccrAB4	CC22	POS	POS	NEG	NEG	POS	NEG	POS	POS	POS	NEG	NEG	POS	POS	POS	NEG	NEG	NEG	POS	NEG	POS	POS	AMB	POS	AMB	POS

Isolate ID	SCCmec type	Clonal complex	PUTATIVE TRANSPORTER				TYPE I RESTRICTION-MODIFICATION SYSTEM, SINGLE SEQUENCE SPECIFICITY PROTEIN													MISCELLANEOUS GENES				HYALURONATE LYASES							
			hypothetical protein, similar to integral membrane protein LmrP				type I site-specific deoxyribo-nuclease subunit, 1st locus	type I site-specific deoxyribonuclease subunit, 2nd locus				type I site-specific deoxyribonuclease subunit, 3rd locus					type I site-specific deoxyribonuclease subunit, unknown locus			ear2 = Q2FXC0	Q2YUB3	Q7A4X2	sdrM / tetEfflux	hysA1 (MRSA252)	hysA1 (MRSA252+RF122) and/or hysA2 (cons)	hysA1 (MRSA252+RF122) and/or hysA2 (COL+USA300)	hysA2 (All Other Than MRSA252)	hysA2 (COL+USA300+NCTC)	hysA2 (All Other Than COL+USA300+NCTC)	hysA2-AllOtherThan COL+USA300+NCTC	hysA2 (MRSA252)
			lmrP (OtherThanRF122)	lmrP (OtherThanRF122)	lmrP (RF122)	lmrP (RF122)	hsdS1-RF122	hsdS2-ST5+ST8	hsdS2-MW2+476	hsdS2-RF122	hsdS2-MRSA252	hsdS3-AllOtherThanRF122+252	hsdS3-ST8+ST1+RF122	hsdS3-Mu50+N315	hsdS3-CC51+252	hsdS3-MRSA252	hsdSx-CC25	hsdSx-CC15	hsdSx-etd												
VIRTUAL HYBRIDISATION for COL, GenBank CP000046	SCCmec I (COL)	CC8	POS	POS	NEG	NEG	NEG	POS	NEG	NEG	NEG	POS	POS	NEG	NEG	NEG	POS	NEG	NEG	POS	NEG	NEG	POS	NEG	POS	NEG	POS	POS	NEG	NEG	NEG
Reference strain COL	SCCmec I (COL)	CC8	POS	POS	NEG	NEG	NEG	POS	NEG	NEG	NEG	POS	POS	NEG	NEG	NEG	POS	NEG	NEG	POS	NEG	NEG	POS	NEG	POS	POS	POS	POS	NEG	NEG	NEG
UKD-0007063	SCCmec I (COL)	CC5	POS	POS	NEG	NEG	NEG	NEG	AMB	NEG	NEG	POS	NEG	POS	AMB	NEG	POS	POS	NEG	NEG	AMB	POS	POS	NEG	POS	NEG	POS	NEG	POS	POS	NEG
UKD-0220759	SCCmec I (COL)	CC5	POS	POS	NEG	NEG	NEG	NEG	NEG	NEG	NEG	POS	NEG	POS	NEG	NEG	POS	NEG	NEG	NEG	NEG	POS	POS	NEG	POS	NEG	NEG	NEG	POS	POS	NEG
UKD-0308986	SCCmec I (COL)	CC5	POS	POS	NEG	NEG	NEG	NEG	NEG	NEG	NEG	POS	NEG	POS	NEG	NEG	POS	AMB	NEG	NEG	AMB	POS	POS	NEG	POS	NEG	AMB	NEG	POS	POS	NEG
UKD-0500009	SCCmec I (COL)	CC5	POS	POS	NEG	NEG	NEG	NEG	NEG	NEG	NEG	POS	NEG	POS	NEG	NEG	POS	NEG	NEG	NEG	NEG	POS	POS	NEG	POS	NEG	POS	NEG	POS	POS	NEG
UKD-0508483	SCCmec I (COL)	CC5	POS	POS	NEG	NEG	NEG	NEG	NEG	NEG	NEG	POS	NEG	POS	NEG	NEG	POS	NEG	NEG	NEG	NEG	NEG	POS	NEG	POS	NEG	NEG	NEG	POS	POS	NEG
UKD-0519001	SCCmec I (COL)	CC5	POS	AMB	NEG	NEG	NEG	NEG	NEG	NEG	NEG	POS	NEG	POS	NEG	NEG	POS	NEG	NEG	NEG	NEG	NEG	POS	NEG	POS	NEG	NEG	NEG	POS	POS	NEG
UKD-0701876	SCCmec I (COL)	CC5	POS	POS	NEG	NEG	NEG	NEG	POS	NEG	NEG	POS	NEG	POS	NEG	NEG	POS	AMB	NEG	NEG	NEG	NEG	POS	NEG	POS	NEG	POS	NEG	POS	POS	AMB
UKD-0708865	SCCmec I (COL)	CC5	POS	NEG	NEG	NEG	NEG	NEG	NEG	NEG	NEG	POS	NEG	POS	NEG	NEG	POS	NEG	NEG	NEG	NEG	NEG		NEG	POS	NEG	NEG	NEG	AMB	AMB	NEG
UKD-1001782	SCCmec I (COL)	CC5	POS	POS	NEG	NEG	NEG	NEG	NEG	NEG	NEG	POS	NEG	POS	NEG	NEG	POS	NEG	NEG	NEG	AMB	POS	POS	NEG	POS	NEG	POS	NEG	POS	POS	AMB
Prediction for PSP1996, GenBank ANHU	SCCmec I (PSP1996)	CC8	POS	POS	NEG	NEG	NEG	POS	NEG	NEG	NEG	POS	POS	NEG	NEG	NEG	POS	NEG	NEG	POS	NEG	NEG	POS	NEG	POS	NEG	POS	POS	NEG	NEG	NEG
UKD-0261727	SCCmec I (PSP1996)	CC8	POS	POS	NEG	NEG	NEG	POS	NEG	NEG	NEG	POS	POS	NEG	NEG	NEG	POS	NEG	NEG	POS	NEG	NEG	POS	NEG	POS	POS	POS	POS	NEG	NEG	NEG
VIRTUAL HYBRIDISATION for MRSA7 : VIRTUAL HYBRII	SCCmec I+fus+tirS (Geraldine Clone)	CC5	POS	POS	NEG	NEG	NEG	POS	NEG	NEG	NEG	POS	NEG	POS	NEG	NEG	POS	NEG	NEG	NEG	NEG	POS	POS	NEG	POS	NEG	POS	NEG	AMB	POS	NEG
UKD-1221930	SCCmec I+fus+tirS (Geraldine Clone)	CC5	POS	POS	NEG	NEG	NEG	POS	NEG	NEG	NEG	POS	NEG	POS	NEG	NEG	POS	NEG	NEG	NEG	AMB	POS	POS	NEG	POS	NEG	POS	NEG	POS	POS	NEG
Prediction for N315, GenBank BA000018.3	SCCmec II (N315)	CC5	POS	POS	NEG	NEG	NEG	POS	NEG	NEG	NEG	POS	NEG	POS	NEG	NEG	POS	NEG	NEG	NEG	NEG	POS	POS	NEG	POS	NEG	POS	NEG	AMB	POS	NEG
Reference strain N315	SCCmec II (N315)	CC5	POS	POS	NEG	NEG	NEG	POS	NEG	NEG	NEG	POS	NEG	POS	NEG	NEG	POS	NEG	NEG	NEG	NEG	POS	POS	NEG	POS	NEG	POS	NEG	POS	POS	NEG
Prediction for MRSA252, GenBank BX571856.1	SCCmec II (N315)	CC30	POS	POS	NEG	NEG	NEG	NEG	NEG	NEG	POS	NEG	NEG	NEG	POS	POS	AMB	NEG	NEG	NEG	NEG	POS	POS	POS	POS	POS	NEG	NEG	POS	AMB	POS
Reference strain Sanger 252	SCCmec II (N315)	CC30	POS	POS	NEG	NEG	NEG	NEG	NEG	NEG	POS	NEG	NEG	NEG	POS	POS	NEG	NEG	NEG	NEG	NEG	POS	POS	NEG	POS	POS	NEG	POS	POS	POS	POS
UKD-0230792	SCCmec II (N315)	CC30	POS	POS	NEG	NEG	NEG	NEG	NEG	NEG	POS	NEG	NEG	NEG	POS	POS	POS	NEG	NEG	NEG	NEG	POS	POS	POS	POS	POS	NEG	POS	POS	POS	POS
Prediction for Mu50, GenBank BA000017.4	SCCmec II (N315)	CC5	POS	POS	NEG	NEG	NEG	POS	NEG	NEG	NEG	POS	NEG	POS	NEG	NEG	POS	NEG	NEG	NEG	NEG	POS	POS	NEG	POS	NEG	POS	NEG	AMB	POS	NEG
Reference strain MU50	SCCmec II (N315)	CC5	POS	POS	NEG	NEG	NEG	POS	NEG	NEG	NEG	POS	NEG	POS	NEG	NEG	POS	NEG	NEG	NEG	NEG	POS	POS	NEG	POS	NEG	POS	NEG	POS	POS	NEG
Prediction for JH1, GenBank CP000736.1	SCCmec II (JH1/JH9)	CC5	POS	POS	NEG	NEG	NEG	POS	NEG	NEG	NEG	POS	NEG	POS	NEG	NEG	POS	NEG	NEG	NEG	NEG	POS	POS	NEG	POS	NEG	POS	NEG	AMB	POS	NEG
Prediction for JH9, GenBank CP000703.1	SCCmec II (JH1/JH9)	CC5	POS	POS	NEG	NEG	NEG	POS	NEG	NEG	NEG	POS	NEG	POS	NEG	NEG	POS	NEG	NEG	NEG	NEG	POS	POS	NEG	POS	NEG	POS	NEG	AMB	POS	NEG
Reference strain Dublin-DSH_AR07.4_0237	SCCmec II (JH1/JH9)		POS	POS	NEG	NEG	NEG	POS	NEG	NEG	NEG	POS	NEG	POS	NEG	NEG	POS	NEG	NEG	NEG	AMB	POS	POS	NEG	POS	NEG	POS	NEG	POS	POS	NEG
Reference strain NARSA_382 / USA100	SCCmec II (JH1/JH9)	CC5	POS	POS	NEG	NEG	NEG	POS	NEG	NEG	NEG	POS	NEG	POS	NEG	NEG	POS	NEG	NEG	NEG	NEG	POS	POS	NEG	POS	NEG	POS	NEG	POS	POS	AMB
UKD-0046921	SCCmec II (JH1/JH9)	CC5	POS	POS	NEG	NEG	NEG	POS	NEG	NEG	NEG	POS	NEG	POS	NEG	NEG	POS	NEG	NEG	NEG	NEG	POS	POS	NEG	POS	NEG	POS	NEG	POS	POS	NEG
UKD-0333415	SCCmec II (JH1/JH9)	CC5	POS	POS	NEG	NEG	NEG	POS	NEG	NEG	NEG	POS	NEG	POS	NEG	NEG	POS	NEG	NEG	NEG	NEG	POS	POS	NEG	POS	NEG	POS	NEG	POS	POS	NEG
UKD-0341645	SCCmec II (JH1/JH9)	CC5	POS	POS	NEG	NEG	NEG	POS	NEG	NEG	NEG	POS	NEG	POS	NEG	NEG	POS	NEG	NEG	NEG	NEG	POS	POS	NEG	POS	NEG	POS	NEG	POS	POS	NEG
UKD-0504626	SCCmec II (JH1/JH9)	CC5	POS	POS	NEG	NEG	NEG	POS	NEG	NEG	NEG	POS	NEG	POS	NEG	NEG	POS	NEG	NEG	NEG	NEG	POS	POS	NEG	POS	NEG	AMB	NEG	POS	POS	NEG
UKD-0507581	SCCmec II (JH1/JH9)	CC5	POS	POS	NEG	NEG	NEG	POS	NEG	NEG	NEG	POS	NEG	POS	NEG	NEG	POS	NEG	NEG	NEG	NEG	POS	POS	NEG	POS	NEG	POS	NEG	POS	POS	NEG
UKD-0507903	SCCmec II (JH1/JH9)	CC5	POS	POS	NEG	NEG	NEG	POS	NEG	NEG	NEG	POS	NEG	POS	NEG	NEG	POS	NEG	NEG	NEG	NEG	POS	POS	NEG	POS	NEG	POS	NEG	POS	POS	NEG
UKD-0700817	SCCmec II (JH1/JH9)	CC5	POS	NEG	NEG	NEG	NEG	POS	NEG	NEG	NEG	POS	NEG	POS	NEG	NEG	POS	NEG	NEG	NEG	NEG	POS	POS	NEG	POS	NEG	NEG	NEG	POS	POS	NEG
UKD-1014561	SCCmec II (JH1/JH9)	CC5	POS	POS	NEG	NEG	NEG	POS	NEG	NEG	NEG	POS	NEG	POS	NEG	NEG	POS	NEG	NEG	NEG	NEG	POS	POS	NEG	POS	NEG	POS	NEG	POS	POS	NEG
UKD-1037507	SCCmec II (JH1/JH9)	CC5	POS	POS	NEG	NEG	NEG	POS	AMB	NEG	NEG	POS	NEG	POS	NEG	NEG	POS	NEG	NEG	NEG	AMB	POS	POS	NEG	POS	NEG	POS	NEG	POS	POS	AMB
UKD-1038321	SCCmec II (JH1/JH9)	CC5	POS	POS	NEG	NEG	NEG	POS	NEG	NEG	NEG	POS	NEG	POS	NEG	NEG	POS	NEG	NEG	NEG	NEG	POS	POS	NEG	POS	NEG	POS	NEG	POS	POS	NEG
UKD-1111608	SCCmec II (JH1/JH9)	CC5	POS	POS	NEG	NEG	NEG	POS	AMB	NEG	NEG	POS	NEG	POS	NEG	NEG	POS	NEG	NEG	NEG	NEG	POS	POS	NEG	POS	NEG	POS	NEG	POS	POS	NEG
UKD-12753135	SCCmec II (JH1/JH9)	CC5	POS	POS	NEG	NEG	NEG	POS	NEG	NEG	NEG	POS	NEG	POS	NEG	NEG	POS	NEG	NEG	NEG	AMB	POS	POS	NEG	POS	NEG	POS	NEG	POS	POS	NEG
UKD-1312816	SCCmec II (JH1/JH9)	CC5	POS	POS	NEG	NEG	NEG	POS	NEG	NEG	NEG	POS	NEG	POS	NEG	NEG	POS	NEG	NEG	NEG	NEG	POS	POS	NEG	POS	NEG	POS	NEG	POS	POS	NEG
UKD-1401623	SCCmec II (JH1/JH9)	CC5	POS	POS	NEG	NEG	NEG	POS	NEG	NEG	NEG	POS	NEG	POS	NEG	NEG	POS	NEG	NEG	NEG	NEG	POS	POS	NEG	POS	NEG	POS	NEG	POS	POS	POS
UKD-15571943	SCCmec II+czrC+ccrA/B-4	CC5	POS	POS	NEG	NEG	NEG	POS	AMB	NEG	NEG	POS	NEG	POS	NEG	NEG	POS	AMB	NEG	NEG	POS	POS	POS	NEG	POS	NEG	POS	NEG	POS	POS	AMB
UKD-15576888	SCCmec II+czrC+ccrA/B-4	CC5	POS	POS	NEG	NEG	NEG	POS	AMB	NEG	NEG	POS	NEG	POS	NEG	NEG	POS	NEG	NEG	NEG	NEG	POS	POS	NEG	POS	NEG	POS	NEG	POS	POS	NEG
UKD-14576461	SCCmec II+speG+czrC+ccrA/B-4	CC5	POS	POS	NEG	NEG	NEG	POS	NEG	NEG	NEG	POS	NEG	POS	NEG	NEG	POS	NEG	NEG	NEG	AMB	POS	POS	NEG	POS	NEG	POS	NEG	POS	POS	NEG
UKD-15581649	SCCmec II+speG+czrC+ccrA/B-4	CC5	POS	POS	NEG	NEG	NEG	POS	NEG	NEG	NEG	POS	NEG	POS	NEG	NEG	POS	NEG	NEG	NEG	NEG	POS	POS	NEG	POS	NEG	POS	NEG	POS	POS	NEG
Prediction for CIGC345D, GenBank AHVO	SCCmec II A/B/D	CC8	POS	POS	NEG	NEG	NEG	POS	NEG	NEG	NEG	POS	POS	NEG	NEG	NEG	POS	NEG	NEG	POS	NEG	NEG	POS	NEG	POS	NEG	POS	POS	NEG	NEG	NEG
UKD-12K00171	SCCmec II A/B/D	CC8	POS	POS	NEG	NEG	NEG	POS	NEG	NEG	NEG	POS	POS	NEG	NEG	NEG	POS	NEG	NEG	POS	NEG	NEG	POS	NEG	POS	POS	POS	POS	NEG	NEG	NEG
Prediction for JKD6008, GenBank CP002120.1	SCC [mec III+Cd] (JKD6008)	CC8 (ST239)	POS	POS	NEG	NEG	NEG	POS	NEG	NEG	NEG	NEG	NEG	NEG	NEG	NEG	AMB	NEG	NEG	NEG	NEG	NEG	POS	NEG	POS	NEG	POS	POS	NEG	NEG	NEG
Prediction for JKD6009, GenBank ABSA	SCC [mec III+Cd] (JKD6008)	CC8 (ST239)	POS	POS	NEG	NEG	NEG	POS	NEG	NEG	NEG	NEG	NEG	NEG	NEG	NEG	AMB	NEG	NEG	NEG	NEG	NEG	POS	NEG	POS	NEG	POS	POS	NEG	NEG	NEG
Reference strain ATCC_33592	SCC [mec III+Cd] (ATCC33592)	CC8 (ST239)	POS	POS	NEG	NEG	NEG	POS	NEG	NEG	NEG	POS	POS	NEG	NEG	NEG	POS	NEG	NEG	POS	NEG	NEG	POS	NEG	POS	POS	POS	POS	NEG	NEG	NEG
Prediction for PPUKM-775-2009, GenBank AMRE	SCC [mec III+Cd+ccrC] (PPUKM-775-2009)	CC8 (ST239)	POS	POS	NEG	NEG	NEG	POS	NEG	NEG	NEG	POS	POS	NEG	NEG	NEG	POS	NEG	NEG	POS	NEG	NEG	POS	NEG	POS	NEG	POS	POS	NEG	NEG	NEG
Prediction for T0131, GenBank CP002643.1	SCC [mec III+Cd+ccrC] (T0131)	CC8 (ST239)	POS	POS	NEG	NEG	NEG	POS	NEG	NEG	NEG	POS	POS	NEG	NEG	NEG	POS	NEG	NEG	POS	NEG	NEG	POS	NEG	POS	NEG	POS	POS	NEG	NEG	NEG
Prediction for XN108, GenBank CP007447.1	SCC [mec III+Cd+ccrC] (XN108)	CC8 (ST239)	POS	POS	NEG	NEG	NEG	POS	NEG	NEG	NEG	POS	POS	NEG	NEG	NEG	POS	NEG	NEG	POS	NEG	NEG	POS	NEG	POS	NEG	POS	POS	NEG	NEG	NEG
Prediction for CN79, GenBank ANCJ	SCC [mec III+Cd+ccrC] (CN79)	CC8 (ST239)	POS	POS	NEG	NEG	NEG	POS	NEG	NEG	NEG	POS	POS	NEG	NEG	NEG	POS	NEG	NEG	POS	NEG	NEG	POS	NEG	POS	NEG	POS	POS	NEG	NEG	NEG
Prediction for 16K, GenBank BABZ	SCC [mec III+Cd+ccrC] (CN79)	CC8 (ST239)	POS	POS	NEG	NEG	NEG	POS	NEG	NEG	NEG	POS	POS	NEG	NEG	NEG	POS	NEG	NEG	POS	NEG	NEG	POS	NEG	POS	NEG	POS	POS	NEG	NEG	NEG
UKD-0835987	SCC [mec III+Cd+ccrC] (CN79)	CC8 (ST239)	POS	POS	NEG	NEG	NEG	POS	NEG	NEG	NEG	POS	POS	NEG	NEG	NEG	POS	NEG	NEG	POS	NEG	NEG	POS	NEG	POS	POS	POS	POS	NEG	NEG	NEG
UKD-16581769	SCCmec III+Cd+ccrC	CC8 (ST239)	POS	POS	NEG	NEG	NEG	POS	NEG	NEG	NEG	POS	POS	NEG	NEG	NEG	POS	NEG	NEG	POS	NEG	NEG	POS	NEG	POS	POS	POS	POS	NEG	NEG	NEG
Prediction for Bmb9393, GenBank CP005288.1	SCCmec III+Cd/Hg+ccrC (Bmb9393)	CC8 (ST239)	POS	POS	NEG	NEG	NEG	POS	NEG	NEG	NEG	POS	POS	NEG	NEG	NEG	POS	NEG	NEG	POS	NEG	NEG	POS	NEG	POS	NEG	POS	POS	NEG	NEG	NEG
UKD-15574263	SCCmec III+Cd/Hg+ccrC (Bmb9393)	CC8 (ST239)	POS	POS	NEG	NEG	NEG	POS	NEG	NEG	NEG	POS	POS	NEG	NEG	NEG	POS	NEG	NEG	POS	NEG	NEG	POS	NEG	POS	POS	POS	POS	NEG	NEG	AMB
Prediction for SK1585, GenBank AYLT	SCCmec III+ccrC+Cd/Hg (SK1585)	CC8 (ST239)	POS	POS	NEG	NEG	NEG	POS	NEG	NEG	NEG	AMB	NEG	NEG	POS	NEG	AMB	NEG	NEG	NEG	NEG	POS	POS	NEG	NEG	NEG	POS	AMB	NEG	NEG	NEG
UKD-105861	SCCmec III+ccrC+Cd/Hg (SK1585)	CC8 (ST239)	POS	POS	NEG	NEG	NEG	NEG	NEG	NEG	NEG	POS	POS	NEG	NEG	NEG	POS	NEG	NEG	POS	NEG	NEG	POS	NEG	POS	POS	POS	POS	NEG	NEG	NEG
UKD-134572	SCCmec III+ccrC+Cd/Hg (SK1585)	CC8 (ST239)	POS	NEG	NEG	NEG	NEG	NEG	NEG	NEG	NEG	POS	POS	NEG	NEG	NEG	POS	NEG	NEG	POS	NEG	NEG	POS	NEG	POS	POS	NEG	POS	NEG	NEG	NEG
UKD-139143	SCCmec III+ccrC+Cd/Hg (SK1585)	CC8 (ST239)	POS	POS	NEG	NEG	NEG	NEG	NEG	NEG	NEG	POS	POS	NEG	NEG	NEG	POS	NEG	NEG	POS	NEG	NEG	POS	NEG	POS	POS	POS	POS	NEG	NEG	NEG
Prediction for TW20, GenBank FN433596.1	SCC [mec III+Cd/Hg+ccrC] (TW20)	CC8 (ST239)	POS	POS	NEG	NEG	NEG	POS	NEG	NEG	NEG	POS	POS	NEG	NEG	NEG	POS	NEG	NEG	POS	NEG	NEG	POS	NEG	POS	NEG	POS	POS	NEG	NEG	NEG
Prediction for ATCC BAA-39, GenBank AEEK	SCC [mec III+Cd/Hg+ccrC] (TW20)	CC8 (ST239)	POS	POS	NEG	NEG	NEG	POS	NEG	NEG	NEG	POS	POS	NEG	NEG	NEG	POS	NEG	NEG	POS	NEG	NEG	POS	NEG	POS	NEG	POS	POS	NEG	NEG	NEG

Isolate ID	SCCmec type	Clonal complex	PUTATIVE TRANSPORTER				TYPE I RESTRICTION-MODIFICATION SYSTEM, SINGLE SEQUENCE SPECIFICITY PROTEIN													MISCELLANEOUS GENES				HYALURONATE LYASES							
			hypothetical protein, similar to integral membrane protein LmrP				type I site-specific deoxyribo-nuclease subunit, 1st locus	type I site-specific deoxyribonuclease subunit, 2nd locus				type I site-specific deoxyribonuclease subunit, 3rd locus					type I site-specific deoxyribonuclease subunit, unknown locus			ear2 = Q2FXC0	Q2YUB3	Q7A4X2	sdrM / tetEfflux	hysA1 (MRSA252)	hysA1 (MRSA252+RF122) and/or hysA2 (cons)	hysA1 (MRSA252+RF122) and/or hysA2 (COL+USA300)	hysA2 (All Other Than MRSA252)	hysA2 (COL+USA300+NCTC)	hysA2 (All Other Than COL+USA300+NCTC)	hysA2-AllOtherThan COL+USA300+NCTC	hysA2 (MRSA252)
			lmrP (OtherThanRF122)	lmrP (OtherThanRF122)	lmrP (RF122)	lmrP (RF122)	hsdS1-RF122	hsdS2-ST5+ST8	hsdS2-MW2+476	hsdS2-RF122	hsdS2-MRSA252	hsdS3-AllOtherThanRF122+252	hsdS3-ST8+ST1+RF122	hsdS3-Mu50+N315	hsdS3-CC51+252	hsdS3-MRSA252	hsdSx-CC25	hsdSx-CC15	hsdSx-etd												
Prediction for MW2, GenBank BA000033.2	*SCCmec IVa (MW2)*	*CC1*	*POS*	*POS*	*NEG*	*NEG*	*NEG*	*NEG*	*POS*	*NEG*	*NEG*	*POS*	*POS*	*NEG*	*NEG*	*NEG*	*POS*	*NEG*	*NEG*	*POS*	*NEG*	*NEG*	*POS*	*NEG*	*POS*	*NEG*	*POS*	*NEG*	*POS*	*AMB*	*NEG*
**Reference strain MW2**	**SCCmec IVa (MW2)**	**CC1**	**POS**	**POS**	**NEG**	**NEG**	**NEG**	**NEG**	**POS**	**NEG**	**NEG**	**POS**	**POS**	**NEG**	**NEG**	**NEG**	**POS**	**NEG**	**NEG**	**POS**	**NEG**	**NEG**	**POS**	**NEG**	**POS**	**NEG**	**POS**	**NEG**	**POS**	**POS**	**NEG**
**Reference strain CA05 (JCSC1968)**	**SCCmec IVa (MW2)**	**CC45**	**POS**	**POS**	**NEG**	**NEG**	**NEG**	**NEG**	**NEG**	**NEG**	**POS**	**NEG**	**NEG**	**NEG**	**NEG**	**NEG**	**POS**	**NEG**	**NEG**	**NEG**	**NEG**	**POS**	**POS**	**NEG**	**POS**	**NEG**	**NEG**	**NEG**	**POS**	**NEG**	**NEG**
**UKD-0127106**	SCCmec IVa (MW2)	CC1	POS	POS	NEG	NEG	NEG	NEG	POS	NEG	NEG	POS	POS	NEG	NEG	NEG	POS	NEG	NEG	POS	NEG	NEG	POS	NEG	POS	NEG	POS	NEG	POS	POS	NEG
**UKD-0701446**	SCCmec IVa (MW2)	CC1	POS	POS	NEG	NEG	NEG	NEG	NEG	NEG	NEG	POS	POS	POS	NEG	NEG	POS	NEG	NEG	POS	NEG	NEG	POS	NEG	POS	NEG	POS	NEG	POS	POS	NEG
**UKD-0947486**	SCCmec IVa (MW2)	CC1	POS	POS	NEG	NEG	NEG	NEG	POS	NEG	NEG	POS	POS	NEG	NEG	NEG	POS	NEG	NEG	POS	NEG	NEG	POS	NEG	POS	NEG	POS	NEG	POS	POS	NEG
**UKD-1031910**	SCCmec IVa (MW2)	CC1	POS	POS	NEG	NEG	NEG	NEG	POS	NEG	NEG	POS	POS	NEG	NEG	NEG	POS	NEG	NEG	POS	NEG	NEG	POS	NEG	POS	NEG	POS	NEG	POS	POS	NEG
**UKD-14571328**	SCCmec IVa (MW2)	CC1	POS	POS	NEG	NEG	NEG	NEG	POS	NEG	NEG	POS	POS	NEG	NEG	NEG	POS	NEG	NEG	POS	NEG	NEG	POS	NEG	POS	NEG	POS	NEG	POS	POS	POS
**UKD-03003012**	SCCmec IVa (MW2)	CC5	POS	POS	NEG	NEG	NEG	POS	AMB	NEG	NEG	POS	NEG	POS	NEG	NEG	POS	NEG	NEG	NEG	NEG	POS	POS	NEG	POS	NEG	AMB	NEG	POS	POS	AMB
**UKD-15582582**	SCCmec IVa (MW2)	CC6	POS	POS	NEG	NEG	NEG	NEG	NEG	NEG	NEG	POS	POS	NEG	POS	NEG	POS	POS	NEG	POS	NEG	NEG	POS	NEG	POS	NEG	POS	NEG	POS	POS	NEG
**UKD-16574230**	SCCmec IVa (MW2)	CC6	POS	POS	NEG	NEG	NEG	NEG	POS	NEG	NEG	POS	POS	NEG	POS	NEG	POS	AMB	NEG	POS	NEG	NEG	POS	NEG	POS	NEG	NEG	NEG	POS	POS	NEG
**UKD-16582560**	SCCmec IVa (MW2)	CC6	POS	POS	NEG	NEG	NEG	NEG	NEG	NEG	NEG	POS	POS	NEG	POS	NEG	POS	AMB	NEG	POS	NEG	NEG	POS	NEG	POS	NEG	NEG	NEG	POS	POS	NEG
**UKD-0802855**	SCCmec IVa (MW2)	CC7	POS	POS	NEG	NEG	NEG	NEG	NEG	NEG	NEG	NEG	NEG	NEG	NEG	NEG	NEG	NEG	NEG	NEG	NEG	POS	POS	NEG	POS	NEG	NEG	NEG	POS	POS	NEG
**KHDN-10405258**	SCCmec IVa (MW2)	CC8	POS	POS	NEG	NEG	NEG	POS	NEG	NEG	NEG	POS	POS	NEG	NEG	NEG	POS	NEG	NEG	POS	NEG	NEG	POS	NEG	POS	POS	POS	POS	NEG	NEG	NEG
**UKD-15573497**	SCCmec IVa (MW2)	CC22	POS	POS	NEG	NEG	NEG	POS	NEG	NEG	NEG	POS	NEG	NEG	NEG	NEG	POS	POS	NEG	NEG	POS	POS	NEG	NEG	POS	NEG	POS	NEG	POS	POS	POS
**UKD-0634729**	SCCmec IVa (MW2)	CC30	POS	POS	NEG	NEG	NEG	NEG	NEG	NEG	POS	AMB	NEG	NEG	POS	POS	POS	POS	NEG	NEG	NEG	POS	POS	POS	POS	POS	NEG	POS	POS	POS	POS
**UKD-1119771**	SCCmec IVa (MW2)	CC30	POS	POS	NEG	NEG	NEG	NEG	AMB	NEG	POS	NEG	NEG	NEG	POS	POS	POS	NEG	NEG	NEG	AMB	POS	POS	POS	POS	POS	NEG	POS	POS	POS	POS
**KHDN-10411458**	SCCmec IVa (MW2)	CC30	POS	POS	NEG	NEG	NEG	NEG	NEG	NEG	POS	NEG	NEG	NEG	POS	POS	POS	NEG	NEG	NEG	NEG	POS	POS	POS	POS	POS	NEG	POS	POS	POS	POS
**KHDN-398609**	SCCmec IVa (MW2)	CC30	POS	POS	NEG	NEG	NEG	NEG	NEG	NEG	POS	NEG	NEG	NEG	POS	POS	POS	AMB	NEG	NEG	AMB	POS	POS	POS	POS	POS	NEG	POS	POS	POS	POS
**KHDN-01081998**	SCCmec IVa (MW2)	CC30	POS	POS	NEG	NEG	NEG	NEG	NEG	NEG	POS	NEG	NEG	NEG	POS	POS	POS	AMB	NEG	NEG	AMB	POS	POS	POS	POS	POS	NEG	POS	POS	POS	POS
**KHDN-10399223**	SCCmec IVa (MW2)	CC30	POS	POS	NEG	NEG	NEG	NEG	NEG	NEG	POS	NEG	NEG	NEG	POS	POS	POS	NEG	NEG	NEG	NEG	POS	POS	POS	POS	POS	NEG	POS	POS	POS	POS
**KHDN-W261158**	SCCmec IVa (MW2)	CC30	POS	POS	NEG	NEG	NEG	NEG	NEG	NEG	POS	NEG	NEG	NEG	POS	POS	POS	NEG	NEG	NEG	NEG	POS	POS	POS	POS	POS	NEG	POS	POS	POS	POS
**UKD-0002046**	SCCmec IVa (MW2)	CC45 [agr I]	POS	POS	NEG	NEG	NEG	NEG	NEG	NEG	POS	NEG	NEG	NEG	NEG	NEG	POS	NEG	NEG	NEG	NEG	NEG	POS	NEG	POS	NEG	AMB	NEG	POS	NEG	NEG
**UKD-0029618**	SCCmec IVa (MW2)	CC45 [agr I]	POS	POS	NEG	NEG	NEG	NEG	NEG	NEG	POS	NEG	NEG	NEG	NEG	NEG	AMB	NEG	NEG	NEG	NEG	POS	POS	NEG	POS	NEG	AMB	NEG	AMB	NEG	NEG
**UKD-0043085**	SCCmec IVa (MW2)	CC45 [agr I]	POS	POS	NEG	NEG	NEG	NEG	NEG	NEG	POS	NEG	NEG	NEG	NEG	NEG	AMB	NEG	NEG	NEG	NEG	POS	POS	NEG	POS	NEG	AMB	NEG	POS	NEG	NEG
**UKD-0047053**	SCCmec IVa (MW2)	CC45 [agr I]	POS	NEG	NEG	NEG	NEG	NEG	NEG	NEG	POS	NEG	NEG	NEG	NEG	NEG	NEG	NEG	NEG	NEG	NEG	NEG	NEG	NEG	NEG	NEG	NEG	NEG	NEG	NEG	NEG
**UKD-0047429**	SCCmec IVa (MW2)	CC45 [agr I]	POS	POS	NEG	NEG	NEG	NEG	NEG	NEG	POS	NEG	NEG	NEG	NEG	NEG	AMB	NEG	NEG	NEG	NEG	POS	POS	NEG	POS	NEG	AMB	NEG	POS	NEG	NEG
**UKD-0112465**	SCCmec IVa (MW2)	CC45 [agr I]	POS	POS	NEG	NEG	NEG	NEG	NEG	NEG	POS	NEG	NEG	NEG	NEG	NEG	NEG	NEG	NEG	NEG	NEG	NEG	POS	NEG	POS	NEG	NEG	NEG	AMB	NEG	NEG
**UKD-0103483**	SCCmec IVa (MW2)	CC45 [agr I]	POS	POS	NEG	NEG	NEG	NEG	NEG	NEG	POS	NEG	NEG	NEG	NEG	NEG	NEG	NEG	NEG	NEG	NEG	AMB	NEG	NEG	AMB	NEG	NEG	NEG	NEG	NEG	NEG
**UKD-0305014**	SCCmec IVa (MW2)	CC45 [agr I]	POS	POS	NEG	NEG	NEG	NEG	NEG	NEG	POS	NEG	NEG	NEG	NEG	NEG	POS	NEG	NEG	NEG	NEG	POS	POS	NEG	POS	NEG	AMB	NEG	POS	NEG	NEG
**UKD-0401119**	SCCmec IVa (MW2)	CC45 [agr I]	POS	POS	NEG	NEG	NEG	NEG	NEG	NEG	POS	NEG	NEG	NEG	NEG	NEG	AMB	NEG	NEG	NEG	NEG	NEG	POS	NEG	POS	NEG	AMB	NEG	POS	NEG	NEG
**UKD-0537969**	SCCmec IVa (MW2)	CC45 [agr I]	POS	POS	NEG	NEG	NEG	NEG	NEG	NEG	POS	NEG	NEG	NEG	NEG	NEG	POS	NEG	NEG	NEG	NEG	NEG	POS	NEG	POS	NEG	NEG	NEG	POS	NEG	NEG
**UKD-0703373**	SCCmec IVa (MW2)	CC45 [agr I]	POS	AMB	NEG	NEG	NEG	NEG	NEG	NEG	AMB	NEG	NEG	NEG	NEG	NEG	NEG	NEG	NEG	NEG	NEG	NEG	AMB	NEG	NEG	NEG	NEG	NEG	NEG	NEG	NEG
**UKD-0813653**	SCCmec IVa (MW2)	CC45 [agr I]	POS	POS	NEG	NEG	NEG	NEG	NEG	NEG	POS	NEG	NEG	NEG	NEG	NEG	POS	NEG	NEG	NEG	NEG	POS	POS	NEG	POS	NEG	NEG	NEG	POS	NEG	NEG
**UKD-0839175**	SCCmec IVa (MW2)	CC45 [agr I]	POS	POS	NEG	NEG	NEG	NEG	POS	NEG	POS	NEG	NEG	NEG	NEG	NEG	POS	POS	NEG	NEG	POS	NEG	POS	NEG	POS	NEG	POS	NEG	POS	NEG	NEG
**UKD-1108422**	SCCmec IVa (MW2)	CC45 [agr I]	POS	POS	NEG	NEG	NEG	NEG	POS	NEG	POS	NEG	NEG	NEG	NEG	NEG	POS	POS	NEG	NEG	POS	NEG	POS	NEG	POS	NEG	POS	NEG	POS	NEG	NEG
**UKD-1380645**	SCCmec IVa (MW2)	CC45 [agr I]	POS	POS	NEG	NEG	NEG	NEG	NEG	NEG	POS	NEG	NEG	NEG	NEG	NEG	POS	NEG	NEG	NEG	NEG	POS	POS	NEG	POS	NEG	AMB	NEG	POS	NEG	NEG
**UKD-148016**	SCCmec IVa (MW2)	CC45 [agr I]	POS	POS	NEG	NEG	NEG	NEG	NEG	NEG	POS	NEG	NEG	NEG	NEG	NEG	POS	POS	NEG	NEG	AMB	POS	POS	NEG	POS	NEG	POS	NEG	POS	NEG	NEG
**UKD-16571773**	SCCmec IVa (MW2)	CC45 [agr I]	POS	POS	NEG	NEG	NEG	NEG	NEG	NEG	POS	NEG	NEG	NEG	NEG	NEG	POS	POS	NEG	AMB	NEG	POS	POS	NEG	POS	NEG	POS	NEG	POS	NEG	NEG
**KHDN-10407738**	SCCmec IVa (MW2)	CC45 [ST617]	NEG	NEG	POS	POS	NEG	NEG	NEG	NEG	POS	POS	NEG	NEG	POS	NEG	POS	AMB	NEG	NEG	NEG	POS	POS	NEG	POS	POS	POS	POS	NEG	POS	NEG
**UKD-0853670**	SCCmec IVa (MW2)	CC88	POS	POS	NEG	NEG	POS	NEG	NEG	NEG	NEG	POS	NEG	NEG	NEG	POS	POS	NEG	NEG	NEG	NEG	NEG	POS	NEG	POS	NEG	NEG	NEG	POS	POS	NEG
**UKD-1417352**	SCCmec IVa (MW2)	CC88	POS	POS	NEG	NEG	POS	NEG	NEG	NEG	NEG	POS	NEG	NEG	NEG	POS	POS	NEG	NEG	NEG	AMB	NEG	POS	NEG	POS	NEG	POS	NEG	POS	POS	NEG
**UKD-15571283**	SCCmec IVa (MW2)	CC88	POS	POS	NEG	NEG	POS	NEG	POS	NEG	NEG	POS	NEG	NEG	NEG	POS	POS	POS	NEG	NEG	POS	NEG	POS	NEG	POS	NEG	POS	NEG	POS	POS	AMB
**KHDN-10406719**	SCCmec IVa (MW2)	ST93	POS	POS	NEG	NEG	NEG	NEG	NEG	NEG	NEG	NEG	NEG	NEG	NEG	POS	NEG	AMB	NEG	NEG	NEG	POS	POS	NEG	POS	POS	NEG	POS	NEG	NEG	POS
**KHDN-0060294**	SCCmec IVa (MW2)	ST93	POS	POS	NEG	NEG	NEG	NEG	NEG	NEG	NEG	NEG	NEG	NEG	NEG	POS	NEG	NEG	NEG	NEG	NEG	POS	POS	NEG	POS	POS	NEG	POS	NEG	NEG	NEG
**UKD-0734190**	SCCmec IVa (MW2)	CC97	POS	POS	NEG	NEG	NEG	NEG	NEG	NEG	NEG	NEG	NEG	NEG	NEG	NEG	POS	NEG	NEG	NEG	NEG	POS	POS	NEG	POS	NEG	NEG	NEG	POS	POS	NEG
**UKD-13006983**	SCCmec IVa (MW2)	CC97	POS	POS	NEG	NEG	NEG	NEG	NEG	NEG	NEG	POS	NEG	NEG	NEG	NEG	POS	NEG	NEG	NEG	POS	POS	POS	NEG	POS	NEG	POS	NEG	POS	POS	AMB
Prediction for H131520133	*SCCmec IVa (H131520133)*	*CC152*	*NEG*	*NEG*	*POS*	*POS*	*NEG*	*POS*	*NEG*	*NEG*	*NEG*	*NEG*	*NEG*	*NEG*	*POS*	*POS*	*AMB*	*NEG*	*POS*	*NEG*	*NEG*	*NEG*	*POS*	*NEG*	*POS*	*NEG*	*NEG*	*AMB*	*NEG*	*NEG*	*POS*
**KHDN-325892**	SCCmec IVa (H131520133)	CC30	POS	POS	NEG	NEG	NEG	NEG	NEG	NEG	POS	NEG	NEG	NEG	POS	POS	POS	AMB	NEG	NEG	AMB	POS	POS	POS	POS	POS	NEG	POS	POS	POS	POS
**UKD-15580207**	SCCmec IVa (H131520133)	CC88	POS	POS	NEG	NEG	POS	NEG	AMB	NEG	NEG	POS	NEG	NEG	NEG	POS	POS	AMB	NEG	NEG	NEG	NEG	POS	NEG	POS	NEG	POS	NEG	POS	POS	NEG
**UKD-0521159**	SCCmec Iva	CC8	POS	POS	NEG	NEG	NEG	POS	NEG	NEG	NEG	POS	POS	NEG	NEG	NEG	POS	NEG	NEG	POS	NEG	NEG	POS	NEG	POS	POS	AMB	POS	NEG	NEG	NEG
Prediction for CMFT503 : HF569113.1	*SCCmec IVa (CMFT503)*																														
Prediction for 21343, GenBank AHKV	*SCCmec IVa (CMFT503)*	*CC88*	*POS*	*POS*	*NEG*	*NEG*	*POS*	*NEG*	*NEG*	*NEG*	*NEG*	*NEG*	*NEG*	*NEG*	*NEG*	*POS*	*AMB*	*NEG*	*NEG*	*NEG*	*NEG*	*NEG*	*POS*	*NEG*	*POS*	*NEG*	*POS*	*NEG*	*AMB*	*POS*	*NEG*
**UKD-1420740**	SCCmec IVa (CMFT503)	CC22	POS	POS	AMB	NEG	NEG	POS	POS	NEG	NEG	POS	NEG	NEG	NEG	NEG	POS	POS	NEG	NEG	POS	POS	NEG	NEG	POS	NEG	AMB	NEG	POS	POS	POS
**UKD-15575124**	SCCmec IVa (CMFT503)	CC22	POS	POS	NEG	NEG	NEG	POS	NEG	NEG	NEG	POS	NEG	NEG	NEG	NEG	POS	AMB	NEG	NEG	NEG	POS	NEG	NEG	POS	NEG	NEG	NEG	POS	POS	POS
**UKD-16584947**	SCCmec IVa (CMFT503)	CC22	POS	POS	NEG	NEG	NEG	POS	NEG	NEG	NEG	NEG	NEG	NEG	NEG	NEG	AMB	NEG	NEG	NEG	NEG	POS	NEG	NEG	POS	NEG	NEG	NEG	POS	POS	NEG
**UKD-16575077**	SCCmec IVa (CMFT503)	CC88	POS	POS	NEG	NEG	POS	NEG	NEG	NEG	NEG	POS	NEG	NEG	NEG	POS	POS	NEG	NEG	NEG	NEG	NEG	POS	NEG	POS	NEG	POS	NEG	POS	POS	NEG
Prediction for TCH1516, GenBank CP000730.1	*SCCmec IVa+ACME1+Cu (USA300)*	*CC8*	*POS*	*POS*	*NEG*	*NEG*	*NEG*	*POS*	*NEG*	*NEG*	*NEG*	*POS*	*POS*	*NEG*	*NEG*	*NEG*	*POS*	*NEG*	*NEG*	*POS*	*NEG*	*NEG*	*POS*	*NEG*	*POS*	*NEG*	*POS*	*POS*	*NEG*	*NEG*	*NEG*
**Reference strain USA300-TCH1516**	SCCmec IVa+ACME1+Cu (USA300)	CC8	POS	POS	NEG	NEG	NEG	POS	AMB	NEG	NEG	POS	POS	NEG	NEG	NEG	POS	NEG	NEG	POS	NEG	NEG	POS	NEG	POS	POS	POS	POS	NEG	NEG	NEG
Prediction for FPR3757, GenBank CP000255.1	*SCCmec IVa+ACME1+Cu (USA300)*	*CC8*	*POS*	*POS*	*NEG*	*NEG*	*NEG*	*POS*	*NEG*	*NEG*	*NEG*	*POS*	*POS*	*NEG*	*NEG*	*NEG*	*POS*	*NEG*	*NEG*	*POS*	*NEG*	*NEG*	*POS*	*NEG*	*POS*	*NEG*	*POS*	*POS*	*NEG*	*NEG*	*NEG*
**UKD-0516386**	SCCmec IVa+ACME1+Cu (USA300)	CC8	POS	NEG	NEG	NEG	NEG	POS	NEG	NEG	NEG	POS	POS	NEG	NEG	NEG	POS	NEG	NEG	POS	NEG	NEG	POS	NEG	POS	POS	NEG	POS	NEG	NEG	NEG
**UKD-0815773**	SCCmec IVa+ACME1+Cu (USA300)	CC8	POS	POS	NEG	NEG	NEG	POS	NEG	NEG	NEG	POS	POS	NEG	NEG	NEG	POS	NEG	NEG	POS	AMB	NEG	POS	NEG	POS	POS	POS	POS	NEG	NEG	NEG
**UKD-1414654**	SCCmec IVa+ACME1+Cu (USA300)	CC8	POS	POS	NEG	NEG	NEG	POS	AMB	NEG	NEG	POS	POS	NEG	NEG	NEG	POS	NEG	NEG	POS	NEG	NEG	POS	NEG	POS	POS	POS	POS	NEG	NEG	POS
**UKD-0408036**	SCCmec IVa+ACME2	CC45 [agr I]	POS	POS	NEG	NEG	NEG	NEG	NEG	NEG	POS	NEG	NEG	NEG	NEG	NEG	POS	AMB	NEG	NEG	NEG	NEG	POS	NEG	POS	NEG	POS	NEG	POS	NEG	NEG
**UKD-0044035**	SCCmec IVa+ccrC (Hannover)	CC8	POS	POS	NEG	NEG	NEG	POS	NEG	NEG	NEG	POS	POS	NEG	NEG	NEG	POS	NEG	NEG	POS	NEG	NEG	POS	NEG	POS	POS	POS	POS	NEG	NEG	NEG
**UKD-0136287**	SCCmec IVa+ccrC (Hannover)	CC8	POS	POS	NEG	NEG	NEG	POS	NEG	NEG	NEG	POS	POS	NEG	NEG	NEG	POS	NEG	NEG	POS	NEG	NEG	POS	NEG	POS	POS	POS	POS	NEG	NEG	NEG

Isolate ID	SCCmec type	Clonal complex	PUTATIVE TRANSPORTER				TYPE I RESTRICTION-MODIFICATION SYSTEM, SINGLE SEQUENCE SPECIFICITY PROTEIN													MISCELLANEOUS GENES				HYALURONATE LYASES							
			hypothetical protein, similar to integral membrane protein LmrP				type I site-specific deoxyribo-nuclease subunit, 1st locus	type I site-specific deoxyribonuclease subunit, 2nd locus				type I site-specific deoxyribonuclease subunit, 3rd locus					type I site-specific deoxyribonuclease subunit, unknown locus			ear2 = Q2FXC0	Q2YUB3	Q7A4X2	sdrM / tetEfflux	hysA1 (MRSA252)	hysA1 (MRSA252+RF122) and/or hysA2 (cons)	hysA1 (MRSA252+RF122) and/or hysA2 (COL+USA300)	hysA2 (All Other Than MRSA252)	hysA2 (COL+USA300+NCTC)	hysA2 (All Other Than COL+USA300+NCTC)	hysA2-AllOtherThan COL+USA300+NCTC	hysA2 (MRSA252)
			lmrP (OtherThanRF122)	lmrP (OtherThanRF122)	lmrP (RF122)	lmrP (RF122)	hsdS1-RF122	hsdS2-ST5+ST8	hsdS2-MW2+476	hsdS2-RF122	hsdS2-MRSA252	hsdS3-AllOtherThanRF122+252	hsdS3-ST8+ST1+RF122	hsdS3-Mu50+N315	hsdS3-CC51+252	hsdS3-MRSA252	hsdSx-CC25	hsdSx-CC15	hsdSx-etd												
Prediction for JCSC1978, GenBank AB063173.1	SCCmec IVb/d/i (JCSC1978/6668/4469)																														
Reference strain JCSC1978 (8/6-3P)	SCCmec IVb/d/i (JCSC1978/6668/4469)	CC8	POS	POS	NEG	NEG	NEG	POS	NEG	NEG	NEG	POS	POS	NEG	NEG	NEG	POS	NEG	NEG	POS	NEG	NEG	POS	NEG	POS	POS	POS	POS	NEG	NEG	NEG
Prediction for JCSC6668, GenBank AB425823.1	SCCmec IVb/d/i (JCSC1978/6668/4469)																														
Reference strain JCSC4469	SCCmec IVb/d/i (JCSC1978/6668/4469)	CC5	POS	POS	NEG	NEG	NEG	POS	NEG	NEG	NEG	POS	NEG	POS	NEG	NEG	POS	NEG	NEG	NEG	NEG	POS	POS	NEG	POS	NEG	POS	NEG	POS	POS	NEG
UKD-1103429	SCCmec IVb/d/i (JCSC1978/6668/4469)	CC7	POS	POS	NEG	NEG	NEG	NEG	AMB	NEG	NEG	NEG	NEG	NEG	NEG	NEG	NEG	NEG	POS	NEG	NEG	POS	POS	NEG	POS	NEG	NEG	NEG	POS	POS	NEG
UKD-1306141	SCCmec IVb/d/i (JCSC1978/6668/4469)	CC7	POS	POS	NEG	NEG	NEG	NEG	NEG	NEG	NEG	NEG	NEG	NEG	NEG	NEG	POS	NEG	POS	NEG	AMB	POS	POS	NEG	POS	NEG	AMB	NEG	POS	POS	NEG
UKD-0500089	SCCmec IVb/d/i (JCSC1978/6668/4469)	CC5	POS	POS	NEG	NEG	NEG	POS	NEG	NEG	NEG	POS	NEG	POS	NEG	NEG	POS	NEG	NEG	NEG	NEG	POS	POS	NEG	POS	NEG	NEG	NEG	POS	POS	NEG
UKD-0501416	SCCmec IVb/d/i (JCSC1978/6668/4469)	CC5	POS	POS	NEG	NEG	NEG	POS	NEG	NEG	NEG	POS	NEG	POS	NEG	NEG	POS	NEG	NEG	NEG	NEG	POS	AMB	NEG	POS	NEG	POS	NEG	POS	POS	NEG
UKD-0934574	SCCmec IVb/d/i (JCSC1978/6668/4469)	CC5	POS	POS	NEG	NEG	NEG	POS	NEG	NEG	NEG	POS	NEG	POS	NEG	NEG	POS	NEG	NEG	NEG	NEG	POS	POS	NEG	POS	NEG	POS	NEG	POS	POS	NEG
UKD-0746779	SCCmec IVb/d/i (JCSC1978/6668/4469)	CC45 [agr I]	POS	POS	NEG	NEG	POS	NEG	NEG	NEG	POS	NEG	NEG	NEG	NEG	NEG	POS	NEG	NEG	NEG	NEG	POS	POS	NEG	POS	NEG	NEG	NEG	POS	NEG	NEG
UKD-0812518	SCCmec IVb/d/i (JCSC1978/6668/4469)	CC45 [agr I]	POS	POS	NEG	NEG	POS	NEG	NEG	NEG	POS	NEG	NEG	NEG	NEG	NEG	POS	NEG	NEG	NEG	NEG	POS	POS	NEG	POS	NEG	NEG	NEG	POS	NEG	NEG
Prediction for 21209, GenBank AGRP	SCCmec IVb/d/i (21209)	CC8	POS	POS	NEG	NEG	NEG	POS	NEG	NEG	NEG	POS	POS	NEG	NEG	NEG	POS	NEG	NEG	POS	NEG	NEG	POS	NEG	POS	NEG	POS	POS	NEG	NEG	NEG
UKD-0233579	SCCmec IVb/d/i (21209)	CC8	POS	POS	NEG	NEG	NEG	POS	NEG	NEG	NEG	POS	POS	NEG	NEG	NEG	POS	NEG	NEG	POS	AMB	NEG	POS	NEG	POS	POS	POS	POS	NEG	NEG	POS
UKD-0504920	SCCmec IVb/d/i (21209)	CC8	POS	POS	NEG	NEG	NEG	POS	NEG	NEG	NEG	POS	POS	NEG	NEG	NEG	POS	NEG	NEG	POS	NEG	NEG	POS	NEG	POS	POS	AMB	POS	NEG	NEG	NEG
UKD-0725999	SCCmecIV b/d/i+Cu	CC30	POS	POS	NEG	NEG	NEG	NEG	NEG	NEG	POS	NEG	NEG	NEG	POS	POS	POS	NEG	NEG	NEG	NEG	POS	POS	AMB	POS	POS	NEG	POS	POS	POS	POS
Prediction for IS-105, GenBank AHLR	SCCmec IVc (IS-105)	CC22	POS	POS	NEG	NEG	NEG	POS	NEG	NEG	NEG	AMB	NEG	NEG	NEG	NEG	POS	NEG	NEG	NEG	NEG	NEG	NEG	NEG	POS	NEG	NEG	NEG	POS	AMB	NEG
UKD-0402421	SCCmec IVc (IS-105), merA/B+	CC5	POS	POS	NEG	NEG	NEG	POS	NEG	NEG	NEG	POS	NEG	POS	NEG	NEG	POS	NEG	NEG	NEG	NEG	POS	POS	NEG	POS	NEG	POS	NEG	POS	POS	NEG
UKD-0731319	SCCmec IVc (IS-105), merA/B+	CC5	POS	POS	NEG	NEG	NEG	POS	NEG	NEG	NEG	POS	NEG	POS	NEG	NEG	POS	NEG	NEG	NEG	NEG	POS	POS	NEG	POS	NEG	POS	NEG	POS	POS	NEG
UKD-0733137	SCCmec IVc (IS-105)	CC8	POS	POS	NEG	NEG	NEG	POS	NEG	NEG	NEG	POS	POS	NEG	NEG	NEG	POS	NEG	NEG	POS	NEG	NEG	POS	NEG	POS	POS	POS	POS	NEG	NEG	AMB
UKD-0305690	SCCmec IVc (IS-105)	CC22	POS		NEG	NEG	NEG	POS	NEG	NEG	NEG	NEG	NEG	NEG	NEG	NEG	NEG	NEG	NEG	NEG	NEG	POS	NEG	NEG	POS	NEG	NEG	NEG	POS	POS	AMB
UKD-1424706	SCCmec IVc (IS-105)	CC22	POS	POS	NEG	NEG	NEG	POS	NEG	NEG	NEG	POS	NEG	NEG	NEG	NEG	POS	POS	NEG	NEG	AMB	POS	NEG	NEG	POS	NEG	NEG	NEG	POS	POS	AMB
Prediction for TCH60, GenBank CP002110.1	SCCmec IVc (TCH60)	CC30	POS	POS	NEG	NEG	NEG	NEG	NEG	NEG	POS	NEG	NEG	NEG	POS	POS	AMB	NEG	NEG	NEG	NEG	POS	POS	POS	POS	POS	NEG	NEG	POS	AMB	POS
Reference strain JCSC4788	SCCmec IVc (TCH60)	CC8	POS	POS	NEG	NEG	NEG	POS	NEG	NEG	NEG	POS	POS	NEG	NEG	NEG	POS	NEG	NEG	POS	NEG	NEG	POS	NEG	NEG	NEG	NEG	NEG	NEG	NEG	NEG
UKD-1318318	SCCmec IVc (TCH60)	CC5	POS	POS	NEG	NEG	NEG	POS	NEG	NEG	NEG	POS	NEG	POS	NEG	NEG	POS	NEG	NEG	NEG	NEG	POS	POS	NEG	POS	NEG	POS	NEG	POS	POS	NEG
UKD-14580050	SCCmec IVc (TCH60)	CC5	POS	POS	NEG	NEG	NEG	POS	NEG	NEG	NEG	POS	NEG	POS	NEG	NEG	POS	NEG	NEG	NEG	NEG	POS	POS	NEG	POS	NEG	POS	NEG	POS	POS	NEG
UKD-08F01314	SCCmec IVc (TCH60)	CC8	POS	NEG	NEG	NEG	NEG	POS	NEG	NEG	NEG	POS	POS	NEG	NEG	NEG	POS	NEG	NEG	POS	NEG	NEG	POS	NEG	POS	NEG	NEG	POS	NEG	NEG	NEG
KHDN-50361518	SCCmec IVc (TCH60)	CC8	POS	POS	NEG	NEG	NEG	POS	NEG	NEG	NEG	POS	POS	NEG	NEG	NEG	POS	NEG	NEG	POS	AMB	NEG	POS	NEG	POS	POS	POS	POS	NEG	NEG	NEG
UKD-0307912	SCCmec IVc (TCH60)	CC22	POS	POS	NEG	NEG	NEG	POS	NEG	NEG	NEG	POS	NEG	NEG	NEG	NEG	POS	NEG	NEG	NEG	NEG	POS	NEG	NEG	POS	NEG	NEG	NEG	POS	POS	NEG
UKD-0220390	SCCmec IVc (TCH60)	CC45 [agr I]	POS	POS	NEG	NEG	NEG	NEG	NEG	NEG	POS	NEG	NEG	NEG	NEG	NEG	NEG	NEG	NEG	NEG	NEG	POS	AMB	NEG	AMB	NEG	NEG	NEG	NEG	NEG	NEG
UKD-0311106	SCCmec IVc (TCH60)	CC45 [agr I]	POS	POS	NEG	NEG	NEG	NEG	AMB	NEG	POS	NEG	NEG	NEG	NEG	NEG	POS	AMB	NEG	NEG	POS	POS	POS	NEG	POS	NEG	POS	NEG	POS	NEG	NEG
UKD-0719855	SCCmec IVc (TCH60)	CC45 [agr I]	POS	POS	NEG	NEG	NEG	NEG	NEG	NEG	POS	NEG	NEG	NEG	NEG	NEG	NEG	NEG	NEG	NEG	NEG	POS	AMB	NEG	NEG	NEG	NEG	NEG	POS	NEG	NEG
UKD-0422079	SCCmec IVc (TCH60)	CC45 [agr I]	POS	POS	NEG	NEG	NEG	NEG	NEG	NEG	POS	NEG	NEG	NEG	NEG	NEG	AMB	NEG	NEG	NEG	NEG	POS	POS	NEG	POS	NEG	NEG	NEG	POS	NEG	NEG
UKD-0409235	SCCmec IVc (TCH60)	CC80	POS	POS	NEG	NEG	NEG	NEG	NEG	NEG	NEG	NEG	NEG	NEG	NEG	NEG	AMB	AMB	POS	NEG	NEG	NEG	POS	NEG	POS	NEG	POS	NEG	POS	POS	POS
UKD-0409807	SCCmec IVc (TCH60)	CC80	POS	POS	NEG	NEG	NEG	NEG	POS	NEG	NEG	NEG	NEG	NEG	NEG	NEG	POS	POS	POS	NEG	NEG	NEG	POS	NEG	POS	NEG	POS	NEG	POS	POS	POS
UKD-0416073	SCCmec IVc (TCH60)	CC80	POS	POS	NEG	NEG	NEG	NEG	NEG	NEG	NEG	NEG	NEG	NEG	NEG	NEG	NEG	NEG	POS	NEG	NEG	NEG	POS	NEG	POS	NEG	AMB	NEG	POS	POS	NEG
UKD-0431728	SCCmec IVc (TCH60)	CC80	POS	POS	NEG	NEG	NEG	NEG	NEG	NEG	NEG	NEG	NEG	NEG	NEG	NEG	NEG	NEG	AMB	NEG	NEG	NEG	POS	NEG	POS	NEG	POS	NEG	POS	POS	NEG
UKD-1001508	SCCmec IVc (TCH60)	CC80	POS	POS	NEG	NEG	NEG	NEG	NEG	NEG	NEG	NEG	NEG	NEG	NEG	NEG	POS	AMB	POS	NEG	NEG	NEG	POS	NEG	POS	NEG	POS	NEG	POS	POS	POS
UKD-1010412	SCCmec IVc (TCH60)	CC80	POS	POS	AMB	NEG	NEG	NEG	NEG	NEG	NEG	NEG	NEG	NEG	AMB	NEG	AMB	AMB	AMB	NEG	POS	NEG	POS	NEG	POS	NEG	POS	NEG	POS	AMB	POS
UKD-12753005	SCCmec IVc (TCH60)	CC80	POS	POS	NEG	NEG	NEG	NEG	NEG	NEG	NEG	NEG	NEG	NEG	NEG	NEG	AMB	NEG	POS	NEG	AMB	NEG	POS	NEG	POS	NEG	AMB	NEG	POS	POS	NEG
UKD-15574809	SCCmec IVc (TCH60)	CC80	POS	POS	NEG	NEG	NEG	NEG	NEG	NEG	NEG	NEG	NEG	NEG	NEG	NEG	NEG	NEG	POS	NEG	NEG	NEG	POS	NEG	POS	NEG	POS	NEG	POS	POS	POS
KHDN-16120291	SCCmec IVc (TCH60)	CC88	POS	POS	NEG	NEG	POS	NEG	NEG	NEG	NEG	POS	NEG	NEG	NEG	POS	POS	NEG	NEG	NEG	NEG	NEG	POS	NEG	POS	NEG	POS	NEG	POS	POS	NEG
Prediction for WW2703/97, GenBank ACSW	SCCmec IVc (WW2703/97)	CC30	POS	POS	NEG	NEG	NEG	NEG	NEG	NEG	POS	NEG	NEG	NEG	POS	POS	NEG	NEG	NEG	NEG	NEG	POS	POS	POS	POS	POS	NEG	NEG	POS	AMB	POS
UKD-14580088	SCCmec IVc (WW2703/97)	CC398	POS	POS	NEG	NEG	NEG	NEG	NEG	NEG	NEG	NEG	NEG	NEG	NEG	NEG	NEG	POS	NEG	NEG	NEG	NEG	POS	NEG	POS	POS	NEG	POS	NEG	NEG	NEG
Prediction for SA_ST125_MupR, GenBank ASTH	SCCmec IVc+speG+ccrAB4 (SA_ST125)	CC5	POS	POS	NEG	NEG	NEG	POS	NEG	NEG	NEG	POS	NEG	POS	NEG	NEG	POS	NEG	NEG	NEG	NEG	POS	POS	NEG	POS	NEG	POS	NEG	AMB	POS	NEG
UKD-0439909	SCCmec IVc+speG+ccrAB4 (SA_ST125)	CC5	POS	POS	NEG	NEG	NEG	POS	AMB	NEG	NEG	POS	NEG	AMB	NEG	NEG	POS	NEG	NEG	NEG	NEG	POS	POS	NEG	POS	NEG	POS	NEG	POS	POS	NEG
Prediction for MRSA177, GenBank AECP	SCCmec IVc+Cu/Hg (MRSA177)		POS	POS	NEG	NEG	NEG	POS	NEG	NEG	NEG	POS	POS	NEG	NEG	NEG	POS	NEG	NEG	POS	NEG	NEG	POS	NEG	POS	NEG	POS	POS	NEG	NEG	NEG
UKD-1279734	SCCmec IVc+Cu/Hg (MRSA177)	CC8	POS	POS	NEG	NEG	NEG	POS	NEG	NEG	NEG	POS	POS	NEG	NEG	NEG	POS	NEG	NEG	POS	AMB	NEG	POS	NEG	POS	POS	POS	POS	NEG	NEG	POS
UKD-14580899	SCCmec IVc+Cu/Hg (MRSA177)	CC8	POS	POS	NEG	NEG	NEG	POS	NEG	NEG	NEG	POS	POS	NEG	NEG	NEG	POS	NEG	NEG	POS	AMB	NEG	POS	NEG	POS	POS	POS	POS	NEG	NEG	POS
UKD-16002228	SCCmec IVc+Cu/Hg (MRSA177)	CC8	POS	POS	NEG	NEG	NEG	POS	NEG	NEG	NEG	POS	POS	NEG	NEG	NEG	POS	NEG	NEG	POS	NEG	NEG	POS	NEG	POS	POS	POS	POS	NEG	NEG	NEG
Prediction for HO 5096 0412, GenBank HE681097.1	SCCmec IVh/j (HO50960412/JCSC6670)	CC22	POS	POS	NEG	NEG	NEG	POS	NEG	NEG	NEG	AMB	NEG	NEG	NEG	NEG	POS	NEG	NEG	NEG	NEG	POS	NEG	NEG	POS	NEG	NEG	NEG	POS	AMB	NEG
Prediction for JCSC6670, GenBank AB425824.1	SCCmec IVh/j (HO50960412/JCSC6670)																														
Reference strain Dublin_DSH_E1749	SCCmec IVh/j (HO50960412/JCSC6670)		POS	POS	NEG	NEG	NEG	POS	NEG	NEG	NEG	AMB	NEG	NEG	NEG	NEG	POS	NEG	NEG	NEG	NEG	POS	NEG	NEG	POS	NEG	NEG	NEG	POS	POS	NEG
UKD-14576248	SCCmec IVh/j (HO50960412/JCSC6670)	CC8	POS	POS	NEG	NEG	NEG	POS	NEG	NEG	NEG	POS	POS	NEG	NEG	NEG	POS	NEG	NEG	POS	NEG	NEG	POS	NEG	POS	POS	POS	POS	NEG	NEG	NEG
UKD-0120122	SCCmec IVh/j (HO50960412/JCSC6670)	CC22	POS	POS	NEG	NEG	NEG	POS	NEG	NEG	NEG	NEG	NEG	NEG	NEG	NEG	POS	NEG	NEG	NEG	NEG	POS	NEG	NEG	POS	NEG	NEG	NEG	POS	POS	NEG
UKD-0205319	SCCmec IVh/j (HO50960412/JCSC6670)	CC22	POS	POS	NEG	NEG	NEG	POS	NEG	NEG	NEG	AMB	NEG	NEG	NEG	NEG	POS	NEG	NEG	NEG	NEG	POS	NEG	NEG	POS	NEG	NEG	NEG	POS	POS	NEG
UKD-0221259	SCCmec IVh/j (HO50960412/JCSC6670)	CC22	POS	POS	NEG	NEG	NEG	POS	NEG	NEG	NEG	NEG	NEG	NEG	NEG	NEG	AMB	NEG	NEG	NEG	NEG	POS	NEG	NEG	POS	NEG	NEG	NEG	POS	POS	NEG
UKD-0418654	SCCmec IVh/j (HO50960412/JCSC6670)	CC22	POS	POS	NEG	NEG	NEG	POS	NEG	NEG	NEG	NEG	NEG	NEG	NEG	NEG	AMB	NEG	NEG	NEG	NEG	POS	NEG	NEG	POS	NEG	NEG	NEG	POS	POS	AMB
UKD-0429721	SCCmec IVh/j (HO50960412/JCSC6670)	CC22	POS	POS	NEG	NEG	NEG	POS	NEG	NEG	NEG	NEG	NEG	NEG	NEG	NEG	NEG	NEG	NEG	NEG	NEG	POS	NEG	NEG	POS	NEG	NEG	NEG	POS	POS	NEG
UKD-1116194	SCCmec IVh/j (HO50960412/JCSC6670)	CC22	POS	POS	NEG	NEG	NEG	POS	AMB	NEG	NEG	POS	NEG	NEG	NEG	NEG	POS	NEG	NEG	NEG	AMB	POS	NEG	NEG	POS	NEG	NEG	NEG	POS	POS	AMB
UKD-1122367	SCCmec IVh/j (HO50960412/JCSC6670)	CC22	POS	POS	NEG	NEG	NEG	POS	AMB	NEG	NEG	POS	NEG	NEG	NEG	NEG	POS	NEG	NEG	NEG	NEG	POS	NEG	NEG	POS	NEG	AMB	NEG	POS	POS	POS
UKD-14012840	SCCmec IVh/j (HO50960412/JCSC6670)	CC22	POS	POS	NEG	NEG	NEG	POS	NEG	NEG	NEG	POS	NEG	NEG	NEG	NEG	POS	AMB	NEG	NEG	POS	POS	NEG	NEG	POS	NEG	NEG	NEG	POS	POS	AMB
UKD-14579833	SCCmec IVh/j (HO50960412/JCSC6670)	CC22	POS	POS	NEG	NEG	NEG	POS	NEG	NEG	NEG	POS	NEG	NEG	NEG	NEG	POS	AMB	NEG	NEG	NEG	POS	NEG	NEG	POS	NEG	NEG	NEG	POS	POS	AMB
UKD-15579198	SCCmec IVh/j (HO50960412/JCSC6670)	CC22	POS	POS	NEG	NEG	NEG	POS	AMB	NEG	NEG	POS	NEG	NEG	NEG	NEG	POS	POS	NEG	NEG	POS	POS	AMB	NEG	POS	NEG	POS	NEG	POS	POS	POS
UKD-4052193	SCCmec IVh/j (HO50960412/JCSC6670)	CC22	POS	POS	NEG	NEG	NEG	POS	NEG	NEG	NEG	POS	NEG	NEG	NEG	NEG	POS	AMB	NEG	NEG	POS	POS	NEG	NEG	POS	NEG	AMB	NEG	POS	POS	POS
UKD-124968	SCCmec IVh/j (HO50960412/JCSC6670)	CC22	POS	POS	NEG	NEG	NEG	POS	NEG	NEG	NEG	POS	NEG	NEG	NEG	NEG	POS	NEG	NEG	NEG	NEG	POS	NEG	NEG	POS	NEG	AMB	NEG	POS	POS	POS
UKD-14572546	SCCmec IVh/j (HO50960412/JCSC6670)	CC22	POS	POS	NEG	NEG	NEG	POS	POS	NEG	NEG	POS	NEG	NEG	NEG	NEG	POS	POS	NEG	NEG	POS	POS	NEG	NEG	POS	NEG	AMB	NEG	POS	POS	POS
UKD-14582287	SCCmec IVh/j (HO50960412/JCSC6670)	CC22	POS	POS	NEG	NEG	NEG	POS	POS	NEG	NEG	POS	NEG	NEG	NEG	NEG	POS	POS	NEG	NEG	POS	POS	NEG	NEG	POS	NEG	POS	NEG	POS	POS	POS
UKD-14583376	SCCmec IVh/j (HO50960412/JCSC6670)	CC22	POS	POS	NEG	NEG	NEG	POS	POS	NEG	NEG	POS	NEG	NEG	NEG	NEG	POS	POS	NEG	NEG	POS	POS	NEG	NEG	POS	NEG	POS	NEG	POS	POS	POS
UKD-14583496	SCCmec IVh/j (HO50960412/JCSC6670)	CC22	POS	POS	NEG	NEG	NEG	POS	POS	NEG	NEG	POS	NEG	NEG	NEG	NEG	POS	POS	NEG	NEG	POS	POS	AMB	NEG	POS	NEG	POS	NEG	POS	POS	POS
UKD-0013324	SCCmec IVh/j (HO50960412/JCSC6670)	CC22	POS	POS	NEG	NEG	NEG	POS	NEG	NEG	NEG	NEG	NEG	NEG	NEG	NEG	POS	NEG	NEG	NEG	NEG	POS	NEG	NEG	POS	NEG	NEG	NEG	POS	POS	NEG
UKD-0125329	SCCmec IVh/j (HO50960412/JCSC6670)	CC22	POS	POS	NEG	NEG	NEG	POS	NEG	NEG	NEG	NEG	NEG	NEG	NEG	NEG	AMB	NEG	NEG	NEG	NEG	POS	NEG	NEG	POS	NEG	NEG	NEG	POS	POS	NEG
UKD-0215498	SCCmec IVh/j (HO50960412/JCSC6670)	CC22	POS	POS	NEG	NEG	NEG	POS	NEG	NEG	NEG	NEG	NEG	NEG	NEG	NEG	NEG	NEG	NEG	NEG	NEG	POS	NEG	NEG	POS	NEG	NEG	NEG	POS	POS	NEG
Prediction for M08-0126, GenBank FR753166.1	SCCmec IVh/j+ACME2 (M08-0126)	CC22																													
UKD-1379483I	SCCmec IVh/j+ACME2 (M08-0126)	CC22	POS	POS	AMB	NEG	NEG	POS	NEG	NEG	NEG	POS	NEG	NEG	NEG	NEG	POS	POS	NEG	NEG	POS	POS	NEG	NEG	POS	NEG	POS	NEG	POS	POS	POS
UKD-5150204	SCCmec IV+speG+Cu/czrC+ccrAB4	CC22	POS	POS	NEG	NEG	NEG	POS	POS	NEG	NEG	POS	NEG	NEG	NEG	NEG	POS	NEG	NEG	NEG	POS	POS	NEG	NEG	POS	NEG	NEG	NEG	POS	POS	AMB
UKD-15578423	SCCmec IV+speG+czrC+ccrAB4 var. 1	CC22	POS	POS	NEG	NEG	NEG	POS	NEG	NEG	NEG	POS	NEG	NEG	NEG	NEG	POS	AMB	NEG	NEG	NEG	POS	NEG	NEG	POS	NEG	POS	NEG	POS	POS	POS
UKD-16582870	SCCmec IV+speG+czrC+ccrAB4 var. 1	CC22	POS	POS	NEG	NEG	NEG	POS	NEG	NEG	NEG	NEG	NEG	NEG	NEG	NEG	POS	NEG	NEG	NEG	NEG	POS	NEG	NEG	POS	NEG	NEG	NEG	POS	POS	NEG
UKD-15571940	SCCmec IV+speG+czrC+ccrAB4 var. 2	CC22	POS	POS	NEG	NEG	NEG	POS	NEG	NEG	NEG	POS	NEG	NEG	NEG	NEG	POS	AMB	NEG	NEG	NEG	POS	AMB	NEG	POS	NEG	POS	NEG	POS	POS	POS

Isolate ID	SCCmec type	Clonal complex	PUTATIVE TRANSPORTER				TYPE I RESTRICTION-MODIFICATION SYSTEM, SINGLE SEQUENCE SPECIFICITY PROTEIN													MISCELLANEOUS GENES				HYALURONATE LYASES							
			hypothetical protein, similar to integral membrane protein LmrP				type I site-specific deoxyribonuclease subunit, 1st locus	type I site-specific deoxyribonuclease subunit, 2nd locus				type I site-specific deoxyribonuclease subunit, 3rd locus					type I site-specific deoxyribonuclease subunit, unknown locus			ear2 = Q2FXC0	Q2YUB3	Q7A4X2	sdrM / tetEfflux	hysA1 (MRSA252)	hysA1 (MRSA252+RF122) and/or hysA2 (cons)	hysA1 (MRSA252+RF122) and/or hysA2 (COL+USA300)	hysA2 (All Other Than MRSA252)	hysA2 (COL+USA300+NCTC)	hysA2 (All Other Than COL+USA300+NCTC)	hysA2-AllOtherThan COL+USA300+NCTC	hysA2 (MRSA252)
			lmrP (OtherThanRF122)	lmrP (OtherThanRF122)	lmrP (RF122)	lmrP (RF122)	hsdS1-RF122	hsdS2-ST5+ST8	hsdS2-MW2+476	hsdS2-RF122	hsdS2-MRSA252	hsdS3-AllOtherThanRF122+252	hsdS3-ST8+ST1+RF122	hsdS3-Mu50+N315	hsdS3-CC51+252	hsdS3-MRSA252	hsdSx-CC25	hsdSx-CC15	hsdSx-etd												
Prediction for CN1, GenBank CP003979.1	SCCmec IVA (CN1)	CC8 (ST72)	POS	POS	NEG	NEG	NEG	NEG	NEG	NEG	NEG	NEG	NEG	NEG	POS	NEG	POS	NEG	NEG	NEG	NEG	POS	POS	NEG	POS	NEG	POS	NEG	POS	AMB	NEG
UKD-0900871	SCCmec IVA (CN1)	CC8 (ST72)	POS	POS	NEG	NEG	NEG	NEG	NEG	NEG	NEG	AMB	NEG	NEG	POS	NEG	POS	NEG	NEG	NEG	NEG	POS	POS	NEG	POS	NEG	POS	NEG	POS	POS	NEG
Prediction for CMFT1723, GenBank HF569096.1	SCCmec V (Bengal Bay)																														
KHDN-10405134	SCCmec V (Bengal Bay)	CC1	POS	POS	NEG	NEG	NEG	NEG	POS	NEG	NEG	POS	POS	NEG	NEG	NEG	POS	NEG	NEG	POS	NEG	NEG	POS	NEG	POS	NEG	POS	NEG	POS	POS	NEG
UKD-0932405	SCCmec V (Bengal Bay)	CC1 (ST573/772)	POS	POS	NEG	NEG	NEG	NEG	NEG	NEG	NEG	NEG	NEG	NEG	NEG	NEG	NEG	NEG	NEG	NEG	NEG	POS	POS	NEG	POS	NEG	POS	NEG	POS	POS	AMB
UKD-1003510	SCCmec V (Bengal Bay)	CC1 (ST573/772)	POS	POS	NEG	NEG	NEG	NEG	NEG	NEG	NEG	AMB	NEG	NEG	NEG	NEG	AMB	NEG	NEG	NEG	AMB	POS	POS	NEG	POS	NEG	POS	NEG	POS	POS	POS
UKD-1112420	SCCmec V (Bengal Bay)	CC1 (ST573/772)	POS	POS	NEG	NEG	NEG	NEG	NEG	NEG	NEG	POS	NEG	NEG	NEG	NEG	AMB	NEG	NEG	NEG	AMB	POS	POS	NEG	POS	NEG	POS	NEG	POS	POS	NEG
UKD-1275832	SCCmec V (Bengal Bay)	CC1 (ST573/772)	POS	POS	NEG	NEG	NEG	NEG	NEG	NEG	NEG	POS	NEG	NEG	NEG	NEG	POS	AMB	NEG	NEG	AMB	POS	POS	NEG	POS	NEG	POS	NEG	POS	POS	NEG
UKD-13023811	SCCmec V (Bengal Bay)	CC1 (ST573/772)	POS	POS	NEG	NEG	NEG	NEG	NEG	NEG	NEG	NEG	NEG	NEG	NEG	NEG	NEG	POS	NEG	NEG	NEG	POS	POS	NEG	POS	NEG	POS	NEG	POS	POS	NEG
UKD-14572163	SCCmec V	CC5	POS	POS	NEG	NEG	NEG	POS	NEG	NEG	NEG	POS	NEG	POS	NEG	NEG	POS	NEG	NEG	NEG	NEG	POS	POS	NEG	POS	NEG	POS	NEG	POS	POS	NEG
KHDN-10421133	SCCmec V+fus	CC97	POS	POS	NEG	NEG	NEG	NEG	NEG	NEG	NEG	POS	NEG	NEG	NEG	NEG	POS	NEG	NEG	NEG	NEG	POS	POS	NEG	POS	NEG	AMB	NEG	POS	POS	NEG
KHDN-21343541	SCCmec V+fus+tir+ccrAB1	CC1	POS	POS	NEG	NEG	NEG	NEG	POS	NEG	NEG	POS	POS	NEG	NEG	NEG	POS	AMB	NEG	POS	NEG	NEG	POS	NEG	POS	NEG	POS	NEG	POS	POS	NEG
KHDN-10416893	SCCmec V+czrC+speG+ccrAB4	CC22	POS	POS	NEG	NEG	NEG	POS	NEG	NEG	NEG	POS	NEG	NEG	NEG	NEG	POS	NEG	NEG	NEG	NEG	POS	NEG	NEG	POS	NEG	NEG	NEG	POS	POS	NEG
Prediction for GR1, GenBank AJLX	SCCmec VT (GR1)	CC361	POS	POS	NEG	NEG	NEG	NEG	POS	NEG	NEG	NEG	NEG	NEG	POS	NEG	NEG	NEG	NEG	NEG	NEG	POS	POS	NEG	POS	NEG	POS	POS	NEG	NEG	NEG
UKD-0422872	SCCmec VT (GR1)	CC152	NEG	NEG	POS	POS	NEG	POS	AMB	NEG	NEG	POS	NEG	NEG	POS	POS	POS	AMB	POS	NEG	POS	NEG	POS	NEG	POS	POS	NEG	POS	NEG	NEG	POS
Prediction for PM1, GenBank BAFA	SCCmec VT (PM1)	CC59	NEG	NEG	POS	POS	NEG	NEG	NEG	NEG	NEG	NEG	NEG	NEG	POS	NEG	NEG	NEG	POS	NEG	NEG	NEG	POS	NEG	POS	NEG	POS	NEG	POS	AMB	NEG
KHDN-000871	SCCmec VT (PM1)	CC45 (agr I)	POS	POS	NEG	NEG	NEG	NEG	AMB	NEG	POS	NEG	NEG	NEG	NEG	NEG	POS	AMB	NEG	NEG	NEG	POS	POS	NEG	POS	NEG	AMB	NEG	POS	NEG	NEG
UKD-0725052	SCCmec VT (PM1)	CC59	NEG	NEG	POS	POS	NEG	NEG	NEG	NEG	NEG	NEG	NEG	NEG	POS	NEG	NEG	NEG	POS	NEG	NEG	NEG	POS	NEG	POS	NEG	NEG	NEG	POS	POS	NEG
UKD-1312138	SCCmec VT (PM1)	CC59	NEG	NEG	POS	POS	NEG	NEG	POS	NEG	NEG	POS	NEG	NEG	POS	NEG	NEG	POS	POS	NEG	POS	NEG	POS	NEG	POS	NEG	POS	NEG	POS	POS	NEG
UKD-Z4728	SCCmec VT (PM1)	CC59	NEG	NEG	POS	POS	NEG	NEG	NEG	NEG	NEG	AMB	NEG	NEG	POS	NEG	NEG	NEG	POS	NEG	NEG	NEG	POS	NEG	POS	NEG	POS	NEG	POS	POS	NEG
UKD-K0199	SCCmec VT unknown var.1	CC7	POS	POS	NEG	NEG	NEG	NEG	NEG	NEG	NEG	NEG	NEG	NEG	NEG	NEG	AMB	NEG	POS	NEG	NEG	POS	POS	NEG	POS	NEG	NEG	NEG	POS	POS	NEG
UKD-15584619	SCCmec VT unknown var.2	CC30	POS	POS	NEG	NEG	NEG	NEG	POS	NEG	POS	AMB	NEG	NEG	POS	POS	POS	POS	NEG	NEG	NEG	POS	POS	POS	POS	POS	NEG	POS	POS	POS	POS
UKD-0712568	SCCmec VT unknown var.3	CC45 (agr I)	POS	POS	NEG	NEG	NEG	NEG	NEG	NEG	POS	NEG	NEG	NEG	NEG	NEG	AMB	NEG	NEG	NEG	NEG	POS	AMB	NEG	POS	NEG	NEG	NEG	POS	NEG	NEG
KHDN-10420296	SCCmec VT+ACME2	CC45 (agr I)	POS	POS	NEG	NEG	NEG	NEG	POS	NEG	POS	NEG	NEG	NEG	NEG	NEG	POS	AMB	NEG	NEG	AMB	POS	POS	NEG	POS	NEG	POS	NEG	POS	NEG	NEG
UKD-15577956	SCCmec VT+Cu/czrC+ccrA1	CC398	POS	POS	NEG	NEG	NEG	NEG	NEG	NEG	NEG	NEG	NEG	NEG	NEG	NEG	POS	NEG	NEG	NEG	NEG	NEG	POS	NEG	POS	POS	NEG	POS	NEG	NEG	POS
Prediction for S0385, GenBank AM990992	SCCmec VT+czrC (SO385)	CC398	POS	POS	NEG	NEG	NEG	NEG	NEG	NEG	NEG	NEG	NEG	NEG	NEG	NEG	NEG	POS	NEG	NEG	NEG	NEG	POS	NEG	POS	AMB	NEG	POS	NEG	NEG	POS
UKD-0628560	SCCmec VT+czrC (SO385)	CC8	POS	POS	NEG	NEG	NEG	POS	NEG	NEG	NEG	POS	POS	NEG	NEG	NEG	POS	NEG	NEG	POS	NEG	NEG	POS	NEG	POS	POS	AMB	POS	NEG	NEG	NEG
UKD-0510018	SCCmec VT+czrC (SO385)	CC398	POS	AMB	NEG	NEG	NEG	NEG	NEG	NEG	NEG	NEG	NEG	NEG	NEG	NEG	NEG	POS	NEG	NEG	NEG	NEG	POS	NEG	POS	POS	NEG	POS	NEG	NEG	NEG
UKD-0903558	SCCmec VT+czrC (SO385)	CC398	POS	POS	NEG	NEG	NEG	NEG	NEG	NEG	NEG	NEG	NEG	NEG	NEG	NEG	NEG	POS	NEG	NEG	NEG	NEG	POS	NEG	POS	POS	NEG	POS	NEG	NEG	NEG
UKD-0929815	SCCmec VT+czrC (SO385)	CC398	POS	POS	NEG	NEG	NEG	NEG	NEG	NEG	NEG	NEG	NEG	NEG	NEG	NEG	NEG	POS	NEG	NEG	NEG	NEG	POS	NEG	POS	POS	NEG	POS	NEG	NEG	NEG
UKD-0907762	SCCmec VT+czrC (SO385)	CC398	POS	POS	NEG	NEG	NEG	NEG	NEG	NEG	NEG	NEG	NEG	NEG	NEG	NEG	NEG	POS	NEG	NEG	NEG	NEG	POS	NEG	POS	POS	NEG	POS	NEG	NEG	NEG
UKD-1025105	SCCmec VT+czrC (SO385)	CC398	POS	POS	NEG	NEG	NEG	NEG	NEG	NEG	NEG	NEG	NEG	NEG	NEG	NEG	NEG	POS	NEG	NEG	NEG	NEG	POS	NEG	POS	POS	NEG	POS	NEG	NEG	NEG
UKD-11780898	SCCmec VT+czrC (SO385)	CC398	POS	POS	NEG	NEG	NEG	NEG	POS	NEG	NEG	NEG	NEG	NEG	NEG	NEG	POS	POS	NEG	NEG	AMB	NEG	POS	NEG	POS	POS	NEG	POS	NEG	NEG	POS
UKD-1377009	SCCmec VT+czrC (SO385)	CC398	POS	POS	NEG	NEG	NEG	NEG	NEG	NEG	NEG	NEG	NEG	NEG	NEG	NEG	NEG	POS	NEG	NEG	NEG	NEG	POS	NEG	POS	POS	NEG	POS	NEG	NEG	AMB
UKD-143406	SCCmec VT+czrC (SO385)	CC398	POS	POS	NEG	NEG	NEG	NEG	POS	NEG	NEG	AMB	NEG	NEG	POS	NEG	POS	POS	NEG	NEG	POS	NEG	POS	NEG	POS	POS	NEG	POS	NEG	POS	POS
UKD-1400363	SCCmec VT+czrC (SO385)	CC398	POS	POS	NEG	NEG	NEG	NEG	AMB	NEG	NEG	NEG	NEG	NEG	NEG	NEG	NEG	POS	NEG	NEG	NEG	NEG	POS	NEG	POS	POS	NEG	POS	NEG	NEG	NEG
UKD-14571852	SCCmec VT+czrC (SO385)	CC398	POS	POS	NEG	NEG	NEG	NEG	POS	NEG	NEG	NEG	NEG	NEG	AMB	NEG	POS	POS	NEG	NEG	POS	NEG	POS	NEG	POS	POS	NEG	POS	NEG	POS	POS
UKD-14582574	SCCmec VT+czrC (SO385)	CC398	POS	POS	NEG	NEG	NEG	NEG	NEG	NEG	NEG	NEG	NEG	NEG	NEG	NEG	NEG	POS	NEG	NEG	NEG	NEG	POS	NEG	POS	POS	NEG	POS	NEG	NEG	NEG
UKD-152108	SCCmec VT+czrC (SO385)	CC398	POS	POS	NEG	NEG	NEG	NEG	NEG	NEG	NEG	NEG	NEG	NEG	NEG	NEG	NEG	POS	NEG	NEG	NEG	NEG	POS	NEG	POS	POS	NEG	POS	NEG	NEG	NEG
UKD-15573321	SCCmec VT+czrC (SO385)	CC398	POS	POS	NEG	NEG	NEG	NEG	AMB	NEG	NEG	NEG	NEG	NEG	NEG	NEG	POS	POS	NEG	NEG	NEG	NEG	POS	NEG	POS	POS	NEG	POS	NEG	NEG	NEG
UKD-15584034	SCCmec VT+czrC , ydhK-neg. variant	CC398	POS	POS	NEG	NEG	NEG	NEG	NEG	NEG	NEG	NEG	NEG	NEG	NEG	NEG	POS	POS	NEG	NEG	NEG	NEG	POS	NEG	POS	POS	NEG	POS	NEG	NEG	POS
UKD-0932581	SCCmecVT+fus+czrC	CC22	POS	POS	NEG	NEG	NEG	POS	NEG	NEG	NEG	POS	NEG	NEG	NEG	NEG	POS	NEG	NEG	NEG	NEG	POS	NEG	NEG	POS	NEG	NEG	NEG	POS	POS	NEG
UKD-1121994	SCCmecVT+fus+czrC	CC22	POS	POS	NEG	NEG	NEG	POS	AMB	NEG	NEG	POS	NEG	NEG	NEG	NEG	POS	NEG	NEG	NEG	NEG	POS	NEG	NEG	POS	NEG	NEG	NEG	POS	POS	POS
UKD-1272252	SCCmecVT+fus+czrC	CC22	POS	POS	NEG	NEG	NEG	POS	NEG	NEG	NEG	POS	NEG	NEG	NEG	NEG	POS	NEG	NEG	NEG	AMB	POS	NEG	NEG	POS	NEG	NEG	NEG	POS	POS	AMB
Prediction for MRSA18	SCCmec VI+fus (MRSA18)																														
UKD-1103380	SCCmec VI+fus (MRSA18)	CC7	POS	POS	NEG	NEG	NEG	NEG	NEG	NEG	NEG	NEG	NEG	NEG	NEG	NEG	NEG	NEG	POS	NEG	NEG	POS	POS	NEG	POS	NEG	NEG	NEG	POS	POS	NEG
Prediction for M10/0061, GenBank FR823292.1	SCCmec XI (LGA251/M10-61)	CC130																													
Reference strain Dublin-DSH_M10 0061	SCCmec XI (LGA251/M10-61)	CC130	NEG	NEG	POS	POS	NEG	NEG	NEG	POS	NEG	AMB	POS	NEG	NEG	NEG	POS	AMB	POS	NEG	AMB	NEG	POS	NEG	POS	NEG	NEG	NEG	POS	POS	NEG
Prediction for LGA251, GenBank FR821779.1	SCCmec XI (LGA251/M10-61)	ST425																													
UKD-14015835	SCCmec XI (LGA251/M10-61)	CC130	NEG	NEG	POS	POS	NEG	NEG	NEG	POS	NEG	AMB	POS	NEG	NEG	NEG	POS	NEG	POS	NEG	NEG	NEG	POS	NEG	NEG	NEG	NEG	NEG	POS	POS	NEG
UKD-16573252	SCCmec XI (LGA251/M10-61)	CC130	NEG	AMB	POS	POS	NEG	NEG	AMB	POS	NEG	POS	POS	NEG	NEG	NEG	POS	NEG	POS	NEG	NEG	NEG	POS	NEG	NEG	NEG	NEG	NEG	POS	POS	NEG
UKD-0217002	PseudoSCCmec, class B mec complex+Hg	CC8	POS	POS	NEG	NEG	NEG	POS	NEG	NEG	NEG	POS	POS	NEG	NEG	NEG	POS	NEG	NEG	POS	NEG	NEG	POS	NEG	POS	POS	POS	POS	NEG	NEG	NEG
UKD-0008510	PseudoSCCmec, class B mec complex+Hg	CC8	POS	POS	NEG	NEG	NEG	POS	NEG	NEG	NEG	POS	POS	NEG	NEG	NEG	POS	NEG	NEG	POS	NEG	NEG	POS	NEG	POS	POS	POS	POS	NEG	NEG	NEG
UKD-0110962	PseudoSCCmec, class B mec complex+Hg	CC8	POS	POS	NEG	NEG	NEG	POS	NEG	NEG	NEG	POS	POS	NEG	NEG	NEG	POS	NEG	NEG	POS	NEG	NEG	POS	NEG	POS	POS	POS	POS	NEG	NEG	NEG
KHDN-10408593	PseudoSCCmec, class B mec complex+Cu/Hg	CC8	POS	POS	NEG	NEG	NEG	POS	NEG	NEG	NEG	POS	POS	NEG	NEG	NEG	POS	NEG	NEG	POS	NEG	NEG	POS	NEG	POS	POS	POS	POS	NEG	NEG	NEG
UKD-0007201	PseudoSCCmec, class B mec complex	CC5	POS	POS	NEG	NEG	NEG	NEG	NEG	NEG	NEG	POS	NEG	POS	NEG	NEG	POS	NEG	NEG	NEG	NEG	POS	POS	NEG	POS	NEG	POS	NEG	POS	POS	NEG
UKD-0033204	PseudoSCCmec, class B mec complex	CC5	POS	POS	NEG	NEG	NEG	NEG	NEG	NEG	NEG	POS	NEG	POS	NEG	NEG	POS	NEG	NEG	NEG	NEG	POS	POS	NEG	POS	NEG	POS	NEG	POS	POS	NEG
UKD-0134329	PseudoSCCmec, class B mec complex	CC5	POS	POS	NEG	NEG	NEG	NEG	NEG	NEG	NEG	AMB	NEG	POS	NEG	NEG	POS	NEG	NEG	NEG	NEG	POS	AMB	NEG	AMB	NEG	NEG	NEG	AMB	POS	NEG
UKD-0806946	PseudoSCCmec class C+As/Cu	CC398	POS	POS	NEG	NEG	NEG	NEG	NEG	NEG	NEG	NEG	NEG	NEG	NEG	NEG	NEG	POS	NEG	NEG	NEG	NEG	POS	NEG	POS	POS	NEG	POS	NEG	NEG	NEG
UKD-0500610	ACME2	CC8	POS	POS	NEG	NEG	NEG	POS	NEG	NEG	NEG	POS	POS	NEG	NEG	NEG	POS	NEG	POS	POS	NEG	NEG	POS	NEG	POS	POS	POS	POS	NEG	NEG	NEG
UKD-0610438	ACME2	CC8	POS	POS	NEG	NEG	NEG	POS	NEG	NEG	NEG	POS	POS	NEG	NEG	NEG	POS	NEG	NEG	POS	POS	NEG	POS	NEG	POS	POS	POS	POS	NEG	NEG	NEG
KHDN-10409344	ACME2	CC8	POS	POS	NEG	NEG	NEG	POS	NEG	NEG	NEG	POS	POS	NEG	NEG	NEG	POS	NEG	NEG	POS	AMB	NEG	POS	NEG	POS	POS	POS	POS	NEG	NEG	NEG
UKD-0322559	ACME2+speG+czrC+ccrAB4	CC8	POS	POS	NEG	NEG	NEG	POS	NEG	NEG	NEG	POS	POS	NEG	NEG	NEG	POS	NEG	NEG	POS	NEG	NEG	POS	NEG	POS	POS	POS	POS	NEG	NEG	NEG
UKD-0501998	ACME2+speG+czrC+ccrAB4	CC8	POS	POS	NEG	NEG	NEG	POS	NEG	NEG	NEG	POS	POS	NEG	NEG	NEG	POS	NEG	NEG	POS	NEG	NEG	POS	NEG	POS	POS	AMB	POS	NEG	NEG	NEG
UKD-0505213	ACME2+speG+czrC+ccrAB4	CC8	POS	POS	NEG	NEG	NEG	POS	NEG	NEG	NEG	POS	POS	NEG	NEG	NEG	POS	NEG	NEG	POS	NEG	NEG	POS	NEG	POS	POS	POS	POS	NEG	NEG	NEG
Prediction for MSSA476, GenBank BX571857.1	fus+tir+ccrAB1 (MSSA476)	CC1	POS	POS	NEG	NEG	NEG	NEG	POS	NEG	NEG	POS	POS	NEG	NEG	NEG	POS	NEG	NEG	POS	NEG	NEG	POS	NEG	POS	NEG	POS	NEG	POS	AMB	NEG
Reference strain Sanger 476	fus+tir+ccrAB1 (MSSA476)	CC1	POS	POS	NEG	NEG	NEG	NEG	POS	NEG	NEG	POS	POS	NEG	NEG	NEG	POS	NEG	NEG	POS	NEG	NEG	POS	NEG	POS	NEG	POS	NEG	POS	POS	NEG
UKD-14023914	fus+tir+ccrAB1 (MSSA476)	CC1	POS	POS	NEG	NEG	NEG	NEG	POS	NEG	NEG	POS	POS	NEG	NEG	NEG	POS	NEG	NEG	POS	NEG	NEG	POS	NEG	POS	NEG	POS	NEG	POS	POS	NEG
UKD-K00034	kdp+ccrAB2	CC182	NEG	NEG	POS	POS	NEG	NEG	AMB	NEG	NEG	NEG	NEG	NEG	NEG	NEG	NEG	NEG	NEG	NEG	NEG	NEG	POS	NEG	POS	NEG	NEG	NEG	POS	POS	NEG
UKD-15580369	As+ccrAB4	CC22	POS	POS	AMB	NEG	NEG	POS	NEG	NEG	NEG	POS	NEG	NEG	NEG	NEG	POS	AMB	NEG	NEG	POS	POS	NEG	NEG	POS	NEG	POS	NEG	POS	POS	POS
UKD-15570100	ACME3+ccrAB1 (Strain 21342)	CC80	POS	POS	NEG	NEG	NEG	NEG	AMB	NEG	NEG	NEG	NEG	NEG	NEG	NEG	NEG	AMB	NEG	NEG	AMB	NEG	POS	NEG	POS	NEG	POS	NEG	POS	POS	NEG
UKD-1010678	speG+czrC+ccrAB4	CC8	POS	POS	NEG	NEG	NEG	POS	NEG	NEG	NEG	POS	POS	NEG	NEG	NEG	POS	NEG	NEG	POS	NEG	NEG	POS	NEG	POS	POS	POS	POS	NEG	NEG	NEG
UKD-1008243	speG+czrC+ccrAB4	CC8	POS	POS	NEG	NEG	NEG	POS	NEG	NEG	NEG	POS	POS	NEG	NEG	NEG	POS	NEG	NEG	POS	NEG	NEG	POS	NEG	POS	POS	POS	POS	NEG	NEG	NEG
UKD-1009430	speG+czrC+ccrAB4	CC8	POS	POS	NEG	NEG	NEG	POS	NEG	NEG	NEG	POS	POS	NEG	NEG	NEG	POS	NEG	NEG	POS	NEG	NEG	POS	NEG	POS	POS	POS	POS	NEG	NEG	NEG
UKD-12071828	speG+czrC+ccrAB4	CC22	POS	POS	NEG	NEG	NEG	POS	NEG	NEG	NEG	AMB	NEG	NEG	NEG	NEG	POS	AMB	NEG	NEG	AMB	POS	NEG	NEG	POS	NEG	NEG	NEG	POS	POS	AMB
UKD-14011883	speG+czrC+ccrAB4	CC22	POS	POS	NEG	NEG	NEG	POS	NEG	NEG	NEG	POS	NEG	NEG	NEG	NEG	POS	AMB	NEG	NEG	POS	POS	NEG	NEG	POS	NEG	NEG	NEG	POS	POS	AMB
UKD-15008293	speG+czrC+ccrAB4	CC22	POS	POS	NEG	NEG	NEG	POS	NEG	NEG	NEG	POS	NEG	NEG	NEG	NEG	POS	NEG	NEG	NEG	NEG	POS	NEG	NEG	POS	NEG	NEG	NEG	POS	POS	AMB
UKD-1514877	speG+czrC+ccrAB4	CC22	POS	POS	NEG	NEG	NEG	POS	NEG	NEG	NEG	POS	NEG	NEG	NEG	NEG	POS	POS	NEG	NEG	NEG	POS	NEG	NEG	POS	NEG	NEG	NEG	POS	POS	AMB
UKD-15580971	speG+czrC+ccrAB4	CC22	POS	POS	NEG	NEG	NEG	POS	NEG	NEG	NEG	POS	NEG	NEG	NEG	NEG	POS	AMB	NEG	NEG	AMB	POS	NEG	NEG	POS	NEG	AMB	NEG	POS	POS	AMB
UKD-15582646	speG+czrC+ccrAB4	CC22	POS	POS	NEG	NEG	NEG	POS	NEG	NEG	NEG	POS	NEG	NEG	NEG	NEG	POS	AMB	NEG	NEG	NEG	POS	NEG	NEG	POS	NEG	NEG	NEG	POS	POS	AMB
