# Supplementary material for: Diversity of SCCmec Elements in Staphylococcus aureus as Observed in South-Eastern Germany
Source: PLoS One. 2016 Sep 20;11(9):e0162654. doi: 10.1371/journal.pone.0162654 (PMC5029946; doi:10.1371/journal.pone.0162654)
Supplement: S2 Table — (PDF) [file pone.0162654.s002.pdf]

Gene/Marker	Gene product/Function	Comments	Reference sequence	Probe name	Probe coordinates	Primer name	Primer coordinates
***adhC*- FPR3757**	Alcohol dehydrogenase, zinc-containing	Part of ACME 1 and ACME 3 clusters, that occurs alone or in combination with SCC*mec* elements. Consensus marker. Allele from the "USA300" CA-MRSA strain-FPR3757, GenBank CP000255.1 and TCH1516, GenBank CP000730.1	CP000255.1, 64125...65222	hp1245_adhC<br>hp1498_adhC	**CP000255.1 [64223:64246]**<br>**CP000255.1 [65064:65089]**	lb2337|adhC<br>lb2563|adhC	**CP000255.1 [65133:65154:r]**<br>**CP000255.1 [64251:64273:r]**
***arcA*- SCC**	Arginine deiminase	Part of ACME 1 and ACME 2 clusters, that occurs alone or in combination with SCC*mec* elements. Common in Coagulase-negatives, present in the "USA300" CA-MRSA strain-FPR3757, GenBank CP000255.1 and TCH1516, GenBank CP000730.1	CP000255.1, 73113...74348	hp_arcA_611	**CP000255.1 [73382:73407:r]**	lb_arcA_651_rv	**CP000255.1 [73337:73356]**
***arcB*- SCC**	Ornithine carbamoyltransferase		CP000255.1, 69839...70837	hp_arcB_611	**CP000255.1 [70158:70184:r]**	lb_arcB_651_rv	**CP000255.1 [70133:70151]**
***arcC*- SCC**	Carbamate kinase		CP000255.1, 68890...69819	hp_arcC_611	**CP000255.1 [69480:69508:r]**	lb_arcC_651_rv	**CP000255.1 [69448:69467]**
***arcD*- SCC**	Arginine/ornithine antiporter		CP000255.1, 71606...73027	hp_arcD_611	**CP000255.1 [72289:72317:r]**	lb_arcD_651_rv	**CP000255.1 [72258:72275]**
***arsB*- SCC**	Arsenical pump membrane protein	Three different probes were designed for SCC-born alleles of that gene, as for instance in JCSC6943, JCSC6945, M10/0061	FR823292.1, 28161...29450<br>AB505628.1, 31886...33175<br>AB705453.1[2380:3669:r]	hp1526_arsB<br>hp1478_arsB<br>hp1536_arsB	**FR823292.1**<br>**AB505628.1 [32855:32882:r]**<br>**AB705453.1[2958:2985:r]**	lb2575|arsB<br>lb2564|arsB<br>lb2565|arsB	**FR823292.1 [29284:29304]**<br>**AB505628.1 [32810:32830]**<br>**AB705453.1 [2908:2928]**
***arsC*- SCC**	Arsenate reductase	Probe was designed for SCC-born alleles of that gene, as for instance in JCSC6943, JCSC6945	AB505628.1, 31467...31868	hp1546_arsC	**AB505628.1 [38309:38333:r]**	lb2591|arsC	**AB505628.1 [38250:38270]**
**B2Y834**	Abortive phage resistance protein	Subtyping SCC*mec* IV, *i.e*., identification of SCC*mec* IV A, G, c and SCC*mec* MRSAZH47	AE015929.1 50641...51441	hp1206_B2Y834	**AE015929.1 [51095:51119]**	lb2301|B2Y834	**AE015929.1 [51124:51144:r]**
**B6VQU0**	Putative protein	Subtyping SCC*mec* IV, *i.e*., identification of SCC*mec* IVh/j	AB425824.1 19950...20882	hp1207_B6VQU0	**AB425824.1 [20412:20443]**	lb2302|B6VQU0	**AB425824.1 [20498:20525:r]**
***blaZ*- SCC*mec* XI**	Beta-lactamase	Solely present in SCC*mec* XI; this is a different *blaZ* allele than in ubiquitous staphylococcal plasmids	FR823292.1, 796...1647	hp_01_blaZ_M10	**FR823292.1 [1453:1479:r]**	lp_01_blaZ_M10	**FR823292.1 [1429:1448]**
**C5QAP8-SCC*mec* XI**	Putative protein	Identification of SCC*mec* XI	FR821779.1 53134...53913	hp1212_C5QAP8	**FR821779.1 [53676:53704]**	lb2307|C5QAP8	**FR821779.1 [53752:53772:r]**
***cadD*- R35**	Cadmium transport protein D	Probe was designed for SCC-born allele(s of that gene, as for instance in strain R35, GenBank L10909.1 or strain 85/2082, GenBank AB037671.1	L10909.1, 5577...6194	hp1461_cadD<br>hp1463_cadD	**L10909.1 [5911:5937]**<br>**L10909.1 [6038:6062]**	lb2515|cadD<br>lb2449|cadD	**L10909.1 [5963:5979:r]**<br>**L10909.1 [6088:6108:r]**
***cadX*- JCSC6943**	Putative regulator of cadmium efflux	Probe was designed for SCC-born alleles of that gene, as for instance in JCSC6943, GenBank AB505628.1	AB505628.1, 31106...31447	hp1334_cadX	**AB505628.1 [31136:31165]**	lb2423|cadX	**AB505628.1 [31203:31229:r]**
cap 1	Locus encoding SCC associated capsule type 1	Individual probes for probes for *cap* H1, J1, K1	U10927.2	hp_capH1_611<br>hp_capJ1_611<br>hp_capK1_611	**U10927.2 [19165:19192]**<br>**U10927.2 [21322:21350]**<br>**U10927.2 [22439:22466]**	lb_capH1_651_rv<br>lb_capJ1_651_rv<br>lb_capK1_651_rv	**U10927.2 [19210:19230:r]**<br>**U10927.2 [21367:21385:r]**<br>**U10927.2 [22490:22508:r]**
***cas1*- M06-0171**	CRISPR-associated endonuclease 1	Present in M06/0171, GenBank HE980450.1	HE980450.1, 48518...49423	hp1256_cas1v2<br>hp1257_cas1v2	**HE980450.1 [48631:48661]**<br>**HE980450.1 [49165:49194]**	lb2348|cas1v2	**HE980450.1 [49220:49240:r]**
ccrA-1	Cassette chromosome recombinase A, type 1	Cassette chromosome recombinase A allele found in SCC*mec* I, IX, X, SCC*fus* (as in MSSA476, GenBank BX571857.1) and in composite SCC*mec/fus* elements	CP000046.1, 47998...49347	hp_ccrA-1_611	**CP000046.1 [48646:48672:r]**	lb_ccrA-1_651_rv<br>lb_ccrA-1_652_rv	**CP000046.1 [48623:48642]**<br>**CP000046.1 [48372:48390]**
ccrA-2	Cassette chromosome recombinase A, type 2	Cassette chromosome recombinase A allele found in SCC*mec* II and IV elements	BA000033.2, 48017...49366	hp_ccrA-2_611	**BA000033.2 [49323:49351:r]**	lb_ccrA-2_651_rv<br>lb_ccrA-2_652_rv<br>lb_ccrA-2_653_rv	**BA000017.4 [66290:66306]**<br>**BA000017.4 [65738:65756]**<br>**AB063173.1 [6486:6506:r]**
ccrA-3	Cassette chromosome recombinase A, type 3	Cassette chromosome recombinase A allele found in SCC*mec* III elements	AB037671.1, 5430...6776	hp_ccrA-3_611	**AB037671.1 [5550:5575]**	lb_ccrA-3_651_rv<br>lb_ccrA-3_652_rv	**AB014436.1 [283:300:r]**<br>**AB014436.1 [826:844:r]**
ccrA-4	Cassette chromosome recombinase A, type 4	Cassette chromosome recombinase A allele found in SCC*mec* VI and SCC*mec* VIII elements	AF411935.3, 7849...9210	hp_ccrA-4_612	**AF411935.3 [8779:8805]**	lb_ccrA-4_651_rv<br>lb_ccrA-4_652_rv	**AF411935.1 [8582:8602:r]**<br>**AF411935.1 [8803:8823:r]**
ccrAA	"Cassette chromosome recombinase AA"	Gene for a hypothetical protein accompanying the *ccrC* gene in SCC*mec* V and SCC*mec* VT elements. Two separate probes were used that usually, but not always, yield identical results	AB121219.1, 14264...15907,<br>AM292304.1, 5654...7273	hp_ccrAA_612<br>hp_ccrAA_613	**AP006716.1 [63246:63271]**<br>**AP006716.1 [63636:63661]**	lb_ccrAA_652_rv<br>lb_ccrAA_651_rv<br>lb_ccrAA_653_rv	**AB037671.1 [61397:61418]**<br>**AM292304.1 [6629:6648:r]**<br>**AM292304.1 [7020:7038:r]**

Gene/Marker	Gene product/Function	Comments	Reference sequence	Probe name	Probe coordinates	Primer name	Primer coordinates
ccrB-1	Cassette chromosome recombinase B, type 1	Cassette chromosome recombinase B allele found in SCC*mec* I, IX, SCC*fus* (as in MSSA476, GenBank BX571857.1) and in composite SCC*mec/fu* s elements	GU122149.1, 11999...13624	hp_ccrB-1_612<br>hp_ccrB-1_613	**GU122149.1 [13276:13300:r]**<br>**GU122149.1 [13442:13471:r]**	lb_ccrB-1_651_rv<br>lb_ccrB-1_652_rv	**CP000046.1 [47771:47788]**<br>**CP000046.1 [47594:47614]**
ccrB-2	Cassette chromosome recombinase B, type 2	Cassette chromosome recombinase B allele found in SCC*mec* II and IV elements	BA000033.2, 46367...47995	hp_ccrB-2_611	**BA000033.2 [46952:46979:r]**	lb_ccrB-2_651_rv<br>lb_ccrB-2_652_rv	**BA000017.4 [63916:63937]**<br>**DQ483074.1 [378:401:r]**
ccrB-3	Cassette chromosome recombinase B, type 3	Cassette chromosome recombinase B allele found in SCC*mec* III elements	AB037671.1, 6797...8425	hp_ccrB-3_611	**AB037671.1 [7406:7431]**	lb_ccrB-3_651_rv<br>lb_ccrB-3_652_rv	**AB014436.1 [2160:2179:r]**<br>**AB014436.1 [2265:2286:r]**
ccrB-4	Cassette chromosome recombinase B, type 4	Cassette chromosome recombinase B allele found in SCC*mec* VI and SCC*mec* VIII elements	AE015929.1, 58592...60220	hp_ccrB-4_611	**AE015929.1 [59605:59629:r]**	lb_ccrB-4_651_rv<br>lb_ccrB-4_652_rv	**AE015929.1 [59580:59600]**<br>**AE015929.1 [59457:59474]**
ccrC-85-2082	Cassette chromosome recombinase C	Cassette chromosome recombinase C allele found in in SCC*mec* V, SCC*mec* VT and SCC*mec* VII elements	AB037671.1, 6797...8425	hp_ccrC_611	**AB037671.1 [60667:60697:r]**	lb_ccrC_651_rv	**AB037671.1 [60643:60662]**
***copA2*- SCC**	copper exporting ATPase	Probe was designed for SCC-born alleles of that gene, as for instance in FPR3757, GenBank CP000255.1 or JCSC6943, GenBank AB505628.1	CP000255.1, 86055...88118	hp1385_copA2_SCC	**CP000255.1 [87564:87593]**	lb2458|copA2_SCC<br>lb2459|copA2_SCC	**CP000255.1 [87633:87650:r]**<br>**CP000255.1 [87201:87221:r]**
cstB-SCC1-Q2G1R6	CsoR-like sulfur transferase-regulated genes B/metallo-beta-lactamase superfamily protein. Pseudogene containing two stop codons	Subtyping SCC*mec* II (usually present, but absent in Irish SCC*mec* II variants A to E) and III (usually present, but absent in CMFT492 GenBank HF569112.1). Also present in SCC*mec* VIII and irregular elements such as *Staphylococcus fleurettii* GenBank AB546266	BA000017.4, 50957...52291	hp1324_Q2G1R6<br>hp1325_Q2G1R6	**BA000017.4 [51742:51768:r]**<br>**BX571856.1[51356:51381:r]**	lb2413|Q2G1R6<br>lb2414|Q2G1R6	**BA000017.4 [51708:51725]**<br>**BA000017.4 [51405:51426]**
cstB-SCC2-Q2G1R6	CsoR-like sulfur transferase-regulated genes B/metallo-beta-lactamase superfamily protein.	Present in SCC*mec* IVa (truncated) and SCC*mec* X, variably present in SCC*mec* I (usually present, but absent from MR1 GenBank ACZQ, Geraldine Clone) and VT. The corresponding probe was used in this study to distinguish SCC*mec* IVa from other SCCmec IV subtypes.	CP000046.1, 53428...54756	hp1221_Q2G1R6<br>hp1223_Q2G1R6	**CP000046.1 [54284:54313:r]**<br>**CP000046.1 [53882:53910:r]**	lb2601|Q2G1R6<br>lb2317|Q2G1R6	**CP000029.1 [2492569:2492590:r]**<br>**CP000046.1[53836:53854]**
czrC	cadmium and zinc resistance gene C, heavy metal translocating P-type ATPase	Frequently associated with SCC*mec* elements from livestock MRSA	AE015929.1, 64066...66000	hp1327_czrC<br>hp1328_czrC	**AE015929.1 [65056:65084]**<br>**AE015929.1 [65520:65547]**	lb2415|czrC<br>lb2583|czrC	**AE015929.1 [65088:65105:r]**<br>**AE015929.1 [65570:65587:r]**
**D1GU38**	Putative protein	Subtyping SCCmec III, identification of SCCmec VT, SCCmec ZH47, SCCmec VII because of an association with-additional/second ccrC copies	FN433596.1, 34888...35751	hp1214_D1GU38<br>hp1213_D1GU38	**FN433596.1 [35446:35474]**<br>**FN433596.1 [35040:35065]**	lb2308|D1GU38<br>lb2309|D1GU38	**FN433596.1 [35113:35134:r]**<br>**FN433596.1 [35517:35535:r]**
**D1GU55**	Putative membrane protein	Subtyping SCC*mec* III, additional marker for SCC*mec* VII	FN433596.1, 52909...53208	hp1501_D1GU55	**FN433596.1 [53056:53081]**	lb2516|D1GU55	**FN433596.1 [53098:53120:r]**
**D3JD07**	Putative protein	Present in some composite SCC elements such as, *e.g.* , M06/0171, GenBank HE980450.1 and 45394F, GenBank GU122149.1	GU122149.1, 8985...9797	hp1216_D3JD07<br>hp1217_D3JD07	**GU122149.1 [9581:9606:r]**<br>**GU122149.1 [9112:9146:r]**	lb2529|D3JD07<br>lb2312|D3JD07<br>lb2530|D3JD07	**GU122149.1[9542:9563]**<br>**HE980450.1[44440:44462:r]**<br>**GU122149.1[9066:9088]**
**Delta *mec* R1**	Truncated methicillin resist. operon repressor 1	Truncated *mec* R1 is present in SCC*mec* I, IV, V, VI, VII; complete absence of *mec* R1 from SCC*mec* V, IX, X	BA000033.2, 41708...42682	hp_mecR_611	**BA000033.2 [41812:41841]**	lb_mecR_651_rv	**CP000046.1 [41885:41906:r]**
**DUF1958**	Putative protein	Subtyping SCC*mec* VT. Present, *e.g.* , in PM1, GenBank BAFA but absent, *e.g.* , in Strain 3957, GenBank AOFU	GQ902038.2, 38193...38519	hp1218_DUF1958	**GQ902038.2 [38303:38331:r]**	lb2528|DUF1958	**GQ902038.2 [38254:38271]**
fusC-Q6GD50	SCC-associated fusidic acid resistance gene	Present alone in "SCC*fus* " elements or together with *mecA* in composite elements. Most common in CC1 and CC5 strains	BX571857.1, 52820...53458	hp_Q6GD50_611	**BX571857.1 [53173:53202]**	lb_Q6GD50_651_rv	**AF411935.1 [372:390]**
***kdpA*- SCC**	Potassium-translocating ATPase A, chain 2	Present in SCC*mec* II (although absent from Irish SCC*mec* II variants A to E)	BA000018.3, 77116...78792	hp_kdpA-SCC_611	**BA000018.3 [77635:77661]**	lb_kdpA-SCC_651_rv<br>lb_kdpA-SCC_652_rv	**BA000017.4 [77655:77675:r]**<br>**BA000017.4 [77890:77910:r]**
***kdpB*- SCC**	Potassium-transporting ATPase B, chain 1	Present in SCC*mec* II (although absent from Irish SCC*mec* II variants A to E)	BA000018.3, 78811...80832	hp_kdpB-SCC_611	**BA000018.3 [79775:79802]**	lb_kdpB-SCC_651_rv	**BA000017.4 [79776:79795:r]**
***kdpC*- SCC**	Potassium-translocating ATPase C, chain 2	Present in SCC*mec* II (although absent from Irish SCC*mec* II variants A to E)	BA000017.4, 80809...81366	hp_kdpC-SCC_612	**BA000017.4 [81035:81061:r]**	lb_kdpC-SCC_651_rv<br>lb_kdpC-SCC_652_rv	**BA000017.4 [80962:80980:r]**<br>**BA000017.4 [81011:81031]**
***kdpD*- SCC**	Sensor kinase protein	Present in SCC*mec* II (although absent from Irish SCC*mec* II variants A to E)	BA000018.3, 74179...76899	hp_kdpD-SCC_611	**BA000018.3 [76409:76436:r]**	lb_kdpD-SCC_651_rv	**BA000017.4 [76331:76349]**
***kdpE*- SCC**	KDP operon transcriptional regulatory protein	Present in SCC*mec* II (although absent from Irish SCC*mec* II variants A to E)	BA000018.3, 73509...74204	hp_kdpE-SCC_611	**BA000018.3 [73783:73808:r]**	lb_kdpE-SCC_651_rv	**BA000017.4 [73717:73735]**
***mco*- SCC**	Multi copper oxidase	Probe was designed for SCC-born allele(s of that gene, as for instance in JCSC6943, GenBank AB505628.1	AB505628.1, 35823...37256	hp1390_mco<br>hp1493_mco	**AB505628.1 [37177:37207]**<br>**AB505628.1 [36547:36574]**	lb2460|mco<br>lb2462|mco<br>lb2463|mco	**AB505628.1 [36608:36628:r]**<br>**AB505628.1 [37209:37229:r]**<br>**AB505628.1 [37209:37230:r]**
mecA	Modified penicillin binding protein-PBP2a	Modified penicillin binding protein-PBP2a causing oxacillin/methicillin resistance and thus defining MRSA	BA000017.4, 44992...46998	mecA_11<br>mecA-1,4	**BA000017.4 [45390:45417:r]**<br>**BA000017.4 [45264:45291:r]**	mecA_PM4<br>mecA_51	**CP000046.1 [39857:39876:r]**<br>**CP000046.1 [40007:40025]**
mecC	Alternate gene encoding a modified penicillin binding protein	Present in, and characteristic for, SCC*mec* XI	FR823292.1, 1741...3738	17_mecA_hp<br>25_mecA_hp	**FR823292.1 [3006:3032:r]**<br>**FR823292.1 [2830:2862:r]**	06_mecA_lp<br>16_mecA_lp	**FR823292.1 [2968:2988]**<br>**FR823292.1 [2764:2788]**
mecI	Methicillin-resistance regulatory protein	Present in SCC*mec* II (although absent from Irish SCC*mec* II variants C and E), SCC*mec* III, SCC*mec* VIII	BA000017.4, 48855...49226	hp_mecI_611	**BA000017.4 [49133:49162]**	lb_mecI_651_rv	**BA000017.4 [49169:49190:r]**
***mec* R1**	Methicillin resistance operon repressor 1	Un-truncated sequence in SCC*mec* II, SCC*mec* III, SCC*mec* VIII	BA000017.4, 47098...48855	hp_mecR_612	**BA000017.4 [48685:48711]**	lb_mecR_652_rv	**BA000017.4 [48726:48745:r]**

Gene/Marker	Gene product/Function	Comments	Reference sequence	Probe name	Probe coordinates	Primer name	Primer coordinates
merA	Mercury reductase	Parts of a mercury resistance operon that is plasmid born, although the plasmid can be integrated into SCCmec elements-for instance, in strains 85/2082, GenBank AB037671.1 or TW20, GenBank FN433596.1	AB037671.1, 38289...39932	hp_merA_611	**AB037671.1 [39354:39382:r]**	lb_merA_651_rv	**AB037671.1 [39315:39334]**
merB	Alkylmercury lyase		AB037671.1, 37557...38207	hp_merB_611	**AB037671.1 [38021:38046:r]**	lb_merB_651_rv	**AB037671.1 [38000:38018]**
***mvaS*- SCC**	Truncated 3-hydroxy-3-methylglutaryl CoA synthase	Subtyping SCC*mec* I, II, IV, V	BA000033.2, 37179...37531	hp1294_mvaS<br>hp1295_mvaS	**BA000033.2 [37280:37304]**<br>**BA000033.2 [37432:37460]**	lb2384|mvaS<br>lb2548|mvaS	**BA000033.2 [37307:37326:r]**<br>**BA000033.2 [37488:37510:r]**
opp3B	Oligopeptide permease, channel-forming protein	Part of ACME 1 and ACME 3 clusters that occurs alone or in combination with SCCmec elements. consensus probe	CP000255.1, 81950...82906	hp1296_opp3B	**ACSQ01000050.1 [4287:4313]**	lb2522|opp3B<br>lb2523|opp3B<br>lb2524|opp3B	**ACSQ01000050.1 [4334:4354:r]**<br>**AKHJ01000038.1 [36072:36092]**<br>**CP000255.1 [82101:82118:r]**
opp3B-C427		Specific probe for an allele known from coagulase-negatives	ACSQ01000050.1, 4183...5139	hp1297_opp3B	**ACSQ01000050.1 [4969:4997]**	lb2389|opp3B	**ACSQ01000050.1 [5005:5023:r]**
opp3B-FPR3757		Specific probe for an allele from the USA 300 CA-MRSA strain-FPR3757, GenBank CP000255.1 and TCH1516, GenBank CP000730.1 was used	CP000255.1, 81950...82906	hp1298_opp3B	**CP000255.1 [82736:82764]**	lb2390|opp3B	**CP000255.1 [82768:82785:r]**
opp3C-C427	Oligopeptide permease, channel-forming protein	Part of ACME 1 and ACME 3 clusters that occurs alone or in combination with SCC*mec* elements. Allele from known coagulase-negatives	ACSQ01000050.1, 5139...5906	hp1299_opp3C<br>hp1304_opp3C<br>hp1474_opp3C	**ACSQ01000050.1 [5150:5174]**<br>**ACSQ01000050.1 [5518:5548]**<br>**ACSQ01000050.1 [5259:5281]**	lb2391|opp3C	**ACSQ01000050.1 [5201:5222:r]**
opp3C-FPR3757	Oligopeptide permease, channel-forming protein	Part of ACME 1 and ACME 3 clusters that occurs alone or in combination with SCCmec elements. Allele from the USA 300 CA-MRSA strain-FPR3757, GenBank CP000255.1 and TCH1516, GenBank CP000730.1	CP000255.1, 82906...83673	hp1300_opp3C<br>hp1301_opp3C<br>hp1303_opp3C	**CP000255.1 [82917:82941]**<br>**CP000255.1 [83025:83048]**<br>**CP000255.1 [83286:83315]**	lb2392|opp3C	**CP000255.1 [82969:82988:r]**
pls-SCC-COL	Plasmin-sensitive surface protein, prevents bacterial adhesion in vitro, located in SCC, close to *mec* operon	Subtyping SCCmec I-Note: an additional, but unrecognised allele is also present in the irregular SCC element of WA MRSA-40: JQ746621.1	CP000046.1, 57212...61858	hp_plsSCC_611	**CP000046.1 [57330:57354]**	lb_plsSCC_651_rv	**CP000046.1 [57378:57398:r]**
**PSM-*mec***	Phenol soluble modulin from SCC*mec*	Present in SCCmec II-although absent from Irish SCCmec II variants C and E, SCCmec III, SCCmec VIII	BA000017.4, 49311...49379	hp1305_psmMEC	**BA000017.4 [49319:49347]**	lb2397|psmMEC	**BA000017.4 [49353:49372:r]**
**Q3YK51**	Putative protein	Subtyping SCC*mec* IV, *i.e*., identification of SCC*mec* IV g	DQ106887.1, 196...1944	hp1226_Q3YK51<br>hp1227_Q3YK51<br>hp1505_Q3YK51	**DQ106887.1 [1420:1447:r]**<br>**DQ106887.1 [902:931:r]**<br>**DQ106887.1 [1905:1929:r]**	lb2318|Q3YK51<br>lb2541|Q3YK51<br>lb2542|Q3YK51	**DQ106887.1 [1844:1867]**<br>**DQ106887.1 [1395:1417]**<br>**DQ106887.1 [862:884]**
**Q4LAG7**	Putative protein located within SCC*mec* type V/SCC*fus* elements	Identification of SCC*mec* V/VT elements and of SCC*fus* elements, consensus	AM990992.1, 50512...50940	hp1232_Q4LAG7	**AM990992.1 [50554:50583:r]**	lb2527|Q4LAG7<br>lb2526|Q4LAG7<br>lb2527|Q4LAG7	**AM990992.1[50527:50550]**<br>**BX571857.1[55466:55491]**<br>**JQ746621.1[47996:48019]**
		Identification of SCC*mec* V/VT elements		hp1229_Q4LAG7	**AM990992.1 [50702:50728:r]**	lb2518|Q4LAG7	**AM990992.1[50664:50682]**
		Identification of SCC*fus* elements	BX571857.1, 55452...55880	hp1228_Q4LAG7	**BX571857.1**	lb2519|Q4LAG7	**BX571857.1[55607:55627]**
**Q8CU82**	Putative protein	Present in some SCC*mec* /*fus* composite elements such as, *e.g.*, CMFT120, GenBank HF569094.1 and CMFT2, GenBank HF569101.1	AE015929.1, 32604...32786	hp1513_Q8CU82	**AE015929.1 [32671:32705]**	lb2557|Q8CU82<br>lb2562|Q8CU82	**AE015929.1 [32715:32735:r]**<br>**AE015929.1 [32713:32739:r]**
**Q933A2**	Putative ADP-ribosyltransferase	Subtyping SCC*mec* III and SCC*mec* IX	FN433596.1, 101805...102377	hp1240_Q933A2<br>hp1241_Q933A2	**FN433596.1 [102195:102222:r]**<br>**FN433596.1 [102284:102313:r]**	lb2331|Q933A2<br>lb2332|Q933A2	**FN433596.1 [102250:102268]**<br>**FN433596.1 [102164:102185]**
**Q93IB7**	LytTR domain DNA-binding regulator	Subtyping SCCmec III-present in, e.g., TW20 GenBank FN433596.1, but absent in, e.g., Bmb9393 GenBank CP005288.1 and IV-usually absent, but present in, e.g., CMFT503 GenBank HF569113.1	FN433596.1, 67873...68115	hp1242_Q93IB7	**FN433596.1 [434532:434550]**	lb2333|Q93IB7	**FN433596.1 [68041:68058]**
**Q9S0M4**	Putative protein	Subtyping SCC*mec* I, SCC*mec* /ACME composites and SCC*mec* from WA40	JQ746621.1, 10406...11456	hp1243_Q9S0M4	**JQ746621.1 [11192:11219:r]**	lb2334|Q9S0M4<br>lb2554|Q9S0M4	**JQ746621.1 [11164:11186]**<br>**JQ746621.1 [10490:10510]**

Gene/Marker	Gene product/Function	Comments	Reference sequence	Probe name	Probe coordinates	Primer name	Primer coordinates
Q9XB68-dcs	Located at the terminus of SCC*mec* directly next to *orfX*.	This locus comprises the downstream constant segment-dcs that in turn comprises a copy of the SCC direct repeat DR_SCC-AGAAGCTTATCATAAGTAA	*dcs* : CP000046.1, 34192…34371<br>Q9XB68: CP000046.1, 34372...35667	hp_Q9XB68_611<br>dcs_hp13	**CP000046.1 [34948:34976]**<br>**CP000046.1 [34224:34253]**	lb_Q9XB68_651_rv<br>dcs_rv12	**CP000046.1 [35004:35027:r]**<br>**CP000046.1[34269:34293:r]**
**SCC terminus 01**	SCC integration site alternate to *dcs*		GU235983.1, 488...808	sccterm_hp25	**GU235983.1 [568:594]**	lb2586|sccterm01	**FN433596.1 [67354:67380:r]**
**SCC terminus 02**	SCC integration site alternate to *dcs*		FN433596.1, 34140...34456	sccterm_hp27	**FN433596.1 [2708765:2708789:r]**	lb2585|sccterm12	**CP003808.1 [34446:34462:r]**
**SCC terminus 03**	SCC integration site alternate to *dcs*		FR753166.1, 481...568	sccterm_hp29	**FR753166.1 [649:672]**	lb2584|sccterm08	**HF569116.1 [461:480:r]**
**SCC terminus 04**	SCC integration site alternate to *dcs*		ACSW01000146.1, 46259...46316	sccterm_hp31	**ACSW01000146.1 [46381:46412]**	sccterm_rv32	**ACSW01000146.1 [46483:46505:r]**
**SCC terminus 05**	SCC integration site alternate to *dcs*		AB425427.1, 606...1027	sccterm_hp33	**AB425427.1 [754:779]**	sccterm_rv34	**AB425427.1 [800:824:r]**
**SCC terminus 06**	SCC integration site alternate to *dcs*	Associated mainly to SCCfus-as in MSSA476, GenBank BX571857.1	BX571857.1, 34169...34545	sccterm_hp35	**BX571857.1 [34282:34308]**	sccterm_rv36	**BX571857.1 [34321:34344:r]**
**SCC terminus 07**	SCC integration site alternate to *dcs*		GU122149.1, 119...222	sccterm_hp37	**GU122149.1 [182:215]**	sccterm_rv30	**GU122149.1 [23486:23508:r]**
**SCC terminus 09**	SCC integration site alternate to *dcs*		AB121219.1, 898...1198	sccterm_hp39	**AB121219.1 [1061:1088]**	sccterm_rv40	**AB121219.1 [1140:1163:r]**
**SCC terminus 10**	SCC integration site alternate to *dcs*		AB505630.1, 581...881	sccterm_hp21	**AB505630.1 [656:680]**	sccterm_rv22	**AB505630.1 [731:756:r]**
**SCC terminus 11**	SCC integration site alternate to *dcs*		HF569096.1, 746...1034	sccterm_hp23	**HF569096.1 [912:937]**	sccterm_rv24	**HF569096.1 [1007:1027:r]**
**SCC terminus 12**	SCC integration site alternate to *dcs*		CP003808.1, 34287...34414	hp1447_sccterm12	**CP003808.1 [34381:34406]**	lb2585|sccterm12	**CP003808.1 [34446:34462:r]**
**SCC terminus 13**	SCC integration site alternate to *dcs*		HF569114.1, 481...599	hp1448_sccterm13	**ARXY01000001.1 [131196:131224]**	lb2509|sccterm13<br>lb2510|sccterm13	**ARXY01000001.1 [131269:131294:r]**<br>**ARXY01000001.1 [131269:131294:r]**
**SCC terminus 14**	SCC integration site alternate to *dcs*		HF569093.1, 481...644	hp1449_sccterm14	**HF569093.1 [493:523]**	lb2511|sccterm14	**HF569093.1 [609:636:r]**
speG-FPR3757	Spermidine N-acetyltransferase	Usually associated with ACME or composite SCC*mec* /ACME elements	CP000255.1, 63100...63597	hp1496_speG	**CP000255.1 [63316:63341]**	lb2551|speG	**CP000255.1 [63356:63377:r]**
tirS	Staphylococcal TIR-protein binding protein	Subtyping SCC*fus* because it is frequently, but not always, accompanying *fusC*	BX571857.1, 50640...51482	hp1313_tirS<br>hp1514_tirS	**BX571857.1 [51339:51367]**<br>**BX571857.1 [51062:51094]**	lb2520|tirS<br>lb2521|tirS	**BX571857.1 [51103:51129:r]**<br>**BX571857.1 [51373:51394:r]**
ugpQ	Glycerophosphoryl diester phosphodiesterase	Accompanies *mecA* in all SCC*mec* sequences except SCC*mec* IV A from CN1, GenBank CP003979.1 and an irregular/composite element from *Staph. epidermidis* BCM-HMP0060, GenBank ACHE	BA000018.3, 43717...44460	hp_ugpQ_611	**BA000018.3 [44053:44078]**	lb_ugpQ_651_rv	**CP000046.1 [38701:38719:r]**
xylR/mecR2	Methicillin resistance operon repressor 2, Homolog of xylose repressor	Located next to *mec* operon downstream of *mecI* (not present if *mecI* is truncated). Present in SCC*mec* II although absent from Irish SCC*mec* II variants C and E, SCC*mec* III, SCC*mec* VIII	BA000018.3, 49738...50882	hp_xylR_611	**BA000018.3 [50152:50180]**	lb_xylR_651_rv	**BA000017.4 [50152:50171:r]**
ydhK-FPR3757	Putative lipoprotein	Present in some composite elements comprising SCC*mec* and heavy metal resistance genes including the one in FPR3757, GenBank: CP000255.1	CP000255.1 88136...88681	hp1469_ydhK<br>hp1470_ydhK<br>hp1471_ydhK	**CP000255.1 [88365:88389]**<br>**CP000255.1 [88590:88620]**<br>**CP000255.1 [88590:88618]**	lb2406|ydhK<br>lb2407|ydhK<br>lb2409|ydhK<br>lb2408|ydhK	**CP000255.1 [88405:88426:r]**<br>**CP000255.1 [88642:88662:r]**<br>**CP000255.1 [88352:88368:r]**<br>**CP000255.1 [88441:88462:r]**
yeeA	Putative DNA methyltransferase	Subtyping SCC*mec* IV/*fus* composite elements	HF569093.1, 2580...5294	hp1319_yeeA<br>hp1320_yeeA	**HF569093.1 [4397:4425:r]**<br>**HF569093.1 [3884:3908:r]**	lb2410|yeeA<br>lb2411|yeeA	**HF569093.1 [4366:4385]**<br>**HF569093.1 [3859:3879]**
